# Supplementary material for: Protocol for tumour-focused dose-escalated adaptive radiotherapy for the radical treatment of bladder cancer in a multicentre phase II randomised controlled trial (RAIDER): radiotherapy planning and delivery guidance
Source: BMJ Open. 2020 Dec 31;10(12):e041005. doi: 10.1136/bmjopen-2020-041005 (PMC7780718; doi:10.1136/bmjopen-2020-041005)
Supplement: Supplementary data [file bmjopen-2020-041005supp001.pdf]

A **R**andomised phase II trial of **A**daptive **I**mage guided standard or **D**ose **E**scalated tumour boost **R**adiotherapy in the treatment of transitional cell carcinoma of the bladder

PROTOCOL

Version: 3.0

Dated: 23/01/2019

Chief Investigator:	Prof Robert Huddart
Sponsor:	The Institute of Cancer Research
Approval:	Cancer Research UK: Clinical Trials Awards and Advisory Committee (CTAAC)
Funders:	Cancer Research UK
Coordinating Trials Unit:	ICR Clinical Trials and Statistics Unit (ICR-CTSU)<br>The Institute of Cancer Research

Main REC Reference Number:	15/LO/0539
ICR-CTSU Protocol Number:	ICR-CTSU/2014/10049
Sponsor’s identifier	CCR4249
ISRCTN:	26779187
ClinicalTrials.gov Identifier	NCT02447549
CRUK Reference Number:	CRUK/14/016

The RAIDER trial has been scientifically approved
by Cancer Research UK’s Clinical Trials Awards & Advisory Committee (CTAAC)
The RAIDER trial is part of the National Institute for
Health Research Clinical Research Network Trial Portfolio

This protocol is a controlled document and should not be copied, distributed or reproduced without the written permission of the ICR-CTSU. This document should only be used for the management of patients in the RAIDER trial.

Hafeez S, *et al. BMJ Open* 2020; 10:e041005. doi: 10.1136/bmjopen-2020-041005

RAIDER Protocol
ICR-CTSU

ADMINISTRATION

Clinical/Scientific Coordination

Prof Robert Huddart (Chief Investigator)
Academic Radiotherapy/Division of Radiotherapy and Imaging
The Royal Marsden NHS Foundation Trust/The Institute of Cancer Research
London
UK
Tel: 0208 661 3457
Robert.Huddart@icr.ac.uk

Dr Shaista Hafeez (Clinical Coordinator)
The Royal Marsden NHS Foundation Trust/The Institute of Cancer Research
London
UK

Tel: 0208 661 3274
Shaista.Hafeez@icr.ac.uk

Dr Farshad Foroudi (TROG lead)
Director Radiation Oncology
Austin Health
Melbourne
Australia

Trial Coordination

ICR-CTSU (a UKCRC registered NCRI cancer clinical trials unit) is responsible for the day to day conduct of the trial in the UK.

ICR Clinical Trials and Statistics Unit (ICR-CTSU),
Division of Clinical Studies,
The Institute of Cancer Research,
Sir Richard Doll Building,
Cotswold Road,
Sutton, Surrey SM2 5NG

ICR-CTSU Scientific/Methodology Lead: Emma Hall
Tel: 020 8722 4013
Emma.Hall@icr.ac.uk

ICR-CTSU Statistician: Clare Griffin
Tel: 0208 722 4187
RAIDER-icrctsu@icr.ac.uk

ICR-CTSU Clinical Trials Programme Manager: Rebecca Lewis
Tel: 020 8722 4081
RAIDER-icrctsu@icr.ac.uk

RAIDER Trial Manager
Tel: 020 8722 4613
RAIDER-icrctsu@icr.ac.uk

ICR-CTSU Data Manager: Chisomo Mutafya
Tel: 020 8722 4513
Chisomo.Mutafya@icr.ac.uk

Any questions relating to this protocol should be addressed in the first instance to the RAIDER Trial Manager within ICR-CTSU:

Email: RAIDER-icrctsu@icr.ac.uk
General enquiries: 020 8722 4613
Fax: 020 8770 7876

Hafeez S, *et al. BMJ Open* 2020; 10:e041005. doi: 10.1136/bmjopen-2020-041005

BMJ Publishing Group Limited (BMJ) disclaims all liability and responsibility arising from any reliance placed on this supplemental material which has been supplied by the author(s)

PROTOCOL DEVELOPMENT GROUP

Dr Robert Huddart	Chief Investigator	The Institute of Cancer Research/Royal Marsden NHSFT
Dr Emma Hall	Deputy Director (Research)	ICR-CTSU, The Institute of Cancer Research, London
Dr Shaista Hafeez	Consultant Oncologist	Institute of Cancer Research/ Royal Marsden NHSFT
Dr Ananya Choudhury	Consultant Oncologist	The Christie NHS Foundation Trust
Dr Ann Henry	Consultant Oncologist	Leeds University Teaching Hospitals
Dr Duncan McLaren	Consultant Oncologist	Western General Hospital, NHS Lothian
Dr Anita Mitra	Consultant Oncologist	University College Hospital, London
Dr Daniel Saunders	Consultant Oncologist	Nottingham University Hospitals NHS Trust
Mr Hugh Mostafid	Consultant Urologist	Hampshire Hospitals NHS Trust
Miss Joanne Cresswell	Consultant Urological Surgeon	South Tees Foundation Hospitals Trust
Dr Farshad Foroudi	Consultant Oncologist; ANZ Trans-Tasman Radiation Oncology Group Trial Chairperson	Austin Health, Melbourne
Dr Vibeke Hansen	Physicist	Royal Marsden NHS Foundation Trust
Prof Tomas Kron	Medical Physicist	Peter MacCallum Cancer Centre
Dr Helen McNair	Research Radiographer	Royal Marsden NHS Foundation Trust
Elizabeth Miles	NCRI Radiotherapy Trials QA Group (RTTQA) Co-ordinator	Mount Vernon Hospital
Emma Parsons	RTTQA Radiographer	Mount Vernon Hospital
Rebecca Lewis	Clinical Trials Programme Manager	ICR-CTSU, The Institute of Cancer Research, London
Clare Griffin	Medical Statistician	ICR-CTSU, The Institute of Cancer Research, London

The Trial Management Group (TMG) will be constituted from members of the Protocol Development Group and Principal Investigators from a subset of participating centres. A copy of the current membership of the TMG can be obtained from the RAIDER Trial Manager at ICR-CTSU.

Protocol Authorised by:

Name and Role	Signature	Date
Prof Robert Huddart (Chief Investigator)	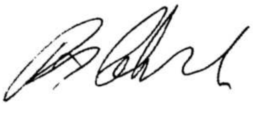	23/01/2019

This protocol describes the RAIDER trial and provides information about procedures for entering participants into this trial. The protocol should not be used as a guide for the treatment of patients outside of this trial.

Every care was taken in the preparation of this protocol, but corrections or amendments may be necessary. Protocol amendments will be circulated to participating sites as they occur, but sites entering patients for the first time are advised to contact ICR-CTSU to confirm they have the most recent version.

Hafeez S, *et al. BMJ Open* 2020; 10:e041005. doi: 10.1136/bmjopen-2020-041005

CONTENTS

1. INTRODUCTION 10
1.1. Background 10
1.1.1. Muscle invasive bladder cancer diagnosis and treatment 10
1.1.2. Challenges to bladder radiotherapy delivery 10
1.1.3. Image guided radiotherapy in bladder cancer 10
1.1.4. Concomitant radiosensitisation 11
1.1.5. Adaptive image guided radiotherapy 11
1.1.6. Tumour focused radiotherapy 12
1.1.7. Dose escalation 12
1.1.8. Tumour delineation – fiducial markers/diffusion weighted MRI 12
1.2. Known risks and benefits of adaptive tumour focused and dose escalated radiotherapy 12
1.2.1. Potential benefits 12
1.2.2. Potential risks 13
1.3. Study rationale 13
2. TRIAL OBJECTIVES 13
2.1. Stage I 13
2.1.1. Primary objective 13
2.1.2. Secondary objectives 13
2.2. Stage II 13
2.2.1. Primary objective 13
2.2.2. Secondary objectives 13
2.3. Exploratory objectives 14
2.3.1. IGRT related 14
3. TRIAL DESIGN 14
4. STUDY ENDPOINTS 14
4.1. Primary endpoint 14
4.1.1. Stage I 14
4.1.2. Stage II 14
4.2. Secondary endpoints 14
4.2.1. Stage I 15
4.2.2. Stage II 15
4.3. Exploratory endpoints 15
4.3.1. IGRT endpoints 15
5. PATIENT SELECTION AND ELIGIBILITY 15
5.1. Number of participants 15
5.2. Source of participants 15
5.3. Inclusion criteria 15
5.4. Exclusion criteria 16
5.5. Lifestyle guidelines 16
6. SCREENING 16
6.1. Screening log 16
6.2. Procedure for obtaining informed consent 16
6.2.1. RAIDER trial consent 17
6.3. Participation in other research 17
7. RANDOMISATION 17
8. TRIAL ASSESSMENTS 17
8.1. Pre-neoadjuvant chemotherapy assessments 17
8.2. Pre-randomisation assessments 18
8.3. Pre-radiotherapy assessments 18
8.4. On-treatment assessments 18
8.4.1. 32 fraction cohort 18

8.4.2. 20 fraction cohort 19
8.5. Post radiotherapy assessments 19
8.5.1. 10 weeks from start of radiotherapy 19
8.5.2. 3 months from last radiotherapy fraction 19
8.5.3. 6 months from last radiotherapy fraction 19
8.5.4. 9 months from last radiotherapy fraction 19
8.5.5. 12 months from last radiotherapy fraction 19
8.5.6. 18 months from last radiotherapy fraction 20
8.5.7. 24 months from last radiotherapy fraction 20
8.5.8. Annually to year 5 20
8.5.9. Annually thereafter 20
8.6. Procedure at disease progression/recurrence 20
8.7. Withdrawal from treatment or follow-up 20
9. SCHEDULE OF ASSESSMENTS 22
10. TREATMENT 23
10.1. Pre-trial treatment 23
10.2. Neo-adjuvant chemotherapy 23
10.3. Treatment timelines 23
10.4. Radiotherapy fractionation schedules 23
10.5. Radiotherapy planning and delivery 23
10.5.1. Group 1: standard Whole Bladder RT (WBRT) (control) 23
10.5.2. Group 2: Standard dose Adaptive tumour focused RT (SART) 23
10.5.3. Group 3: Dose escalated Adaptive tumour boost RT (DART) 24
10.6. Treatment scheduling and gaps 24
10.7. Concomitant therapy 24
10.8. Supportive care guidelines 24
11. RADIOTHERAPY QUALITY ASSURANCE (QA) 25
12. SAFETY REPORTING 25
12.1. Definitions 25
12.2. Reporting adverse events to ICR-CTSU 26
12.3. Reporting serious adverse events to ICR-CTSU 26
12.4. Serious adverse events exempt from expedited reporting 27
12.5. Review of serious adverse events 27
12.6. Expedited reporting of related unexpected SAEs 27
12.7. Follow up of serious adverse events 27
12.8. Annual safety reporting 27
12.9. Reporting pregnancies 27
12.10. Flow diagram for SAE reporting, and action following report 28
13. STATISTICAL CONSIDERATIONS 28
13.1. Statistical design and sample size justification 28
13.2. Treatment allocation 29
13.3. Endpoint definitions 30
13.3.1. Primary endpoints 30
13.3.2. Secondary endpoints 30
13.3.3. Exploratory endpoints 31
13.4. Statistical analysis plan 31
13.5. Interim analyses and stopping rules 32
14. TRIAL MANAGEMENT 33
14.1. Trial management group (TMG) 33
14.2. Trial steering committee (TSC) 33
14.3. Independent data monitoring committee (IDMC) 33
15. RESEARCH GOVERNANCE 34

15.1. Sponsor responsibilities 34
15.2. Participating site responsibilities 34
16. TRIAL ADMINISTRATION AND LOGISTICS 34
16.1. Site activation 34
16.2. Investigator training 34
16.3. Data acquisition 34
16.4. Central data monitoring 34
16.5. On-site monitoring 35
16.6. Completion of the study and definition of study end date 35
16.7. Archiving 35
17. PATIENT PROTECTION AND ETHICAL CONSIDERATIONS 35
17.1. Trial approvals 35
17.2. Trial conduct 35
17.3. Informed consent 35
17.4. Patient confidentiality 35
17.5. Data protection act (DPA) 36
17.6. Liability 36
18. FINANCIAL MATTERS 36
19. PUBLICATION POLICY 36
20. ASSOCIATED STUDIES 36
20.1. Patient reported outcome measures study 36
20.2. RAIDER translational sample collection 36
A1. WHO PERFORMANCE STATUS 38
A2. RTOG/EORTC LATE RADIATION MORBIDITY SCORING SCHEMA 38
A3. TUMOUR LOCALISATION GUIDELINES 39
A3.1 Fiducial marker placement 39
A3.2 Surgical bladder map 39
A3.3 Training and quality assurance 39
A4. PATIENT REPORTED OUTCOMES STUDY 40
A4.1 Background 40
A4.2 Hypotheses 40
A4.3 Quality of life measures 40
A4.4 Study design 40
A4.5 Timing of data collection 40
A4.6 Compliance 40
A4.7 Statistical considerations 41
A5. EXPECTED SERIOUS ADVERSE EVENTS 42
A6. TRANS TASMAN RADIATION ONCOLOGY GROUP SPECIFIC ADDENDUM 43
A6.1 Group specific committees and contacts 44
A6.1.1. TROG Trial Coordinating Centre 44
A6.2 Randomisation 44
A6.3 Safety reporting 44
A6.3.1. Reporting serious adverse events to TROG 44
A6.4 Review of serious adverse events 45
A6.5 Expedited reporting of related unexpected SAEs 45
A6.6 Follow up of serious adverse events 45
A6.7 Flow diagram for SAE reporting, and action following report 46
A6.8 Trial administration and logistics 46
A6.8.1. Site activation 46
A6.8.2. Investigator training 47
A6.8.3. Data acquisition 47
A6.8.4. Central data monitoring 47

Hafeez S, *et al. BMJ Open* 2020; 10:e041005. doi: 10.1136/bmjopen-2020-041005

A6.8.5. On-site monitoring 47
A6.8.6. Completion of the study and definition of study end date 47
A6.8.7. Archiving 47
A6.9 Patient reported outcomes study 47
A6.10 Financial matters 48
A6.11 Research governance 48
A6.11.1. Trial chairperson(s) 48
A6.11.2. Trial management committee 48
A6.11.3. Principal Investigator 48
A6.12 Patient protection and ethical considerations 48
A6.12.1. Aboriginal and Torres Strait Islander values and principles 48
A6.12.2. Insurance and compensation 48
A7. GLOSSARY 50
A8. REFERENCES 51

RAIDER TRIAL SUMMARY

PROTOCOL TITLE	A Randomised phase II trial of Adaptive Image guided standard or Dose Escalated tumour boost Radiotherapy in the treatment of transitional cell carcinoma of the bladder
TARGET DISEASE	Muscle invasive bladder cancer
STUDY OBJECTIVES	To define a feasible and safe adaptive dose escalated tumour boost radiotherapy schedule for MIBC; to investigate the ability to deliver daily adaptive bladder radiotherapy and assess the impact of delivery on patient reported outcomes and health economic related measures.
STUDY DESIGN	Multicentre two stage, three arm phase II randomised controlled trial
TRIAL POPULATION	Patients receiving radical radiotherapy for muscle invasive bladder cancer
RECRUITMENT TARGET	Minimum 120 in each of two fractionation cohorts i.e. sufficient to accrue 57 evaluable DART patients per cohort.
TRIAL TREATMENT	Patients will be randomised (1:1:2) between:<br>1. Standard whole bladder radiotherapy delivery (WBRT) (control)<br>2. Standard dose Adaptive tumour focused radiotherapy (SART)<br>3. Dose escalated Adaptive tumour boost radiotherapy (DART)<br>64Gy/32f and 55Gy/20f fractionation schedules are permitted. Participants in all groups will be permitted to receive concomitant radiosensitising therapy. Full blood count (FBC), urea and electrolytes (U&Es) and acute toxicity will be assessed during radiotherapy. Participants in the Patient Reported Outcomes (PRO) sub-study will be asked to complete a questionnaire prior to trial entry and at the end of radiotherapy.
PRIMARY ENDPOINT	Stage I: Proportion of patients meeting radiotherapy dose constraints to bladder, bowel and rectum in DART groups.<br>Stage II: Proportion of patients experiencing any ≥Grade 3 Common Terminology Criteria for Adverse Events (CTCAE) v.4 late toxicity (6-18 months post radiotherapy).
SECONDARY ENDPOINTS	Stage I:<br>• Recruitment rate<br>• Ability to deliver SART and DART<br>Stage II:<br>• Clinician reported acute toxicity<br>• PRO: acute and late bladder and bowel/rectal symptoms;<br>• Health economic related measures: time for outlining, plan generation, selection and delivery, NHS resource usage subsequent to treatment;<br>• Loco-regional MIBC control<br>• Progression-free survival<br>• Overall survival

EXPLORATORY ENDPOINTS	Image Guided Radiotherapy (IGRT) endpoints:<br>• Use of adaptive plans<br>• Target coverage<br>• Online/offline concordance<br>• Dose volume analysis of adaptive vs. standard planning
FOLLOW UP	Participants will subsequently be assessed at the following intervals:<br>**6 weeks from start of radiotherapy (20f cohort only)**<br>Assessment of acute toxicity (CTCAE v.4)<br>**10 weeks from start of radiotherapy:**<br>Assessment of acute toxicity (CTCAE v.4)<br>**3 months from end of radiotherapy:**<br>Rigid cystoscopy and biopsy of tumour bed, FBC, U&Es, chest x-ray (CXR), acute toxicity (CTCAE), PRO questionnaire (if participating in sub-study).<br>**6 months from end of radiotherapy:**<br>Flexible cystoscopy, FBC, U&Es, CXR or CT chest, CT abdomen and pelvis, late toxicity (CTCAE, RTOG), PRO (if participating in sub-study)<br>**9 months from end of radiotherapy:**<br>Flexible cystoscopy, late toxicity<br>**12 months from end of radiotherapy:**<br>Flexible cystoscopy, CT abdomen and pelvis, CXR or CT chest, late toxicity, PRO (if participating in sub-study)<br>**18 months from end of radiotherapy:**<br>Flexible cystoscopy, CXR or CT chest, late toxicity, PRO (if participating in sub-study)<br>**24 months from end of radiotherapy:**<br>Flexible cystoscopy, CT abdomen and pelvis, CXR or CT chest, late toxicity, PRO (if participating in sub-study)<br>**Yearly to year 5:** Flexible cystoscopy, CXR or CT chest, late toxicity<br>**Annually thereafter:** Survival and disease status

Hafeez S, *et al. BMJ Open* 2020; 10:e041005. doi: 10.1136/bmjopen-2020-041005

RAIDER Protocol
ICR-CTSU

TRIAL SCHEMA

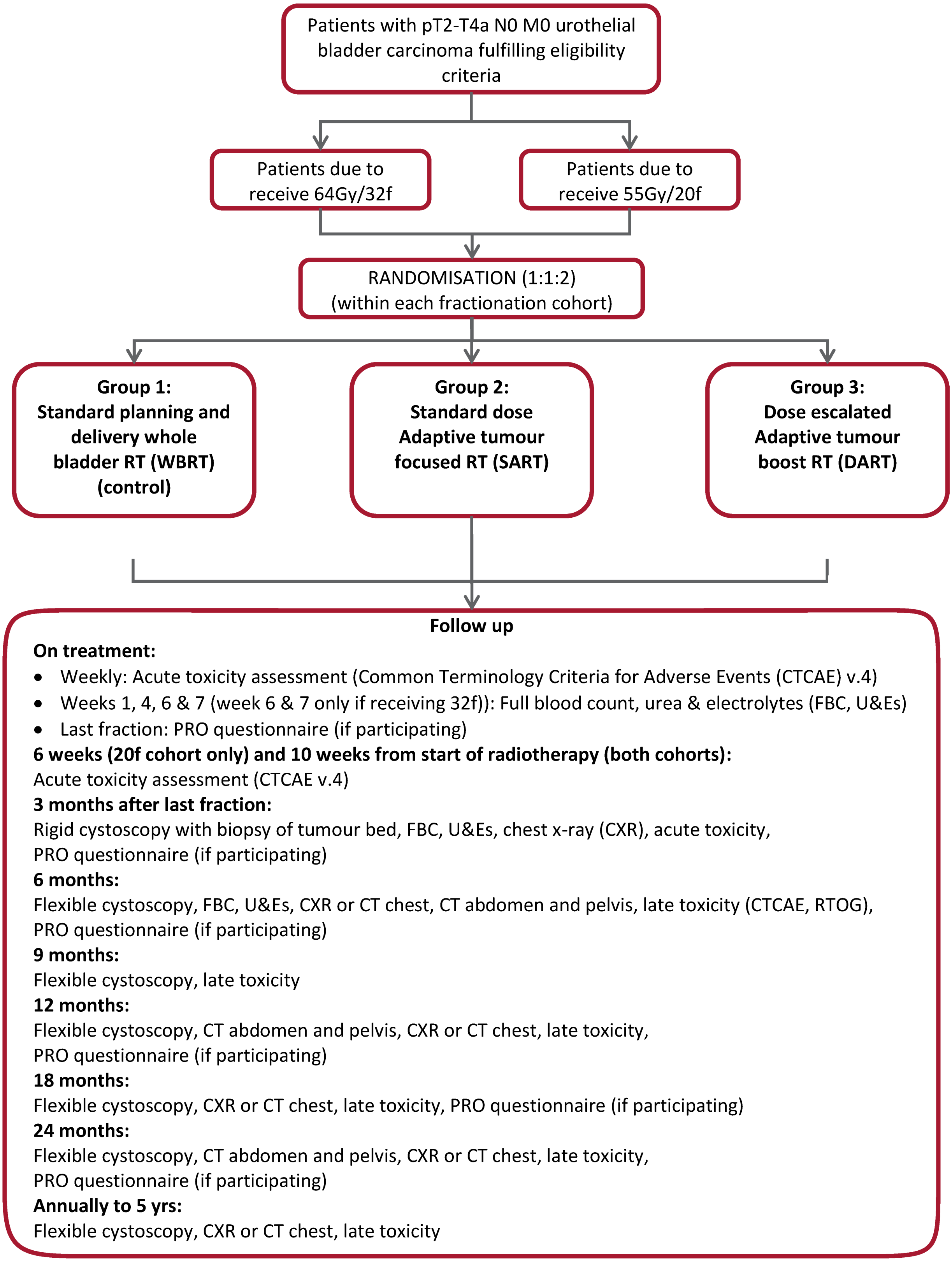

1. INTRODUCTION

1.1. Background

1.1.1. Muscle invasive bladder cancer diagnosis and treatment

Bladder cancer is the 7th most common UK cancer with 10,399 cases diagnosed in 2011 (1), and the 9th most common cancer in Australia, with an estimated 2,400 cases of muscle invasive disease in 2012 (2). Muscle invasive bladder cancer (MIBC) accounts for 25% of new tumour diagnoses and is associated with poor survival (<50% at 5 years)(3). Radical cystectomy is the "gold standard" therapy for MIBC(4), although a transurethral resection (TURBT) followed by daily radical radiotherapy (RT) is a recommended alternative, with similar rates of disease control to cystectomy. MIBC treatment, whether cystectomy or RT, can have high levels of associated side effects and relatively poor long term survival in comparison to some other cancer sites.

Though historically there have been concerns about high rates of recurrence following RT, the BC2001 trial demonstrated modern chemo-radiation can achieve results comparable to those of cystectomy. Two fractionation regimens are in common use within the UK: 64Gy in 32 fractions (f) over 6½ weeks (also commonly used internationally including in Australia/New Zealand) and 55Gy/20f over 4 weeks. To date these schedules are thought to be similar in efficacy. BC2001 included both 32f and 20f regimens and the 2 year local control rate for patients receiving chemo-radiation was over 65%, with only 18% of patients experiencing invasive recurrence at 2 years(5). These results mean that bladder sparing chemo-radiation is becoming a real alternative to surgery. With further development organ conserving treatment may replace radical surgery, as has been seen in breast, anal and head & neck cancer.

1.1.2. Challenges to bladder radiotherapy delivery

Radiotherapy is becoming accepted as a viable treatment option with good long term outcomes, but high dose radiation exposure can damage normal tissue, causing radiotherapy related toxicity. Patients receiving bladder radiotherapy are at particular risk from small bowel and rectal exposure. Though recent results are encouraging there remains room for improvement in minimizing toxicity*(5)*.

A course of standard radiotherapy is planned using a CT scan taken when the patient has an empty bladder. It is assumed that the initial scan is representative of bladder position throughout the course of treatment and radiotherapy delivery has traditionally been aligned using bony anatomy. To compensate for variations in bladder position, patients are treated with large safety margins added around the empty bladder (clinical target volume (CTV)) to create the planning target volume (PTV) to account for uncertainty introduced by microscopic disease not visible on the CT scan, errors in patient set up and day-to-day variation in bladder filling.

However the bladder is a mobile, deformable structure and bladder volume can vary markedly during a course of radiotherapy, despite delivering treatment to a perceived empty bladder (6-12). Movement of the bladder wall by more than 1.5cm has been documented in up to 60% of patients, resulting in inadequate coverage by radiotherapy fields in 33% of treatments (10). A study at the Royal Marsden Hospital (RMH) (13) reported that up to 57% of treatment may be delivered with some element of geographic miss (where the radiotherapy does not "hit" the tumour volume), despite employing safety margins of 1.5cm around the empty bladder (14). Geographical miss leads to the possibility of reduced tumour control, but larger margins would increase the treated volume and the amount of normal tissue exposed to high dose radiation, potentially leading to increased toxicity.

1.1.3. Image guided radiotherapy in bladder cancer

Recently, image guided RT (IGRT) technology such as cone beam CT imaging (CBCT) has allowed visualisation of soft tissue in the treatment room. Although of lower resolution than the original planning CT scan, these can be used both to match bony anatomy automatically and to visualise bladder position, thus helping to ensure that the PTV is correctly delivered and enabling development of adaptive IGRT to deliver RT with reduced safety margins, sparing normal tissue*(13-16)*. CBCT also allows the highest doses of RT to be reliably

focused on the tumour using intensity modulated RT (IMRT)*(13)*, allowing the remaining bladder to be treated at a lower dose*(17)*. Data suggest this technique may reduce the risk of genito-urinary toxicity by reducing exposure of normal bladder tissue to high doses of RT*(18)*,*(19, 20)*. Tumour focused RT also provides scope to increase the dose to which the tumour is exposed (dose escalation), whilst minimizing exposure of the remainder of the bladder. Targeted dose escalation has the potential to increase disease control for patients receiving bladder RT without increasing treatment toxicity.

The UK's ability to undertake image guided intensity modulated RT has recently expanded rapidly with all newly purchased RT machines being IGRT capable and IMRT being offered in 48 of 50 RT centres*(21)*. NHS England is prioritising the increase in capacity for delivery of IMRT and IGRT. Given the challenges of delivering RT to the bladder, the UK's National RT Implementation Group guidelines recommend routine use of CBCT to ensure the bladder is adequately targeted. The guidelines also note that the plan of the day adaptive IGRT technique discussed below has the potential to optimise the treatment of bladder cancer for patients*(22).*

In Australia CBCT is readily available in most radiotherapy centres. TROG 10.01 has demonstrated feasibility of adaptive image guided radiation therapy and in most centres that participated in the trial adaptive image guided radiotherapy is now standard of care for bladder cancer (23).

1.1.4. Concomitant radiosensitisation

The results of the multicentre phase III BC2001 (adding 5FU and mitomycin C to RT) (5) and BCON (hypoxic sensitization with carbogen and nicotinamide)(24) trials strongly suggest that a radiosensitisation approach should be recommended within RAIDER. Addition of low dose gemcitabine to RT has also been shown to achieve excellent local control rates in a phase II trial(25). Cisplatin was shown to be beneficial in the first randomised trial of chemo-radiation(26). There are no comparative data of the superiority of one radiosensitisation approach over another, though a recent paper has suggested the majority of benefit of carbogen is for patients with necrotic tumours(27).

1.1.5. Adaptive image guided radiotherapy

Availability of CBCT has led to the development of adaptive IGRT delivery strategies aimed at maintaining target coverage whilst reducing the amount of normal tissue irradiated. The most commonly described approaches uses a 'plan of the day' strategy where pre-treatment imaging is used to select the 'best fit' plan from a library of pre designed plans.

Selection of the best-fit plan ensures coverage of the CTV whilst minimising exposure of normal tissue in the PTV. Daily imaging with CBCT is required to permit appropriate plan selection based on bladder size and position. Published studies have varied approaches to creating a library of plans(16),(28-31). One study using a 64Gy/32f regimen reported a reduction of 29% in the mean volume of normal tissue irradiated to >45Gy compared to standard delivery bladder RT(16).

Plan of the day is being explored in the treatment of bladder cancer patients receiving weekly RT in the HYBRID trial (ISRCTN18815596). Participants will be randomised between standard and adaptive delivery techniques(32). 3 treatment plans, small, medium and large, will be generated during planning, with the most appropriate plan selected and verified by trained radiographers at time of each treatment delivery(33).

Additionally the Trans-Tasman Radiation Oncology Group (TROG) have completed a multi-centre feasibility study(23) investigating plan of the day adaptive bladder IGRT techniques using on-treatment CBCTs. This study incorporated rigorous RT quality assurance and recruited ahead of proposed timeline, demonstrating that this form of complex treatment delivery is acceptable to bladder cancer patients and a multicentre study is possible. Though in general the study was successful in the generation of acceptable adaptive plans on schedule, it failed to meet its preset goals for 'success' and judged to be not feasible in 31% of patients (due to use of conventional default plan (16%) and post treatment CTV outside PTV (18%))(23). Despite this it is noted that the treatment was well tolerated and the post treatment CTV was only outside the PTV in 5.5% of treatments. This is a substantial improvement over standard care though suggests some adjustment to adaptive protocols may be required.

1.1.6. Tumour focused radiotherapy

Targeting the highest RT dose to the tumour was investigated in a limited fashion in two UK randomised trials. BC2001 included a comparison of standard full dose whole bladder RT with a tumour focused treatment strategy(34). Bladder sparing in BC2001 was modest as it used a 1.5cm margin around the tumour and patients were treated with an empty bladder. CBCT had not yet been developed and treatment alignment was conducted using bony anatomy. 219 participants joined the RT comparison and no significant differences have been reported in late toxicity; with ~8% G3-4 RTOG toxicity in the tumour focused RT group at 2 years. There was no evidence to suggest an increase in recurrence in the tumour focused RT group. Similar findings were reported in a trial using 20f performed at the Christie NHSFT. Patients were randomised to whole bladder RT or RT to the tumour + margin only (57.5Gy/20f or 50Gy/16f). No significant differences in toxicity or local control were reported, although interpretation is limited due to the modest sample size and different radiation doses used for partial bladder RT(35).

1.1.7. Dose escalation

A single centre dose finding study, IDEAL(36), is investigating whether adaptive IGRT techniques allow tumour focused dose escalation. 54 patients had been treated to June 2014, with 21 receiving 68Gy/34f and 23 having 70Gy/32-35f. 30/54 patients received neoadjuvant chemotherapy prior to joining IDEAL and 41/54 received concurrent radiosensitising chemotherapy. With a median follow up of 18 months, only 2 episodes of G3 urinary toxicity and 1 invasive recurrence in dose escalated patients have been reported. IDEAL's final dose determined the 32f escalated dose in RAIDER.

The Christie trial dose escalated from 52.5Gy/20f to 57.5Gy/20f without evidence of excess toxicity(35). This study, co-investigator consensus and an α/β conversion of the likely dose resulting from IDEAL has been used to define the dose for the 20f dose escalated tumour boost in RAIDER.

1.1.8. Tumour delineation – fiducial markers/diffusion weighted MRI

Tumour delineation can be challenging, especially in those patients whose cancer responds well to neo-adjuvant chemotherapy; however the use of bladder maps (completed by surgeons at the time of TURBT) in combination with imaging was used with success in BC2001 and will be the minimum standard within RAIDER. There are also more advanced techniques of tumour definition now available. Diffusion weighted MRI (DWI) which assesses the mobility of water ions in tissues, is now widely available and used extensively in prostate cancer management. Cancers tend, being more cellular, to have a more restricted pattern of water mobility and can be distinguished from normal tissues. A prospective study at The Royal Marsden has demonstrated this is the case for localised bladder cancer and that DWI tumour definition and assessment of treatment response is highly correlated with results of cystoscopy/cystectomy. A Royal Marsden pilot study of target delineation(37) showed that DWI was a useful adjunct to conventional imaging and may add biological/functional information. 55/79 (69%) of patients had a definable tumour volume on MRI prior to radiotherapy; the remainder having had a complete TURBT with no visible tumour. A DWI defined GTV was around 50% smaller than the anatomically defined volume.

Bladder tumours can also be delineated using fiducial markers implanted at time of TURBT, particularly for those whose tumour is difficult to define radiologically. Initial work was with gold seeds(38) and more recently with Lipiodol (ethiodized oil)(39, 40). Fiducial insertion has proved to be safe and practicable and a similar technique would be recommended for use in RAIDER where possible.

1.2. Known risks and benefits of adaptive tumour focused and dose escalated radiotherapy

1.2.1. Potential benefits

It is anticipated that the use of adaptive radiotherapy techniques will improve the accuracy of treatment for patients in the adaptive groups which should lead to a reduction in side effects resulting in normal tissue exposure. Due to the highest radiotherapy dose being focused on the tumour, the remainder of the bladder will be exposed to lower levels of radiation which may also reduce the genito-urinary side effects experienced

by patients in the tumour focused groups. In addition, the patients in the dose escalated tumour boost group may benefit from better disease control as a result of the higher radiation exposure.

1.2.2. Potential risks

The toxicity of the dose escalated tumour boost may be higher than anticipated, however the tumour boost dose in both fractionation groups has been informed by the results of the IDEAL study (with α/β corrections to determine 20f dose). The primary endpoint of stage II is related to toxicity and rates will be monitored by the IDMC throughout the trial.

Participants in the SART and DART groups will receive one additional planning CT scan, however risks are anticipated to be minimal as it represents <1% of the RT dose.

Incorrect plan selection and tumour focused radiotherapy may result in increased risk of geographic miss, however appropriate plan selection will be part of the trial training program, will be verified by a 2nd trained observer prior to treatment delivery and will be monitored throughout the trial. In the IDEAL study with appropriate training a 91% on and offline plan concordance has been achieved with D98 post treatment coverage of 98.7%. Although prior studies have not shown that reduced radiation exposure of the uninvolved bladder increases risk of recurrence, patterns of recurrence and recurrence rates in both adaptive groups will be monitored by the IDMC.

1.3. Study rationale

Improving radiotherapy quality is of clear importance in bladder cancer treatment. RAIDER will assess whether adaptive dose escalated radiotherapy techniques developed at single centres can be successfully translated into radiotherapy practice across the UK, Australia and New Zealand and will prospectively assess the potential benefits of these approaches for patients as part of a multicentre international randomised trial.

RAIDER aims to define a feasible and safe RT schedule for MIBC using modern techniques and will include two fractionation cohorts which will be analysed separately but may provide data on the optimum fractionation schedule. RAIDER will seek to investigate whether modern techniques can allow an increase in the dose of RT to which the tumour is exposed and results will inform the design of a future phase III trial to establish the optimum organ preserving treatment option for patients with MIBC.

2. TRIAL OBJECTIVES

2.1. Stage I

2.1.1. Primary objective

The primary objective of stage I is to ensure that the dose escalated (DART) treatment can be planned and delivered at multiple centres within safe dose constraints.

2.1.2. Secondary objectives

Secondary objectives of stage I are to assess the recruitment rate and the ability of centres to deliver daily bladder SART and DART.

2.2. Stage II

2.2.1. Primary objective

Stage II aims to ensure the proportion of patients experiencing severe or medically significant late toxicity as a result of DART treatment is within acceptable limits.

2.2.2. Secondary objectives

Stage II secondary objectives are to assess clinician reported acute toxicity, and patient reported outcomes (PRO) of acute and late bladder and bowel/rectal symptoms. RAIDER will also investigate health economic related measures including time required for outlining, plan generation, selection and delivery and

healthcare resource usage subsequent to treatment. Disease related objectives include measuring loco-regional MIBC control, progression-free survival and overall survival.

2.3. Exploratory objectives

2.3.1. IGRT related

RAIDER will assess the utilisation of adaptive techniques including how often alternative plans are selected, the selection of appropriate plans and the target coverage and dose volume analysis of adaptive vs standard planning.

3. TRIAL DESIGN

RAIDER is an international multi-centre, multi-arm, two stage non-blinded phase II randomised trial of adaptive tumour focused radiotherapy for bladder cancer.

The trial includes three randomised groups and a 1:1:2 treatment allocation ratio has been used to provide participants with a 75% chance (on average) of receiving a novel radiotherapy technique. Primary endpoints will be assessed in each fractionation cohort separately. Stage I will test feasibility of DART treatment delivery by measuring compliance with dose constraints and stage II will assess late toxicity. The statistical analysis plan includes the flexibility to drop either a fractionation cohort or an experimental treatment group on the advice of the Independent Data Monitoring Committee following completion of stage I. Results will be used to select the RT technique to be employed in future national/international phase III bladder preserving trials.

All patients will receive radical bladder radiotherapy, delivered in either 20 or 32 fractions in accordance with participating centres' standard practice.

Participants allocated to the standard planning group will have one radiotherapy plan generated and this will be used to deliver all treatments, with a cone beam CT scan prior to treatment delivery which can be used by the local investigator to adjust treatment delivery according to local practice.

Participants allocated to Standard dose Adaptive tumour focused RT (SART) will have three radiotherapy plans generated; small, medium and large, with the highest RT dose focused on the tumour, sparing the remaining bladder from full dose radiation. IGRT will be used to select the most appropriate plan of the day.

Participants in the Dose escalated Adaptive tumour boost RT (DART) group will have three radiotherapy plans generated; small, medium and large, with a higher dose than standard targeted at the tumour and the remainder of the bladder treated to the same dose as in the SART group. IGRT will be used to select the most appropriate plan of the day.

Follow up visits will mirror standard practice wherever possible and will take place at 6 weeks (20f cohort only) and 10 weeks (both cohorts) following the start of radiotherapy, 3, 6, 9, 12, 18 and 24 months following the last fraction and annually to five years.

4. STUDY ENDPOINTS

4.1. Primary endpoint

The two fractionation cohorts will be analysed separately for the primary endpoints.

4.1.1. Stage I

- Proportion of participants meeting RT dose constraints in DART group

4.1.2. Stage II

- Late grade 3 or greater toxicity (CTCAE v4) occurring 6-18 months post RT.

4.2. Secondary endpoints

The two fractionation cohorts will be analysed separately and combined for the following secondary endpoints:

4.2.1. Stage I

- Recruitment rate
- Ability of centres to deliver SART and DART

4.2.2. Stage II

The two fractionation cohorts will be analysed separately and combined for the following secondary endpoints:

- Clinician reported acute toxicity (CTCAE v4)
- Patient reported outcomes (PRO) - acute and late bladder and bowel/rectal symptoms using the Patient-Reported Outcomes version of the Common Terminology Criteria for Adverse Events (PRO-CTCAE™), Assessment of Late Effects of RadioTherapy - Bowel (ALERT-B), the King's Health Questionnaire (KHQ), sexual function questions and the EQ5D-5L
- Health economic related measures - time for outlining, plan generation, selection and delivery, healthcare resource usage subsequent to study treatment

The two fractionation cohorts will be combined for the analyses of the following outcome measures:

- Loco-regional MIBC control
- Progression-free survival
- Overall survival

4.3. Exploratory endpoints

4.3.1. IGRT endpoints

- Use of adaptive plans
- Target coverage
- Online/offline concordance
- Dose volume analysis of adaptive vs. standard planning

5. PATIENT SELECTION AND ELIGIBILITY

5.1. Number of participants

The aim is to recruit a minimum of 120 participants to each fractionation cohort, i.e. sufficient to accrue 57 evaluable DART patients per cohort. In each cohort, at least 30 participants will be included in the standard planning group (control), at least 30 participants will be in the SART group and at least 60 participants will be allocated to the DART group.

5.2. Source of participants

Participants will be recruited from participating sites in the UK and Australia/New Zealand.

5.3. Inclusion criteria

1. Written informed consent
2. Age ≥16 years
3. Histologically or cytologically confirmed transitional cell carcinoma (TCC) of the bladder
4. Unifocal bladder TCC staged T2-T4a N0 M0*
5. Fit to receive a radical course of radiotherapy
6. WHO performance status 0-2 (See Appendix A1)

7. Willing and able to comply with study procedures and follow up schedule

* Tumour location must be clearly visible on imaging or recorded on a surgical bladder map

5.4. Exclusion criteria

1. Nodal or metastatic disease
2. Multifocal invasive disease
3. Simultaneous TCC in upper tract or urethra
4. Pregnancy
5. Active malignancy within 2 years of randomisation (not including non melanomatous skin carcinoma, previous non muscle invasive bladder tumours, NCCN low risk prostate cancer (T1/T2a, Gleason 6 PSA <10), in situ carcinoma of any site)
6. Bilateral hip replacements
7. Any other conditions that in the Principal Investigator's opinion would be a contra-indication to radiotherapy (e.g. previous pelvic radiotherapy/inflammatory bowel disease)

5.5. Lifestyle guidelines

It is highly unlikely that the patient population included in RAIDER will be at risk of pregnancy or fathering a child. However, if this is a possibility for any individual patient, this should be discussed and the patient should be advised to use barrier protection and avoid conception for 12 months after treatment.

6. SCREENING

6.1. Screening log

All participating centres will be required to keep a detailed log of all patients with muscle invasive bladder cancer who are considered for radical radiotherapy. This log will capture the following information:

- Date patient identified
- Number of patients approached/accepting/declining participation/ineligible
- Screening outcome
- Trial ID (if applicable)
- Reasons for ineligibility / not approaching / declining as applicable

This information will be used to monitor recruitment activity. No patient identifiable data will be collected at this stage.

6.2. Procedure for obtaining informed consent

The Principal Investigator (or designated individual) must ensure that each trial patient is fully informed about the nature and objectives of the trial and associated sub-studies and possible risks associated with participation. No protocol required assessments should be conducted until the appropriate consent form has been signed and dated by both the patient and the Investigator, unless they are performed routinely as part of standard patient care.

Confirmation of the patient's consent and the informed consent process must be documented in the patient's medical notes. A copy of the signed consent form(s) should be provided to the patient and the original retained in the investigator site file, which must be available for verification by ICR-CTSU study staff.

Hafeez S, *et al. BMJ Open* 2020; 10:e041005. doi: 10.1136/bmjopen-2020-041005

6.2.1. RAIDER trial consent

Participants should be given the current REC approved main RAIDER patient information sheet for their consideration. Patients should only be asked to consent to the study after they have had sufficient time to consider the trial and the opportunity to ask any further questions.

Patients who consent to RAIDER will be asked to consent to participate in the Patient Reported Outcomes (PRO) sub-study. Patients should be made aware that participation in the PRO sub-study is entirely voluntary. Refusal to participate in the PRO sub-study will not result in ineligibility to participate in the main clinical trial and will not impact the medical care received.

6.3. Participation in other research

Patients who fulfil the eligibility criteria will be given the opportunity to participate in RAIDER even if they have participated in other research prior to recruitment.

Participation in research whilst patients are being treated within RAIDER will be considered on a study by study basis by the Trial Management Group.

7. RANDOMISATION

Patients must be randomised centrally by the trials unit (ICR-CTSU) before trial treatment can commence. Patients should be randomised by telephoning ICR-CTSU on:

020 8643 7150

09.00-17.00 (UK time) Monday to Friday

Randomisation should take place within 10 weeks prior to the planned start date of radiotherapy. If planned radiotherapy timelines fall outside this window the ICR-CTSU should be contacted for advice prior to randomisation.

Treatment allocation will be by minimisation (with a random component). An eligibility and randomisation checklist must be completed prior to randomisation. Patients should only be randomised if sufficient trained and RTTQA accredited staff are available for plan selection in accordance with the RAIDER Radiotherapy Planning and Delivery Guidelines.

The following information will be required at randomisation:

- Name of treating and recruiting hospital, consultant and person randomising patient
- Confirmation that patient has given written informed consent for trial and for any sub-studies
- Confirmation that patient is eligible for the trial by completion of the eligibility checklist
- Patient's full name, hospital number, date of birth, postcode and NHS/CHI number

The caller will be given the patient's unique randomisation number (Trial ID) and treatment allocation (see section 14.2).

ICR-CTSU will send written confirmation of trial entry to the data management contact at the recruiting centre.

8. TRIAL ASSESSMENTS

8.1. Pre-neoadjuvant chemotherapy assessments

Information will be collected about the following assessments for RAIDER participants who have received neo-adjuvant chemotherapy:

- Radiological assessment of muscle invasive bladder cancer, ideally undertaken within 8 weeks prior to the start of neoadjuvant chemotherapy. If imaging was conducted outside the 8 week timeframe, the ICR-CTSU should be contacted for advice prior to randomisation. MRI pelvis and CT chest and

abdomen is recommended; the minimum acceptable is a chest, abdomen and pelvis CT or CT chest and CT urogram.

- TURBT with completion of bladder map† and optional placement of fiducial markers (if using, see Appendix A3)
- Histological confirmation of transitional cell carcinoma
- Full blood count, urea and electrolytes

† Bladder map not required if tumour is clearly visible on imaging.

Participants may be randomised into RAIDER whilst receiving neoadjuvant chemotherapy. Radiotherapy should be planned to commence within 10 weeks following completion of neo-adjuvant chemotherapy. If planned radiotherapy timelines fall outside this window the ICR-CTSU should be contacted for advice prior to randomisation.

8.2. Pre-randomisation assessments

For patients who have not received neo-adjuvant chemotherapy, the following assessments should be conducted prior to randomisation:

- Radiological assessment of muscle invasive bladder cancer within a maximum of 8 weeks prior to randomisation. If imaging was conducted outside the 8 week timeframe this should be repeated prior to randomisation. MRI pelvis and CT chest and abdomen is recommended; the minimum acceptable is a chest, abdomen and pelvis CT or CT chest and CT urogram.
- TURBT with completion of bladder map† and optional placement of fiducial markers (see Appendix A3)
- Histological confirmation of transitional cell carcinoma

† Bladder map not required if tumour is clearly visible on imaging.

8.3. Pre-radiotherapy assessments

For patients who have received neo-adjuvant chemotherapy the following assessments should be conducted within 4-6 weeks prior to the start of radiotherapy:

- Optional cystoscopy with placement of fiducial markers (if using)

The following assessments should be conducted for all participants within 2 weeks prior to the start of radiotherapy:

- Assessment of baseline symptoms (CTCAE v. 4)
- Full blood count, urea and electrolytes
- For participants who have consented to the patient reported outcomes (PRO) sub-study: Baseline PRO questionnaire (PRO-CTCAE, ALERT-B, KHQ, sexual function and EQ5D-5L)

8.4. On-treatment assessments

8.4.1. 32 fraction cohort

Weekly during treatment:

- Acute toxicity assessment (CTCAE v.4)

During weeks 1, 4 and 6 of radiotherapy:

- Full blood count, urea and electrolytes

At last fraction:

- Patient reported outcomes (PRO-CTCAE, ALERT-B, KHQ, sexual function and EQ5D-5L)

8.4.2. 20 fraction cohort

Weekly during treatment:

- Acute toxicity assessment (CTCAE v.4)

During weeks 1 and 4 of radiotherapy:

- Full blood count, urea and electrolytes

At last fraction:

- Patient reported outcomes (PRO-CTCAE, ALERT-B, KHQ, sexual function and EQ5D-5L)

6 weeks from start of radiotherapy

- Acute toxicity assessment (CTCAE v.4)

8.5. Post radiotherapy assessments

8.5.1. 10 weeks from start of radiotherapy

- Acute toxicity assessment (CTCAE v.4)

8.5.2. 3 months from last radiotherapy fraction

- Rigid cystoscopy and biopsy of tumour bed
- Full blood count, urea and electrolytes
- Chest x-ray
- Acute toxicity assessment (CTCAE v.4)
- Patient reported outcomes (PRO-CTCAE, ALERT-B, KHQ, sexual function and EQ5D-5L)

8.5.3. 6 months from last radiotherapy fraction

- Flexible cystoscopy
- Full blood count, urea and electrolytes
- CT of abdomen and pelvis
- Chest x-ray or CT chest
- Late toxicity assessment (CTCAE v.4 and RTOG (see Appendix A2))
- Patient reported outcomes (PRO-CTCAE, ALERT-B, KHQ, sexual function and EQ5D-5L). (Questionnaire administered to UK participants by ICR-CTSU.)

8.5.4. 9 months from last radiotherapy fraction

- Flexible cystoscopy
- Late toxicity assessment (CTCAE v.4 and RTOG (see Appendix A2))

8.5.5. 12 months from last radiotherapy fraction

- Flexible cystoscopy
- CT of abdomen and pelvis
- Chest x-ray or CT chest
- Late toxicity assessment (CTCAE v.4 and RTOG (see Appendix A2))
- Patient reported outcomes (PRO-CTCAE, ALERT-B, KHQ, sexual function and EQ5D-5L). (Questionnaire administered to UK participants by ICR-CTSU.)

Hafeez S, *et al. BMJ Open* 2020; 10:e041005. doi: 10.1136/bmjopen-2020-041005

8.5.6. 18 months from last radiotherapy fraction

- Flexible cystoscopy
- Chest x-ray or CT chest
- Late toxicity assessment (CTCAE v.4 and RTOG (see Appendix A2))
- Patient reported outcomes (PRO-CTCAE, ALERT-B, KHQ, sexual function and EQ5D). (Questionnaire administered to UK participants by ICR-CTSU.)

8.5.7. 24 months from last radiotherapy fraction

- Flexible cystoscopy
- CT of abdomen and pelvis
- Chest x-ray or CT chest
- Late toxicity assessment (CTCAE v.4 and RTOG (see Appendix A2))
- Patient reported outcomes (PRO-CTCAE, ALERT-B, KHQ, sexual function and EQ5D). (Questionnaire administered to UK participants by ICR-CTSU.)

8.5.8. Annually to year 5

- Flexible cystoscopy
- Chest x-ray or CT chest
- Late toxicity assessment (CTCAE v.4 and RTOG (see Appendix A2))

8.5.9. Annually thereafter

Data will be requested annually from standard follow up visits relating to:

- Assessment of disease status
- Survival

8.6. Procedure at disease progression/recurrence

Participants should be treated according to local clinical judgement at disease progression/recurrence. Patients with local or pelvic recurrence should continue to be followed up per protocol.

Following any metastatic recurrence (stage M1a/M1b), data will be requested six monthly from routine visits regarding:

- Assessment of disease status
- Survival

8.7. Withdrawal from treatment or follow-up

Participants may withdraw from trial treatment at any time at their own request, or they may be withdrawn at the discretion of the Principal Investigator. Reasons for withdrawal may include:

- Disease progression
- Unacceptable toxicity
- Co-morbidities

Participants who discontinue treatment should continue to be followed up.

If a patient withdraws from further follow-up, a trial deviation form should be submitted to ICR-CTSU stating whether the patient has withdrawn consent for further information to be sent to the ICR-CTSU or whether

they simply no longer wish to attend trial follow up visits. In the very rare event that a patient requests that their data is removed from the study entirely, the implications of this should be discussed with the patient first to ensure that this is their intent and, if confirmed, ICR-CTSU should be notified in writing. The patient should be made aware that any information about them that has already been published or submitted for safety monitoring purposes cannot be withdrawn.

RAIDER Protocol
ICR-CTSU

9. SCHEDULE OF ASSESSMENTS

Visit/Assessment	Pre-neoadjuvant chemotherapy (if given)	Pre-randomisation	Pre-radiotherapy	On treatment	6 weeks after start RT[¥]	10 weeks after start RT	3 months after end RT	6 months after end RT	9 months after end RT	12 months after end RT	18 months after end RT	24 months after end RT	Annually to 5 years	Annually thereafter	At recurrence/ disease progression
Radiological assessment*	X	X												Disease status and survival	Treatment according to local practice. Continue folllow up per protocol after pelvic recurrence. Follow up for disease status and survival after metastatic recurrence
TURBT with completion of bladder map	X	X													
Placement of fiducial markers (optional)	X	X	X[1]												
Assessment of symptoms/toxicity			X[2]	X[2,3]	X[2]	X[2]	X[2]	X[6]	X[6]	X[6]	X[6]	X[6]	X[6]		
Full blood count, urea and electrolytes	X		X	X[4]			X	X							
PRO questionnaire (if participating)			X	X[5]			X	X[7]		X[7]	X[7]	X[7]			
Rigid cystoscopy and biopsy of tumour bed							X								
Chest x-ray							X								
Flexible cystoscopy								X	X	X	X	X	X		
CT of abdomen and pelvis								X		X		X			
Chest x-ray or CT chest								X		X	X	X	X		
Health resource utilisation				X			X	X		X	X	X			

Footnotes

* Recommended imaging: MRI pelvis, CT chest and abdomen. Minimum acceptable is chest, abdomen, pelvis CT or CT chest and CT urogram
† For patients who have not received neo-adjuvant chemotherapy
¥ For patients in the 20f cohort only
1. For patients who received neo-adjuvant chemotherapy
2. CTCAE v.4
3. Weekly on treatment
4. During weeks 1, 4 and 6 (week 6 only if receiving 32f)
5. At last fraction
6. CTCAE v.4 and RTOG
7. Questionnaires administered to UK participants by ICR-CTSU from 6 months onwards

10. TREATMENT

10.1. Pre-trial treatment

All participants should have a transurethral resection of bladder tumour (TURBT) with completion of bladder tumour map by the urologist performing the procedure. Placement of fiducial markers is recommended either during TURBT or at cystoscopy following neo-adjuvant chemotherapy (see Appendix A3).

10.2. Neo-adjuvant chemotherapy

Neo-adjuvant chemotherapy prior to randomisation according to local practice is permitted. Details will be collected on the relevant case report form.

10.3. Treatment timelines

Radiotherapy should commence within 10 weeks following randomisation or completion of neoadjuvant chemotherapy (if used), to allow sufficient time for planning. If planned radiotherapy timelines fall outside this window the ICR-CTSU should be contacted for advice prior to randomisation.

10.4. Radiotherapy fractionation schedules

Two fractionation schedules are permitted: 32 fractions or 20 fractions. Centres will specify their intended fractionation schedule prior to trial initiation and this should be used to treat all RAIDER participants throughout the trial.

10.5. Radiotherapy planning and delivery

Details of radiotherapy planning are provided in the accompanying RAIDER Radiotherapy Planning and Delivery guidelines, available for UK sites on the Radiotherapy Trials Quality Assurance (RTTQA) website (http://www.rttrialsqa.org.uk/rttqa/) and for sites in Australia and New Zealand on the TROG cancer research RAIDER page (http://trog.com.au/TROG-1402-RAIDER-trial-documents). The current version of the RAIDER radiotherapy planning and delivery guidelines must be used as the primary source for planning and delivering radiotherapy treatment within RAIDER.

10.5.1. Group 1: standard Whole Bladder RT (WBRT) (control)

Radiotherapy will be delivered on an empty bladder. One treatment plan will be generated from the planning CT scan taken immediately after voiding (CT0). 64Gy/32f or 55Gy/20f RT will be given daily for 6 ½ or 4 weeks respectively. Pre-treatment CBCT should be conducted for treatment verification.

10.5.2. Group 2: Standard dose Adaptive tumour focused RT (SART)

RT will be delivered on a partially full bladder. 2 planning CTs will be taken at 30 (CT30) and 60 (CT60) minutes after urination and drinking 350 mls water. 2 target volumes will be defined:

GTV= bladder tumour/tumour bed and extravesical spread.

CTV = GTV +whole bladder and extravesical spread

These volumes will be used to create 3 PTVs as follows:

PTVsmall or PTVmedium or PTVLarge = CTV expanded + corresponding PTV2

Where PTV2 = GTV+ 0.5cm isotropic margin for PTV2small and GTV + anisotropic margin for both PTV2medium and PTV2Large

If filling occurs between CT30 and CT60 (difference in CTV>50 mls), the PTV large will be defined from outlines derived from CT60.

PTV1 will be treated to at least 52Gy/32f or 46Gy/20f (+/-5%) and PTV2 to 64Gy/32f or 55Gy/20f. Treatment will be planned using forward planned IMRT, inversed planned IMRT, VMAT or tomotherapy. Use of alternative techniques will require specific approval from the RAIDER TMG and QA team. Centres will be

asked to specify their preferred method of treatment delivery and complete the appropriate Quality Assurance program.

Prior to each fraction, a CBCT will be performed and the optimal plan will be selected for that day's treatment by an accredited individual and verified by a second trained individual.

10.5.3. Group 3: Dose escalated Adaptive tumour boost RT (DART)

Plans and treatment delivery technique will be as for group 2 except an escalated dose will be given to the tumour boost volume (PTV2) of 70Gy/32f or 60Gy/20f.

If normal tissue dose constraints for escalation are not met for the medium plan, with the exception of 'other bowel' V45 and/or V50 (V37.5 and/or V41.7 for 20 fraction treatments), planning data should be provided to the RTTQA team prior to treatment to enable prospective central review by an accredited member of the Trial Management Group. If dose constraints are not met following central review, treatment at standard dose (as group 2) is recommended (following discussion with the RTTQA team).

10.6. Treatment scheduling and gaps

Treatment can start on any day of the week and should be given five days a week until completion.

Delays and treatment gaps should be avoided, however if gaps occur please refer to the RAIDER radiotherapy planning and delivery guidelines for further information. If any issues arise during RAIDER participants' treatment, ICR-CTSU and the RTTQA team should be contacted in real time for guidance.

10.7. Concomitant therapy

Participants in all groups will be permitted to receive concomitant radiosensitising therapy, the BC2001 MMC/5FU regimen or gemcitabine, carbogen or cisplatin.

Any other regimens in standard use at participating centres will require approval by the Trial Management Group. Centres should aim to use the same regimen for all patients receiving radiosensitising treatment throughout the trial. If the patient isn't fit for the centre's usual radiosensitising treatment an alternative may be substituted after discussion with the RAIDER trial manager.

10.8. Supportive care guidelines

All medication considered necessary for the patients' welfare and which is not expected to interfere with the evaluation of the treatment may be given at the discretion of the investigator.

In the event of patient catheterisation during the course of treatment it is expected that the participant will continue and complete radiotherapy in accordance with their allocated treatment group. For patients in group 1 (WBRT), as the bladder requires emptying prior to treatment delivery, the catheter must be on free flow in circumstances where there is a leg bag or voided in circumstances where there is a flip-valve. For patients in groups 2 and 3 (SART and DART), the catheter should be clamped 30 minutes before treatment (if possible).

Participants' symptoms should be managed according to local practice, although the following are suggestions for patient care:

Anaemia: Patients should be maintained by transfusion with haemoglobin above 11 grams. Iron deficiency should be treated with iron supplementation.

Dysuria/Frequency: Check for evidence of infection and treat if present with appropriate antibiotics, anticholinergics (eg oxybutynin, tolterodine), NSAIDs, analgesics.

Diarrhoea: Loperamide or opioid

Proctitis: steroid suppository +/- local anaesthetics (e.g. sheriproct, proctosedyl)

Hafeez S, et al. BMJ Open 2020; 10:e041005. doi: 10.1136/bmjopen-2020-041005

11. RADIOTHERAPY QUALITY ASSURANCE (QA)

A comprehensive QA programme for the RAIDER trial will be designed and implemented by the NCRI Radiotherapy Trials Quality Assurance (NCRI RTTQA) group (UK) and TROG QA group (Australia/NZ). This will include pre-trial and on-trial components. For full details of the QA programme refer to the RAIDER Radiotherapy Planning and Delivery Guidelines.

12. SAFETY REPORTING

12.1. Definitions

Adverse Event (AE)
An AE is any untoward medical occurrence in a patient administered a study treatment; the event does not necessarily have a causal relationship with the treatment.

Serious Adverse Event (SAE)
An SAE is any untoward medical occurrence that occurs after the commencement of radiotherapy and within 30 days of the last fraction of radiotherapy and:

- results in death,
- is life-threatening
- requires hospitalisation or prolongation of existing inpatients´ hospitalisation
- results in persistent or significant disability or incapacity
- is a congenital anomaly or birth defect
- is a dose limiting (grade 4) toxicity

In addition, between 6 and 18 months following completion of radiotherapy the following should be reported as an SAE:

- Radiotherapy related grade 3, 4 or 5 events

Important adverse events that are not immediately life-threatening or do not result in death or hospitalisation but may jeopardise the subject or may require intervention to prevent one of the other outcomes listed in the definition above, may also be considered serious.

Progression of the indicated disease and death due to progression of the indicated disease are not considered SAEs.

Pregnancy or aid in the conception of a child whilst participating in a trial is not itself considered an SAE but should be followed up for congenital anomalies or birth defects.

Serious Adverse Reaction (SAR)
A serious adverse reaction is an SAE that is suspected as having a causal relationship to the trial treatment, as assessed by the investigator responsible for the care of the patient. A suspected causal relationship is defined as possibly, probably or definitely related (see definitions of causality table).

Hafeez S, et al. BMJ Open 2020; 10:e041005. doi: 10.1136/bmjopen-2020-041005

Definitions of causality

Relationship	Description
Unrelated	There is no evidence of any causal relationship with the trial treatment
Unlikely	There is little evidence to suggest there is a causal relationship (e.g. the event did not occur within a reasonable time after administration of the trial treatment). There is another reasonable explanation for the event (e.g. the patient's clinical condition, other concomitant treatment)
Possible	There is some evidence to suggest a causal relationship (e.g. because the event occurs within a reasonable time after administration of the trial treatment). However, the influence of other factors may have contributed to the event (e.g. the patient's clinical condition, other concomitant treatments)
Probable	There is evidence to suggest a causal relationship, and the influence of other factors is unlikely
Definitely	There is clear evidence to suggest a causal relationship, and other possible contributing factors can be ruled out
Not assessable	There is insufficient or incomplete evidence to make a clinical judgement of the causal relationship.

Related Unexpected Serious Adverse Event
An adverse event that meets the definition of serious and is assessed by the CI or nominative representative as:

- "Related" – that is, it resulted from administration of any of the research procedures, and
- "Unexpected" – that is, the type of event is not listed in the protocol as an expected occurrence (see Appendix A5)

12.2. Reporting adverse events to ICR-CTSU

Any toxicity, sign or symptom that occurs after commencement of study treatment which is not unequivocally due to progression of disease, should be considered an AE.

All AEs must be reported on the relevant toxicity, sign or symptom CRF.

The severity of AEs should be graded according to CTCAE v4 criteria. For each AE, the highest grade observed since the last visit should be reported.

Whenever one or more toxicity/sign/symptom corresponds to a disease or a well-defined syndrome only the main disease/syndrome should be reported.

12.3. Reporting serious adverse events to ICR-CTSU

Any SAE (except those listed below) that occurs from the start of radiotherapy and up to 30 days following the last day of radiotherapy must be reported. In addition, any radiotherapy related grade 3, 4 or 5 events occurring between 6 and 18 months after completion of radiotherapy must be reported.

All SAEs should be reported to ICR-CTSU within 24 hours of the Principal Investigator (or designated representative) becoming aware of the event, by completing the RAIDER SAE form and faxing to:

The ICR-CTSU safety desk
Fax no: **0208 722 4368**
For the attention of the RAIDER Trial team

As much information as possible, including the Principal Investigator's assessment of causality, must be reported to ICR-CTSU in the first instance. Additional follow up information should be reported as soon as it is available.

All SAE forms must be completed, signed and dated by the Principal Investigator or designated representative.

The Site SAE log should be completed and the SAE form filed in the Site Investigator File.

12.4. Serious adverse events exempt from expedited reporting

The expected adverse events listed in Appendix A5 are exempt from expedited reporting if grade ≤2 but should be reported using the appropriate CRF.

12.5. Review of serious adverse events

The Chief Investigator (or designated representative) will assess all reported SAEs for causality and expectedness (NB. The Chief Investigator cannot down-grade the Principal Investigator's assessment of causality.)

SAEs assessed as having a causal relationship to study treatment and as being unexpected will undergo expedited reporting to the relevant authorities and all other interested parties by ICR-CTSU (see 12.6).

Sites should respond as soon as possible to requests from the Chief Investigator or designated representative (via ICR-CTSU) for further information that may be required for final assessment of an SAE.

12.6. Expedited reporting of related unexpected SAEs

If an SAE is identified as being related and unexpected by the Chief Investigator it will be reported by ICR-CTSU to the main REC, the Sponsor and all other interested parties within 15 days of being notified of the event.

The Principal Investigators at all actively recruiting sites will be informed of any related unexpected SAEs occurring within the trial at appropriate intervals.

The collaborative group in each participating country will report related unexpected SAEs as per their local requirements to IECs and local investigators.

12.7. Follow up of serious adverse events

SAEs should be followed up until clinical recovery is complete or until the condition has stabilised. SAE outcomes should be reported to ICR-CTSU using the relevant section of the SAE form as soon as the Principal Investigator or designee becomes aware of the outcome.

12.8. Annual safety reporting

An annual progress report will be provided to the main REC by ICR-CTSU and copied to the Sponsor and the collaborative group in each participating country at the end of the reporting year. This will include data about related unexpected SAEs and whether any safety concerns have arisen during the reporting period.

12.9. Reporting pregnancies

If any trial participant or a trial participants' partner becomes pregnant while receiving trial treatment or up to 90 days after receiving trial treatment, this should be reported to ICR-CTSU using the pregnancy reporting form. Participants who become pregnant should discontinue from trial treatment immediately. Pregnancies should be followed up until conclusion and all follow-up information should be reported to ICR-CTSU. If the outcome of the pregnancy meets the definition of serious (i.e. congenital abnormality) this should be reported to ICR-CTSU following the serious adverse event reporting procedures described above.

Hafeez S, *et al. BMJ Open* 2020; 10:e041005. doi: 10.1136/bmjopen-2020-041005

12.10. Flow diagram for SAE reporting, and action following report

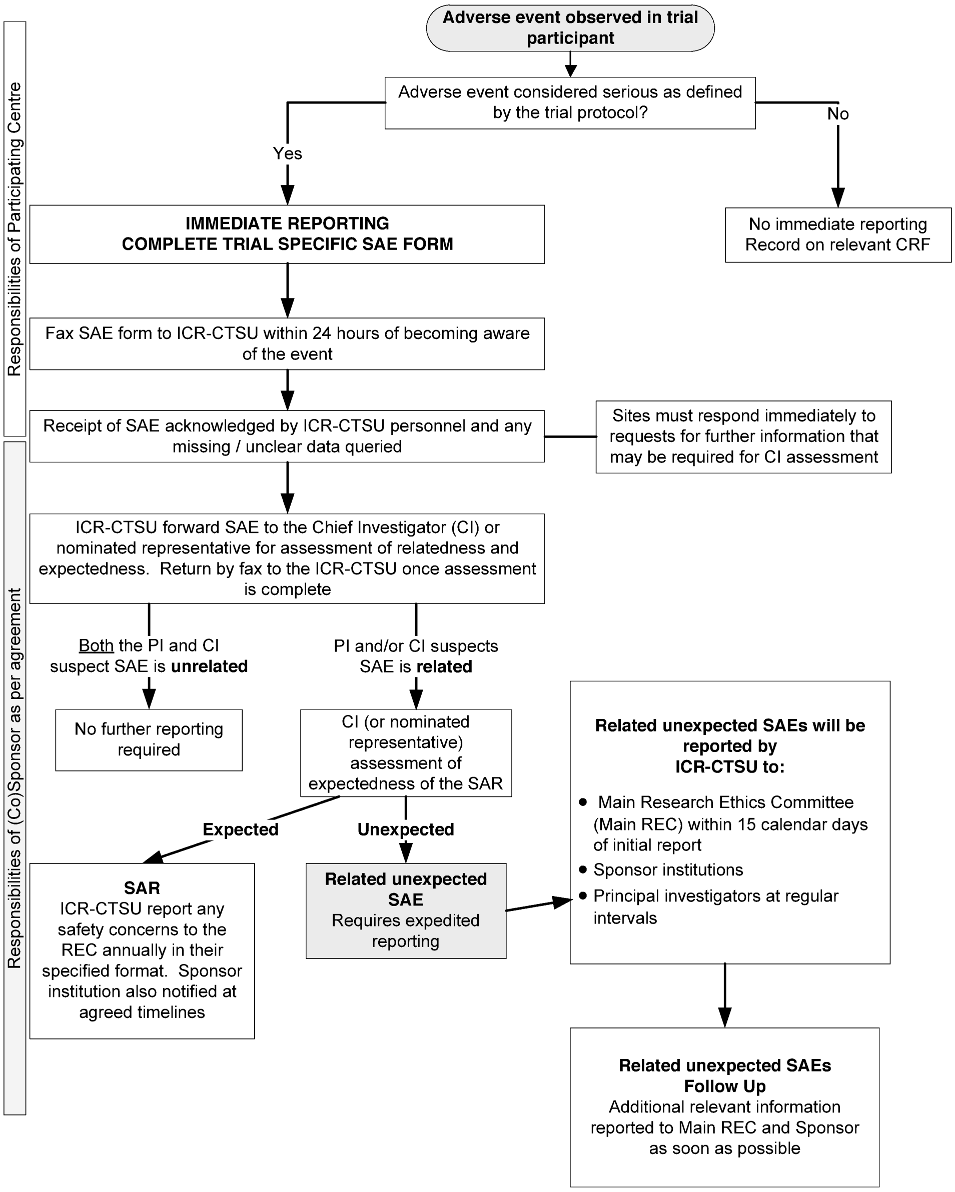

NB. All SAEs should continue to be followed up as specified above

13. STATISTICAL CONSIDERATIONS

13.1. Statistical design and sample size justification

Stage I

Stage I will assess the technical feasibility of delivering DART in a multi-centre setting. Dose constraints will be based on those in the IDEAL trial (36) and predefined by consensus of the co-investigators. Dose constraints will be detailed in the RAIDER radiotherapy planning and delivery guidelines. It is expected that 80% of patients in each DART fractionation cohort will meet dose constraints (as defined in 12.3.1). If less than 50% meet dose constraints then it will be concluded that treatment delivery is not feasible. Using an

A'Hern single stage design (p0=0.5, p1=0.8, 5%α, 80% power) 18 patients are required in each DART cohort. If at least 13/18 meet dose constraints it will be concluded that treatment is feasible. 36 patients in each fractionation cohort will be randomised (1:1:2 ratio) between control, SART and DART groups. Stage I will therefore require a total of 72 patients. The control and SART groups are included to enable SART to be carried forward to stage II if dose constraints cannot be met in the DART group. It also allows the assessment of equipoise and feasibility of recruitment for any subsequent phase III trial.

Assuming dose constraints are met, stage II will determine whether dose escalated RT can be delivered without detriment to long term toxicity within each fractionation cohort. At the end of stage I, the IDMC will review recruitment and toxicity data and will advise on any adaptions to trial design (e.g. unexpected toxicity in an DART fractionation cohort may lead one fractionation to be dropped; if dose constraints are consistently met for DART the SART group could be dropped for stage II; the overall sample size could be inflated to adjust for dose constraint non-compliance seen in stage I). Recruitment to stage II will continue seamlessly whilst stage I is evaluated, unless advised otherwise by the IDMC.

Stage II

Stage II has a non-comparative design aiming to rule out an upper limit of any late ≥G3 CTCAE toxicity in each DART fractionation cohort. It is expected that the proportion of patients in the control group reporting ≥G3 CTCAE toxicity between 6-18 months post-radiotherapy will be 8% (34). With 57 evaluable patients in each DART fractionation cohort, we can exclude >20% G3+ CTCAE toxicity (power 80%, 1-sided 5% α). We can also exclude >40% G2+ toxicity (with expected 20%) with >90% power, or >35% G2+ with >80% power (both 5% 1-sided α). To provide current toxicity data and allow potential transition to a phase III trial powered on oncological outcomes, stage II will be randomised with patients allocated in a 1:1:2 ratio (unless otherwise advised by the IDMC). Patients from stage I will be included in stage II.

Power calculations originally incorporated an allowance for 5% of patients non-evaluable for late toxicity by 18 months giving a target sample size of 120 patients for each fractionation cohort i.e. a total target sample size of 240 (an additional 169 patients recruited for stage II, 84 for each fractionation cohort). In September 2018 non-evaluability rates were reviewed and with the Independent Data Monitoring Committee's endorsement the target sample size (i.e. the estimate of the number of patients needed to obtain 57 evaluable DART patients) was inflated.

Using a non-evaluability rate of 22% in the 20f cohort gives a revised target sample size of 37 WBRT (control), 37 SART and 73 DART participants under the 1:1:2 allocation ratio (total of 147 patients in the 20f cohort).

Using a non-evaluability rate of 16% in the 32f cohort gives a revised target sample size of 34 WBRT (control), 34 SART and 68 DART participants under the 1:1:2 allocation ratio (total of 136 in in the 32f cohort).

The non-evaluability rate will be monitored and, with IDMC endorsement, cohort recruitment will continue until there are 57 evaluable DART patients per cohort.

Given that the primary interest is in outcomes associated with DART, the continuation of all three arms of the study will continue to be reviewed by the TMG and IDMC during stage II of the study. If it is felt that sufficient information has accrued about the feasibility of randomisation and about outcomes in the WBRT and SART arms, consideration may be given to dropping these arms if this would expedite meeting the aims of the trial or transition to subsequent phase III evaluation.

13.2. Treatment allocation

Participants will be randomised between standard radiotherapy delivery (WBRT control), SART and DART on a 1:1:2 basis separately within each fractionation cohort.

Treatment allocation is by minimisation with a random element; balancing factors will be centre, neo-adjuvant chemotherapy use and concomitant radiosensitising therapy use.

13.3. Endpoint definitions

13.3.1. Primary endpoints

Stage I

Proportion of randomised patients meeting radiotherapy dose constraints (in the medium plan only) to bladder, bowel and rectum in DART groups (as randomly allocated). RAIDER dose constraints will be specified in the radiotherapy delivery and planning guidelines and data collected on a plan assessment form. A patient in the 32 fraction cohort will be defined as meeting the dose constraints if all of the following are met for the medium plan: rectum 50Gy, 60Gy, 65Gy and 70Gy absolute constraints; bladder outside PTV2 60Gy and 65Gy absolute constraints and small bowel V55, V60, V65, V70 and V74 mandatory constraints. A patient in the 20 fraction cohort will be defined as meeting the dose constraints if all of the following are met for the medium plan: rectum 41.7Gy, 50Gy, 54.2Gy and 58.3Gy absolute constraints; bladder outside PTV2 50Gy and 54.2Gy absolute constraints; and small bowel V45.8, V50, V54.2, V58.3 and V61.7 mandatory constraints.

Stage II

Proportion of evaluable patients experiencing any ≥G3 Common Terminology Criteria for Adverse Events (CTCAE) v.4 late toxicity (occurring 6-18 months post radiotherapy).

13.3.2. Secondary endpoints

Stage I:

- Recruitment rate – this will be assessed overall, by country and by radiotherapy centre. Specific recruitment targets in terms of number of open centres and number of patients recruited will be defined in collaboration with the Trial Steering Committee at the beginning of the trial.
- Ability to deliver SART and DART – this will be measured by the number of patients that received their allocated treatment (technique and dose) overall, by country and by radiotherapy centre. The number of fractions using adaptive radiotherapy will be reported.

Stage II

- Clinician reported acute toxicity – this will be assessed weekly during treatment, at 6 weeks (20f cohort only) and 10 weeks from the start of radiotherapy and 3 months from the last fraction using CTCAE v.4. The worst toxicity recorded during this acute period is of primary interest.
- Patient reported outcomes (PRO) - acute and late bladder and bowel/rectal symptoms – these will be assessed using PRO-CTCAE, the King's Health Questionnaire (KHQ), ALERT-B, sexual function questions (excerpt of the EORTC QLQ-BLM30) and the EQ5D-5L. Acute is defined as 3 months from the last fraction and late is from 6 months onwards. The time point of primary interest is 18 months from the last fraction.
- Health economic related measures - time for outlining, plan generation, selection and delivery, healthcare resource usage subsequent to treatment.
- Loco-regional MIBC control – this will be defined as bladder cancer (muscle and non-muscle invasive) or cancer of the pelvic nodes. The proportion of patients free from loco-regional recurrence at 2 years will be reported.
- Progression-free survival – this will define an event as the first occurrence of local or distant disease or death and time will be measured from randomisation. Patients with no event will be censored on date of last assessment of disease.
- Overall survival – this will include deaths from any cause and time will be measured from randomisation. Patients who are alive at the time of analysis will be censored on date of last clinical assessment.

Hafeez S, *et al. BMJ Open* 2020; 10:e041005. doi: 10.1136/bmjopen-2020-041005

13.3.3. Exploratory endpoints

IGRT endpoints:

- Use of adaptive plans – this will be assessed by the number of small or large plans being selected rather than the medium plan for patients receiving adaptive radiotherapy.
- Target coverage – this will be assessed by retrospective outlining of selected post treatment CBCT scans and a descriptive comparison made with the plan used for treatment. A random sample of patients will be re-outlined (with the number of patients chosen based on time constraints and feasibility).
- Online/offline concordance - this will be assessed by an independent reviewer to select an appropriate plan (offline) for a random sample of patients. The concordance between the online and independent reviewer plan selection will be presented.
- Dose volume histogram analysis of adaptive and standard planning – this will be exploratory and used to inform future dose-modelling work. This will include assessment of reduction in normal tissue exposure using SART and DART and correlation of dose volume data with toxicity.

13.4. Statistical analysis plan

Primary endpoint analyses will be conducted separately for the 20f and 32f cohorts. Secondary endpoint analysis populations are defined below as appropriate. Analyses will be conducted at ICR-CTSU.

Stage I

Primary endpoint

Principal analysis will be by intention to treat for stage I. For the primary endpoint, the frequency and percentage of randomised patients able to meet the trial dose constraints in the DART group will be presented. Reasons will be presented for any patient for whom the dose constraints could not be met.

Secondary endpoints

For the secondary endpoint of recruitment, data will be presented as monthly recruitment by centre and country. Actual versus predicted recruitment will be presented graphically. Ability to deliver SART and DART will be presented as the proportion of patients who received their allocated treatment in terms of technique and dose. Data from each fractionation cohort will be presented separately and combined.

Stage II

Primary endpoints

Principal analysis of the primary endpoint will be based on the evaluable population, i.e. DART patients receiving at least one fraction of allocated treatment and having at least one toxicity assessment performed between 6 and 18 months after completion of radiotherapy. Toxicity assessments will be censored one month prior to death, bladder cancer recurrence or progression. To ensure sufficient follow-up time to observe any severe adverse reactions analyses will be conducted after all patients have been on the study for at least 18 months following the completion of radiotherapy. The proportion of patients with any G3+ CTCAE toxicity occurring within 6 to 18 months post radiotherapy will be presented for each randomised treatment group together with the 90% one-sided binomial confidence interval (the 90% two-sided confidence interval will also be presented). A sensitivity analysis will be conducted using a per protocol population. The per protocol population will include evaluable patients who received their complete fractionation schedule (either 32f or 20f) according to their randomised allocation (WBRT (control), SART or DART).

Secondary endpoints

Clinician assessed acute and late toxicity will be summarised as frequency and percentage of each grade of toxicity (CTACE and RTOG (late toxicity only)) at each time point. The distribution of acute and late toxicity will also be presented graphically as stacked barcharts. Kaplan-Meier methods may be used to use present

time-to-event data e.g. time to first occurrence of a grade 2 or greater event. Analyses will be conducted separately for each fraction cohort and combined.

Planned subgroup analyses will present toxicity data according to country, whether patients received neo-adjuvant and concomitant therapy. As there is limited published safety data on the use of concomitant gemcitabine with a 55Gy/20f fractionation schedule, data from this subgroup will be presented separately. To maximise the amount of data available, these exploratory subgroup analyses will be presented initially for both fractionation cohorts separately but also combining data for the fractionations.

PRO scores will be generated by combining individual items to produce subscale and total scores for each domain for each of the questionnaires using standard algorithms. Descriptive statistics will be used to present data at each time point by treatment group. Analyses to account for the longitudinal nature of the data will be explored.

Health economic related measures: time for outlining, plan generation, selection and delivery, healthcare resource usage subsequent to treatment. Data will be analysed using descriptive statistics with data presented by treatment group, both within fractionation cohorts and overall.

Loco-regional MIBC control rate at 2 years will be presented by treatment group with a 95% confidence interval. The local control rate will be presented as a proportion with patients only included in the denominator if they were able to have an assessment at 2 years. Data from each fractionation cohort will be combined.

Kaplan-Meier methods will be used to analyse progression-free and overall survival. Data will be presented by treatment group. Data from each fractionation cohort will be combined and the log-rank test (stratified by fractionation cohort) used for an exploratory comparison of the treatment groups. Pre-planned exploratory efficacy analyses will be presented according to standard dose (WBRT and SART groups) versus escalated dose (DART).

Exploratory endpoints

Data on use of the adaptive plans will be presented separately for each adaptive group with the frequency of each small, medium and large plans used, the denominator will be the total number of fractions received within the randomised group. Descriptive statistics will be used to summarise all the exploratory endpoints and data generated will be used to inform future dose modelling work.

Further details of analysis methods will be specified in a Statistical Analysis Plan in accordance with ICR-CTSU Standard Operating Procedures.

13.5. Interim analyses and stopping rules

Adherence to randomised treatment will be monitored closely during recruitment by the ICR-CTSU, particularly during stage I to determine feasibility of delivering DART. During stage I, if the medium size plan for any DART patient does not meet dose constraints, centres will be required to notify ICR-CTSU to enable central review by an accredited TMG member prior to treatment delivery. If patients are not able to receive DART (in either fractionation cohort) for any reason then a deviation form will be requested providing details of the deviation from allocated treatment. By design, the trial could be stopped at stage I, following review by the IDMC, if the reason for treatment deviation is failure to meet dose constraints in 6 or more DART patients in each fractionation cohort.

During stage II, if it is felt that sufficient information has accrued about the feasibility of randomisation and about outcomes in the WBRT and SART arms, consideration may be given to dropping these arms, following review by the IDMC, if this would expedite meeting the aims of the trial or transition to subsequent phase III evaluation.

Acute and late toxicity will be monitored at regular intervals by the IDMC. If there are more than 6 ≥G3 emergent radiotherapy related late toxicity events reported in either DART fractionation cohort the event

rate will exceed the threshold specified in the trial design and, on the IDMC's recommendation, the trial could be stopped or a DART fractionation cohort dropped early.

The safety of giving concomitant gemcitabine with a 55Gy/20f fractionation schedule and concomitant chemotherapy with 20f DART will be monitored by the IDMC as there are few published data for this treatment combination. Toxicity data will be presented separately for IDMC review for the 20f cohort of patients receiving concomitant therapy.

Whilst there are no formal rules for stopping the trial early due to acute toxicity, if, after 6 patients have been treated per cohort, >50% of patients experience acute grade 3 treatment related toxicity, the IDMC would be asked to advise on continuation. The frequency of subsequent acute toxicity review will be determined by the IDMC. Although the study will be underpowered to show non-inferiority of SART in terms of local control, recurrence rates will be monitored closely. A stopping rule will be formalised following discussion with the IDMC. This is likely to be based on the premise that an absolute excess of x loco-regional recurrence or more (where x will be pre-specified in collaboration with the IDMC) would be reason to consider early termination of the trial at the halfway stage of recruitment.

14. TRIAL MANAGEMENT

14.1. Trial management group (TMG)

A Trial Management Group (TMG) will be set up and will include the Chief Investigator, ICR-CTSU Scientific Lead, TROG Trial Chairperson, Co-investigators and identified collaborators, the ICR-CTSU Trial Statistician and Trial Manager. Principal Investigators and key study personnel will be invited to join the TMG as appropriate to ensure representation from a range of sites and professional groups. Membership will include a lay/consumer representative. The TMG will meet at regular intervals, and at least annually. Notwithstanding the legal obligations of the sponsor and Chief Investigator, the TMG have operational responsibility for the conduct of the trial. The Committee's terms of reference, roles and responsibilities will be defined in a charter issued by ICR-CTSU.

14.2. Trial steering committee (TSC)

The RAIDER trial will be overseen by the ICR-CTSU Urology Radiotherapy Trials Steering Committee (TSC) which includes an independent Chairman (not involved directly in the trial other than as a member of the TSC) and not less than two other independent members. The TSC will meet annually. The TSC will provide expert independent oversight of the trial on behalf of the sponsor and funder. The Committee's terms of reference, roles and responsibilities are defined in charter issued by ICR-CTSU.

14.3. Independent data monitoring committee (IDMC)

An Independent Data Monitoring Committee (IDMC) will be set up to monitor the progress of the trial and will comprise a Chairman and at least two further members with clinical or statistical expertise (at least one member must be a statistician). Membership of the IDMC will be proposed by the TMG and approved by the TSC.

The IDMC will meet in confidence at regular intervals, and at least annually. A summary of findings and any recommendations will be produced following each meeting. This summary will be submitted to the TMG and TSC, and if required, the main REC.

The IDMC will reserve the right to release any data on outcomes or side-effects through the TSC to the TMG (and if appropriate to participants) if it determines at any stage that the combined evidence from this and other studies justifies it.

The Committee's terms of reference, roles and responsibilities will be defined in a charter issued by ICR-CTSU.

15. RESEARCH GOVERNANCE

15.1. Sponsor responsibilities

The Sponsor of this clinical trial is the Institute of Cancer Research (ICR).

A coordinating group in each participating (non-UK) country will be delegated responsibility for trial initiation and conduct in that country on behalf of the Sponsor, as defined in an agreement between the Sponsor and the coordinating group.

15.2. Participating site responsibilities

Responsibilities of participating sites are defined in an agreement between the individual participating site and the Sponsor (UK) or the coordinating group delegated that responsibility by the Sponsor (non-UK).

16. TRIAL ADMINISTRATION AND LOGISTICS

16.1. Site activation

Before activating the trial, participating sites are required to sign an agreement accepting responsibility for all trial activity which takes place within their site.

Sites may commence recruitment once the site agreement has been signed by all required signatories, the required trial documentation is in place (as specified by ICR-CTSU) and a site initiation (visit or teleconference) has taken place. Site initiation visits will be conducted at sites where the Principal Investigator has requested one or where ICR-CTSU deems it is appropriate.

16.2. Investigator training

Each centre will complete the comprehensive pre-trial section of the quality assurance programme prior to commencing recruitment, as detailed in section 11. In addition to this, prior to trial initiation, a practical workshop will be held to educate Principal Investigators, radiographers and physicists in adaptive radiotherapy techniques. The radiotherapy quality assurance programme will continue throughout the trial, with investigator training as required.

Training materials relating to fiducial marker placement will be provided and planning CT images for the first participant at each centre with fiducial markers will be centrally reviewed to ensure consistency with placement guidelines.

16.3. Data acquisition

Electronic (e) Case Report Forms (CRF) will be used for the collection of all trial data. Data from all collaborating groups will be held centrally by the ICR-CTSU.

ICR-CTSU will provide guidance to sites to aid the completion of the eCRFs. The Trial Management Group reserves the right to amend or add to the eCRF template as appropriate. Such changes do not constitute a protocol amendment, and revised or additional forms should be used by sites in accordance with the guidelines provided by ICR-CTSU.

16.4. Central data monitoring

Once data has been entered on the eCRF by the site personnel, ICR-CTSU will review it for compliance with the protocol, and for inconsistent or missing data. Should any missing data or data anomalies be found, queries will be raised for resolution by the site.

Any systematic inconsistencies identified through central data monitoring may trigger an on-site monitoring visit.

16.5. On-site monitoring

If a monitoring visit is required, ICR-CTSU will contact the site to arrange the visit. Once a date has been confirmed, the site should ensure that full patient notes of participants selected for source data verification are available for monitoring.

ICR-CTSU staff conducting on-site monitoring (at UK sites) will review essential documentation and carry out source data verification to confirm compliance with the clinical trial agreement and trial protocol. If any problems are detected during the course of the monitoring visit, ICR-CTSU will work with the Principal Investigator or delegated individual to resolve issues and determine appropriate action.

16.6. Completion of the study and definition of study end date

The study end date is deemed to be the date of last data capture.

16.7. Archiving

Essential trial documents should be retained according to local policy and for a sufficient period for possible inspection by the regulatory authorities (at least 5 years after the date of last data capture). Documents should be securely stored and access restricted to authorised personnel.

17. PATIENT PROTECTION AND ETHICAL CONSIDERATIONS

17.1. Trial approvals

This trial has been formally assessed for risk by ICR-CTSU.

In the UK, ICR-CTSU, on behalf of the Sponsor, will ensure that the trial has received ethics approval from a research ethics committee for multi-centre trials and global R&D approval via the NIHR Coordinated System for gaining NHS Permission. Before entering patients, the Principal Investigator at each site is responsible for submitting Site Specific Information and gaining local Research and Development approval of this protocol.

The coordinating group in each country, on behalf of the Sponsor, will ensure that the trial has received all relevant ethical, regulatory and institutional approval prior to the recruitment of any patients. Further details are provided in Appendix A6.

17.2. Trial conduct

This trial will be conducted according to the approved protocol and its amendments, supplementary guidance and manuals supplied by the Sponsor and in accordance with relevant national guidelines.

17.3. Informed consent

Patients should be asked to sign the current ethics approved main RAIDER consent form at trial entry after receiving both verbal and written information about the trial, having been given sufficient time to consider this information. All consent forms must be countersigned by the Principal Investigator or a designated individual. A signature log of delegated responsibilities, listing the designated individuals and the circumstances under which they may countersign consent forms, must be maintained at the participating site. This log, together with original copies of all signed patient consent forms, should be retained in the Site Investigator File and must be available for inspection. The current ethics approved RAIDER patient information sheets should be provided in addition to any standard patient information sheets that are provided by the site and used in routine practice.

17.4. Patient confidentiality

Patients will be asked to consent to their full name being collected at registration in addition to their date of birth, hospital number, postcode and NHS number or equivalent to allow linkage with routinely collected healthcare data.

Each investigator should keep a separate log of all participants' Trial IDs, names, addresses and hospital numbers. The investigator must retain trial documents (e.g. participants' written consent forms) in strict confidence. The investigator must ensure the participants' confidentiality is maintained at all times.

Representatives of ICR-CTSU and the regulatory authorities will require access to participants' hospital notes for quality assurance purposes. ICR-CTSU will maintain the confidentiality of participants at all times and will not reproduce or disclose any information by which participants could be identified.

17.5. Data protection act (DPA)

ICR-CTSU will comply with all applicable data protection laws.

17.6. Liability

The coordinating group in each country will ensure that appropriate indemnity arrangements are place to meet the potential legal liabilities of investigators conducting the trial .

18. FINANCIAL MATTERS

This trial is investigator designed and led and has been approved by the Clinical Trials Advisory and Awards Committee (CTAAC) of Cancer Research UK and Cancer Australia.

ICR has received funding from Cancer Research UK for the central coordination of the trial. In the UK, the trial meets the criteria for R&D support as outlined in the Statement of Partnership on Non-Commercial R&D in the NHS in England. The trial is part of the National Institute for Health Research Clinical Research Network (NCRN) portfolio. NCRN resources should therefore be made available for the trial to cover UK specific research costs.

The coordinating group in other countries will ensure that sufficient funding is available for the coordination and conduct of the trial.

19. PUBLICATION POLICY

The main trial results will be based on data from all collaborative groups and will be published in a peer-reviewed journal, on behalf of all collaborators. The manuscript will be prepared by a writing group, consisting of members of the TMG and selected participating clinicians. All participating clinicians will be acknowledged in the publication.

Any presentations and publications relating to the trial must be authorised by the TMG. Authorship of any secondary publications e.g. those relating to sub-studies, will reflect the intellectual and time input into these studies.

No investigator may present or attempt to publish data relating to the RAIDER trial without prior permission from the TMG.

20. ASSOCIATED STUDIES

20.1. Patient reported outcome measures study

Patient reported outcomes will be a secondary endpoint in the main trial and will be analysed as described in the statistical analysis plan.

Further details are provided in Appendix A4.

20.2. RAIDER translational sample collection

Prospective consent will be sought for access to formalin fixed paraffin embedded (FFPE) tissue blocks routinely obtained at first diagnosis and those from any subsequent first recurrence. FFPE blocks will be requested retrospectively from sites and will be sent to the University of Manchester for storage. Samples

will be held under the custodianship of the Trial Management Group on behalf of the sponsor. Translational analyses will be conducted at a later date once appropriate funding has been secured.

Hafeez S, et al. BMJ Open 2020; 10:e041005. doi: 10.1136/bmjopen-2020-041005

A1. WHO PERFORMANCE STATUS

Grade	Performance Status
0	Able to carry out all normal activity without restriction.
1	Restricted in physically strenuous activity but ambulatory and able to carry out light work.
2	Ambulatory and capable of all self-care but unable to carry out any work; up and about more than 50% of waking hours.
3	Capable of only limited self-care; confined to bed or chair more than 50% of waking hours.
4	Completely disabled; cannot carry on any self-care; totally confined to bed or chair.

A2. RTOG/EORTC LATE RADIATION MORBIDITY SCORING SCHEMA

0	1	2	3	4	5
BLADDER					
None	Slight epithelial atrophy<br>Minor telangiectasia (microscopic haematuria)	Moderate frequency<br>Generalized telangiectasia<br>Intermittent macroscopic haematuria	Severe frequency and dysuria<br>Severe generalized telangiectasia (often with petechiae)<br>Frequent haematuria<br>Reduction in bladder capacity (<150 cc)	Necrosis/<br>Contracted bladder (capacity <100 cc)<br>Severe haemorrhagic cystitis	Death due to toxicity
SMALL/LARGE INTESTINE					
None	Mild diarrhoea<br>Mild cramping<br>Bowel movement 5 times daily<br>Slight rectal discharge or bleeding	Moderate diarrhoea and colic<br>Bowel movement >5 times daily<br>Excessive rectal mucus or intermittent bleeding	Obstruction or bleeding requiring surgery	Necrosis/<br>Perforation<br>Fistula	Death due to toxicity

A3. TUMOUR LOCALISATION GUIDELINES

For radiotherapy planning the delineated bladder tumour will be defined using all pre-treatment diagnostic imaging, surgical bladder map and the placement of fiducial markers if possible (see Radiotherapy Planning and Delivery Guidelines).

A3.1 Fiducial marker placement

Prior to radiotherapy, where possible fiducial marker insertion (gold seed or Lipiodol) into the bladder wall surrounding the tumour should be considered. Only patients medically fit to undergo a general anaesthetic should be considered for gold seed insertion. Only patients fit for general anaesthetic and without a history of contrast medium sensitivity or active thyroid disease should be considered for Lipiodol insertion.
The fiducial markers are inserted into the bladder wall to demarcate the maximum extent of visible tumour or tumour bed. Gold seeds need to be inserted via a customised introducer.
The recommended procedure for Lipiodol injection is

1. Undertake cystoscopy under general anaesthetic performing a cystourethroscopy with visual mapping of scars and lesions. Record details on trial proforma. Measure bladder volume. If required biopsy scar plus/minus random biopsy.
2. Lipiodol is inserted using a 5 French ‘Botox’ needle.
3. Draw up 5 mls of Lipiodol
4. With the bladder full, inject 0.5 mls subepithelially 2cms away from scar or residual tumour. Use 4-6 injections circumferentially around scar. Do not exceed injection volume as this can lead to pelvic leakage.
5. Diathermy injection sites to prevent Lipiodol leaking back out
6. Record details of procedures on bladder map, make note of number of injections, position and distance from scar.

Fiducial marker placement is unlikely to result in side effects over and above the toxicities associated with cystoscopy +/- general anaesthetic.

A3.2 Surgical bladder map

At the time of cystoscopy the urologist will be ask to localise the tumour (size and position) on a surgical bladder map to aid tumour localisation for radiotherapy planning.

A3.3 Training and quality assurance

A video demonstrating the fiducial marker placement technique will be available on the ICR-CTSU website. Each centre will be requested to nominate a lead surgeon providing oversight of fiducial marker placement for RAIDER trial participants (if using). Lead surgeons will be asked to provide details of their centres’ fiducial marker placement experience and to provide assurance that those placing fiducial markers have completed the required training.

Planning CT images for the first participant with fiducial marker placement at each centre will be centrally reviewed.

A4. PATIENT REPORTED OUTCOMES STUDY

A4.1 Background

Patient reported outcomes (PRO) are a key secondary endpoint within RAIDER. PRO within RAIDER will focus on the impact of bladder radiotherapy on symptoms experienced by patients. The aim will be to collect detailed information about the impact of bladder radiotherapy on participants' daily lives, with a focus on side effects being experienced but also including a measure of general wellbeing.

The objective of the PRO sub-study within RAIDER is to compare the impact of adaptive planned radiotherapy on side effects as reported by the participants. This will help to support any differences in toxicity established within the primary endpoint of clinician reported toxicity. In addition, PRO data will be compared with clinician reported toxicity to give an indication of the concordance of the two measures.

A4.2 Hypotheses

1. SART minimises treatment toxicity and improves patient reported symptoms /quality of life
2. DART is tolerated well and has no or minimal impact on patients' reported experiences

A4.3 Quality of life measures

Patient reported outcomes will be measured using the PRO-CTCAE™ questionnaire, King's Health Questionnaire (KHQ), sexual function questions, ALERT-B and the EQ-5D.

PRO-CTCAE is a patient-reported outcome measure developed to evaluate the frequency, severity and interference of symptomatic toxicity in patients on cancer clinical trials. It was designed to be used as a companion to the Common Terminology Criteria for Adverse Events (CTCAE). PRO-CTCAE includes an item library representing symptomatic toxicities drawn from the CTCAE. Items selected for inclusion relate to gastrointestinal symptoms (41).

Urinary side-effects experienced by participants will be captured using the KHQ, which has been validated for use in patients with overactive bladder(42) and captures details of the severity of symptoms and the impact of urinary incontinence on day to day living. Impact on sexual function will be assessed using an excerpt of the EORTC QLQ-BLM30, a muscle invasive bladder cancer specific questionnaire (43).

Participants will also be asked to complete the EQ5D questionnaire, a brief standardised instrument which provides a simple descriptive profile of health status (44) and the three-item ALERT-B Questionnaire which provides a validated screening tool to detect chronic gastrointestinal symptoms after pelvic radiotherapy in cancer survivors (45).

A4.4 Study design

Patients are eligible for the PRO study if they fulfil the RAIDER eligibility criteria. Participants will be asked in the patient information sheet to consent to regular completion of PRO questionnaires. Patients who decline to take part in the RAIDER PRO study will remain eligible for the main trial. PRO is a secondary endpoint in the main trial and the primary timepoint of interest is 18 months after completion of radiotherapy.

A4.5 Timing of data collection

Participants will be asked to complete a questionnaire in clinic within 2 weeks prior to the start of radiotherapy. Further questionnaires will be completed in clinic at the end of treatment delivery and 3 months from the end of treatment. Four further booklets will be sent to participants' homes by ICR-CTSU at 6, 12, 18 and 24 months from the end of treatment.

A4.6 Compliance

Missing data may hamper interpretation of PRO. Missing data may arise because participants do not complete the questionnaires at the appropriate time (unit non-response), or because patients may miss questions within the questionnaires (item non-response). In a population of patients with low performance status such as those included in RAIDER, there is potential for non-response and informative censoring (with

data not missing at random). During the study, compliance with PRO questionnaire completion will be monitored by the trial oversight committees.

A4.7 Statistical considerations

Patient reported outcome analyses will be used to supplement results of clinician assessed treatment toxicity, therefore a formal sample size calculation has not been performed. An analysis plan will be developed in consultation with the TMG with key endpoints identified from each questionnaire. Standard algorithms will be used to derive scores and handle missing data in quality of life questionnaires. Quality of life data will be presented at individual time-points and analyses to account for the longitudinal nature of the data may be used.

A5. EXPECTED SERIOUS ADVERSE EVENTS

Hospitalisation for any of the following adverse events is exempt from expedited reporting if the event is grade 2 or less:

- Transfusion secondary to bleeding from bladder tumour or anaemia
- Haematuria
- Dysuria/frequency
- Nausea/vomiting
- Bladder spasms or pain
- Diarrhoea
- Constipation
- Abdominal pain
- Urinary tract infection
- Urinary/clot retention
- Fatigue
- Neutropaenia (related to concomitant chemotherapy)
- Thrombocytopaenia (related to concomitant chemotherapy)
- Neutropaenic sepsis (related to concomitant chemotherapy)

A6. TRANS TASMAN RADIATION ONCOLOGY GROUP SPECIFIC ADDENDUM

TROG 14.02

Final GSA Version 3 Date:	23/01/2019

Collaborating Group: Trans Tasman Radiation Oncology Group (TROG)
ABN: 45 132 672 292
Address: PO Box 88, Waratah, NSW, Australia, 2298
Phone: +61 2 401 43911
Email: trog@trog.com.au
Website: www.trog.com.au
TROG Representative: Susan Goode

GSA Authorisation:

_______________________ _______________
sign *date*

TROG Trial Chair: Associate Professor Farshad Foroudi
Address: Olivia Newton-John Cancer & Wellness Centre, Austin Health
145 Studley Road
PO Box 5555 Heidelberg, VIC, Australia, 3084
Email: farshad.foroudi@austin.org.au
Phone: +61 3 9496 9797

GSA Authorisation:

_______________________ _______________
sign *date*

FORWARD

The Trans Tasman Radiation Oncology Group (TROG) has been authorised by the Institute of Cancer Research (ICR) to undertake a coordinating role for participants enrolled in Australia and New Zealand on this trial.

The involvement of TROG necessitates a number of changes to the procedures documented in the main body of the RAIDER protocol. The following sections have been adjusted for TROG trial sites and participants and replace, or add to, the above RAIDER protocol sections where relevant.

A6.1 Group specific committees and contacts

A6.1.1. TROG Trial Coordinating Centre

Additional to protocol page 1

The TROG Trial Coordinating Centre (TCC) will be the liaison between the ICR and the ANZ trial sites.

**TROG Trial Coordinator**	Patrick Wheeler
**Address**	TROG Trial Coordinating Centre<br>PO Box 88, Waratah, NSW<br>2298, Australia
**Email**	RAIDER@trog.com.au
**Phone**	+61 2 4014 3903

A6.2 Randomisation

Replaces section 7 of the protocol

Patients will be randomised by the ICR Clinical Trials and Statistics Unit (ICR-CTSU) via the TROG Central Operations Office (TCOO).

Randomisation Case Report Forms (paper forms) confirming that the patient is eligible and has provided written consent for the trial must be forwarded to the TCOO on:

0061 2 4014 3902 or RAIDER@trog.com.au

Randomisation requests will be processed through the ICR-CTSU system during the next business day after receipt.

Randomisation should take place within 10 weeks prior to the planned start date of radiotherapy. Treatment allocation will be by minimisation (with a random component). An eligibility and randomisation checklist must be completed prior to randomisation.

The following information will be required at randomisation:

- Name of treating and recruiting hospital, consultant and person randomising patient
- Confirmation that patient has given written informed consent for trial and for any sub-studies;
- Confirmation that patient is eligible for the trial by completion of the eligibility checklist

ICR-CTSU will send written confirmation of trial entry, confirming the patient's unique randomisation number (Trial ID) and treatment allocation to the TCOO who shall notify the data management contact at the recruiting centre.

A6.3 Safety reporting

A6.3.1. Reporting serious adverse events to TROG

Replaces section 12.3 of the protocol

Any Serious Adverse Event (SAE) (except those listed in Appendix A5 of the ICR protocol) that occurs from the start of radiotherapy and up to 30 days following the last day of radiotherapy must be reported.

TROG trial sites in Australia and New Zealand shall report SAEs within 24 hours of the Principal Investigator (or designated representative) becoming aware of the event, by completing the RAIDER SAE form and faxing to:

The TROG Central Operations Office

Fax no: 0061 2 4014 3902

As much information as possible, including the Principal Investigator's assessment of causality, must be reported to the TROG TCC in the first instance. Additional follow up information should be reported as soon as it is available.

All SAE forms must be completed, signed and dated by the Principal Investigator or designated representative.

The Site SAE log should be completed and the SAE form filed in the Site Investigator File.

A6.4 Review of serious adverse events

Replaces section 12.5 of the protocol

The TROG Trial Chairperson (or designated representative) will assess all reported SAEs for Australian and New Zealand (ANZ) sites for causality and expectedness (NB. The TROG Trial Chairperson cannot down-grade the Principal Investigator's assessment of causality.)

SAEs assessed as having a causal relationship to study treatment and as being unexpected will undergo expedited reporting to the relevant regulatory authorities and all other interested parties by the TROG TCC (see 3.3).

Sites should respond as soon as possible to requests from the TROG Trial Chairperson or designated representative (via TROG) for further information that may be required for final assessment of an SAE. ICR-CTSU will be provided with details of every reported SAE once final assessment is completed.

A6.5 Expedited reporting of related unexpected SAEs

Replaces section 12.6 of the protocol

If an SAE is identified as being related and unexpected by the TROG Trial Chairperson it will be reported by the TROG TCC to the lead Human Research Ethics Committee (HREC), the Sponsor (via ICR-CTSU) and all other interested parties within each parties' reporting timelines.

The Principal Investigators at all actively recruiting sites will be informed of any related unexpected SAEs occurring within the trial at appropriate intervals.

A6.6 Follow up of serious adverse events

Replaces section 12.7 of the protocol

SAEs should be followed up until clinical recovery is complete or until the condition has stabilised. SAE outcomes should be reported to the TROG TCC using the relevant section of the SAE form as soon as the Principal Investigator or designee becomes aware of the outcome.

RAIDER Protocol
ICR-CTSU

A6.7 Flow diagram for SAE reporting, and action following report

Replaces section 12.10 of the protocol

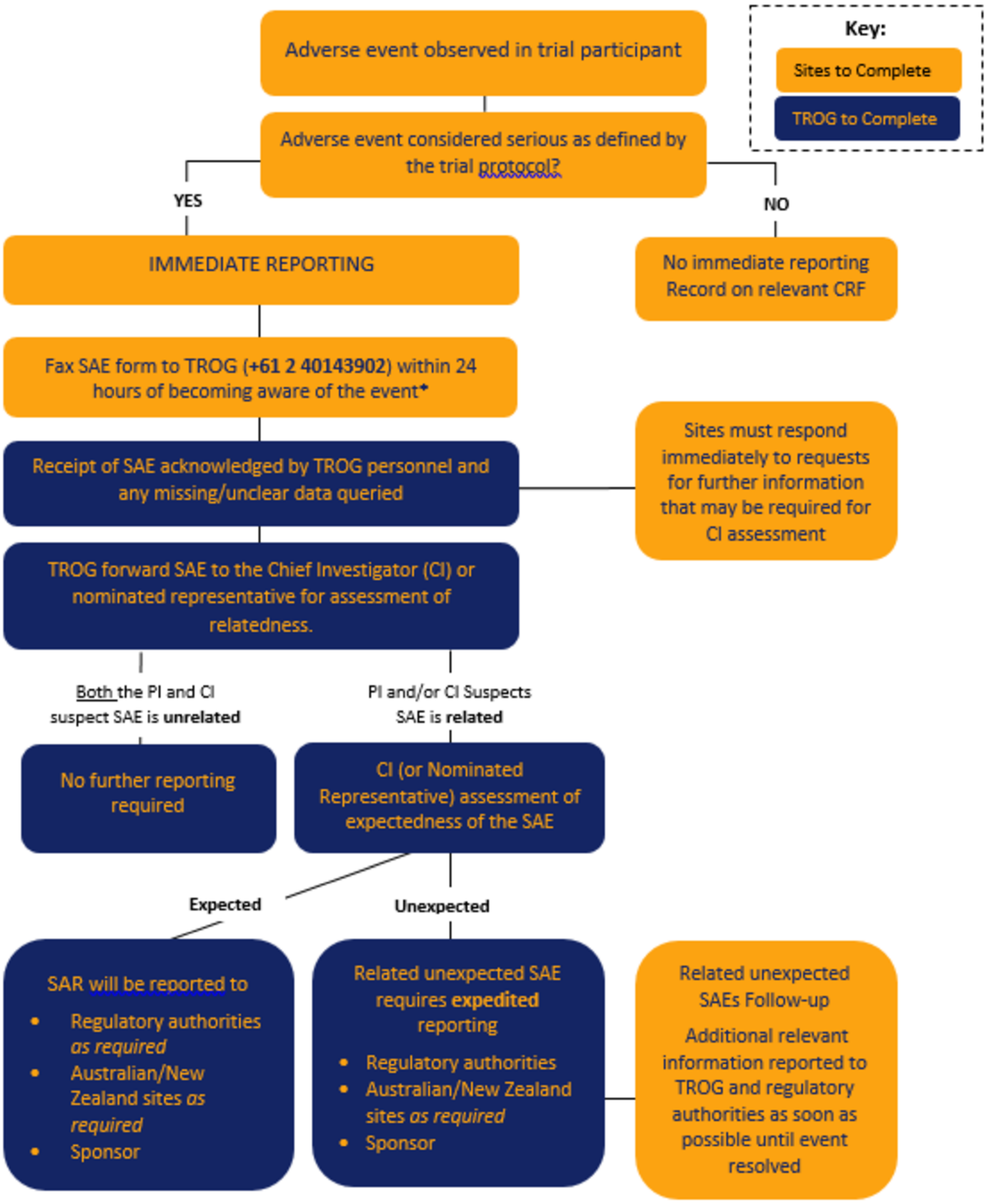

***Site investigator to also report SAE to approving HREC and/or RGO as required**

A6.8 Trial administration and logistics

Replaces section 16 of the protocol

A6.8.1. Site activation

Before activating the trial, participating sites are required to sign an agreement, issued by TROG, accepting responsibility for all trial activity which takes place within their site.

Hafeez S, *et al. BMJ Open* 2020; 10:e041005. doi: 10.1136/bmjopen-2020-041005

Sites may commence recruitment once the site agreement has been signed by all required signatories, the required trial documentation is in place (as specified by TROG) and a site initiation (visit or teleconference) has taken place.

A6.8.2. Investigator training

Each centre will complete radiotherapy quality assurance procedures, as described in the TROG 14.02 Radiotherapy Planning and Delivery Guidelines, available on request from the TROG trial coordinator, prior to commencing recruitment. The quality assurance programme will continue throughout the trial, with investigator training as required.

Training materials relating to fiducial marker placement will be provided and planning CT images for the first participant at each centre with fiducial markers will be centrally reviewed to ensure consistency with placement guidelines.

A6.8.3. Data acquisition

Electronic (e) Case Report Forms (CRF) will be used for the collection of all trial data. Data from all collaborating groups will be held centrally by the ICR-CTSU.

The TROG Central Operations Office will provide guidance to ANZ sites to aid the completion of the eCRFs. The Trial Management Group reserves the right to amend or add to the eCRF template as appropriate. Such changes do not constitute a protocol amendment.

A6.8.4. Central data monitoring

Once data has been entered on the eCRF by the site personnel, ICR-CTSU will review it for compliance with the protocol, and for inconsistent or missing data. Should any missing data or data anomalies be found, queries will be raised for resolution by the site.

Any systematic inconsistencies identified through central data monitoring may trigger an on-site monitoring visit.

A6.8.5. On-site monitoring

If a monitoring visit is required, TROG will contact the site to arrange the visit. Once a date has been confirmed, the site should ensure that full patient notes of participants selected for source data verification are available for monitoring.

TROG staff conducting on-site monitoring will review essential documentation and carry out source data verification to confirm compliance with the clinical trial agreement and trial protocol. If any problems are detected during the course of the monitoring visit, TROG will work with the Principal Investigator or delegated individual to resolve issues and determine appropriate action.

A6.8.6. Completion of the study and definition of study end date

The study end date is deemed to be the date of last data capture.

A6.8.7. Archiving

Essential trial documents and source documentation (including medical histories, radiological imaging, laboratory tests, chemotherapy and radiotherapy treatment records, verification films and portal images) , must be retained for 15 years after completion of the trial in accordance with ICH GCP Guidelines. Documents should be securely stored and access restricted to authorised personnel.

A6.9 Patient reported outcomes study

Replaces section A4.5 of the protocol

Participants will be asked to complete a questionnaire in clinic within 2 weeks prior to randomisation. Further questionnaires will be completed in clinic at the end of treatment delivery and at 3, 6, 12, 18 and 24 months from the end of treatment.

Following progression patients should continue to be asked to complete booklets in accordance with the follow up schedule if they are willing to do so.

A6.10 Financial matters

Replaces section 18 of the protocol

Funding is being sought from competitive grants in Australia and New Zealand. A Cancer Australia grant has been awarded for Australian Sites. A Cancer Society of New Zealand grant has been awarded for New Zealand sites.

A6.11 Research governance

Additional to section 15 of the protocol

A6.11.1. Trial chairperson(s)

TROG is the sponsor's legal representative for this trial in Australia and New Zealand (ANZ). The TROG Trial Chairperson(s) shall be responsible for the conduct of the trial in Australia and New Zealand as set out in the Agreement between TROG and the ICR.

A6.11.2. Trial management committee

The ANZ Trial Management Committee (TMC) will be responsible for monitoring of the progress of the trial in TROG trial sites, decision making, education and information services and reporting as described in TROG Policy Statement TPS E8 Trial Management Committee Responsibilities. The TMC will feedback to the RAIDER Trial Management Group via the TROG Trial Chairperson (who will be a member of the RAIDER Trial Management Group) and other TROG representatives as appropriate.

A6.11.3. Principal Investigator

In each participating centre a Principal Investigator (Radiation Oncologist) will be identified, and will be responsible for identification, recruitment, data collection and completion of CRFs along with follow up of study patients and adherence to the study protocol. Each Principal Investigator will be a member of TROG and adhere to TROG Policy Statements. One investigator per country will be nominated as national coordinator and one investigator per ethics jurisdiction within Australia and within New Zealand will be nominated as Lead Ethics Coordinator. Further details regarding the responsibilities and delegations are set out in the Clinical Trial Agreement between TROG and the participating centre.

A6.12 Patient protection and ethical considerations

Additional to section 17 of the protocol

A6.12.1. Aboriginal and Torres Strait Islander values and principles

TROG recognises and commits to the respect of Aboriginal and Torres Strait Islander cultural values and principles.

Although this trial is not targeted specifically to Aboriginal and Torres Strait Islander peoples, a person from one of these communities may be invited to participate if they meet the eligibility criteria of this trial. This decision will be at the discretion of the Principal Investigator at the Trial Site who shall consent and treat the patient according to the principles set forth in the Guidelines for Ethical Conduct in Aboriginal and Torres Strait Islander Health Research and any specific requirements of the approving Human Research Ethics Committee.

A6.12.2. Insurance and compensation

TROG endorses the principles of the Medicines Australia Guidelines for Compensation for Injury Resulting from Participation in a Company Sponsored Trial and the Research Medicines Industry equivalent in New Zealand.

BMJ Publishing Group Limited (BMJ) disclaims all liability and responsibility arising from any reliance placed on this supplemental material which has been supplied by the author(s)

To provide protection for trial participants involved in TROG Clinical Trials, TROG maintains a clinical trials insurance policy.

Hafeez S, *et al. BMJ Open* 2020; 10:e041005. doi: 10.1136/bmjopen-2020-041005

A7. GLOSSARY

AE	Adverse Event
APPLY	Adaptive predictive planning for hypofractionated bladder radiotherapy
CBCT	Cone Beam CT
CI	Chief Investigator
CI	Confidence Interval
CIS	Carcinoma In Situ
CRF	Case Report Form
CT	Computed Tomography
CTCAE	Common Terminology Criteria for Adverse Events
CTV	Clinical Target Volume
CXR	Chest X-Ray
DART	Dose escalated Adaptive tumour focused Radiotherapy
DCF	Data Capture Form
DVH	Dose Volume Histogram
dwMRI	Diffusion weighted Magnetic resonance Imaging
EORTC	European Organisation for Research and Treatment of Cancer
f	Fraction
FBC	Full Blood Count
GI	Gastrointestinal
GSA	Group Specific Addendum
GTV	Gross Tumour Volume
GU	Genitourinary
Gy	Gray
HR	Hazard Ratio
ICR	The Institute of Cancer Research
ICR-CTSU	The Institute of Cancer Research Clinical Trials and Statistics Unit
IDMC	Independent Data Monitoring Committee
IGRT	Image Guided Radiotherapy
IMRT	Intensity Modulated Radiotherapy
KHQ	King's Health Questionnaire
MDT	Multi-Disciplinary Team
MIBC	Muscle Invasive Bladder Cancer
MRI	Magnetic Resonance Imaging
NCRI	National Cancer Research Institute
NCRI RTTQA	NCRI Radiotherapy Clinical Trials Quality Assurance group
NICE	National Institute for Health and Clinical Excellence
NSAID	Non-Steroidal Anti-Inflammatory Drug
PI	Principal Investigator
PIS	Patient Information Sheet
PRO	Patient Reported Outcomes
PTV	Planning Target Volume
QA	Quality Assurance
R&D	Research and Development
REC	Research Ethics Committee
RMH	Royal Marsden Hospital
RT	Radiotherapy
RTOG	Radiation Therapy Oncology Group
RTTQA	Radiotherapy Trials Quality Assurance
SAE	Serious Adverse Event
SAR	Serious Adverse Reaction
SART	Standard dose Adaptive tumour focused Radiotherapy
TCC	Transitional Cell Carcinoma
TMG	Trial Management Group
TSC	Trial Steering Committee
TURBT	Transurethral resection of Bladder Tumour
U & Es	Urea and Electrolytes
WHO	World Health Organisation

Hafeez S, *et al. BMJ Open* 2020; 10:e041005. doi: 10.1136/bmjopen-2020-041005

A8. REFERENCES

1 Bladder cancer Key Facts. http://publications.cancerresearchuk.org/publicationformat/formatfactsheet/bladderkeyfacts.html: Cancer Research UK; 2014.
2 Cancer in Australia: an overview, 2012. 74 ed. http://www.aihw.gov.au/publication-detail/?id=60129542359: Australian Institute of Health and Welfare & Australasian Association of Cancer Registries 2012.; 2014.
3 Rodel C. Combined-Modality Treatment and Selective Organ Preservation in Invasive Bladder Cancer: Long-Term Results. Journal of Clinical Oncology. 2002;**20**(14):3061-71.
4 NICE. Guidance on Cancer Services Improving Outcomes in Urological Cancers The Manual. online: NICE; 2002.
5 James N, Hall E, Jenkins P, et al. Radiotherapy with or without chemotherapy in muscle invasive bladder cancer. New England Journal of Medicine. 2012;**366**:1477-88.
6 Fokdal L, Honore H, Hoyer M, Meldgaard P, Fode K, von der Maase H. Impact of changes in bladder and rectal filling volume on organ motion and dose distribution of the bladder in radiotherapy for urinary bladder cancer. Int J Radiat Oncol Biol Phys. 2004 Jun 1;**59**(2):436-44.
7 Lotz HT, Pos FJ, Hulshof MC, et al. Tumor motion and deformation during external radiotherapy of bladder cancer. Int J Radiat Oncol Biol Phys. 2006 Apr 1;**64**(5):1551-8.
8 Meijer GJ, Rasch C, Remeijer P, Lebesque JV. Three-dimensional analysis of delineation errors, setup errors, and organ motion during radiotherapy of bladder cancer. Int J Radiat Oncol Biol Phys. 2003 Apr 1;**55**(5):1277-87.
9 Muren LP, Smaaland R, Dahl O. Organ motion, set-up variation and treatment margins in radical radiotherapy of urinary bladder cancer. Radiother Oncol. 2003 Dec;**69**(3):291-304.
10 Sur RK, Clinkard J, Jones WG, et al. Changes in target volume during radiotherapy treatment of invasive bladder carcinoma. Clin Oncol (R Coll Radiol). 1993;**5**(1):30-3.
11 Turner SL, Swindell R, Bowl N, et al. Bladder movement during radiation therapy for bladder cancer: implications for treatment planning. Int J Radiat Oncol Biol Phys. 1997 Sep 1;**39**(2):355-60.
12 Pos F, Remeijer P. Adaptive management of bladder cancer radiotherapy. Semin Radiat Oncol. 2010 Apr;**20**(2):116-20.
13 Lalondrelle S, Huddart R. Improving radiotherapy for bladder cancer: an opportunity to integrate new technologies. Clin Oncol (R Coll Radiol). 2009 Jun;**21**(5):380-4.
14 Lalondrelle S, Huddart R, Warren-Oseni K, et al. Adaptive-predictive organ localization using cone-beam computed tomography for improved accuracy in external beam radiotherapy for bladder cancer. Int J Radiat Oncol Biol Phys. 2011 Mar 1;**79**(3):705-12.
15 Pos FJ, Hulshof M, Lebesque J, et al. Adaptive radiotherapy for invasive bladder cancer: a feasibility study. Int J Radiat Oncol Biol Phys. 2006 Mar 1;**64**(3):862-8.
16 Foroudi F, Wong J, Kron T, et al. Online adaptive radiotherapy for muscle-invasive bladder cancer: results of a pilot study. Int J Radiat Oncol Biol Phys. 2011 Nov 1;**81**(3):765-71.
17 Huddart RA. SJ. What is the most effective way of reducing normal tissue toxicity in radical radiotherapy treatment of muscle invasive bladder cancer? Radiother Oncol 2002;**64(Suppl 1):S305 (1043a)**.
18 Emami B, Lyman J, Brown A, et al. Tolerance of normal tissue to therapeutic irradiation. Int J Radiat Oncol Biol Phys. 1991 May 15;**21**(1):109-22.
19 Marks LB, Carroll PR, Dugan TC, Anscher MS. The response of the urinary bladder, urethra, and ureter to radiation and chemotherapy. Int J Radiat Oncol Biol Phys. 1995 Mar 30;**31**(5):1257-80.
20 Mangar SA, Foo K, Norman A, et al. Evaluating the effect of reducing the high-dose volume on the toxicity of radiotherapy in the treatment of bladder cancer. Clin Oncol (R Coll Radiol). 2006 Aug;**18**(6):466-73.
21 The Radiotherapy Innovation Fund An Evaluation of the Prime Minister's £23 million Fund. http://www.cancerresearchuk.org/prod_consump/groups/cr_common/@nre/@pol/documents/generalcontent/rifevalreport.pdf: Cancer Research UK; 2013 July 2013.
22 National Radiotherapy Implementation Group Report Image Guided Radiotherapy (IGRT) Guidance for implementation and use. http://ncat.nhs.uk/sites/default/files/uploaded/National%20Radiotherapy%20Implementation%20Group%20Report%20IGRTAugust%202012l.pdf: National Cancer Action Team; 2012.
23 Foroudi F, Pham D, Rolfo A, et al. The outcome of a multi-centre feasibility study of online adaptive radiotherapy for muscle-invasive bladder cancer TROG 10.01 BOLART. Radiother Oncol. 2014 May;**111**(2):316-20.
24 Hoskin PJ, Rojas AM, Bentzen SM, Saunders MI. Radiotherapy with concurrent carbogen and nicotinamide in bladder carcinoma. J Clin Oncol. 2010 Nov 20;**28**(33):4912-8.
25 Choudhury A, Swindell R, Logue JP, et al. Phase II study of conformal hypofractionated radiotherapy with concurrent gemcitabine in muscle-invasive bladder cancer. J Clin Oncol. 2011 Feb 20;**29**(6):733-8.

26 Coppin CM, Gospodarowicz MK, James K, et al. Improved local control of invasive bladder cancer by concurrent cisplatin and preoperative or definitive radiation. The National Cancer Institute of Canada Clinical Trials Group. Journal of Clinical Oncology. 1996 Nov;**14**(11):2901-7.
27 Eustace A, Irlam JJ, Taylor J, et al. Necrosis predicts benefit from hypoxia-modifying therapy in patients with high risk bladder cancer enrolled in a phase III randomised trial. Radiotherapy and Oncology. 2013 Jul;**108**(1):40-7.
28 Tuomikoski L, Collan J, Keyrilainen J, Visapaa H, Saarilahti K, Tenhunen M. Adaptive radiotherapy in muscle invasive urinary bladder cancer--an effective method to reduce the irradiated bowel volume. Radiother Oncol. 2011 Apr;**99**(1):61-6.
29 Burridge N, Amer A, Marchant T, et al. Online adaptive radiotherapy of the bladder: small bowel irradiated-volume reduction. Int J Radiat Oncol Biol Phys. 2006 Nov 1;**66**(3):892-7.
30 Vestergaard A, Sondergaard J, Petersen JB, Hoyer M, Muren LP. A comparison of three different adaptive strategies in image-guided radiotherapy of bladder cancer. Acta Oncol. 2010 Oct;**49**(7):1069-76.
31 Murthy V, Master Z, Adurkar P, et al. 'Plan of the day' adaptive radiotherapy for bladder cancer using helical tomotherapy. Radiother Oncol. 2011 Apr;**99**(1):55-60.
32 Huddart R, McDonald F, Lewis R, Hall E. HYBRID - Evaluating New Radiation Technology in Patients with Unmet Needs. Clin Oncol (R Coll Radiol). 2013 Jun 21.
33 McDonald F, Lalondrelle S, Taylor H, et al. Clinical Implementation of Adaptive Hypofractionated Bladder Radiotherapy for Improvement in Normal Tissue Irradiation. Clinical Oncology. 2013 9//;**25**(9):549-56.
34 Huddart RA, Hall E, Hussain SA, et al. Randomized Noninferiority Trial of Reduced High-Dose Volume Versus Standard Volume Radiation Therapy for Muscle-Invasive Bladder Cancer: Results of the BC2001 Trial (CRUK/01/004). International Journal of Radiation Oncology*Biology*Physics. 2013 10/1/;**87**(2):261-9.
35 Cowan R, McBain C, Ryder W, et al. Radiotherapy for muscle-invasive carcinoma of the bladder: results of a randomized trial comparing conventional whole bladder with dose-escalated partial bladder radiotherapy. Int J Radiat Oncol Biol Phys. 2004;**59**(1):197-207.
36 IDEAL - Image Guided Dose Escalated Adaptive Bladder Radiotherapy. [cited 2013 31/07/2013]; Available from: http://public.ukcrn.org.uk/Search/StudyDetail.aspx?StudyID=7653
37 Hafeez S, Koh M, Sohaib A, Huddart R. The role of functional MRI to characterise tumour boost delineation in patients undergoing radical radiotherapy for muscle invasive bladder cancer (MIBC). NCRI Cancer Conference. Liverpool; 2012.
38 Mangar S, Thompson A, Miles E, Huddart R, Horwich A, Khoo V. A feasibility study of using gold seeds as fiducial markers for bladder localization during radical radiotherapy. Br J Radiol. 2007 Apr;**80**(952):279-83.
39 Chai X, van Herk M, van de Kamer JB, et al. Behavior of lipiodol markers during image guided radiotherapy of bladder cancer. Int J Radiat Oncol Biol Phys. 2010 May 1;**77**(1):309-14.
40 Pos F, Bex A, Dees-Ribbers HM, Betgen A, van Herk M, Remeijer P. Lipiodol injection for target volume delineation and image guidance during radiotherapy for bladder cancer. Radiother Oncol. 2009 Nov;**93**(2):364-7.
41 Basch E, Reeve BB, Mitchell SA, et al. Development of the National Cancer Institute's patient-reported outcomes version of the common terminology criteria for adverse events (PRO-CTCAE). J Natl Cancer Inst. 2014 Sep;**106**(9).
42 Reese P, Pleil A, Okano G, Kelleher C. Multinational study of reliability and validity of the King's Health Questionnaire in patients with overactive bladder. Qual Life Res. 2003 2003/06/01;**12**(4):427-42.
43 Fayers P, NKAaronson, Bjordal K, Groenvold M, Curran D, Bottomley A. EORTC QLQ-C30 Scoring Manual. 3rd ed; 2001.
44 Herdman M, Gudex C, Lloyd A, et al. Development and preliminary testing of the new five-level version of EQ-5D (EQ-5D-5L). Qual Life Res. 2011 Dec;**20**(10):1727-36.
45 Taylor S, Byrne A, Adams R, et al. The Three-item ALERT-B Questionnaire Provides a Validated Screening Tool to Detect Chronic Gastrointestinal Symptoms after Pelvic Radiotherapy in Cancer Survivors. Clin Oncol (R Coll Radiol). 2016 Oct;**28**(10):e139-47.
46 Barajas RF, Jr., Rubenstein JL, Chang JS, Hwang J, Cha S. Diffusion-weighted MR imaging derived apparent diffusion coefficient is predictive of clinical outcome in primary central nervous system lymphoma. AJNR Am J Neuroradiol. 2010 Jan;**31**(1):60-6.
47 Pope WB, Kim HJ, Huo J, et al. Recurrent glioblastoma multiforme: ADC histogram analysis predicts response to bevacizumab treatment. Radiology. 2009 Jul;**252**(1):182-9.
48 Kyriazi S, Collins DJ, Messiou C, et al. Metastatic ovarian and primary peritoneal cancer: assessing chemotherapy response with diffusion-weighted MR imaging--value of histogram analysis of apparent diffusion coefficients. Radiology. 2011 Oct;**261**(1):182-92.
49 Cui Y, Zhang XP, Sun YS, Tang L, Shen L. Apparent diffusion coefficient: potential imaging biomarker for prediction and early detection of response to chemotherapy in hepatic metastases. Radiology. 2008 Sep;**248**(3):894-900.

50 Harry VN, Semple SI, Gilbert FJ, Parkin DE. Diffusion-weighted magnetic resonance imaging in the early detection of response to chemoradiation in cervical cancer. Gynecologic oncology. 2008 Nov;**111**(2):213-20.
51 Barbaro B, Vitale R, Valentini V, et al. Diffusion-weighted magnetic resonance imaging in monitoring rectal cancer response to neoadjuvant chemoradiotherapy. Int J Radiat Oncol Biol Phys. 2012 Jun 1;**83**(2):594-9.
52 Pearson RA, Thelwall PE, Snell J, et al. Evaluation of early response to neoadjuvant chemotherapy in muscle-invasive bladder cancer using dynamic contrast-enhanced MRI and diffusion weighted MRI: MARBLE study. Journal of Clinical Oncology. 2016;**34**(2_suppl):403-.
53 Messiou C, Collins DJ, Morgan VA, Desouza NM. Optimising diffusion weighted MRI for imaging metastatic and myeloma bone disease and assessing reproducibility. Eur Radiol. 2011 Aug;**21**(8):1713-8.
54 Koh DM, Blackledge M, Collins DJ, et al. Reproducibility and changes in the apparent diffusion coefficients of solid tumours treated with combretastatin A4 phosphate and bevacizumab in a two-centre phase I clinical trial. Eur Radiol. 2009 Nov;**19**(11):2728-38.
55 Bilgili MY. Reproductibility of apparent diffusion coefficients measurements in diffusion-weighted MRI of the abdomen with different b values. Eur J Radiol. 2011 Jul 1.
56 Braithwaite AC, Dale BM, Boll DT, Merkle EM. Short- and midterm reproducibility of apparent diffusion coefficient measurements at 3.0-T diffusion-weighted imaging of the abdomen. Radiology. 2009 Feb;**250**(2):459-65.
57 Yoshida S, Koga F, Kobayashi S, et al. Role of diffusion-weighted magnetic resonance imaging in predicting sensitivity to chemoradiotherapy in muscle-invasive bladder cancer. Int J Radiat Oncol Biol Phys. 2012 May 1;**83**(1):e21-7.
58 Hafeez S, Koh M, Sohaib A, Huddart R. PD-0464: Assessing response to chemotherapy with diffusion weighted MRI (DW-MRI) in muscle invasive bladder cancer (MIBC). Radiotherapy and Oncology. 2013 3//;**106, Supplement 2**(0):S180-S1.
59 Hafeez S, Koh M, Sohaib A, Huddart R. PO-0734: Assessing response to radiotherapy with diffusion weighted MRI (DW-MRI) in muscle invasive bladder cancer (MIBC). Radiotherapy and Oncology. 2014 //;**111, Supplement 1**(0):S34.
60 Hafeez S KM, Sohaib A, Huddart R. Use of diffusion weighted-MRI (DW-MRI) as a biomarker of clinical outcome in muscle invasive bladder cancer (MIBC). NCRI Conference. 2015:http://abstracts.ncri.org.uk/abstract/use-of-diffusion-weighted-mri-dw-mri-as-a-biomarker-of-clinical-outcome-in-muscle-invasive-bladder-cancer-mibc-2/.
61 Hafeez S KM, McQuaid D, Sohaib A, Huddart R. Characterisation of tumour boost with diffusion weighted MRI (DW-MRI) to inform biological target volume for radical radiotherapy in muscle invasive bladder cancer (MIBC). NCRI Conference. 2015:http://abstracts.ncri.org.uk/abstract/characterisation-of-tumour-boost-with-diffusion-weighted-mri-dw-mri-to-inform-biological-target-volume-for-radical-radiotherapy-in-muscle-invasive-bladder-cancer-mibc-2/.
62 Jenkins P, Anjarwalla S, Gilbert H, Kinder R. Defining the clinical target volume for bladder cancer radiotherapy treatment planning. International Journal of Radiation Oncology Biology Physics. 2009 Dec 1;**75**(5):1379-84.
63 Kim JK, Park SY, Ahn HJ, Kim CS, Cho KS. Bladder cancer: analysis of multi-detector row helical CT enhancement pattern and accuracy in tumor detection and perivesical staging. Radiology. 2004 Jun;**231**(3):725-31.
64 Salo JO, Kivisaari L, Lehtonen T. CT in determining the depth of infiltration of bladder tumors. Urol Radiol. 1985;**7**(2):88-93.
65 El-Assmy A, Abou-El-Ghar ME, Mosbah A, et al. Bladder tumour staging: comparison of diffusion- and T2-weighted MR imaging. Eur Radiol. 2009 Jul;**19**(7):1575-81.
66 Weiss E, Hess CF. The impact of gross tumor volume (GTV) and clinical target volume (CTV) definition on the total accuracy in radiotherapy theoretical aspects and practical experiences. Strahlenther Onkol. 2003 Jan;**179**(1):21-30.
67 Njeh CF. Tumor delineation: The weakest link in the search for accuracy in radiotherapy. Journal of medical physics / Association of Medical Physicists of India. 2008 Oct;**33**(4):136-40.
68 Logue JP, Sharrock CL, Cowan RA, Read G, Marrs J, Mott D. Clinical variability of target volume description in conformal radiotherapy planning. Int J Radiat Oncol Biol Phys. 1998 Jul 1;**41**(4):929-31.
69 Hafeez S, Warren-Oseni K, McNair HA, et al. Prospective Study Delivering Simultaneous Integrated High-dose Tumor Boost (</=70 Gy) With Image Guided Adaptive Radiation Therapy for Radical Treatment of Localized Muscle-Invasive Bladder Cancer. Int J Radiat Oncol Biol Phys. 2016 Apr 01;**94**(5):1022-30.

RAIDER Protocol
ICR-CTSU

This protocol is a controlled document and should not be copied, distributed or reproduced without the written permission of the ICR-CTSU. This document should only be used for the management of patients in the RAIDER trial.
