# Supplementary material for: Protocol for tumour-focused dose-escalated adaptive radiotherapy for the radical treatment of bladder cancer in a multicentre phase II randomised controlled trial (RAIDER): radiotherapy planning and delivery guidance
Source: BMJ Open. 2020 Dec 31;10(12):e041005. doi: 10.1136/bmjopen-2020-041005 (PMC7780718; doi:10.1136/bmjopen-2020-041005)
Supplement: Supplementary data [file bmjopen-2020-041005supp002.pdf]

A **R**andomised phase II trial of **A**daptive **I**mage guided standard or **D**ose **E**scalated tumour boost **R**adiotherapy in the treatment of transitional cell carcinoma of the bladder

RADIOTHERAPY PLANNING AND DELIVERY GUIDELINES

Version:4.3

Dated:13/02/2020

Chief Investigator:	Prof Robert Huddart
Sponsor:	The Institute of Cancer Research
Approval:	Cancer Research UK: Clinical Trials Awards& Advisory Committee (CTAAC)
Funders:	Cancer Research UK
Coordinating Trials Unit:	ICR Clinical Trials and Statistics Unit (ICR-CTSU)
	The Institute of Cancer Research

Main REC Reference Number:	15/LO/0539
ISRCTN:	26779187
ClinicalTrials.gov Identifier	NCT02447549
CRUK Reference Number:	CRUK/14/016

The RAIDER trial has been scientifically approved
by Cancer Research UK's Clinical Trials Awards & Advisory Committee (CTAAC)
The RAIDER trial is part of the National Institute for
Health Research Clinical Research Network Trial Portfolio

This is a controlled document which should be referred to in conjunction with the RAIDER protocol and should not be copied, distributed or reproduced without the written permission of the ICR-CTSU. This document should only be used for the management of patients in the RAIDER trial.

RAIDER radiotherapy planning and delivery guidelines

This document sets out guidelines for treatment of patients within the RAIDER trial and should be referred to in combination with the protocol.

This document should only be used for the purpose of the RAIDER trial.

The Trial Management Group reserves the right to amend or add to the radiotherapy guidelines as appropriate. Such changes do not constitute an amendment, and revised guidelines will be circulated to participating centres as needed. Changes between versions will be noted prior to the introduction. Sites treating RAIDER patients for the first time are advised to contact ICR-CTSU to confirm they have the most recent version.

Any questions relating to the detail of the radiotherapy planning and delivery guidelines should be addressed in the first instance to the QA team (See Appendices A& B).

RAIDER radiotherapy planning and delivery guidelines

CONTACT DETAILS

Scientific Coordination

Chief Investigator
Prof Robert Huddart
The Royal Marsden NHS Foundation Trust,
The Institute of Cancer Research,
London,
United Kingdom

Tel: 0208 661 3457
Email: Robert.Huddart@icr.ac.uk

Clinical Coordinator
Dr Shaista Hafeez
The Royal Marsden NHS Foundation Trust,
The Institute of Cancer Research
London
United Kingdom

Tel: 0208 661 3274
Email: Shaista.Hafeez@icr.ac.uk

TROG lead
Prof Farshad Foroudi
Director Radiation Oncology
Austin Health
Melbourne
Australia

Email: farshad.foroudi@austin.org.au

Trial Management

ICR Clinical Trials & Statistics Unit
Division of Clinical Studies,
The Institute of Cancer Research,
Sir Richard Doll Building, Cotswold Road,
Sutton, Surrey SM2 5NG

RAIDER Trial Manager
Tel: 0208 722 4613
Email: RAIDER-icrctsu@icr.ac.uk

Quality Assurance

Radiotherapy Trials and Quality Assurance Group:
Amanda Webster
Mount Vernon Cancer Centre,
Rickmansworth Road,
United Kingdom

Tel: 02038262320
Email: raiderqa.enh-tr@nhs.net

Hafeez S, *et al. BMJ Open* 2020; 10:e041005. doi: 10.1136/bmjopen-2020-041005

RAIDER radiotherapy planning and delivery guidelines

CONTENTS

1 INTRODUCTION 12
2 PLANNING CT SCAN FOR RADIOTHERAPY 13
2.1 Patient preparation 13
2.1.1 Group 1, 2 and 3: General Preparation 13
2.1.2 Group 1: Empty Bladder 13
2.1.3 Group 2 and 3: Bladder Filling 13
2.2 Patient positioning 13
2.3 Planning CT acquisition, scan limits and slice thickness 13
2.4 Planning CT export, fusion and evaluation of bladder filling 13
3 LOCALISATION OF THE TARGET VOLUME AND ORGANS AT RISK 15
3.1 Volume definition 15
3.2 Gross tumour volume 15
3.3 Clinical target volume 15
3.4 Organs at risk 15
3.5 Planning target volume 16
3.5.1 Group 1: standard whole bladder RT 16
3.5.2 Group 2 and 3: adaptive tumour focused RT (standard dose and dose escalated) 16
Table 1: CTV to PTV Expansion Details 17
Table 2: GTV to PTV2 Expansion details (group 2 and 3 only) 18
3.6 Voluming Checklist 18
3.7 Nomenclature 22
Table 3: RAIDER Trial Target Volume and OAR Nomenclature 22
4 PLANNING 24
4.1 Standard Planning 24
4.2 Adaptive Tumour Focused Planning 24
Table 4: Prescription doses 25
4.3 Target volume dose objectives 25
Table 5: Target dose objectives 25
4.4 Normal tissue dose constraints 26
Table 6a: OAR Dose Constraints for 32-fraction schedule 26
Table 6b: OAR Dose constraints for 20-fraction schedule 27
4.5 Planning recommendations 28
4.6 Pre-Treatment checks 28
5 TREATMENT DELIVERY 29
5.1 Standard whole bladder RT (group 1) 29

5.2 Adaptive Tumour Focused RT (SART and DART) ... 29
5.2.1 Image Match and Plan Selection Steps: ... 29
5.2.2 Image Match and Plan Selection Tips: ... 30
5.3 Post treatment ... 31
5.4 On completion of radiotherapy ... 31
6 TREATMENT SCHEDULING AND GAPS ... 31
APPENDIX A: ... 33
United Kingdom RAIDER QA Programme ... 33
APPENDIX B: ... 36
Trans Tasman Radiation Oncology Group: Australia & NZ ... 36
APPENDIX C: ... 40
PRE-TRIAL OUTLINING BENCHMARK CASE ... 40
Outlining Benchmark Case 1: T2N0M0 Male Pelvis ... 40
Outlining Benchmark Case 2: T2N0M0 Female Pelvis ... 42
PRE-TRIAL PLANNING BENCHMARK CASE ... 43
Benchmark Planning Case: T2N0M0 TCC male pelvis ... 43
APPENDIX D ... 45
APPENDIX E ... 46
APPENDIX F ... 47
APPENDIX G ... 52
APPENDIX H ... 53
APPENDIX I ... 54

RAIDER radiotherapy planning and delivery guidelines

VERSION CHANGES

V1.0 - Original RT planning and delivery guidelines

V2.0 – RT planning and delivery guidelines updated to include TROG QA appendix and contact details.

V3.0- The following changes have been made:

- Section 2.4 (Pg 11) and Section 5 (Pg 20)- Clarification provided on what CT30 and CT60 are to be used for
- Section 5 Table 4 & 5 (Pg 21/22)- Clarification regarding which target volumes the dose objectives are for
- Section5: Table 5 (Pg 22)- D50% +/- 1% for PTV2 moved from "Optimal" to "Mandatory"
- Section 5.5 (Pg 24/25)- Planning recommendations added based on pre-trial QA exercise
- Section 7.4 (Pg 28)- Recommendations for exporting CBCT data from Aria/Mosaiq
- Appendix A: QA Programme UK – Pre-trial point 7: IGRT training video now provided
- Appendix B: TROG QA Programme- QA programme updated with IGRT training/assessment information

V4.0- The following changes have been made:

- Section 1 (Pg 10) Clarification on the start date of radiotherapy
- Section 2.3 (Pg 11) Clarification on contrast. Contrast is permitted; however it is recommended to perform the planning CT scan without it
- Section 3.5 (Pg 14) Clarification on treatment volumes for each randomisation group
- Section 3.6 (Pg 16) NEW voluming checklist with clarification on outlining the GTV and CTV
- Section 3.7 (Pg 20) Nomenclature table updated
- Section 4 (Pg 22) Clarification on actions to take when replanning required
- Section 4.3 (Pg 2) Clarification on target dose objectives, with emphasis placed on PTV2
- Section 4.4 (Pg 24) Clarification on normal tissue dose constraints
- Section 4.4 (Pg 24/25) Table 6a and 6b updated to include new optimisation targets for the CTV
- Section 4.5 (Pg 25) Planning recommendations updated, with emphasis placed on PTV2
- Section 4.6 (Pg 26) Further clarification and guidance for pre-treatment checks
- Section 5 (Pg 27) Treatment delivery updated
- Section 5.2 (Pg 27/28) NEW guidance on Adaptive Tumour Focused Radiotherapy (SART & DART)
- Section 5.2.1 (Pg 27/28) NEW plan selection steps
- Sections 5.2.2 (Pg 28) NEW plan selection tips
- Section 5.3 (Pg 28) Clarification on post-treatment CBCTs
- Appendix A: QA Programme UK
    - On trial QA updated – site visits removed and IGRT/POD selection support introduced
    - Clarification on data export
    - NEW Table 7: RAIDER data checklist
- Appendix B: TROG QA Programme
    - On trial QA updated to include IGRT/POD selection support
- Appendix C: QA Benchmark Cases for Outlining and Planning moved to Appendix C
    - Additional diagnostic information for outlining case 1 included (NEW)
- Appendix D: Quick Contouring Checklist (NEW)
- Appendix E: Clarification on treatment interventions
- Appendix F: Text updated to be in line with Section 5.2
- Appendix G: Quick Reference Guide for Exporting CBCTs from ARIA (NEW)
- Appendix H: Quick Reference Guide for Exporting CBCTs from MOSAIQ (NEW)

V4.1- The following changes have been made:

- Section 4.4: Clarification on updated (V4.0) Normal Tissue Dose Constraints

V4.2 - The following changes have been made:

- Front cover updated (Pg 1/2) RAIDER Guidelines only to be used for the purpose of RAIDER

Hafeez S, et al. BMJ Open 2020; 10:e041005. doi: 10.1136/bmjopen-2020-041005

RAIDER radiotherapy planning and delivery guidelines

- Trial Management Details (Pg 3) ICR-CTSU contact details upated
- RAIDER TRIAL SUMMARY (Pg 7) Recuitment Target updated
- Trial Schema (Pg 9) Updated
- Section 1 (Pg 10) A list of RAIDER QA credentialed staff must retained in site investigator file
- Section 4.4 (Pg 24) Clarification do not compromise dose to PTV2 or PTV in order to meet the normal tissue dose constraints
- Section 5.2.1 (Pg 27-28) Clarification on Image Match and Plan Selection Steps:
    - Consider magnitude of soft tissue shift and review if over 1cm
- Section 5.2.2 (Pg 28-29) Clarification on Image Match and Plan Selection Tips:
    - Fractions must not be omitted or missed due to unfavourable positioning of normal anatomy
    - Review post-treatment CBCTs for intrafraction filling
- Section 6 (Pg 29-30) Clarification on Treatment Scheduling and Gaps:
    - Avoid gaps where possible
    - Further guidance when staff unavailable/machine breakdown
    - Compensation not expected due to toxicity
    - Involved PI
- Appendix G (Pg 50) Additional information for Aria Export

V4.3 - The following changes have been made:

- Appendix A (Pg 33) Data transfer details updated for RTTQA
- Appendix B (Pg 37) Data export and upload updated for TROG
- Appendix G (Pg 51) Please contact RTTQA for information on suitable anonymization software for CBCTs from ARIA
- Appendix I (Pg 53) Please contact RTTQA for information when exporting CBCTs from Raystation

Hafeez S, *et al. BMJ Open* 2020; 10:e041005. doi: 10.1136/bmjopen-2020-041005

RAIDER radiotherapy planning and delivery guidelines

RAIDER TRIAL SUMMARY

PROTOCOL TITLE	A Randomised phase II trial of Adaptive Image guided standard or Dose Escalated tumour boost Radiotherapy in the treatment of transitional cell carcinoma of the bladder
TARGET DISEASE	Muscle invasive bladder cancer
STUDY OBJECTIVES	To define a feasible and safe adaptive dose escalated tumour boost radiotherapy schedule for MIBC; to investigate the ability to deliver daily adaptive bladder radiotherapy and assess the impact of delivery on patient reported outcomes and health economic related measures.
STUDY DESIGN	Multicentre two stage, three arm phase II randomised controlled trial
TRIAL POPULATION	Patients receiving radical radiotherapy for muscle invasive bladder cancer
RECRUITMENT TARGET	Minimum 120 in each of two fractionation cohorts i.e. sufficient to accrue 57 evaluable DART patients per cohort
TRIAL TREATMENT	Patients will be randomised (1:1:2) between:<br>1. Standard whole bladder radiotherapy delivery (WBRT) (control)<br>2. Standard dose Adaptive tumour focused radiotherapy (SART)<br>3. Dose escalated Adaptive tumour boost radiotherapy (DART)<br>64Gy/32f and 55Gy/20f fractionation schedules are permitted. Participants in all groups will be permitted to receive concomitant radio sensitising chemotherapy. Full blood count (FBC), urea and electrolytes (U&Es) and acute toxicity will be assessed during radiotherapy. Participants in the Patient Reported Outcomes (PRO) sub-study will be asked to complete questionnaire prior to trial entry and at the end of radiotherapy.
PRIMARY ENDPOINT	Stage I: Proportion of patients meeting radiotherapy dose constraints to bladder, bowel and rectum in DART groups.<br>Stage II: Proportion of patients experiencing any ≥Grade 3 Common Terminology Criteria for Adverse Events (CTCAE) v.4 late toxicity (6-18 months post radiotherapy).
SECONDARY ENDPOINTS	Stage I:<br>• Recruitment rate<br>• Ability to deliver SART and DART<br>Stage II:<br>• Clinician reported acute toxicity<br>• PRO: acute and late bladder and bowel/rectal symptoms;

Hafeez S, et al. BMJ Open 2020; 10:e041005. doi: 10.1136/bmjopen-2020-041005

	• Health economic related measures: time for outlining, plan generation, selection and delivery, NHS resource usage subsequent to treatment;<br>• Loco-regional MIBC control<br>• Progression-free survival<br>• Overall survival
EXPLORATORY ENDPOINTS	Image Guided Radiotherapy (IGRT) endpoints:<br>• Use of adaptive plans<br>• Target coverage<br>• Online/offline concordance<br>• Dose volume analysis of adaptive vs. standard planning
FOLLOW UP	Participants will subsequently be assessed at the following intervals:<br>**6 weeks from start of radiotherapy (20f cohort only)**<br>Assessment of acute toxicity (CTCAE v.4)<br>**10 weeks from start of radiotherapy:**<br>Assessment of acute toxicity (CTCAE v.4)<br>**3 months from end of radiotherapy:**<br>Rigid cystoscopy and biopsy of tumour bed, FBC, U&Es, chest x-ray (CXR), acute toxicity (CTCAE), PRO questionnaire (if participating in sub-study).<br>**6 months from end of radiotherapy:**<br>Flexible cystoscopy, FBC, U&Es, CXR or CT chest, CT abdomen and pelvis, late toxicity (CTCAE, RTOG), PRO(if participating in sub-study)<br>**9months from end of radiotherapy:**<br>Flexible cystoscopy, late toxicity<br>**12months from end of radiotherapy:**<br>Flexible cystoscopy, CT abdomen and pelvis, CXR or CT chest, late toxicity, PRO(if participating in sub-study)<br>**18months from end of radiotherapy:**<br>Flexible cystoscopy, CXR or CT chest, late toxicity, PRO(if participating in sub-study)<br>**24 months from end of radiotherapy:**<br>Flexible cystoscopy, CT abdomen and pelvis, CXR or CT chest, late toxicity, PRO(if participating in sub-study)<br>**Yearly to year 5:** Flexible cystoscopy, CXR or CT chest, late toxicity

Annually thereafter: Survival and disease status

RAIDER radiotherapy planning and delivery guidelines

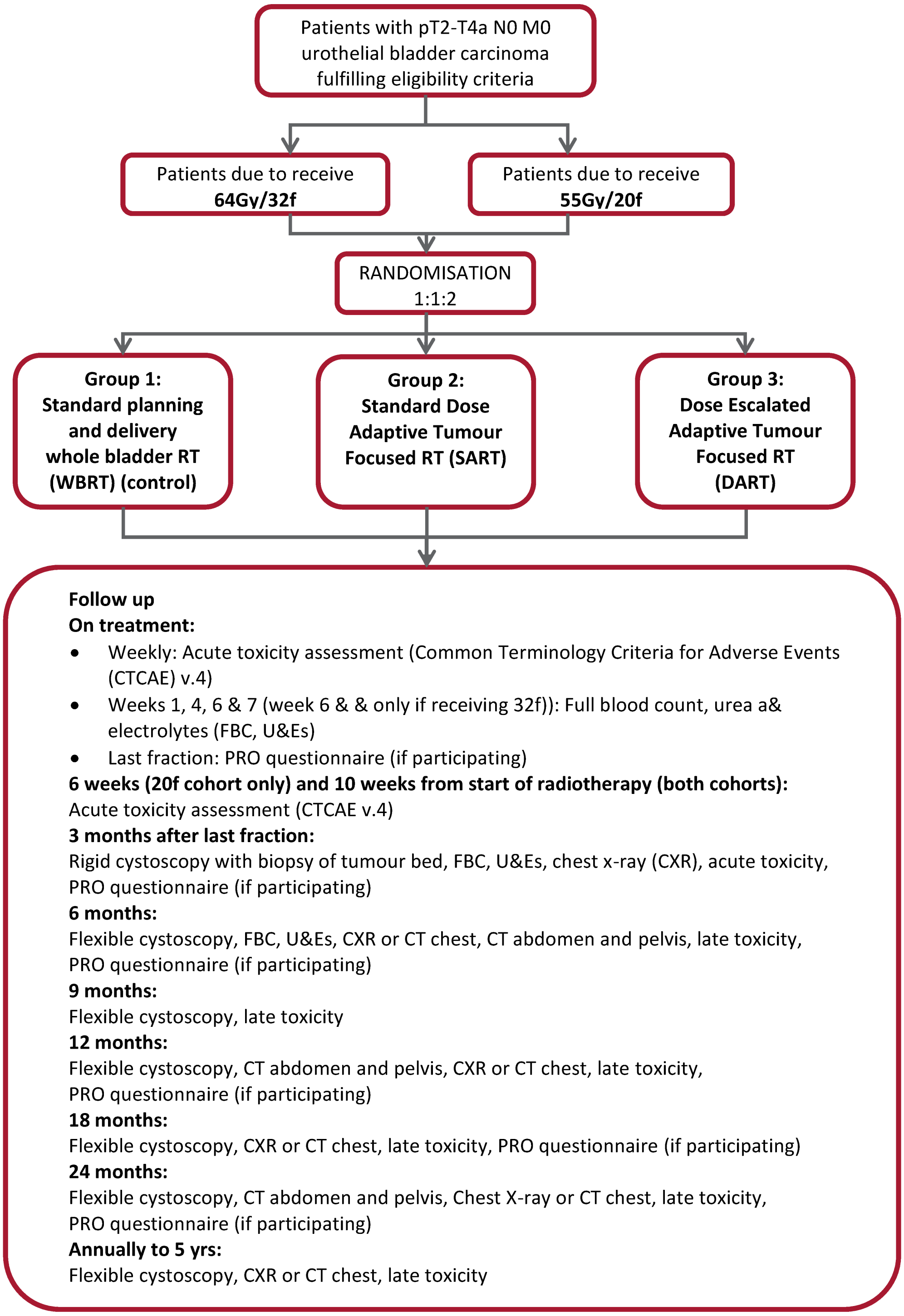

RAIDER radiotherapy planning and delivery guidelines

1 INTRODUCTION

All patients will be planned to receive radical daily radiotherapy delivered in 32 or 20 fractions. Patients will be randomised between 3 groups;

1. Standard Whole Bladder Radiotherapy (WBRT) (control)
2. Standard dose Adaptive tumour focused Radiotherapy (SART)
3. Dose escalated Adaptive tumour boost Radiotherapy (DART)

In all 3 groups concomitant therapy is permitted at the local investigators discretion.

Radiotherapy should ideally commence within 6 weeks following randomisation. However up to 10 weeks is permitted, to allow sufficient time for chemotherapy and/or radiotherapy planning

Participants allocated to standard planning and delivery (control arm, ***group 1***) will have:

- One (1) radiotherapy plan generated to deliver all fractions on an empty bladder.

The IGRT process is as detailed in the NRIG IGRT 2012 report for this patient group. The NRIG IGRT report contains detailed guidelines for IGRT practice in UK. (It is in the RAIDER trial documents on the TROG website for reference).

Participants allocated to adaptive planning (***group 2 and 3***) will have:

- Three (3) radiotherapy plans generated (small, medium and large).
- A simultaneous integrated boost delivered at conventional dose (group 2) or dose escalation (group 3) to the bladder tumour in a single phase IMRT technique.
- All fractions treated on a partially filled bladder.
- A cone beam CT (MV or kV) taken prior to each treatment delivery to select the most appropriate 'plan of the day' depending on the bladder volume size.

A comprehensive QA programme will be implemented for the RAIDER trial. This will include pre-accrual and during accrual components. Selection of appropriate treatment plans for the adaptive planning group will be independently monitored as part of the on-going RTQA process.

Plan selection is authorised to be carried out only by site personnel who have attained concordance with the gold standard for PTV selection through either RTTQA IGRT QA credentialing for UK centres and TROG IGRT credentialing for Australia/NZ. This is to ensure they have the advanced level skills required for the study. A record of QA credentialed staff should be retained in the site investigator file.

This document should only be used for the management of patients in the RAIDER trial.

Hafeez S, *et al. BMJ Open* 2020; 10:e041005. doi: 10.1136/bmjopen-2020-041005

2 PLANNING CT SCAN FOR RADIOTHERAPY

2.1 Patient preparation

2.1.1 Group 1, 2 and 3: General Preparation

Ideally all patients should be encouraged to empty the rectum of flatus and faeces. The routine use of micro enemas (e.g. relaxit) is permissible if it is standard local practice.

Bladder preparation is dependent on patient randomisation.

2.1.2 Group 1: Empty Bladder

Ensure patient has an empty bladder. Therefore all patients should be asked to void immediately before planning CT scan (CT0) is performed and not to drink fluids for 30minutes before the scan.

2.1.3 Group 2 and 3: Bladder Filling

30 minutes prior to the planning CT scan, patients are instructed to empty their bladder and then drink 350ml of water. Two planning scans will be acquired to inform pattern of bladder filling over time:

- The first scan will be at 30 minutes (CT30) following drinking
- The second scan will be at 60 minutes (CT60) following drinking.

Voiding is not permitted between the 2 scans; however if unavoidable, the CT30 scan should be used for planning.

2.2 Patient positioning

All patients will be scanned and treated supine with arms displaced out of the radiotherapy field, using appropriate immobilisation techniques.

2.3 Planning CT acquisition, scan limits and slice thickness

Planning CT scans will be performed in the treatment position at CT slice thickness 3mm or less. It is recommended that the planning CT is performed without contrast, but the use of contrast is permitted if it is local clinical practice. Recommended scanning levels are at least 4cm above the dome of the bladder to 2cm below ischial tuberosities.

For patients treated in group 2 and 3, the planning CT scan is performed (as above) at 'Time=30 minutes' (scan 1, CT30). A second planning CT is acquired at 'Time= 60 minutes' (scan 2, CT60) from end of drinking. The time of each scan is recorded.

2.4 Planning CT export, fusion and evaluation of bladder filling

The planning CT scan is exported via DICOM transfer to the treatment planning system for localization.

For patients treated in group 2 and 3 the bladder filling must be assessed. In order to do so fuse both planning CT scans in the treatment planning system. <u>CT30 will be the primary data set</u> and CT60 will be the fused secondary dataset. To evaluate bladder filling, the whole bladder is localized on the CT30 and on the CT60 scan.

Hafeez S, *et al. BMJ Open* 2020; 10:e041005. doi: 10.1136/bmjopen-2020-041005

- If the difference in bladder filling between the 2 planning scans is less than 50ml i.e. no significant filling occurs all contours are to be created on CT30.
- If difference is greater than 50mls i.e. filling occurs, the large GTV and CTV will be created on CT60.These shall be used to create PTV2 and PTV for the large plan

Please note all planning and dose calculation is to be done on CT30 (See section 4), therefore all volumes should be assigned to a single structure set on the CT30 scan.

RAIDER radiotherapy planning and delivery guidelines

3 LOCALISATION OF THE TARGET VOLUME AND ORGANS AT RISK

3.1 Volume definition

Volumes will be defined according to the International Commission on Radiation Units and Measurements (ICRU) report 50, supplement report ICRU 62: Prescribing, Recording and Reporting Photon Beam Therapy and ICRU 83: Prescribing, Recording and Reporting Photon-Beam Intensity Modulated Radiotherapy (IMRT). Outlining should be carried out with the aid of all diagnostic information including position of fiducial markers, surgical bladder map, MRI and CT scans.

3.2 Gross tumour volume

The gross tumour volume (GTV) is defined as the bladder tumour/bed. It will be delineated using position of fiducial markers (where available), diagnostic imaging (imaging prior to neoadjuvant chemotherapy where applicable) and the surgical bladder map (where available). When delineating the tumour any extravesical tumour should be included in GTV as should pathological bladder wall thickening unless clearly not due to tumour. If no tumour is visible, the appropriate section of the bladder should be included based on surgical bladder map +/- discussion with urologist who performed TURBT. In these circumstances consider repeating TURBT and placing fiducial markers adjacent to resection scar whenever possible.

For patients treated in group 2 and 3, if the difference in bladder filling between CT30 and CT60 is greater than 50ml i.e. filling occurs, GTV is to be contoured on both scans.

3.3 Clinical target volume

The clinical target volume (CTV) is the contour encompassing the tumour/bed (GTV), the whole bladder and any area of extravesical spread. The CTV should also include 1.5cm of prostatic urethra in males or 1cm of urethra in females if tumour is at the base of bladder or if distant CIS is present.

For patients treated in group 2 and 3, if the difference in bladder filling between CT30 and CT60 is greater than 50ml i.e. filling occurs, CTV is to be contoured on both scans.

3.4 Organs at risk

Organs at risk (OAR) will be outlined as solid structures by defining their outer wall. All OAR should be outlined on the CT0 for group 1 and CT30 only for group 2 and 3. The following OARs should be contoured:

- Rectum:

The rectum is outlined to include the full circumference and rectal contents. Outlining should extend from the lowest level of ischial tuberosities to the recto-sigmoid junction. The recto-sigmoid junction will be defined as the level at which there is an anterior inflection of the bowel, best appreciated on sagittal reconstructions on the CT planning scan.

- Other bowel:

The small and large bowel (including sigmoid colon) will be outlined as a single structure. The entire small and large bowel visible on relevant levels of the planning scan will be outlined as individual bowel loops. The superior extent of outlining should be 2cm beyond the superior extent of PTV (group 1) and Large PTV (group 2 and 3).

- Femoral heads:

Both the femoral heads are outlined to the bottom of the curvature of their heads (femoral necks are not included).

3.5 Planning target volume

The CTV(s) will be expanded, as below, to create the PTV(s).

For the tumour focused RT (group 2 and 3) the GTV(s) will be expanded, as below to create the PTV2s.

3.5.1 Group 1: standard whole bladder RT

For patients in group 1, receiving standard whole bladder RT, a single PTV will be created.

1. PTV= CTV with anisotropic margin applied, as per Table 1.

3.5.2 Group 2 and 3: adaptive tumour focused RT (standard dose and dose escalated)

For patients in group 2 and 3, receiving adaptive tumour focused RT a library of 3 PTVs will be created, from the CTV(s) (Table 1):

1. PTV_Sm
2. PTV_Med
3. PTV_Lar

- For patients in group 2 and 3, if the difference in bladder filling between CT30 and CT60 is less than 50ml i.e. filling does not occur, the PTV_Sm, PTV_Med and PTV_Lar will be produced from the 30 minute CTV.

- However, if the difference in bladder filling between CT30 and CT60 is greater than 50ml i.e. filling occurs, the PTV_Sm and PTV_Med will be produced from the 30 minute CTV. The PTV_Lar will be produced from the 60 minute CTV, as described in Table 1.

RAIDER radiotherapy planning and delivery guidelines

Table 1: CTV to PTV Expansion Details

Patient Randomisation	CT data set	PTV	CTV to PTV Expansion (cm)				
			Laterally	Anteriorly	Posteriorly	Superiorly	Inferiorly
Group 1<br>Standard Whole Bladder	CT0	PTV	0.8	1.5	1.2	1.5	0.8
Group 2 and 3<br>Adaptive Tumour Focused	CT30	PTV_Sm	0.5	0.5	0.5	0.5	0.5
	CT30	PTV_Med	0.5	1.5	1.0	1.5	0.5
	If CT60-CT30 <50mls then						
	CT30	PTV_Lar_30	0.8	2.0	1.2	2.5	0.8
	If CT60-CT30>50mls then						
	CT60	PTV_Lar_60	0.5	1.5	1.0	1.5	0.5

Additionally 3 PTV2s will also be created, from the GTV(s) (Table 2):

1. PTV2_Sm
2. PTV2_Med
3. PTV2_Lar

- For patients in group 2 and 3, if the difference in bladder filling between CT30 and CT60 is less than 50ml i.e. filling does not occur, the PTV2_Sm, PTV2_Med and PTV2_Lar will be produced from the 30 minute GTV.
- However, if the difference in bladder filling between CT30 and CT60 is greater than 50ml i.e. filling occurs, the PTV2_Sm and PTV2_Med will be produced from the 30 minute GTV. The PTV2_Lar will be produced from the 60 minute GTV, as described in Table 2.

Hafeez S, *et al. BMJ Open* 2020; 10:e041005. doi: 10.1136/bmjopen-2020-041005

RAIDER radiotherapy planning and delivery guidelines

Table 2: GTV to PTV2 Expansion details (group 2 and 3 only)

Patient Randomisation	CT data set	PTV2	GTV to PTV2 Expansion (cm)				
			Laterally	Anteriorly	Posteriorly	Superiorly	Inferiorly
Group 1<br>Standard Whole Bladder	Not applicable						
Group 2 and 3<br>Adaptive Tumour Focused	CT30	PTV2_Sm	0.5	0.5	0.5	0.5	0.5
	CT30	PTV2_Med	0.5	1.5	1.0	1.5	0.5
	If CT60-CT30 <50mls then						
	CT30	PTV2_Lar_30	0.8	2.0	1.2	2.5	0.8
	If CT60-CT30>50mls then						
	CT60	PTV2_Lar_60	0.5	1.5	1.0	1.5	0.5

3.6 Voluming Checklist

1. For group 2 and 3, the inferior border of the CTV should be the same on both the CT30 and CT60 following fusion, Figure 1.

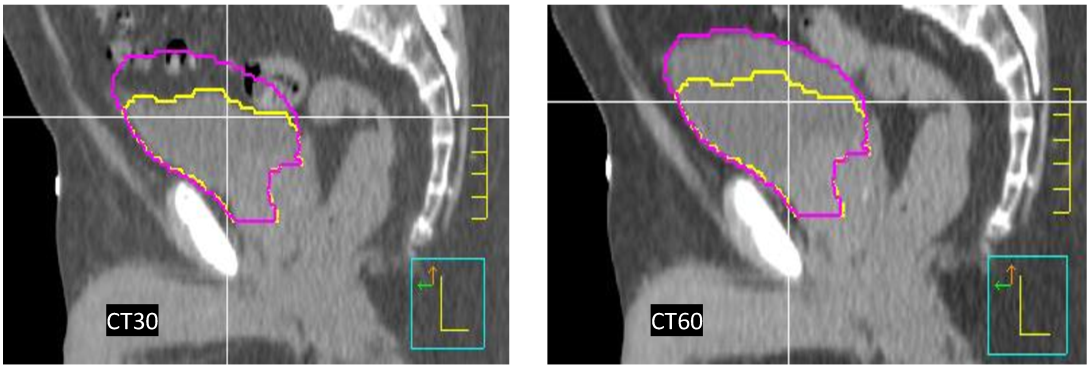

Figure 1: Inferior border of CTV anticipated to be at same level for both scans. Can be achieved by copying inferior contour on fused scan. This ensures that changes in volume are due to filling and not to variation/contouring error between the 2 scans.

RAIDER radiotherapy planning and delivery guidelines

2. For Groups 1, 2 and 3 please ensure that 1.5cm of prostatic urethra in males or 1cm of urethra in females is included when the tumour is at the bladder base or if distant CIS is present. Please contact RTTQA if further guidance is required on this aspect of outlining.

3. For Group 2 and 3, the CTV drawn on CT60 should not be within/smaller than the CTV drawn on the CT30. Thus, the CTV drawn on CT60 should encompass the CTV drawn on CT30. This could occur as a result of contouring error, however it is also possible that bowel motion/filling between the 2 scans may also cause this (see Figures 2a-2d). In these circumstances it is advised that the CTV drawn on CT60 is summed to include the CTV drawn on CT30; allowing all excursions of the bladder to be included in the volume. This can be done by application of Boolean operators within the treatment planning system used.

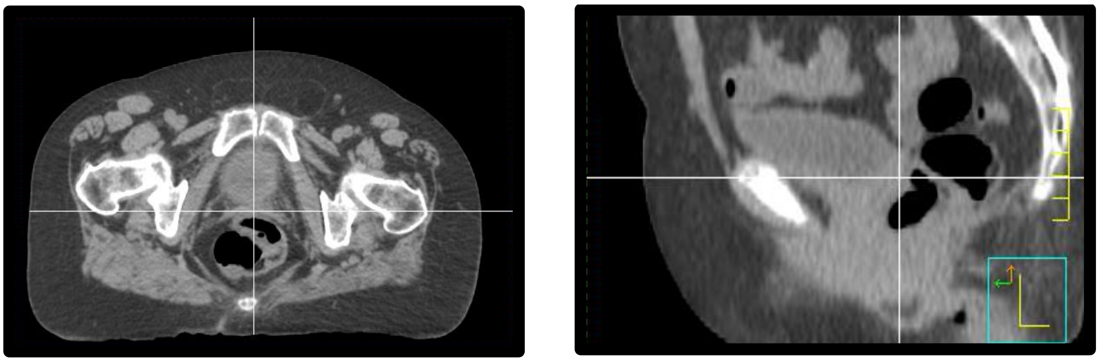

Figure 2a: CT30 scan.

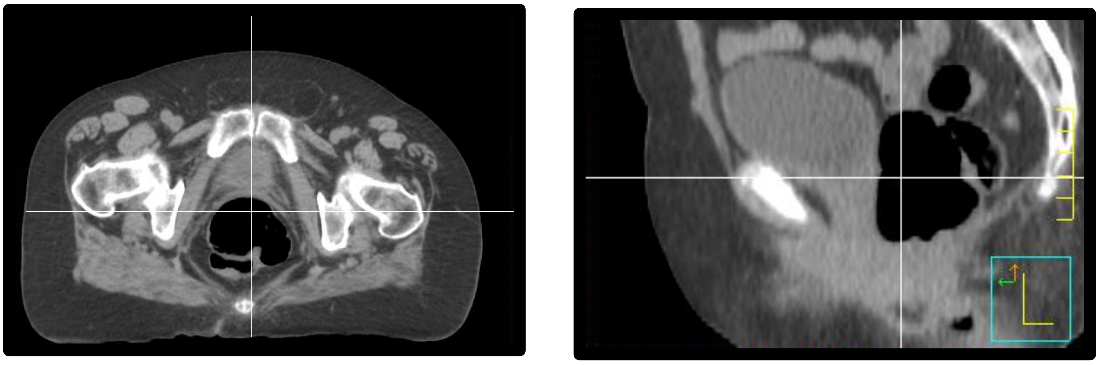

Figure 2b: CT60 scan. Bladder filling occurs but is also pushed anterior due to rectal distension secondary to flatus

RAIDER radiotherapy planning and delivery guidelines

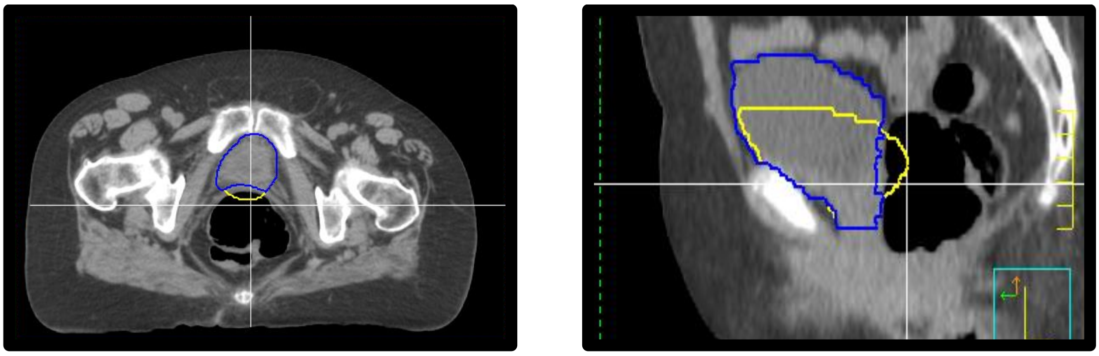

Figure 2c: Yellow contour reflects CTV on CT30. Blue contour reflects CTV on CT60. (Difference between two volumes >50cc).

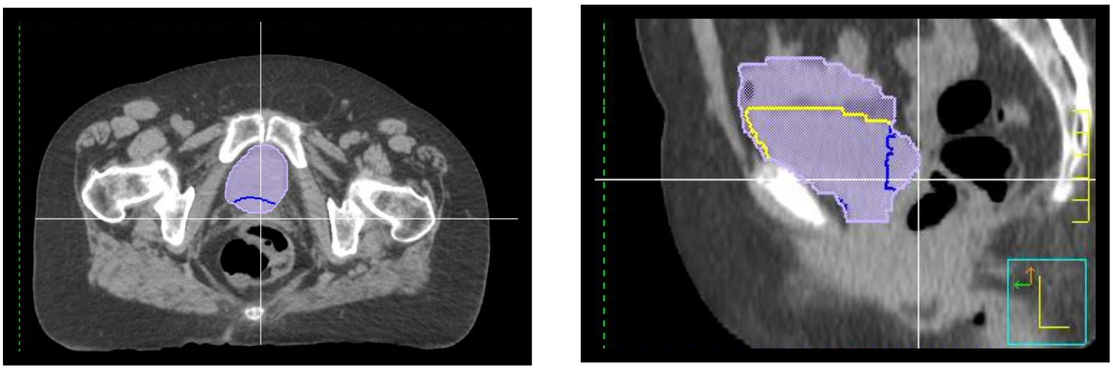

Figure 2d: Boolean operator applied to create summed CTV (purple) from which PTV_Lar_60 (and PTV2_Lar_60) will be created.

4. The GTV should not exceed beyond the CTV outline. Where there is extra-vesical spread included in the GTV, the CTV must be extended outside the bladder wall to include all the GTV. The CTV should be summed to include all the GTV and should share same outer-contour, see Figure 3a-3c.

- The CTV_30 should encompass the GTV_30 and share the same outer contour.
- The CTV_60 should encompass the GTV_60 (and thus the GTV_30) and should share the same outer contour.

RAIDER radiotherapy planning and delivery guidelines

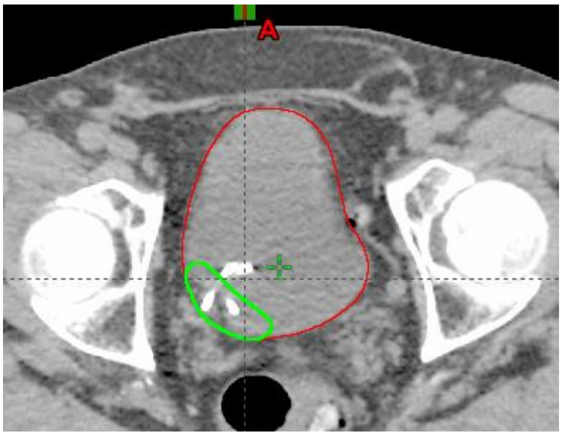

Figure 3a: CT30 GTV (green) shares same bladder wall as CTV (red)

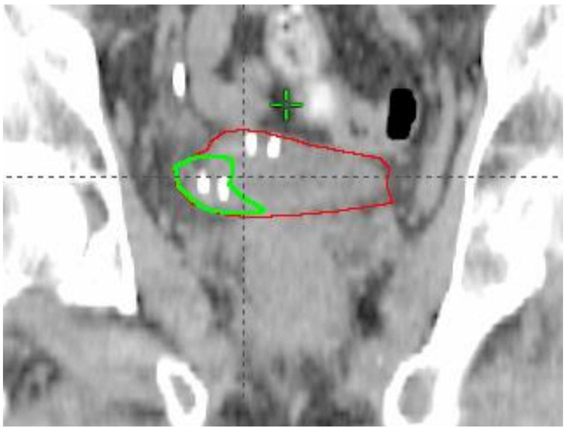

Figure 3b: CT30 GTV (green) shares same bladder wall as CTV (red)

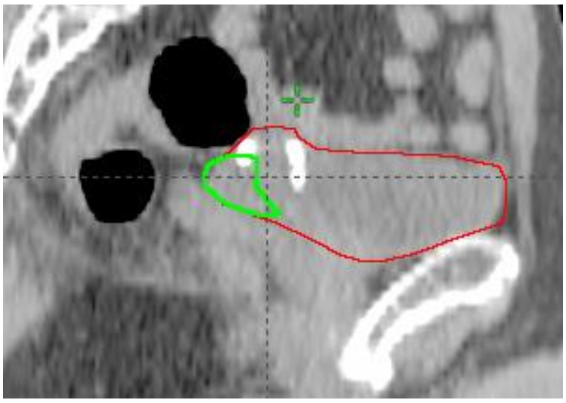

Figure 3c: CT30 GTV (green) shares same bladder wall as CTV (red)

Hafeez S, *et al. BMJ Open* 2020; 10:e041005. doi: 10.1136/bmjopen-2020-041005

5. The GTV_60 should generally extend further superiorly and anteriorly, in comparison with the GTV_30, to account for expansion upon filling.

6. The PTV_Lar should encompass the PTV_Med.

7. The PTV2 should not exceed beyond the corresponding PTV outline. For planning purposes, the PTV2 should share the same outer contour as the corresponding PTV outline, i.e. it should ‘appear’ attached to the outer contour of the PTV.

8. The clinician should indicate to the planner which CT data set (CT30 or CT60) has been used to create the large target volumes.

9. The OTHER_BOWEL should extend to 2cm beyond the largest PTV.

10. The rectum should extend to the lowest level of the ischial tuberosities.

A “Quick Contouring Checklist” has been provided in Appendix E to ensure the above has been successfully achieved.

3.7 Nomenclature

Consistent naming of contoured structures used in radiotherapy treatment planning is essential to facilitate the comparison of dose-volume statistics across patients for quality assurance and outcomes analysis. Maintaining consistency in structure names is particularly important (and challenging) in multi-institutional clinical trials, in which treatment planning data are collected from many participating institutions. A scheme for uniform naming of contoured structures for RAIDER is provided in the following table. The following names must be used for treatment planning of all trial patients.

Table 3: RAIDER Trial Target Volume and OAR Nomenclature

Structure Name	Description
GTV_0	Contouring the primary bladder tumour for patients randomised to Group 1 (WBRT) is not required for trial purposes. Local standard practice should be followed if contouring GTV aids subsequent CTV delineation (on CT_0)
GTV_30	Primary bladder tumour contoured on CT30 for group 2 and 3

Hafeez S, et al. BMJ Open 2020; 10:e041005. doi: 10.1136/bmjopen-2020-041005

GTV_60	Primary bladder tumour contoured on CT60 for group 2 and 3 if the difference in bladder filling between CT30 and CT60 is greater than 50ml
CTV_0	GTV + whole bladder + extravesical spread (+ 1.5cm of prostatic urethra in males or 1cm of urethra in females if tumour is at the base of bladder or if distant CIS is present) contoured on CT0 for group 1
CTV_30	GTV + whole bladder + extravesical spread (+ 1.5cm of prostatic urethra in males or 1cm of urethra in females if tumour is at the base of bladder or if distant CIS is present) contoured on CT30 for group 2 and 3
CTV_60	GTV + whole bladder + extravesical spread (+ 1.5cm of prostatic urethra in males or 1cm of urethra in females if tumour is at the base of bladder or if distant CIS is present) contoured on CT60 for group 2 and 3
PTV_Std	CTV_0 + anisotropic margin (see table 1)<br>**<u>For Standard Whole Bladder-group 1 only</u>**
PTV_Sm	CTV_30 + margin
PTV_Med	CTV _30 + margins
PTV_Lar_30	CTV_30 +margins, **when no filling occurs between CT30 and CT60)**
PTV_Lar_60	CTV_60 +margins, **when filling occurs between CT30 and CT60)**
PTV2_Sm	GTV_30 + margin
PTV2_Med	GTV_30 +margins
PTV2_Lar_30	GTV_30 + margins, **when no filling occurs between CT30 and CT60)**
PTV2_Lar_60	GTV_60 + 0.5 – 1.5cm margins, **when filling occurs between CT30 and CT60)**
PTV_Sm-PTV2_Sm	PTV_Sm minus PTV2_Sm
PTV_Med-PTV2_Med	PTV_Med minus PTV2_Med
PTV_Lar-PTV2_Lar	PTV_Lar minus PTV2_Lar
RECTUM	Rectum
OTHER_BOWEL	Small and large bowel (including sigmoid colon)
FEMORALJOINT_L (UK) FemJoint_L (Aus)	Left femoral head (ball only)
FEMORALJOINT_R (UK)FemJoint_R (Aus)	Right femoral head (ball only)

4 PLANNING

It is mandatory for all RAIDER patients to be CT planned. In general, the method of treatment planning will vary from site to site and should be specified in each centre's process document.

For group 1 the use of three or four 3D conformal radiotherapy (3DCRT) fields, five to seven static fields intensity modulated radiotherapy (IMRT) or volumetric modulated radiotherapy (VMAT) treatments (e.g. RapidARC, VMAT, Tomotherapy) are acceptable. Please inform the relevant QA group and the CTU if a group 1 patient requires re-planning (or re-scanning) during treatment.

For group 2 and 3 the aim of the plan is to do partial bladder sparing while maintaining the PTV prescription dose, use of 5-7 static fields IMRT (5 fields are preferred due to shorter treatment time) or VMAT is recommended. The use of forward planned simple IMRT ('field in field') or tomotherapy are also acceptable alternatives. The same technique should be used for all patients randomised to group 2 and 3. All plan and dose calculation is to be done on CT30 irrespective of filling.

Please inform the relevant QA group and the CTU if a group 2 or 3 patient requires re-planning (or re-scanning) during treatment.

The local investigator should ensure appropriate quality assurance methodologies are in place for the chosen planning technique.

4.1 Standard Planning

For patients randomised to group 1 standard planning a single plan will be created.

- Plan_Std = PTV_Std

4.2 Adaptive Tumour Focused Planning

For those randomised to adaptive planning a series of 3 plans will be created using the PTV Small, PTV Medium and PTV Large and the corresponding PTV2. The isocentre for all 3 plans must be identical.

- Plan_Sm = PTV_Sm& PTV2_Sm
- Plan_Med = PTV_Med& PTV2_Med
- Plan_Lar = PTV_Lar_30 and PTV2_Lar_30 or PTV_Lar_60 and PTV2_Lar_60, depending on filling.

For IMRT planning centres may prescribe plan to either a mean or median dose to PTV2, depending on their normal practice, as it is anticipated in an optimised plan the difference between these two parameters will be minimal. For 3D-CRT the prescription should be 100% at the ICRU reference point.

The prescription doses for RAIDER patients are detailed in Table 4.

RAIDER radiotherapy planning and delivery guidelines

Table 4: Prescription doses

Patient Randomisation	Volume	Dose (Gy)	Fractions	Dose per fraction (Gy)	Dose (Gy)	Fractions	Dose per fraction (Gy)
**Group 1** **Standard plan**	PTV_Std	64	32	2	55	20	2.75
**Group 2** Standard dose adaptive tumour focused **(SART)**	PTV2	64	32	2	55	20	2.75
	PTV (PTV – PTV2)	52	32	1.625	46	20	2.3
**Group 3** Dose escalated adaptive tumour boost RT (DART)	PTV2	70	32	2.1875	60	20	3
	PTV (PTV – PTV2)	52	32	1.625	46	20	2.3

4.3 Target volume dose objectives

Three dimensional dose distributions should be produced. The dose distribution should be assessed for coverage of the PTV and normal tissues using appropriate transverse sagittal and coronal views. The following optimal and mandatory target volume dose constraints are proposed:

Table 5: Target dose objectives

Volume	Dose Constraints	Optimal	Mandatory
PTV2	$D_{98\%}$	≥95% of prescribed dose	≥90% of prescribed dose
	*$D_{50\%}$	-	+/- 1% of prescribed dose
	$D_{2\%}$	≤105% of prescribed dose	≤107% of prescribed dose
PTV (PTV – PTV2)	D98%	≥95% of prescribed dose	≥90% of prescribed dose

*Please note that D50% constraint refers only to PTV2. PTV D50% is likely to be exceeded depending on size of PTV2. Therefore no compromise to PTV2 coverage should be made at the expense of achieving D50% PTV constraint.

4.4 Normal tissue dose constraints

The dose to OAR should be minimised. The following dose volume constraints are a guide.

Ideally, for Groups 2 & 3 optimal constraints for other bowel should be met for Plan 1 (small) and mandatory constraints for other bowel should be met for Plan 2 (medium). Provided the Plan 3 (large) has been adequately optimised it is recognised that some of the rectum and other bowel mandatory constraints may not be achieved.

Likewise for group 1 patients, provided the plan has been suitably optimised, some rectum and other bowel dose tolerances may be exceeded due to inclusion within the PTV. It is at the local Principal Investigator's discretion to accept the OAR doses and should be noted on the plan assessment form. The DVH assessment for each plan should be with the overall prescribed dose.

For patients in group 3, if mandatory dose constraints are not met on the medium plan advice must be sought from the RAIDER QA team.

For this reason it is recommended for adaptive planning the medium plan is produced first, this should then be copied and the relevant objectives and prescription volume are changed for the small and large plans. **Dose to the PTV2 or PTV must not be compromised in order to meet the normal tissue constraints.**

Table 6a: OAR Dose Constraints for 32-fraction schedule

**Rectum**	**V30Gy**<br>**V50Gy**<br>**V60Gy**<br>**V65Gy**<br>**V70Gy**	**80%**<br>**60%**<br>**50%**<br>**30%**<br>**15%**	
**Femoral Heads**	**V50Gy**	**50%**	
**Other Bowel**	 **V45Gy**<br>**V50Gy**<br>**V55Gy**<br>**V60Gy**<br>**V65Gy**<br>**V70Gy**<br>**V74Gy**	**Optimal**<br>**116cc**<br>**104cc**<br>**91cc**<br>**73cc**<br>**23cc**<br>**0cc**<br>**0cc**	**Mandatory**<br>**139cc**<br>**127cc**<br>**115cc**<br>**98cc**<br>**40cc**<br>**10cc**<br>**0cc**
****Whole bladder constraint (i.e. CTV)**	 **V60Gy**<br>**V65Gy**	**Optimal**<br>**50%**<br>**40% only in Group 3 (DART)**<br>**Otherwise 0% in Group 2 (SART)**	**Mandatory**<br>**80%**<br>**50% only in Group 3 (DART)**<br>**Otherwise 5% in Group 2 (SART)**

**Body-PTV (Normal Tissue)**	D_{1cc}	**≤105% of prescribed dose**	**≤110% of prescribed dose**

******Whole bladder (CTV) constraint specified above (Table 6a) should be used to **inform plan optimisation**. Please contact the RAIDER QA team if you fail to meet this constraint.

Bladder outside PTV2 (i.e. CTV-PTV2) meeting V60Gy and V65Gy absolute constraint of 80% and 50% respectively will be collected for reporting of primary endpoint.

Table 6b: OAR Dose constraints for 20-fraction schedule

**Rectum**	**V25Gy<br>V41.7Gy<br>V50Gy<br>V54.2Gy<br>V58.3Gy**	**80%<br>60%<br>50%<br>30%<br>15%**	
**Femoral Heads**	**V41.7Gy**	**50%**	
**Other Bowel**	 **V37.5Gy<br>V41.7Gy<br>V45.8Gy<br>V50Gy<br>V54.2Gy<br>V58.3Gy<br>V61.7Gy**	**Optimal<br>116cc<br>104cc<br>91cc<br>73cc<br>23cc<br>0cc<br>0cc**	**Mandatory<br>139cc<br>127cc<br>115cc<br>98cc<br>40cc<br>10cc<br>0cc**
*****Whole bladder constraint (i.e. CTV)**	**V50Gy<br>V54.2Gy**	**Optimal<br>50%<br>40% only in Group 3 (DART)<br>Otherwise 0% in Group 2 (SART)**	**Mandatory<br>80%<br>50% only in Group 3 (DART)<br>otherwise 5% in Group 2 (SART)**
**Body-PTV (Normal Tissue)**	D_{1cc}	**≤105% of prescribed dose**	**≤110% of prescribed dose**

*******Whole bladder (CTV) constraint specified above (Table 6b) should be used to **inform plan optimisation**. Please contact the RAIDER QA team if you fail to meet this constraint.

Bladder outside PTV2 (i.e. CTV-PTV2) meeting V50Gy and V54.2Gy absolute constraint of 80% and 50% respectively will be collected for reporting of primary endpoint.

Normal tissue dose constraints and dose volume histograms (DVH) for 20 fraction schedule are presented in Table 6b (recalculated from above using linear quadratic equation, assuming that all α/β of organs at risk is 3 and that dose to tumour boost is delivered in 3Gy per fraction).

4.5 Planning recommendations

- Produce the medium plan first.

- For the planning priorities should be as follows, in order of importance:
    1. ***Achieve 95% coverage of PTV2***
    2. ***Achieve 95% coverage of the PTV***
    3. Achieve mandatory OAR dose constraints
    4. Achieve optimal OAR dose constraints
    5. Reduce areas of high dose within PTV-PTV2 especially away from boost site

Therefore, for the majority of cases dose coverage of the PTV_2 and PTV volumes should not be compromised to reduce dose to OAR without first contacting RTTQA for advice.

- For Group 2 & 3, review all three plans together after planning using the plan comparison function. The dose to the RECTUM and OTHER_BOWEL should stay the same or get progressively higher as the PTV size gets larger i.e. the dose statistics should not be better for the large plan than the small or medium plan.
- If throughout the course of treatment any patient requires re-planning please contact the relevant QA group.
- If the PTV volume is outside the patient's body please contact the relevant QA group.

4.6 Pre-Treatment checks

To minimise risk of error at the time of importing, exporting and plan selection, please ensure that each beam name and ID reflects the assigned plan i.e. Sm_Plan.

At the time of plan exporting, it is recommended to find a way of ensuring that centres' local record and verify systems cannot mix beams from different plans. For example create each plan with slightly different contributions from each field so that only the correct combination of beams can be chosen on any given day (Applicable only to Mosaiq). This could be done by adding 2 points diagonally on the isocentre slice with a dose close to the 100% isodose. Then all beams can be assigned from a plan to each of the points as the reference point.

Please ensure that the safe scheduling of multiple treatment fields and recording the dose delivered is considered for RAIDER patients. Additionally, please ensure that the safe scheduling of imaging only fields is considered. These procedures will be captured in the process document. If you have any questions please contact the relevant QA group.

5 TREATMENT DELIVERY

It is important to ensure that patients follow the bladder preparation instruction for their group as they have done at the planning CT scan appointment (i.e. empty for group 1 and partially full for group 2 and 3).

5.1 Standard whole bladder RT (group 1)

Acquire pre-treatment cone beam CT images using a full pelvis scan. Image match and register bone, according to the recommendations stated in the NRIG IGRT report.

Make required corrections/shifts as per departmental practice.

The acquired cone beam CT image can be used for assessment of target coverage at the individual department's discretion. Such use will be documented. Any changes made on the basis of the scan should be reported in the CRF and to RTTQA (including exposures not resulting in treatment because of patient factors).

Schedule imaging for Group 1 patients, as per departmental protocol. In addition to this please ensure a post-treatment CBCT is taken in the first week and weekly for these patients.

5.2 Adaptive Tumour Focused RT (SART and DART)

Acquire pre-treatment cone beam CT image and register bone according to the recommendations stated in the NRIG IGRT report. Make any corrections according to departmental practice.

An appropriately RTTQA trained and accredited healthcare professional should review the bone matched CBCT, to assess bladder filling and PTV coverage. The following steps should assist centres when assessing the CBCTs.

The aim in treating adaptive patients is to use the **smallest plan possible**, so that the dose is minimised to the OARs without compromising both PTV and PTV2 coverage.

The steps below describe how this can be best achieved at plan selection. It is important to review overall bladder filling, and PTV2 boost position as determined on the planning CT scans before plan selection.

5.2.1 Image Match and Plan Selection Steps:

1. Following CBCT, the bladder filling and size should be checked against CTV_30 contour.
2. If the bladder is of similar shape and size to the CTV at planning (i.e. CTV_30), then the small plan should be preferably considered in the first instance for treatment.
3. Once the bladder filling and shape has been assessed begin to review the appropriate PTVs. An appropriate plan provides suitable coverage of the PTV2 and PTV, with minimal normal tissue irradiation.
4. Manual (soft tissue) moves should be made to ensure the bladder is adequately covered whilst selecting the smallest plan possible to spare normal tissue. Please be mindful of the magnitude of the soft-tissue shift, moves greater than 1cm can impact on the accuracy of the expected dosimetry. Discuss with planning department if this shifts over 1cm occur and contact RTTQA retrospectively.

5. Additional manual moves can be undertaken if felt it could further optimise PTV2 coverage. Manual moves prioritising coverage to the boost region over the normal bladder wall is permitted if it avoids excessive normal tissue irradiation that would have occurred by selecting a larger plan. Again, please be mindful of the magnitude of the soft-tissue shift, moves greater than 1cm can impact on the accuracy of the expected dosimetry. Discuss with planning department if this shifts over 1cm occur and contact RTTQA retrospectively.
6. Assess the OAR. Review if they are similar to planning scan. Additionally it is important to check where they are relative to PTV2 i.e. ensuring not in PTV2, especially for DART patients.

The actions taken for each patient must be confirmed by a second appropriately trained and accredited healthcare professional. Once agreement has been reached any correction should be performed and the plan selection agreed and confirmed for treatment.

5.2.2 Image Match and Plan Selection Tips:

This guidance is provided to assist RAIDER trained staff with potential solutions for scenarios that may arise on treatment. Further guidance is provided in APPENDIX E. Fractions must not be omitted or missed due to unfavourable positioning of normal anatomy such as rectal distention due to flatus or faeces.

- Please only choose a large plan if the bladder has filled. (E.g. *If the bladder has filled more than the CTV_30, and the patient has a CTV_60, overlay the CTV_60 to assess the magnitude of the filling).*
- Please consider the nature of bladder filling. We would expect filling to occur in the anterior and superior direction.
- Please be mindful of minimising image match and plan selection time.
- If the bladder is significantly smaller than the CTV 30 contour at planning, it is likely that the PTV2 boost is in the incorrect position and, or does not achieve adequate normal bladder sparing. In these circumstances patient should not proceed but be removed from the couch, and encouraged to fill the bladder by drinking further, and/or increasing the time interval of image acquisition. The local PI and RTTQA/TROG should be informed.
- In the event that the bladder has over filled and none of the library PTVs cover the entire bladder despite manual move the patient should be asked to minimally void and the CBCT is repeated. If this is not possible, patient should void completely and restart drinking protocol but consider reducing the time interval for CBCT acquisition (see appendix C and D). In these circumstances a member of the clinical team should also be notified to ensure the patient is not in urinary retention. If the large plan is being regularly selected, during a single treatment week, please contact the RTTQA/TROG to discuss this patient.
- Caution should be taken when changing the drinking protocol. Please consider changing one aspect of the protocol at a time, i.e. change the timing only or change the amount of water only.
- If no contours are suitable because of rectal gas, then remove the patient from the bed and ask them to void. Repeat the above steps. If contours still not suitable select the most suitable plan, i.e. optimising coverage to PTV2 and minimising the inclusion of Oars. If this occurs repeatedly (e.g. more than twice in 5 fractions) please contact RTTQA for advice.
- All CBCT exposures including those not resulting in treatment should therefore be recorded on the CRF and plan selection form.
- The patient's hydration may be different on chemotherapy days. Care should be taken when adjusting the drinking protocol on these fractions.

Hafeez S, *et al. BMJ Open* 2020; 10:e041005. doi: 10.1136/bmjopen-2020-041005

- Please review the post-treatment CBCTs to ensure the patient's bladder has not filled beyond the plan selected. If this has occurred, then consider the length of time taken for plan selection and see section 5.3.

5.3 Post treatment

A post treatment cone beam CT should be taken in the first week and once a week thereafter (for all randomised groups, including group 1). It should be reviewed locally to ensure intra fraction filling has been accommodated in plan selection. Please discuss with the PI and contact RTTQA if there are any issues with intra-fraction filling.

5.4 On completion of radiotherapy

On completion of radiotherapy planning, all plans including CT images, structures, plan and dose matrix and plan assessment form (PAF), should be exported, anonymised and sent to the RTTQA or TROG team electronically following the exporting data guidelines in the QA appendix A&B. On completion of a patient's treatment, the plan selection form, first week and then weekly paired (pre and post radiotherapy) CBCT scans and the registration objects (Aria only) should be sent to the RTTQA or TROG team. Investigators should notify the QA team before deleting any relevant data.

Please ensure that all patient data is anonymised prior to sending it to RTTQA/TROG. If you have any difficulties anonymising the data, please contact RTTQA/TROG.

Sending data from Elekta/Mosaiq systems:

Week 1 and weekly pre and post treatment CBCT data should be exported from the XVI using "Option 3". This ensures the CBCT data is sent to the QA team in the correct treatment position.

Sending data from Varian/Aria systems:

Week 1 and weekly pre and post treatment CBCT data should be exported from Aria along with the online registration object. This ensures that the CBCT data can be reviewed in the treatment position by the QA team. Please refer to guidance in Appendix G& H.

6 TREATMENT SCHEDULING AND GAPS

Treatment interuptions during radiotherapy should be avoided as they have detrimental effect on outcome. Treatment must not be interrupted due to staffing issues. Advice should be sought from the RTTQA team and ICR-CTSU in real time if issues with patient scheduling and gaps arise.

If IGRT is unavailable due to unpredicted staff changes, machine breakdown and/or gap day treatment, patients in group 2 and 3 may be treated for up to 5 days using the PTV medium plan without plan selection. Pre-treatment CBCT where possible should still be acquired.. These pre-treatment CBCTs should be sent to RTTQA for review in addition to the CBCTs which are routinely collected.

Hafeez S, et al. BMJ Open 2020; 10:e041005. doi: 10.1136/bmjopen-2020-041005

RAIDER radiotherapy planning and delivery guidelines

In the event of machine service, breakdown, or bank holiday, compensation for the missed fraction should be made. In the first instance the local PI should advise on how this should be made, but it is expected to be achieved by either treating at a weekend or undertaking two fractions a day (ideally on a Friday with a minimum 6 hour gap between treatments). If the treatment machine is unavailable for more than 3 days, please contact the ICR-CTSU and QA team.

Should a treatment break occur due to toxicity, sites are advised to contact ICR-CTSU and/or RTTQA in real time. Compensation is not expected in these circumstance.

All missed fractions are to be reported to the ICR-CTSU and QA team.

Hafeez S, *et al. BMJ Open* 2020; 10:e041005. doi: 10.1136/bmjopen-2020-041005

APPENDIX A:

United Kingdom RAIDER QA Programme

Contact Details:

Contact for scientific queries	Dr Shaista Hafeez<br>Tel: Tel: 0208 661 3274<br>Email: Shaista.Hafeez@icr.ac.uk
Radiotherapy Trials Quality Assurance Team	Amanda Webster RTTQA Group<br>Tel: 0203 826 2320<br>Email:<br>raiderqa.enh-tr@nhs.net

The UK quality assurance programme for RAIDER comprises the following exercises detailed below:

Pre-Trial Quality Assurance

1. Facility questionnaire: This is designed to gauge the IGRT experience of a centre to date. It collects information regarding the type of IGRT used, action thresholds, frequency of interventions and imaging doses. This survey should be accessed online via: http://www.rttrialsqa.org.uk/rttqa/

2. Process document: Details are collected on all aspects of tasks for the complete patient pathway and includes details on all imaging procedures. A process document template can be found at http://www.rttrialsqa.org.uk/rttqa/

3. Outlining Benchmark cases: One case with lipiodol and one case without lipiodol will be provided by the RTTQA to be completed by the P.I for each recruiting site. The targets and organs at risk are to be named and delineated as per the RAIDER radiotherapy planning and delivery guidelines. Those sites that have successfully completed the outlining for another related NIHR trial will not be required to outline the organs at risk, only the target volumes. Please contact the QA team to confirm.

4. Planning Benchmark case: One planning benchmark case provided by RTTQA is to be planned according to the Group 3 randomisation arm.

5. In house IGRT training programme: It is a requirement of RAIDER that sites have an established IGRT training programme already in place before joining the trial. Sites should be utilising cone beam CT for treatment of bladder patients.

6. Bladder 'Plan of the Day' training: Two practice cases with 6 CBCTS each are provided for centres to work through with an accompanying training document. Case 1 includes answers with some step-by-step instructions, for case 2 the answers only are

provided. The cases can be provided on an Elekta or Varian database depending on which system is used by a site. Please contact RTTQA for the training guidance document. Those that have successfully completed the 'Plan of the Day' training for HYBRID do not need to complete this again.

7. RAIDER 'Plan of the Day' training video will be available for streaming on the RAIDER ICR website. Please contact RTTQA or ICR for access

8. RAIDER Plan of the Day assessment: Centres will be given details of two patients with 6 CBCTs each (12 match decisions) to allow individuals to make plan of the day decisions/choices. The match results will be exported to the RTTQA group for review. Those staff members that have completed the 'Plan of the Day' assessment for Hybrid will not be required to complete the assessment. All QA approved individuals will receive a confirmation of their RAIDER accreditation to undertake plan of the day assessments for RAIDER patients.

9. Verification of electronic data transfer: Check DICOM or RTOG data can be suitably anonymised and transferred to and from centres. This includes the planning data and on treatment data, i.e. CBCT and registration objects.

On-Trial Quality Assurance

1. Prospective plan review: The outlining and planning for at least the first adaptive patient and the first dose escalated patient (if the first patient is not dose escalated) will be subject to prospective review by the RTTQA group.

2. Ongoing data collection: All planning data (CT,RS, RD, RP files, PAF) and treatment delivery data (weekly pre and post treatment CBCT and registration objects (if available) will be collected by the RTTQA group for retrospective review

3. IGRT/POD selection support: The first adaptive patient randomised by all sites will be subject to a retrospective review. If centres have any difficulties in the plan selection, please do not hesitate to contact RTTQA.

Data Export

Please contact raiderqa.enh-tr@nhs.net for information on data transfer.

Please ensure that data is appropriately labelled when sent to RTTQA, i.e. trial name, randomisation number and type of data (e.g. planning/CBCTs).

RAIDER radiotherapy planning and delivery guidelines

For all patients please ensure the following data is sent for all RAIDER patients:

Table 7:

**RAIDER Data Checklist**	**Sent**
Diagnostic Information (e.g. report, screenshot of imaging)	
ALL planning CTs (e.g. include CT_30 and CT_60 for adaptive patients)	
Structure set, Dose Cube, Plan	
Plan Assessment Form	
Plan Selection Form (For all trial patients)	
Week 1 (#1-5) and weekly pre and post CBCTs	

Hafeez S, *et al. BMJ Open* 2020; 10:e041005. doi: 10.1136/bmjopen-2020-041005

APPENDIX B:

Trans Tasman Radiation Oncology Group: Australia & NZ

RAIDER Trial QA programme

Contact Details:

Primary ANZ sponsor contact details	Trans Tasman Radiation Oncology Group
Contact for public queries	Patrick Wheeler<br>+61 2 4014 3903<br>RAIDER@trog.com.au
Contact for scientific queries	Prof Farshad Foroudi<br>+61 3 9496 9797<br>farshad.foroudi@austin.org.au
Radiotherapy Quality Assurance Team	TROG<br>Email: qa@trog.com.au

Pre-Trial QA

1. Facility questionnaire: This is designed to gauge the IGRT experience of a centre to date. It collects information regarding the type of IGRT used, action thresholds, frequency of interventions and imaging doses. For simpler completion at centres, TROG have created two sections, one for Radiation Therapists and another section for Radiation Oncology Medical Physicists, which may be completed separately. This questionnaire can be accessed online at: www.trog.com.au. In addition, sites wishing to use inversely planned techniques must be credentialed to do so. Please contact qa@trog.com.au for further information.

2. Process document: Details are collected on all aspects of tasks for the complete patient pathway and includes details on all imaging procedures. A process document template can be found at http://www.trog.com.au/Trials-and-research-projects, RAIDER, Quality Assurance. TROG website password required.

3. Fiducial Marker Quality Assurance Document: To be completed by sites who have indicated that they intend to use fiducial markers. This document can be found at http://www.trog.com.au/Trials-and-research-projects, RAIDER, Quality Assurance. TROG website password required.

4. Outlining Benchmark cases: The P.I. from each recruiting site should complete the two outlining benchmarking cases available on the TROG website. One male (with lipiodol and one female (without lipiodol). The targets and organs at risk are to be named and delineated as per the RAIDER Radiotherapy Planning, Delivery and QA Guidelines.

   Male Case 1: Download TROG 14.02 BMKXX1 from http://www.trog.com.au/Trials-and-research-projects, RAIDER, Quality Assurance. TROG website password required.

Female Case 2: Download TROG 14.02 BMKXX2 from http://www.trog.com.au/Trials-and-research-projects, RAIDER, Quality Assurance. TROG website password required.

Once the outlining cases have been completed by the local P.I. an export of the CT images and structure sets in DICOM should be returned to the TROG QA team. All data must be transferred via Central Quality Management System (CQMS). CQMS can be accessed through https://cqms.trog.com.au/login.jsp. If you require a CQMS account, please contact qa@trog.com.au

5. Planning Benchmark case: One planning benchmark is to be planned according to the Group 3 randomisation arm of the trial.

   Download TROG 14.02 BMKXX3 from http://www.trog.com.au/Trials-and-research-projects, RAIDER, Quality Assurance. TROG website password required.

   Once the planning benchmark case (with three plans) has been created (with appropriate review and acceptance by the local P.I.) the export of the CT images, dose matrices, RT plan and structure sets in DICOM should be returned to the TROG QA team. All data must be transferred via the Central Quality Management System (CQMS). CQMS can be accessed through https://cqms.trog.com.au/login.jsp. If you require a CQMS account, please contact qa@trog.com.au.

6. In house IGRT training programme: It is a requirement of the RAIDER trial that sites have an established IGRT training programme already in place before joining the trial. Sites should be utilising cone beam CT for treatment of bladder patients. Successful participation in the TROG 10.01 BOLART trial including the e-learning will be regarded as satisfying these criteria. IGRT training programme details are requested as part of the Facility Questionnaire.

7. IGRT Credentialing:
   a. Bladder 'Plan of the Day' training: Two practice cases with six CBCTS each are provided for centres to work through with an accompanying training document. Case 1 includes answers with some step-by-step instructions, for case 2 the answers only are provided. The cases are provided in DICOM format via the TROG website. Sites must import the two cases with accompanying CBCTS into appropriate image matching/registration software. The training guidance document is accessible via the TROG website.

   b. Bladder 'Plan of the Day' training video: Available on the TROG website: www.trog.com.au

   c. RAIDER Plan of the Day assessment: Two patients with six CBCTs each (twelve match decisions) will be provided to sites to allow individuals to make plan of the day decisions/choices. Staff must record their match results using the Plan of the Day Assessment Form. Assessment forms must be submitted to qa@trog.com.au.

8. Dosimetry Audit: All sites who have participated in the TROG 10.01 BOLART trial will not be required to complete a dosimetry phantom study. For sites who have not participated

Hafeez S, *et al. BMJ Open* 2020; 10:e041005. doi: 10.1136/bmjopen-2020-041005

in TROG 10.01 BOLART please contact the TROG QA office at qa@trog.com for further information.

On-Trial QA

Please contact TROG QA for advice as required for the first patient planning and treatment. Teleconference facilities may be made available if required.

1. Quality assurance Pre-treatment Plan Review: Pre-treatment review of at least the **first** adaptive patient and the **first** dose escalated patient (if the first patient is not dose escalated) recruited from each site will be conducted by TROG. The **first** participant with fiducial marker placement from each must also be submitted for review.

   The treatment plan will be required for review at least one week prior to treatment commencement. If the review results are acceptable the participant will proceed to treatment.

   The following **four** adaptive patients from each site will undergo timely review (to be completed during the first week of treatment).

   A checklist of the source data required for each RT QA case will be provided by the TROG QA Office with specifications of timelines for data submission included. See below for instructions for the upload of source data required for real time pre-treatment radiotherapy QA.

2. Quality assurance Post-treatment Plan Review: Adaptive patients will be sampled for QA review at a rate of **one in five** for post-treatment review following the initial pre-treatment/timely sampling.

   Patients in the standard whole bladder radiotherapy arm will be sampled for QA review at a rate of **one in five** for post-treatment review.

   All participants will be required to submit data at the end of treatment. This data should be submitted via CQMS. To assist with this process a checklist of the source data required will be provided to you by TROG QA. Timelines for data submission will also be specified.

3. IGRT/POD selection support: The first adaptive patient randomised by all sites will be subject to a retrospective review. If centres have any difficulties in the plan selection, please do not hesitate to contact the TROG QA team.

Data Export and Upload

Australia and New Zealand sites uploading data to TROG CQMS are not required to anonymise data prior to upload. All DICOM files are automatically de-identified at the point of upload to CQMS.

Radiation Therapy Treatment Plan
An electronic export of the radiotherapy treatment planning data file from the treatment planning system is required in **DICOM-RT format.** Please submit the RT Treatment Plan Export

[CT files, structure set, RT plan and dose matrix for each PTV] using the **[RT Plan Upload]** function listed in CQMS. Exported files must be uploaded as a single zipped file into CQMS.

- Please include ALL planning CTs (e.g. include CT_30 and CT_60 for adaptive patients)

<u>CBCT Data Upload</u>
The following files are also to be uploaded using the **[RT Plan Upload]** function listed in CQMS for final end of RT QA review:

- All pre and post treatment CBCT (exported in DICOM format). Each CBCT dataset should be uploaded as a single zipped file. As a minimum for all groups, this should include week 1 and weekly post-treatment CBCTs.
- Registration objects
- Please refer to Appendix G, H and I for additional assistance when exporting CBCT datasets from ARIA, MOSAIQ and Raystation

<u>Supplementary Information Data Upload</u>
The following files are to be uploaded using the **[Other Upload]** function listed in CQMS for final end of RT QA review:

- Treatment prescription (including total dose, number of fractions, dose per fraction, prescription isodose)
- Treatment plan summary (including field information and beam parameters)
- Plan of the day decisions (Plan Selection Form)
- Verification images demonstrating correct export of the radiotherapy plan (JPEG of the DVH and isodose distribution).
- Daily Dose Record (including dates of treatment delivery)
- Imaging Log (verification of imaging performed)

Hafeez S, *et al. BMJ Open* 2020; 10:e041005. doi: 10.1136/bmjopen-2020-041005

APPENDIX C:

QA Benchmark Cases: Outlining and Planning

PRE-TRIAL OUTLINING BENCHMARK CASE

All centres wishing to participate in the RAIDER trial will need to complete the following contouring exercise. DICOM CT data sets can be downloaded from the RTTQA website (www.rttrialsqa.org.uk) or TROG website (www.trog.com.au). See QA Appendix A& B for more information.

Outlining Benchmark Case 1: T2N0M0 Male Pelvis

History: 72 year old male, presented with haematuria. Proceeded with TURBT and 3 cycles neo-adjuvant chemotherapy (cisplatin-gemcitabine). Post treatment cystoscopy shows pathological complete response (pCR). Patient is planned for chemo-radiotherapy to bladder.

Biopsy: biopsy at diagnosis consistent with pT2a G3 TCC with no associated distant CIS

Initial staging diagnostic information: Information available for contouring/GTV delineation includes:

- bladder map/cystoscopy showing tumour present left lateral wall around left ureteric orifice
- CT baseline (pre-chemotherapy)
- MRI baseline (pre-chemotherapy) and lipiodol injected at tumour / scar (post chemotherapy)

Radiotherapy contouring/planning (according to group 2 and 3): Planning scan at 30 minutes and 60 minutes reflect no filling (<50cc)**.**

Contouring Instructions:

Please import Case 1 into your TPS.
Please outline the following volumes:

- GTV_30
- CTV_30
- CTV_60
- RECTUM
- OTHER_BOWEL
- FEMORALJOINT_L or FemJoint_L
- FEMORALJOINT_R or Femjoint_R

Please create the following PTV volumes using the expansion information contained in Table 1 and Table 2:

- PTV_Sm
- PTV_Med
- PTV_Lar_30
- PTV2_Sm
- PTV2_Med

RAIDER radiotherapy planning and delivery guidelines

- PTV2_Lar_30

NB: Please refer to the additional diagnostic information available for this patient.

Additional Diagnostic Information for Outlining Benchmark Case 1

Letter from surgeon identifying clinical position of tumour at cystoscopy (Bladder map not available):

Diagnoses:

1. **G3 pT2 at least TCC bladder.**
2. **Exercise induced asthma.**

I would be grateful if you could review this pleasant retired nurse urgently. I performed his TURBT on the 19th June and he had a large bladder tumour on the left lateral wall with a solid base. It was invading anteriorly into the prostate and bladder neck. Post-operative EUA revealed a mobile mass and a firm prostate. The histology has come as G3 pT2 tcc bladder.

He initially failed a trial without catheter and is having his catheter removed again today. He is otherwise fit and well.

Yours sincerely

Dictated and approved by Consultant

Post-chemotherapy Cystoscopy

Cystoscopy, bladder biopsy and lipiodol injection

Lithotomy, WHO checklist, pressure points protected, gentamicin.

Bimanual no pelvic mass, smooth prostate

Excellent views with 21 Fr scope and 12° lens. No visible tumour in bladder. Scar above left ureteric orifice - biopsied and lipiodol injected submucosally. New red patch at dome seen– region biopsied and lipiodol injected. Additional three random biopsies taken.

Histopathology:

Fibrosis only at all biopsied sites. Therefore red patch at bladder dome although injected with lipiodol should not to be included in GTV volume)

Outlining Benchmark Case 2: T2N0M0 Female Pelvis

History: 66 year old female presented to urologist with isolated episode of frank haematuria. Proceeded to TURBT. She started neo-adjuvant chemotherapy (cisplatin-gemcitabine) but suffered deterioration in hearing so went on to have chemo-radiotherapy to bladder after 1 cycle.

Biopsy: G3 pT2 TCC bladder with focal adjacent CIS

Initial staging diagnostic information: Information available for contouring/GTV delineation bladder map/cystoscopy tumour present left posterior wall ureteric orifice, CT baseline (pre-chemotherapy), MRI baseline (pre-chemotherapy, post TURBT) - non-contributory.

Radiotherapy contouring/planning (according to group 2 and 3): Planning scan at 30 min and 60 min reflect filling (>50cc)**.**

Contouring Instructions:

Please import Case 2 into your TPS.

Please outline the following volumes in accordance with instructions

- GTV_30
- GTV_60
- CTV_30
- CTV_60
- RECTUM
- OTHER_BOWEL
- FEMORALJOINT_L or FemJoint_L
- FEMORALJOINT_R or FemJoint_R

Please create the following PTV volumes using the expansion information contained in Table 1 and Table 2:

- PTV_Sm
- PTV_Med
- PTV_Lar_60
- PTV2_Sm
- PTV2_Med
- PTV2_Lar_60

Data Export

Once the outlining benchmark cases have been created, reviewed and accepted by the local PI, the export of the CT images and structure sets in DICOM should be returned to the appropriate QA team. See QA appendix A & B for information on data export.

PRE-TRIAL PLANNING BENCHMARK CASE

All trial centres must complete and submit the RAIDER pre-trial planning benchmark case. The CT DICOM data and pre-outlined structure set is available for download from www.rttrialsqa.org.uk (UK) or www.trog.com.au (Australia/NZ). Details for the planning cases are given in the next section.

Benchmark Planning Case: T2N0M0 TCC male pelvis

History: 63 year old male presented with frank haematuria, proceeded to TURBT; unsuitable for neo-adjuvant chemotherapy in view of co-morbidities, received chemo-radiotherapy to bladder

Biopsy: G3 pT2a TCC bladder

Initial staging diagnostic information: Information available for contouring/GTV delineation includes:

- Radiotherapy Bladder map/cystoscopy tumour present right bladder wall
- CT baseline,
- MRI baseline (post TURBT non-contributory).

Radiotherapy contouring/planning (according to group 3): Planning scan at 30 min and 60 min; filling (>50cc)**.** PTV small/medium contours on 30minute scan; PTV Large created on 60minute scan

Planning Exercise:

Please import the CT and structure sets into your own TPS system. Following the RAIDER trial protocol and radiotherapy planning and delivery guidelines please produce a series of three radiotherapy plans as the patient is randomised to the dose escalated adaptive tumour boost DART (group 3)

Structures:

The CT has been delineated by a trial clinician with the following structures:

Structure	Volume (cc)
GTV_30	10.0
GTV_60	12.3
CTV_30	153.4
CTV_60	272.2
PTV_Sm_	273.2
PTV_Med_	385.3
PTV_Lar_60	577.0
PTV2_Sm	40.5
PTV2_Med	76.1
PTV2_Lar_60	82.3
RECTUM	65.4
OTHER_BOWEL	314.5
FEMORALJOINT_L	63.1

BMJ Publishing Group Limited (BMJ) disclaims all liability and responsibility arising from any reliance placed on this supplemental material which has been supplied by the author(s)

FEMORALJOINT_R	62.1

Please DO NOT edit any of these structures. The names and volumes on which the structures appear on have been given so that you can check that the structures have been imported properly.

Data Export

Once the planning benchmark case (three plans) has been created and reviewed and accepted by the local PI, the export of the Plan Assessment Form (PAF), CT images, dose matrices, RTplan and structure sets in DICOM should be returned to the RTTQA team (UK) or TROG team (Australia/NZ). For information on data export see QA appendix A & B.

Hafeez S, *et al*. *BMJ Open* 2020; 10:e041005. doi: 10.1136/bmjopen-2020-041005

APPENDIX D

Quick Contouring Checklist Adaptive Arms

1) Outline CTV_30 on CT30. Check whether appropriate for urethra to be included.
2) Copy and paste CTV_30 onto CT60. Rename CTV_60. Edit this volume to take into consideration bladder filling taking care not to make the volume smaller inferiorly.
3) Check CTV_30 does not expand beyond CTV_60. Use Boolean functions can be used to ensure CTV_60 always encompasses CTV_30.
4) Check volume difference between CTV_30 and CTV_60. If less than 50cc, expand all PTV volumes from target volumes on CT30, if greater than 50cc create large PTVs from CT60 target volumes.
5) Outline GTV_30 (and GTV_60 if applicable)
6) Ensure GTV shares the same bladder wall as CTV by either manual editing and/or use of Boolean operators.
7) Create adaptive PTVs from CTV using the relevant expansions
8) Create adaptive PTV2s from GTV using the relevant expansions
9) Check PTV_Lar encompasses PTV_Med and PTV_Sml
10) All OARs are always outlined on CT30 (FEMORALJOINT_L, FEMORALJOINT_R, RECTUM and OTHER_BOWEL. Check OTHER_BOWEL volume is at least 2.0cm superior to PTV _Lar

Hafeez S, *et al. BMJ Open* 2020; 10:e041005. doi: 10.1136/bmjopen-2020-041005

RAIDER radiotherapy planning and delivery guidelines

APPENDIX E

Proposed patient intervention to be considered in circumstances where plan selection is not felt to be optimal.

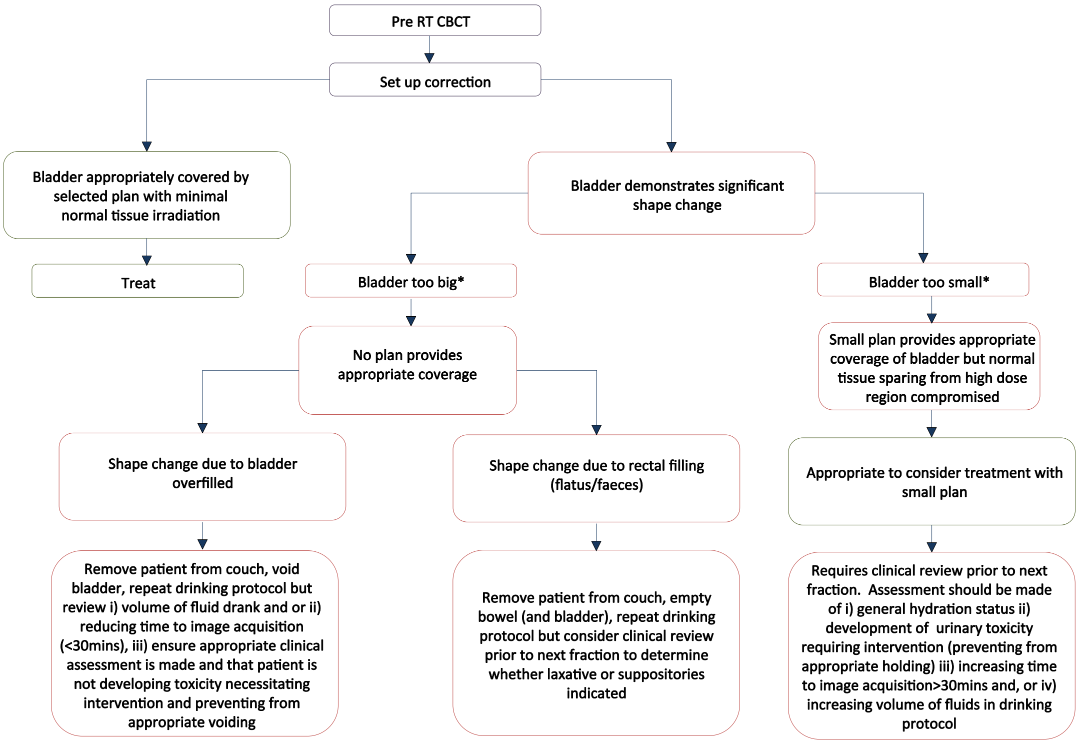

*If no plan provides appropriate coverage (bladder persistently too big or too small) more than twice in 5 fractions despite intervention, it is advised that the RTQA team is contacted for advice as in rare circumstances replan maybe indicated.
If the bladder is too small sites can consider treating with the small plan OR removing the patient from the treatment couch to allow for more filling.
**When considering changing the drinking protocol, only consider 1 timing or drinking change at a time. This will allow centres to establish which intervention has an effect on the patient's bladder.

Hafeez S, et al. BMJ Open 2020; 10:e041005. doi: 10.1136/bmjopen-2020-041005

RAIDER radiotherapy planning and delivery guidelines

APPENDIX F

RAIDER training cases and examples

1. Acceptable for treatment with chosen plan

Reference image

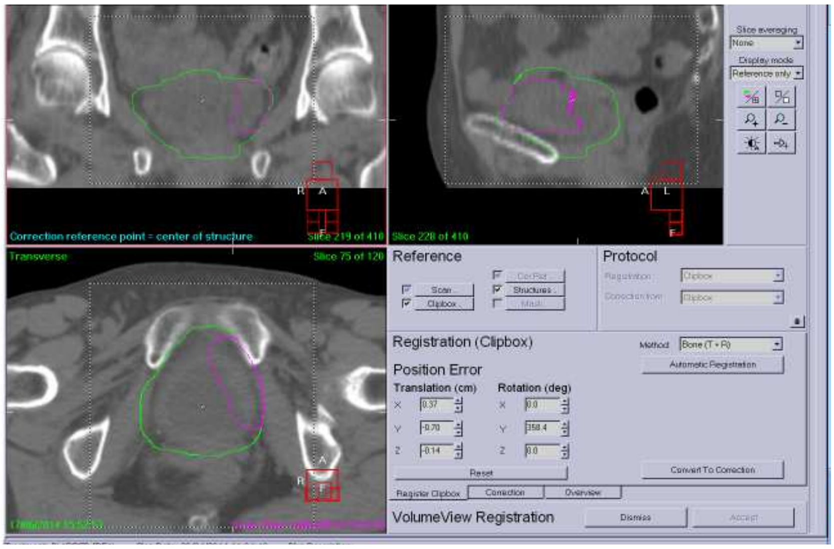

Localisation image

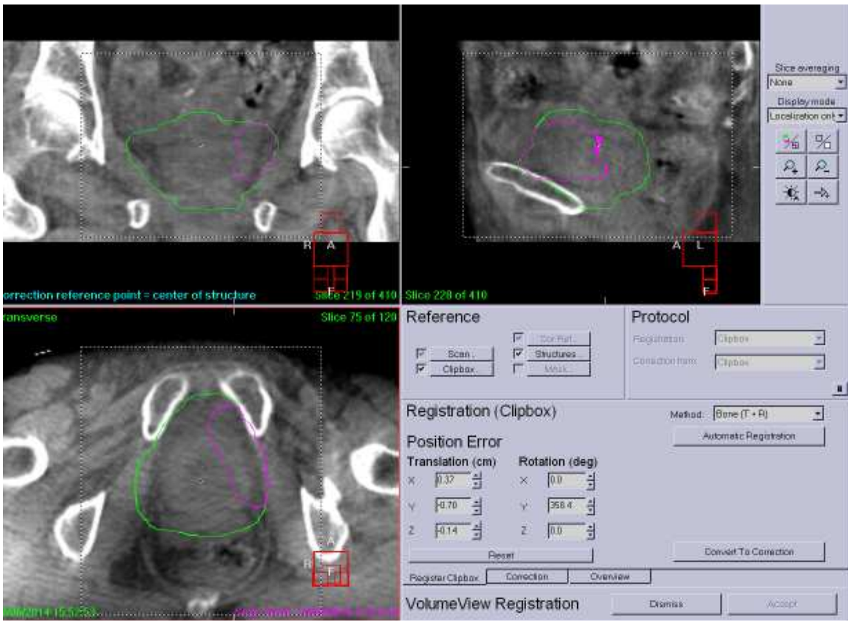

Hafeez S, *et al. BMJ Open* 2020; 10:e041005. doi: 10.1136/bmjopen-2020-041005

RAIDER radiotherapy planning and delivery guidelines

2. Bladder too small.

Bladder appears significantly under filled compared reference image, most apparent on sagittal localisation view. Note boost dose includes large proportion of bladder.

Acceptable for treatment with chosen plan as no compromise to bladder coverage. Patient review is recommended prior to next fraction to assess drinking protocol and, or time to image acquisition in order to optimise normal tissue sparing.

Reference image

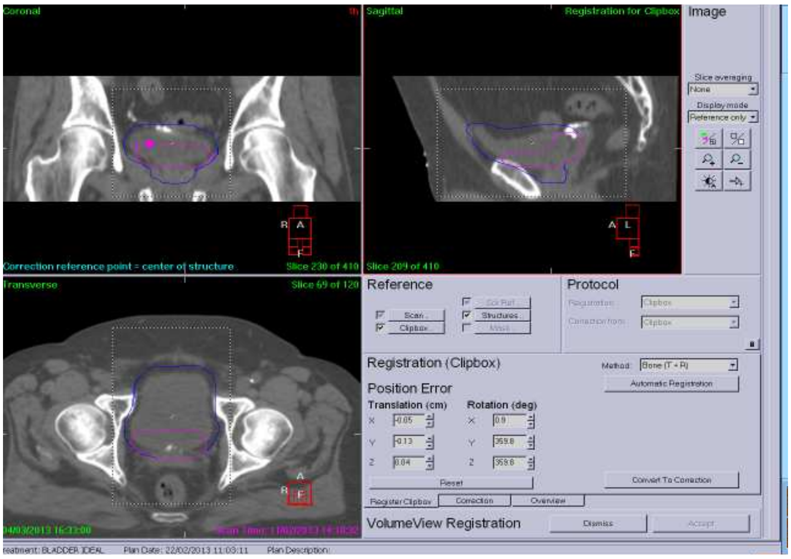

Localisation image

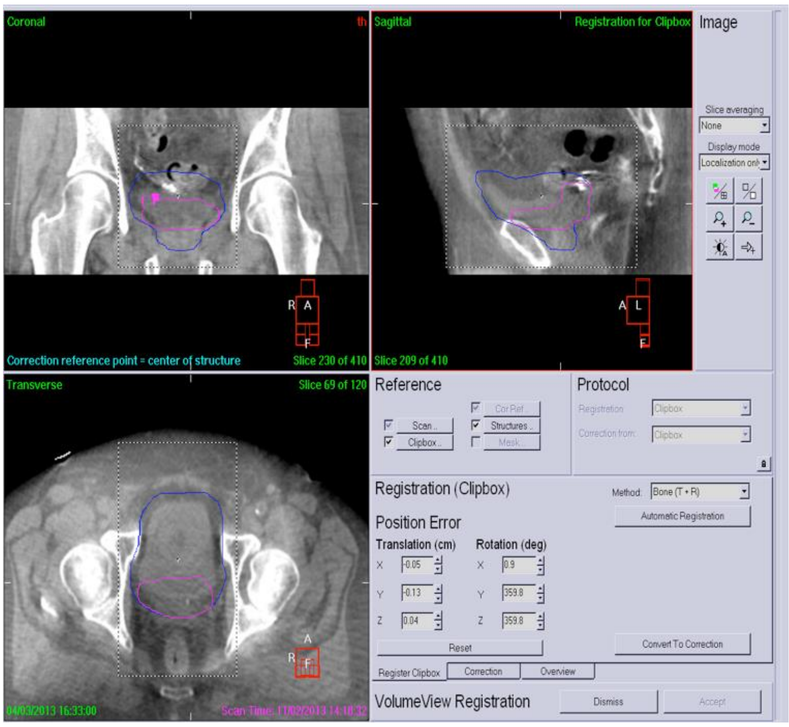

Hafeez S, *et al. BMJ Open* 2020; 10:e041005. doi: 10.1136/bmjopen-2020-041005

RAIDER radiotherapy planning and delivery guidelines

3. Bladder too large

No plan selection is acceptable for treatment (without compromise to bladder coverage).

Patient to empty bladder and repeat set-up with review of drinking protocol/time to CBCT acquisition.

Reference image

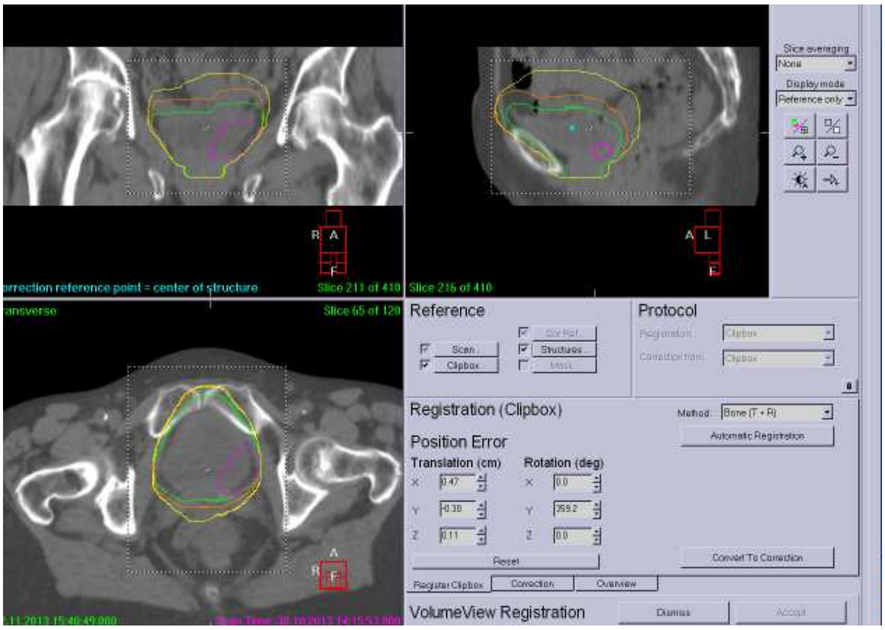

Localisation image with no structures

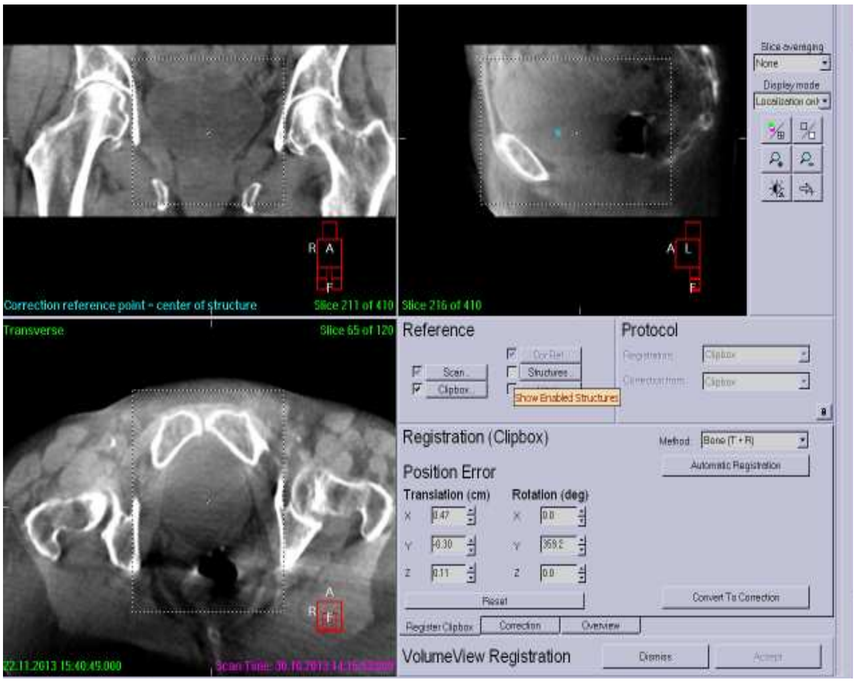

RAIDER radiotherapy planning and delivery guidelines

Reference image

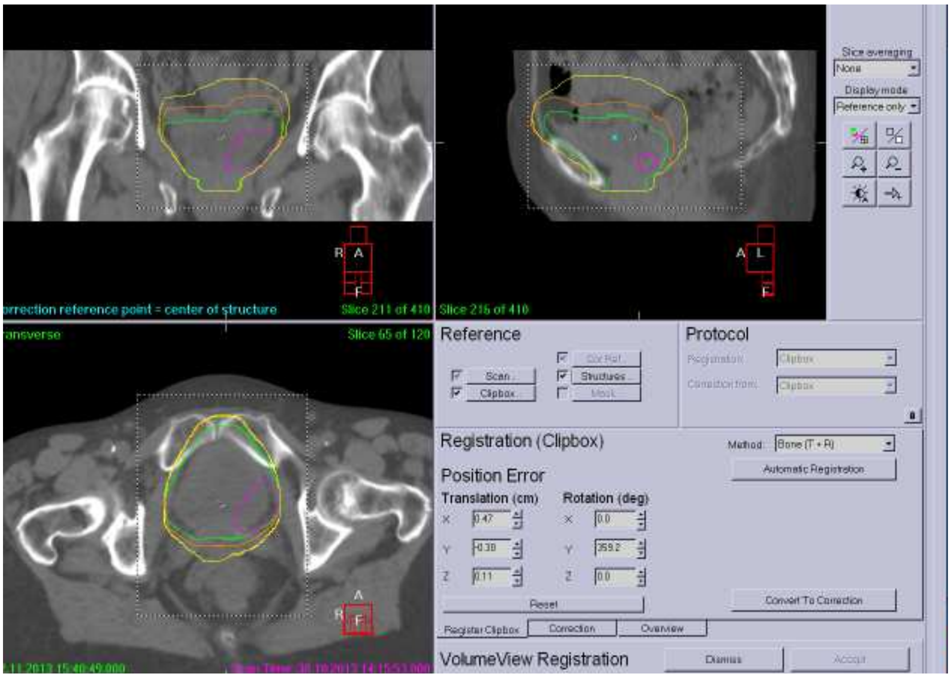

Localisation image with structures

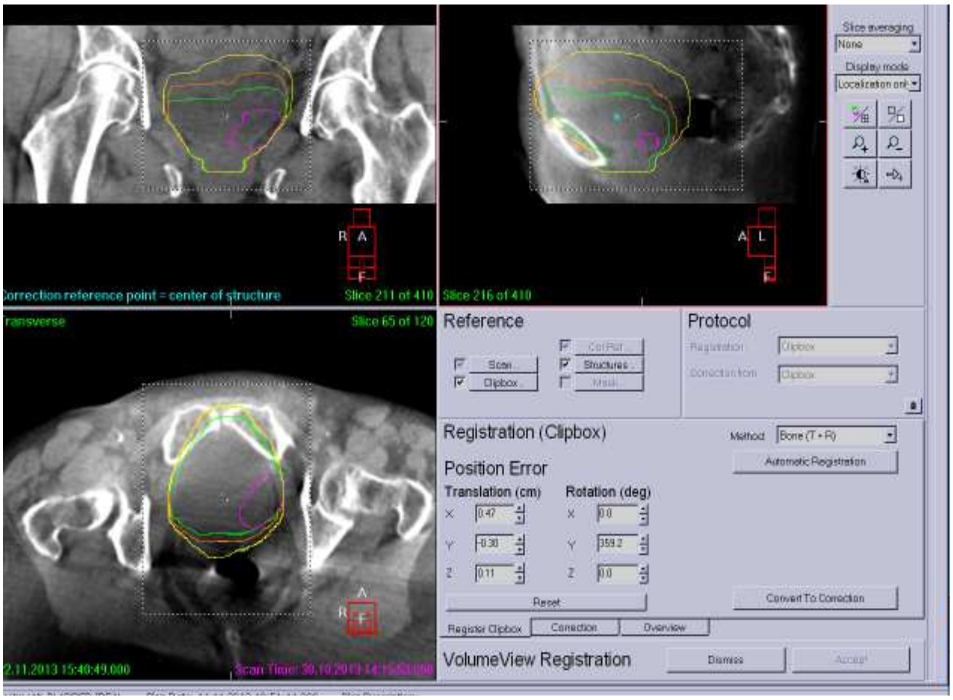

RAIDER radiotherapy planning and delivery guidelines

4. Fiducial marker artefact on CBCT interpretation

Image below demonstrates effect of lipiodol spill outside bladder resulting in significant artefact and degradation of image. Recommendation is that each CBCT should be closely reviewed with reference image and delineated bladder volume.

Reference image

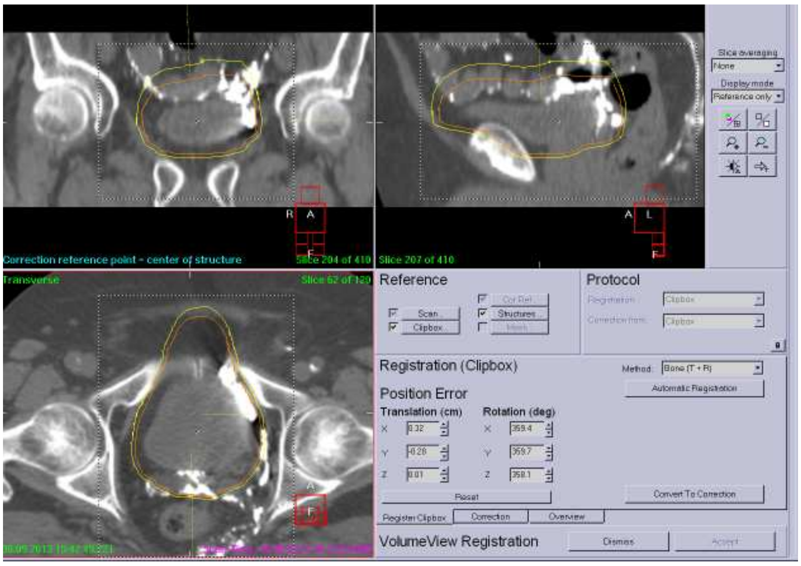

Localisation image

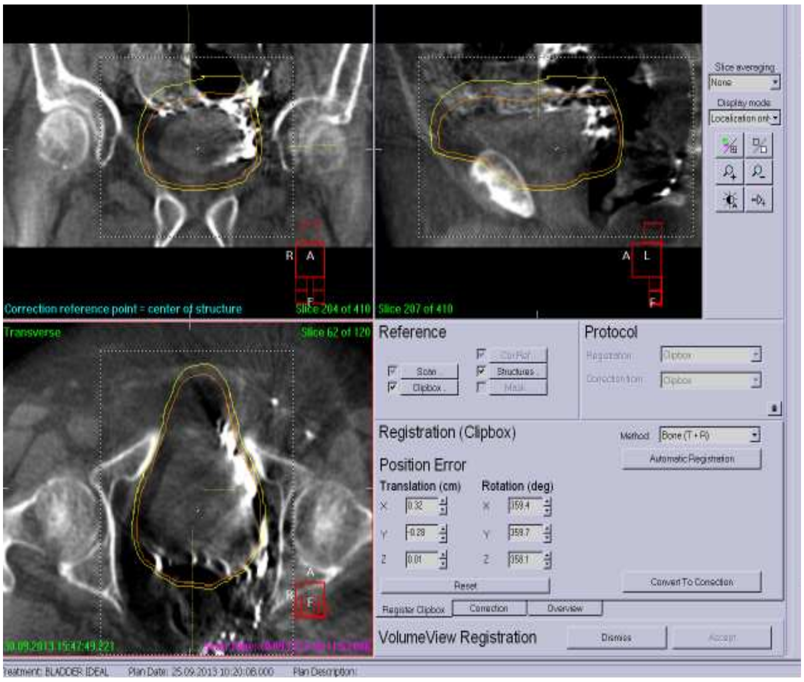

RAIDER radiotherapy planning and delivery guidelines

APPENDIX G

Quick Reference Guide Exporting CBCTs from Aria

These steps are for guidance only. Please contact your QA group if you have difficulties exporting data.

1) Load patient in Aria, go to Offline review and select one (any is ok) of the CBCTs to load
2) Select 'Session Timeline' tab at the bottom of the screen
3) Right click on the CBCT to export and select 'Export to DICOM' >> 'To 'DICOM Export to Pinnacle/External Anonymisation Software/Folder''
4) The Import Export window loads. Select the 'Show/Hide Tree' button to bring up a list of all CBCTs and registration objects
5) Select the radio buttons next to the CBCTs to export, using the date to identify them.
6) Click on the + to expand the folder called 'Registrations':

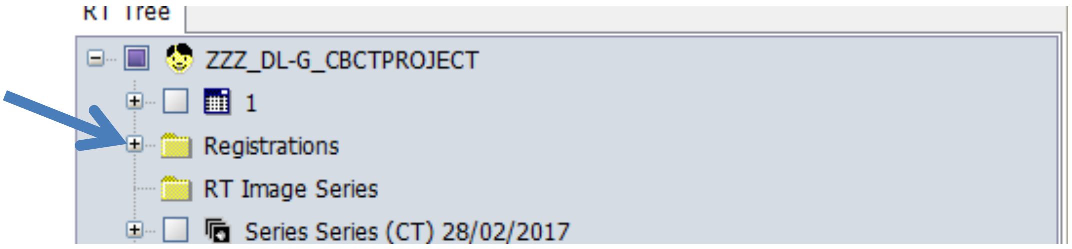

7) Select the radio buttons of the registration objects that have the same date as the CBCTs you wish to export.

8) Click the right arrow to export to the export folder/destination.

9) In windows explorer navigate to the export folder/destination and select the CBCTs and registration object files. Copy them to the DICOM anonymiser folder and run through your anonymiser to anonymise as per standard departmental working instructions. Please check the anonymization software does not remove the registration objects. If this occurs please contact RTTQA.

10) Save in a folder ready for export to RTTQA.

Please note that, for ease, whole patient exports can be submitted by the centre to RTTQA

For further information on suitable anonymisation software for ARIA exports please contact raiderqa.enh-tr@nhs.net

Hafeez S, et al. BMJ Open 2020; 10:e041005. doi: 10.1136/bmjopen-2020-041005

APPENDIX H

Quick Reference Guide Exporting CBCTs from Elekta/XVI

These steps are for guidance only. Please contact your QA group if you have difficulties exporting data.

Exporting from XVI

Images need to be exported individually, not as a treatment.

1. On the XVI acquisition PC select the image to be exported
2. Select IMAGE from the tool bar
    2.1. EXPORT
    2.2. DICOM SERVER 'select TPS/online server'
    2.3. OK
3. The next screen gives 3 options
    - Option 1 – In the Option 1 list, select a multiple of the voxel size in the reconstructed volume for the CT slice thickness. This can be done without a reference dataset being available and hence imports the CBCT into pinnacle without any co-ordinates related to the reference image. This is not likely to be useful.
    - Option 2 – Only available if image registration was done and approved for this reference image. The position of the VolumeView™ exported is the position **before** registration.
    - Option 3 – Only available if image registration was done and approved for this reference image. The position of the VolumeView™ exported is the position **after** registration.

3.1 Select Option 3 as the information required is as the patient was treated i.e. after registration (e.g. if patient was treated with correction).

NB Registration has to be performed for option 3 to be available.

3.2. In the Export options area, click the Create CT button.

3.3 EXPORT

APPENDIX I

Exporting CBCTs from Raystation

For centres exporting CBCTs from Raystation please contact raiderqa.enh-tr@nhs.net

This is a controlled document and should not be copied, distributed or reproduced without the written permission of the ICR-CTSU

Hafeez S, *et al*. *BMJ Open* 2020; 10:e041005. doi: 10.1136/bmjopen-2020-041005
