# Supplementary material for: Protocol for tumour-focused dose-escalated adaptive radiotherapy for the radical treatment of bladder cancer in a multicentre phase II randomised controlled trial (RAIDER): radiotherapy planning and delivery guidance
Source: BMJ Open. 2020 Dec 31;10(12):e041005. doi: 10.1136/bmjopen-2020-041005 (PMC7780718; doi:10.1136/bmjopen-2020-041005)
Supplement: Supplementary data [file bmjopen-2020-041005supp003.pdf]

Please print on hospital's headed paper

RAIDER

A **R**andomised phase II trial of **A**daptive **I**mage guided standard or **D**ose **E**scalated tumour boost **R**adiotherapy in the treatment of transitional cell carcinoma of the bladder

PATIENT INFORMATION SHEET

We would like to invite you to take part in a research study called RAIDER.

Before you decide whether to take part, it is important that you understand why the research is being done and what it would involve for you. One of your doctors or nurses will go through this information sheet with you and answer any questions you may have. Please take time to read the information carefully and to discuss it with relatives, friends and your GP if you wish. Please ask if anything is unclear or you need any further information.

Thank you for reading this and considering taking part in our research.

Why am I being invited to take part?

We are inviting you to join this study because your doctor has found cancer that has grown into the wall of your bladder (muscle invasive bladder cancer) and you are interested in receiving radiotherapy treatment which will be given in small doses every day for 7 weeks.

What is radiotherapy treatment?

Radiotherapy uses targeted beams of high strength x-rays to kill cancer cells. Because radiotherapy can also cause damage to non-cancer cells, the treatment is carefully planned by doctors and physicists so that only your bladder and a small border surrounding it is treated with the highest radiotherapy dose.

Radiotherapy is individually designed for each patient, based on a CT scan taken a few weeks before treatment. This CT scan tells your doctor about the position and shape of your bladder.

The bladder can move within the body depending on how full it is and because of where it is in relation to the bowel. It is important that the radiotherapy does not miss any of the bladder tumour because of this movement, so a safety margin is added around the bladder on the radiotherapy treatment plan.

CANCER RESEARCH UK

Hafeez S, *et al. BMJ Open* 2020; 10:e041005. doi: 10.1136/bmjopen-2020-041005

Each patient would usually have one radiotherapy treatment plan designed for them so that the radiotherapy is targeted at their tumour.

When radiotherapy treatment is given, the patient lies still on a bed whilst the radiotherapy machine moves around to send the radiotherapy beams from different directions. These beams all focus on where the bladder is, to make sure the whole bladder receives the highest radiotherapy dose possible.

What is adaptive radiotherapy treatment?
We are now able to take a scan of where the bladder is when a patient is lying on the radiotherapy bed before each treatment. This means we can target the bladder more precisely.

In this study we are looking at whether it is possible to design three treatment plans (small, medium and large) and then choose the one that best fits the size of the bladder on the patient's treatment day. This is called 'adaptive radiotherapy'. Adaptive radiotherapy may allow treatment to be given with smaller safety margins, which may lead to fewer side effects.

What is tumour focused radiotherapy treatment?
It is also possible to focus the highest dose of radiotherapy on the bladder tumour. This means that the rest of the bladder can be given a lower dose of radiotherapy. This may mean fewer side effects as the rest of the bladder will be given less radiotherapy. It also allows us to find out whether a higher dose of radiotherapy can be given only to the tumour (tumour boost), to see if this will reduce the chance of the cancer returning, whilst also keeping any side effects as mild as possible.

What is the purpose of RAIDER?
RAIDER is based on a study of adaptive tumour boost radiotherapy which was conducted at one UK hospital. We hope to show that this complex radiotherapy can be given by radiotherapy departments at different hospitals. If this is possible, we want to look at whether the side effects are similar to those experienced by people receiving standard radiotherapy.

RAIDER is based on several smaller studies conducted at hospitals worldwide, which suggest that treatment using these radiotherapy techniques could help to reduce side effects. Although these smaller studies are promising, RAIDER is the largest study of these techniques and has been designed to give us as much information as possible. We will use the results of RAIDER to develop a future study to investigate whether these techniques could improve how well radiotherapy cures bladder cancer.

What would happen if I took part?
At least 240 people in the UK, Australia and New Zealand will be included in RAIDER. All RAIDER participants will be treated with daily radiotherapy.

Hafeez S, *et al. BMJ Open* 2020; 10:e041005. doi: 10.1136/bmjopen-2020-041005

Everyone who agrees to take part in this research study will be in one of three groups:

1. Group 1: Standard radiotherapy: One out of four people taking part will be given standard bladder radiotherapy using the same radiotherapy treatment plan each time, treating their whole bladder to the same dose of radiotherapy.

2. Group 2: Adaptive tumour focused radiotherapy: One out of four participants will be given adaptive tumour focused radiotherapy, using the radiotherapy treatment plan which fits the size of their bladder the best. The standard dose of radiotherapy will be targeted at the tumour, with the rest of the bladder receiving a lower dose than normal.

3. Group 3: Adaptive tumour boost radiotherapy: Two out of four participants will have adaptive tumour boost radiotherapy, using the radiotherapy treatment plan which fits the size of their bladder the best. A higher than standard dose of radiotherapy will be targeted at the tumour, with the rest of the bladder receiving a lower dose than normal.

The only way to make sure that the people in the three groups are as similar as possible is to have the treatment decided upon by chance: a process called randomisation. This process ensures that the treatments are compared fully and fairly.

If you agree to take part, your doctor or nurse will ring the research centre. The centre will then record your details and tell your specialist your treatment, which will be selected by chance. This means you could have any of the three treatments described above. Whichever group you are in, you will be treated with the best possible care and will be monitored closely.

What do I have to do before my radiotherapy treatment?

To make sure your treatment is as effective as possible, it has to be carefully planned by your doctor and other specialised staff (radiographers and physicists).

To help with this, your doctor may wish you to have small markers (fiducial markers) placed in your bladder. If this applies to you, you will be given the bladder tumour marker information leaflet to read.

You will need to visit the hospital for a planning CT scan before you start radiotherapy. The radiographers will also take measurements that are needed for the treatment plan and will make small permanent marks which help to line up the radiation beam. Radiotherapy is a very precise treatment and it is important that you are able to lie in exactly the same position for every treatment.

Hafeez S, *et al. BMJ Open* 2020; 10:e041005. doi: 10.1136/bmjopen-2020-041005

Group 1 – standard radiotherapy planning visit:
You will have a planning CT scan taken with your bladder empty. All of the planning procedures for the standard group are part of the routine care for patients receiving bladder radiotherapy, so you would have them even if you choose not to take part in the RAIDER research study. The planning session at the radiotherapy department usually takes place once and lasts about 30 minutes.

Group 2 and 3 – adaptive tumour focused radiotherapy planning visit:
If you are in group 2 or 3, we will ask you to empty your bladder and then to drink 350ml of water (just over ½ pint). We will take the first scan at 30 minutes and the next at 60 minutes after drinking, as your bladder fills. If you decide to join the study you will be given a leaflet describing drinking guidelines. The planning session lasts about 70 minutes in total, but each scan will only take a few minutes.

What do I have to do during my radiotherapy treatment?
Your treatment will be given daily. If you are in group one we will ask you to empty your bladder immediately before each treatment. If you are in group 2 or 3, we will ask you to empty your bladder and then drink 350mls of water 30 minutes before your planned radiotherapy treatment.

For all groups, once the radiographer has helped you to get into position and made sure that you are comfortable, we may take a scan in the treatment room. This will take about 2 minutes.

For patients in group one receiving standard radiotherapy, this scan will be used to make sure the bladder is in the area which will receive the highest dose of radiotherapy.

If you are receiving adaptive tumour focused radiotherapy we will use the information from the pre-treatment scan to choose the best radiotherapy treatment plan to fit your bladder size and the position of your tumour. This will take around five to ten minutes. The plan will be selected by a specially trained doctor or radiographer and checked by a second trained person before you receive your radiotherapy treatment.

You will need to lie still for up to 20 minutes whilst the machine moves around you to deliver the radiotherapy from different angles. You will not feel anything, as it is similar to having an x-ray.

During your radiotherapy treatment you will be seen by your doctor and/or nurse/radiographer every week to record and treat any side effects that you may be experiencing. They will also take a small sample of blood before treatment starts and again during the first, fourth and sixth week of radiotherapy.

Hafeez S, *et al. BMJ Open* 2020; 10:e041005. doi: 10.1136/bmjopen-2020-041005

Chemotherapy treatment during radiotherapy

Your doctor may suggest that you also have chemotherapy while you have your radiotherapy treatment. You will be able to take part in RAIDER whether or not you have chemotherapy. Your doctor will discuss the possibility of chemotherapy with you.

How often will I need to visit the hospital after my treatment?

Everyone in the RAIDER study will be asked to visit the hospital for check-ups on the same schedule after treatment,as described below:

- *10 weeks after the start of radiotherapy:* assessment of side effects
- *3 months after the end of radiotherapy:* cystoscopy (inspection of your bladder with a telescope) under general anaesthetic & biopsy of the site of the tumour; blood sample; chest x-ray; assessment of side effects
- *6 months:* cystoscopy under local anaesthetic; blood sample; CT scan of abdomen and pelvis; chest x-ray or CT scan; assessment of side effects
- *9 months:* cystoscopy under local anaesthetic; assessment of side effects
- *12 months:* cystoscopy under local anaesthetic; CT scan of abdomen and pelvis; chest x-ray or CT scan; assessment of side effects
- *18 months:* cystoscopy under local anaesthetic; chest x-ray or CT scan; assessment of side effects
- *24 months:* cystoscopy under local anaesthetic; CT scan of abdomen and pelvis; chest x-ray or CT scan; assessment of side effects
- *36, 48, 60 months:* cystoscopy under local anaesthetic; chest x-ray; assessment of side effects

A summary of these visits is on page 15 of the patient information sheet, for your reference should you decide to join the study.

These visits have been designed to be as similar as possible to what would happen if you decided not to join the RAIDER study. If your cancer is found to have returned during study check-ups your doctor will discuss further treatment options with you.

After your 5 year (60 month) visit, we would like to collect basic information about your health from any routine follow up visits you have, and also from national electronic databases which are kept on everyone’s health status.

Hafeez S, *et al. BMJ Open* 2020; 10:e041005. doi: 10.1136/bmjopen-2020-041005

RAIDER
Study Design

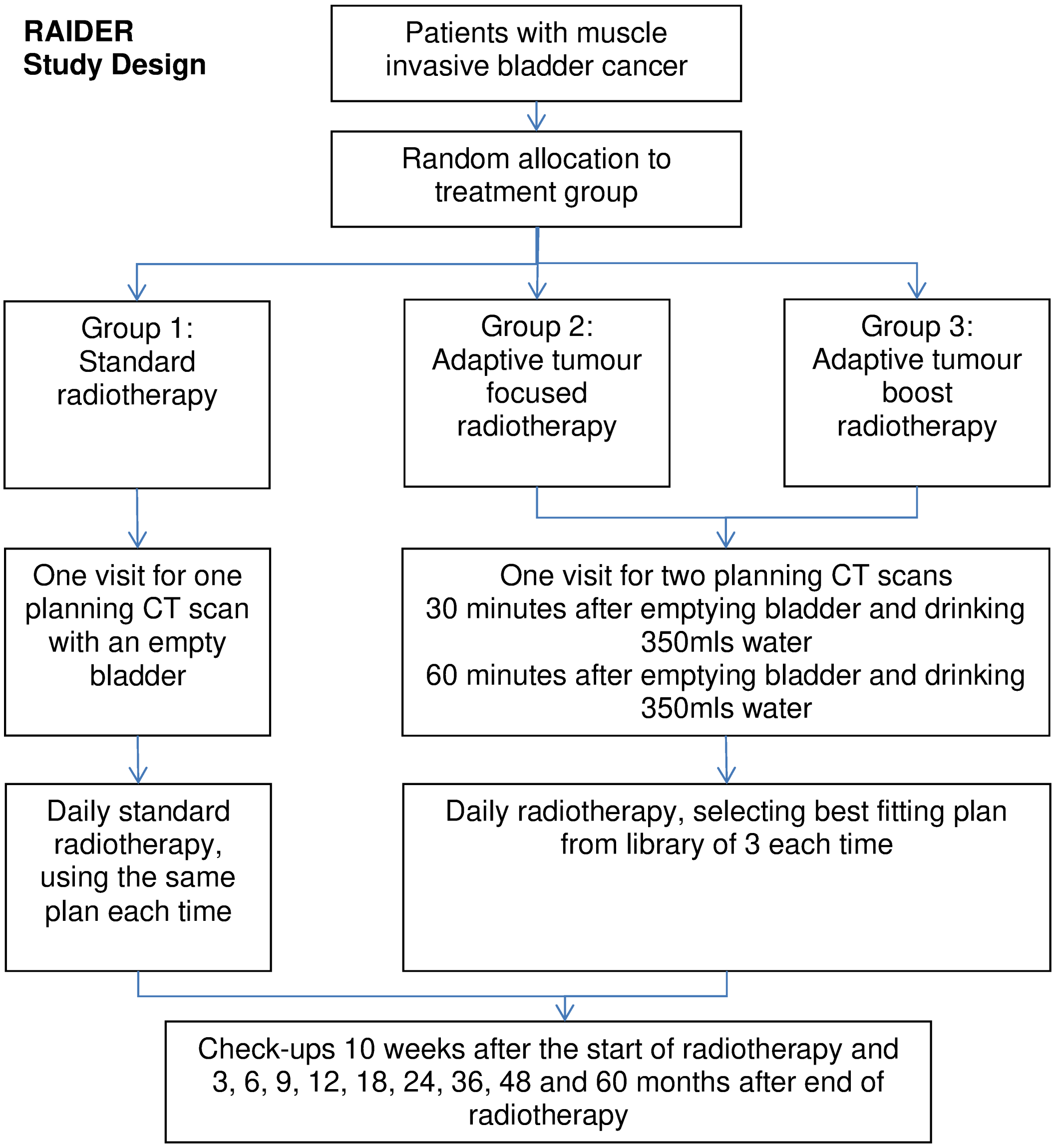

What are the possible side effects of treatment?

Patients who have radiotherapy can experience some side effects. These can occur in anyone receiving radiotherapy to the bladder whether or not they are in the RAIDER study. It is difficult to predict whether you will have some, all, or none of the side effects, or how severe they may be. They are usually mild and short lived but can sometimes be more serious.

Please let your doctor or nurse know about any side effects that you are concerned about so they can advise you what to do. Their telephone numbers are listed on page 12. There is also 24 hour support available from your hospital, to provide access to immediate medical care in the event of any serious problems.

Not all patients will experience all of these side effects and we can give you medications to treat any side effects you may experience.

You will be able to carry out most of your normal activities during radiotherapy, but you may feel more tired than normal and may need to rest more.

Side effects that can develop during radiotherapy may include:
- diarrhoea (around 3 in 10 people)
- needing to urinate more often (around 5 in 10 people)
- bleeding, pain or discomfort on passing urine (around 2 in 10 people)
- passing stools more frequently or with pain (around 1 in 10 people)

Some side effects can develop several months after radiotherapy ends. These include:
- a need to urinate more often or more urgently (around 2 in 10 people)
- bowel changes due to scarring or bleeding (around 5 in 100 people)
- vaginal scarring (around 3 in 10 women)
- problems with getting and maintaining erections (around 2 in 10 men)
- infertility (all women, around 5 in 10 men)

A few patients may develop long term effects. These are usually mild but may occasionally be serious and require treatment.

If you are in group 3 and receive a tumour focused boost as part of your radiotherapy treatment, you will be receiving a higher total dose of radiotherapy to the tumour than people in other groups. There is therefore a possible increased risk of radiotherapy side effects, but the radiotherapy techniques used in this trial aim to keep the risk of side effects similar to those experienced following the standard dose. Side effects in group 3 will be very closely monitored throughout the study and if they appear to be higher than expected, treatment within this group will be stopped, and everyone in group 3 will receive standard dose tumour focused radiotherapy.

Do I have to take part?

No, it is up to you to decide whether to take part or not. If you decide to take part, you will be given this information sheet to keep and will be asked to sign a consent form. You are free to change your mind and withdraw from the study at any time without giving a reason. If you do choose to withdraw, your doctor will discuss with you the best treatment options available.

What are the alternatives to this study?

If you decide not to participate in this study, it will not affect the usual standard of care you receive. Standard recommended treatments for muscle invasive bladder cancer are surgery to remove the bladder or daily radiotherapy using one treatment plan and the

Hafeez S, *et al. BMJ Open* 2020; 10:e041005. doi: 10.1136/bmjopen-2020-041005

same dose to treat the whole bladder. If you do not take part in RAIDER your doctor will discuss all your alternative options with you.

What are the possible benefits of taking part?
Everyone in the study will receive scans regularly throughout treatment. This may make the radiotherapy more accurate than if it was given without the scan. If you are in the adaptive radiotherapy groups you will receive radiotherapy treatment with the smallest possible safety margin each time and this may reduce the risk of side effects. If you are in the tumour focused boost group, the increased radiation dose to your tumour may control your bladder cancer better.

What are the possible disadvantages of taking part?
Tumour focused adaptive radiotherapy could cause an increased risk of missing the tumour, but everyone who gives treatment as part of RAIDER will be fully trained and treatment will be checked by a second trained observer before it is given. There is also a risk that sparing the bladder from exposure to full dose radiotherapy could cause an increased risk of the cancer returning elsewhere in the bladder, however previous studies have not suggested this, and any indication of this will be carefully monitored in all patients who join RAIDER.

It is possible that the side effects of tumour focused boost radiotherapy might be worse than with standard treatment, but this will be monitored for all RAIDER participants and the study will be stopped if people experience bad side effects.

The selection and confirmation of the radiotherapy treatment plan will extend the length of each radiotherapy treatment by about 5 to 10 minutes for patients receiving adaptive radiotherapy.

You may have more CT scans than you would if you did not take part in RAIDER because you will have one before each treatment, and if you are in the tumour focused groups you will have two planning scans rather than one. If you are in the tumour focused boost group you will receive a higher dose of radiotherapy than you would otherwise. All of these factors mean that you could be exposed to more radiation than you would be otherwise, which may lead to an increased risk of developing a second cancer later in life.

Before participating you should also consider if this will affect any insurance you have and seek advice if necessary.

How will confidentiality be maintained?
Your medical notes will be seen by authorised members of the research team at your hospital, so that they can collect information needed for the RAIDER study. When you join the study, your name, date of birth, postcode, hospital number and NHS or Community Health Index (CHI) number will be passed to the Institute of Cancer Research Clinical Trials and Statistics Unit(ICR-CTSU) where the study is being coordinated. You will be

Hafeez S, et al. BMJ Open 2020; 10:e041005. doi: 10.1136/bmjopen-2020-041005

given a unique registration number, which will be used together with your initials and date of birth on forms that the research staff will send to the ICR-CTSU. All information about you will be coded with this registration number and will be stored securely. It will be treated as strictly confidential and nothing that might identify you will be revealed to any third party.

Scientific employees of ICR-CTSU, and those conducting the study with them, including the national radiotherapy quality assurance team, may need to examine your medical records to ensure the study is being run properly and that the information collected on the forms is correct, but your confidentiality will be protected at all times.

We will contact your hospital over the years to find out how you are getting on. Ideally we would like to do this for life, but patients often change address or GP or lose touch with their hospital. If this happens we would like to use national records which are kept on everyone's health status to find out how you are. One of these is held at the General Register Office (GRO). We will need to give them enough information to identify you. This is usually your name, date of birth and NHS number (or Community Health Index and/or hospital number in Scotland). These details are confidential and will only be used for the purposes of the RAIDER study. Please initial the consent form to show that we have your permission to do this – if you do not agree, we will not seek this information.

The Institute of Cancer Research is the Sponsor for this study based in the United Kingdom. We will be using information from you and/or your medical records in order to undertake this study and will act as the data controller for this study. This means that we are responsible for looking after your information and using it properly. Our legal basis for processing your data is task in the public interest for scientific research purposes. The Institute of Cancer Research will keep identifiable information about you for 20 years after the study has finished.

Your rights to access, change or move your information are limited, as we need to manage your information in specific ways in order for the research to be reliable and accurate. If you withdraw from the study, we will keep the information about you that we have already obtained. To safeguard your rights, we will use the minimum personally-identifiable information possible.

You can find out more about how we use your information at www.icr.ac.uk/our-research/centres-and-collaborations/centres-at-the-icr/clinical-trials-and-statistics-unit/transparency.

[NHS site] will collect information from you and/or your medical records for this research study in accordance with our instructions.

[NHS site] will use your name, NHS number and contact details to contact you about the research study, and make sure that relevant information about the study is recorded for your care, and to oversee the quality of the study. Individuals from The Institute of Cancer Research and regulatory organisations may look at your medical and research records to check the accuracy of the research study. [NHS site] will pass these details to The Institute

Hafeez S, et al. BMJ Open 2020; 10:e041005. doi: 10.1136/bmjopen-2020-041005

of Cancer Research along with the information collected from you and/or your medical records. The only people in The Institute of Cancer Research who will have access to information that identifies you will be people who need to contact you to send a Quality of Life booklet by post or audit the data collection process. Only members of the research teams at your hospital and the ICR-CTSU will have access to the information that could allow this trial ID number to be linked to you.

[NHS site] will keep identifiable information about you from this study for 20 years after the study has finished.

Data sharing

When you agree to take part in a research study, the information about your health and care may be provided to researchers running other research studies in this organisation and in other organisations. These organisations may be universities, NHS organisations or companies involved in health and care research in this country or abroad. Your information will only be used by organisations and researchers to conduct research in accordance with the UK Policy Framework for Health and Social Care Research.

Your information could be used for research in any aspect of health or care, and could be combined with information about you from other sources held by researchers, the NHS or government.

Where this information could identify you, the information will be held securely with strict arrangements about who can access the information. The information will only be used for the purpose of health and care research, or to contact you about future opportunities to participate in research. It will not be used to make decisions about future services available to you, such as insurance.

Where there is a risk that you can be identified your data will only be used in research that has been independently reviewed by an ethics committee.

What will happen to the results of the research study?
Independent experts will review the safety and progress of the research whilst it is being carried out, and the results will be published in a respected medical journal once we are sure they are reliable. No information that could identify you will be included and you will not be identified in any report or publication.

We will summarise the results for participants once they are available. Your hospital will be able to give you a copy and results will also be available on Cancer Research UK's patient website (www.cancerhelp.org.uk).

What if relevant new information becomes available?
Sometimes we get new information about the treatment being studied. If this happens, your doctor will tell you and discuss whether you should continue in the study. If you decide not to carry on, your doctor will make arrangements for your care to continue.

Hafeez S, *et al. BMJ Open* 2020; 10:e041005. doi: 10.1136/bmjopen-2020-041005

Will I be paid for taking part in this study?
No. Neither you nor your doctor will be paid for taking part in this study.

What if there is a problem?
Any complaint about the way you have been dealt with during this study, or any possible harm you might suffer, will be addressed. Your progress will be watched closely and you will be offered whatever help is available to cope with any side effects. Occasionally some patients need a short stay in hospital for side effects to be treated, and on rare occasions these can be serious. If this were to happen, full details of what has happened will be reviewed carefully by the Doctor who has overall responsibility for the RAIDER study . It is unlikely that anything will go wrong with your treatment or care, but if you wish to complain about any aspect of the way you have been approached or treated during the course of the study you can do so using the normal NHS complaints procedure. Concerns should be raised by speaking to a member of staff at your hospital or by talking to the local Patient Advice and Liaison Service (PALS) which has been established in every NHS Trust.

NHS bodies are liable for clinical negligence and other negligent harm to individuals covered by their duty of care. In the event that something does go wrong and you are harmed during the research and this is due to someone‘s negligence then you may have grounds for a legal action for compensation against the NHS Trust but you may have to pay your legal costs. Alternative indemnity arrangements apply to private clinics.

What if I don’t want to carry on with the study?
You are free to withdraw from the study at any time. You do not have to give a reason and your future treatment and care will not be affected. If you change your mind about having treatment or follow up within this study, we would still like to collect information about how you are getting on. The information we need is routinely recorded in your medical records at your standard hospital visits and you would not need to do anything.

Who is organising and funding the research?
RAIDER is organised by doctors at the Royal Marsden Hospital in London, in collaboration with other leading doctors across the country and the Institute of Cancer Research Clinical Trials and Statistics Unit in London. The research is approved and funded by Cancer Research UK, who are providing funding to run the trial in the UK. The National Health Service Research and Development Executive will pay for any extra nursing and administrative costs incurred by participating hospitals and the National Institute for Health Research will pay for some of the costs of conducting the research at participating hospitals.

Who reviewed this study?
All research in the NHS is reviewed by an independent group of people, called a Research Ethics Committee, to protect participants’ safety, rights, wellbeing and dignity. RAIDER has been reviewed and approved by London – Surrey Borders Ethics Committee on behalf

Hafeez S, *et al. BMJ Open* 2020; 10:e041005. doi: 10.1136/bmjopen-2020-041005

of all hospitals throughout the UK. It has also been reviewed and approved by Cancer Research UK and reviewed and endorsed by patient and carer representatives from the NCRI Consumer Liaison Group (www.ncri.org.uk).

What happens now?

You will have some time to think about the study and make your decision. Your doctor, nurse or radiographer will be happy to answer any questions. You may wish to discuss it with your family or friends. Once you have reached your decision please let your doctor or nurse know. You will be asked to sign a consent form and will be given a copy to keep together with this information sheet. Please keep this information sheet and copies of the signed consent form. Your GP will be told that you are taking part in the RAIDER study. If at any time you have any questions about the study you should contact your hospital consultant.

Further information

Macmillan Cancer support is a registered charity and helps with all the things that people affected by cancer want and need, from specialist health care and information to practical, emotional and financial support (www.macmillan.org.uk).You can also learn more about clinical trials on the Cancer Research UK's patient website (www.cancerhelp.org.uk).

Contact details

If at any time you have any questions about the study please contact your local study team:

Local consultant's name:

Local research nurse/radiographer:

Address:

Telephone:

24 hour contact number:

Thank you for your interest in our research.

Optional components to the study:

If you agree to participate in the main RAIDER trial, you will be invited to take part in the following sub-studies:

1. Side effects questionnaire study
2. Donation of routine samples from surgery

If I want to be part of the RAIDER study, do I have to take part in the sub-studies?

No. Taking part in RAIDER does not mean you have to take part in the sub-studies. You will be given the chance to discuss RAIDER and you can then decide whether you want to take part.

The following pages of this information sheet give further details about these sub-studies.

1. Side effects questionnaire study

The main reason we are carrying out the RAIDER study is to look at the side effects of the radiotherapy treatment. If you decide to take part in RAIDER, we would like you to complete short questionnaires to describe any side effects that you may experience.

This is an optional part of the study but completed questionnaires will help us to understand more about the side effects of this radiotherapy treatment from your point of view. Completing a questionnaire should take no longer than 20 minutes.

If you agree to take part, we will ask you to fill in a questionnaire before you start radiotherapy, at the end of your radiotherapy treatment and then at 3, 6, 12, 18 and 24 months afterwards. We know from other patients that they feel such surveys are very important, but you do not have to complete them if you do not want to.

The first questionnaires will be given to you by your hospital. From 6 months onwards questionnaires will be posted to you at your home address by The Institute of Cancer Research Clinical Trials & Statistics Unit (ICR-CTSU).

Before sending you the questionnaire, ICR-CTSU will contact your hospital or GP to check how you are; therefore we would like to ask for your permission to give ICR-CTSU your full name and address as well as your GP's name and address. If you agree to this, please initial the consent form to show that we have your permission.

Hafeez S, *et al. BMJ Open* 2020; 10:e041005. doi: 10.1136/bmjopen-2020-041005

2. **Donating samples from surgery**

RAIDER gives us the opportunity to ask many people with cancer similar to yours whether we can collect samples that will aid future research.

When you were diagnosed bladder cancer, your hospital will have kept a sample of the tumour your surgeon removed during surgery. We would like your permission to collect this and samples which are taken at any future surgery you may have, so that we can look at it in combination with samples from other people who have joined the study. This will allow us to test for genetic differences in the make-up of individuals, indicate why they develop cancer and predict how they react to treatment. If we show that genetic differences do explain why some patients develop bladder cancer or react to their treatment differently, this knowledge could help many patients in the future. Any genetic analysis would be for research purposes only and will not affect any insurance you may hold.

When they are collected from your hospital, the samples will be coded and your personal details will be removed. The coding will maintain your confidentiality whilst allowing biological details to be compared to treatment findings.

The samples collected in this study will be stored indefinitely. It is possible that in the future other research may be carried out on the samples collected within this trial. This research may be conducted in the UK or overseas. Your personal details will not be shared with other researchers. Any future research on samples will be approved by an ethics committee before it is done.

Sample donation is entirely optional and you do not have to participate if you do not wish to. If you chose to join the study there will be a section to complete when you sign the study consent form to indicate if you agree to donate these samples.

Hafeez S, *et al. BMJ Open* 2020; 10:e041005. doi: 10.1136/bmjopen-2020-041005

Summary of study assessments

**when→** **what↓**	10 weeks after start of radiotherapy	3 months*	6 months*	9 months*	12 months*	18 months*	2 years	3 years	4 years	5 years
Assessment of side effects	✓	✓	✓	✓	✓	✓	✓	✓	✓	✓
Blood sample		✓	✓							
Cystoscopy under general anæsthetic with biopsy of tumour site		✓								
Cytoscopy under local anæsthetic			✓	✓	✓	✓	✓	✓	✓	✓
Chest x-ray		✓								
CT scan of abdomen & pelvis			✓		✓		✓			
Chest x-ray or CT scan			✓		✓	✓	✓	✓	✓	✓

*after the end of radiotherapy

Hafeez S, *et al. BMJ Open* 2020; 10:e041005. doi: 10.1136/bmjopen-2020-041005

(Form to be printed on hospital's headed paper)
CONSENT FORM

RAIDER:
A Randomised phase II trial of Adaptive Image guided standard or Dose Escalated tumour boost Radiotherapy in the treatment of transitional cell carcinoma of the bladder

Ethics Committee Reference: 15/LO/0539

RAIDER trial ID:

Name of Researcher taking consent:

Please write your initials in the box to the right of each statement if you agree, and please sign at the bottom

		**Initials**
1.	I confirm that I have read and understand the RAIDER Patient Information Sheet 32f Version 2.0 dated 23/01/2019 for the above study. I have had the opportunity to consider the information, ask questions and have had these answered satisfactorily.	
2.	I understand that my participation is voluntary and that I am free to withdraw at any time without giving any reason, without my medical care or legal rights being affected.	
3.	If I withdraw from the study, I consent to my doctor providing authorised researchers with basic clinical information that would be routinely collected and written in my medical records.	
4.	I understand that sections of any of my medical notes may be looked at by responsible individuals from the research team, from ethics committees, or from the NHS Trust, where it is relevant to my taking part in research. I give permission for these individuals to have access to my records.	
5.	I consent to the Institute of Cancer Research using information held by the NHS and national databases to follow up my health status.	
6.	I agree to my GP being informed of my participation in the study.	
7.	*Data sharing: I grant advance authorisation for the possible future sharing of information collected about me with other organisations, with the understanding that I will not be identifiable from this information (optional).*	

Hafeez S, *et al. BMJ Open* 2020; 10:e041005. doi: 10.1136/bmjopen-2020-041005

8. *I agree to participate in the side effects questionnaire study. (optional)* ☐

   8a. *I consent to researchers from The Institute of Cancer Research being sent my address and GP contact details.* ☐

   8b. *I consent to researchers from The Institute of Cancer Research contacting my GP to confirm I am fit and well to receive questionnaire booklets to be sent out by post.* ☐

   8c. *I consent to my GP disclosing my health status to researchers from The Institute of Cancer Research* ☐

9. *I consent to donating routinely collected samples from surgery (optional)* ☐

   9a. *I agree that my tumour tissue samples will be analysed for potential changes in DNA (genetic changes)* ☐

   9b. *I grant advance authorisation for the possible future sharing of information collected about me with other organisations, with the understanding that I will not be identifiable from this information* ☐

   9c. *I grant advance authorisation for possible future research on my stored samples, with the understanding that I will not be identifiable from these samples. I understand that that approval of an ethics committee will be obtained beforehand.* ☐

10. I agree to take part in RAIDER ☐

....	....	....
Name of participant	Date	Signature
....	....	....
Name of person taking consent	Date	Signature

1 copy for participant; 1 copy for Principal Investigator; 1 copy to be kept with hospital notes

RAIDER consent 32f
2 pages

Version 2.0 23/01/2019
