# Supplementary material for: Protocol for tumour-focused dose-escalated adaptive radiotherapy for the radical treatment of bladder cancer in a multicentre phase II randomised controlled trial (RAIDER): radiotherapy planning and delivery guidance
Source: BMJ Open. 2020 Dec 31;10(12):e041005. doi: 10.1136/bmjopen-2020-041005 (PMC7780718; doi:10.1136/bmjopen-2020-041005)
Supplement: Author's manuscript [file bmjopen-2020-041005.draft_revisions.pdf]

BMJ Open is committed to open peer review. As part of this commitment we make the peer review history of every article we publish publicly available.

When an article is published we post the peer reviewers' comments and the authors' responses online. We also post the versions of the paper that were used during peer review. These are the versions that the peer review comments apply to.

The versions of the paper that follow are the versions that were submitted during the peer review process. They are not the versions of record or the final published versions. They should not be cited or distributed as the published version of this manuscript.

BMJ Open is an open access journal and the full, final, typeset and author-corrected version of record of the manuscript is available on our site with no access controls, subscription charges or pay-per-view fees (http://bmjopen.bmj.com).

If you have any questions on BMJ Open's open peer review process please email info.bmjopen@bmj.com

BMJ Open

Protocol for tumour focused dose escalated adaptive radiotherapy for the radical treatment of bladder cancer in a multi-centre phase II randomised controlled trial (RAIDER): radiotherapy planning and delivery guidance

Journal:	*BMJ Open*
Manuscript ID	bmjopen-2020-041005
Article Type:	Protocol
Date Submitted by the Author:	31-May-2020

Complete List of Authors:	Hafeez, Shaista; The Institute of Cancer Research, Radiotherapy and Imaging ; Royal Marsden NHS Foundation Trust, Radiotherapy and Imaging<br>Webster, Amanda; National Radiotherapy Trials Quality Assurance Group, Mount Vernon Hospital<br>Hansen, Vibeke; Odense University Hospital, Laboratory of Radiation Physics<br>McNair, Helen; The Institute of Cancer Research, Radiotherapy and Imaging ; Royal Marsden Hospital NHS Trust, Radiotherapy and Imaging<br>Warren-oseni, Karole; The Institute of Cancer Research, Joint Department of Physics ; Royal Marsden Hospital NHS Trust, Joint Department of Physics<br>Patel, Emma; National Radiotherapy Trials Quality Assurance Group, Mount Vernon Hospital<br>Choudhury, A; The University of Manchester, Faculty of Biology, Medicine and Health; Christie NHS Foundation Trust<br>creswell, Joanne; South Tees Hospitals NHS Foundation Trust<br>Foroudi , Farshad ; Austin Health, Department of Radiation Oncology<br>Henry, Ann; University of Leeds; Leeds Teaching Hospitals NHS Trust<br>Kron, Tomas ; Peter MacCallum Cancer Centre<br>McLaren, Duncan ; Western General Hospital<br>Mitra, Anita; University College London Hospitals NHS Foundation Trust<br>Mostafid, Hugh; Royal Surrey County Hospital NHS Foundation Trust, The Stokes Centre for Urology<br>Saunders, Daniel; Nottingham University Hospitals NHS Trust<br>Miles, Elizabeth; National Radiotherapy Trials Quality Assurance Group, Mount Vernon Hospital<br>Griffin, Clare; The Institute of Cancer Research, Clinical Trials and Statistics Unit<br>Lewis, Rebecca; The Institute of Cancer Research, Clinical Trials and Statistics Unit<br>Hall, Emma; The Institute of Cancer Research, Clinical Trials and Statistics Unit<br>Huddart, Robert; The Institute of Cancer Research, Department of Radiotherapy and Imaging; Royal Marsden Hospital NHS Trust, Department of Radiotherapy and Imaging

Keywords:	ONCOLOGY, Urological tumours < ONCOLOGY, RADIOTHERAPY, Clinical

	trials < THERAPEUTICS

SCHOLARONE™
Manuscripts

I, the Submitting Author has the right to grant and does grant on behalf of all authors of the Work (as defined in the below author licence), an exclusive licence and/or a non-exclusive licence for contributions from authors who are: i) UK Crown employees; ii) where BMJ has agreed a CC-BY licence shall apply, and/or iii) in accordance with the terms applicable for US Federal Government officers or employees acting as part of their official duties; on a worldwide, perpetual, irrevocable, royalty-free basis to BMJ Publishing Group Ltd ("BMJ") its licensees and where the relevant Journal is co-owned by BMJ to the co-owners of the Journal, to publish the Work in this journal and any other BMJ products and to exploit all rights, as set out in our licence.

The Submitting Author accepts and understands that any supply made under these terms is made by BMJ to the Submitting Author unless you are acting as an employee on behalf of your employer or a postgraduate student of an affiliated institution which is paying any applicable article publishing charge ("APC") for Open Access articles. Where the Submitting Author wishes to make the Work available on an Open Access basis (and intends to pay the relevant APC), the terms of reuse of such Open Access shall be governed by a Creative Commons licence – details of these licences and which Creative Commons licence will apply to this Work are set out in our licence referred to above.

Other than as permitted in any relevant BMJ Author's Self Archiving Policies, I confirm this Work has not been accepted for publication elsewhere, is not being considered for publication elsewhere and does not duplicate material already published. I confirm all authors consent to publication of this Work and authorise the granting of this licence.

For peer review only

Protocol for tumour focused dose escalated adaptive radiotherapy for the radical treatment of bladder cancer in a multi-centre phase II randomised controlled trial (RAIDER): radiotherapy planning and delivery guidance

Hafeez S[1], Webster A[2], Hansen N V[3], McNair H[1], Warren-Oseni K[1], Patel E[2], Choudhury A[4], Cresswell J[5], Foroudi F[6], Henry A[7], Kron T[8], McLaren D B[9], Mitra A[10], Mostafid H[11], Saunders D[12], Miles E[2], Griffin C[13], Lewis R[13], Hall E[13], Huddart R[1]

[1]Division of Radiotherapy and Imaging, The Institute of Cancer Research and The Royal Marsden NHS Foundation Trust, London, UK.
[2]National Radiotherapy Trials Quality Assurance Group, Mount Vernon Hospital, Northwood, UK
[3]Odense University Hospital, Laboratory of Radiation Physics, Denmark
[4]Faculty of Biology, Medicine and Health, University of Manchester and The Christie NHS Foundation Trust, Manchester, UK
[5]Department of Surgery, South Tees Hospitals NHS Foundation Trust, UK
[6]Department of Radiation Oncology, Austin Health, Melbourne, Australia
[7]University of Leeds and the Leeds Teaching Hospital NHS Trust, Leeds, UK
[8]Department of Physics, Peter MacCallum Cancer Centre, Melbourne, Australia
[9]Western General Hospital, NHS Lothian, Edinburgh, Scotland, UK
[10]University College Hospital, London, UK
[11]The Stokes Centre for Urology, Royal Surrey, Guildford, UK
[12]Nottingham University Hospitals NHS Trust, Nottingham, UK
[13]Clinical Trials and Statistics Unit, The Institute of Cancer Research, London, UK.

Author for correspondence
Dr S Hafeez
The Royal Marsden NHS Foundation Trust
Downs Road
Sutton
Surrey
SM2 5PT
UK
shaista.hafeez@icr.ac.uk

For peer review only

Abstract

Introduction

Daily radiotherapy delivered with radiosensitisation offers patients with muscle invasive bladder cancer (MIBC) comparable outcomes to cystectomy with functional organ preservation. Most recurrences following radiotherapy occur within the bladder. Increasing the delivered radiotherapy dose to the tumour may further improve local control. Developments in image guided radiotherapy have allowed bladder tumour focused 'plan of the day' radiotherapy delivery. We aim to test within a randomised multi-centre phase II trial whether this technique will enable dose escalation with acceptable rates of toxicity.

Methods and analysis

Patients with T2-T4aN0M0 unifocal MIBC will be randomised (1:1:2) between standard/control whole bladder single plan radiotherapy (WBRT), standard dose adaptive tumour focused radiotherapy (SART), or dose escalated adaptive tumour focused radiotherapy (DART). Adaptive tumour focused radiotherapy will use a library of three plans (small, medium, and large) for treatment. A cone beam CT taken prior to each treatment will be used to visualise the anatomy and inform selection of the most appropriate plan for treatment.

Two radiotherapy fractionation schedules (32f and 20f) are permitted. A minimum of 120 participants will be randomised in each fractionation cohort (to ensure 57 evaluable DART patients per cohort).

A comprehensive radiotherapy quality assurance programme including pre-trial and on-trial components is instituted to ensure standardisation of radiotherapy planning and delivery.

The trial has a two-stage non-comparative design. The primary endpoint of stage I is the proportion of patients meeting pre-defined normal tissue constraints in the DART group. The primary endpoint of stage II is late CTCAE grade 3 or worse toxicity aiming to exclude a rate of >20% (80% power and 5% alpha, one sided) in each DART fractionation cohort. Secondary endpoints include loco-regional MIBC control, progression-free survival overall survival, and patient reported outcomes.

Ethics

The trial is approved by the London-Surrey Borders Research Ethics Committee, (15/LO/0539).

For peer review only - http://bmjopen.bmj.com/site/about/guidelines.xhtml

For peer review only

Trial registration number NCT02447549.

Keywords

muscle invasive bladder cancer, image guided adaptive radiotherapy, randomised controlled trial, phase II

For peer review only

For peer review only - http://bmjopen.bmj.com/site/about/guidelines.xhtml

Strengths and limitations of this study

- Phase II international multi-centre randomised controlled study evaluating a novel adaptive radiotherapy technique (strength).
- Treatment allocation favours 75% of participants receiving novel adaptive radiotherapy techniques (strength).
- Detailed guidance and training are provided for the contouring, planning, and delivery of this radiotherapy technique to ensure standardisation across participating centres with robust pre-trial and on trial radiotherapy quality assurance programme (strength).
- Primary endpoint focus is based on determining safety of treatment based on late grade 3 toxicity scoring (strength).
- Non-comparative trial design (limitation).

For peer review only

For peer review only - http://bmjopen.bmj.com/site/about/guidelines.xhtml

Article summary

We present the first international randomised controlled trial protocol evaluating a dose escalated tumour focused image guided adaptive radiotherapy technique. The study population are patients with unifocal localised muscle invasive bladder cancer. Patients will be randomised (1:1:2) between standard (control) whole bladder single plan radiotherapy (WBRT), or an adaptive standard dose adaptive tumour focused radiotherapy (SART), or dose escalated adaptive tumour focused radiotherapy (DART). For those randomised to adaptive tumour focused radiotherapy groups treatment will be delivered using a library of three plans (plan of the day). If successful, the trial will demonstrate feasibly of multicentre implementation of this new radiotherapy technique and inform design of a future phase III trial to establish the optimum organ preserving treatment option for patients with MIBC.

For peer review only

For peer review only - http://bmjopen.bmj.com/site/about/guidelines.xhtml

Introduction

Radical management of localised muscle invasive bladder cancer (MIBC) involves either radical cystectomy or a course of daily radiotherapy delivered with radiosensitisation over 4-7 weeks [1-5]. Although both have comparable overall survival outcomes in appropriately selected patients, radiotherapy offers opportunity for cancer cure with functional organ preservation [6].

Most recurrences following radiotherapy occur within the bladder, the majority of which are believed to occur at the original MIBC tumour site, suggesting persistent occult local disease [7]. The modelled dose response relationship of MIBC to radiotherapy suggests improved local control and overall survival would be expected at higher doses [8-10].

The ability to safely increase dose beyond the current accepted standard has been restrained by reliable radiotherapy delivery to the bladder. The bladder is a mobile organ which is subject to marked shape and volume change during the course of treatment [11-13]. This bladder motion means historically up to 57% of fractions (f) incur some element of geographical miss even when safety margins of up to 1.5cm are applied to create the planning target volume (PTV) [14]. The expected consequence of improving bladder radiotherapy targeting would be improved tumour control and reduced treatment related toxicity.

Optimisation of target coverage has been enabled by technology integrated on current generation linear accelerators which allow a 3D image known as a cone beam CT (CBCT) to be acquired. This is of sufficient contrast to allow soft tissue visualisation. When acquired immediately prior to treatment it informs positional adjustment to ensure coverage of target with the radiotherapy plan [15].

A solution enabled by CBCT soft tissue visualisation is 'plan of the day'. Rather than having a single plan available for treatment, a library of plans of varying PTV bladder sizes can be created to cover the range of expected filling and positional variation of the bladder. A plan which best fits the bladder target with least normal tissue irradiation as seen on CBCT immediately prior to treatment is then selected for use each day [14]. In bladder cancer radiotherapy treatment delivery based on a library of plans has reported benefit in reducing normal tissue irradiation compared to single plan treatment delivery [16-19]. It is yet to be demonstrated whether this approach translates to improved clinical outcomes.

For peer review only - http://bmjopen.bmj.com/site/about/guidelines.xhtml

Tumour focused radiotherapy delivery may offer further opportunity to reduce normal tissue irradiation. Sparing the uninvolved bladder does not appear to compromise local control but randomised controlled studies have failed to demonstrate statistically significant improvement in toxicity [20, 21]. Bladder sparing is unlikely to have been optimally achieved in radiotherapy delivery pre-dating CBCT image guidance given the positional uncertainties, the large margins applied, and treatment delivery on an empty bladder.

In a single centre phase I study (NCT01124682) feasibility and safety of tumour focused dose escalation to 70Gy delivered using plan of the day has been demonstrated. The RAIDER trial seeks to examine feasibility of this approach in a multi-centre setting and to determine the clinical benefit of bladder tumour focused dose escalation.

Below, we describe the RAIDER trial protocol with particular emphasis on the radiotherapy procedural aspects, including preparatory imaging, treatment planning, and delivery with the aim of providing comprehensive description of the radiotherapy implemented for the study.

Hypothesis

Tumour focused dose escalated adaptive radiotherapy using library of three plans can be translated to multiple centres. It will be well tolerated and offer the opportunity to improve local disease control for bladder cancer patients.

For peer review only

Materials and analysis

Study design

RAIDER is an international multicentre, multi-arm, two-stage non-blinded phase II randomised controlled trial conducted in accordance with the Research Governance Framework for Health and Social Care and principles of Good Clinical Practice. The trial is registered on the clinicaltrials.gov database (NCT02447549) and is included in the National Institute for Health Research (NIHR) Clinical Research Network portfolio. The final ethics approved version of RAIDER trial protocol is provided in the supplementary files (Appendix 1).

Patients will be randomised (1:1:2) between standard (control) whole bladder single plan radiotherapy (WBRT), standard dose adaptive tumour focused radiotherapy (SART), or dose escalated adaptive tumour focused radiotherapy (DART). Treatment allocation is by minimisation with a random element; balancing factors will be centre, neo-adjuvant chemotherapy use and concomitant radiosensitising therapy use. Randomisation will take place centrally by the trials unit (ICR-CTSU) within a maximum of 10 weeks prior to the planned radiotherapy start date.

Within the UK there are two commonly used radiotherapy schedules to treat bladder cancer, both supported by the National Institute of Clinical for Health and Clinical Excellence (NICE)[1]. Therefore, to accommodate this practice radiotherapy will be delivered daily in either 20f over 4 weeks or 32f over 6.5 weeks in accordance with the participating centre's standard practice. The choice of fractionation will be confirmed by each site before trial commencement and will be used for all patients at that site. The two fractionation cohorts will be analysed separately for the primary end point.

For stage I, the primary endpoint is the proportion of participants in the DART group meeting the pre-defined normal tissue radiotherapy dose constraints. The secondary endpoints of stage I are recruitment rate and the ability of the participating centres to deliver SART and DART treatment as per protocol.

For stage II, the primary endpoint is grade 3 or greater toxicity occurring 6-18 months following radiotherapy as assessed using Common Terminology Criteria for Adverse Events (CTCAE v.4). The secondary endpoints of stage II are acute toxicity as measured by CTCAE v.4, patient reported outcomes as measured by a number of

For peer review only

instruments including the Patient-Reported Outcomes version of the Common Terminology Criteria for Adverse Events (PRO- CTCAE™), Assessment of Late Effects of RadioTherapy - Bowel (ALERT-B), the King's Health Questionnaire (KHQ), sexual function questions and the 5-level EQ-5D version (EQ5D-5L). Additional secondary endpoints include health economic related measures, loco-regional MIBC control, progression free survival, and overall survival.

The trial has a number of exploratory secondary endpoints related to use of adaptive plans including appropriate identification of plan selection, target coverage, and dose volume comparison between control (WBRT) and adaptive (SART and DART) planning.

Figure 1 shows the trial schema and overview of follow-up. Table 1 provides summary of the scheduled pre-randomisation, on treatment, and post treatment assessments.

Participants and eligibility

Total target recruitment is set at a minimum of 240 participants with a minimum 120 be recruited to each fractionation cohort (20f or 32f cohort). The final sample size in each fractionation cohort will be determined as that sufficient to accrue 57 DART patients evaluable for the primary endpoint of late toxicity.

Patients with histological or cytological confirmation of unifocal (T2-T4aN0M0) transitional cell carcinoma (TCC) of the bladder suitable for radical daily radiotherapy will be approached for inclusion. Eligible patients should be willing to accept assessment with cystoscopy and follow-up schedule as outlined in Table 1.

Patients with multi-focal invasive disease or history of other malignancy within 2 years of randomisation except for non-melanomatous skin carcinoma, previous non-muscle invasive bladder tumours, and low risk prostate cancer (as defined by NCCN risk stratification as T1/T2a, Gleason 6 PSA <10) will be excluded. Those with bilateral prosthetic hip replacements, previous history of radiation to the pelvis or other contra-indication to pelvic radiotherapy e.g. inflammatory bowel disease will also be excluded.

For peer review only - http://bmjopen.bmj.com/site/about/guidelines.xhtml
For peer review only

Study treatment

All participants should have had a trans urethral resection of the bladder tumour (TURBT) with completion of a bladder map by the performing urologist to aid tumour localisation for radiotherapy. Insertion of fiducial markers to further assist tumour localisation for radiotherapy is also recommended at the time of cystoscopy. Neo-adjuvant chemotherapy use prior to randomisation is permitted and encouraged for suitable patients.

Radiotherapy should be planned to commence within a maximum of 10 weeks after randomisation or neo-adjuvant chemotherapy completion (if used), to allow sufficient time for treatment planning.

Delivery of radiotherapy with concomitant radiosentiser is permitted. Regimes approved for use within the protocol include mitomycin C and 5FU [2], gemcitabine [22], cisplatin [23], or carbogen [3]. Each centre should aim to use the same regimen for all their participants. Where this is not possible appropriate substitution is permitted for that participant following discussion with the RAIDER lead investigators.

Participants allocated to the WBRT (control) group will have one radiotherapy plan created treating the whole empty bladder to either 64Gy in 32f or 55Gy in 20f. A CBCT scan acquired just prior to treatment delivery can be used by the local investigators to inform an online position correction in accordance with National Radiotherapy Implementation Group Report on Image Guided Radiotherapy (IGRT) [15] and standard local practice.

Participants allocated to the adaptive tumour focused planning groups (SART and DART) will have three radiotherapy plans generated a small, medium, and large plan. The bladder tumour boost volume will be treated to either standard dose (64Gy in 32f or 55Gy in 20f) or escalated dose (70Gy in 32f or 60Gy in 20f) depending on whether the participant is allocated to SART or DART respectively. The uninvolved bladder will receive a lower planned dose either 52Gy/32f or 46Gy/20f depending on fractionation cohort irrespective of SART or DART randomisation. A CBCT taken immediately prior to each treatment delivery will be used to select the most appropriate 'plan of the day' depending on the bladder volume and shape. A second trained individual verifies the plan selected for treatment.

For peer review only - http://bmjopen.bmj.com/site/about/guidelines.xhtml

Plan selection is authorised to be carried out only by radiographers or other delegated practitioners who have attained concordance with the gold standard PTV selection through the Radiotherapy Trials Quality Assurance Group (RTTQA) IGRT credentialing for UK centres and Trans Tasman Radiation Oncology Group (TROG) IGRT credentialing for Australian and New Zealand centres. This is to ensure all those participating in plan selection have the necessary advanced skill level required for the study.

A comprehensive quality assurance (QA) programme has been implemented for the RAIDER trial. This includes pre-trial and on-trial components. Selection of appropriate treatment plans for the adaptive planning group will be also be independently monitored during patient recruitment as part of the radiotherapy QA process.

Radiotherapy planning and delivery

Radiotherapy planning CT scan

Bladder preparation procedures vary depending on randomisation group. For WBRT, an empty bladder is required. Patients should be asked to abstain from drinking fluids for 30 minutes before the scheduled planning CT scan and are required to void their bladder immediately before the planning CT scan is acquired (CT0).

For both SART and DART groups patients are instructed to void their bladder and then drink 350mls of water. Two planning scans are acquired, the first at 30 minutes following drinking (CT30) and the second 60 minutes following drinking (CT60). No voiding is permitted between the two scans. However, if voiding is unavoidable because of patient discomfort, then only the available CT30 scan is used for planning.

Given bladder deformation can occur with a loaded rectum, all participants should be encouraged to evacuate their bowels of flatus and faeces prior to acquisition of the radiotherapy planning scanning. The use of micro enemas is permitted if it is standard local practice but is not mandated.

All patients will be positioned supine with arms comfortably positioned out of the radiotherapy field using appropriate immobilisation techniques for planning CT scan acquisition. CT slices of $\leq$3 mm thickness will be obtained from at least 4cm above

For peer review only - http://bmjopen.bmj.com/site/about/guidelines.xhtml

the dome of the bladder to 2cm below the ischial tuberosities. No oral or intravenous contrast is required.

The planning CT scan is exported via DICOM transfer to the radiotherapy treatment planning system for target and organs at risk (OAR) localisation. Bladder filling occurring between CT30 and CT60 scans is determined for those randomised to SART or DART. This is achieved by fusing both CT30 and CT60 data sets and contouring the bladder on both scans. If the difference in bladder volume between the two scans is less than 50mls i.e. no significant bladder filling occurs, then all target and OAR contours are created using CT30. If difference in bladder filling is greater than 50mls i.e. bladder filling occurs, the target volumes for large plan is created using CT60 anatomy.

Target volume definition

Volumes will be defined according to the International Commission on Radiation Units and Measurements (ICRU) report 50, supplement report ICRU 62: Prescribing, Recording and Reporting Photon Beam Therapy and ICRU 83: Prescribing, Recording and Reporting Photon- Beam Intensity Modulated radiotherapy (IMRT) [24]. Consistent structure naming convention for target volumes and organs at risk is adopted for all patients participating within the trial.

The gross tumour volume (GTV) is defined as the bladder tumour or the resected tumour bed. It is delineated using position of fiducial markers (where available), diagnostic imaging (prior to neoadjuvant chemotherapy where applicable) and the surgical bladder map (where available). When delineating the tumour any extravesical tumour should be included in the GTV. If no tumour is visible then the appropriate section of the bladder should be included based on surgical bladder map following discussion with the urologist who performed the TURBT. Alternatively, repeating the cystoscopy and placing fiducial markers adjacent to resected bladder tumour scar should be considered.

The clinical target volume (CTV) is contoured to encompass the GTV, the whole bladder, and any area of extravesical spread. The CTV should also include 1.5cm of prostatic urethra in male patients or 1cm of urethra in female patients if tumour is at the base of bladder or if distant carcinoma in situ (CIS) is present.

For peer review only - http://bmjopen.bmj.com/site/about/guidelines.xhtml

A checklist for contouring is provided in the radiotherapy planning and delivery guidelines (supplementary file, Appendix 2, p17). The expansions applied to generate the PTVs are summarised in Table 2. The PTV expansion margins were derived from earlier phase I work [14, 16, 25].

Organs at risk delineation

Organs at risk (OARs) are identified as other bowel, rectum, and femoral heads in all groups. To quantify normal bladder sparing, the normal bladder outside the boost (PTV2) is also identified for participants in the adaptive tumour focused radiotherapy groups.

All OARs will be outlined as solid structures by defining their outer wall. The rectum is outlined to include the full circumference and rectal contents. The rectal outlining should extend from the lowest level of the ischial tuberosities to the rectosigmoid junction which identified as the level at which there is an anterior inflection of the bowel, best appreciated on sagittal reconstructions on the CT planning scan.

The small and large bowel (including sigmoid colon) will be outlined as a single structure labelled ‘other bowel’. Small and large bowel visible on relevant axial slices of the planning scan will be outlined as individual loops. The cranial extent of ‘other bowel’ outlining should be 2cm beyond the superior extent of the standard PTV or large PTV as appropriate.

Both the femoral heads are outlined to the bottom of the femoral head curvature. The femoral necks not included.

The normal bladder outside the boost (PTV2) is created by subtracting the PTV2 from the corresponding CTV.

Radiotherapy planning

All patients are CT planned. For WBRT, a single plan created using either 3D conformal radiotherapy (3DCRT) with three or four fields, static 5-7-field intensity modulated radiotherapy (IMRT), or volumetric modulated arc radiotherapy (VMAT) technique is permitted. It is accepted that the preferred treatment planning method will vary between participating sites but should be specified in the centre’s pre-trial process document and be used for all patients enrolled at that centre. Changes in

For peer review only - http://bmjopen.bmj.com/site/about/guidelines.xhtml

For peer review only

centres preferred planning method from that specified should be brought to the attention of RTTQA.

For participants in the adaptive tumour focused radiotherapy groups, the planning and dose calculation is done on CT30 data set, therefore all target and OARs volumes are assigned to the CT30 scan. They will have three plans created (small, medium, or large) generated from the respective PTV and PTV2 volumes. To enable bladder sparing, these plans are created using either static 5-7-field IMRT or VMAT. The same technique should be used for all patients randomised to adaptive tumour focused radiotherapy at that centre.

The prescription doses for the PTV are outlined in Table 3. All plans should be created with the intention of achieving the target volume objectives as outlined in Table 4. Dose to OARs should be as low possible. The OARs dose volume constraints for both fractionations are summarised in Table 5.

The other bowel, rectum, and femoral heads constraints for the 32f schedule were derived from previous phase III prostate (CHHiP) and bladder (BC2001) studies [2, 26, 27] and from phase I work [28]. The absence of previously defined OARs constraints when dose escalating in 20f meant that the OARs constraints at higher doses were marginally more conservative than if otherwise converted exactly from 32f constraint level using the linear quadratic model alone [29]. The constraints used for the 20f schedule were estimated from the 32f constraint level using the linear quadratic equation assuming that all α/β of organs at risk is 3 but the dose constraint is reached in 3Gy per fraction.

Dose objectives to the PTV should not be compromised to achieve dose to OAR constraints. The recommended hierarchy of planning priorities is provided radiotherapy planning and delivery guidelines (supplementary file, Appendix 2, p27).

For patients randomised to WBRT, it is at the local principal investigator's (PI) discretion to accept the OAR doses. For those randomised to adaptive tumour focused radiotherapy groups it is recommended that the pre-defined optimal dose constraints are met for the small plan, and the mandatory constraints for the medium plan wherever possible. It is accepted that the rectum and bowel dose constraints of the large plan may not be met despite adequate optimisation. Assessment of 'other bowel' dose on the large plan represents an over estimation of actual dose compared

For peer review only - http://bmjopen.bmj.com/site/about/guidelines.xhtml

to 'other bowel' when this plan is actually used to deliver treatment. This is because when the large plan is selected for treatment, a proportion of bowel moves out of the field with bladder filling.

For patients allocated to DART, if the mandatory constraints are not met on the medium plan advice must be sought from the RTTQA team. Decision will be then made by the RAIDER trial team regarding the appropriateness of proceeding at the DART prescription dose or to lowering the prescribed dose as per SART randomisation. It is therefore recommended that the medium plan be optimised first. If patients are not able to receive DART (in either fractionation cohort) for any reason then details of the deviation from allocated treatment will be requested

Pre-radiotherapy checks

To minimise risk of error at the time of plan importing, exporting, and plan selection, it is recommended that each plan, beam name and ID reflect the assigned plan e.g. Sm_Plan used for labelling the beams making up the small plan in the adaptive tumour focused radiotherapy groups. It is also important to ensure that the local record and verify systems for 3DCRT and IMRT cannot mix beams from different plans at the time exporting or deliver more than one plan at treatment.

Treatment scheduling

Radiotherapy can start on any day of the week and should be delivered five days a week until completion. Interruptions during radiotherapy should be avoided as they have detrimental effect on outcome [30]. All missed fractions are to be reported to the ICR-CTSU and RTTQA team.

In the event of missed fractions due to machine breakdown, bank holiday, or any other logistical reason compensation for the missed fraction is advised. This is expected to be achieved by either treating at a weekend or by hyperfractionating i.e. undertaking two fractions a day (ideally on a Friday) with a minimum 6 hour gap between treatments. Should a treatment break occur due to toxicity, centres are advised to contact ICR-CTSU and/or RTTQA. Compensation is not expected in circumstances where missed treatment is a result of radiotherapy related toxicity.

For those allocated to adaptive tumour focused radiotherapy groups if plan selection capabilities are unavailable, either because of absence of trained staff, machine

For peer review only - http://bmjopen.bmj.com/site/about/guidelines.xhtml

breakdown and/or gap day treatment, patients may be treated for up to 5 days using the PTV medium plan without plan selection. These pre-treatment CBCTs (if acquired) should be sent to RTTQA for review.

Treatment delivery

The same patient preparation instructions used at planning CT should be implemented prior to each fraction delivered.

For those patients allocated to SART or DART, CBCT of the pelvis should be acquired prior to each fraction. For those patients randomised to WBRT, pre-treatment CBCT should be used in accordance with guidance provided in the NRIG IGRT report [15]. It is therefore expected that this CBCT will inform appropriate corrections (either manual or automatic) to be applied prior to the delivered fraction in accordance with the centre's local practice to ensure that treatment is accurately directed. Any changes made on the basis of the scan including exposures that do not lead to treatment because of patient factors should be reported in the case report forms (CRF) and to RTTQA.

For those randomised to adaptive tumour focused radiotherapy groups, the pre-treatment CBCT is acquired and registered to bone in accordance according to the guidance provided in the NRIG IGRT report [15]. An appropriately trained radiographer or practitioner reviews the bone matched CBCT assessing the bladder size and position in relation to the PTVs and the coverage they provide.

To assist trained radiographers or practitioners with optimal plan selection the following sequential assessment is advised:

i) Following CBCT acquisition, the bladder filling and shape is first checked against CTV_30 contour. If the bladder is of similar size and shape to the CTV at planning (i.e. CTV_30), then the small plan should be considered in the first instance for treatment.

ii) The appropriate plan provides suitable coverage of the CTV and boost region by the corresponding PTV and PTV2 contours with minimal normal tissue irradiation.

iii) Manual (soft tissue) moves should be made to ensure the bladder (CTV) is

For peer review only

adequately covered whilst selecting the smallest plan possible to spare normal tissue.

iv) Care should be taken when applying any soft-tissue shifts greater than 1cm as it can impact on the accuracy of the expected dosimetry. If shifts over 1cm occur they should be discussed with the planning department and RTTQA should be contacted following treatment.

v) Manual moves should be undertaken if further optimisation of PTV2 coverage can be achieved. Manual moves prioritising coverage to the boost region over the normal bladder wall is permitted if it avoids excessive normal tissue irradiation that would have occurred by selecting a larger plan.

vi) Finally, the OARs as seen on the CBCT is reviewed and compared to the position on the planning CT. The position of OARs relative to the boost is assessed to ensure that excessive normal tissue does not sit within the PTV2, especially for DART patients. If this is case, manual move is permitted to minimise normal tissue irradiation but should not be at the expense of target coverage.

vii) A second accredited radiographer or practitioner must confirm selected plan and any additional actions taken. Once agreement has been reached any necessary couch correction is performed prior to treatment delivery with the selected plan.

Fractions must not be omitted or missed due to unfavourable positioning of normal anatomy such as rectal distention due to flatus or faeces. Additional guidance and potential solutions are provided for scenarios that may arise on treatment are given in the radiotherapy planning and delivery guidelines (supplementary file, Appendix 2, p45). The flow chart of potential interventions is derived from phase I experience previously published [28].

For example, if the bladder is significantly smaller than the CTV_30 contour at planning, it is likely that the PTV2 boost will be in the incorrect position and, or does not achieve adequate normal bladder sparing. In these circumstances patients should be removed from the treatment couch, and encouraged to fill the bladder by drinking further, and or increasing the time interval of image acquisition.

For peer review only

In the event that the bladder has over filled and none of the PTVs provide adequate coverage despite manual moves, the patient should be asked to minimally void and the CBCT is repeated. If this is not possible, patient should void completely and restart drinking protocol with a reduction in the time interval for CBCT acquisition. In these circumstances a member of the clinical team should also be notified to ensure the patient is not in urinary retention.

When amending the drinking protocol to optimise patient's anatomy to fit the existing PTV contours, it is advised that one aspect is changed at a time i.e. interval for CBCT acquisition timing or the amount of water that is drunk. This is so the impact of the intervention can be determined and altered for subsequent fractions as required.

If no PTV contours are suitable to cover the target because of rectal gas, then the patient should be removed from the bed and ask to void. The CBCT image acquisition is then repeated. If the PTV contours still are not optimal, it is recommended that the most suitable plan is selected which optimises coverage of PTV2 and minimises the inclusion of OARs is chosen for treatment. If this occurs repeatedly (e.g. more than twice in 5 fractions) RTTQA should be contacted for advice.

All CBCT exposures including those not resulting in treatment should be recorded on the CRF and plan selection form.

In all randomised groups, a post treatment CBCT should be taken during the first week and once a week thereafter. This CBCT should be reviewed locally to ensure intra fraction filling has been accommodated for at the time of plan selection.

Radiotherapy protocol compliance programme

The RAIDER trial is subject to radiotherapy QA programme that aims to standardise contouring, planning, and delivery of image guided and adaptive bladder radiotherapy in participating centres. The RTTQA Group coordinates the UK QA programme for the study. For Australian and New Zealand participants, this is co-ordinated by the TROG QA Team.

The QA programme has a pre-trial and on-trial component. Each centre will be

For peer review only - http://bmjopen.bmj.com/site/about/guidelines.xhtml

required to complete the pre-trial QA prior to commencing recruitment.

Prior to trial entry, participating centres will be asked to complete a facility questionnaire in order to gauge current local IGRT experience. A separate process document is used to collect task details of all aspects of a complete patient pathway.

The PI at each participating site is asked to contour two benchmark clinical cases as per protocol. One case includes tumour bed GTV as defined by placement of fiducial markers (radio-opaque contrast agent, lipiodol). UK PIs who completed outlining benchmark cases for the preceding phase II adaptive bladder radiotherapy trial (HYBRID Trial, NCT01810757) will be asked to contour only the target volumes as the OARs contouring is unchanged for the RAIDER protocol [31]. Structured feedback to the PI will be provided via RTTQA team.

All participating trial centres will also be required to complete a planning benchmark case. Centres will be provided with access to CT DICOM data and pre-outlined structure set. They will be requested to the plan this patient in their own treatment planning system as if randomised to the DART arm. It is the responsibility of the local investigator to ensure that appropriate plan checking QA process is in place at their local institution. Once the three plans of the benchmark case have been created, reviewed, and accepted by the local PI, the DICOM CT, dose cubes, RTplan, and structure sets are returned to the RTTQA team and structured feedback is provided.

It is a pre-trial requirement that all participating centres have both an established IGRT training programme in place for their radiographers and be utilising CBCT to assess bladder treatment delivery. Trial specific bladder IGRT competency will be completed through an online plan selection training package, and practical workshop [32].

The online plan selection training consists of two practice cases each with 6 CBCTs to work through. Step by step instructions with correct plan selections is provided. Following this, a credentialing assessment consisting of 12 plan selections will be carried out. The plan selections and matched reviews will be assessed by RTTQA and structured feedback provided. Only those who meet minimum threshold of concordance of plan selection as pre-defined by the trial team will be approved for performing RAIDER plan selection. Those who were accredited for plan selection in

For peer review only - http://bmjopen.bmj.com/site/about/guidelines.xhtml

the HYBRID study [31] or in the TROG 10.1 BOLART trial training (NCT01142102) [33] will not be asked to repeat this assessment.

As part of the on-trial QA, the contouring and planning of at least the first adaptive patient and the first DART will be subject to prospective review by the RTTQA group.

All planning data and treatment delivery data including paired weekly pre- and post treatment CBCTs, registration objects, and treatment forms will be collected and reviewed retrospectively by the RTTQA group to ensure adherence to the RAIDER planning and delivery protocol is maintained. Remote retrospective plan selection review will take place for adaptive radiotherapy patients during the trial.

Statistical considerations

The primary aim of the study is to evaluate the feasibility (stage I) and safety (stage II) of DART. Control (WBRT) and SART treatment groups are included to enable SART to be carried forward to stage II if dose constraints cannot be met in the DART group and to assess equipoise and feasibility of recruitment for any subsequent phase III trial Prospectively collected contemporaneous toxicity data for WBRT and SART will also allow benchmarking of DART results. Patients are randomised 1:1:2 to maximise information on DART. Recruitment to stage II will continue seamlessly whilst stage I is evaluated, unless advised otherwise by the Independent Data Monitoring Committee (IDMC). Patients recruited in stage I will contribute to analysis of stage II.

The sample size of stage I is based on proportion of patients allocated to DART meeting the pre-defined dose constraints of bladder, bowel, and rectum on the medium plan. A patient in the 32f cohort will be defined as meeting the dose constraints if all mandatory constraints of the following are met for the medium plan: rectum constraints at 50Gy, 60Gy, 65Gy and 70Gy; bladder outside PTV2 at 60Gy and 65Gy; and small bowel at V55, V60, V65, V70 and V74. A patient in the 20f cohort will be defined as meeting the dose constraints if all mandatory constraints of the following are met for the medium plan: rectum constraints at 41.7Gy, 50Gy, 54.2Gy and 58.3Gy; bladder outside PTV2 at 50Gy and 54.2Gy; and bowel at V45.8, V50, V54.2, V58.3 and V61.7.

It is expected that in 80% of DART patients the pre-defined dose constraints of the

For peer review only - http://bmjopen.bmj.com/site/about/guidelines.xhtml

medium plan to the normal bladder, bowel, and rectum will be met. If this proportion is less than 50% it will be concluded that DART delivery is not feasible. Using an A'Hern single stage design (p0=0.5, p1=0.8, 5% alpha, and 80% power) 18 patients are required in each DART fractionation cohort. If at least 13/18 meet dose constraints it will be concluded that DART treatment is feasible; if dose constraints are not met for 6 or more patients in either fractionation cohort the IDMC will advise on continuation of the trial with the option of dropping the DART arm in one or both fractionation cohorts and continuing to stage II with randomization to WBRT versus SART. Stage I will therefore require a total of 72 patients (36 in each fractionation cohort) randomised 1:1:2 between WBRT, SART and DART.

There are no formal early stopping rules for acute toxicity or efficacy but if after 6 patients have been treated per fractionation cohort, more than 50% of patients experience acute ≥grade 3 treatment related toxicity, the IDMC would be asked to advise on suitability of continuation.

Stage II has a non-comparative design aiming to rule out an upper limit of any late ≥G3 CTCAE toxicity in each DART fractionation cohort. To be considered evaluable for the primary endpoint of late toxicity a patient must receive at least one fraction of allocated treatment and have at least one toxicity assessment performed between 6 and 18 months after completing radiotherapy. It is expected that the proportion of patients in the control group reporting ≥grade 3 toxicity CTCAE toxicity between 6-18 months post-radiotherapy will be 8% [20]. Again using an A'Hern single stage design (p0 (toxicity free)=0.80, p1=0.92, 5% alpha, and 80% power), 57 patients in each DART fractionation cohort, will allow a >20% ≥grade 3 toxicity CTCAE toxicity to be excluded. If more than 6/57 evaluable DART patients experience ≥grade 3 toxicity in either fractionation cohort, then the late toxicity threshold will be exceeded and on the IDMC's recommendation the trial could either be stopped or the DART arm dropped.

Allowing for 5% non-evaluability for late toxicity by 18 months gives a sample size of 120 patients (30 WBRT, 30 SART, 60 DART) for each fractionation cohort i.e. a total target sample size of 240. The non-evaluability rate will be monitored and, with IDMC endorsement, cohort recruitment will continue until there are 57 evaluable DART patients per cohort. During stage II, following IDMC review, consideration would be given to dropping the WBRT or SART arms, if it was felt sufficient data had accrued for these arms and it would expedite meeting the aims of the trial. The IDMC will also monitor recurrence rates. If an absolute excess of loco-regional recurrence

For peer review only - http://bmjopen.bmj.com/site/about/guidelines.xhtml

are seen early termination of the trial would be considered.

For stage I, the primary endpoint will be presented as the frequency and percentage of randomised patients able to meet the trial dose constraints in the DART group. For stage II, the primary endpoint will be based on the evaluable population. The proportion of patients with any ≥grade 3 CTCAE toxicity occurring within 6 to 18 months post radiotherapy will be presented for each randomised treatment group together with the 90% one-sided binomial confidence interval (the 90% two-sided confidence interval will also be presented). A sensitivity analysis will be conducted using a per protocol population. The per protocol population will include evaluable patients who received their complete fractionation schedule (either 32f or 20f) according to their randomised allocation group.

The local control rate at 2 years will be presented by treatment group with a 95% confidence interval. Acute and late toxicity will be summarised by frequencies and proportions at each time point by treatment group. Kaplan-Meier methods will also be used to analyse time to local disease progression and overall survival with data presented by randomised group.

Ethics and dissemination

The trial is approved by the London-Surrey Borders Research Ethics Committee, 15/LO/0539).

Safety reporting

Data is collected at each trial visit regarding any adverse events graded according to CTCAE v4 criteria on the CRF. The highest grade observed since the last visit should be reported. All serious adverse events (SAEs) occurring from the start of radiotherapy up to 30 days following the last fraction and any radiotherapy related ≥grade 3 events occurring between 6 and 18 months are reported to the ICR-CTSU within 24 hours of the PI becoming aware of the event. SAEs should be followed up until clinical recovery is complete or until the condition has stabilised. Any safety concerns will be reported to the main REC by ICR-CTSU as part of the annual progress report.

Trial monitoring and oversight

A Trial Management Group (TMG) will be set up and will include the Chief

For peer review only - http://bmjopen.bmj.com/site/about/guidelines.xhtml

Investigator, ICR-CTSU Methodology Lead, co-investigators, identified collaborators, the trial statistician, trial manager, and patient representative.

The ICR-CTSU Urology Radiotherapy Trials Steering Committee (TSC) includes a chairperson not directly involved in the trial, and at least two other independent members who will oversee the RAIDER trial. The TSC will meet annually.

An IDMC will be set up to monitor the progress of the trial and will include at least three independent members, one of whom will be a medical statistician. The Committee's terms of reference, roles and responsibilities will be defined in a charter issued by ICR-CTSU. The IDMC will meet in confidence at regular intervals, and at least annually. A summary of findings and any recommendations will be produced following each meeting. This summary will be submitted to the TMG and TSC, and if required, the main REC.

Trial status and dissemination of results

The first participant was enrolled in October 2015. The study recruitment is expected to complete in Spring 2020. It is expected that the trial will report in 2022.

Patient and public involvement

The RAIDER trial has been reviewed and endorsed by patient and carer representatives from the National Cancer Research Institute (NCRI) Consumer Liaison Group and the NCRI Clinical and Translational Radiotherapy Research Group (CTRad) working group. The CTRad consumer group also approved the proposal for randomisation ratio to be weighted towards participants receiving advanced radiotherapy techniques.

Patient and public involvement began at the protocol design and development stage via national and local consumer oversight committee review. This included the NIHR Biomedical Research Centre radiotherapy studies consumer panel at The Institute of Cancer Research and The Royal Marsden NHS Foundation Trust, and the National Cancer Research Institute (NCRI) Bladder Clinical Studies Group, which includes consumer representation.

For peer review only - http://bmjopen.bmj.com/site/about/guidelines.xhtml

Patients who had participated in the phase I bladder radiotherapy studies [25, 28] were asked to assess if the burden of involvement required for participation was appropriate. This included review of the patient reported outcomes questionnaires.

The trial patient information sheet and consent form were reviewed by the South West London Cancer Research Network consumer group. Their feedback was adopted and incorporated in to the final version of both documents. Copy of the ethics approved final version of the patient information sheet and consent form are provided in the supplementary files (Appendix 3).

Patient representation on the Trial Management Group advises on day-to-day management of the trial including patient recruitment, and it is expected that they will also participate in dissemination of results via bladder cancer patient groups.

Conclusions

RAIDER represents the first randomised trial of dose escalated adaptive tumour focused ‘plan of the day’ radiotherapy and provides a framework for multi-centre implementation of this technique. It seeks to investigate whether this approach will allow an increase of radiation dose to be delivered to the tumour with acceptable toxicity. Results will inform the design of a future phase III trial to establish the optimum organ preserving treatment option for patients with MIBC.

For peer review only

For peer review only - http://bmjopen.bmj.com/site/about/guidelines.xhtml

Figures

Figure 1. Trial schema

Tables

Table 1. Schedule of assessments

Table 2. PTV expansion details

Table 3. Prescription doses

Table 4. Target volume dose objectives

Table 5. Organ at risk dose constraint guide

Supplementary information

Appendix 1. RAIDER trial protocol (Version 3.0; dated 23rd January 2019)

Appendix 2. RAIDER radiotherapy planning and delivery guidelines (Version 4.3, dated 27th January 2020)

Appendix 3. RAIDER patient information sheet and consent form (Version 2.0; dated 27th January 2019)

For peer review only

Table 1. Schedule of assessments

Visit/Assessment	Pre-neoadjuvant chemotherapy (if given)	Pre-randomisation†	Pre-radiotherapy	On treatment	6 weeks after start RT¥	10 weeks after start RT	3 months after end RT	6 months after end RT	9 months after end RT	12 months after end RT	18 months after end RT	24 months after end RT	Annually to 5 years	Annually thereafter	At recurrence/ disease progression
Radiological assessment*	X	X												Disease status and survival	Treatment according to local practice, follow up for disease status and survival
TURBT with completion of bladder map	X	X													
Placement of fiducial markers (optional)	X	X	X[1]												
Assessment of symptoms/toxicity			X[2]	X[2,3]	X[2]	X[2]	X[2]	X[6]	X[6]	X[6]	X[6]	X[6]	X[6]		
Full blood count, urea and electrolytes			X	X[4]			X	X							
PRO questionnaire (if participating)			X	X[5]			X	X[7]		X[7]	X[7]	X[7]			
Rigid cystoscopy and biopsy of tumour bed							X								
Chest x-ray							X								
Flexible cystoscopy								X	X	X	X	X	X		
CT of abdomen and pelvis								X		X		X			
Chest x-ray or CT chest								X		X	X	X	X		
Health resource utilisation				X			X	X		X	X	X			

Footnotes
* Recommended imaging: MRI pelvis, CT chest and abdomen. Minimum acceptable is chest, abdomen, pelvis CT or CT chest and CT urogram † For patients who have not received neo-adjuvant chemotherapy
¥ For patients in the 20f cohort only
1. For patients who received neo-adjuvant chemotherapy
2. CTCAE v.4
3. Weekly on treatment
4. During weeks 1, 4 and 6 (week 6 only if receiving 32f)
5. At last fraction
6. CTCAE v.4 and RTOG
7. Questionnaires administered to UK participants by ICR-CTSU from 6 months onwards

For peer review only

For peer review only - http://bmjopen.bmj.com/site/about/guidelines.xhtml

Table 2. PTV expansion details

Patient Randomisation	CT data set	PTV	CTV to PTV expansion (cm)					PTV2	GTV to PTV2 expansion (cm)				
			Laterally	Anteriorly	Posteriorly	Superiorly	Inferiorly		Laterally	Anteriorly	Posteriorly	Superiorly	Inferiorly
**Group 1 Standard Whole Bladder (WBRT)**	CT0	PTV	0.8	1.5	1.2	1.5	0.8	Not applicable					
**Group 2 and 3 Adaptive Tumour Focused (SART and DART)**	CT30	PTV_Sm	0.5	0.5	0.5	0.5	0.5	PTV2_Sm	0.5	0.5	0.5	0.5	0.5
	CT30	PTV_Med	0.5	1.5	1.0	1.5	0.5	PTV2_Med	0.5	1.5	1.0	1.5	0.5
	If CT60-CT30 bladder filling <50mls then apply												
	CT30	PTV_Lar_30	0.8	2.0	1.2	2.5	0.8	PTV2_Lar_30	0.8	2.0	1.2	2.5	0.8
	If CT60-CT30 bladder filling >50mls then apply												
	CT60	PTV_Lar_60	0.5	1.5	1.0	1.5	0.5	PTV2_Lar_60	0.5	1.5	1.0	1.5	0.5

For peer review only - http://bmjopen.bmj.com/site/about/guidelines.xhtml

Table 3. Prescription doses

Patient Randomisation	Volume	32 fraction cohort		20 fraction cohort	
		Dose (Gy)	Dose per fraction (Gy)	Dose (Gy)	Dose per fraction (Gy)
**Group 1** **WBRT**	PTV_Std	64	2	55	2.75
**Group 2** **SART**	PTV2	64	2	55	2.75
	PTV (PTV – PTV2)	52	1.625	46	2.3
**Group 3** **DART**	PTV2	70	2.1875	60	3
	PTV (PTV – PTV2)	52	1.625	46	2.3

Table 4. Target volume dose objectives

Volume	Dose Constraints	Optimal	Mandatory
PTV2	$D_{98\%}$	≥95% of prescribed dose	≥90% of prescribed dose
	*$D_{50\%}$	-	+/- 1% of prescribed dose
	$D_{2\%}$	≤105% of prescribed dose	≤107% of prescribed dose
PTV (PTV – PTV2)	D98%	≥95% of prescribed dose	≥90% of prescribed dose

*Please note that D50% constraint refers only to PTV2. PTV D50% is likely to be exceeded depending on size of PTV2. Therefore no compromise to PTV2 coverage should be made at the expense of achieving D50% PTV constraint.

For peer review only - http://bmjopen.bmj.com/site/about/guidelines.xhtml

Table 5. Organ at risk dose constraint guide

Normal tissue	32 fraction cohort			20 fraction cohort		
	Constraint	Optimal	Mandatory	Constraint	Optimal	Mandatory
**Rectum**	V30Gy<br>V50Gy<br>V60Gy<br>V65Gy<br>V70Gy		80%<br>60%<br>50%<br>30%<br>15%	V25Gy<br>V41.7Gy<br>V50Gy<br>V54.2Gy<br>V58.3Gy		80%<br>60%<br>50%<br>30%<br>15%
**Femoral Heads**	V50Gy		50%	V41.7Gy		50%
**Other Bowel**	V45Gy<br>V50Gy<br>V55Gy<br>V60Gy<br>V65Gy<br>V70Gy<br>V74Gy	116cc<br>104cc<br>91cc<br>73cc<br>23cc<br>0cc<br>0cc	139cc<br>127cc<br>115cc<br>98cc<br>40cc<br>10cc<br>0cc	V37.5Gy<br>V41.7Gy<br>V45.8Gy<br>V50Gy<br>V54.2Gy<br>V58.3Gy<br>V61.7Gy	116cc<br>104cc<br>91cc<br>73cc<br>23cc<br>0cc<br>0cc	139cc<br>127cc<br>115cc<br>98cc<br>40cc<br>10cc<br>0cc
****Whole bladder constraint (i.e. CTV)**	V60Gy<br>V65Gy	50%<br>40% only in DART<br>Otherwise 0% in SART	80%<br>50% only in DART<br>Otherwise 5% SART	V50Gy<br>V54.2Gy	50%<br>40% only in DART<br>Otherwise 0% in SART	80%<br>50% only in DART<br>Otherwise 5% SART
**Body-PTV (Normal Tissue)**	D_{1cc}	≤105% of prescribed dose	≤110% of prescribed dose	D_{1cc}	≤105% of prescribed dose	≤110% of prescribed dose

**Whole bladder (CTV) constraint specified should be used to inform plan optimisation. Bladder outside PTV2 (i.e. CTV-PTV2) meeting these contraints will also be collected for reporting of the primary endpoint.

For peer review only - http://bmjopen.bmj.com/site/about/guidelines.xhtml
For peer review only

Contribution

All authors meet at least of one the criteria recommended by the ICMJE. RH and EH conceived the study design. SH wrote the first draft of the radiotherapy protocol and manuscript. All authors were involved in protocol development and contributed to subsequent revisions of the protocol and manuscript.

Competing interest

SH reports non-financial support from Elekta (Elekta AB, Stockholm, Sweden), non-financial support from Merck Sharp & Dohme (MSD), personal fees and non-financial support from Roche outside the submitted work; AC reports grants from National Institute of Health Research Manchester Biomedical Research Centre, grants from Cancer Research, UK, grants from Medical Research Council, UK, grants from Prostate Cancer, UK, grants from Bayer, UK, personal fees from Janssen Pharmaceutical, non-financial support from ASCO, grants and non-financial support from Elekta AB, outside the submitted work; AH reports grants from CRUK, grants from MRC, grants from NIHR, outside the submitted work; TK reports reports grants from Cancer Australia , during the conduct of the study; and his group has a research collaborative agreement with Varian Medical System not related to this project; EH reports grants from Cancer Research UK during the conduct of the study; grants from Accuray Inc., grants from Varian Medical Systems Inc., outside the submitted work; RH reports non-financial support from Janssen, grants and personal fees from MSD, personal fees from Bristol Myers Squibb, grants from Cancer Research UK, other from Nektar Therapeutics, personal fees and non-financial support from Roche outside the submitted work. AW, KWO, VNH, HM, EP, JC, FF, DM, AM, HM, DS, EM, CG, and RL have no conflicts to disclose.

Funding

The RAIDER trial is funded by Cancer Research UK (CRUK/14/016) with programme grants to support the work of the Clinical Trials and Statistics Unit at The Institute of Cancer Research (ICR-CTSU) (grant number grant number C1491/A15955).

Acknowledgements

SH, KW, HMcN, RL, EH, and RH acknowledge this study represents independent research supported by the National Institute for Health Research (NIHR) Biomedical Research Centre at The Royal Marsden NHS Foundation Trust and The Institute of Cancer Research, London. AC is supported by the NIHR Manchester Biomedical Research Centre. The views expressed are those of the author(s) and not

For peer review only - http://bmjopen.bmj.com/site/about/guidelines.xhtml

necessarily those of the NIHR or the Department of Health and Social Care. The trial was supported by the National Radiotherapy Trials Quality Assurance Team (RTTQA).

Data sharing

No additional data are available as submission relates to trial protocol only.

For peer review only

References

1. **National Institute for Health and Clinical Excellence (NICE) Guidance. Bladder cancer: diagnosis and management** http://wwwniceorguk/guidance/ng2/evidence/full-guideline-3744109 2015.
2. James ND, Hussain SA, Hall E, Jenkins P, Tremlett J, Rawlings C, Crundwell M, Sizer B, Sreenivasan T, Hendron C *et al*: **Radiotherapy with or without chemotherapy in muscle-invasive bladder cancer**. *N Engl J Med* 2012, **366**(16):1477-1488.
3. Hoskin PJ, Rojas AM, Bentzen SM, Saunders MI: **Radiotherapy with concurrent carbogen and nicotinamide in bladder carcinoma**. *J Clin Oncol* 2010, **28**(33):4912-4918.
4. Bellmunt J, Orsola A, Leow JJ, Wiegel T, De Santis M, Horwich A, Group EGW: **Bladder cancer: ESMO Practice Guidelines for diagnosis, treatment and follow-up**. *Ann Oncol* 2014, **25 Suppl 3**(suppl 3):iii40-48.
5. Witjes JA, Lebret T, Comperat EM, Cowan NC, De Santis M, Bruins HM, Hernandez V, Espinos EL, Dunn J, Rouanne M *et al*: **Updated 2016 EAU Guidelines on Muscle-invasive and Metastatic Bladder Cancer**. *European Urology* 2017, **71**(3):462-475.
6. Hafeez S, Horwich A, Omar O, Mohammed K, Thompson A, Kumar P, Khoo V, Van As N, Eeles R, Dearnaley D *et al*: **Selective organ preservation with neo-adjuvant chemotherapy for the treatment of muscle invasive transitional cell carcinoma of the bladder**. *Br J Cancer* 2015, **112**(10):1626-1635.
7. Zietman AL, Grocela J, Zehr E, Kaufman DS, Young RH, Althausen AF, Heney NM, Shipley WU: **Selective bladder conservation using transurethral resection, chemotherapy, and radiation: management and consequences of Ta, T1, and Tis recurrence within the retained bladder**. *Urology* 2001, **58**(3):380-385.
8. Majewski W, Maciejewski B, Majewski S, Suwinski R, Miszczyk L, Tarnawski R: **Clinical radiobiology of stage T2-T3 bladder cancer**. *Int J Radiat Oncol Biol Phys* 2004, **60**(1):60-70.
9. Steel G: **Basic Clinical Radiobiology.** *Oxford University Press,* 3rd edition; 2002.
10. Pos FJ, Hart G, Schneider C, Sminia P: **Radical radiotherapy for invasive bladder cancer: What dose and fractionation schedule to choose?** *Int J Radiat Oncol Biol Phys* 2006, **64**(4):1168-1173.
11. Fokdal L, Honore H, Hoyer M, Meldgaard P, Fode K, von der Maase H: **Impact of changes in bladder and rectal filling volume on organ motion and dose distribution of the bladder in radiotherapy for urinary bladder cancer**. *Int J Radiat Oncol Biol Phys* 2004, **59**(2):436-444.
12. Lotz HT, Pos FJ, Hulshof MC, van Herk M, Lebesque JV, Duppen JC, Remeijer P: **Tumor motion and deformation during external radiotherapy of bladder cancer**. *Int J Radiat Oncol Biol Phys* 2006, **64**(5):1551-1558.
13. Meijer GJ, Rasch C, Remeijer P, Lebesque JV: **Three-dimensional analysis of delineation errors, setup errors, and organ motion during radiotherapy of bladder cancer**. *Int J Radiat Oncol Biol Phys* 2003, **55**(5):1277-1287.
14. Lalondrelle S, Huddart R, Warren-Oseni K, Hansen VN, McNair H, Thomas K, Dearnaley D, Horwich A, Khoo V: **Adaptive-predictive organ localization using cone-beam computed tomography for improved accuracy in external beam radiotherapy for bladder cancer**. *Int J Radiat Oncol Biol Phys* 2011, **79**(3):705-712.
15. **National Radiotherapy Implementation Group Report Image Guided Radiotherapy (IGRT) Guidance for implementation and use.**

For peer review only - http://bmjopen.bmj.com/site/about/guidelines.xhtml

https://wwwsororg/sites/default/files/document-versions/National%20Radiotherapy%20Implementation%20Group%20Report%20IGRT%20Finalpdf August 2012.

16. McDonald F, Lalondrelle S, Taylor H, Warren-Oseni K, Khoo V, McNair HA, Harris V, Hafeez S, Hansen VN, Thomas K *et al*: **Clinical implementation of adaptive hypofractionated bladder radiotherapy for improvement in normal tissue irradiation**. *Clin Oncol (R Coll Radiol)* 2013, **25**(9):549-556.
17. Vestergaard A, Muren LP, Lindberg H, Jakobsen KL, Petersen JB, Elstrom UV, Agerbaek M, Hoyer M: **Normal tissue sparing in a phase II trial on daily adaptive plan selection in radiotherapy for urinary bladder cancer**. *Acta Oncol* 2014, **53**(8):997-1004.
18. Foroudi F, Wong J, Kron T, Rolfo A, Haworth A, Roxby P, Thomas J, Herschtal A, Pham D, Williams S *et al*: **Online adaptive radiotherapy for muscle-invasive bladder cancer: results of a pilot study**. *Int J Radiat Oncol Biol Phys* 2011, **81**(3):765-771.
19. Tuomikoski L, Collan J, Keyrilainen J, Visapaa H, Saarilahti K, Tenhunen M: **Adaptive radiotherapy in muscle invasive urinary bladder cancer - An effective method to reduce the irradiated bowel volume**. *Radiother Oncol* 2011.
20. Huddart RA, Hall E, Hussain SA, Jenkins P, Rawlings C, Tremlett J, Crundwell M, Adab FA, Sheehan D, Syndikus I *et al*: **Randomized noninferiority trial of reduced high-dose volume versus standard volume radiation therapy for muscle-invasive bladder cancer: results of the BC2001 trial (CRUK/01/004)**. *Int J Radiat Oncol Biol Phys* 2013, **87**(2):261-269.
21. Cowan RA, McBain CA, Ryder WD, Wylie JP, Logue JP, Turner SL, Van der Voet J, Collins CD, Khoo VS, Read GR: **Radiotherapy for muscle-invasive carcinoma of the bladder: results of a randomized trial comparing conventional whole bladder with dose-escalated partial bladder radiotherapy**. *Int J Radiat Oncol Biol Phys* 2004, **59**(1):197-207.
22. Caffo O, Thompson C, De Santis M, Kragelj B, Hamstra DA, Azria D, Fellin G, Pappagallo GL, Galligioni E, Choudhury A: **Concurrent gemcitabine and radiotherapy for the treatment of muscle-invasive bladder cancer: A pooled individual data analysis of eight phase I-II trials**. *Radiother Oncol* 2016, **121**(2):193-198.
23. Coppin CM, Gospodarowicz MK, James K, Tannock IF, Zee B, Carson J, Pater J, Sullivan LD: **Improved local control of invasive bladder cancer by concurrent cisplatin and preoperative or definitive radiation. The National Cancer Institute of Canada Clinical Trials Group**. *J Clin Oncol* 1996, **14**(11):2901-2907.
24. Measurements ICoRUa: **ICRU Report 83 Prescribing, Recording, and Reporting Intensity-Modulated Photon-Beam Therapy (IMRT)**. 2010.
25. Hafeez S, McDonald F, Lalondrelle S, McNair H, Warren-Oseni K, Jones K, Harris V, Taylor H, Khoo V, Thomas K *et al*: **Clinical Outcomes of Image Guided Adaptive Hypofractionated Weekly Radiation Therapy for Bladder Cancer in Patients Unsuitable for Radical Treatment**. *Int J Radiat Oncol Biol Phys* 2017, **98**(1):115-122.
26. Dearnaley D, Syndikus I, Sumo G, Bidmead M, Bloomfield D, Clark C, Gao A, Hassan S, Horwich A, Huddart R *et al*: **Conventional versus hypofractionated high-dose intensity-modulated radiotherapy for prostate cancer: preliminary safety results from the CHHiP randomised controlled trial**. *Lancet Oncol* 2012, **13**(1):43-54.
27. McDonald F, Waters R, Gulliford S, Hall E, James N, Huddart RA: **Defining bowel dose volume constraints for bladder radiotherapy treatment planning**. *Clin Oncol (R Coll Radiol)* 2015, **27**(1):22-29.

For peer review only - http://bmjopen.bmj.com/site/about/guidelines.xhtml

28. Hafeez S, Warren-Oseni K, McNair HA, Hansen VN, Jones K, Tan M, Khan A, Harris V, McDonald F, Lalondrelle S *et al*: **Prospective Study Delivering Simultaneous Integrated High-dose Tumor Boost (≤70 Gy) With Image Guided Adaptive Radiation Therapy for Radical Treatment of Localized Muscle-Invasive Bladder Cancer**. *International Journal of Radiation Oncology*Biology*Physics* 2016, **94**(5):1022-1030.
29. Dale RG: **The application of the linear-quadratic dose-effect equation to fractionated and protracted radiotherapy**. *Br J Radiol* 1985, **58**(690):515-528.
30. Bese NS, Hendry J, Jeremic B: **Effects of prolongation of overall treatment time due to unplanned interruptions during radiotherapy of different tumor sites and practical methods for compensation**. *Int J Radiat Oncol Biol Phys* 2007, **68**(3):654-661.
31. Hafeez S PE, Webster A, Warren-Oseni K, Hansen V, McNair H, Miles E, Lewis R, Hall E, Huddart R: **Protocol for hypofractionated adaptive radiotherapy to the bladder within a multicentre phase II randomised trial: radiotherapy planning and delivery guidance**. *BMJ open* 2020, http://dx.doi.org/10.1136/bmjopen-2020-037134.
32. Patel E, Tsang Y, Baker A, Callender J, Hafeez S, Hall E, Hansen VN, Lewis R, McNair H, Miles E *et al*: **Quality assuring "Plan of the day" selection in a multicentre adaptive bladder trial: Implementation of a pre-accrual IGRT guidance and assessment module**. *Clinical and Translational Radiation Oncology* 2019, **19**:27-32.
33. Foroudi F, Pham D, Rolfo A, Bressel M, Tang CI, Tan A, Turner S, Hruby G, Williams S, Hayne D *et al*: **The outcome of a multi-centre feasibility study of online adaptive radiotherapy for muscle-invasive bladder cancer TROG 10.01 BOLART**. *Radiother Oncol* 2014, **111**(2):316-320.

For peer review only

Figure 1. Trial schema

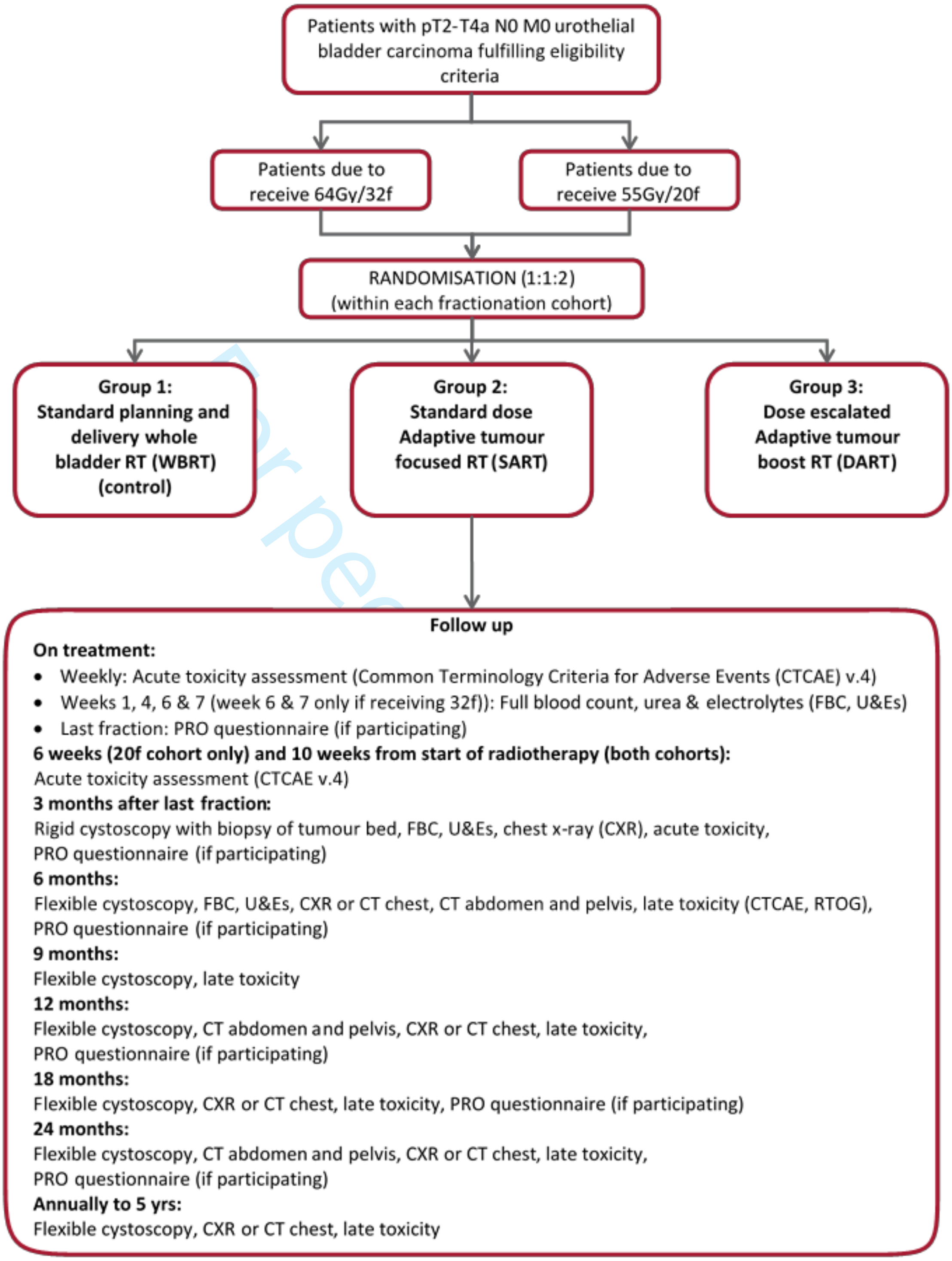

For peer review only - http://bmjopen.bmj.com/site/about/guidelines.xhtml

A **R**andomised phase II trial of **A**daptive **I**mage guided standard or **D**ose **E**scalated tumour boost **R**adiotherapy in the treatment of transitional cell carcinoma of the bladder

RADIOTHERAPY PLANNING AND DELIVERY GUIDELINES

Version:4.3

Dated:13/02/2020

Chief Investigator:	Prof Robert Huddart
Sponsor:	The Institute of Cancer Research
Approval:	Cancer Research UK: Clinical Trials Awards& Advisory Committee (CTAAC)
Funders:	Cancer Research UK
Coordinating Trials Unit:	ICR Clinical Trials and Statistics Unit (ICR-CTSU)<br>The Institute of Cancer Research

Main REC Reference Number:	15/LO/0539
ISRCTN:	26779187
ClinicalTrials.gov Identifier	NCT02447549
CRUK Reference Number:	CRUK/14/016

The RAIDER trial has been scientifically approved
by Cancer Research UK's Clinical Trials Awards & Advisory Committee (CTAAC)
The RAIDER trial is part of the National Institute for
Health Research Clinical Research Network Trial Portfolio

This is a controlled document which should be referred to in conjunction with the RAIDER protocol and should not be copied, distributed or reproduced without the written permission of the ICR-CTSU. This document should only be used for the management of patients in the RAIDER trial.

For peer review only

RAIDER radiotherapy planning and delivery guidelines

This document sets out guidelines for treatment of patients within the RAIDER trial and should be referred to in combination with the protocol.

This document should only be used for the purpose of the RAIDER trial.

The Trial Management Group reserves the right to amend or add to the radiotherapy guidelines as appropriate. Such changes do not constitute an amendment, and revised guidelines will be circulated to participating centres as needed. Changes between versions will be noted prior to the introduction. Sites treating RAIDER patients for the first time are advised to contact ICR-CTSU to confirm they have the most recent version.

Any questions relating to the detail of the radiotherapy planning and delivery guidelines should be addressed in the first instance to the QA team (See Appendices A& B).

For peer review only

CONTACT DETAILS

Scientific Coordination

Chief Investigator
Prof Robert Huddart
The Royal Marsden NHS Foundation Trust,
The Institute of Cancer Research,
London,
United Kingdom

Tel: 0208 661 3457
Email: Robert.Huddart@icr.ac.uk

Clinical Coordinator
Dr Shaista Hafeez
The Royal Marsden NHS Foundation Trust,
The Institute of Cancer Research
London
United Kingdom

Tel: 0208 661 3274
Email: Shaista.Hafeez@icr.ac.uk

TROG lead
Prof Farshad Foroudi
Director Radiation Oncology
Austin Health
Melbourne
Australia

Email: farshad.foroudi@austin.org.au

Trial Management

ICR Clinical Trials & Statistics Unit
Division of Clinical Studies,
The Institute of Cancer Research,
Sir Richard Doll Building, Cotswold Road,
Sutton, Surrey SM2 5NG

RAIDER Trial Manager
Tel: 0208 722 4613
Email: RAIDER-icrctsu@icr.ac.uk

Quality Assurance

Radiotherapy Trials and Quality Assurance Group:
Amanda Webster
Mount Vernon Cancer Centre,
Rickmansworth Road,
United Kingdom

Tel: 02038262320
Email: raiderqa.enh-tr@nhs.net

For peer review only

For peer review only - http://bmjopen.bmj.com/site/about/guidelines.xhtml

CONTENTS

1 INTRODUCTION 12
2 PLANNING CT SCAN FOR RADIOTHERAPY 13
2.1 Patient preparation 13
2.1.1 Group 1, 2 and 3: General Preparation 13
2.1.2 Group 1: Empty Bladder 13
2.1.3 Group 2 and 3: Bladder Filling 13
2.2 Patient positioning 13
2.3 Planning CT acquisition, scan limits and slice thickness 13
2.4 Planning CT export, fusion and evaluation of bladder filling 13
3 LOCALISATION OF THE TARGET VOLUME AND ORGANS AT RISK 15
3.1 Volume definition 15
3.2 Gross tumour volume 15
3.3 Clinical target volume 15
3.4 Organs at risk 15
3.5 Planning target volume 16
3.5.1 Group 1: standard whole bladder RT 16
3.5.2 Group 2 and 3: adaptive tumour focused RT (standard dose and dose escalated) 16
Table 1: CTV to PTV Expansion Details 17
Table 2: GTV to PTV2 Expansion details (group 2 and 3 only) 18
3.6 Voluming Checklist 18
3.7 Nomenclature 22
Table 3: RAIDER Trial Target Volume and OAR Nomenclature 22
4 PLANNING 24
4.1 Standard Planning 24
4.2 Adaptive Tumour Focused Planning 24
Table 4: Prescription doses 25
4.3 Target volume dose objectives 25
Table 5: Target dose objectives 25
4.4 Normal tissue dose constraints 26
Table 6a: OAR Dose Constraints for 32-fraction schedule 26
Table 6b: OAR Dose constraints for 20-fraction schedule 27
4.5 Planning recommendations 28
4.6 Pre-Treatment checks 28
5 TREATMENT DELIVERY 29
5.1 Standard whole bladder RT (group 1) 29

For peer review only - http://bmjopen.bmj.com/site/about/guidelines.xhtml

5.2 Adaptive Tumour Focused RT (SART and DART) 29
5.2.1 Image Match and Plan Selection Steps: 29
5.2.2 Image Match and Plan Selection Tips: 30
5.3 Post treatment 31
5.4 On completion of radiotherapy 31
6 TREATMENT SCHEDULING AND GAPS 31
APPENDIX A: 33
United Kingdom RAIDER QA Programme 33
APPENDIX B: 36
Trans Tasman Radiation Oncology Group: Australia & NZ 36
APPENDIX C: 40
PRE-TRIAL OUTLINING BENCHMARK CASE 40
Outlining Benchmark Case 1: T2N0M0 Male Pelvis 40
Outlining Benchmark Case 2: T2N0M0 Female Pelvis 42
PRE-TRIAL PLANNING BENCHMARK CASE 43
Benchmark Planning Case: T2N0M0 TCC male pelvis 43
APPENDIX D 45
APPENDIX E 46
APPENDIX F 47
APPENDIX G 52
APPENDIX H 53
APPENDIX I 54

For peer review only - http://bmjopen.bmj.com/site/about/guidelines.xhtml

VERSION CHANGES

V1.0 - Original RT planning and delivery guidelines

V2.0 – RT planning and delivery guidelines updated to include TROG QA appendix and contact details.

V3.0- The following changes have been made:

- Section 2.4 (Pg 11) and Section 5 (Pg 20)- Clarification provided on what CT30 and CT60 are to be used for
- Section 5 Table 4 & 5 (Pg 21/22)- Clarification regarding which target volumes the dose objectives are for
- Section5: Table 5 (Pg 22)- D50% +/- 1% for PTV2 moved from "Optimal" to "Mandatory"
- Section 5.5 (Pg 24/25)- Planning recommendations added based on pre-trial QA exercise
- Section 7.4 (Pg 28)- Recommendations for exporting CBCT data from Aria/Mosaiq
- Appendix A: QA Programme UK – Pre-trial point 7: IGRT training video now provided
- Appendix B: TROG QA Programme- QA programme updated with IGRT training/assessment information

V4.0- The following changes have been made:

- Section 1 (Pg 10) Clarification on the start date of radiotherapy
- Section 2.3 (Pg 11) Clarification on contrast. Contrast is permitted; however it is recommended to perform the planning CT scan without it
- Section 3.5 (Pg 14) Clarification on treatment volumes for each randomisation group
- Section 3.6 (Pg 16) NEW voluming checklist with clarification on outlining the GTV and CTV
- Section 3.7 (Pg 20) Nomenclature table updated
- Section 4 (Pg 22) Clarification on actions to take when replanning required
- Section 4.3 (Pg 2) Clarification on target dose objectives, with emphasis placed on PTV2
- Section 4.4 (Pg 24) Clarification on normal tissue dose constraints
- Section 4.4 (Pg 24/25) Table 6a and 6b updated to include new optimisation targets for the CTV
- Section 4.5 (Pg 25) Planning recommendations updated, with emphasis placed on PTV2
- Section 4.6 (Pg 26) Further clarification and guidance for pre-treatment checks
- Section 5 (Pg 27) Treatment delivery updated
- Section 5.2 (Pg 27/28) NEW guidance on Adaptive Tumour Focused Radiotherapy (SART & DART)
- Section 5.2.1 (Pg 27/28) NEW plan selection steps
- Sections 5.2.2 (Pg 28) NEW plan selection tips
- Section 5.3 (Pg 28) Clarification on post-treatment CBCTs
- Appendix A: QA Programme UK
    - On trial QA updated – site visits removed and IGRT/POD selection support introduced
    - Clarification on data export
    - NEW Table 7: RAIDER data checklist
- Appendix B: TROG QA Programme
    - On trial QA updated to include IGRT/POD selection support
- Appendix C: QA Benchmark Cases for Outlining and Planning moved to Appendix C
    - Additional diagnostic information for outlining case 1 included (NEW)
- Appendix D: Quick Contouring Checklist (NEW)
- Appendix E: Clarification on treatment interventions
- Appendix F: Text updated to be in line with Section 5.2
- Appendix G: Quick Reference Guide for Exporting CBCTs from ARIA (NEW)
- Appendix H: Quick Reference Guide for Exporting CBCTs from MOSAIQ (NEW)

V4.1- The following changes have been made:

- Section 4.4: Clarification on updated (V4.0) Normal Tissue Dose Constraints

V4.2 - The following changes have been made:

- Front cover updated (Pg 1/2) RAIDER Guidelines only to be used for the purpose of RAIDER

For peer review only - http://bmjopen.bmj.com/site/about/guidelines.xhtml

- Trial Management Details (Pg 3) ICR-CTSU contact details upated
- RAIDER TRIAL SUMMARY (Pg 7) Recuitment Target updated
- Trial Schema (Pg 9) Updated
- Section 1 (Pg 10) A list of RAIDER QA credentialed staff must retained in site investigator file
- Section 4.4 (Pg 24) Clarification do not compromise dose to PTV2 or PTV in order to meet the normal tissue dose constraints
- Section 5.2.1 (Pg 27-28) Clarification on Image Match and Plan Selection Steps:
    - Consider magnitude of soft tissue shift and review if over 1cm
- Section 5.2.2 (Pg 28-29) Clarification on Image Match and Plan Selection Tips:
    - Fractions must not be omitted or missed due to unfavourable positioning of normal anatomy
    - Review post-treatment CBCTs for intrafraction filling
- Section 6 (Pg 29-30) Clarification on Treatment Scheduling and Gaps:
    - Avoid gaps where possible
    - Further guidance when staff unavailable/machine breakdown
    - Compensation not expected due to toxicity
    - Involved PI
- Appendix G (Pg 50) Additional information for Aria Export

V4.3 - The following changes have been made:

- Appendix A (Pg 33) Data transfer details updated for RTTQA
- Appendix B (Pg 37) Data export and upload updated for TROG
- Appendix G (Pg 51) Please contact RTTQA for information on suitable anonymization software for CBCTs from ARIA
- Appendix I (Pg 53) Please contact RTTQA for information when exporting CBCTs from Raystation

For peer review only - http://bmjopen.bmj.com/site/about/guidelines.xhtml

RAIDER TRIAL SUMMARY

PROTOCOL TITLE	A Randomised phase II trial of Adaptive Image guided standard or Dose Escalated tumour boost Radiotherapy in the treatment of transitional cell carcinoma of the bladder
TARGET DISEASE	Muscle invasive bladder cancer
STUDY OBJECTIVES	To define a feasible and safe adaptive dose escalated tumour boost radiotherapy schedule for MIBC; to investigate the ability to deliver daily adaptive bladder radiotherapy and assess the impact of delivery on patient reported outcomes and health economic related measures.
STUDY DESIGN	Multicentre two stage, three arm phase II randomised controlled trial
TRIAL POPULATION	Patients receiving radical radiotherapy for muscle invasive bladder cancer
RECRUITMENT TARGET	Minimum 120 in each of two fractionation cohorts i.e. sufficient to accrue 57 evaluable DART patients per cohort
TRIAL TREATMENT	Patients will be randomised (1:1:2) between:<br>1. Standard whole bladder radiotherapy delivery (WBRT) (control)<br>2. Standard dose Adaptive tumour focused radiotherapy (SART)<br>3. Dose escalated Adaptive tumour boost radiotherapy (DART)<br>64Gy/32f and 55Gy/20f fractionation schedules are permitted. Participants in all groups will be permitted to receive concomitant radio sensitising chemotherapy. Full blood count (FBC), urea and electrolytes (U&Es) and acute toxicity will be assessed during radiotherapy. Participants in the Patient Reported Outcomes (PRO) sub-study will be asked to complete questionnaire prior to trial entry and at the end of radiotherapy.
PRIMARY ENDPOINT	Stage I: Proportion of patients meeting radiotherapy dose constraints to bladder, bowel and rectum in DART groups.<br>Stage II: Proportion of patients experiencing any ≥Grade 3 Common Terminology Criteria for Adverse Events (CTCAE) v.4 late toxicity (6-18 months post radiotherapy).
SECONDARY ENDPOINTS	Stage I:<br>• Recruitment rate<br>• Ability to deliver SART and DART<br>Stage II:<br>• Clinician reported acute toxicity<br>• PRO: acute and late bladder and bowel/rectal symptoms;

For peer review only - http://bmjopen.bmj.com/site/about/guidelines.xhtml

	• Health economic related measures: time for outlining, plan generation, selection and delivery, NHS resource usage subsequent to treatment;<br>• Loco-regional MIBC control<br>• Progression-free survival<br>• Overall survival
EXPLORATORY ENDPOINTS	Image Guided Radiotherapy (IGRT) endpoints:<br>• Use of adaptive plans<br>• Target coverage<br>• Online/offline concordance<br>• Dose volume analysis of adaptive vs. standard planning
FOLLOW UP	Participants will subsequently be assessed at the following intervals:<br>**6 weeks from start of radiotherapy (20f cohort only)**<br>Assessment of acute toxicity (CTCAE v.4)<br>**10 weeks from start of radiotherapy:**<br>Assessment of acute toxicity (CTCAE v.4)<br>**3 months from end of radiotherapy:**<br>Rigid cystoscopy and biopsy of tumour bed, FBC, U&Es, chest x-ray (CXR), acute toxicity (CTCAE), PRO questionnaire (if participating in sub-study).<br>**6 months from end of radiotherapy:**<br>Flexible cystoscopy, FBC, U&Es, CXR or CT chest, CT abdomen and pelvis, late toxicity (CTCAE, RTOG), PRO(if participating in sub-study)<br>**9months from end of radiotherapy:**<br>Flexible cystoscopy, late toxicity<br>**12months from end of radiotherapy:**<br>Flexible cystoscopy, CT abdomen and pelvis, CXR or CT chest, late toxicity, PRO(if participating in sub-study)<br>**18months from end of radiotherapy:**<br>Flexible cystoscopy, CXR or CT chest, late toxicity, PRO(if participating in sub-study)<br>**24 months from end of radiotherapy:**<br>Flexible cystoscopy, CT abdomen and pelvis, CXR or CT chest, late toxicity, PRO(if participating in sub-study)<br>**Yearly to year 5:** Flexible cystoscopy, CXR or CT chest, late toxicity

For peer review only

For peer review only - http://bmjopen.bmj.com/site/about/guidelines.xhtml

Annually thereafter: Survival and disease status

For peer review only

For peer review only - http://bmjopen.bmj.com/site/about/guidelines.xhtml

RAIDER radiotherapy planning and delivery guidelines

Patients with pT2-T4a N0 M0 urothelial bladder carcinoma fulfilling eligibility criteria

Patients due to receive **64Gy/32f**

Patients due to receive **55Gy/20f**

RANDOMISATION 1:1:2

Group 1: Standard planning and delivery whole bladder RT (WBRT) (control)

Group 2: Standard Dose Adaptive Tumour Focused RT (SART)

Group 3: Dose Escalated Adaptive Tumour Focused RT (DART)

Follow up

On treatment:

- Weekly: Acute toxicity assessment (Common Terminology Criteria for Adverse Events (CTCAE) v.4)
- Weeks 1, 4, 6 & 7 (week 6 & & only if receiving 32f)): Full blood count, urea a& electrolytes (FBC, U&Es)
- Last fraction: PRO questionnaire (if participating)

6 weeks (20f cohort only) and 10 weeks from start of radiotherapy (both cohorts):
Acute toxicity assessment (CTCAE v.4)

3 months after last fraction:
Rigid cystoscopy with biopsy of tumour bed, FBC, U&Es, chest x-ray (CXR), acute toxicity, PRO questionnaire (if participating)

6 months:
Flexible cystoscopy, FBC, U&Es, CXR or CT chest, CT abdomen and pelvis, late toxicity, PRO questionnaire (if participating)

9 months:
Flexible cystoscopy, late toxicity

12 months:
Flexible cystoscopy, CT abdomen and pelvis, CXR or CT chest, late toxicity, PRO questionnaire (if participating)

18 months:
Flexible cystoscopy, CXR or CT chest, late toxicity, PRO questionnaire (if participating)

24 months:
Flexible cystoscopy, CT abdomen and pelvis, Chest X-ray or CT chest, late toxicity, PRO questionnaire (if participating)

Annually to 5 yrs:
Flexible cystoscopy, CXR or CT chest, late toxicity

For peer review only - http://bmjopen.bmj.com/site/about/guidelines.xhtml

RAIDER radiotherapy planning and delivery guidelines

1 INTRODUCTION

All patients will be planned to receive radical daily radiotherapy delivered in 32 or 20 fractions. Patients will be randomised between 3 groups;

1. Standard Whole Bladder Radiotherapy (WBRT) (control)
2. Standard dose Adaptive tumour focused Radiotherapy (SART)
3. Dose escalated Adaptive tumour boost Radiotherapy (DART)

In all 3 groups concomitant therapy is permitted at the local investigators discretion.

Radiotherapy should ideally commence within 6 weeks following randomisation. However up to 10 weeks is permitted, to allow sufficient time for chemotherapy and/or radiotherapy planning

Participants allocated to standard planning and delivery (control arm, ***group 1***) will have:

- One (1) radiotherapy plan generated to deliver all fractions on an empty bladder.

The IGRT process is as detailed in the NRIG IGRT 2012 report for this patient group. The NRIG IGRT report contains detailed guidelines for IGRT practice in UK. (It is in the RAIDER trial documents on the TROG website for reference).

Participants allocated to adaptive planning (***group 2 and 3***) will have:

- Three (3) radiotherapy plans generated (small, medium and large).
- A simultaneous integrated boost delivered at conventional dose (group 2) or dose escalation (group 3) to the bladder tumour in a single phase IMRT technique.
- All fractions treated on a partially filled bladder.
- A cone beam CT (MV or kV) taken prior to each treatment delivery to select the most appropriate 'plan of the day' depending on the bladder volume size.

A comprehensive QA programme will be implemented for the RAIDER trial. This will include pre-accrual and during accrual components. Selection of appropriate treatment plans for the adaptive planning group will be independently monitored as part of the on-going RTQA process.

Plan selection is authorised to be carried out only by site personnel who have attained concordance with the gold standard for PTV selection through either RTTQA IGRT QA credentialing for UK centres and TROG IGRT credentialing for Australia/NZ. This is to ensure they have the advanced level skills required for the study. A record of QA credentialed staff should be retained in the site investigator file.

This document should only be used for the management of patients in the RAIDER trial.

For peer review only - http://bmjopen.bmj.com/site/about/guidelines.xhtml

2 PLANNING CT SCAN FOR RADIOTHERAPY

2.1 Patient preparation

2.1.1 Group 1, 2 and 3: General Preparation

Ideally all patients should be encouraged to empty the rectum of flatus and faeces. The routine use of micro enemas (e.g. relaxit) is permissible if it is standard local practice.

Bladder preparation is dependent on patient randomisation.

2.1.2 Group 1: Empty Bladder

Ensure patient has an empty bladder. Therefore all patients should be asked to void immediately before planning CT scan (CT0) is performed and not to drink fluids for 30minutes before the scan.

2.1.3 Group 2 and 3: Bladder Filling

30 minutes prior to the planning CT scan, patients are instructed to empty their bladder and then drink 350ml of water. Two planning scans will be acquired to inform pattern of bladder filling over time:

- The first scan will be at 30 minutes (CT30) following drinking
- The second scan will be at 60 minutes (CT60) following drinking.

Voiding is not permitted between the 2 scans; however if unavoidable, the CT30 scan should be used for planning.

2.2 Patient positioning

All patients will be scanned and treated supine with arms displaced out of the radiotherapy field, using appropriate immobilisation techniques.

2.3 Planning CT acquisition, scan limits and slice thickness

Planning CT scans will be performed in the treatment position at CT slice thickness 3mm or less. It is recommended that the planning CT is performed without contrast, but the use of contrast is permitted if it is local clinical practice. Recommended scanning levels are at least 4cm above the dome of the bladder to 2cm below ischial tuberosities.

For patients treated in group 2 and 3, the planning CT scan is performed (as above) at 'Time=30 minutes' (scan 1, CT30). A second planning CT is acquired at 'Time= 60 minutes' (scan 2, CT60) from end of drinking. The time of each scan is recorded.

2.4 Planning CT export, fusion and evaluation of bladder filling

The planning CT scan is exported via DICOM transfer to the treatment planning system for localization.

For patients treated in group 2 and 3 the bladder filling must be assessed. In order to do so fuse both planning CT scans in the treatment planning system. <u>CT30 will be the primary data set</u> and CT60 will be the fused secondary dataset. To evaluate bladder filling, the whole bladder is localized on the CT30 and on the CT60 scan.

For peer review only - http://bmjopen.bmj.com/site/about/guidelines.xhtml

- If the difference in bladder filling between the 2 planning scans is less than 50ml i.e. no significant filling occurs all contours are to be created on CT30.
- If difference is greater than 50mls i.e. filling occurs, the large GTV and CTV will be created on CT60.These shall be used to create PTV2 and PTV for the large plan

Please note all planning and dose calculation is to be done on CT30 (See section 4), therefore all volumes should be assigned to a single structure set on the CT30 scan.

For peer review only

For peer review only - http://bmjopen.bmj.com/site/about/guidelines.xhtml

3 LOCALISATION OF THE TARGET VOLUME AND ORGANS AT RISK

3.1 Volume definition

Volumes will be defined according to the International Commission on Radiation Units and Measurements (ICRU) report 50, supplement report ICRU 62: Prescribing, Recording and Reporting Photon Beam Therapy and ICRU 83: Prescribing, Recording and Reporting Photon-Beam Intensity Modulated Radiotherapy (IMRT). Outlining should be carried out with the aid of all diagnostic information including position of fiducial markers, surgical bladder map, MRI and CT scans.

3.2 Gross tumour volume

The gross tumour volume (GTV) is defined as the bladder tumour/bed. It will be delineated using position of fiducial markers (where available), diagnostic imaging (imaging prior to neoadjuvant chemotherapy where applicable) and the surgical bladder map (where available). When delineating the tumour any extravesical tumour should be included in GTV as should pathological bladder wall thickening unless clearly not due to tumour. If no tumour is visible, the appropriate section of the bladder should be included based on surgical bladder map +/- discussion with urologist who performed TURBT. In these circumstances consider repeating TURBT and placing fiducial markers adjacent to resection scar whenever possible.

For patients treated in group 2 and 3, if the difference in bladder filling between CT30 and CT60 is greater than 50ml i.e. filling occurs, GTV is to be contoured on both scans.

3.3 Clinical target volume

The clinical target volume (CTV) is the contour encompassing the tumour/bed (GTV), the whole bladder and any area of extravesical spread. The CTV should also include 1.5cm of prostatic urethra in males or 1cm of urethra in females if tumour is at the base of bladder or if distant CIS is present.

For patients treated in group 2 and 3, if the difference in bladder filling between CT30 and CT60 is greater than 50ml i.e. filling occurs, CTV is to be contoured on both scans.

3.4 Organs at risk

Organs at risk (OAR) will be outlined as solid structures by defining their outer wall. All OAR should be outlined on the CT0 for group 1 and CT30 only for group 2 and 3. The following OARs should be contoured:

- Rectum:

  The rectum is outlined to include the full circumference and rectal contents. Outlining should extend from the lowest level of ischial tuberosities to the recto-sigmoid junction. The recto-sigmoid junction will be defined as the level at which there is an anterior inflection of the bowel, best appreciated on sagittal reconstructions on the CT planning scan.

For peer review only - http://bmjopen.bmj.com/site/about/guidelines.xhtml

- Other bowel:

The small and large bowel (including sigmoid colon) will be outlined as a single structure. The entire small and large bowel visible on relevant levels of the planning scan will be outlined as individual bowel loops. The superior extent of outlining should be 2cm beyond the superior extent of PTV (group 1) and Large PTV (group 2 and 3).

- Femoral heads:

Both the femoral heads are outlined to the bottom of the curvature of their heads (femoral necks are not included).

3.5 Planning target volume

The CTV(s) will be expanded, as below, to create the PTV(s).

For the tumour focused RT (group 2 and 3) the GTV(s) will be expanded, as below to create the PTV2s.

3.5.1 Group 1: standard whole bladder RT

For patients in group 1, receiving standard whole bladder RT, a single PTV will be created.

1. PTV= CTV with anisotropic margin applied, as per Table 1.

3.5.2 Group 2 and 3: adaptive tumour focused RT (standard dose and dose escalated)

For patients in group 2 and 3, receiving adaptive tumour focused RT a library of 3 PTVs will be created, from the CTV(s) (Table 1):

1. PTV_Sm

2. PTV_Med

3. PTV_Lar

- For patients in group 2 and 3, if the difference in bladder filling between CT30 and CT60 is less than 50ml i.e. filling does not occur, the PTV_Sm, PTV_Med and PTV_Lar will be produced from the 30 minute CTV.

- However, if the difference in bladder filling between CT30 and CT60 is greater than 50ml i.e. filling occurs, the PTV_Sm and PTV_Med will be produced from the 30 minute CTV. The PTV_Lar will be produced from the 60 minute CTV, as described in Table 1.

For peer review only - http://bmjopen.bmj.com/site/about/guidelines.xhtml

Table 1: CTV to PTV Expansion Details

Patient Randomisation	CT data set	PTV	CTV to PTV Expansion (cm)				
			Laterally	Anteriorly	Posteriorly	Superiorly	Inferiorly
Group 1 Standard Whole Bladder	CT0	PTV	0.8	1.5	1.2	1.5	0.8
Group 2 and 3 Adaptive Tumour Focused	CT30	PTV_Sm	0.5	0.5	0.5	0.5	0.5
	CT30	PTV_Med	0.5	1.5	1.0	1.5	0.5
	If CT60-CT30 <50mls then						
	CT30	PTV_Lar_30	0.8	2.0	1.2	2.5	0.8
	If CT60-CT30>50mls then						
	CT60	PTV_Lar_60	0.5	1.5	1.0	1.5	0.5

Additionally 3 PTV2s will also be created, from the GTV(s) (Table 2):

1. PTV2_Sm
2. PTV2_Med
3. PTV2_Lar

- For patients in group 2 and 3, if the difference in bladder filling between CT30 and CT60 is less than 50ml i.e. filling does not occur, the PTV2_Sm, PTV2_Med and PTV2_Lar will be produced from the 30 minute GTV.
- However, if the difference in bladder filling between CT30 and CT60 is greater than 50ml i.e. filling occurs, the PTV2_Sm and PTV2_Med will be produced from the 30 minute GTV. The PTV2_Lar will be produced from the 60 minute GTV, as described in Table 2.

For peer review only

For peer review only - http://bmjopen.bmj.com/site/about/guidelines.xhtml

RAIDER radiotherapy planning and delivery guidelines

Table 2: GTV to PTV2 Expansion details (group 2 and 3 only)

Patient Randomisation	CT data set	PTV2	GTV to PTV2 Expansion (cm)				
			Laterally	Anteriorly	Posteriorly	Superiorly	Inferiorly
Group 1<br>Standard Whole Bladder	Not applicable						
Group 2 and 3<br>Adaptive Tumour Focused	CT30	PTV2_Sm	0.5	0.5	0.5	0.5	0.5
	CT30	PTV2_Med	0.5	1.5	1.0	1.5	0.5
	If CT60-CT30 <50mls then						
	CT30	PTV2_Lar_30	0.8	2.0	1.2	2.5	0.8
	If CT60-CT30>50mls then						
	CT60	PTV2_Lar_60	0.5	1.5	1.0	1.5	0.5

3.6 Voluming Checklist

1. For group 2 and 3, the inferior border of the CTV should be the same on both the CT30 and CT60 following fusion, Figure 1.

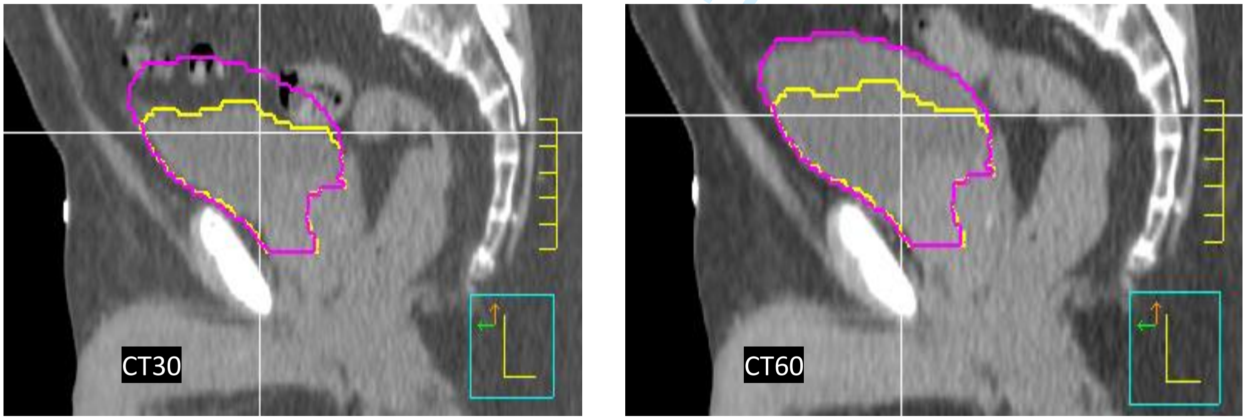

Figure 1: Inferior border of CTV anticipated to be at same level for both scans. Can be achieved by copying inferior contour on fused scan. This ensures that changes in volume are due to filling and not to variation/contouring error between the 2 scans.

For peer review only - http://bmjopen.bmj.com/site/about/guidelines.xhtml

2. For Groups 1, 2 and 3 please ensure that 1.5cm of prostatic urethra in males or 1cm of urethra in females is included when the tumour is at the bladder base or if distant CIS is present. Please contact RTTQA if further guidance is required on this aspect of outlining.

3. For Group 2 and 3, the CTV drawn on CT60 should not be within/smaller than the CTV drawn on the CT30. Thus, the CTV drawn on CT60 should encompass the CTV drawn on CT30. This could occur as a result of contouring error, however it is also possible that bowel motion/filling between the 2 scans may also cause this (see Figures 2a-2d). In these circumstances it is advised that the CTV drawn on CT60 is summed to include the CTV drawn on CT30; allowing all excursions of the bladder to be included in the volume. This can be done by application of Boolean operators within the treatment planning system used.

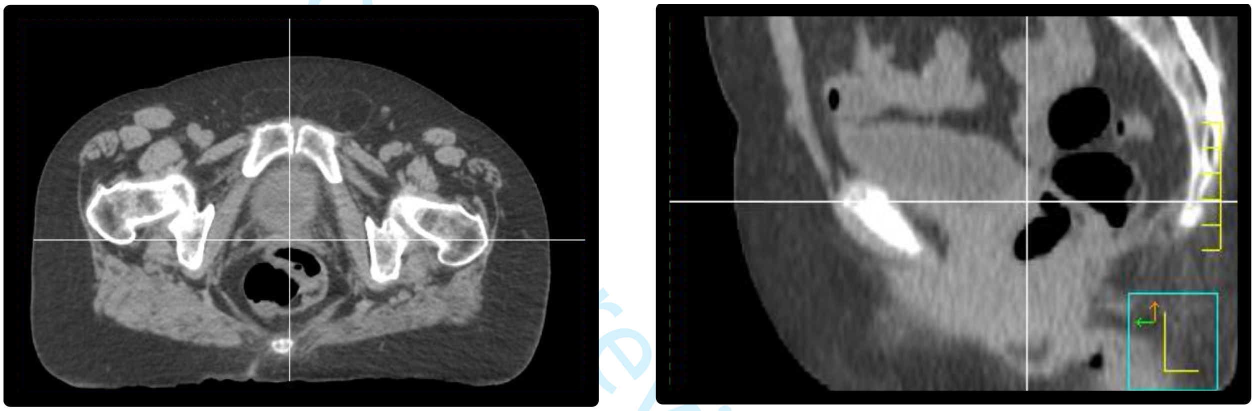

Figure 2a: CT30 scan.

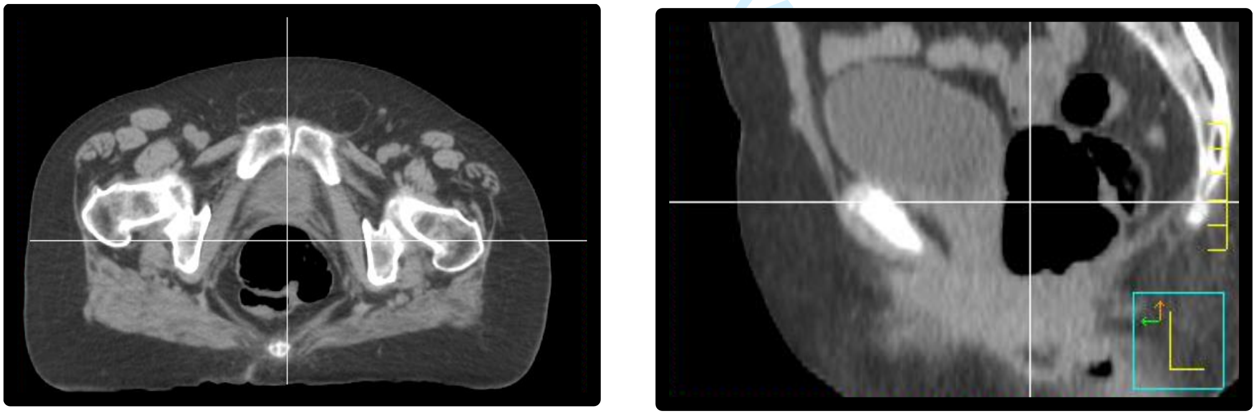

Figure 2b: CT60 scan. Bladder filling occurs but is also pushed anterior due to rectal distension secondary to flatus

For peer review only - http://bmjopen.bmj.com/site/about/guidelines.xhtml

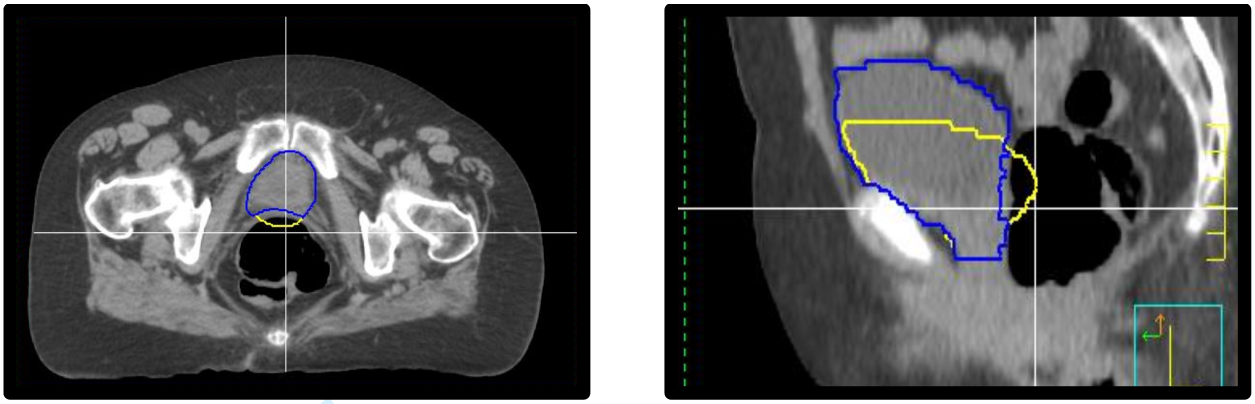

Figure 2c: Yellow contour reflects CTV on CT30. Blue contour reflects CTV on CT60. (Difference between two volumes >50cc).

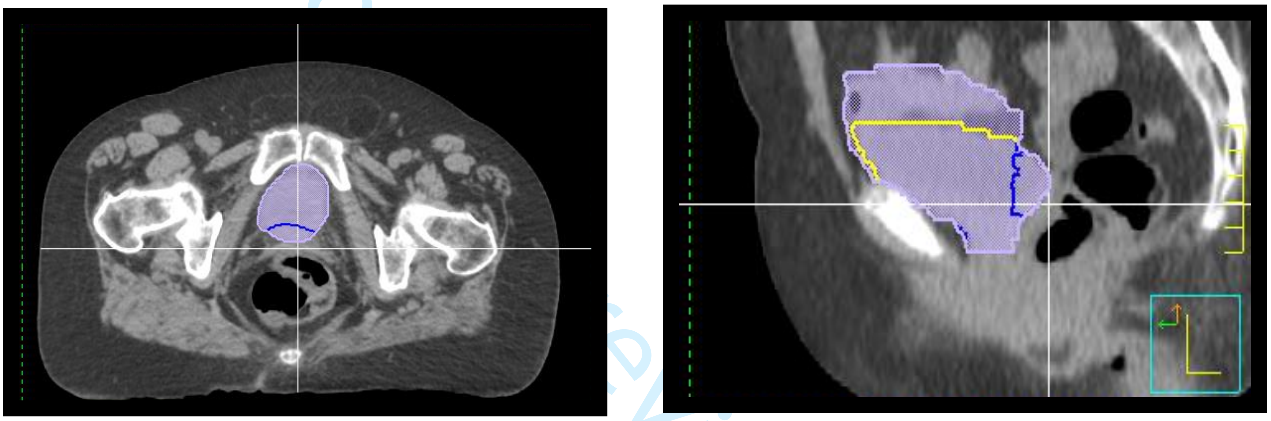

Figure 2d: Boolean operator applied to create summed CTV (purple) from which PTV_Lar_60 (and PTV2_Lar_60) will be created.

4. The GTV should not exceed beyond the CTV outline. Where there is extra-vesical spread included in the GTV, the CTV must be extended outside the bladder wall to include all the GTV. The CTV should be summed to include all the GTV and should share same outer-contour, see Figure 3a-3c.

- The CTV_30 should encompass the GTV_30 and share the same outer contour.
- The CTV_60 should encompass the GTV_60 (and thus the GTV_30) and should share the same outer contour.

For peer review only - http://bmjopen.bmj.com/site/about/guidelines.xhtml

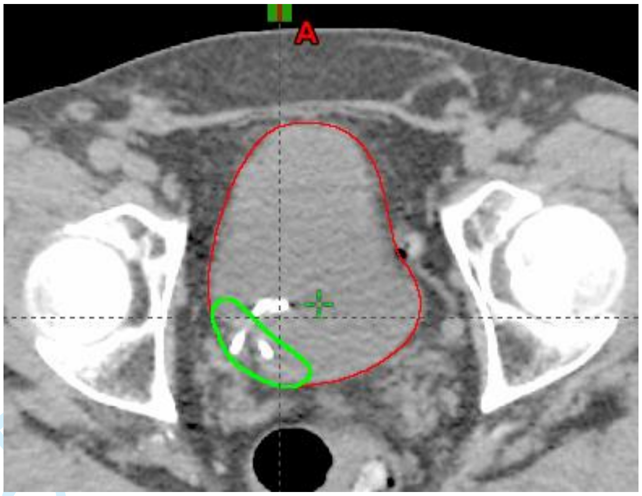

Figure 3a: CT30 GTV (green) shares same bladder wall as CTV (red)

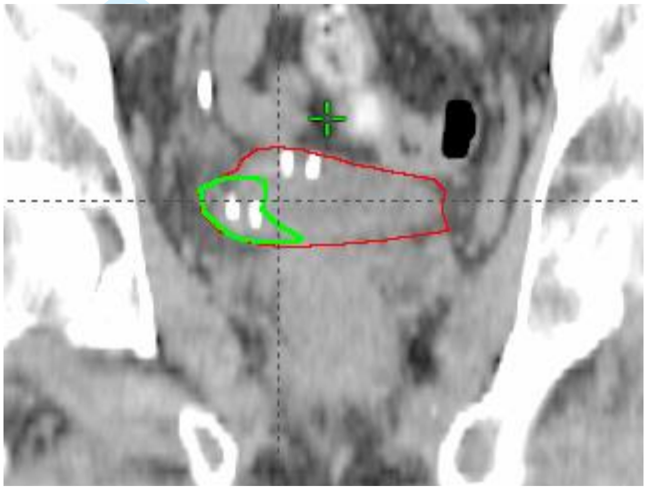

Figure 3b: CT30 GTV (green) shares same bladder wall as CTV (red)

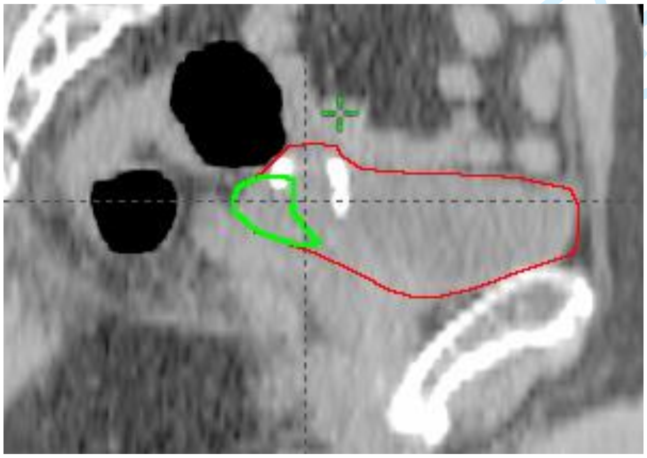

Figure 3c: CT30 GTV (green) shares same bladder wall as CTV (red)

For peer review only - http://bmjopen.bmj.com/site/about/guidelines.xhtml

5. The GTV_60 should generally extend further superiorly and anteriorly, in comparison with the GTV_30, to account for expansion upon filling.

6. The PTV_Lar should encompass the PTV_Med.

7. The PTV2 should not exceed beyond the corresponding PTV outline. For planning purposes, the PTV2 should share the same outer contour as the corresponding PTV outline, i.e. it should 'appear' attached to the outer contour of the PTV.

8. The clinician should indicate to the planner which CT data set (CT30 or CT60) has been used to create the large target volumes.

9. The OTHER_BOWEL should extend to 2cm beyond the largest PTV.

10. The rectum should extend to the lowest level of the ischial tuberosities.

A "Quick Contouring Checklist" has been provided in Appendix E to ensure the above has been successfully achieved.

3.7 Nomenclature

Consistent naming of contoured structures used in radiotherapy treatment planning is essential to facilitate the comparison of dose-volume statistics across patients for quality assurance and outcomes analysis. Maintaining consistency in structure names is particularly important (and challenging) in multi-institutional clinical trials, in which treatment planning data are collected from many participating institutions. A scheme for uniform naming of contoured structures for RAIDER is provided in the following table. The following names must be used for treatment planning of all trial patients.

Table 3: RAIDER Trial Target Volume and OAR Nomenclature

Structure Name	Description
GTV_0	Contouring the primary bladder tumour for patients randomised to Group 1 (WBRT) is not required for trial purposes. Local standard practice should be followed if contouring GTV aids subsequent CTV delineation (on CT_0)
GTV_30	Primary bladder tumour contoured on CT30 for group 2 and 3

For peer review only - http://bmjopen.bmj.com/site/about/guidelines.xhtml

For peer review only

GTV_60	Primary bladder tumour contoured on CT60 for group 2 and 3 if the difference in bladder filling between CT30 and CT60 is greater than 50ml
CTV_0	GTV + whole bladder + extravesical spread (+ 1.5cm of prostatic urethra in males or 1cm of urethra in females if tumour is at the base of bladder or if distant CIS is present) contoured on CT0 for group 1
CTV_30	GTV + whole bladder + extravesical spread (+ 1.5cm of prostatic urethra in males or 1cm of urethra in females if tumour is at the base of bladder or if distant CIS is present) contoured on CT30 for group 2 and 3
CTV_60	GTV + whole bladder + extravesical spread (+ 1.5cm of prostatic urethra in males or 1cm of urethra in females if tumour is at the base of bladder or if distant CIS is present) contoured on CT60 for group 2 and 3
PTV_Std	CTV_0 + anisotropic margin (see table 1)<br>**For Standard Whole Bladder-group 1 only**
PTV_Sm	CTV_30 + margin
PTV_Med	CTV _30 + margins
PTV_Lar_30	CTV_30 +margins, **when no filling occurs between CT30 and CT60)**
PTV_Lar_60	CTV_60 +margins, **when filling occurs between CT30 and CT60)**
PTV2_Sm	GTV_30 + margin
PTV2_Med	GTV_30 +margins
PTV2_Lar_30	GTV_30 + margins, **when no filling occurs between CT30 and CT60)**
PTV2_Lar_60	GTV_60 + 0.5 – 1.5cm margins, **when filling occurs between CT30 and CT60)**
PTV_Sm-PTV2_Sm	PTV_Sm minus PTV2_Sm
PTV_Med-PTV2_Med	PTV_Med minus PTV2_Med
PTV_Lar-PTV2_Lar	PTV_Lar minus PTV2_Lar
RECTUM	Rectum
OTHER_BOWEL	Small and large bowel (including sigmoid colon)
FEMORALJOINT_L (UK) FemJoint_L (Aus)	Left femoral head (ball only)
FEMORALJOINT_R (UK)FemJoint_R (Aus)	Right femoral head (ball only)

For peer review only - http://bmjopen.bmj.com/site/about/guidelines.xhtml

4 PLANNING

It is mandatory for all RAIDER patients to be CT planned. In general, the method of treatment planning will vary from site to site and should be specified in each centre's process document.

For group 1 the use of three or four 3D conformal radiotherapy (3DCRT) fields, five to seven static fields intensity modulated radiotherapy (IMRT) or volumetric modulated radiotherapy (VMAT) treatments (e.g. RapidARC, VMAT, Tomotherapy) are acceptable. Please inform the relevant QA group and the CTU if a group 1 patient requires re-planning (or re-scanning) during treatment.

For group 2 and 3 the aim of the plan is to do partial bladder sparing while maintaining the PTV prescription dose, use of 5-7 static fields IMRT (5 fields are preferred due to shorter treatment time) or VMAT is recommended. The use of forward planned simple IMRT ('field in field') or tomotherapy are also acceptable alternatives. The same technique should be used for all patients randomised to group 2 and 3. All plan and dose calculation is to be done on CT30 irrespective of filling.

Please inform the relevant QA group and the CTU if a group 2 or 3 patient requires re-planning (or re-scanning) during treatment.

The local investigator should ensure appropriate quality assurance methodologies are in place for the chosen planning technique.

4.1 Standard Planning

For patients randomised to group 1 standard planning a single plan will be created.

- Plan_Std = PTV_Std

4.2 Adaptive Tumour Focused Planning

For those randomised to adaptive planning a series of 3 plans will be created using the PTV Small, PTV Medium and PTV Large and the corresponding PTV2. The isocentre for all 3 plans must be identical.

- Plan_Sm = PTV_Sm& PTV2_Sm
- Plan_Med = PTV_Med& PTV2_Med
- Plan_Lar = PTV_Lar_30 and PTV2_Lar_30 or PTV_Lar_60 and PTV2_Lar_60, depending on filling.

For IMRT planning centres may prescribe plan to either a mean or median dose to PTV2, depending on their normal practice, as it is anticipated in an optimised plan the difference between these two parameters will be minimal. For 3D-CRT the prescription should be 100% at the ICRU reference point.

The prescription doses for RAIDER patients are detailed in Table 4.

For peer review only - http://bmjopen.bmj.com/site/about/guidelines.xhtml

Table 4: Prescription doses

Patient Randomisation	Volume	Dose (Gy)	Fractions	Dose per fraction (Gy)	Dose (Gy)	Fractions	Dose per fraction (Gy)
**Group 1** **Standard plan**	PTV_Std	64	32	2	55	20	2.75
**Group 2** Standard dose adaptive tumour focused **(SART)**	PTV2	64	32	2	55	20	2.75
	PTV (PTV – PTV2)	52	32	1.625	46	20	2.3
**Group 3** Dose escalated adaptive tumour boost RT (DART)	PTV2	70	32	2.1875	60	20	3
	PTV (PTV – PTV2)	52	32	1.625	46	20	2.3

4.3 Target volume dose objectives

Three dimensional dose distributions should be produced. The dose distribution should be assessed for coverage of the PTV and normal tissues using appropriate transverse sagittal and coronal views. The following optimal and mandatory target volume dose constraints are proposed:

For peer review only

Table 5: Target dose objectives

Volume	Dose Constraints	Optimal	Mandatory
PTV2	$D_{98\%}$	≥95% of prescribed dose	≥90% of prescribed dose
	*$D_{50\%}$	-	+/- 1% of prescribed dose
	$D_{2\%}$	≤105% of prescribed dose	≤107% of prescribed dose
PTV (PTV – PTV2)	D98%	≥95% of prescribed dose	≥90% of prescribed dose

For peer review only - http://bmjopen.bmj.com/site/about/guidelines.xhtml

*Please note that D50% constraint refers only to PTV2. PTV D50% is likely to be exceeded depending on size of PTV2. Therefore no compromise to PTV2 coverage should be made at the expense of achieving D50% PTV constraint.

4.4 Normal tissue dose constraints

The dose to OAR should be minimised. The following dose volume constraints are a guide.

Ideally, for Groups 2 & 3 optimal constraints for other bowel should be met for Plan 1 (small) and mandatory constraints for other bowel should be met for Plan 2 (medium). Provided the Plan 3 (large) has been adequately optimised it is recognised that some of the rectum and other bowel mandatory constraints may not be achieved.

Likewise for group 1 patients, provided the plan has been suitably optimised, some rectum and other bowel dose tolerances may be exceeded due to inclusion within the PTV. It is at the local Principal Investigator's discretion to accept the OAR doses and should be noted on the plan assessment form. The DVH assessment for each plan should be with the overall prescribed dose.

For patients in group 3, if mandatory dose constraints are not met on the medium plan advice must be sought from the RAIDER QA team.

For this reason it is recommended for adaptive planning the medium plan is produced first, this should then be copied and the relevant objectives and prescription volume are changed for the small and large plans. **Dose to the PTV2 or PTV must not be compromised in order to meet the normal tissue constraints.**

Table 6a: OAR Dose Constraints for 32-fraction schedule

**Rectum**	**V30Gy**	**80%**	
	**V50Gy**	**60%**	
	**V60Gy**	**50%**	
	**V65Gy**	**30%**	
	**V70Gy**	**15%**	
**Femoral Heads**	**V50Gy**	**50%**	
**Other Bowel**		**Optimal**	**Mandatory**
	**V45Gy**	**116cc**	**139cc**
	**V50Gy**	**104cc**	**127cc**
	**V55Gy**	**91cc**	**115cc**
	**V60Gy**	**73cc**	**98cc**
	**V65Gy**	**23cc**	**40cc**
	**V70Gy**	**0cc**	**10cc**
	**V74Gy**	**0cc**	**0cc**
****Whole bladder constraint (i.e. CTV)**		**Optimal**	**Mandatory**
	**V60Gy**	**50%**	**80%**
	**V65Gy**	**40% only in Group 3 (DART)**	**50% only in Group 3 (DART)**
		**Otherwise 0% in Group 2 (SART)**	**Otherwise 5% in Group 2 (SART)**

For peer review only - http://bmjopen.bmj.com/site/about/guidelines.xhtml

**Body-PTV (Normal Tissue)**	**D_{1cc}**	**≤105% of prescribed dose**	**≤110% of prescribed dose**

<u>Whole bladder (CTV) constraint specified above (Table 6a) should be used to **inform plan optimisation.</u> Please contact the RAIDER QA team if you fail to meet this constraint.

Bladder outside PTV2 (i.e. CTV-PTV2) meeting V60Gy and V65Gy absolute constraint of 80% and 50% respectively will be collected for reporting of primary endpoint.

Table 6b: OAR Dose constraints for 20-fraction schedule

**Rectum**	**V25Gy**	**80%**	
	**V41.7Gy**	**60%**	
	**V50Gy**	**50%**	
	**V54.2Gy**	**30%**	
	**V58.3Gy**	**15%**	
**Femoral Heads**	**V41.7Gy**	**50%**	
**Other Bowel**		**Optimal**	**Mandatory**
	**V37.5Gy**	**116cc**	**139cc**
	**V41.7Gy**	**104cc**	**127cc**
	**V45.8Gy**	**91cc**	**115cc**
	**V50Gy**	**73cc**	**98cc**
	**V54.2Gy**	**23cc**	**40cc**
	**V58.3Gy**	**0cc**	**10cc**
	**V61.7Gy**	**0cc**	**0cc**
*****Whole bladder constraint (i.e. CTV)**		**Optimal**	**Mandatory**
	**V50Gy**	**50%**	**80%**
	**V54.2Gy**	**40% only in Group 3 (DART) Otherwise 0% in Group 2 (SART)**	**50% only in Group 3 (DART) otherwise 5% in Group 2 (SART)**
**Body-PTV (Normal Tissue)**	**D_{1cc}**	**≤105% of prescribed dose**	**≤110% of prescribed dose**

***<u>Whole bladder (CTV) constraint specified above (Table 6b) should be used to **inform plan optimisation**.</u> Please contact the RAIDER QA team if you fail to meet this constraint.

Bladder outside PTV2 (i.e. CTV-PTV2) meeting V50Gy and V54.2Gy absolute constraint of 80% and 50% respectively will be collected for reporting of primary endpoint.

Normal tissue dose constraints and dose volume histograms (DVH) for 20 fraction schedule are presented in Table 6b (recalculated from above using linear quadratic equation, assuming that all α/β of organs at risk is 3 and that dose to tumour boost is delivered in 3Gy per fraction).

For peer review only - http://bmjopen.bmj.com/site/about/guidelines.xhtml

4.5 Planning recommendations

- Produce the medium plan first.

- For the planning priorities should be as follows, in order of importance:
  1. ***Achieve 95% coverage of PTV2***
  2. ***Achieve 95% coverage of the PTV***
  3. Achieve mandatory OAR dose constraints
  4. Achieve optimal OAR dose constraints
  5. Reduce areas of high dose within PTV-PTV2 especially away from boost site

Therefore, for the majority of cases dose coverage of the PTV_2 and PTV volumes should not be compromised to reduce dose to OAR without first contacting RTTQA for advice.

- For Group 2 & 3, review all three plans together after planning using the plan comparison function. The dose to the RECTUM and OTHER_BOWEL should stay the same or get progressively higher as the PTV size gets larger i.e. the dose statistics should not be better for the large plan than the small or medium plan.
- If throughout the course of treatment any patient requires re-planning please contact the relevant QA group.
- If the PTV volume is outside the patient’s body please contact the relevant QA group.

4.6 Pre-Treatment checks

To minimise risk of error at the time of importing, exporting and plan selection, please ensure that each beam name and ID reflects the assigned plan i.e. Sm_Plan.

At the time of plan exporting, it is recommended to find a way of ensuring that centres’ local record and verify systems cannot mix beams from different plans. For example create each plan with slightly different contributions from each field so that only the correct combination of beams can be chosen on any given day (Applicable only to Mosaiq). This could be done by adding 2 points diagonally on the isocentre slice with a dose close to the 100% isodose. Then all beams can be assigned from a plan to each of the points as the reference point.

Please ensure that the safe scheduling of multiple treatment fields and recording the dose delivered is considered for RAIDER patients. Additionally, please ensure that the safe scheduling of imaging only fields is considered. These procedures will be captured in the process document. If you have any questions please contact the relevant QA group.

For peer review only - http://bmjopen.bmj.com/site/about/guidelines.xhtml

5 TREATMENT DELIVERY

It is important to ensure that patients follow the bladder preparation instruction for their group as they have done at the planning CT scan appointment (i.e. empty for group 1 and partially full for group 2 and 3).

5.1 Standard whole bladder RT (group 1)

Acquire pre-treatment cone beam CT images using a full pelvis scan. Image match and register bone, according to the recommendations stated in the NRIG IGRT report.

Make required corrections/shifts as per departmental practice.

The acquired cone beam CT image can be used for assessment of target coverage at the individual department's discretion. Such use will be documented. Any changes made on the basis of the scan should be reported in the CRF and to RTTQA (including exposures not resulting in treatment because of patient factors).

Schedule imaging for Group 1 patients, as per departmental protocol. In addition to this please ensure a post-treatment CBCT is taken in the first week and weekly for these patients.

5.2 Adaptive Tumour Focused RT (SART and DART)

Acquire pre-treatment cone beam CT image and register bone according to the recommendations stated in the NRIG IGRT report. Make any corrections according to departmental practice.

An appropriately RTTQA trained and accredited healthcare professional should review the bone matched CBCT, to assess bladder filling and PTV coverage. The following steps should assist centres when assessing the CBCTs.

The aim in treating adaptive patients is to use the **smallest plan possible**, so that the dose is minimised to the OARs without compromising both PTV and PTV2 coverage.

The steps below describe how this can be best achieved at plan selection. It is important to review overall bladder filling, and PTV2 boost position as determined on the planning CT scans before plan selection.

5.2.1 Image Match and Plan Selection Steps:

1. Following CBCT, the bladder filling and size should be checked against CTV_30 contour.
2. If the bladder is of similar shape and size to the CTV at planning (i.e. CTV_30), then the small plan should be preferably considered in the first instance for treatment.
3. Once the bladder filling and shape has been assessed begin to review the appropriate PTVs. An appropriate plan provides suitable coverage of the PTV2 and PTV, with minimal normal tissue irradiation.
4. Manual (soft tissue) moves should be made to ensure the bladder is adequately covered whilst selecting the smallest plan possible to spare normal tissue. Please be mindful of the magnitude of the soft-tissue shift, moves greater than 1cm can impact on the accuracy of the expected dosimetry. Discuss with planning department if this shifts over 1cm occur and contact RTTQA retrospectively.

For peer review only - http://bmjopen.bmj.com/site/about/guidelines.xhtml

5. Additional manual moves can be undertaken if felt it could further optimise PTV2 coverage. Manual moves prioritising coverage to the boost region over the normal bladder wall is permitted if it avoids excessive normal tissue irradiation that would have occurred by selecting a larger plan. Again, please be mindful of the magnitude of the soft-tissue shift, moves greater than 1cm can impact on the accuracy of the expected dosimetry. Discuss with planning department if this shifts over 1cm occur and contact RTTQA retrospectively.
6. Assess the OAR. Review if they are similar to planning scan. Additionally it is important to check where they are relative to PTV2 i.e. ensuring not in PTV2, especially for DART patients.

The actions taken for each patient must be confirmed by a second appropriately trained and accredited healthcare professional. Once agreement has been reached any correction should be performed and the plan selection agreed and confirmed for treatment.

5.2.2 Image Match and Plan Selection Tips:

This guidance is provided to assist RAIDER trained staff with potential solutions for scenarios that may arise on treatment. Further guidance is provided in APPENDIX E. Fractions must not be omitted or missed due to unfavourable positioning of normal anatomy such as rectal distention due to flatus or faeces.

- Please only choose a large plan if the bladder has filled. (E.g. *If the bladder has filled more than the CTV_30, and the patient has a CTV_60, overlay the CTV_60 to assess the magnitude of the filling).*
- Please consider the nature of bladder filling. We would expect filling to occur in the anterior and superior direction.
- Please be mindful of minimising image match and plan selection time.
- If the bladder is significantly smaller than the CTV 30 contour at planning, it is likely that the PTV2 boost is in the incorrect position and, or does not achieve adequate normal bladder sparing. In these circumstances patient should not proceed but be removed from the couch, and encouraged to fill the bladder by drinking further, and/or increasing the time interval of image acquisition. The local PI and RTTQA/TROG should be informed.
- In the event that the bladder has over filled and none of the library PTVs cover the entire bladder despite manual move the patient should be asked to minimally void and the CBCT is repeated. If this is not possible, patient should void completely and restart drinking protocol but consider reducing the time interval for CBCT acquisition (see appendix C and D). In these circumstances a member of the clinical team should also be notified to ensure the patient is not in urinary retention. If the large plan is being regularly selected, during a single treatment week, please contact the RTTQA/TROG to discuss this patient.
- Caution should be taken when changing the drinking protocol. Please consider changing one aspect of the protocol at a time, i.e. change the timing only or change the amount of water only.
- If no contours are suitable because of rectal gas, then remove the patient from the bed and ask them to void. Repeat the above steps. If contours still not suitable select the most suitable plan, i.e. optimising coverage to PTV2 and minimising the inclusion of Oars. If this occurs repeatedly (e.g. more than twice in 5 fractions) please contact RTTQA for advice.
- All CBCT exposures including those not resulting in treatment should therefore be recorded on the CRF and plan selection form.
- The patient's hydration may be different on chemotherapy days. Care should be taken when adjusting the drinking protocol on these fractions.

For peer review only - http://bmjopen.bmj.com/site/about/guidelines.xhtml

- Please review the post-treatment CBCTs to ensure the patient's bladder has not filled beyond the plan selected. If this has occurred, then consider the length of time taken for plan selection and see section 5.3.

5.3 Post treatment

A post treatment cone beam CT should be taken in the first week and once a week thereafter (for all randomised groups, including group 1). It should be reviewed locally to ensure intra fraction filling has been accommodated in plan selection. Please discuss with the PI and contact RTTQA if there are any issues with intra-fraction filling.

5.4 On completion of radiotherapy

On completion of radiotherapy planning, all plans including CT images, structures, plan and dose matrix and plan assessment form (PAF), should be exported, anonymised and sent to the RTTQA or TROG team electronically following the exporting data guidelines in the QA appendix A&B. On completion of a patient's treatment, the plan selection form, first week and then weekly paired (pre and post radiotherapy) CBCT scans and the registration objects (Aria only) should be sent to the RTTQA or TROG team. Investigators should notify the QA team before deleting any relevant data.

Please ensure that all patient data is anonymised prior to sending it to RTTQA/TROG. If you have any difficulties anonymising the data, please contact RTTQA/TROG.

Sending data from Elekta/Mosaiq systems:

Week 1 and weekly pre and post treatment CBCT data should be exported from the XVI using "Option 3". This ensures the CBCT data is sent to the QA team in the correct treatment position.

Sending data from Varian/Aria systems:

Week 1 and weekly pre and post treatment CBCT data should be exported from Aria along with the online registration object. This ensures that the CBCT data can be reviewed in the treatment position by the QA team. Please refer to guidance in Appendix G& H.

6 TREATMENT SCHEDULING AND GAPS

Treatment interuptions during radiotherapy should be avoided as they have detrimental effect on outcome. Treatment must not be interrupted due to staffing issues. Advice should be sought from the RTTQA team and ICR-CTSU in real time if issues with patient scheduling and gaps arise.

If IGRT is unavailable due to unpredicted staff changes, machine breakdown and/or gap day treatment, patients in group 2 and 3 may be treated for up to 5 days using the PTV medium plan without plan selection. Pre-treatment CBCT where possible should still be acquired.. These pre-treatment CBCTs should be sent to RTTQA for review in addition to the CBCTs which are routinely collected.

For peer review only - http://bmjopen.bmj.com/site/about/guidelines.xhtml

In the event of machine service, breakdown, or bank holiday, compensation for the missed fraction should be made. In the first instance the local PI should advise on how this should be made, but it is expected to be achieved by either treating at a weekend or undertaking two fractions a day (ideally on a Friday with a minimum 6 hour gap between treatments). If the treatment machine is unavailable for more than 3 days, please contact the ICR-CTSU and QA team.

Should a treatment break occur due to toxicity, sites are advised to contact ICR-CTSU and/or RTTQA in real time. Compensation is not expected in these circumstance.

All missed fractions are to be reported to the ICR-CTSU and QA team.

For peer review only

For peer review only - http://bmjopen.bmj.com/site/about/guidelines.xhtml

APPENDIX A:

United Kingdom RAIDER QA Programme

Contact Details:

Contact for scientific queries	Dr Shaista Hafeez<br>Tel: Tel: 0208 661 3274<br>Email: Shaista.Hafeez@icr.ac.uk
Radiotherapy Trials Quality Assurance Team	Amanda Webster RTTQA Group<br>Tel: 0203 826 2320<br>Email:<br>raiderqa.enh-tr@nhs.net

The UK quality assurance programme for RAIDER comprises the following exercises detailed below:

Pre-Trial Quality Assurance

1. Facility questionnaire: This is designed to gauge the IGRT experience of a centre to date. It collects information regarding the type of IGRT used, action thresholds, frequency of interventions and imaging doses. This survey should be accessed online via: http://www.rttrialsqa.org.uk/rttqa/

2. Process document: Details are collected on all aspects of tasks for the complete patient pathway and includes details on all imaging procedures. A process document template can be found at http://www.rttrialsqa.org.uk/rttqa/

3. Outlining Benchmark cases: One case with lipiodol and one case without lipiodol will be provided by the RTTQA to be completed by the P.I for each recruiting site. The targets and organs at risk are to be named and delineated as per the RAIDER radiotherapy planning and delivery guidelines. Those sites that have successfully completed the outlining for another related NIHR trial will not be required to outline the organs at risk, only the target volumes. Please contact the QA team to confirm.

4. Planning Benchmark case: One planning benchmark case provided by RTTQA is to be planned according to the Group 3 randomisation arm.

5. In house IGRT training programme: It is a requirement of RAIDER that sites have an established IGRT training programme already in place before joining the trial. Sites should be utilising cone beam CT for treatment of bladder patients.

6. Bladder 'Plan of the Day' training: Two practice cases with 6 CBCTS each are provided for centres to work through with an accompanying training document. Case 1 includes answers with some step-by-step instructions, for case 2 the answers only are

For peer review only - http://bmjopen.bmj.com/site/about/guidelines.xhtml

provided. The cases can be provided on an Elekta or Varian database depending on which system is used by a site. Please contact RTTQA for the training guidance document. Those that have successfully completed the 'Plan of the Day' training for HYBRID do not need to complete this again.

7. RAIDER 'Plan of the Day' training video will be available for streaming on the RAIDER ICR website. Please contact RTTQA or ICR for access

8. RAIDER Plan of the Day assessment: Centres will be given details of two patients with 6 CBCTs each (12 match decisions) to allow individuals to make plan of the day decisions/choices. The match results will be exported to the RTTQA group for review. Those staff members that have completed the 'Plan of the Day' assessment for Hybrid will not be required to complete the assessment. All QA approved individuals will receive a confirmation of their RAIDER accreditation to undertake plan of the day assessments for RAIDER patients.

9. Verification of electronic data transfer: Check DICOM or RTOG data can be suitably anonymised and transferred to and from centres. This includes the planning data and on treatment data, i.e. CBCT and registration objects.

On-Trial Quality Assurance

1. Prospective plan review: The outlining and planning for at least the first adaptive patient and the first dose escalated patient (if the first patient is not dose escalated) will be subject to prospective review by the RTTQA group.

2. Ongoing data collection: All planning data (CT,RS, RD, RP files, PAF) and treatment delivery data (weekly pre and post treatment CBCT and registration objects (if available) will be collected by the RTTQA group for retrospective review

3. IGRT/POD selection support: The first adaptive patient randomised by all sites will be subject to a retrospective review. If centres have any difficulties in the plan selection, please do not hesitate to contact RTTQA.

Data Export

Please contact raiderqa.enh-tr@nhs.net for information on data transfer.

Please ensure that data is appropriately labelled when sent to RTTQA, i.e. trial name, randomisation number and type of data (e.g. planning/CBCTs).

For peer review only - http://bmjopen.bmj.com/site/about/guidelines.xhtml

For all patients please ensure the following data is sent for all RAIDER patients:

Table 7:

RAIDER Data Checklist	Sent
Diagnostic Information (e.g. report, screenshot of imaging)	
ALL planning CTs (e.g. include CT_30 and CT_60 for adaptive patients)	
Structure set, Dose Cube, Plan	
Plan Assessment Form	
Plan Selection Form (For all trial patients)	
Week 1 (#1-5) and weekly pre and post CBCTs	

For peer review only - http://bmjopen.bmj.com/site/about/guidelines.xhtml

APPENDIX B:

Trans Tasman Radiation Oncology Group: Australia & NZ

RAIDER Trial QA programme

Contact Details:

Primary ANZ sponsor contact details	Trans Tasman Radiation Oncology Group
Contact for public queries	Patrick Wheeler<br>+61 2 4014 3903<br>RAIDER@trog.com.au
Contact for scientific queries	Prof Farshad Foroudi<br>+61 3 9496 9797<br>farshad.foroudi@austin.org.au
Radiotherapy Quality Assurance Team	TROG<br>Email: qa@trog.com.au

Pre-Trial QA

1. Facility questionnaire: This is designed to gauge the IGRT experience of a centre to date. It collects information regarding the type of IGRT used, action thresholds, frequency of interventions and imaging doses. For simpler completion at centres, TROG have created two sections, one for Radiation Therapists and another section for Radiation Oncology Medical Physicists, which may be completed separately. This questionnaire can be accessed online at: www.trog.com.au. In addition, sites wishing to use inversely planned techniques must be credentialed to do so. Please contact qa@trog.com.au for further information.

2. Process document: Details are collected on all aspects of tasks for the complete patient pathway and includes details on all imaging procedures. A process document template can be found at http://www.trog.com.au/Trials-and-research-projects, RAIDER, Quality Assurance. TROG website password required.

3. Fiducial Marker Quality Assurance Document: To be completed by sites who have indicated that they intend to use fiducial markers. This document can be found at http://www.trog.com.au/Trials-and-research-projects, RAIDER, Quality Assurance. TROG website password required.

4. Outlining Benchmark cases: The P.I. from each recruiting site should complete the two outlining benchmarking cases available on the TROG website. One male (with lipiodol and one female (without lipiodol). The targets and organs at risk are to be named and delineated as per the RAIDER Radiotherapy Planning, Delivery and QA Guidelines.

   Male Case 1: Download TROG 14.02 BMKXX1 from http://www.trog.com.au/Trials-and-research-projects, RAIDER, Quality Assurance. TROG website password required.

For peer review only - http://bmjopen.bmj.com/site/about/guidelines.xhtml

Female Case 2: Download TROG 14.02 BMKXX2 from http://www.trog.com.au/Trials-and-research-projects, RAIDER, Quality Assurance. TROG website password required.

Once the outlining cases have been completed by the local P.I. an export of the CT images and structure sets in DICOM should be returned to the TROG QA team. All data must be transferred via Central Quality Management System (CQMS). CQMS can be accessed through https://cqms.trog.com.au/login.jsp. If you require a CQMS account, please contact qa@trog.com.au

5. Planning Benchmark case: One planning benchmark is to be planned according to the Group 3 randomisation arm of the trial.

   Download TROG 14.02 BMKXX3 from http://www.trog.com.au/Trials-and-research-projects, RAIDER, Quality Assurance. TROG website password required.

   Once the planning benchmark case (with three plans) has been created (with appropriate review and acceptance by the local P.I.) the export of the CT images, dose matrices, RT plan and structure sets in DICOM should be returned to the TROG QA team. All data must be transferred via the Central Quality Management System (CQMS). CQMS can be accessed through https://cqms.trog.com.au/login.jsp. If you require a CQMS account, please contact qa@trog.com.au.

6. In house IGRT training programme: It is a requirement of the RAIDER trial that sites have an established IGRT training programme already in place before joining the trial. Sites should be utilising cone beam CT for treatment of bladder patients. Successful participation in the TROG 10.01 BOLART trial including the e-learning will be regarded as satisfying these criteria. IGRT training programme details are requested as part of the Facility Questionnaire.

7. IGRT Credentialing:
   a. Bladder 'Plan of the Day' training: Two practice cases with six CBCTS each are provided for centres to work through with an accompanying training document. Case 1 includes answers with some step-by-step instructions, for case 2 the answers only are provided. The cases are provided in DICOM format via the TROG website. Sites must import the two cases with accompanying CBCTS into appropriate image matching/registration software. The training guidance document is accessible via the TROG website.

   b. Bladder 'Plan of the Day' training video: Available on the TROG website: www.trog.com.au

   c. RAIDER Plan of the Day assessment: Two patients with six CBCTs each (twelve match decisions) will be provided to sites to allow individuals to make plan of the day decisions/choices. Staff must record their match results using the Plan of the Day Assessment Form. Assessment forms must be submitted to qa@trog.com.au.

8. Dosimetry Audit: All sites who have participated in the TROG 10.01 BOLART trial will not be required to complete a dosimetry phantom study. For sites who have not participated

For peer review only - http://bmjopen.bmj.com/site/about/guidelines.xhtml

in TROG 10.01 BOLART please contact the TROG QA office at qa@trog.com for further information.

On-Trial QA

Please contact TROG QA for advice as required for the first patient planning and treatment. Teleconference facilities may be made available if required.

1. Quality assurance Pre-treatment Plan Review: Pre-treatment review of at least the **first** adaptive patient and the **first** dose escalated patient (if the first patient is not dose escalated) recruited from each site will be conducted by TROG. The **first** participant with fiducial marker placement from each must also be submitted for review.

   The treatment plan will be required for review at least one week prior to treatment commencement. If the review results are acceptable the participant will proceed to treatment.

   The following **four** adaptive patients from each site will undergo timely review (to be completed during the first week of treatment).

   A checklist of the source data required for each RT QA case will be provided by the TROG QA Office with specifications of timelines for data submission included. See below for instructions for the upload of source data required for real time pre-treatment radiotherapy QA.

2. Quality assurance Post-treatment Plan Review: Adaptive patients will be sampled for QA review at a rate of **one in five** for post-treatment review following the initial pre-treatment/timely sampling.

   Patients in the standard whole bladder radiotherapy arm will be sampled for QA review at a rate of **one in five** for post-treatment review.

   All participants will be required to submit data at the end of treatment. This data should be submitted via CQMS. To assist with this process a checklist of the source data required will be provided to you by TROG QA. Timelines for data submission will also be specified.

3. IGRT/POD selection support: The first adaptive patient randomised by all sites will be subject to a retrospective review. If centres have any difficulties in the plan selection, please do not hesitate to contact the TROG QA team.

Data Export and Upload

Australia and New Zealand sites uploading data to TROG CQMS are not required to anonymise data prior to upload. All DICOM files are automatically de-identified at the point of upload to CQMS.

Radiation Therapy Treatment Plan

An electronic export of the radiotherapy treatment planning data file from the treatment planning system is required in **DICOM-RT format.** Please submit the RT Treatment Plan Export

For peer review only - http://bmjopen.bmj.com/site/about/guidelines.xhtml

[CT files, structure set, RT plan and dose matrix for each PTV] using the **[RT Plan Upload]** function listed in CQMS. Exported files must be uploaded as a single zipped file into CQMS.

- Please include ALL planning CTs (e.g. include CT_30 and CT_60 for adaptive patients)

CBCT Data Upload

The following files are also to be uploaded using the **[RT Plan Upload]** function listed in CQMS for final end of RT QA review:

- All pre and post treatment CBCT (exported in DICOM format). Each CBCT dataset should be uploaded as a single zipped file. As a minimum for all groups, this should include week 1 and weekly post-treatment CBCTs.
- Registration objects
- Please refer to Appendix G, H and I for additional assistance when exporting CBCT datasets from ARIA, MOSAIQ and Raystation

Supplementary Information Data Upload

The following files are to be uploaded using the **[Other Upload]** function listed in CQMS for final end of RT QA review:

- Treatment prescription (including total dose, number of fractions, dose per fraction, prescription isodose)
- Treatment plan summary (including field information and beam parameters)
- Plan of the day decisions (Plan Selection Form)
- Verification images demonstrating correct export of the radiotherapy plan (JPEG of the DVH and isodose distribution).
- Daily Dose Record (including dates of treatment delivery)
- Imaging Log (verification of imaging performed)

For peer review only

For peer review only - http://bmjopen.bmj.com/site/about/guidelines.xhtml

APPENDIX C:

QA Benchmark Cases: Outlining and Planning

PRE-TRIAL OUTLINING BENCHMARK CASE

All centres wishing to participate in the RAIDER trial will need to complete the following contouring exercise. DICOM CT data sets can be downloaded from the RTTQA website (www.rttrialsqa.org.uk) or TROG website (www.trog.com.au). See QA Appendix A& B for more information.

Outlining Benchmark Case 1: T2N0M0 Male Pelvis

History: 72 year old male, presented with haematuria. Proceeded with TURBT and 3 cycles neo-adjuvant chemotherapy (cisplatin-gemcitabine). Post treatment cystoscopy shows pathological complete response (pCR). Patient is planned for chemo-radiotherapy to bladder.

Biopsy: biopsy at diagnosis consistent with pT2a G3 TCC with no associated distant CIS

Initial staging diagnostic information: Information available for contouring/GTV delineation includes:

- bladder map/cystoscopy showing tumour present left lateral wall around left ureteric orifice
- CT baseline (pre-chemotherapy)
- MRI baseline (pre-chemotherapy) and lipiodol injected at tumour / scar (post chemotherapy)

Radiotherapy contouring/planning (according to group 2 and 3): Planning scan at 30 minutes and 60 minutes reflect no filling (<50cc)**.**

Contouring Instructions:

Please import Case 1 into your TPS.
Please outline the following volumes:

- GTV_30
- CTV_30
- CTV_60
- RECTUM
- OTHER_BOWEL
- FEMORALJOINT_L or FemJoint_L
- FEMORALJOINT_R or Femjoint_R

Please create the following PTV volumes using the expansion information contained in Table 1 and Table 2:

- PTV_Sm
- PTV_Med
- PTV_Lar_30
- PTV2_Sm
- PTV2_Med

For peer review only - http://bmjopen.bmj.com/site/about/guidelines.xhtml

- PTV2_Lar_30

NB: Please refer to the additional diagnostic information available for this patient.

Additional Diagnostic Information for Outlining Benchmark Case 1

Letter from surgeon identifying clinical position of tumour at cystoscopy (Bladder map not available):

Diagnoses:
1. G3 pT2 at least TCC bladder.
2. Exercise induced asthma.

I would be grateful if you could review this pleasant retired nurse urgently. I performed his TURBT on the 19th June and he had a large bladder tumour on the left lateral wall with a solid base. It was invading anteriorly into the prostate and bladder neck. Post-operative EUA revealed a mobile mass and a firm prostate. The histology has come as G3 pT2 tcc bladder.

He initially failed a trial without catheter and is having his catheter removed again today. He is otherwise fit and well.

Yours sincerely

Dictated and approved by Consultant

eview only

Post-chemotherapy Cystoscopy

Cystoscopy, bladder biopsy and lipiodol injection

Lithotomy, WHO checklist, pressure points protected, gentamicin.

Bimanual no pelvic mass, smooth prostate

Excellent views with 21 Fr scope and 12° lens. No visible tumour in bladder. Scar above left ureteric orifice - biopsied and lipiodol injected submucosally. New red patch at dome seen– region biopsied and lipiodol injected. Additional three random biopsies taken.

Histopathology:

Fibrosis only at all biopsied sites. Therefore red patch at bladder dome although injected with lipiodol should not to be included in GTV volume)

For peer review only - http://bmjopen.bmj.com/site/about/guidelines.xhtml

Outlining Benchmark Case 2: T2N0M0 Female Pelvis

History: 66 year old female presented to urologist with isolated episode of frank haematuria. Proceeded to TURBT. She started neo-adjuvant chemotherapy (cisplatin-gemcitabine) but suffered deterioration in hearing so went on to have chemo-radiotherapy to bladder after 1 cycle.

Biopsy: G3 pT2 TCC bladder with focal adjacent CIS

Initial staging diagnostic information: Information available for contouring/GTV delineation bladder map/cystoscopy tumour present left posterior wall ureteric orifice, CT baseline (pre-chemotherapy), MRI baseline (pre-chemotherapy, post TURBT) - non-contributory.

Radiotherapy contouring/planning (according to group 2 and 3): Planning scan at 30 min and 60 min reflect filling (>50cc).

Contouring Instructions:

Please import Case 2 into your TPS.

Please outline the following volumes in accordance with instructions

- GTV_30
- GTV_60
- CTV_30
- CTV_60
- RECTUM
- OTHER_BOWEL
- FEMORALJOINT_L or FemJoint_L
- FEMORALJOINT_R or FemJoint_R

Please create the following PTV volumes using the expansion information contained in Table 1 and Table 2:

- PTV_Sm
- PTV_Med
- PTV_Lar_60
- PTV2_Sm
- PTV2_Med
- PTV2_Lar_60

Data Export

Once the outlining benchmark cases have been created, reviewed and accepted by the local PI, the export of the CT images and structure sets in DICOM should be returned to the appropriate QA team. See QA appendix A & B for information on data export.

For peer review only - http://bmjopen.bmj.com/site/about/guidelines.xhtml

PRE-TRIAL PLANNING BENCHMARK CASE

All trial centres must complete and submit the RAIDER pre-trial planning benchmark case. The CT DICOM data and pre-outlined structure set is available for download from www.rttrialsqa.org.uk (UK) or www.trog.com.au (Australia/NZ). Details for the planning cases are given in the next section.

Benchmark Planning Case: T2N0M0 TCC male pelvis

History: 63 year old male presented with frank haematuria, proceeded to TURBT; unsuitable for neo-adjuvant chemotherapy in view of co-morbidities, received chemo-radiotherapy to bladder

Biopsy: G3 pT2a TCC bladder

Initial staging diagnostic information: Information available for contouring/GTV delineation includes:

- Radiotherapy Bladder map/cystoscopy tumour present right bladder wall
- CT baseline,
- MRI baseline (post TURBT non-contributory).

Radiotherapy contouring/planning (according to group 3): Planning scan at 30 min and 60 min; filling (>50cc)**.** PTV small/medium contours on 30minute scan; PTV Large created on 60minute scan

Planning Exercise:

Please import the CT and structure sets into your own TPS system. Following the RAIDER trial protocol and radiotherapy planning and delivery guidelines please produce a series of three radiotherapy plans as the patient is randomised to the dose escalated adaptive tumour boost DART (group 3)

Structures:

The CT has been delineated by a trial clinician with the following structures:

Structure	Volume (cc)
GTV_30	10.0
GTV_60	12.3
CTV_30	153.4
CTV_60	272.2
PTV_Sm_	273.2
PTV_Med_	385.3
PTV_Lar_60	577.0
PTV2_Sm	40.5
PTV2_Med	76.1
PTV2_Lar_60	82.3
RECTUM	65.4
OTHER_BOWEL	314.5
FEMORALJOINT_L	63.1

For peer review only - http://bmjopen.bmj.com/site/about/guidelines.xhtml

FEMORALJOINT_R	62.1

Please DO NOT edit any of these structures. The names and volumes on which the structures appear on have been given so that you can check that the structures have been imported properly.

Data Export

Once the planning benchmark case (three plans) has been created and reviewed and accepted by the local PI, the export of the Plan Assessment Form (PAF), CT images, dose matrices, RTplan and structure sets in DICOM should be returned to the RTTQA team (UK) or TROG team (Australia/NZ). For information on data export see QA appendix A & B.

For peer review only

For peer review only - http://bmjopen.bmj.com/site/about/guidelines.xhtml

APPENDIX D

Quick Contouring Checklist Adaptive Arms

1) Outline CTV_30 on CT30. Check whether appropriate for urethra to be included.
2) Copy and paste CTV_30 onto CT60. Rename CTV_60. Edit this volume to take into consideration bladder filling taking care not to make the volume smaller inferiorly.
3) Check CTV_30 does not expand beyond CTV_60. Use Boolean functions can be used to ensure CTV_60 always encompasses CTV_30.
4) Check volume difference between CTV_30 and CTV_60. If less than 50cc, expand all PTV volumes from target volumes on CT30, if greater than 50cc create large PTVs from CT60 target volumes.
5) Outline GTV_30 (and GTV_60 if applicable)
6) Ensure GTV shares the same bladder wall as CTV by either manual editing and/or use of Boolean operators.
7) Create adaptive PTVs from CTV using the relevant expansions
8) Create adaptive PTV2s from GTV using the relevant expansions
9) Check PTV_Lar encompasses PTV_Med and PTV_Sml
10) All OARs are always outlined on CT30 (FEMORALJOINT_L, FEMORALJOINT_R, RECTUM and OTHER_BOWEL. Check OTHER_BOWEL volume is at least 2.0cm superior to PTV _Lar

For peer review only - http://bmjopen.bmj.com/site/about/guidelines.xhtml

APPENDIX E

Proposed patient intervention to be considered in circumstances where plan selection is not felt to be optimal.

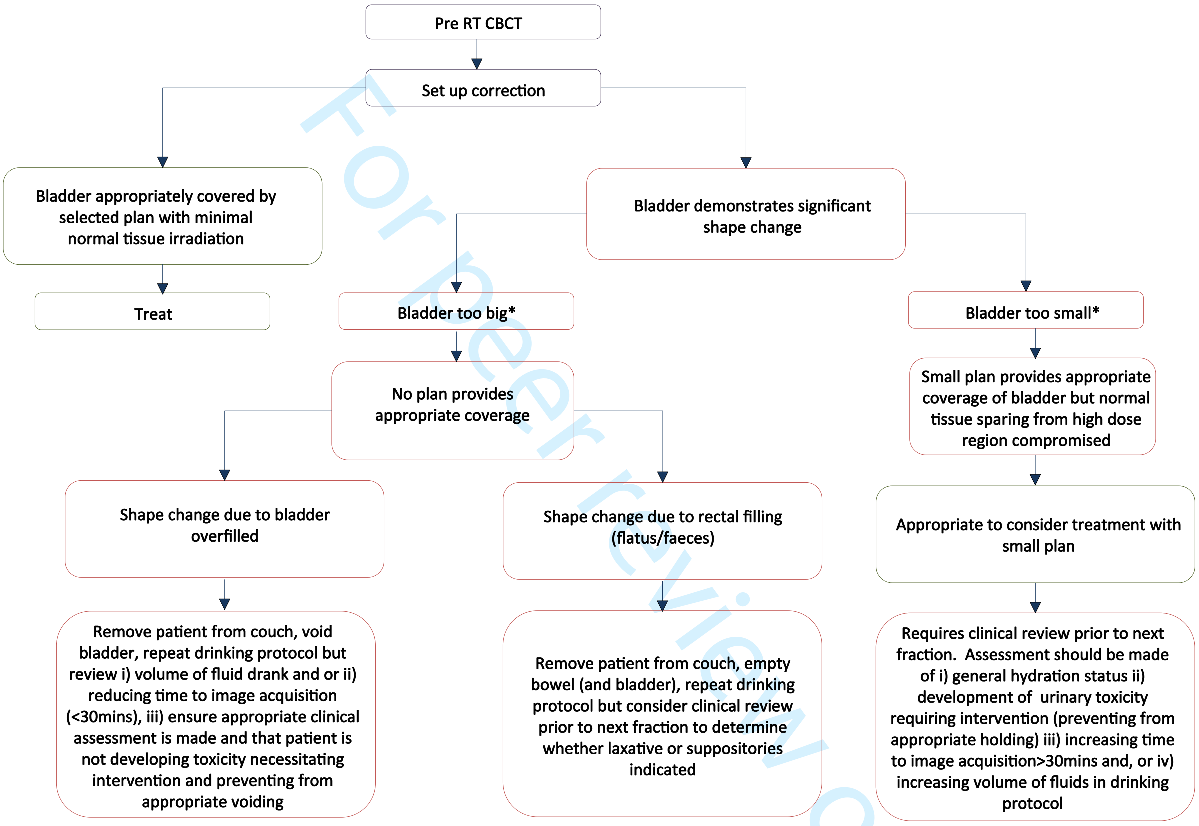

*If no plan provides appropriate coverage (bladder persistently too big or too small) more than twice in 5 fractions despite intervention, it is advised that the RTQA team is contacted for advice as in rare circumstances replan maybe indicated.

If the bladder is too small sites can consider treating with the small plan OR removing the patient from the treatment couch to allow for more filling.

**When considering changing the drinking protocol, only consider 1 timing or drinking change at a time. This will allow centres to establish which intervention has an effect on the patient's bladder.

For peer review only - http://bmjopen.bmj.com/site/about/guidelines.xhtml

RAIDER radiotherapy planning and delivery guidelines

APPENDIX F

RAIDER training cases and examples

1. Acceptable for treatment with chosen plan

Reference image

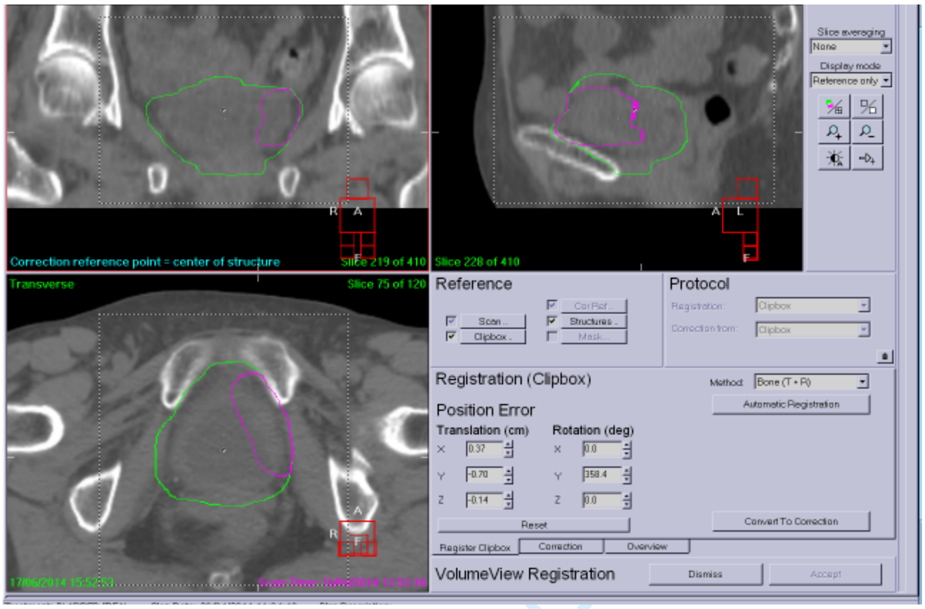

Localisation image

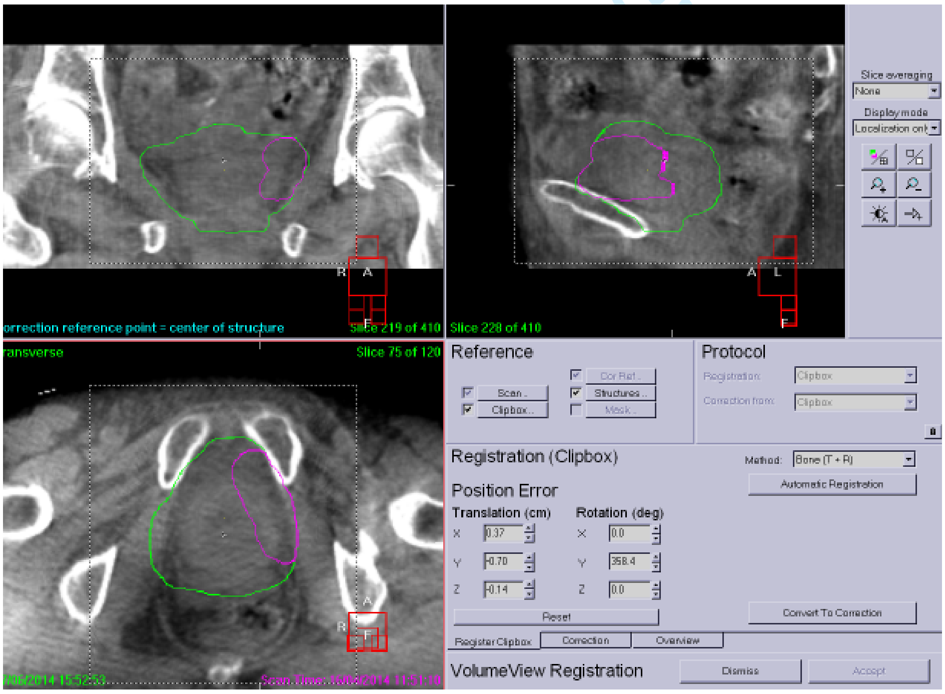

RAIDER radiotherapy planning and delivery guidelines

2. Bladder too small.

Bladder appears significantly under filled compared reference image, most apparent on sagittal localisation view. Note boost dose includes large proportion of bladder.

Acceptable for treatment with chosen plan as no compromise to bladder coverage. Patient review is recommended prior to next fraction to assess drinking protocol and, or time to image acquisition in order to optimise normal tissue sparing.

Reference image

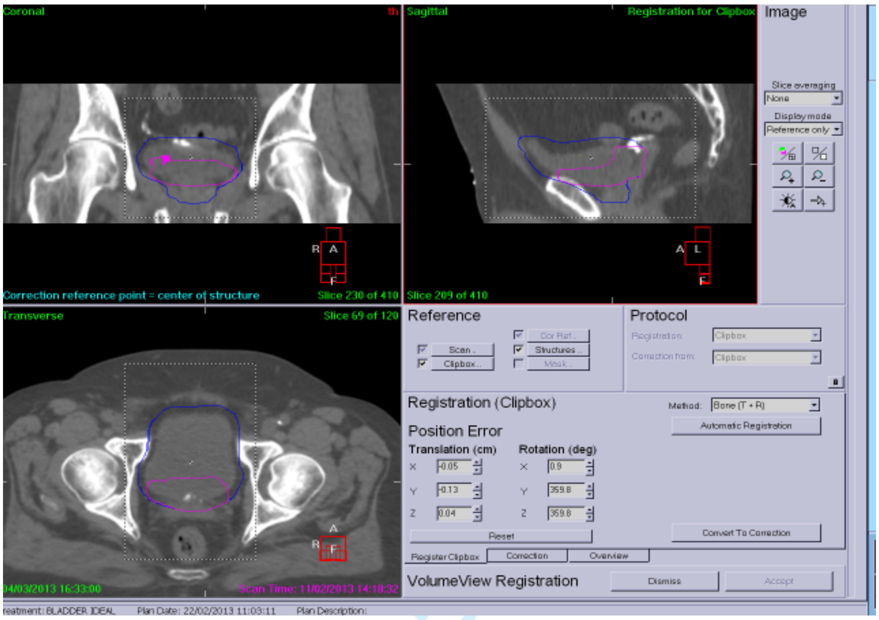

Localisation image

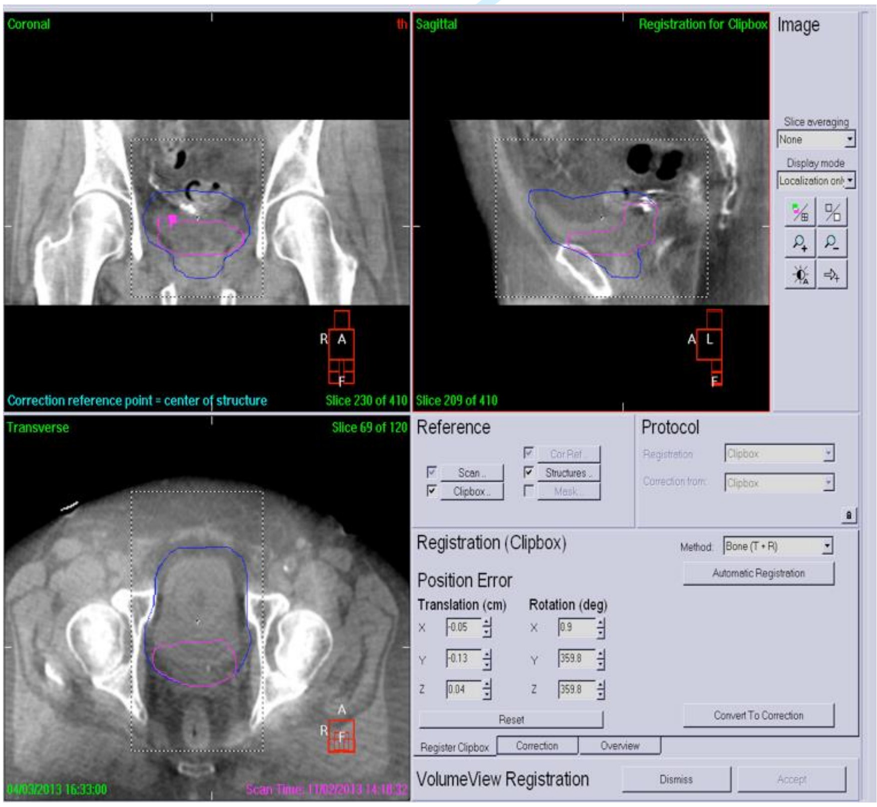

For peer review only - http://bmjopen.bmj.com/site/about/guidelines.xhtml

3. Bladder too large

No plan selection is acceptable for treatment (without compromise to bladder coverage).

Patient to empty bladder and repeat set-up with review of drinking protocol/time to CBCT acquisition.

Reference image

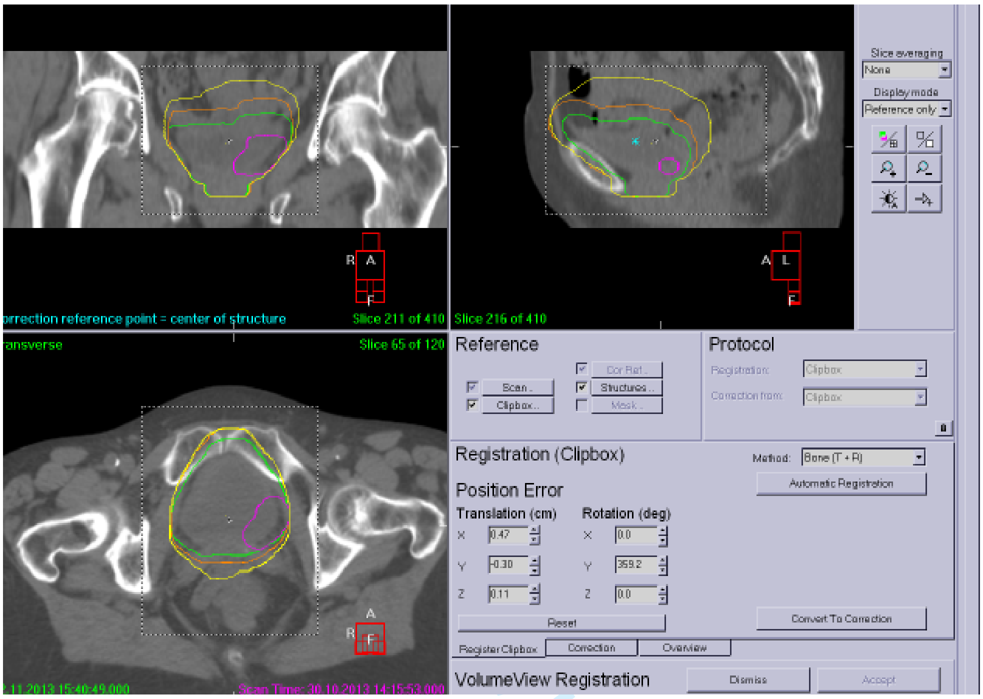

Localisation image with no structures

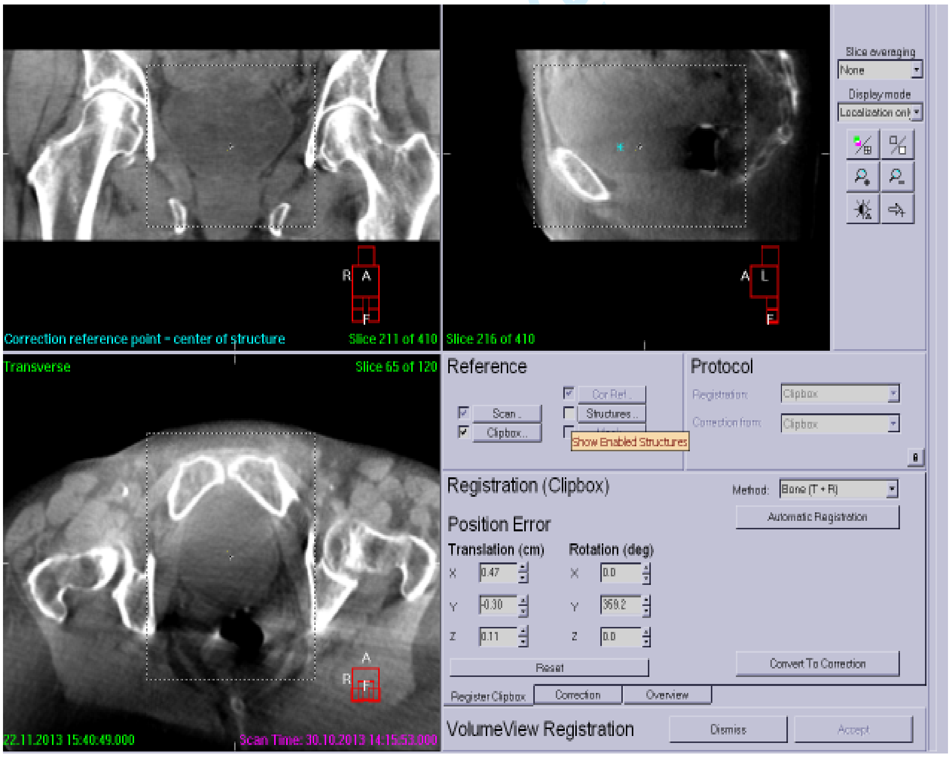

For peer review only - http://bmjopen.bmj.com/site/about/guidelines.xhtml

RAIDER radiotherapy planning and delivery guidelines

Reference image

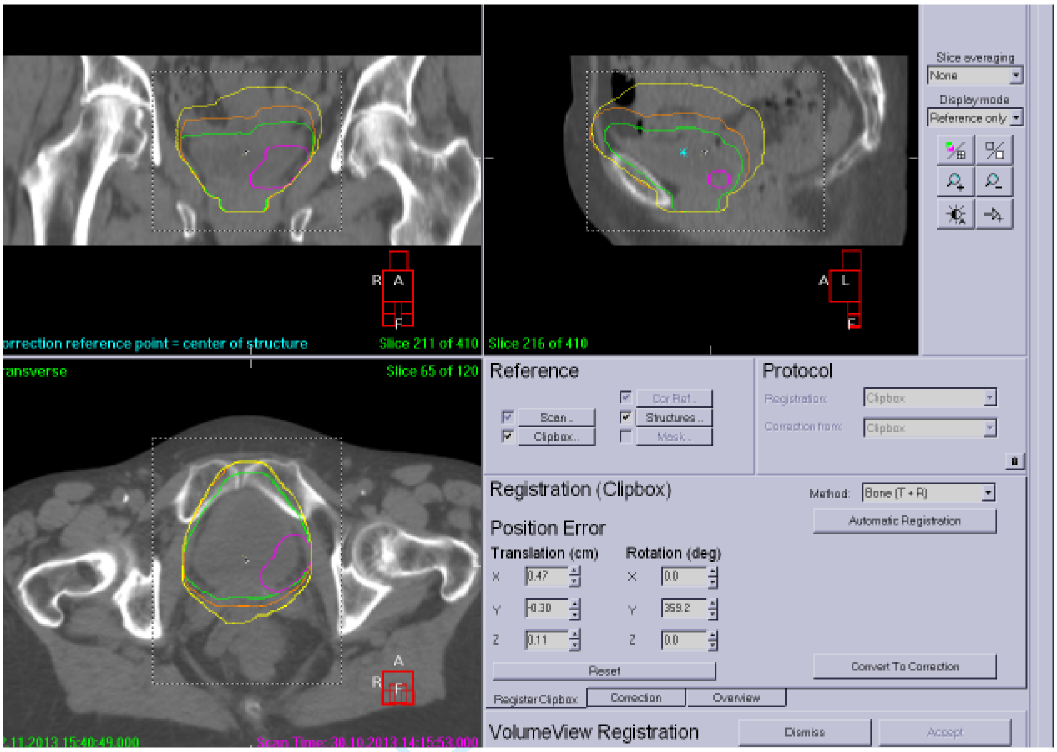

Localisation image with structures

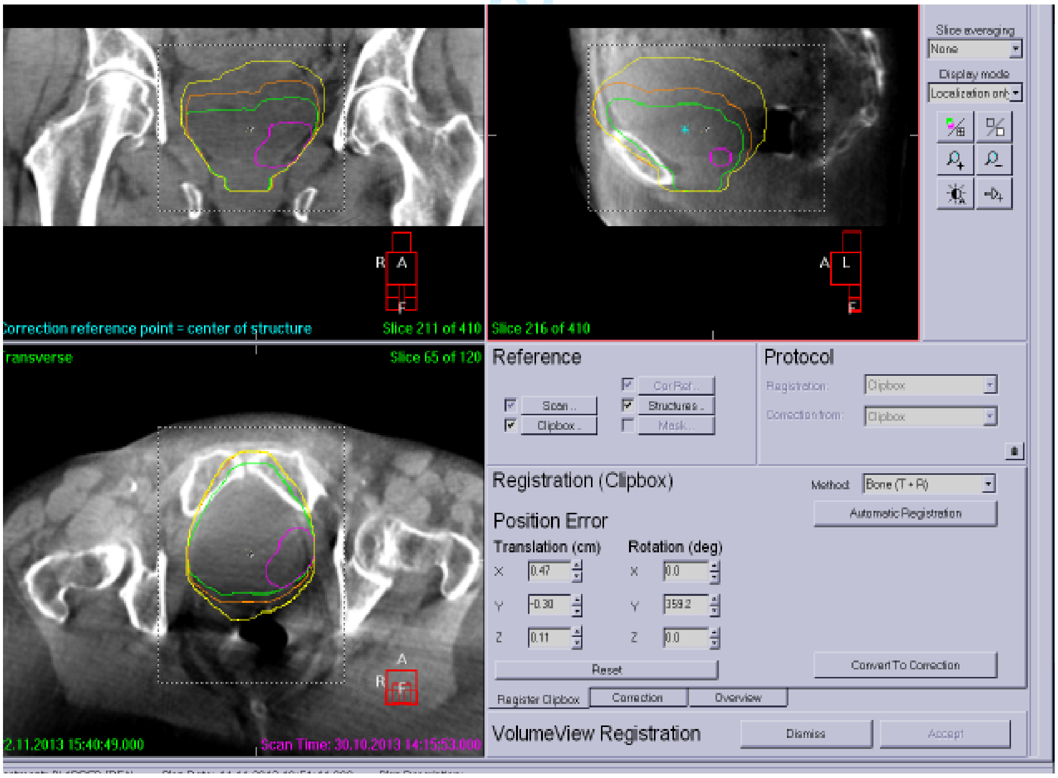

For peer review only - http://bmjopen.bmj.com/site/about/guidelines.xhtml

4. Fiducial marker artefact on CBCT interpretation

Image below demonstrates effect of lipiodol spill outside bladder resulting in significant artefact and degradation of image. Recommendation is that each CBCT should be closely reviewed with reference image and delineated bladder volume.

Reference image

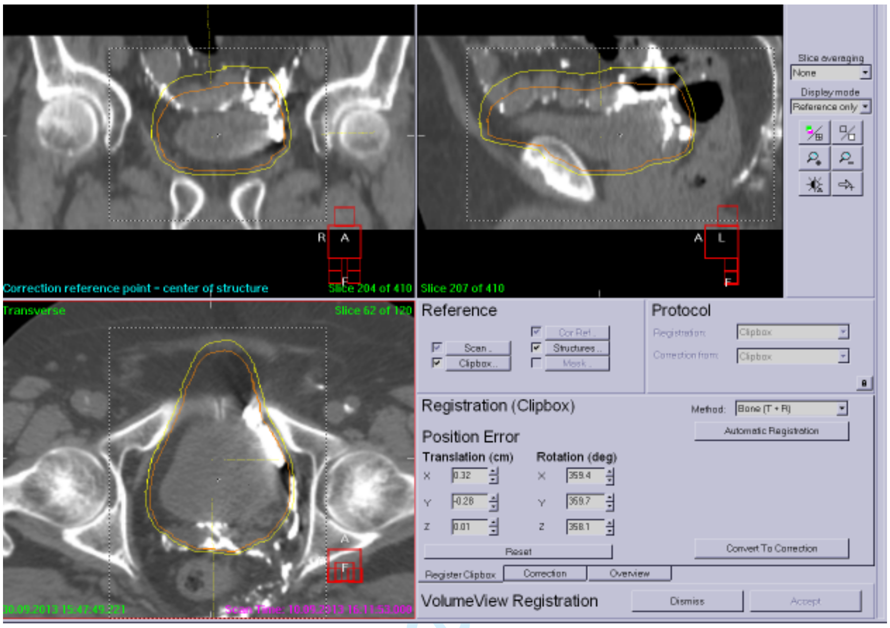

Localisation image

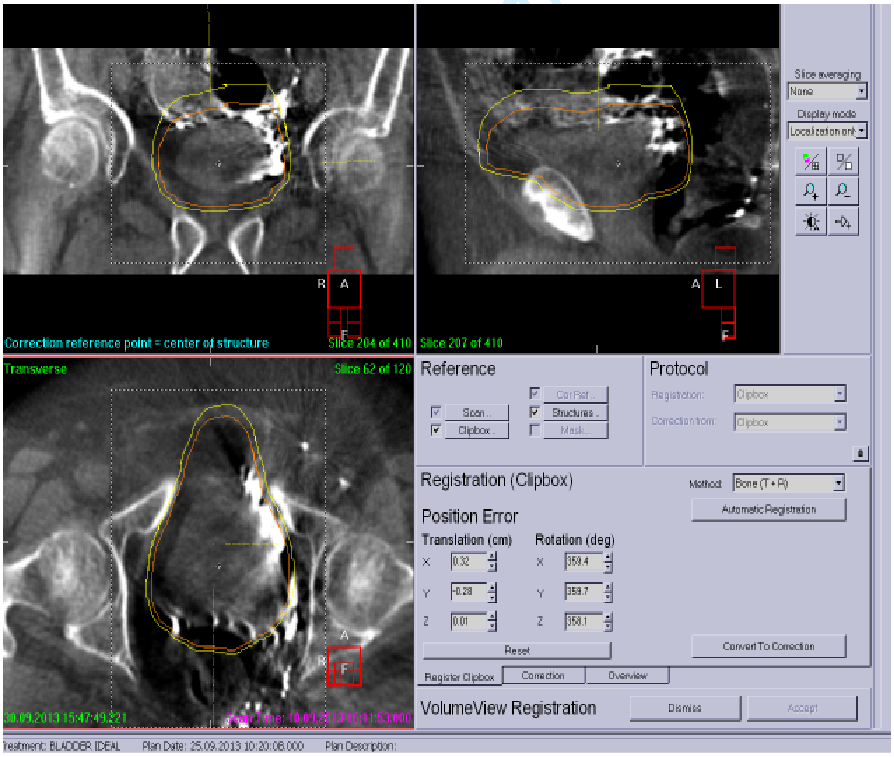

For peer review only - http://bmjopen.bmj.com/site/about/guidelines.xhtml

APPENDIX G

Quick Reference Guide Exporting CBCTs from Aria

These steps are for guidance only. Please contact your QA group if you have difficulties exporting data.

1) Load patient in Aria, go to Offline review and select one (any is ok) of the CBCTs to load
2) Select 'Session Timeline' tab at the bottom of the screen
3) Right click on the CBCT to export and select 'Export to DICOM' >> 'To 'DICOM Export to Pinnacle/External Anonymisation Software/Folder''
4) The Import Export window loads. Select the 'Show/Hide Tree' button to bring up a list of all CBCTs and registration objects
5) Select the radio buttons next to the CBCTs to export, using the date to identify them.
6) Click on the + to expand the folder called 'Registrations':

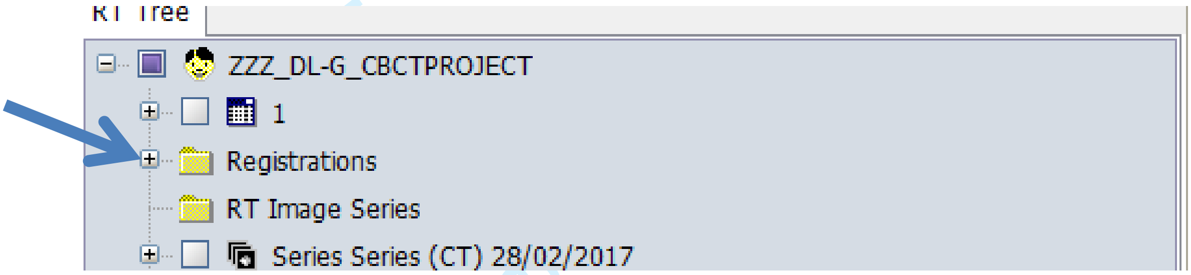

7) Select the radio buttons of the registration objects that have the same date as the CBCTs you wish to export.

8) Click the right arrow to export to the export folder/destination.

9) In windows explorer navigate to the export folder/destination and select the CBCTs and registration object files. Copy them to the DICOM anonymiser folder and run through your anonymiser to anonymise as per standard departmental working instructions. Please check the anonymization software does not remove the registration objects. If this occurs please contact RTTQA.

10) Save in a folder ready for export to RTTQA.

Please note that, for ease, whole patient exports can be submitted by the centre to RTTQA

For further information on suitable anonymisation software for ARIA exports please contact raiderqa.enh-tr@nhs.net

For peer review only - http://bmjopen.bmj.com/site/about/guidelines.xhtml

APPENDIX H

Quick Reference Guide Exporting CBCTs from Elekta/XVI

These steps are for guidance only. Please contact your QA group if you have difficulties exporting data.

Exporting from XVI

Images need to be exported individually, not as a treatment.

1. On the XVI acquisition PC select the image to be exported
2. Select IMAGE from the tool bar
   2.1. EXPORT
   2.2. DICOM SERVER 'select TPS/online server'
   2.3. OK
3. The next screen gives 3 options
   - Option 1 – In the Option 1 list, select a multiple of the voxel size in the reconstructed volume for the CT slice thickness. This can be done without a reference dataset being available and hence imports the CBCT into pinnacle without any co-ordinates related to the reference image. This is not likely to be useful.
   - Option 2 – Only available if image registration was done and approved for this reference image. The position of the VolumeView™ exported is the position **before** registration.
   - Option 3 – Only available if image registration was done and approved for this reference image. The position of the VolumeView™ exported is the position **after** registration.

3.1 Select Option 3 as the information required is as the patient was treated i.e. after registration (e.g. if patient was treated with correction).

NB Registration has to be performed for option 3 to be available.

3.2. In the Export options area, click the Create CT button.

3.3 EXPORT

For peer review only - http://bmjopen.bmj.com/site/about/guidelines.xhtml

APPENDIX I

Exporting CBCTs from Raystation

For centres exporting CBCTs from Raystation please contact raiderqa.enh-tr@nhs.net

For peer review only

This is a controlled document and should not be copied, distributed or reproduced without the written permission of the ICR-CTSU

For peer review only - http://bmjopen.bmj.com/site/about/guidelines.xhtml

Please print on hospital's headed paper

RAIDER

A **R**andomised phase II trial of **A**daptive **I**mage guided standard or **D**ose **E**scalated tumour boost **R**adiotherapy in the treatment of transitional cell carcinoma of the bladder

PATIENT INFORMATION SHEET

For peer review only

We would like to invite you to take part in a research study called RAIDER.

Before you decide whether to take part, it is important that you understand why the research is being done and what it would involve for you. One of your doctors or nurses will go through this information sheet with you and answer any questions you may have. Please take time to read the information carefully and to discuss it with relatives, friends and your GP if you wish. Please ask if anything is unclear or you need any further information.

Thank you for reading this and considering taking part in our research.

Why am I being invited to take part?

We are inviting you to join this study because your doctor has found cancer that has grown into the wall of your bladder (muscle invasive bladder cancer) and you are interested in receiving radiotherapy treatment which will be given in small doses every day for 7 weeks.

What is radiotherapy treatment?

Radiotherapy uses targeted beams of high strength x-rays to kill cancer cells. Because radiotherapy can also cause damage to non-cancer cells, the treatment is carefully planned by doctors and physicists so that only your bladder and a small border surrounding it is treated with the highest radiotherapy dose.

Radiotherapy is individually designed for each patient, based on a CT scan taken a few weeks before treatment. This CT scan tells your doctor about the position and shape of your bladder.

The bladder can move within the body depending on how full it is and because of where it is in relation to the bowel. It is important that the radiotherapy does not miss any of the bladder tumour because of this movement, so a safety margin is added around the bladder on the radiotherapy treatment plan.

CANCER RESEARCH UK

Each patient would usually have one radiotherapy treatment plan designed for them so that the radiotherapy is targeted at their tumour.

When radiotherapy treatment is given, the patient lies still on a bed whilst the radiotherapy machine moves around to send the radiotherapy beams from different directions. These beams all focus on where the bladder is, to make sure the whole bladder receives the highest radiotherapy dose possible.

What is adaptive radiotherapy treatment?

We are now able to take a scan of where the bladder is when a patient is lying on the radiotherapy bed before each treatment. This means we can target the bladder more precisely.

In this study we are looking at whether it is possible to design three treatment plans (small, medium and large) and then choose the one that best fits the size of the bladder on the patient's treatment day. This is called 'adaptive radiotherapy'. Adaptive radiotherapy may allow treatment to be given with smaller safety margins, which may lead to fewer side effects.

What is tumour focused radiotherapy treatment?

It is also possible to focus the highest dose of radiotherapy on the bladder tumour. This means that the rest of the bladder can be given a lower dose of radiotherapy. This may mean fewer side effects as the rest of the bladder will be given less radiotherapy. It also allows us to find out whether a higher dose of radiotherapy can be given only to the tumour (tumour boost), to see if this will reduce the chance of the cancer returning, whilst also keeping any side effects as mild as possible.

What is the purpose of RAIDER?

RAIDER is based on a study of adaptive tumour boost radiotherapy which was conducted at one UK hospital. We hope to show that this complex radiotherapy can be given by radiotherapy departments at different hospitals. If this is possible, we want to look at whether the side effects are similar to those experienced by people receiving standard radiotherapy.

RAIDER is based on several smaller studies conducted at hospitals worldwide, which suggest that treatment using these radiotherapy techniques could help to reduce side effects. Although these smaller studies are promising, RAIDER is the largest study of these techniques and has been designed to give us as much information as possible. We will use the results of RAIDER to develop a future study to investigate whether these techniques could improve how well radiotherapy cures bladder cancer.

What would happen if I took part?

At least 240 people in the UK, Australia and New Zealand will be included in RAIDER. All RAIDER participants will be treated with daily radiotherapy.

For peer review only

Everyone who agrees to take part in this research study will be in one of three groups:

1. Group 1: Standard radiotherapy: One out of four people taking part will be given standard bladder radiotherapy using the same radiotherapy treatment plan each time, treating their whole bladder to the same dose of radiotherapy.

2. Group 2: Adaptive tumour focused radiotherapy: One out of four participants will be given adaptive tumour focused radiotherapy, using the radiotherapy treatment plan which fits the size of their bladder the best. The standard dose of radiotherapy will be targeted at the tumour, with the rest of the bladder receiving a lower dose than normal.

3. Group 3: Adaptive tumour boost radiotherapy: Two out of four participants will have adaptive tumour boost radiotherapy, using the radiotherapy treatment plan which fits the size of their bladder the best. A higher than standard dose of radiotherapy will be targeted at the tumour, with the rest of the bladder receiving a lower dose than normal.

The only way to make sure that the people in the three groups are as similar as possible is to have the treatment decided upon by chance: a process called randomisation. This process ensures that the treatments are compared fully and fairly.

If you agree to take part, your doctor or nurse will ring the research centre. The centre will then record your details and tell your specialist your treatment, which will be selected by chance. This means you could have any of the three treatments described above. Whichever group you are in, you will be treated with the best possible care and will be monitored closely.

<u>What do I have to do before my radiotherapy treatment?</u>
To make sure your treatment is as effective as possible, it has to be carefully planned by your doctor and other specialised staff (radiographers and physicists).

To help with this, your doctor may wish you to have small markers (fiducial markers) placed in your bladder. If this applies to you, you will be given the bladder tumour marker information leaflet to read.

You will need to visit the hospital for a planning CT scan before you start radiotherapy. The radiographers will also take measurements that are needed for the treatment plan and will make small permanent marks which help to line up the radiation beam. Radiotherapy is a very precise treatment and it is important that you are able to lie in exactly the same position for every treatment.

For peer review only

Group 1 – standard radiotherapy planning visit:

You will have a planning CT scan taken with your bladder empty. All of the planning procedures for the standard group are part of the routine care for patients receiving bladder radiotherapy, so you would have them even if you choose not to take part in the RAIDER research study. The planning session at the radiotherapy department usually takes place once and lasts about 30 minutes.

Group 2 and 3 – adaptive tumour focused radiotherapy planning visit:

If you are in group 2 or 3, we will ask you to empty your bladder and then to drink 350ml of water (just over ½ pint). We will take the first scan at 30 minutes and the next at 60 minutes after drinking, as your bladder fills. If you decide to join the study you will be given a leaflet describing drinking guidelines. The planning session lasts about 70 minutes in total, but each scan will only take a few minutes.

<u>What do I have to do during my radiotherapy treatment?</u>

Your treatment will be given daily. If you are in group one we will ask you to empty your bladder immediately before each treatment. If you are in group 2 or 3, we will ask you to empty your bladder and then drink 350mls of water 30 minutes before your planned radiotherapy treatment.

For all groups, once the radiographer has helped you to get into position and made sure that you are comfortable, we may take a scan in the treatment room. This will take about 2 minutes.

For patients in group one receiving standard radiotherapy, this scan will be used to make sure the bladder is in the area which will receive the highest dose of radiotherapy.

If you are receiving adaptive tumour focused radiotherapy we will use the information from the pre-treatment scan to choose the best radiotherapy treatment plan to fit your bladder size and the position of your tumour. This will take around five to ten minutes. The plan will be selected by a specially trained doctor or radiographer and checked by a second trained person before you receive your radiotherapy treatment.

You will need to lie still for up to 20 minutes whilst the machine moves around you to deliver the radiotherapy from different angles. You will not feel anything, as it is similar to having an x-ray.

During your radiotherapy treatment you will be seen by your doctor and/or nurse/radiographer every week to record and treat any side effects that you may be experiencing. They will also take a small sample of blood before treatment starts and again during the first, fourth and sixth week of radiotherapy.

For peer review only

Chemotherapy treatment during radiotherapy

Your doctor may suggest that you also have chemotherapy while you have your radiotherapy treatment. You will be able to take part in RAIDER whether or not you have chemotherapy. Your doctor will discuss the possibility of chemotherapy with you.

How often will I need to visit the hospital after my treatment?

Everyone in the RAIDER study will be asked to visit the hospital for check-ups on the same schedule after treatment,as described below:

- *10 weeks after the start of radiotherapy:* assessment of side effects
- *3 months after the end of radiotherapy:* cystoscopy (inspection of your bladder with a telescope) under general anaesthetic & biopsy of the site of the tumour; blood sample; chest x-ray; assessment of side effects
- *6 months:* cystoscopy under local anaesthetic; blood sample; CT scan of abdomen and pelvis; chest x-ray or CT scan; assessment of side effects
- *9 months:* cystoscopy under local anaesthetic; assessment of side effects
- *12 months:* cystoscopy under local anaesthetic; CT scan of abdomen and pelvis; chest x-ray or CT scan; assessment of side effects
- *18 months:* cystoscopy under local anaesthetic; chest x-ray or CT scan; assessment of side effects
- *24 months:* cystoscopy under local anaesthetic; CT scan of abdomen and pelvis; chest x-ray or CT scan; assessment of side effects
- *36, 48, 60 months:* cystoscopy under local anaesthetic; chest x-ray; assessment of side effects

A summary of these visits is on page 15 of the patient information sheet, for your reference should you decide to join the study.

Commented [RL1]: NOTE TO CENTRES – UPDATE PAGE NUMBER WHEN PRINTING ON HEADED PAPER

These visits have been designed to be as similar as possible to what would happen if you decided not to join the RAIDER study. If your cancer is found to have returned during study check-ups your doctor will discuss further treatment options with you.

After your 5 year (60 month) visit, we would like to collect basic information about your health from any routine follow up visits you have, and also from national electronic databases which are kept on everyone's health status.

For peer review only - http://bmjopen.bmj.com/site/about/guidelines.xhtml

RAIDER
Study Design

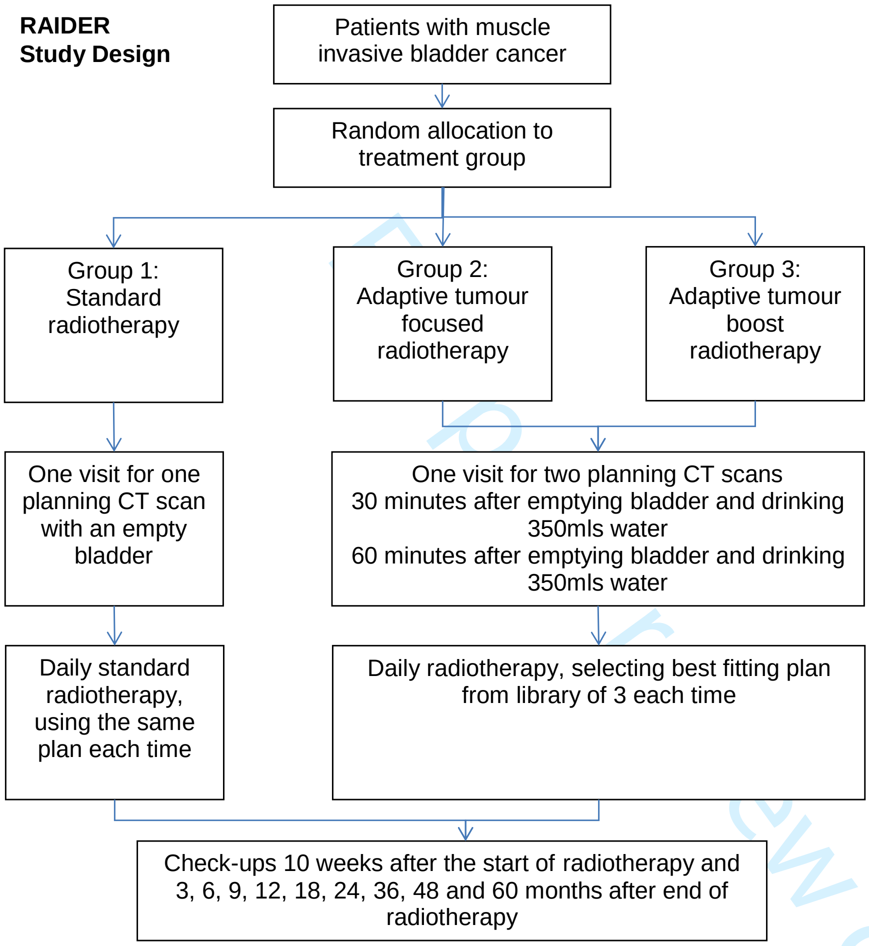

What are the possible side effects of treatment?

Patients who have radiotherapy can experience some side effects. These can occur in anyone receiving radiotherapy to the bladder whether or not they are in the RAIDER study. It is difficult to predict whether you will have some, all, or none of the side effects, or how severe they may be. They are usually mild and short lived but can sometimes be more serious.

Please let your doctor or nurse know about any side effects that you are concerned about so they can advise you what to do. Their telephone numbers are listed on page 12. There is also 24 hour support available from your hospital, to provide access to immediate medical care in the event of any serious problems.

Commented [BP2]: NOTE TO CENTRES – UPDATE PAGE NUMBER WHEN PRINTING ON HEADED PAPER

For peer review only - http://bmjopen.bmj.com/site/about/guidelines.xhtml

Not all patients will experience all of these side effects and we can give you medications to treat any side effects you may experience.

You will be able to carry out most of your normal activities during radiotherapy, but you may feel more tired than normal and may need to rest more.

Side effects that can develop during radiotherapy may include:

- diarrhoea (around 3 in 10 people)
- needing to urinate more often (around 5 in 10 people)
- bleeding, pain or discomfort on passing urine (around 2 in 10 people)
- passing stools more frequently or with pain (around 1 in 10 people)

Some side effects can develop several months after radiotherapy ends. These include:

- a need to urinate more often or more urgently (around 2 in 10 people)
- bowel changes due to scarring or bleeding (around 5 in 100 people)
- vaginal scarring (around 3 in 10 women)
- problems with getting and maintaining erections (around 2 in 10 men)
- infertility (all women, around 5 in 10 men)

A few patients may develop long term effects. These are usually mild but may occasionally be serious and require treatment.

If you are in group 3 and receive a tumour focused boost as part of your radiotherapy treatment, you will be receiving a higher total dose of radiotherapy to the tumour than people in other groups. There is therefore a possible increased risk of radiotherapy side effects, but the radiotherapy techniques used in this trial aim to keep the risk of side effects similar to those experienced following the standard dose. Side effects in group 3 will be very closely monitored throughout the study and if they appear to be higher than expected, treatment within this group will be stopped, and everyone in group 3 will receive standard dose tumour focused radiotherapy.

Do I have to take part?

No, it is up to you to decide whether to take part or not. If you decide to take part, you will be given this information sheet to keep and will be asked to sign a consent form. You are free to change your mind and withdraw from the study at any time without giving a reason. If you do choose to withdraw, your doctor will discuss with you the best treatment options available.

What are the alternatives to this study?

If you decide not to participate in this study, it will not affect the usual standard of care you receive. Standard recommended treatments for muscle invasive bladder cancer are surgery to remove the bladder or daily radiotherapy using one treatment plan and the

For peer review only

same dose to treat the whole bladder. If you do not take part in RAIDER your doctor will discuss all your alternative options with you.

What are the possible benefits of taking part?

Everyone in the study will receive scans regularly throughout treatment. This may make the radiotherapy more accurate than if it was given without the scan. If you are in the adaptive radiotherapy groups you will receive radiotherapy treatment with the smallest possible safety margin each time and this may reduce the risk of side effects. If you are in the tumour focused boost group, the increased radiation dose to your tumour may control your bladder cancer better.

What are the possible disadvantages of taking part?

Tumour focused adaptive radiotherapy could cause an increased risk of missing the tumour, but everyone who gives treatment as part of RAIDER will be fully trained and treatment will be checked by a second trained observer before it is given. There is also a risk that sparing the bladder from exposure to full dose radiotherapy could cause an increased risk of the cancer returning elsewhere in the bladder, however previous studies have not suggested this, and any indication of this will be carefully monitored in all patients who join RAIDER.

It is possible that the side effects of tumour focused boost radiotherapy might be worse than with standard treatment, but this will be monitored for all RAIDER participants and the study will be stopped if people experience bad side effects.

The selection and confirmation of the radiotherapy treatment plan will extend the length of each radiotherapy treatment by about 5 to 10 minutes for patients receiving adaptive radiotherapy.

You may have more CT scans than you would if you did not take part in RAIDER because you will have one before each treatment, and if you are in the tumour focused groups you will have two planning scans rather than one. If you are in the tumour focused boost group you will receive a higher dose of radiotherapy than you would otherwise. All of these factors mean that you could be exposed to more radiation than you would be otherwise, which may lead to an increased risk of developing a second cancer later in life.

Before participating you should also consider if this will affect any insurance you have and seek advice if necessary.

How will confidentiality be maintained?

Your medical notes will be seen by authorised members of the research team at your hospital, so that they can collect information needed for the RAIDER study. When you join the study, your name, date of birth, postcode, hospital number and NHS or Community Health Index (CHI) number will be passed to the Institute of Cancer Research Clinical Trials and Statistics Unit(ICR-CTSU) where the study is being coordinated. You will be

For peer review only

given a unique registration number, which will be used together with your initials and date of birth on forms that the research staff will send to the ICR-CTSU. All information about you will be coded with this registration number and will be stored securely. It will be treated as strictly confidential and nothing that might identify you will be revealed to any third party.

Scientific employees of ICR-CTSU, and those conducting the study with them, including the national radiotherapy quality assurance team, may need to examine your medical records to ensure the study is being run properly and that the information collected on the forms is correct, but your confidentiality will be protected at all times.

We will contact your hospital over the years to find out how you are getting on. Ideally we would like to do this for life, but patients often change address or GP or lose touch with their hospital. If this happens we would like to use national records which are kept on everyone's health status to find out how you are. One of these is held at the General Register Office (GRO). We will need to give them enough information to identify you. This is usually your name, date of birth and NHS number (or Community Health Index and/or hospital number in Scotland). These details are confidential and will only be used for the purposes of the RAIDER study. Please initial the consent form to show that we have your permission to do this – if you do not agree, we will not seek this information.

The Institute of Cancer Research is the Sponsor for this study based in the United Kingdom. We will be using information from you and/or your medical records in order to undertake this study and will act as the data controller for this study. This means that we are responsible for looking after your information and using it properly. Our legal basis for processing your data is task in the public interest for scientific research purposes. The Institute of Cancer Research will keep identifiable information about you for 20 years after the study has finished.

Your rights to access, change or move your information are limited, as we need to manage your information in specific ways in order for the research to be reliable and accurate. If you withdraw from the study, we will keep the information about you that we have already obtained. To safeguard your rights, we will use the minimum personally-identifiable information possible.

You can find out more about how we use your information at www.icr.ac.uk/our-research/centres-and-collaborations/centres-at-the-icr/clinical-trials-and-statistics-unit/transparency.

[NHS site] will collect information from you and/or your medical records for this research study in accordance with our instructions.

Commented [BP3]: NOTE TO CENTRES TO UPDATE

[NHS site] will use your name, NHS number and contact details to contact you about the research study, and make sure that relevant information about the study is recorded for your care, and to oversee the quality of the study. Individuals from The Institute of Cancer Research and regulatory organisations may look at your medical and research records to check the accuracy of the research study. [NHS site] will pass these details to The Institute

Commented [BP4]: NOTE TO CENTRES TO UPDATE

Commented [BP5]: NOTE TO CENTRES TO UPDATE

For peer review only - http://bmjopen.bmj.com/site/about/guidelines.xhtml

of Cancer Research along with the information collected from you and/or your medical records. The only people in The Institute of Cancer Research who will have access to information that identifies you will be people who need to contact you to send a Quality of Life booklet by post or audit the data collection process. Only members of the research teams at your hospital and the ICR-CTSU will have access to the information that could allow this trial ID number to be linked to you.

[NHS site] will keep identifiable information about you from this study for 20 years after the study has finished.

Commented [BP6]: NOTE TO SITE TO UPDATE

Data sharing

When you agree to take part in a research study, the information about your health and care may be provided to researchers running other research studies in this organisation and in other organisations. These organisations may be universities, NHS organisations or companies involved in health and care research in this country or abroad. Your information will only be used by organisations and researchers to conduct research in accordance with the UK Policy Framework for Health and Social Care Research.

Your information could be used for research in any aspect of health or care, and could be combined with information about you from other sources held by researchers, the NHS or government.

Where this information could identify you, the information will be held securely with strict arrangements about who can access the information. The information will only be used for the purpose of health and care research, or to contact you about future opportunities to participate in research. It will not be used to make decisions about future services available to you, such as insurance.

Where there is a risk that you can be identified your data will only be used in research that has been independently reviewed by an ethics committee.

What will happen to the results of the research study?

Independent experts will review the safety and progress of the research whilst it is being carried out, and the results will be published in a respected medical journal once we are sure they are reliable. No information that could identify you will be included and you will not be identified in any report or publication.

We will summarise the results for participants once they are available. Your hospital will be able to give you a copy and results will also be available on Cancer Research UK's patient website (www.cancerhelp.org.uk).

What if relevant new information becomes available?

Sometimes we get new information about the treatment being studied. If this happens, your doctor will tell you and discuss whether you should continue in the study. If you decide not to carry on, your doctor will make arrangements for your care to continue.

For peer review only - http://bmjopen.bmj.com/site/about/guidelines.xhtml

Will I be paid for taking part in this study?
No. Neither you nor your doctor will be paid for taking part in this study.

What if there is a problem?
Any complaint about the way you have been dealt with during this study, or any possible harm you might suffer, will be addressed. Your progress will be watched closely and you will be offered whatever help is available to cope with any side effects. Occasionally some patients need a short stay in hospital for side effects to be treated, and on rare occasions these can be serious. If this were to happen, full details of what has happened will be reviewed carefully by the Doctor who has overall responsibility for the RAIDER study . It is unlikely that anything will go wrong with your treatment or care, but if you wish to complain about any aspect of the way you have been approached or treated during the course of the study you can do so using the normal NHS complaints procedure. Concerns should be raised by speaking to a member of staff at your hospital or by talking to the local Patient Advice and Liaison Service (PALS) which has been established in every NHS Trust.

Commented [RL7]: NOTE TO CENTRES: PLEASE REPLACE PALS INFORMATION WITH EQUIVALENT LOCAL ORGANISATION IF CENTRE IS OUTSIDE ENGLAND

NHS bodies are liable for clinical negligence and other negligent harm to individuals covered by their duty of care. In the event that something does go wrong and you are harmed during the research and this is due to someone's negligence then you may have grounds for a legal action for compensation against the NHS Trust but you may have to pay your legal costs. Alternative indemnity arrangements apply to private clinics.

What if I don't want to carry on with the study?
You are free to withdraw from the study at any time. You do not have to give a reason and your future treatment and care will not be affected. If you change your mind about having treatment or follow up within this study, we would still like to collect information about how you are getting on. The information we need is routinely recorded in your medical records at your standard hospital visits and you would not need to do anything.

Who is organising and funding the research?
RAIDER is organised by doctors at the Royal Marsden Hospital in London, in collaboration with other leading doctors across the country and the Institute of Cancer Research Clinical Trials and Statistics Unit in London. The research is approved and funded by Cancer Research UK, who are providing funding to run the trial in the UK. The National Health Service Research and Development Executive will pay for any extra nursing and administrative costs incurred by participating hospitals and the National Institute for Health Research will pay for some of the costs of conducting the research at participating hospitals.

Who reviewed this study?
All research in the NHS is reviewed by an independent group of people, called a Research Ethics Committee, to protect participants' safety, rights, wellbeing and dignity. RAIDER has been reviewed and approved by London – Surrey Borders Ethics Committee on behalf

For peer review only - http://bmjopen.bmj.com/site/about/guidelines.xhtml

of all hospitals throughout the UK. It has also been reviewed and approved by Cancer Research UK and reviewed and endorsed by patient and carer representatives from the NCRI Consumer Liaison Group (www.ncri.org.uk).

What happens now?

You will have some time to think about the study and make your decision. Your doctor, nurse or radiographer will be happy to answer any questions. You may wish to discuss it with your family or friends. Once you have reached your decision please let your doctor or nurse know. You will be asked to sign a consent form and will be given a copy to keep together with this information sheet. Please keep this information sheet and copies of the signed consent form. Your GP will be told that you are taking part in the RAIDER study. If at any time you have any questions about the study you should contact your hospital consultant.

Further information

Macmillan Cancer support is a registered charity and helps with all the things that people affected by cancer want and need, from specialist health care and information to practical, emotional and financial support (www.macmillan.org.uk).You can also learn more about clinical trials on the Cancer Research UK's patient website (www.cancerhelp.org.uk).

Contact details

Commented [BP8]: NOTE TO SITE TO UPDATE

If at any time you have any questions about the study please contact your local study team:

Local consultant's name:

Local research nurse/radiographer:

Address:

Telephone:

24 hour contact number:

Thank you for your interest in our research.

For peer review only

Optional components to the study:

If you agree to participate in the main RAIDER trial, you will be invited to take part in the following sub-studies:

1. Side effects questionnaire study
2. Donation of routine samples from surgery

If I want to be part of the RAIDER study, do I have to take part in the sub-studies?

No. Taking part in RAIDER does not mean you have to take part in the sub-studies. You will be given the chance to discuss RAIDER and you can then decide whether you want to take part.

The following pages of this information sheet give further details about these sub-studies.

1. Side effects questionnaire study

The main reason we are carrying out the RAIDER study is to look at the side effects of the radiotherapy treatment. If you decide to take part in RAIDER, we would like you to complete short questionnaires to describe any side effects that you may experience.

This is an optional part of the study but completed questionnaires will help us to understand more about the side effects of this radiotherapy treatment from your point of view. Completing a questionnaire should take no longer than 20 minutes.

If you agree to take part, we will ask you to fill in a questionnaire before you start radiotherapy, at the end of your radiotherapy treatment and then at 3, 6, 12, 18 and 24 months afterwards. We know from other patients that they feel such surveys are very important, but you do not have to complete them if you do not want to.

The first questionnaires will be given to you by your hospital. From 6 months onwards questionnaires will be posted to you at your home address by The Institute of Cancer Research Clinical Trials & Statistics Unit (ICR-CTSU).

Before sending you the questionnaire, ICR-CTSU will contact your hospital or GP to check how you are; therefore we would like to ask for your permission to give ICR-CTSU your full name and address as well as your GP's name and address. If you agree to this, please initial the consent form to show that we have your permission.

For peer review only

For peer review only - http://bmjopen.bmj.com/site/about/guidelines.xhtml

2. Donating samples from surgery

RAIDER gives us the opportunity to ask many people with cancer similar to yours whether we can collect samples that will aid future research.

When you were diagnosed bladder cancer, your hospital will have kept a sample of the tumour your surgeon removed during surgery. We would like your permission to collect this and samples which are taken at any future surgery you may have, so that we can look at it in combination with samples from other people who have joined the study. This will allow us to test for genetic differences in the make-up of individuals, indicate why they develop cancer and predict how they react to treatment. If we show that genetic differences do explain why some patients develop bladder cancer or react to their treatment differently, this knowledge could help many patients in the future. Any genetic analysis would be for research purposes only and will not affect any insurance you may hold.

When they are collected from your hospital, the samples will be coded and your personal details will be removed. The coding will maintain your confidentiality whilst allowing biological details to be compared to treatment findings.

The samples collected in this study will be stored indefinitely. It is possible that in the future other research may be carried out on the samples collected within this trial. This research may be conducted in the UK or overseas. Your personal details will not be shared with other researchers. Any future research on samples will be approved by an ethics committee before it is done.

Sample donation is entirely optional and you do not have to participate if you do not wish to. If you chose to join the study there will be a section to complete when you sign the study consent form to indicate if you agree to donate these samples.

For peer review only

For peer review only - http://bmjopen.bmj.com/site/about/guidelines.xhtml

Summary of study assessments

**when→** **what↓**	10 weeks after start of radiotherapy	3 months*	6 months*	9 months*	12 months*	18 months*	2 years	3 years	4 years	5 years
Assessment of side effects	✓	✓	✓	✓	✓	✓	✓	✓	✓	✓
Blood sample		✓	✓							
Cystoscopy under general anæsthetic with biopsy of tumour site		✓								
Cytoscopy under local anæsthetic			✓	✓	✓	✓	✓	✓	✓	✓
Chest x-ray		✓								
CT scan of abdomen & pelvis			✓		✓		✓			
Chest x-ray or CT scan			✓		✓	✓	✓	✓	✓	✓

*after the end of radiotherapy

For peer review only

(Form to be printed on hospital's headed paper)

CONSENT FORM

RAIDER:

A Randomised phase II trial of Adaptive Image guided standard or Dose Escalated tumour boost Radiotherapy in the treatment of transitional cell carcinoma of the bladder

Ethics Committee Reference: 15/LO/0539

RAIDER trial ID:

Name of Researcher taking consent:

Please write your initials in the box to the right of each statement if you agree, and please sign at the bottom

	Statement	Initials
1.	I confirm that I have read and understand the RAIDER Patient Information Sheet 32f Version 2.0 dated 23/01/2019 for the above study. I have had the opportunity to consider the information, ask questions and have had these answered satisfactorily.	
2.	I understand that my participation is voluntary and that I am free to withdraw at any time without giving any reason, without my medical care or legal rights being affected.	
3.	If I withdraw from the study, I consent to my doctor providing authorised researchers with basic clinical information that would be routinely collected and written in my medical records.	
4.	I understand that sections of any of my medical notes may be looked at by responsible individuals from the research team, from ethics committees, or from the NHS Trust, where it is relevant to my taking part in research. I give permission for these individuals to have access to my records.	
5.	I consent to the Institute of Cancer Research using information held by the NHS and national databases to follow up my health status.	
6.	I agree to my GP being informed of my participation in the study.	
7.	*Data sharing: I grant advance authorisation for the possible future sharing of information collected about me with other organisations, with the understanding that I will not be identifiable from this information (optional).*	

RAIDER consent 32f Version 2.0 23/01/2019
2 pages

For peer review only

8.	*I agree to participate in the side effects questionnaire study. (optional)*	☐
8a.	*I consent to researchers from The Institute of Cancer Research being sent my address and GP contact details.*	☐
8b.	*I consent to researchers from The Institute of Cancer Research contacting my GP to confirm I am fit and well to receive questionnaire booklets to be sent out by post.*	☐
8c.	*I consent to my GP disclosing my health status to researchers from The Institute of Cancer Research*	☐
9.	*I consent to donating routinely collected samples from surgery (optional)*	☐
9a.	*I agree that my tumour tissue samples will be analysed for potential changes in DNA (genetic changes)*	☐
9b.	*I grant advance authorisation for the possible future sharing of information collected about me with other organisations, with the understanding that I will not be identifiable from this information*	☐
9c.	*I grant advance authorisation for possible future research on my stored samples, with the understanding that I will not be identifiable from these samples. I understand that that approval of an ethics committee will be obtained beforehand.*	☐
**10.**	**I agree to take part in RAIDER**	☐

Name of participant	Date	Signature

Name of person taking consent	Date	Signature

1 copy for participant; 1 copy for Principal Investigator; 1 copy to be kept with hospital notes

RAIDER consent 32f
2 pages

Version 2.0 23/01/2019

For peer review only - http://bmjopen.bmj.com/site/about/guidelines.xhtml

SPIRIT
STANDARD PROTOCOL ITEMS: RECOMMENDATIONS FOR INTERVENTIONAL TRIALS

SPIRIT 2013 Checklist: Recommended items to address in a clinical trial protocol and related documents*

Section/item	Item No	Description	Addressed on page number
**Administrative information**			
Title	1	Descriptive title identifying the study design, population, interventions, and, if applicable, trial acronym	p1 manuscript (p1 protocol)
Trial registration	2a	Trial identifier and registry name. If not yet registered, name of intended registry	p7 manuscript (p1 protocol)
	2b	All items from the World Health Organization Trial Registration Data Set	n/a
Protocol version	3	Date and version identifier	V 3.0 dated 23/1/2019 (supplementary material)
Funding	4	Sources and types of financial, material, and other support	p30 manuscript (p36 protocol)
Roles and responsibilities	5a	Names, affiliations, and roles of protocol contributors	p1 manuscript
	5b	Name and contact information for the trial sponsor	p1 manuscript (p1 protocol)
	5c	Role of study sponsor and funders, if any, in study design; collection, management, analysis, and interpretation of data; writing of the report; and the decision to submit the report for publication, including whether they will have ultimate authority over any of these activities	p33-34, 36 protocol

For peer review only

	5d	Composition, roles, and responsibilities of the coordinating centre, steering committee, endpoint adjudication committee, data management team, and other individuals or groups overseeing the trial, if applicable (see Item 21a for data monitoring committee)	p21 manuscript (p33-34, 36 protocol)
**Introduction**			
Background and rationale	6a	Description of research question and justification for undertaking the trial, including summary of relevant studies (published and unpublished) examining benefits and harms for each intervention	p5-6 manuscript (p10-13 protocol)
	6b	Explanation for choice of comparators	p11 protocol
Objectives	7	Specific objectives or hypotheses	p6 manuscript (p13 protocol)
Trial design	8	Description of trial design including type of trial (eg, parallel group, crossover, factorial, single group), allocation ratio, and framework (eg, superiority, equivalence, noninferiority, exploratory)	p7 manuscript (p14 protocol)
**Methods: Participants, interventions, and outcomes**			
Study setting	9	Description of study settings (eg, community clinic, academic hospital) and list of countries where data will be collected. Reference to where list of study sites can be obtained	p15 protocol
Eligibility criteria	10	Inclusion and exclusion criteria for participants. If applicable, eligibility criteria for study centres and individuals who will perform the interventions (eg, surgeons, psychotherapists)	p8 manuscript (p15 protocol)
Interventions	11a	Interventions for each group with sufficient detail to allow replication, including how and when they will be administered	p8-17 manuscript (p23-24 protocol and radiotherapy planning and delivery protocol)
	11b	Criteria for discontinuing or modifying allocated interventions for a given trial participant (eg, drug dose change in response to harms, participant request, or improving/worsening disease)	p15 manuscript (p20, 30 protocol)

For peer review only - http://bmjopen.bmj.com/site/about/guidelines.xhtml

	11c	Strategies to improve adherence to intervention protocols, and any procedures for monitoring adherence (eg, drug tablet return, laboratory tests)	p10, 17-18 manuscript (p25, 32 protocol and additional radiotherapy planning and delivery protocol)
	11d	Relevant concomitant care and interventions that are permitted or prohibited during the trial	p7 manuscript; 24 protocol
Outcomes	12	Primary, secondary, and other outcomes, including the specific measurement variable (eg, systolic blood pressure), analysis metric (eg, change from baseline, final value, time to event), method of aggregation (eg, median, proportion), and time point for each outcome. Explanation of the clinical relevance of chosen efficacy and harm outcomes is strongly recommended	p7 manuscript (p14-15 protocol)
Participant timeline	13	Time schedule of enrolment, interventions (including any run-ins and washouts), assessments, and visits for participants. A schematic diagram is highly recommended (see Figure)	Table 1 manuscript (Table p22 of protocol)
Sample size	14	Estimated number of participants needed to achieve study objectives and how it was determined, including clinical and statistical assumptions supporting any sample size calculations	p19-20 manuscript (p28 protocol)
Recruitment	15	Strategies for achieving adequate participant enrolment to reach target sample size	p29-30 protocol

Methods: Assignment of interventions (for controlled trials)

Allocation:

Sequence generation	16a	Method of generating the allocation sequence (eg, computer-generated random numbers), and list of any factors for stratification. To reduce predictability of a random sequence, details of any planned restriction (eg, blocking) should be provided in a separate document that is unavailable to those who enrol participants or assign interventions	p16, 25 protocol

For peer review only - http://bmjopen.bmj.com/site/about/guidelines.xhtml

Allocation concealment mechanism	16b	Mechanism of implementing the allocation sequence (eg, central telephone; sequentially numbered, opaque, sealed envelopes), describing any steps to conceal the sequence until interventions are assigned	p17,44 protocol
Implementation	16c	Who will generate the allocation sequence, who will enrol participants, and who will assign participants to interventions	p17, 44 protocol
Blinding (masking)	17a	Who will be blinded after assignment to interventions (eg, trial participants, care providers, outcome assessors, data analysts), and how	n/a
	17b	If blinded, circumstances under which unblinding is permissible, and procedure for revealing a participant's allocated intervention during the trial	n/a
**Methods: Data collection, management, and analysis**			
Data collection methods	18a	Plans for assessment and collection of outcome, baseline, and other trial data, including any related processes to promote data quality (eg, duplicate measurements, training of assessors) and a description of study instruments (eg, questionnaires, laboratory tests) along with their reliability and validity, if known. Reference to where data collection forms can be found, if not in the protocol	p34, 36, 40, 47 protocol
	18b	Plans to promote participant retention and complete follow-up, including list of any outcome data to be collected for participants who discontinue or deviate from intervention protocols	p20 protocol
Data management	19	Plans for data entry, coding, security, and storage, including any related processes to promote data quality (eg, double data entry; range checks for data values). Reference to where details of data management procedures can be found, if not in the protocol	p34, 37 protocol
Statistical methods	20a	Statistical methods for analysing primary and secondary outcomes. Reference to where other details of the statistical analysis plan can be found, if not in the protocol	p19-21 manuscript (p31-32 protocol)
	20b	Methods for any additional analyses (eg, subgroup and adjusted analyses)	p19-21 manuscript (p31-32 protocol)
	20c	Definition of analysis population relating to protocol non-adherence (eg, as randomised analysis), and any statistical methods to handle missing data (eg, multiple imputation)	p19 manuscript (p29, 32, 34-35 40-41, 47 protocol)

Methods: Monitoring

Data monitoring	21a	Composition of data monitoring committee (DMC); summary of its role and reporting structure; statement of whether it is independent from the sponsor and competing interests; and reference to where further details about its charter can be found, if not in the protocol. Alternatively, an explanation of why a DMC is not needed	p22 manuscript (p33 protocol)
	21b	Description of any interim analyses and stopping guidelines, including who will have access to these interim results and make the final decision to terminate the trial	p19-20 manuscript (p32-33 protocol)
Harms	22	Plans for collecting, assessing, reporting, and managing solicited and spontaneously reported adverse events and other unintended effects of trial interventions or trial conduct	p21 manuscript (p25-28 protocol)
Auditing	23	Frequency and procedures for auditing trial conduct, if any, and whether the process will be independent from investigators and the sponsor	n/a

Ethics and dissemination

Research ethics approval	24	Plans for seeking research ethics committee/institutional review board (REC/IRB) approval	p21 manuscript (p1, p35 protocol)
Protocol amendments	25	Plans for communicating important protocol modifications (eg, changes to eligibility criteria, outcomes, analyses) to relevant parties (eg, investigators, REC/IRBs, trial participants, trial registries, journals, regulators)	p2 protocol
Consent or assent	26a	Who will obtain informed consent or assent from potential trial participants or authorised surrogates, and how (see Item 32)	p16, 35 protocol
	26b	Additional consent provisions for collection and use of participant data and biological specimens in ancillary studies, if applicable	p36-37 protocol
Confidentiality	27	How personal information about potential and enrolled participants will be collected, shared, and maintained in order to protect confidentiality before, during, and after the trial	p35-36 protocol
Declaration of interests	28	Financial and other competing interests for principal investigators for the overall trial and each study site	p30 manuscript

For peer review only - http://bmjopen.bmj.com/site/about/guidelines.xhtml

Access to data	29	Statement of who will have access to the final trial dataset, and disclosure of contractual agreements that limit such access for investigators	p30 manuscript
Ancillary and post-trial care	30	Provisions, if any, for ancillary and post-trial care, and for compensation to those who suffer harm from trial participation	p36 protocol
Dissemination policy	31a	Plans for investigators and sponsor to communicate trial results to participants, healthcare professionals, the public, and other relevant groups (eg, via publication, reporting in results databases, or other data sharing arrangements), including any publication restrictions	p36 protocol
	31b	Authorship eligibility guidelines and any intended use of professional writers	p36 protocol
	31c	Plans, if any, for granting public access to the full protocol, participant-level dataset, and statistical code	n/a
**Appendices**			
Informed consent materials	32	Model consent form and other related documentation given to participants and authorised surrogates	Included as supplementary
Biological specimens	33	Plans for collection, laboratory evaluation, and storage of biological specimens for genetic or molecular analysis in the current trial and for future use in ancillary studies, if applicable	p36 protocol

*It is strongly recommended that this checklist be read in conjunction with the SPIRIT 2013 Explanation & Elaboration for important clarification on the items. Amendments to the protocol should be tracked and dated. The SPIRIT checklist is copyrighted by the SPIRIT Group under the Creative Commons "Attribution-NonCommercial-NoDerivs 3.0 Unported" license.

For peer review only

BMJ Open

Protocol for tumour focused dose escalated adaptive radiotherapy for the radical treatment of bladder cancer in a multi-centre phase II randomised controlled trial (RAIDER): radiotherapy planning and delivery guidance

Journal:	*BMJ Open*
Manuscript ID	bmjopen-2020-041005.R1
Article Type:	Protocol
Date Submitted by the Author:	03-Nov-2020
Complete List of Authors:	Hafeez, Shaista; The Institute of Cancer Research, Radiotherapy and Imaging ; Royal Marsden NHS Foundation Trust, Radiotherapy and Imaging<br>Webster, Amanda; National Radiotherapy Trials Quality Assurance Group, Mount Vernon Hospital<br>Hansen, Vibeke; Odense University Hospital, Laboratory of Radiation Physics<br>McNair, Helen; The Institute of Cancer Research, Radiotherapy and Imaging ; Royal Marsden Hospital NHS Trust, Radiotherapy and Imaging<br>Warren-oseni, Karole; The Institute of Cancer Research, Joint Department of Physics ; Royal Marsden Hospital NHS Trust, Joint Department of Physics<br>Patel, Emma; National Radiotherapy Trials Quality Assurance Group, Mount Vernon Hospital<br>Choudhury, A; The University of Manchester, Faculty of Biology, Medicine and Health; Christie NHS Foundation Trust<br>creswell, Joanne; South Tees Hospitals NHS Foundation Trust<br>Foroudi , Farshad ; Austin Health, Department of Radiation Oncology<br>Henry, Ann; University of Leeds; Leeds Teaching Hospitals NHS Trust<br>Kron, Tomas ; Peter MacCallum Cancer Centre<br>McLaren, Duncan ; Western General Hospital<br>Mitra, Anita; University College London Hospitals NHS Foundation Trust<br>Mostafid, Hugh; Royal Surrey County Hospital NHS Foundation Trust, The Stokes Centre for Urology<br>Saunders, Daniel; Nottingham University Hospitals NHS Trust<br>Miles, Elizabeth; National Radiotherapy Trials Quality Assurance Group, Mount Vernon Hospital<br>Griffin, Clare; The Institute of Cancer Research, Clinical Trials and Statistics Unit<br>Lewis, Rebecca; The Institute of Cancer Research, Clinical Trials and Statistics Unit<br>Hall, Emma; The Institute of Cancer Research, Clinical Trials and Statistics Unit<br>Huddart, Robert; The Institute of Cancer Research, Department of Radiotherapy and Imaging; Royal Marsden Hospital NHS Trust, Department of Radiotherapy and Imaging
Primary Subject	Urology

Heading:	
Secondary Subject Heading:	Oncology
Keywords:	ONCOLOGY, Urological tumours < ONCOLOGY, RADIOTHERAPY, Clinical trials < THERAPEUTICS

I, the Submitting Author has the right to grant and does grant on behalf of all authors of the Work (as defined in the below author licence), an exclusive licence and/or a non-exclusive licence for contributions from authors who are: i) UK Crown employees; ii) where BMJ has agreed a CC-BY licence shall apply, and/or iii) in accordance with the terms applicable for US Federal Government officers or employees acting as part of their official duties; on a worldwide, perpetual, irrevocable, royalty-free basis to BMJ Publishing Group Ltd ("BMJ") its licensees and where the relevant Journal is co-owned by BMJ to the co-owners of the Journal, to publish the Work in this journal and any other BMJ products and to exploit all rights, as set out in our licence.

The Submitting Author accepts and understands that any supply made under these terms is made by BMJ to the Submitting Author unless you are acting as an employee on behalf of your employer or a postgraduate student of an affiliated institution which is paying any applicable article publishing charge ("APC") for Open Access articles. Where the Submitting Author wishes to make the Work available on an Open Access basis (and intends to pay the relevant APC), the terms of reuse of such Open Access shall be governed by a Creative Commons licence – details of these licences and which Creative Commons licence will apply to this Work are set out in our licence referred to above.

Other than as permitted in any relevant BMJ Author's Self Archiving Policies, I confirm this Work has not been accepted for publication elsewhere, is not being considered for publication elsewhere and does not duplicate material already published. I confirm all authors consent to publication of this Work and authorise the granting of this licence.

For peer review only

Protocol for tumour focused dose escalated adaptive radiotherapy for the radical treatment of bladder cancer in a multi-centre phase II randomised controlled trial (RAIDER): radiotherapy planning and delivery guidance

Hafeez S[1], Webster A[2], Hansen VN[3], McNair H[1], Warren-Oseni K[1], Patel E[2], Choudhury A[4], Cresswell J[5], Foroudi F[6], Henry A[7], Kron T[8], McLaren D B[9], Mitra A[10], Mostafid H[11], Saunders D[12], Miles E[2], Griffin C[13], Lewis R[13], Hall E[13], Huddart R[1]

[1]Division of Radiotherapy and Imaging, The Institute of Cancer Research and The Royal Marsden NHS Foundation Trust, London, UK.
[2]National Radiotherapy Trials Quality Assurance Group, Mount Vernon Hospital, Northwood, UK
[3]Odense University Hospital, Laboratory of Radiation Physics, Denmark
[4]Faculty of Biology, Medicine and Health, University of Manchester and The Christie NHS Foundation Trust, Manchester, UK
[5]Department of Surgery, South Tees Hospitals NHS Foundation Trust, UK
[6]Department of Radiation Oncology, Austin Health, Melbourne, Australia
[7]University of Leeds and the Leeds Teaching Hospital NHS Trust, Leeds, UK
[8]Department of Physics, Peter MacCallum Cancer Centre, Melbourne, Australia
[9]Western General Hospital, NHS Lothian, Edinburgh, Scotland, UK
[10]University College Hospital, London, UK
[11]The Stokes Centre for Urology, Royal Surrey, Guildford, UK
[12]Nottingham University Hospitals NHS Trust, Nottingham, UK
[13]Clinical Trials and Statistics Unit, The Institute of Cancer Research, London, UK.

Author for correspondence
Dr S Hafeez
The Royal Marsden NHS Foundation Trust
Downs Road
Sutton
Surrey
SM2 5PT
UK
shaista.hafeez@icr.ac.uk

For peer review only

Abstract

Introduction

Daily radiotherapy delivered with radiosensitisation offers patients with muscle invasive bladder cancer (MIBC) comparable outcomes to cystectomy with functional organ preservation. Most recurrences following radiotherapy occur within the bladder. Increasing the delivered radiotherapy dose to the tumour may further improve local control. Developments in image guided radiotherapy have allowed bladder tumour focused 'plan of the day' radiotherapy delivery. We aim to test within a randomised multi-centre phase II trial whether this technique will enable dose escalation with acceptable rates of toxicity.

Methods and analysis

Patients with T2-T4aN0M0 unifocal MIBC will be randomised (1:1:2) between standard/control whole bladder single plan radiotherapy (WBRT), standard dose adaptive tumour focused radiotherapy (SART), or dose escalated adaptive tumour focused radiotherapy (DART). Adaptive tumour focused radiotherapy will use a library of three plans (small, medium, and large) for treatment. A cone beam CT taken prior to each treatment will be used to visualise the anatomy and inform selection of the most appropriate plan for treatment.

Two radiotherapy fractionation schedules (32f and 20f) are permitted. A minimum of 120 participants will be randomised in each fractionation cohort (to ensure 57 evaluable DART patients per cohort).

A comprehensive radiotherapy quality assurance programme including pre-trial and on-trial components is instituted to ensure standardisation of radiotherapy planning and delivery.

The trial has a two-stage non-comparative design. The primary endpoint of stage I is the proportion of patients meeting pre-defined normal tissue constraints in the DART group. The primary endpoint of stage II is late CTCAE grade 3 or worse toxicity aiming to exclude a rate of >20% (80% power and 5% alpha, one sided) in each DART fractionation cohort. Secondary endpoints include loco-regional MIBC control, progression-free survival overall survival, and patient reported outcomes.

Ethics and dissemination

For peer review only - http://bmjopen.bmj.com/site/about/guidelines.xhtml

The trial is approved by the London-Surrey Borders Research Ethics Committee, (15/LO/0539). The results will be disseminated via peer- reviewed scientific journals, conference presentations and submission to regulatory authorities.

Trial registration number NCT02447549.

Keywords

muscle invasive bladder cancer, image guided adaptive radiotherapy, randomised controlled trial, phase II

For peer review only

Strengths and limitations of this study

- Phase II international multi-centre randomised controlled study evaluating a novel adaptive radiotherapy technique (strength).
- Treatment allocation favours 75% of participants receiving novel adaptive radiotherapy techniques (strength).
- Detailed guidance and training are provided for the contouring, planning, and delivery of this radiotherapy technique to ensure standardisation across participating centres with robust pre-trial and on trial radiotherapy quality assurance programme (strength).
- Primary endpoint focus is based on determining safety of treatment based on late grade 3 toxicity scoring (strength).
- Non-comparative trial design (limitation).

For peer review only

Article summary

We present the first international randomised controlled trial protocol evaluating a dose escalated tumour focused image guided adaptive radiotherapy technique. The study population are patients with unifocal localised muscle invasive bladder cancer. Patients will be randomised (1:1:2) between standard (control) whole bladder single plan radiotherapy (WBRT), or standard dose adaptive tumour focused radiotherapy (SART), or dose escalated adaptive tumour focused radiotherapy (DART). For those randomised to adaptive tumour focused radiotherapy groups treatment will be delivered using a library of three plans (plan of the day). If successful, the trial will demonstrate feasibly of multicentre implementation of this new radiotherapy technique and inform design of a future phase III trial to establish the optimum organ preserving treatment option for patients with MIBC.

For peer review only

Introduction

Radical management of localised muscle invasive bladder cancer (MIBC) involves either radical cystectomy or a course of daily radiotherapy delivered with radiosensitisation over 4-7 weeks [1-5]. Although both have comparable overall survival outcomes in appropriately selected patients, radiotherapy offers opportunity for cancer cure with functional organ preservation [6].

Most recurrences following radiotherapy occur within the bladder, the majority of which are believed to occur at the original MIBC tumour site, suggesting persistent occult local disease [7]. The modelled dose response relationship of MIBC to radiotherapy suggests improved local control and overall survival would be expected at higher doses [8-10].

The ability to safely increase dose beyond the current accepted standard has been restrained by reliable radiotherapy delivery to the bladder. The bladder is a mobile organ which is subject to marked shape and volume change during the course of treatment [11-13]. This bladder motion means historically up to 57% of fractions (f) incur some element of geographical miss even when safety margins of up to 1.5cm are applied to create the planning target volume (PTV) [14]. The expected consequence of improving bladder radiotherapy targeting would be improved tumour control and reduced treatment related toxicity.

Optimisation of target coverage has been enabled by technology integrated on current generation linear accelerators which allow a 3D image known as a cone beam CT (CBCT) to be acquired. This is of sufficient contrast to allow soft tissue visualisation. When acquired immediately prior to treatment it informs positional adjustment to ensure coverage of target with the radiotherapy plan [15].

A solution enabled by CBCT soft tissue visualisation is ‘plan of the day’. Rather than having a single plan available for treatment, a library of plans of varying PTV bladder sizes can be created to cover the range of expected filling and positional variation of the bladder. A plan which best fits the bladder target with least normal tissue irradiation as seen on CBCT immediately prior to treatment is then selected for use each day [14]. In bladder cancer radiotherapy treatment delivery based on a library of plans has reported benefit in reducing normal tissue irradiation compared to single plan treatment delivery [16-19]. It is yet to be demonstrated whether this approach translates to improved clinical outcomes.

For peer review only

Tumour focused radiotherapy delivery may offer further opportunity to reduce normal tissue irradiation. Sparing the uninvolved bladder does not appear to compromise local control but randomised controlled studies have failed to demonstrate statistically significant improvement in toxicity [20, 21]. Bladder sparing is unlikely to have been optimally achieved in radiotherapy delivery pre-dating CBCT image guidance given the positional uncertainties, the large margins applied, and treatment delivery on an empty bladder.

In a single centre phase I study (NCT01124682) feasibility and safety of tumour focused dose escalation to 70Gy delivered using plan of the day has been demonstrated. The RAIDER trial seeks to examine feasibility of this approach in a multi-centre setting and to determine the clinical benefit of bladder tumour focused dose escalation.

Below, we describe the RAIDER trial protocol with particular emphasis on the radiotherapy procedural aspects, including preparatory imaging, treatment planning, and delivery with the aim of providing comprehensive description of the radiotherapy implemented for the study.

Hypothesis

Tumour focused dose escalated adaptive radiotherapy using library of three plans can be translated to multiple centres. It will be well tolerated and offer the opportunity to improve local disease control for bladder cancer patients.

For peer review only - http://bmjopen.bmj.com/site/about/guidelines.xhtml

Materials and analysis

Study design

RAIDER is an international multicentre, multi-arm, two-stage non-blinded phase II randomised controlled trial conducted in accordance with the Research Governance Framework for Health and Social Care and principles of Good Clinical Practice. The trial is registered on the clinicaltrials.gov database (NCT02447549) and is included in the National Institute for Health Research (NIHR) Clinical Research Network portfolio. The final ethics approved version of RAIDER trial protocol is provided in the supplementary files (Appendix 1).

Patients will be randomised (1:1:2) between standard (control) whole bladder single plan radiotherapy (WBRT), standard dose adaptive tumour focused radiotherapy (SART), or dose escalated adaptive tumour focused radiotherapy (DART). Treatment allocation is by minimisation with a random element; balancing factors will be centre, neo-adjuvant chemotherapy use and concomitant radiosensitising therapy use. Randomisation will take place centrally by the trials unit (ICR-CTSU) within a maximum of 10 weeks prior to the planned radiotherapy start date.

Within the UK there are two commonly used radiotherapy schedules to treat bladder cancer, both supported by the National Institute of Clinical for Health and Clinical Excellence (NICE)[1]. Therefore, to accommodate this practice radiotherapy will be delivered daily in either 20f over 4 weeks or 32f over 6.5 weeks in accordance with the participating centre's standard practice. The choice of fractionation will be confirmed by each site before trial commencement and will be used for all patients at that site. The two fractionation cohorts will be analysed separately for the primary end point.

For stage I, the primary endpoint is the proportion of participants in the DART group meeting the pre-defined normal tissue radiotherapy dose constraints. The secondary endpoints of stage I are recruitment rate and the ability of the participating centres to deliver SART and DART treatment as per protocol.

For stage II, the primary endpoint is grade 3 or greater toxicity occurring 6-18 months following radiotherapy as assessed using Common Terminology Criteria for Adverse Events (CTCAE v.4). The secondary endpoints of stage II are acute toxicity as measured by CTCAE v.4, patient reported outcomes as measured by a number of instruments including the Patient-Reported Outcomes version of the Common

For peer review only

Terminology Criteria for Adverse Events (PRO- CTCAE™), Assessment of Late Effects of RadioTherapy - Bowel (ALERT-B), the King's Health Questionnaire (KHQ), sexual function questions and the 5-level EQ-5D version (EQ5D-5L). Additional secondary endpoints include health economic related measures, loco-regional MIBC control, progression free survival, and overall survival.

The trial has a number of exploratory secondary endpoints related to use of adaptive plans including appropriate identification of plan selection, target coverage, and dose volume comparison between control (WBRT) and adaptive (SART and DART) planning.

Figure 1 shows the trial schema and overview of follow-up. Table 1 provides summary of the scheduled pre-randomisation, on treatment, and post treatment assessments.

Participants and eligibility

Total target recruitment is set at a minimum of 240 participants with a minimum 120 be recruited to each fractionation cohort (20f or 32f cohort). The final sample size in each fractionation cohort will be determined as that sufficient to accrue 57 DART patients evaluable for the primary endpoint of late toxicity.

Patients with histological or cytological confirmation of unifocal (T2-T4aN0M0) transitional cell carcinoma (TCC) of the bladder suitable for radical daily radiotherapy will be approached for inclusion. Eligible patients should be willing to accept assessment with cystoscopy and follow-up schedule as outlined in Table 1.

Patients with multi-focal invasive disease or history of other malignancy within 2 years of randomisation except for non-melanomatous skin carcinoma, previous non-muscle invasive bladder tumours, and low risk prostate cancer (as defined by NCCN risk stratification as T1/T2a, Gleason 6 PSA <10) will be excluded. Those with bilateral prosthetic hip replacements, previous history of radiation to the pelvis or other contra-indication to pelvic radiotherapy e.g. inflammatory bowel disease will also be excluded.

Study treatment

All participants should have had a trans urethral resection of the bladder tumour (TURBT) with completion of a bladder map by the performing urologist to aid tumour

For peer review only - http://bmjopen.bmj.com/site/about/guidelines.xhtml

localisation for radiotherapy. Insertion of fiducial markers to further assist tumour localisation for radiotherapy is also recommended at the time of cystoscopy. Neo-adjuvant chemotherapy use prior to randomisation is permitted and encouraged for suitable patients.

Radiotherapy should be planned to commence within a maximum of 10 weeks after randomisation or neo-adjuvant chemotherapy completion (if used), to allow sufficient time for treatment planning.

Delivery of radiotherapy with concomitant radiosentiser is permitted. Regimes approved for use within the protocol include mitomycin C and 5FU [2], gemcitabine [22], cisplatin [23], or carbogen [3]. Each centre should aim to use the same regimen for all their participants. Where this is not possible appropriate substitution is permitted for that participant following discussion with the RAIDER lead investigators.

Participants allocated to the WBRT (control) group will have one radiotherapy plan created treating the whole empty bladder to either 64Gy in 32f or 55Gy in 20f. A CBCT scan acquired just prior to treatment delivery can be used by the local investigators to inform an online position correction in accordance with National Radiotherapy Implementation Group Report on Image Guided Radiotherapy (IGRT) [15] and standard local practice.

Participants allocated to the adaptive tumour focused planning groups (SART and DART) will have three radiotherapy plans generated a small, medium, and large plan. The bladder tumour boost volume will be treated to either standard dose (64Gy in 32f or 55Gy in 20f) or escalated dose (70Gy in 32f or 60Gy in 20f) depending on whether the participant is allocated to SART or DART respectively. The uninvolved bladder will receive a lower planned dose either 52Gy/32f or 46Gy/20f depending on fractionation cohort irrespective of SART or DART randomisation. A CBCT taken immediately prior to each treatment delivery will be used to select the most appropriate ‘plan of the day’ depending on the bladder volume and shape. A second trained individual verifies the plan selected for treatment.

Plan selection is authorised to be carried out only by radiographers or other delegated practitioners who have attained concordance with the gold standard PTV selection through the Radiotherapy Trials Quality Assurance Group (RTTQA) IGRT credentialing for UK centres and Trans Tasman Radiation Oncology Group (TROG)

For peer review only - http://bmjopen.bmj.com/site/about/guidelines.xhtml

IGRT credentialing for Australian and New Zealand centres. This is to ensure all those participating in plan selection have the necessary advanced skill level required for the study.

A comprehensive quality assurance (QA) programme has been implemented for the RAIDER trial. This includes pre-trial and on-trial components. Selection of appropriate treatment plans for the adaptive planning group will be also be independently monitored during patient recruitment as part of the radiotherapy QA process.

Radiotherapy planning and delivery

Radiotherapy planning CT scan

Bladder preparation procedures vary depending on randomisation group. For WBRT, an empty bladder is required. Patients should be asked to abstain from drinking fluids for 30 minutes before the scheduled planning CT scan and are required to void their bladder immediately before the planning CT scan is acquired (CT0).

For both SART and DART groups patients are instructed to void their bladder and then drink 350mls of water. Two planning scans are acquired, the first at 30 minutes following drinking (CT30) and the second 60 minutes following drinking (CT60). No voiding is permitted between the two scans. However, if voiding is unavoidable because of patient discomfort, then only the available CT30 scan is used for planning.

Given bladder deformation can occur with a loaded rectum, all participants should be encouraged to evacuate their bowels of flatus and faeces prior to acquisition of the radiotherapy planning scanning. The use of micro enemas is permitted if it is standard local practice but is not mandated.

All patients will be positioned supine with arms comfortably positioned out of the radiotherapy field using appropriate immobilisation techniques for planning CT scan acquisition. CT slices of $\leq$3 mm thickness will be obtained from at least 4cm above the dome of the bladder to 2cm below the ischial tuberosities. No oral or intravenous contrast is required.

The planning CT scan is exported via DICOM transfer to the radiotherapy treatment planning system for target and organs at risk (OAR) localisation. Bladder filling occurring between CT30 and CT60 scans is determined for those randomised to SART or DART. This is achieved by fusing both CT30 and CT60 data sets and contouring

For peer review only - http://bmjopen.bmj.com/site/about/guidelines.xhtml

the bladder on both scans. If the difference in bladder volume between the two scans is less than 50mls i.e. no significant bladder filling occurs, then all target and OAR contours are created using CT30. If difference in bladder filling is greater than 50mls i.e. bladder filling occurs, the target volumes for large plan is created using CT60 anatomy.

Target volume definition

Volumes will be defined according to the International Commission on Radiation Units and Measurements (ICRU) report 50, supplement report ICRU 62: Prescribing, Recording and Reporting Photon Beam Therapy and ICRU 83: Prescribing, Recording and Reporting Photon- Beam Intensity Modulated radiotherapy (IMRT) [24]. Consistent structure naming convention for target volumes and organs at risk is adopted for all patients participating within the trial.

The gross tumour volume (GTV) is defined as the bladder tumour or the resected tumour bed. It is delineated using position of fiducial markers (where available), diagnostic imaging (prior to neoadjuvant chemotherapy where applicable) and the surgical bladder map (where available). When delineating the tumour any extravesical tumour should be included in the GTV. If no tumour is visible then the appropriate section of the bladder should be included based on surgical bladder map following discussion with the urologist who performed the TURBT. Alternatively, repeating the cystoscopy and placing fiducial markers adjacent to resected bladder tumour scar should be considered.

The clinical target volume (CTV) is contoured to encompass the GTV, the whole bladder, and any area of extravesical spread. The CTV should also include 1.5cm of prostatic urethra in male patients or 1cm of urethra in female patients if tumour is at the base of bladder or if distant carcinoma in situ (CIS) is present.

A checklist for contouring is provided in the radiotherapy planning and delivery guidelines (supplementary file, Appendix 2, p17). The expansions applied to generate the PTVs are summarised in Table 2. The PTV expansion margins were derived from earlier phase I work [14, 16, 25].

Organs at risk delineation

Organs at risk (OARs) are identified as other bowel, rectum, and femoral heads in all groups. To quantify normal bladder sparing, the normal bladder outside the boost

For peer review only - http://bmjopen.bmj.com/site/about/guidelines.xhtml
For peer review only

(PTV2) is also identified for participants in the adaptive tumour focused radiotherapy groups.

All OARs will be outlined as solid structures by defining their outer wall. The rectum is outlined to include the full circumference and rectal contents. The rectal outlining should extend from the lowest level of the ischial tuberosities to the rectosigmoid junction which identified as the level at which there is an anterior inflection of the bowel, best appreciated on sagittal reconstructions on the CT planning scan.

The small and large bowel (including sigmoid colon) will be outlined as a single structure labelled 'other bowel'. Small and large bowel visible on relevant axial slices of the planning scan will be outlined as individual loops. The cranial extent of 'other bowel' outlining should be 2cm beyond the superior extent of the standard PTV or large PTV as appropriate.

Both the femoral heads are outlined to the bottom of the femoral head curvature. The femoral necks not included.

The normal bladder outside the boost (PTV2) is created by subtracting the PTV2 from the corresponding CTV.

Radiotherapy planning

All patients are CT planned. For WBRT, a single plan created using either 3D conformal radiotherapy (3DCRT) with three or four fields, static 5-7-field intensity modulated radiotherapy (IMRT), or volumetric modulated arc radiotherapy (VMAT) technique is permitted. It is accepted that the preferred treatment planning method will vary between participating sites but should be specified in the centre's pre-trial process document and be used for all patients enrolled at that centre. Changes in centres preferred planning method from that specified should be brought to the attention of RTTQA.

For participants in the adaptive tumour focused radiotherapy groups, the planning and dose calculation is done on CT30 data set, therefore all target and OARs volumes are assigned to the CT30 scan. They will have three plans created (small, medium, or large) generated from the respective PTV and PTV2 volumes. To enable bladder sparing, these plans are created using either static 5-7-field IMRT or VMAT. The same

For peer review only - http://bmjopen.bmj.com/site/about/guidelines.xhtml

technique should be used for all patients randomised to adaptive tumour focused radiotherapy at that centre.

The prescription doses for the PTV are outlined in Table 3. All plans should be created with the intention of achieving the target volume objectives as outlined in Table 4. Dose to OARs should be as low possible. The OARs dose volume constraints for both fractionations are summarised in Table 5.

The other bowel, rectum, and femoral heads constraints for the 32f schedule were derived from previous phase III prostate (CHHiP) and bladder (BC2001) studies [2, 26, 27] and from phase I work [28]. The absence of previously defined OARs constraints when dose escalating in 20f meant that the OARs constraints at higher doses were marginally more conservative than if otherwise converted exactly from 32f constraint level using the linear quadratic model alone [29]. The constraints used for the 20f schedule were estimated from the 32f constraint level using the linear quadratic equation assuming that all α/β of organs at risk is 3 but the dose constraint is reached in 3Gy per fraction.

Dose objectives to the PTV should not be compromised to achieve dose to OAR constraints. The recommended hierarchy of planning priorities is provided radiotherapy planning and delivery guidelines (supplementary file, Appendix 2, p27).

For patients randomised to WBRT, it is at the local principal investigator's (PI) discretion to accept the OAR doses. For those randomised to adaptive tumour focused radiotherapy groups it is recommended that the pre-defined optimal dose constraints are met for the small plan, and the mandatory constraints for the medium plan wherever possible. It is accepted that the rectum and bowel dose constraints of the large plan may not be met despite adequate optimisation. Assessment of 'other bowel' dose on the large plan represents an over estimation of actual dose compared to 'other bowel' when this plan is actually used to deliver treatment. This is because when the large plan is selected for treatment, a proportion of bowel moves out of the field with bladder filling.

For patients allocated to DART, if the mandatory constraints are not met on the medium plan advice must be sought from the RTTQA team. Decision will be then made by the RAIDER trial team regarding the appropriateness of proceeding at the DART prescription dose or to lowering the prescribed dose as per SART

For peer review only - http://bmjopen.bmj.com/site/about/guidelines.xhtml

randomisation. It is therefore recommended that the medium plan be optimised first. If patients are not able to receive DART (in either fractionation cohort) for any reason then details of the deviation from allocated treatment will be requested

Pre-radiotherapy checks

To minimise risk of error at the time of plan importing, exporting, and plan selection, it is recommended that each plan, beam name and ID reflect the assigned plan e.g. Sm_Plan used for labelling the beams making up the small plan in the adaptive tumour focused radiotherapy groups. It is also important to ensure that the local record and verify systems for 3DCRT and IMRT cannot mix beams from different plans at the time exporting or deliver more than one plan at treatment.

Treatment scheduling

Radiotherapy can start on any day of the week and should be delivered five days a week until completion. Interruptions during radiotherapy should be avoided as they have detrimental effect on outcome [30]. All missed fractions are to be reported to the ICR-CTSU and RTTQA team.

In the event of missed fractions due to machine breakdown, bank holiday, or any other logistical reason compensation for the missed fraction is advised. This is expected to be achieved by either treating at a weekend or by hyperfractionating i.e. undertaking two fractions a day (ideally on a Friday) with a minimum 6 hour gap between treatments. Should a treatment break occur due to toxicity, centres are advised to contact ICR-CTSU and/or RTTQA. Compensation is not expected in circumstances where missed treatment is a result of radiotherapy related toxicity.

For those allocated to adaptive tumour focused radiotherapy groups if plan selection capabilities are unavailable, either because of absence of trained staff, machine breakdown and/or gap day treatment, patients may be treated for up to 5 days using the PTV medium plan without plan selection. These pre-treatment CBCTs (if acquired) should be sent to RTTQA for review.

Treatment delivery

The same patient preparation instructions used at planning CT should be implemented prior to each fraction delivered.

For peer review only - http://bmjopen.bmj.com/site/about/guidelines.xhtml

For those patients allocated to SART or DART, CBCT of the pelvis should be acquired prior to each fraction. For those patients randomised to WBRT, pre-treatment CBCT should be used in accordance with guidance provided in the NRIG IGRT report [15]. It is therefore expected that this CBCT will inform appropriate corrections (either manual or automatic) to be applied prior to the delivered fraction in accordance with the centre's local practice to ensure that treatment is accurately directed. Any changes made on the basis of the scan including exposures that do not lead to treatment because of patient factors should be reported in the case report forms (CRF) and to RTTQA.

For those randomised to adaptive tumour focused radiotherapy groups, the pre-treatment CBCT is acquired and registered to bone in accordance according to the guidance provided in the NRIG IGRT report [15]. An appropriately trained radiographer or practitioner reviews the bone matched CBCT assessing the bladder size and position in relation to the PTVs and the coverage they provide.

To assist trained radiographers or practitioners with optimal plan selection the following sequential assessment is advised:

i) Following CBCT acquisition, the bladder filling and shape is first checked against CTV_30 contour. If the bladder is of similar size and shape to the CTV at planning (i.e. CTV_30), then the small plan should be considered in the first instance for treatment.

ii) The appropriate plan provides suitable coverage of the CTV and boost region by the corresponding PTV and PTV2 contours with minimal normal tissue irradiation.

iii) Manual (soft tissue) moves should be made to ensure the bladder (CTV) is adequately covered whilst selecting the smallest plan possible to spare normal tissue.

iv) Care should be taken when applying any soft-tissue shifts greater than 1cm as it can impact on the accuracy of the expected dosimetry. If shifts over 1cm occur they should be discussed with the planning department and RTTQA should be contacted following treatment.

For peer review only - http://bmjopen.bmj.com/site/about/guidelines.xhtml

v) Manual moves should be undertaken if further optimisation of PTV2 coverage can be achieved. Manual moves prioritising coverage to the boost region over the normal bladder wall is permitted if it avoids excessive normal tissue irradiation that would have occurred by selecting a larger plan.

vi) Finally, the OARs as seen on the CBCT is reviewed and compared to the position on the planning CT. The position of OARs relative to the boost is assessed to ensure that excessive normal tissue does not sit within the PTV2, especially for DART patients. If this is case, manual move is permitted to minimise normal tissue irradiation but should not be at the expense of target coverage.

vii) A second accredited radiographer or practitioner must confirm selected plan and any additional actions taken. Once agreement has been reached any necessary couch correction is performed prior to treatment delivery with the selected plan.

Fractions must not be omitted or missed due to unfavourable positioning of normal anatomy such as rectal distention due to flatus or faeces. Additional guidance and potential solutions are provided for scenarios that may arise on treatment are given in the radiotherapy planning and delivery guidelines (supplementary file, Appendix 2, p45). The flow chart of potential interventions is derived from phase I experience previously published [28].

For example, if the bladder is significantly smaller than the CTV_30 contour at planning, it is likely that the PTV2 boost will be in the incorrect position and, or does not achieve adequate normal bladder sparing. In these circumstances patients should be removed from the treatment couch, and encouraged to fill the bladder by drinking further, and or increasing the time interval of image acquisition.

In the event that the bladder has over filled and none of the PTVs provide adequate coverage despite manual moves, the patient should be asked to minimally void and the CBCT is repeated. If this is not possible, patient should void completely and restart drinking protocol with a reduction in the time interval for CBCT acquisition. In these circumstances a member of the clinical team should also be notified to ensure the patient is not in urinary retention.

For peer review only - http://bmjopen.bmj.com/site/about/guidelines.xhtml

When amending the drinking protocol to optimise patient's anatomy to fit the existing PTV contours, it is advised that one aspect is changed at a time i.e. interval for CBCT acquisition timing or the amount of water that is drunk. This is so the impact of the intervention can be determined and altered for subsequent fractions as required.

If no PTV contours are suitable to cover the target because of rectal gas, then the patient should be removed from the bed and ask to void. The CBCT image acquisition is then repeated. If the PTV contours still are not optimal, it is recommended that the most suitable plan is selected which optimises coverage of PTV2 and minimises the inclusion of OARs is chosen for treatment. If this occurs repeatedly (e.g. more than twice in 5 fractions) RTTQA should be contacted for advice.

All CBCT exposures including those not resulting in treatment should be recorded on the CRF and plan selection form.

In all randomised groups, a post treatment CBCT should be taken during the first week and once a week thereafter. This CBCT should be reviewed locally to ensure intra fraction filling has been accommodated for at the time of plan selection.

Radiotherapy protocol compliance programme

The RAIDER trial is subject to radiotherapy QA programme that aims to standardise contouring, planning, and delivery of image guided and adaptive bladder radiotherapy in participating centres. The RTTQA Group coordinates the UK QA programme for the study. For Australian and New Zealand participants, this is co-ordinated by the TROG QA Team.

The QA programme has a pre-trial and on-trial component. Each centre will be required to complete the pre-trial QA prior to commencing recruitment.

Prior to trial entry, participating centres will be asked to complete a facility questionnaire in order to gauge current local IGRT experience. A separate process document is used to collect task details of all aspects of a complete patient pathway.

The PI at each participating site is asked to contour two benchmark clinical cases as per protocol. One case includes tumour bed GTV as defined by placement of fiducial markers (radio-opaque contrast agent, lipiodol). UK PIs who completed outlining

For peer review only - http://bmjopen.bmj.com/site/about/guidelines.xhtml

benchmark cases for the preceding phase II adaptive bladder radiotherapy trial (HYBRID Trial, NCT01810757) will be asked to contour only the target volumes as the OARs contouring is unchanged for the RAIDER protocol [31]. Structured feedback to the PI will be provided via RTTQA team.

All participating trial centres will also be required to complete a planning benchmark case. Centres will be provided with access to CT DICOM data and pre-outlined structure set. They will be requested to the plan this patient in their own treatment planning system as if randomised to the DART arm. It is the responsibility of the local investigator to ensure that appropriate plan checking QA process is in place at their local institution. Once the three plans of the benchmark case have been created, reviewed, and accepted by the local PI, the DICOM CT, dose cubes, RTplan, and structure sets are returned to the RTTQA team and structured feedback is provided.

It is a pre-trial requirement that all participating centres have both an established IGRT training programme in place for their radiographers and be utilising CBCT to assess bladder treatment delivery. Trial specific bladder IGRT competency will be completed through an online plan selection training package, and practical workshop [32].

The online plan selection training consists of two practice cases each with 6 CBCTs to work through. Step by step instructions with correct plan selections is provided. Following this, a credentialing assessment consisting of 12 plan selections will be carried out. The plan selections and matched reviews will be assessed by RTTQA and structured feedback provided. Only those who meet minimum threshold of concordance of plan selection as pre-defined by the trial team will be approved for performing RAIDER plan selection. Those who were accredited for plan selection in the HYBRID study [31] or in the TROG 10.1 BOLART trial training (NCT01142102) [33] will not be asked to repeat this assessment.

As part of the on-trial QA, the contouring and planning of at least the first adaptive patient and the first DART will be subject to prospective review by the RTTQA group.

All planning data and treatment delivery data including paired weekly pre- and post treatment CBCTs, registration objects, and treatment forms will be collected and reviewed retrospectively by the RTTQA group to ensure adherence to the RAIDER planning and delivery protocol is maintained. Remote retrospective plan selection review will take place for adaptive radiotherapy patients during the trial.

For peer review only - http://bmjopen.bmj.com/site/about/guidelines.xhtml

Statistical considerations

The primary aim of the study is to evaluate the feasibility (stage I) and safety (stage II) of DART. Control (WBRT) and SART treatment groups are included to enable SART to be carried forward to stage II if dose constraints cannot be met in the DART group and to assess equipoise and feasibility of recruitment for any subsequent phase III trial Prospectively collected contemporaneous toxicity data for WBRT and SART will also allow benchmarking of DART results. Patients are randomised 1:1:2 to maximise information on DART. Recruitment to stage II will continue seamlessly whilst stage I is evaluated, unless advised otherwise by the Independent Data Monitoring Committee (IDMC). Patients recruited in stage I will contribute to analysis of stage II.

The sample size of stage I is based on proportion of patients allocated to DART meeting the pre-defined dose constraints of bladder, bowel, and rectum on the medium plan. A patient in the 32f cohort will be defined as meeting the dose constraints if all mandatory constraints of the following are met for the medium plan: rectum constraints at 50Gy, 60Gy, 65Gy and 70Gy; bladder outside PTV2 at 60Gy and 65Gy; and small bowel at V55, V60, V65, V70 and V74. A patient in the 20f cohort will be defined as meeting the dose constraints if all mandatory constraints of the following are met for the medium plan: rectum constraints at 41.7Gy, 50Gy, 54.2Gy and 58.3Gy; bladder outside PTV2 at 50Gy and 54.2Gy; and bowel at V45.8, V50, V54.2, V58.3 and V61.7.

It is expected that in 80% of DART patients the pre-defined dose constraints of the medium plan to the normal bladder, bowel, and rectum will be met. If this proportion is less than 50% it will be concluded that DART delivery is not feasible. Using an A'Hern single stage design (p0=0.5, p1=0.8, 5% alpha, and 80% power) 18 patients are required in each DART fractionation cohort. If at least 13/18 meet dose constraints it will be concluded that DART treatment is feasible; if dose constraints are not met for 6 or more patients in either fractionation cohort the IDMC will advise on continuation of the trial with the option of dropping the DART arm in one or both fractionation cohorts and continuing to stage II with randomization to WBRT versus SART. Stage I will therefore require a total of 72 patients (36 in each fractionation cohort) randomised 1:1:2 between WBRT, SART and DART.

There are no formal early stopping rules for acute toxicity or efficacy but if after 6 patients have been treated per fractionation cohort, more than 50% of patients

For peer review only

experience acute ≥grade 3 treatment related toxicity, the IDMC would be asked to advise on suitability of continuation.

Stage II has a non-comparative design aiming to rule out an upper limit of any late ≥G3 CTCAE toxicity in each DART fractionation cohort. To be considered evaluable for the primary endpoint of late toxicity a patient must receive at least one fraction of allocated treatment and have at least one toxicity assessment performed between 6 and 18 months after completing radiotherapy. It is expected that the proportion of patients in the control group reporting ≥grade 3 toxicity CTCAE toxicity between 6-18 months post-radiotherapy will be 8% [20]. Again using an A'Hern single stage design (p0 (toxicity free)=0.80, p1=0.92, 5% alpha, and 80% power), 57 patients in each DART fractionation cohort, will allow a >20% ≥grade 3 toxicity CTCAE toxicity to be excluded. If more than 6/57 evaluable DART patients experience ≥grade 3 toxicity in either fractionation cohort, then the late toxicity threshold will be exceeded and on the IDMC's recommendation the trial could either be stopped or the DART arm dropped.

Allowing for 5% non-evaluability for late toxicity by 18 months gives a sample size of 120 patients (30 WBRT, 30 SART, 60 DART) for each fractionation cohort i.e. a total target sample size of 240. The non-evaluability rate will be monitored and, with IDMC endorsement, cohort recruitment will continue until there are 57 evaluable DART patients per cohort. During stage II, following IDMC review, consideration would be given to dropping the WBRT or SART arms, if it was felt sufficient data had accrued for these arms and it would expedite meeting the aims of the trial. The IDMC will also monitor recurrence rates. If an absolute excess of loco-regional recurrence are seen early termination of the trial would be considered.

For stage I, the primary endpoint will be presented as the frequency and percentage of randomised patients able to meet the trial dose constraints in the DART group. For stage II, the primary endpoint will be based on the evaluable population. The proportion of patients with any ≥grade 3 CTCAE toxicity occurring within 6 to 18 months post radiotherapy will be presented for each randomised treatment group together with the 90% one-sided binomial confidence interval (the 90% two-sided confidence interval will also be presented). A sensitivity analysis will be conducted using a per protocol population. The per protocol population will include evaluable patients who received their complete fractionation schedule (either 32f or 20f) according to their randomised allocation group.

For peer review only - http://bmjopen.bmj.com/site/about/guidelines.xhtml

The local control rate at 2 years will be presented by treatment group with a 95% confidence interval. Acute and late toxicity will be summarised by frequencies and proportions at each time point by treatment group. Kaplan-Meier methods will also be used to analyse time to local disease progression and overall survival with data presented by randomised group.

Ethics and dissemination

The trial is approved by the London-Surrey Borders Research Ethics Committee, 15/LO/0539).

The first participant was enrolled in October 2015. The study recruitment is scheduled to complete in Spring 2020. It is expected that the trial will report in 2022, following which the results will be disseminated via peer-reviewed scientific journals, conference presentations and submission to regulatory authorities.

Safety reporting

Data is collected at each trial visit regarding any adverse events graded according to CTCAE v4 criteria on the CRF. The highest grade observed since the last visit should be reported. All serious adverse events (SAEs) occurring from the start of radiotherapy up to 30 days following the last fraction and any radiotherapy related ≥grade 3 events occurring between 6 and 18 months are reported to the ICR-CTSU within 24 hours of the PI becoming aware of the event. SAEs should be followed up until clinical recovery is complete or until the condition has stabilised. Any safety concerns will be reported to the main REC by ICR-CTSU as part of the annual progress report.

Trial monitoring and oversight

A Trial Management Group (TMG) will be set up and will include the Chief Investigator, ICR-CTSU Methodology Lead, co-investigators, identified collaborators, the trial statistician, trial manager, and patient representative.

The ICR-CTSU Urology Radiotherapy Trials Steering Committee (TSC) includes a chairperson not directly involved in the trial, and at least two other independent members who will oversee the RAIDER trial. The TSC will meet annually.

An IDMC will be set up to monitor the progress of the trial and will include at least three independent members, one of whom will be a medical statistician. The Committee's terms of reference, roles and responsibilities will be defined in a charter issued by ICR-

For peer review only - http://bmjopen.bmj.com/site/about/guidelines.xhtml

CTSU. The IDMC will meet in confidence at regular intervals, and at least annually. A summary of findings and any recommendations will be produced following each meeting. This summary will be submitted to the TMG and TSC, and if required, the main REC.

Patient and public involvement

The RAIDER trial has been reviewed and endorsed by patient and carer representatives from the National Cancer Research Institute (NCRI) Consumer Liaison Group and the NCRI Clinical and Translational Radiotherapy Research Group (CTRad) working group. The CTRad consumer group also approved the proposal for randomisation ratio to be weighted towards participants receiving advanced radiotherapy techniques.

Patient and public involvement began at the protocol design and development stage via national and local consumer oversight committee review. This included the NIHR Biomedical Research Centre radiotherapy studies consumer panel at The Institute of Cancer Research and The Royal Marsden NHS Foundation Trust, and the National Cancer Research Institute (NCRI) Bladder Clinical Studies Group, which includes consumer representation.

Patients who had participated in the phase I bladder radiotherapy studies [25, 28] were asked to assess if the burden of involvement required for participation was appropriate. This included review of the patient reported outcomes questionnaires.

The trial patient information sheet and consent form were reviewed by the South West London Cancer Research Network consumer group. Their feedback was adopted and incorporated in to the final version of both documents. Copy of the ethics approved final version of the patient information sheet and consent form are provided in the supplementary files (Appendix 3).

Patient representation on the Trial Management Group advises on day-to-day management of the trial including patient recruitment, and it is expected that they will also participate in dissemination of results via bladder cancer patient groups.

Conclusions

For peer review only - http://bmjopen.bmj.com/site/about/guidelines.xhtml

RAIDER represents the first randomised trial of dose escalated adaptive tumour focused 'plan of the day' radiotherapy and provides a framework for multi-centre implementation of this technique.   It seeks to investigate whether this approach will allow an increase of radiation dose to be delivered to the tumour with acceptable toxicity. Results will inform the design of a future phase III trial to establish the optimum organ preserving treatment option for patients with MIBC.

For peer review only

Figures

Figure 1. Trial schema

Tables

Table 1. Schedule of assessments
Table 2. PTV expansion details
Table 3. Prescription doses
Table 4. Target volume dose objectives
Table 5. Organ at risk dose constraint guide

Supplementary information

Appendix 1. RAIDER trial protocol (Version 3.0; dated 23rd January 2019)

Appendix 2. RAIDER radiotherapy planning and delivery guidelines (Version 4.3, dated 27th January 2020)

Appendix 3. RAIDER patient information sheet and consent form (Version 2.0; dated 27th January 2019)

For peer review only

Table 1. Schedule of assessments

Visit/Assessment	Pre-neoadjuvant chemotherapy (if given)	Pre-randomisation†	Pre-radiotherapy	On treatment	6 weeks after start RT¥	10 weeks after start RT	3 months after end RT	6 months after end RT	9 months after end RT	12 months after end RT	18 months after end RT	24 months after end RT	Annually to 5 years	Annually thereafter	At recurrence/ disease progression
Radiological assessment*	X	X												Disease status and survival	Treatment according to local practice, follow up for disease status and survival
TURBT with completion of bladder map	X	X													
Placement of fiducial markers (optional)	X	X	X[1]												
Assessment of symptoms/toxicity			X[2]	X[2,3]	X[2]	X[2]	X[2]	X[6]	X[6]	X[6]	X[6]	X[6]	X[6]		
Full blood count, urea and electrolytes			X	X[4]			X	X							
PRO questionnaire (if participating)			X	X[5]			X	X[7]		X[7]	X[7]	X[7]			
Rigid cystoscopy and biopsy of tumour bed							X								
Chest x-ray							X								
Flexible cystoscopy								X	X	X	X	X	X		
CT of abdomen and pelvis								X		X		X			
Chest x-ray or CT chest								X		X	X	X	X		
Health resource utilisation				X			X	X		X	X	X			

Footnotes
* Recommended imaging: MRI pelvis, CT chest and abdomen. Minimum acceptable is chest, abdomen, pelvis CT or CT chest and CT urogram † For patients who have not received neo-adjuvant chemotherapy
¥ For patients in the 20f cohort only
1. For patients who received neo-adjuvant chemotherapy
2. CTCAE v.4
3. Weekly on treatment
4. During weeks 1, 4 and 6 (week 6 only if receiving 32f)
5. At last fraction
6. CTCAE v.4 and RTOG
7. Questionnaires administered to UK participants by ICR-CTSU from 6 months onwards

For peer review only

For peer review only - http://bmjopen.bmj.com/site/about/guidelines.xhtml

Table 2. PTV expansion details

Patient Randomisation	CT data set	PTV	CTV to PTV expansion (cm)					PTV2	GTV to PTV2 expansion (cm)				
			Laterally	Anteriorly	Posteriorly	Superiorly	Inferiorly		Laterally	Anteriorly	Posteriorly	Superiorly	Inferiorly
Group 1 Standard Whole Bladder (WBRT)	CT0	PTV	0.8	1.5	1.2	1.5	0.8	Not applicable					
Group 2 and 3 Adaptive Tumour Focused (SART and DART)	CT30	PTV_Sm	0.5	0.5	0.5	0.5	0.5	PTV2_Sm	0.5	0.5	0.5	0.5	0.5
	CT30	PTV_Med	0.5	1.5	1.0	1.5	0.5	PTV2_Med	0.5	1.5	1.0	1.5	0.5
	If CT60-CT30 bladder filling <50mls then apply												
	CT30	PTV_Lar_30	0.8	2.0	1.2	2.5	0.8	PTV2_Lar_30	0.8	2.0	1.2	2.5	0.8
	If CT60-CT30 bladder filling >50mls then apply												
	CT60	PTV_Lar_60	0.5	1.5	1.0	1.5	0.5	PTV2_Lar_60	0.5	1.5	1.0	1.5	0.5

For peer review only

For peer review only - http://bmjopen.bmj.com/site/about/guidelines.xhtml

Table 3. Prescription doses

Patient Randomisation	Volume	32 fraction cohort		20 fraction cohort	
		Dose (Gy)	Dose per fraction (Gy)	Dose (Gy)	Dose per fraction (Gy)
Group 1 WBRT	PTV_Std	64	2	55	2.75
Group 2 SART	PTV2	64	2	55	2.75
	PTV (PTV – PTV2)	52	1.625	46	2.3
Group 3 DART	PTV2	70	2.1875	60	3
	PTV (PTV – PTV2)	52	1.625	46	2.3

Table 4. Target volume dose objectives

Volume	Dose Constraints	Optimal	Mandatory
PTV2	$D_{98\%}$	≥95% of prescribed dose	≥90% of prescribed dose
	*$D_{50\%}$	-	+/- 1% of prescribed dose
	$D_{2\%}$	≤105% of prescribed dose	≤107% of prescribed dose
PTV (PTV – PTV2)	D98%	≥95% of prescribed dose	≥90% of prescribed dose

*Please note that D50% constraint refers only to PTV2. PTV D50% is likely to be exceeded depending on size of PTV2. Therefore no compromise to PTV2 coverage should be made at the expense of achieving D50% PTV constraint.

For peer review only - http://bmjopen.bmj.com/site/about/guidelines.xhtml

Table 5. Organ at risk dose constraint guide

Normal tissue	32 fraction cohort			20 fraction cohort		
	Constraint	Optimal	Mandatory	Constraint	Optimal	Mandatory
**Rectum**	V30Gy<br>V50Gy<br>V60Gy<br>V65Gy<br>V70Gy		80%<br>60%<br>50%<br>30%<br>15%	V25Gy<br>V41.7Gy<br>V50Gy<br>V54.2Gy<br>V58.3Gy		80%<br>60%<br>50%<br>30%<br>15%
**Femoral Heads**	V50Gy		50%	V41.7Gy		50%
**Other Bowel**	V45Gy<br>V50Gy<br>V55Gy<br>V60Gy<br>V65Gy<br>V70Gy<br>V74Gy	116cc<br>104cc<br>91cc<br>73cc<br>23cc<br>0cc<br>0cc	139cc<br>127cc<br>115cc<br>98cc<br>40cc<br>10cc<br>0cc	V37.5Gy<br>V41.7Gy<br>V45.8Gy<br>V50Gy<br>V54.2Gy<br>V58.3Gy<br>V61.7Gy	116cc<br>104cc<br>91cc<br>73cc<br>23cc<br>0cc<br>0cc	139cc<br>127cc<br>115cc<br>98cc<br>40cc<br>10cc<br>0cc
****Whole bladder constraint (i.e. CTV)**	V60Gy<br>V65Gy	50%<br>40% only in DART<br>Otherwise 0% in SART	80%<br>50% only in DART<br>Otherwise 5% SART	V50Gy<br>V54.2Gy	50%<br>40% only in DART<br>Otherwise 0% in SART	80%<br>50% only in DART<br>Otherwise 5% SART
**Body-PTV (Normal Tissue)**	D_{1cc}	≤105% of prescribed dose	≤110% of prescribed dose	D_{1cc}	≤105% of prescribed dose	≤110% of prescribed dose

**Whole bladder (CTV) constraint specified should be used to inform plan optimisation. Bladder outside PTV2 (i.e. CTV-PTV2) meeting these contraints will also be collected for reporting of the primary endpoint.

For peer review only

For peer review only - http://bmjopen.bmj.com/site/about/guidelines.xhtml

Contribution

All authors meet at least of one the criteria recommended by the ICMJE. RH and EH conceived the study design. SH wrote the first draft of the radiotherapy protocol and manuscript. All authors (SH, AW, VNH, HMcN, KWO, EP, AC, JC, FF, AH, TK, DM, AM, HM, DS, EM, CG, RL, EH and RH) were involved in protocol development and contributed to subsequent revisions of the protocol and manuscript.

Competing interest

SH reports non-financial support from Elekta (Elekta AB, Stockholm, Sweden), non-financial support from Merck Sharp & Dohme (MSD), personal fees and non-financial support from Roche outside the submitted work; AC reports grants from National Institute of Health Research Manchester Biomedical Research Centre, grants from Cancer Research, UK, grants from Medical Research Council, UK, grants from Prostate Cancer, UK, grants from Bayer, UK, personal fees from Janssen Pharmaceutical, non-financial support from ASCO, grants and non-financial support from Elekta AB, outside the submitted work; AH reports grants from CRUK, grants from MRC, grants from NIHR, outside the submitted work; TK reports reports grants from Cancer Australia , during the conduct of the study; and his group has a research collaborative agreement with Varian Medical System not related to this project; EH reports grants from Cancer Research UK during the conduct of the study; grants from Accuray Inc., grants from Varian Medical Systems Inc., outside the submitted work; RH reports non-financial support from Janssen, grants and personal fees from MSD, personal fees from Bristol Myers Squibb, grants from Cancer Research UK, other from Nektar Therapeutics, personal fees and non-financial support from Roche outside the submitted work. AW, KWO, VNH, HM, EP, JC, FF, DM, AM, HM, DS, EM, CG, and RL have no conflicts to disclose.

Funding

The RAIDER trial is funded by Cancer Research UK (CRUK/14/016) with programme grants to support the work of the Clinical Trials and Statistics Unit at The Institute of Cancer Research (ICR-CTSU) (grant number grant number C1491/A15955).

Acknowledgements

SH, KW, HMcN, RL, EH, and RH acknowledge this study represents independent research supported by the National Institute for Health Research (NIHR) Biomedical Research Centre at The Royal Marsden NHS Foundation Trust and The Institute of Cancer Research, London. AC is supported by the NIHR Manchester Biomedical

For peer review only - http://bmjopen.bmj.com/site/about/guidelines.xhtml

Research Centre. The views expressed are those of the author(s) and not necessarily those of the NIHR or the Department of Health and Social Care. The trial was supported by the National Radiotherapy Trials Quality Assurance Team (RTTQA).

Data sharing

No additional data are available as submission relates to trial protocol only.

For peer review only

For peer review only - http://bmjopen.bmj.com/site/about/guidelines.xhtml

References

1. **National Institute for Health and Clinical Excellence (NICE) Guidance. Bladder cancer: diagnosis and management** http://wwwniceorguk/guidance/ng2/evidence/full-guideline-3744109 2015.
2. James ND, Hussain SA, Hall E, Jenkins P, Tremlett J, Rawlings C, Crundwell M, Sizer B, Sreenivasan T, Hendron C *et al*: **Radiotherapy with or without chemotherapy in muscle-invasive bladder cancer**. *N Engl J Med* 2012, **366**(16):1477-1488.
3. Hoskin PJ, Rojas AM, Bentzen SM, Saunders MI: **Radiotherapy with concurrent carbogen and nicotinamide in bladder carcinoma**. *J Clin Oncol* 2010, **28**(33):4912-4918.
4. Bellmunt J, Orsola A, Leow JJ, Wiegel T, De Santis M, Horwich A, Group EGW: **Bladder cancer: ESMO Practice Guidelines for diagnosis, treatment and follow-up**. *Ann Oncol* 2014, **25 Suppl 3**(suppl 3):iii40-48.
5. Witjes JA, Lebret T, Comperat EM, Cowan NC, De Santis M, Bruins HM, Hernandez V, Espinos EL, Dunn J, Rouanne M *et al*: **Updated 2016 EAU Guidelines on Muscle-invasive and Metastatic Bladder Cancer**. *European Urology* 2017, **71**(3):462-475.
6. Hafeez S, Horwich A, Omar O, Mohammed K, Thompson A, Kumar P, Khoo V, Van As N, Eeles R, Dearnaley D *et al*: **Selective organ preservation with neo-adjuvant chemotherapy for the treatment of muscle invasive transitional cell carcinoma of the bladder**. *Br J Cancer* 2015, **112**(10):1626-1635.
7. Zietman AL, Grocela J, Zehr E, Kaufman DS, Young RH, Althausen AF, Heney NM, Shipley WU: **Selective bladder conservation using transurethral resection, chemotherapy, and radiation: management and consequences of Ta, T1, and Tis recurrence within the retained bladder**. *Urology* 2001, **58**(3):380-385.
8. Majewski W, Maciejewski B, Majewski S, Suwinski R, Miszczyk L, Tarnawski R: **Clinical radiobiology of stage T2-T3 bladder cancer**. *Int J Radiat Oncol Biol Phys* 2004, **60**(1):60-70.
9. Steel G: **Basic Clinical Radiobiology.** *Oxford University Press,* 3rd edition; 2002.
10. Pos FJ, Hart G, Schneider C, Sminia P: **Radical radiotherapy for invasive bladder cancer: What dose and fractionation schedule to choose?** *Int J Radiat Oncol Biol Phys* 2006, **64**(4):1168-1173.
11. Fokdal L, Honore H, Hoyer M, Meldgaard P, Fode K, von der Maase H: **Impact of changes in bladder and rectal filling volume on organ motion and dose distribution of the bladder in radiotherapy for urinary bladder cancer**. *Int J Radiat Oncol Biol Phys* 2004, **59**(2):436-444.
12. Lotz HT, Pos FJ, Hulshof MC, van Herk M, Lebesque JV, Duppen JC, Remeijer P: **Tumor motion and deformation during external radiotherapy of bladder cancer**. *Int J Radiat Oncol Biol Phys* 2006, **64**(5):1551-1558.
13. Meijer GJ, Rasch C, Remeijer P, Lebesque JV: **Three-dimensional analysis of delineation errors, setup errors, and organ motion during radiotherapy of bladder cancer**. *Int J Radiat Oncol Biol Phys* 2003, **55**(5):1277-1287.
14. Lalondrelle S, Huddart R, Warren-Oseni K, Hansen VN, McNair H, Thomas K, Dearnaley D, Horwich A, Khoo V: **Adaptive-predictive organ localization using cone-beam computed tomography for improved accuracy in external beam radiotherapy for bladder cancer**. *Int J Radiat Oncol Biol Phys* 2011, **79**(3):705-712.
15. **National Radiotherapy Implementation Group Report Image Guided Radiotherapy (IGRT) Guidance for implementation and use**. https://wwwsororg/sites/default/files/document-

For peer review only - http://bmjopen.bmj.com/site/about/guidelines.xhtml

versions/National%20Radiotherapy%20Implementation%20Group%20Report%20IGRT%20Finalpdf August 2012.
16. McDonald F, Lalondrelle S, Taylor H, Warren-Oseni K, Khoo V, McNair HA, Harris V, Hafeez S, Hansen VN, Thomas K *et al*: **Clinical implementation of adaptive hypofractionated bladder radiotherapy for improvement in normal tissue irradiation**. *Clin Oncol (R Coll Radiol)* 2013, **25**(9):549-556.
17. Vestergaard A, Muren LP, Lindberg H, Jakobsen KL, Petersen JB, Elstrom UV, Agerbaek M, Hoyer M: **Normal tissue sparing in a phase II trial on daily adaptive plan selection in radiotherapy for urinary bladder cancer**. *Acta Oncol* 2014, **53**(8):997-1004.
18. Foroudi F, Wong J, Kron T, Rolfo A, Haworth A, Roxby P, Thomas J, Herschtal A, Pham D, Williams S *et al*: **Online adaptive radiotherapy for muscle-invasive bladder cancer: results of a pilot study**. *Int J Radiat Oncol Biol Phys* 2011, **81**(3):765-771.
19. Tuomikoski L, Collan J, Keyrilainen J, Visapaa H, Saarilahti K, Tenhunen M: **Adaptive radiotherapy in muscle invasive urinary bladder cancer - An effective method to reduce the irradiated bowel volume**. *Radiother Oncol* 2011.
20. Huddart RA, Hall E, Hussain SA, Jenkins P, Rawlings C, Tremlett J, Crundwell M, Adab FA, Sheehan D, Syndikus I *et al*: **Randomized noninferiority trial of reduced high-dose volume versus standard volume radiation therapy for muscle-invasive bladder cancer: results of the BC2001 trial (CRUK/01/004)**. *Int J Radiat Oncol Biol Phys* 2013, **87**(2):261-269.
21. Cowan RA, McBain CA, Ryder WD, Wylie JP, Logue JP, Turner SL, Van der Voet J, Collins CD, Khoo VS, Read GR: **Radiotherapy for muscle-invasive carcinoma of the bladder: results of a randomized trial comparing conventional whole bladder with dose-escalated partial bladder radiotherapy**. *Int J Radiat Oncol Biol Phys* 2004, **59**(1):197-207.
22. Caffo O, Thompson C, De Santis M, Kragelj B, Hamstra DA, Azria D, Fellin G, Pappagallo GL, Galligioni E, Choudhury A: **Concurrent gemcitabine and radiotherapy for the treatment of muscle-invasive bladder cancer: A pooled individual data analysis of eight phase I-II trials**. *Radiother Oncol* 2016, **121**(2):193-198.
23. Coppin CM, Gospodarowicz MK, James K, Tannock IF, Zee B, Carson J, Pater J, Sullivan LD: **Improved local control of invasive bladder cancer by concurrent cisplatin and preoperative or definitive radiation. The National Cancer Institute of Canada Clinical Trials Group**. *J Clin Oncol* 1996, **14**(11):2901-2907.
24. Measurements ICoRUa: **ICRU Report 83 Prescribing, Recording, and Reporting Intensity-Modulated Photon-Beam Therapy (IMRT)**. 2010.
25. Hafeez S, McDonald F, Lalondrelle S, McNair H, Warren-Oseni K, Jones K, Harris V, Taylor H, Khoo V, Thomas K *et al*: **Clinical Outcomes of Image Guided Adaptive Hypofractionated Weekly Radiation Therapy for Bladder Cancer in Patients Unsuitable for Radical Treatment**. *Int J Radiat Oncol Biol Phys* 2017, **98**(1):115-122.
26. Dearnaley D, Syndikus I, Sumo G, Bidmead M, Bloomfield D, Clark C, Gao A, Hassan S, Horwich A, Huddart R *et al*: **Conventional versus hypofractionated high-dose intensity-modulated radiotherapy for prostate cancer: preliminary safety results from the CHHiP randomised controlled trial**. *Lancet Oncol* 2012, **13**(1):43-54.
27. McDonald F, Waters R, Gulliford S, Hall E, James N, Huddart RA: **Defining bowel dose volume constraints for bladder radiotherapy treatment planning**. *Clin Oncol (R Coll Radiol)* 2015, **27**(1):22-29.
28. Hafeez S, Warren-Oseni K, McNair HA, Hansen VN, Jones K, Tan M, Khan A, Harris V, McDonald F, Lalondrelle S *et al*: **Prospective Study Delivering**

For peer review only - http://bmjopen.bmj.com/site/about/guidelines.xhtml

Simultaneous Integrated High-dose Tumor Boost (≤70 Gy) With Image Guided Adaptive Radiation Therapy for Radical Treatment of Localized Muscle-Invasive Bladder Cancer. *International Journal of Radiation Oncology*Biology*Physics* 2016, **94**(5):1022-1030.

29. Dale RG: **The application of the linear-quadratic dose-effect equation to fractionated and protracted radiotherapy**. *Br J Radiol* 1985, **58**(690):515-528.
30. Bese NS, Hendry J, Jeremic B: **Effects of prolongation of overall treatment time due to unplanned interruptions during radiotherapy of different tumor sites and practical methods for compensation**. *Int J Radiat Oncol Biol Phys* 2007, **68**(3):654-661.
31. Hafeez S PE, Webster A, Warren-Oseni K, Hansen V, McNair H, Miles E, Lewis R, Hall E, Huddart R: **Protocol for hypofractionated adaptive radiotherapy to the bladder within a multicentre phase II randomised trial: radiotherapy planning and delivery guidance**. *BMJ open* 2020, **http://dx.doi.org/10.1136/bmjopen-2020-037134**.
32. Patel E, Tsang Y, Baker A, Callender J, Hafeez S, Hall E, Hansen VN, Lewis R, McNair H, Miles E *et al*: **Quality assuring "Plan of the day" selection in a multicentre adaptive bladder trial: Implementation of a pre-accrual IGRT guidance and assessment module**. *Clinical and Translational Radiation Oncology* 2019, **19**:27-32.
33. Foroudi F, Pham D, Rolfo A, Bressel M, Tang CI, Tan A, Turner S, Hruby G, Williams S, Hayne D *et al*: **The outcome of a multi-centre feasibility study of online adaptive radiotherapy for muscle-invasive bladder cancer TROG 10.01 BOLART**. *Radiother Oncol* 2014, **111**(2):316-320.

For peer review only

Figure 1. Trial schema

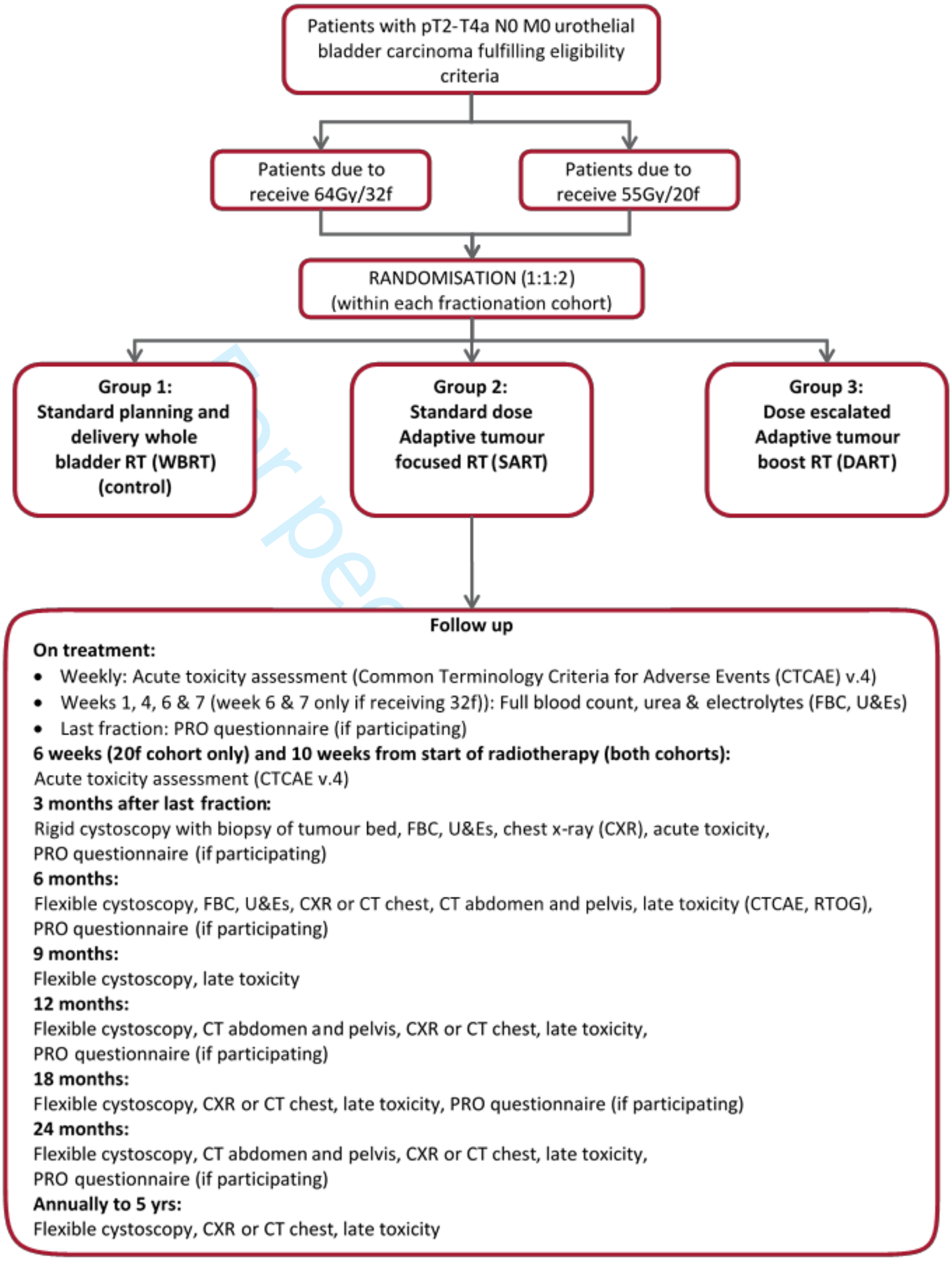

For peer review only - http://bmjopen.bmj.com/site/about/guidelines.xhtml

A Randomised phase II trial of Adaptive Image guided standard or Dose Escalated tumour boost Radiotherapy in the treatment of transitional cell carcinoma of the bladder

PROTOCOL

Version: 3.0

Dated: 23/01/2019

For peer review only

Chief Investigator:	Prof Robert Huddart
Sponsor:	The Institute of Cancer Research
Approval:	Cancer Research UK: Clinical Trials Awards and Advisory Committee (CTAAC)
Funders:	Cancer Research UK
Coordinating Trials Unit:	ICR Clinical Trials and Statistics Unit (ICR-CTSU)<br>The Institute of Cancer Research

Main REC Reference Number:	15/LO/0539
ICR-CTSU Protocol Number:	ICR-CTSU/2014/10049
Sponsor's identifier	CCR4249
ISRCTN:	26779187
ClinicalTrials.gov Identifier	NCT02447549
CRUK Reference Number:	CRUK/14/016

The RAIDER trial has been scientifically approved
by Cancer Research UK's Clinical Trials Awards & Advisory Committee (CTAAC)
The RAIDER trial is part of the National Institute for
Health Research Clinical Research Network Trial Portfolio

This protocol is a controlled document and should not be copied, distributed or reproduced without the written permission of the ICR-CTSU. This document should only be used for the management of patients in the RAIDER trial.

For peer review only - http://bmjopen.bmj.com/site/about/guidelines.xhtml

ADMINISTRATION

Clinical/Scientific Coordination

Prof Robert Huddart (Chief Investigator)
Academic Radiotherapy/Division of Radiotherapy and Imaging
The Royal Marsden NHS Foundation Trust/The Institute of Cancer Research
London
UK
Tel: 0208 661 3457
Robert.Huddart@icr.ac.uk

Dr Shaista Hafeez (Clinical Coordinator)
The Royal Marsden NHS Foundation Trust/The Institute of Cancer Research
London
UK

Tel: 0208 661 3274
Shaista.Hafeez@icr.ac.uk

Dr Farshad Foroudi (TROG lead)
Director Radiation Oncology
Austin Health
Melbourne
Australia

Trial Coordination

ICR-CTSU (a UKCRC registered NCRI cancer clinical trials unit) is responsible for the day to day conduct of the trial in the UK.

ICR Clinical Trials and Statistics Unit (ICR-CTSU),
Division of Clinical Studies,
The Institute of Cancer Research,
Sir Richard Doll Building,
Cotswold Road,
Sutton, Surrey SM2 5NG

ICR-CTSU Scientific/Methodology Lead: Emma Hall
Tel: 020 8722 4013
Emma.Hall@icr.ac.uk

ICR-CTSU Statistician: Clare Griffin
Tel: 0208 722 4187
RAIDER-icrctsu@icr.ac.uk

ICR-CTSU Clinical Trials Programme Manager: Rebecca Lewis
Tel: 020 8722 4081
RAIDER-icrctsu@icr.ac.uk

RAIDER Trial Manager
Tel: 020 8722 4613
RAIDER-icrctsu@icr.ac.uk

ICR-CTSU Data Manager: Chisomo Mutafya
Tel: 020 8722 4513
Chisomo.Mutafya@icr.ac.uk

Any questions relating to this protocol should be addressed in the first instance to the RAIDER Trial Manager within ICR-CTSU:

Email: RAIDER-icrctsu@icr.ac.uk
General enquiries: 020 8722 4613
Fax: 020 8770 7876

For peer review only

For peer review only - http://bmjopen.bmj.com/site/about/guidelines.xhtml

PROTOCOL DEVELOPMENT GROUP

Dr Robert Huddart	Chief Investigator	The Institute of Cancer Research/Royal Marsden NHSFT
Dr Emma Hall	Deputy Director (Research)	ICR-CTSU, The Institute of Cancer Research, London
Dr Shaista Hafeez	Consultant Oncologist	Institute of Cancer Research/ Royal Marsden NHSFT
Dr Ananya Choudhury	Consultant Oncologist	The Christie NHS Foundation Trust
Dr Ann Henry	Consultant Oncologist	Leeds University Teaching Hospitals
Dr Duncan McLaren	Consultant Oncologist	Western General Hospital, NHS Lothian
Dr Anita Mitra	Consultant Oncologist	University College Hospital, London
Dr Daniel Saunders	Consultant Oncologist	Nottingham University Hospitals NHS Trust
Mr Hugh Mostafid	Consultant Urologist	Hampshire Hospitals NHS Trust
Miss Joanne Cresswell	Consultant Urological Surgeon	South Tees Foundation Hospitals Trust
Dr Farshad Foroudi	Consultant Oncologist; ANZ Trans-Tasman Radiation Oncology Group Trial Chairperson	Austin Health, Melbourne
Dr Vibeke Hansen	Physicist	Royal Marsden NHS Foundation Trust
Prof Tomas Kron	Medical Physicist	Peter MacCallum Cancer Centre
Dr Helen McNair	Research Radiographer	Royal Marsden NHS Foundation Trust
Elizabeth Miles	NCRI Radiotherapy Trials QA Group (RTTQA) Co-ordinator	Mount Vernon Hospital
Emma Parsons	RTTQA Radiographer	Mount Vernon Hospital
Rebecca Lewis	Clinical Trials Programme Manager	ICR-CTSU, The Institute of Cancer Research, London
Clare Griffin	Medical Statistician	ICR-CTSU, The Institute of Cancer Research, London

The Trial Management Group (TMG) will be constituted from members of the Protocol Development Group and Principal Investigators from a subset of participating centres. A copy of the current membership of the TMG can be obtained from the RAIDER Trial Manager at ICR-CTSU.

Protocol Authorised by:

Name and Role	Signature	Date
Prof Robert Huddart<br>(Chief Investigator)		23/01/2019

This protocol describes the RAIDER trial and provides information about procedures for entering participants into this trial. The protocol should not be used as a guide for the treatment of patients outside of this trial.

Every care was taken in the preparation of this protocol, but corrections or amendments may be necessary. Protocol amendments will be circulated to participating sites as they occur, but sites entering patients for the first time are advised to contact ICR-CTSU to confirm they have the most recent version.

For peer review only - http://bmjopen.bmj.com/site/about/guidelines.xhtml

RAIDER Protocol
ICR-CTSU

CONTENTS

1. INTRODUCTION 10
1.1. Background 10
1.1.1. Muscle invasive bladder cancer diagnosis and treatment 10
1.1.2. Challenges to bladder radiotherapy delivery 10
1.1.3. Image guided radiotherapy in bladder cancer 10
1.1.4. Concomitant radiosensitisation 11
1.1.5. Adaptive image guided radiotherapy 11
1.1.6. Tumour focused radiotherapy 12
1.1.7. Dose escalation 12
1.1.8. Tumour delineation – fiducial markers/diffusion weighted MRI 12
1.2. Known risks and benefits of adaptive tumour focused and dose escalated radiotherapy 12
1.2.1. Potential benefits 12
1.2.2. Potential risks 13
1.3. Study rationale 13
2. TRIAL OBJECTIVES 13
2.1. Stage I 13
2.1.1. Primary objective 13
2.1.2. Secondary objectives 13
2.2. Stage II 13
2.2.1. Primary objective 13
2.2.2. Secondary objectives 13
2.3. Exploratory objectives 14
2.3.1. IGRT related 14
3. TRIAL DESIGN 14
4. STUDY ENDPOINTS 14
4.1. Primary endpoint 14
4.1.1. Stage I 14
4.1.2. Stage II 14
4.2. Secondary endpoints 14
4.2.1. Stage I 15
4.2.2. Stage II 15
4.3. Exploratory endpoints 15
4.3.1. IGRT endpoints 15
5. PATIENT SELECTION AND ELIGIBILITY 15
5.1. Number of participants 15
5.2. Source of participants 15
5.3. Inclusion criteria 15
5.4. Exclusion criteria 16
5.5. Lifestyle guidelines 16
6. SCREENING 16
6.1. Screening log 16
6.2. Procedure for obtaining informed consent 16
6.2.1. RAIDER trial consent 17
6.3. Participation in other research 17
7. RANDOMISATION 17
8. TRIAL ASSESSMENTS 17
8.1. Pre-neoadjuvant chemotherapy assessments 17
8.2. Pre-randomisation assessments 18
8.3. Pre-radiotherapy assessments 18
8.4. On-treatment assessments 18
8.4.1. 32 fraction cohort 18

For peer review only - http://bmjopen.bmj.com/site/about/guidelines.xhtml

8.4.2. 20 fraction cohort ... 19
8.5. Post radiotherapy assessments ... 19
8.5.1. 10 weeks from start of radiotherapy ... 19
8.5.2. 3 months from last radiotherapy fraction ... 19
8.5.3. 6 months from last radiotherapy fraction ... 19
8.5.4. 9 months from last radiotherapy fraction ... 19
8.5.5. 12 months from last radiotherapy fraction ... 19
8.5.6. 18 months from last radiotherapy fraction ... 20
8.5.7. 24 months from last radiotherapy fraction ... 20
8.5.8. Annually to year 5 ... 20
8.5.9. Annually thereafter ... 20
8.6. Procedure at disease progression/recurrence ... 20
8.7. Withdrawal from treatment or follow-up ... 20
9. SCHEDULE OF ASSESSMENTS ... 22
10. TREATMENT ... 23
10.1. Pre-trial treatment ... 23
10.2. Neo-adjuvant chemotherapy ... 23
10.3. Treatment timelines ... 23
10.4. Radiotherapy fractionation schedules ... 23
10.5. Radiotherapy planning and delivery ... 23
10.5.1. Group 1: standard Whole Bladder RT (WBRT) (control) ... 23
10.5.2. Group 2: Standard dose Adaptive tumour focused RT (SART) ... 23
10.5.3. Group 3: Dose escalated Adaptive tumour boost RT (DART) ... 24
10.6. Treatment scheduling and gaps ... 24
10.7. Concomitant therapy ... 24
10.8. Supportive care guidelines ... 24
11. RADIOTHERAPY QUALITY ASSURANCE (QA) ... 25
12. SAFETY REPORTING ... 25
12.1. Definitions ... 25
12.2. Reporting adverse events to ICR-CTSU ... 26
12.3. Reporting serious adverse events to ICR-CTSU ... 26
12.4. Serious adverse events exempt from expedited reporting ... 27
12.5. Review of serious adverse events ... 27
12.6. Expedited reporting of related unexpected SAEs ... 27
12.7. Follow up of serious adverse events ... 27
12.8. Annual safety reporting ... 27
12.9. Reporting pregnancies ... 27
12.10. Flow diagram for SAE reporting, and action following report ... 28
13. STATISTICAL CONSIDERATIONS ... 28
13.1. Statistical design and sample size justification ... 28
13.2. Treatment allocation ... 29
13.3. Endpoint definitions ... 30
13.3.1. Primary endpoints ... 30
13.3.2. Secondary endpoints ... 30
13.3.3. Exploratory endpoints ... 31
13.4. Statistical analysis plan ... 31
13.5. Interim analyses and stopping rules ... 32
14. TRIAL MANAGEMENT ... 33
14.1. Trial management group (TMG) ... 33
14.2. Trial steering committee (TSC) ... 33
14.3. Independent data monitoring committee (IDMC) ... 33
15. RESEARCH GOVERNANCE ... 34

For peer review only - http://bmjopen.bmj.com/site/about/guidelines.xhtml

15.1. Sponsor responsibilities ... 34
15.2. Participating site responsibilities ... 34
16. TRIAL ADMINISTRATION AND LOGISTICS ... 34
16.1. Site activation ... 34
16.2. Investigator training ... 34
16.3. Data acquisition ... 34
16.4. Central data monitoring ... 34
16.5. On-site monitoring ... 35
16.6. Completion of the study and definition of study end date ... 35
16.7. Archiving ... 35
17. PATIENT PROTECTION AND ETHICAL CONSIDERATIONS ... 35
17.1. Trial approvals ... 35
17.2. Trial conduct ... 35
17.3. Informed consent ... 35
17.4. Patient confidentiality ... 35
17.5. Data protection act (DPA) ... 36
17.6. Liability ... 36
18. FINANCIAL MATTERS ... 36
19. PUBLICATION POLICY ... 36
20. ASSOCIATED STUDIES ... 36
20.1. Patient reported outcome measures study ... 36
20.2. RAIDER translational sample collection ... 36
A1. WHO PERFORMANCE STATUS ... 38
A2. RTOG/EORTC LATE RADIATION MORBIDITY SCORING SCHEMA ... 38
A3. TUMOUR LOCALISATION GUIDELINES ... 39
A3.1 Fiducial marker placement ... 39
A3.2 Surgical bladder map ... 39
A3.3 Training and quality assurance ... 39
A4. PATIENT REPORTED OUTCOMES STUDY ... 40
A4.1 Background ... 40
A4.2 Hypotheses ... 40
A4.3 Quality of life measures ... 40
A4.4 Study design ... 40
A4.5 Timing of data collection ... 40
A4.6 Compliance ... 40
A4.7 Statistical considerations ... 41
A5. EXPECTED SERIOUS ADVERSE EVENTS ... 42
A6. TRANS TASMAN RADIATION ONCOLOGY GROUP SPECIFIC ADDENDUM ... 43
A6.1 Group specific committees and contacts ... 44
A6.1.1. TROG Trial Coordinating Centre ... 44
A6.2 Randomisation ... 44
A6.3 Safety reporting ... 44
A6.3.1. Reporting serious adverse events to TROG ... 44
A6.4 Review of serious adverse events ... 45
A6.5 Expedited reporting of related unexpected SAEs ... 45
A6.6 Follow up of serious adverse events ... 45
A6.7 Flow diagram for SAE reporting, and action following report ... 46
A6.8 Trial administration and logistics ... 46
A6.8.1. Site activation ... 46
A6.8.2. Investigator training ... 47
A6.8.3. Data acquisition ... 47
A6.8.4. Central data monitoring ... 47

For peer review only - http://bmjopen.bmj.com/site/about/guidelines.xhtml

A6.8.5. On-site monitoring ... 47
A6.8.6. Completion of the study and definition of study end date ... 47
A6.8.7. Archiving ... 47
A6.9 Patient reported outcomes study ... 47
A6.10 Financial matters ... 48
A6.11 Research governance ... 48
A6.11.1. Trial chairperson(s) ... 48
A6.11.2. Trial management committee ... 48
A6.11.3. Principal Investigator ... 48
A6.12 Patient protection and ethical considerations ... 48
A6.12.1. Aboriginal and Torres Strait Islander values and principles ... 48
A6.12.2. Insurance and compensation ... 48
A7. GLOSSARY ... 50
A8. REFERENCES ... 51

For peer review only

For peer review only - http://bmjopen.bmj.com/site/about/guidelines.xhtml

RAIDER TRIAL SUMMARY

PROTOCOL TITLE	A Randomised phase II trial of Adaptive Image guided standard or Dose Escalated tumour boost Radiotherapy in the treatment of transitional cell carcinoma of the bladder
TARGET DISEASE	Muscle invasive bladder cancer
STUDY OBJECTIVES	To define a feasible and safe adaptive dose escalated tumour boost radiotherapy schedule for MIBC; to investigate the ability to deliver daily adaptive bladder radiotherapy and assess the impact of delivery on patient reported outcomes and health economic related measures.
STUDY DESIGN	Multicentre two stage, three arm phase II randomised controlled trial
TRIAL POPULATION	Patients receiving radical radiotherapy for muscle invasive bladder cancer
RECRUITMENT TARGET	Minimum 120 in each of two fractionation cohorts i.e. sufficient to accrue 57 evaluable DART patients per cohort.
TRIAL TREATMENT	Patients will be randomised (1:1:2) between:<br>1. Standard whole bladder radiotherapy delivery (WBRT) (control)<br>2. Standard dose Adaptive tumour focused radiotherapy (SART)<br>3. Dose escalated Adaptive tumour boost radiotherapy (DART)<br>64Gy/32f and 55Gy/20f fractionation schedules are permitted. Participants in all groups will be permitted to receive concomitant radiosensitising therapy. Full blood count (FBC), urea and electrolytes (U&Es) and acute toxicity will be assessed during radiotherapy. Participants in the Patient Reported Outcomes (PRO) sub-study will be asked to complete a questionnaire prior to trial entry and at the end of radiotherapy.
PRIMARY ENDPOINT	Stage I: Proportion of patients meeting radiotherapy dose constraints to bladder, bowel and rectum in DART groups.<br>Stage II: Proportion of patients experiencing any ≥Grade 3 Common Terminology Criteria for Adverse Events (CTCAE) v.4 late toxicity (6-18 months post radiotherapy).
SECONDARY ENDPOINTS	Stage I:<br>• Recruitment rate<br>• Ability to deliver SART and DART<br>Stage II:<br>• Clinician reported acute toxicity<br>• PRO: acute and late bladder and bowel/rectal symptoms;<br>• Health economic related measures: time for outlining, plan generation, selection and delivery, NHS resource usage subsequent to treatment;<br>• Loco-regional MIBC control<br>• Progression-free survival<br>• Overall survival

For peer review only

For peer review only - http://bmjopen.bmj.com/site/about/guidelines.xhtml

EXPLORATORY ENDPOINTS	Image Guided Radiotherapy (IGRT) endpoints:<br>• Use of adaptive plans<br>• Target coverage<br>• Online/offline concordance<br>• Dose volume analysis of adaptive vs. standard planning
FOLLOW UP	Participants will subsequently be assessed at the following intervals:<br>**6 weeks from start of radiotherapy (20f cohort only)**<br>Assessment of acute toxicity (CTCAE v.4)<br>**10 weeks from start of radiotherapy:**<br>Assessment of acute toxicity (CTCAE v.4)<br>**3 months from end of radiotherapy:**<br>Rigid cystoscopy and biopsy of tumour bed, FBC, U&Es, chest x-ray (CXR), acute toxicity (CTCAE), PRO questionnaire (if participating in sub-study).<br>**6 months from end of radiotherapy:**<br>Flexible cystoscopy, FBC, U&Es, CXR or CT chest, CT abdomen and pelvis, late toxicity (CTCAE, RTOG), PRO (if participating in sub-study)<br>**9 months from end of radiotherapy:**<br>Flexible cystoscopy, late toxicity<br>**12 months from end of radiotherapy:**<br>Flexible cystoscopy, CT abdomen and pelvis, CXR or CT chest, late toxicity, PRO (if participating in sub-study)<br>**18 months from end of radiotherapy:**<br>Flexible cystoscopy, CXR or CT chest, late toxicity, PRO (if participating in sub-study)<br>**24 months from end of radiotherapy:**<br>Flexible cystoscopy, CT abdomen and pelvis, CXR or CT chest, late toxicity, PRO (if participating in sub-study)<br>**Yearly to year 5:** Flexible cystoscopy, CXR or CT chest, late toxicity<br>**Annually thereafter:** Survival and disease status

For peer review only

For peer review only - http://bmjopen.bmj.com/site/about/guidelines.xhtml

TRIAL SCHEMA

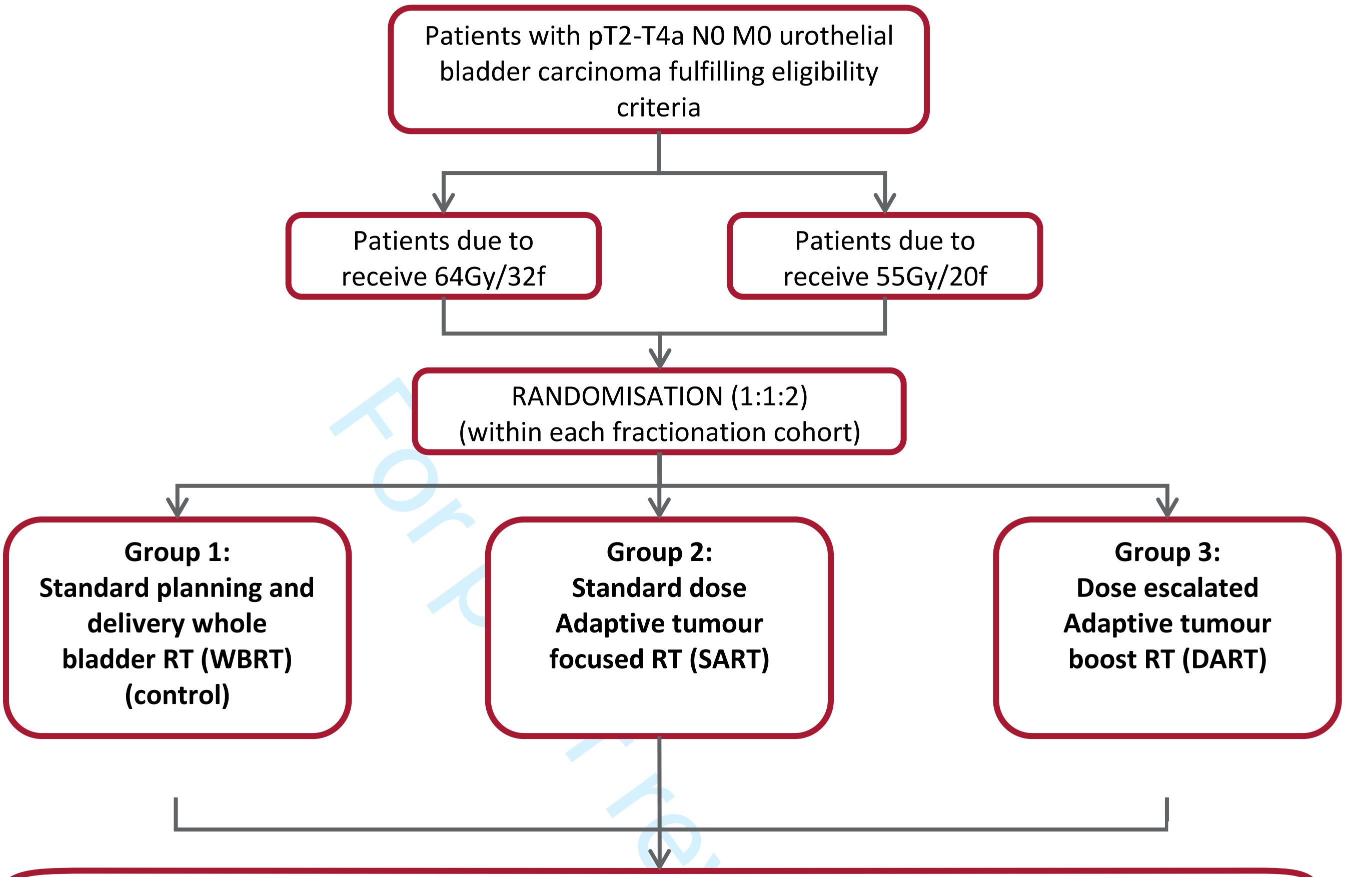

Follow up

On treatment:

- Weekly: Acute toxicity assessment (Common Terminology Criteria for Adverse Events (CTCAE) v.4)
- Weeks 1, 4, 6 & 7 (week 6 & 7 only if receiving 32f)): Full blood count, urea & electrolytes (FBC, U&Es)
- Last fraction: PRO questionnaire (if participating)

6 weeks (20f cohort only) and 10 weeks from start of radiotherapy (both cohorts):
Acute toxicity assessment (CTCAE v.4)

3 months after last fraction:
Rigid cystoscopy with biopsy of tumour bed, FBC, U&Es, chest x-ray (CXR), acute toxicity, PRO questionnaire (if participating)

6 months:
Flexible cystoscopy, FBC, U&Es, CXR or CT chest, CT abdomen and pelvis, late toxicity (CTCAE, RTOG), PRO questionnaire (if participating)

9 months:
Flexible cystoscopy, late toxicity

12 months:
Flexible cystoscopy, CT abdomen and pelvis, CXR or CT chest, late toxicity, PRO questionnaire (if participating)

18 months:
Flexible cystoscopy, CXR or CT chest, late toxicity, PRO questionnaire (if participating)

24 months:
Flexible cystoscopy, CT abdomen and pelvis, CXR or CT chest, late toxicity, PRO questionnaire (if participating)

Annually to 5 yrs:
Flexible cystoscopy, CXR or CT chest, late toxicity

For peer review only - http://bmjopen.bmj.com/site/about/guidelines.xhtml

1. INTRODUCTION

1.1. Background

1.1.1. Muscle invasive bladder cancer diagnosis and treatment

Bladder cancer is the 7th most common UK cancer with 10,399 cases diagnosed in 2011 (1), and the 9th most common cancer in Australia, with an estimated 2,400 cases of muscle invasive disease in 2012 (2). Muscle invasive bladder cancer (MIBC) accounts for 25% of new tumour diagnoses and is associated with poor survival (<50% at 5 years)(3). Radical cystectomy is the "gold standard" therapy for MIBC(4), although a transurethral resection (TURBT) followed by daily radical radiotherapy (RT) is a recommended alternative, with similar rates of disease control to cystectomy. MIBC treatment, whether cystectomy or RT, can have high levels of associated side effects and relatively poor long term survival in comparison to some other cancer sites.

Though historically there have been concerns about high rates of recurrence following RT, the BC2001 trial demonstrated modern chemo-radiation can achieve results comparable to those of cystectomy. Two fractionation regimens are in common use within the UK: 64Gy in 32 fractions (f) over 6½ weeks (also commonly used internationally including in Australia/New Zealand) and 55Gy/20f over 4 weeks. To date these schedules are thought to be similar in efficacy. BC2001 included both 32f and 20f regimens and the 2 year local control rate for patients receiving chemo-radiation was over 65%, with only 18% of patients experiencing invasive recurrence at 2 years(5). These results mean that bladder sparing chemo-radiation is becoming a real alternative to surgery. With further development organ conserving treatment may replace radical surgery, as has been seen in breast, anal and head & neck cancer.

1.1.2. Challenges to bladder radiotherapy delivery

Radiotherapy is becoming accepted as a viable treatment option with good long term outcomes, but high dose radiation exposure can damage normal tissue, causing radiotherapy related toxicity. Patients receiving bladder radiotherapy are at particular risk from small bowel and rectal exposure. Though recent results are encouraging there remains room for improvement in minimizing toxicity*(5)*.

A course of standard radiotherapy is planned using a CT scan taken when the patient has an empty bladder. It is assumed that the initial scan is representative of bladder position throughout the course of treatment and radiotherapy delivery has traditionally been aligned using bony anatomy. To compensate for variations in bladder position, patients are treated with large safety margins added around the empty bladder (clinical target volume (CTV)) to create the planning target volume (PTV) to account for uncertainty introduced by microscopic disease not visible on the CT scan, errors in patient set up and day-to-day variation in bladder filling.

However the bladder is a mobile, deformable structure and bladder volume can vary markedly during a course of radiotherapy, despite delivering treatment to a perceived empty bladder (6-12). Movement of the bladder wall by more than 1.5cm has been documented in up to 60% of patients, resulting in inadequate coverage by radiotherapy fields in 33% of treatments (10). A study at the Royal Marsden Hospital (RMH) (13) reported that up to 57% of treatment may be delivered with some element of geographic miss (where the radiotherapy does not "hit" the tumour volume), despite employing safety margins of 1.5cm around the empty bladder (14). Geographical miss leads to the possibility of reduced tumour control, but larger margins would increase the treated volume and the amount of normal tissue exposed to high dose radiation, potentially leading to increased toxicity.

1.1.3. Image guided radiotherapy in bladder cancer

Recently, image guided RT (IGRT) technology such as cone beam CT imaging (CBCT) has allowed visualisation of soft tissue in the treatment room. Although of lower resolution than the original planning CT scan, these can be used both to match bony anatomy automatically and to visualise bladder position, thus helping to ensure that the PTV is correctly delivered and enabling development of adaptive IGRT to deliver RT with reduced safety margins, sparing normal tissue*(13-16)*. CBCT also allows the highest doses of RT to be reliably

For peer review only - http://bmjopen.bmj.com/site/about/guidelines.xhtml

focused on the tumour using intensity modulated RT (IMRT)*(13)*, allowing the remaining bladder to be treated at a lower dose*(17)*. Data suggest this technique may reduce the risk of genito-urinary toxicity by reducing exposure of normal bladder tissue to high doses of RT*(18)*,*(19, 20)*. Tumour focused RT also provides scope to increase the dose to which the tumour is exposed (dose escalation), whilst minimizing exposure of the remainder of the bladder. Targeted dose escalation has the potential to increase disease control for patients receiving bladder RT without increasing treatment toxicity.

The UK's ability to undertake image guided intensity modulated RT has recently expanded rapidly with all newly purchased RT machines being IGRT capable and IMRT being offered in 48 of 50 RT centres*(21)*. NHS England is prioritising the increase in capacity for delivery of IMRT and IGRT. Given the challenges of delivering RT to the bladder, the UK's National RT Implementation Group guidelines recommend routine use of CBCT to ensure the bladder is adequately targeted. The guidelines also note that the plan of the day adaptive IGRT technique discussed below has the potential to optimise the treatment of bladder cancer for patients*(22)*.

In Australia CBCT is readily available in most radiotherapy centres. TROG 10.01 has demonstrated feasibility of adaptive image guided radiation therapy and in most centres that participated in the trial adaptive image guided radiotherapy is now standard of care for bladder cancer (23).

1.1.4. Concomitant radiosensitisation

The results of the multicentre phase III BC2001 (adding 5FU and mitomycin C to RT) (5) and BCON (hypoxic sensitization with carbogen and nicotinamide)(24) trials strongly suggest that a radiosensitisation approach should be recommended within RAIDER. Addition of low dose gemcitabine to RT has also been shown to achieve excellent local control rates in a phase II trial(25). Cisplatin was shown to be beneficial in the first randomised trial of chemo-radiation(26). There are no comparative data of the superiority of one radiosensitisation approach over another, though a recent paper has suggested the majority of benefit of carbogen is for patients with necrotic tumours(27).

1.1.5. Adaptive image guided radiotherapy

Availability of CBCT has led to the development of adaptive IGRT delivery strategies aimed at maintaining target coverage whilst reducing the amount of normal tissue irradiated. The most commonly described approaches uses a 'plan of the day' strategy where pre-treatment imaging is used to select the 'best fit' plan from a library of pre designed plans.

Selection of the best-fit plan ensures coverage of the CTV whilst minimising exposure of normal tissue in the PTV. Daily imaging with CBCT is required to permit appropriate plan selection based on bladder size and position. Published studies have varied approaches to creating a library of plans(16),(28-31). One study using a 64Gy/32f regimen reported a reduction of 29% in the mean volume of normal tissue irradiated to >45Gy compared to standard delivery bladder RT(16).

Plan of the day is being explored in the treatment of bladder cancer patients receiving weekly RT in the HYBRID trial (ISRCTN18815596). Participants will be randomised between standard and adaptive delivery techniques(32). 3 treatment plans, small, medium and large, will be generated during planning, with the most appropriate plan selected and verified by trained radiographers at time of each treatment delivery(33).

Additionally the Trans-Tasman Radiation Oncology Group (TROG) have completed a multi-centre feasibility study(23) investigating plan of the day adaptive bladder IGRT techniques using on-treatment CBCTs. This study incorporated rigorous RT quality assurance and recruited ahead of proposed timeline, demonstrating that this form of complex treatment delivery is acceptable to bladder cancer patients and a multicentre study is possible. Though in general the study was successful in the generation of acceptable adaptive plans on schedule, it failed to meet its preset goals for 'success' and judged to be not feasible in 31% of patients (due to use of conventional default plan (16%) and post treatment CTV outside PTV (18%))(23). Despite this it is noted that the treatment was well tolerated and the post treatment CTV was only outside the PTV in 5.5% of treatments. This is a substantial improvement over standard care though suggests some adjustment to adaptive protocols may be required.

For peer review only - http://bmjopen.bmj.com/site/about/guidelines.xhtml

1.1.6. Tumour focused radiotherapy

Targeting the highest RT dose to the tumour was investigated in a limited fashion in two UK randomised trials. BC2001 included a comparison of standard full dose whole bladder RT with a tumour focused treatment strategy(34). Bladder sparing in BC2001 was modest as it used a 1.5cm margin around the tumour and patients were treated with an empty bladder. CBCT had not yet been developed and treatment alignment was conducted using bony anatomy. 219 participants joined the RT comparison and no significant differences have been reported in late toxicity; with ~8% G3-4 RTOG toxicity in the tumour focused RT group at 2 years. There was no evidence to suggest an increase in recurrence in the tumour focused RT group. Similar findings were reported in a trial using 20f performed at the Christie NHSFT. Patients were randomised to whole bladder RT or RT to the tumour + margin only (57.5Gy/20f or 50Gy/16f). No significant differences in toxicity or local control were reported, although interpretation is limited due to the modest sample size and different radiation doses used for partial bladder RT(35).

1.1.7. Dose escalation

A single centre dose finding study, IDEAL(36), is investigating whether adaptive IGRT techniques allow tumour focused dose escalation. 54 patients had been treated to June 2014, with 21 receiving 68Gy/34f and 23 having 70Gy/32-35f. 30/54 patients received neoadjuvant chemotherapy prior to joining IDEAL and 41/54 received concurrent radiosensitising chemotherapy. With a median follow up of 18 months, only 2 episodes of G3 urinary toxicity and 1 invasive recurrence in dose escalated patients have been reported. IDEAL's final dose determined the 32f escalated dose in RAIDER.

The Christie trial dose escalated from 52.5Gy/20f to 57.5Gy/20f without evidence of excess toxicity(35). This study, co-investigator consensus and an α/β conversion of the likely dose resulting from IDEAL has been used to define the dose for the 20f dose escalated tumour boost in RAIDER.

1.1.8. Tumour delineation – fiducial markers/diffusion weighted MRI

Tumour delineation can be challenging, especially in those patients whose cancer responds well to neo-adjuvant chemotherapy; however the use of bladder maps (completed by surgeons at the time of TURBT) in combination with imaging was used with success in BC2001 and will be the minimum standard within RAIDER. There are also more advanced techniques of tumour definition now available. Diffusion weighted MRI (DWI) which assesses the mobility of water ions in tissues, is now widely available and used extensively in prostate cancer management. Cancers tend, being more cellular, to have a more restricted pattern of water mobility and can be distinguished from normal tissues. A prospective study at The Royal Marsden has demonstrated this is the case for localised bladder cancer and that DWI tumour definition and assessment of treatment response is highly correlated with results of cystoscopy/cystectomy. A Royal Marsden pilot study of target delineation(37) showed that DWI was a useful adjunct to conventional imaging and may add biological/functional information. 55/79 (69%) of patients had a definable tumour volume on MRI prior to radiotherapy; the remainder having had a complete TURBT with no visible tumour. A DWI defined GTV was around 50% smaller than the anatomically defined volume.

Bladder tumours can also be delineated using fiducial markers implanted at time of TURBT, particularly for those whose tumour is difficult to define radiologically. Initial work was with gold seeds(38) and more recently with Lipiodol (ethiodized oil)(39, 40). Fiducial insertion has proved to be safe and practicable and a similar technique would be recommended for use in RAIDER where possible.

1.2. Known risks and benefits of adaptive tumour focused and dose escalated radiotherapy

1.2.1. Potential benefits

It is anticipated that the use of adaptive radiotherapy techniques will improve the accuracy of treatment for patients in the adaptive groups which should lead to a reduction in side effects resulting in normal tissue exposure. Due to the highest radiotherapy dose being focused on the tumour, the remainder of the bladder will be exposed to lower levels of radiation which may also reduce the genito-urinary side effects experienced

For peer review only - http://bmjopen.bmj.com/site/about/guidelines.xhtml

by patients in the tumour focused groups. In addition, the patients in the dose escalated tumour boost group may benefit from better disease control as a result of the higher radiation exposure.

1.2.2. Potential risks

The toxicity of the dose escalated tumour boost may be higher than anticipated, however the tumour boost dose in both fractionation groups has been informed by the results of the IDEAL study (with α/β corrections to determine 20f dose). The primary endpoint of stage II is related to toxicity and rates will be monitored by the IDMC throughout the trial.

Participants in the SART and DART groups will receive one additional planning CT scan, however risks are anticipated to be minimal as it represents <1% of the RT dose.

Incorrect plan selection and tumour focused radiotherapy may result in increased risk of geographic miss, however appropriate plan selection will be part of the trial training program, will be verified by a 2nd trained observer prior to treatment delivery and will be monitored throughout the trial. In the IDEAL study with appropriate training a 91% on and offline plan concordance has been achieved with D98 post treatment coverage of 98.7%. Although prior studies have not shown that reduced radiation exposure of the uninvolved bladder increases risk of recurrence, patterns of recurrence and recurrence rates in both adaptive groups will be monitored by the IDMC.

1.3. Study rationale

Improving radiotherapy quality is of clear importance in bladder cancer treatment. RAIDER will assess whether adaptive dose escalated radiotherapy techniques developed at single centres can be successfully translated into radiotherapy practice across the UK, Australia and New Zealand and will prospectively assess the potential benefits of these approaches for patients as part of a multicentre international randomised trial.

RAIDER aims to define a feasible and safe RT schedule for MIBC using modern techniques and will include two fractionation cohorts which will be analysed separately but may provide data on the optimum fractionation schedule. RAIDER will seek to investigate whether modern techniques can allow an increase in the dose of RT to which the tumour is exposed and results will inform the design of a future phase III trial to establish the optimum organ preserving treatment option for patients with MIBC.

2. TRIAL OBJECTIVES

2.1. Stage I

2.1.1. Primary objective

The primary objective of stage I is to ensure that the dose escalated (DART) treatment can be planned and delivered at multiple centres within safe dose constraints.

2.1.2. Secondary objectives

Secondary objectives of stage I are to assess the recruitment rate and the ability of centres to deliver daily bladder SART and DART.

2.2. Stage II

2.2.1. Primary objective

Stage II aims to ensure the proportion of patients experiencing severe or medically significant late toxicity as a result of DART treatment is within acceptable limits.

2.2.2. Secondary objectives

Stage II secondary objectives are to assess clinician reported acute toxicity, and patient reported outcomes (PRO) of acute and late bladder and bowel/rectal symptoms. RAIDER will also investigate health economic related measures including time required for outlining, plan generation, selection and delivery and

For peer review only - http://bmjopen.bmj.com/site/about/guidelines.xhtml

healthcare resource usage subsequent to treatment. Disease related objectives include measuring loco-regional MIBC control, progression-free survival and overall survival.

2.3. Exploratory objectives

2.3.1. IGRT related

RAIDER will assess the utilisation of adaptive techniques including how often alternative plans are selected, the selection of appropriate plans and the target coverage and dose volume analysis of adaptive vs standard planning.

3. TRIAL DESIGN

RAIDER is an international multi-centre, multi-arm, two stage non-blinded phase II randomised trial of adaptive tumour focused radiotherapy for bladder cancer.

The trial includes three randomised groups and a 1:1:2 treatment allocation ratio has been used to provide participants with a 75% chance (on average) of receiving a novel radiotherapy technique. Primary endpoints will be assessed in each fractionation cohort separately. Stage I will test feasibility of DART treatment delivery by measuring compliance with dose constraints and stage II will assess late toxicity. The statistical analysis plan includes the flexibility to drop either a fractionation cohort or an experimental treatment group on the advice of the Independent Data Monitoring Committee following completion of stage I. Results will be used to select the RT technique to be employed in future national/international phase III bladder preserving trials.

All patients will receive radical bladder radiotherapy, delivered in either 20 or 32 fractions in accordance with participating centres' standard practice.

Participants allocated to the standard planning group will have one radiotherapy plan generated and this will be used to deliver all treatments, with a cone beam CT scan prior to treatment delivery which can be used by the local investigator to adjust treatment delivery according to local practice.

Participants allocated to Standard dose Adaptive tumour focused RT (SART) will have three radiotherapy plans generated; small, medium and large, with the highest RT dose focused on the tumour, sparing the remaining bladder from full dose radiation. IGRT will be used to select the most appropriate plan of the day.

Participants in the Dose escalated Adaptive tumour boost RT (DART) group will have three radiotherapy plans generated; small, medium and large, with a higher dose than standard targeted at the tumour and the remainder of the bladder treated to the same dose as in the SART group. IGRT will be used to select the most appropriate plan of the day.

Follow up visits will mirror standard practice wherever possible and will take place at 6 weeks (20f cohort only) and 10 weeks (both cohorts) following the start of radiotherapy, 3, 6, 9, 12, 18 and 24 months following the last fraction and annually to five years.

4. STUDY ENDPOINTS

4.1. Primary endpoint

The two fractionation cohorts will be analysed separately for the primary endpoints.

4.1.1. Stage I

- Proportion of participants meeting RT dose constraints in DART group

4.1.2. Stage II

- Late grade 3 or greater toxicity (CTCAE v4) occurring 6-18 months post RT.

4.2. Secondary endpoints

The two fractionation cohorts will be analysed separately and combined for the following secondary endpoints:

For peer review only - http://bmjopen.bmj.com/site/about/guidelines.xhtml

4.2.1. Stage I

- Recruitment rate
- Ability of centres to deliver SART and DART

4.2.2. Stage II

The two fractionation cohorts will be analysed separately and combined for the following secondary endpoints:

- Clinician reported acute toxicity (CTCAE v4)
- Patient reported outcomes (PRO) - acute and late bladder and bowel/rectal symptoms using the Patient-Reported Outcomes version of the Common Terminology Criteria for Adverse Events (PRO-CTCAE™), Assessment of Late Effects of RadioTherapy - Bowel (ALERT-B), the King's Health Questionnaire (KHQ), sexual function questions and the EQ5D-5L
- Health economic related measures - time for outlining, plan generation, selection and delivery, healthcare resource usage subsequent to study treatment

The two fractionation cohorts will be combined for the analyses of the following outcome measures:

- Loco-regional MIBC control
- Progression-free survival
- Overall survival

4.3. Exploratory endpoints

4.3.1. IGRT endpoints

- Use of adaptive plans
- Target coverage
- Online/offline concordance
- Dose volume analysis of adaptive vs. standard planning

5. PATIENT SELECTION AND ELIGIBILITY

5.1. Number of participants

The aim is to recruit a minimum of 120 participants to each fractionation cohort, i.e. sufficient to accrue 57 evaluable DART patients per cohort. In each cohort, at least 30 participants will be included in the standard planning group (control), at least 30 participants will be in the SART group and at least 60 participants will be allocated to the DART group.

5.2. Source of participants

Participants will be recruited from participating sites in the UK and Australia/New Zealand.

5.3. Inclusion criteria

1. Written informed consent
2. Age ≥16 years
3. Histologically or cytologically confirmed transitional cell carcinoma (TCC) of the bladder
4. Unifocal bladder TCC staged T2-T4a N0 M0*
5. Fit to receive a radical course of radiotherapy
6. WHO performance status 0-2 (See Appendix A1)

For peer review only - http://bmjopen.bmj.com/site/about/guidelines.xhtml

7. Willing and able to comply with study procedures and follow up schedule

* Tumour location must be clearly visible on imaging or recorded on a surgical bladder map

5.4. Exclusion criteria

1. Nodal or metastatic disease
2. Multifocal invasive disease
3. Simultaneous TCC in upper tract or urethra
4. Pregnancy
5. Active malignancy within 2 years of randomisation (not including non melanomatous skin carcinoma, previous non muscle invasive bladder tumours, NCCN low risk prostate cancer (T1/T2a, Gleason 6 PSA <10), in situ carcinoma of any site)
6. Bilateral hip replacements
7. Any other conditions that in the Principal Investigator's opinion would be a contra-indication to radiotherapy (e.g. previous pelvic radiotherapy/inflammatory bowel disease)

5.5. Lifestyle guidelines

It is highly unlikely that the patient population included in RAIDER will be at risk of pregnancy or fathering a child. However, if this is a possibility for any individual patient, this should be discussed and the patient should be advised to use barrier protection and avoid conception for 12 months after treatment.

6. SCREENING

6.1. Screening log

All participating centres will be required to keep a detailed log of all patients with muscle invasive bladder cancer who are considered for radical radiotherapy. This log will capture the following information:

- Date patient identified
- Number of patients approached/accepting/declining participation/ineligible
- Screening outcome
- Trial ID (if applicable)
- Reasons for ineligibility / not approaching / declining as applicable

This information will be used to monitor recruitment activity. No patient identifiable data will be collected at this stage.

6.2. Procedure for obtaining informed consent

The Principal Investigator (or designated individual) must ensure that each trial patient is fully informed about the nature and objectives of the trial and associated sub-studies and possible risks associated with participation. No protocol required assessments should be conducted until the appropriate consent form has been signed and dated by both the patient and the Investigator, unless they are performed routinely as part of standard patient care.

Confirmation of the patient's consent and the informed consent process must be documented in the patient's medical notes. A copy of the signed consent form(s) should be provided to the patient and the original retained in the investigator site file, which must be available for verification by ICR-CTSU study staff.

For peer review only

For peer review only - http://bmjopen.bmj.com/site/about/guidelines.xhtml

6.2.1. RAIDER trial consent

Participants should be given the current REC approved main RAIDER patient information sheet for their consideration. Patients should only be asked to consent to the study after they have had sufficient time to consider the trial and the opportunity to ask any further questions.

Patients who consent to RAIDER will be asked to consent to participate in the Patient Reported Outcomes (PRO) sub-study. Patients should be made aware that participation in the PRO sub-study is entirely voluntary. Refusal to participate in the PRO sub-study will not result in ineligibility to participate in the main clinical trial and will not impact the medical care received.

6.3. Participation in other research

Patients who fulfil the eligibility criteria will be given the opportunity to participate in RAIDER even if they have participated in other research prior to recruitment.

Participation in research whilst patients are being treated within RAIDER will be considered on a study by study basis by the Trial Management Group.

7. RANDOMISATION

Patients must be randomised centrally by the trials unit (ICR-CTSU) before trial treatment can commence. Patients should be randomised by telephoning ICR-CTSU on:

020 8643 7150

09.00-17.00 (UK time) Monday to Friday

Randomisation should take place within 10 weeks prior to the planned start date of radiotherapy. If planned radiotherapy timelines fall outside this window the ICR-CTSU should be contacted for advice prior to randomisation.

Treatment allocation will be by minimisation (with a random component). An eligibility and randomisation checklist must be completed prior to randomisation. Patients should only be randomised if sufficient trained and RTTQA accredited staff are available for plan selection in accordance with the RAIDER Radiotherapy Planning and Delivery Guidelines.

The following information will be required at randomisation:

- Name of treating and recruiting hospital, consultant and person randomising patient
- Confirmation that patient has given written informed consent for trial and for any sub-studies
- Confirmation that patient is eligible for the trial by completion of the eligibility checklist
- Patient's full name, hospital number, date of birth, postcode and NHS/CHI number

The caller will be given the patient's unique randomisation number (Trial ID) and treatment allocation (see section 14.2).

ICR-CTSU will send written confirmation of trial entry to the data management contact at the recruiting centre.

8. TRIAL ASSESSMENTS

8.1. Pre-neoadjuvant chemotherapy assessments

Information will be collected about the following assessments for RAIDER participants who have received neo-adjuvant chemotherapy:

- Radiological assessment of muscle invasive bladder cancer, ideally undertaken within 8 weeks prior to the start of neoadjuvant chemotherapy. If imaging was conducted outside the 8 week timeframe, the ICR-CTSU should be contacted for advice prior to randomisation. MRI pelvis and CT chest and

For peer review only - http://bmjopen.bmj.com/site/about/guidelines.xhtml

abdomen is recommended; the minimum acceptable is a chest, abdomen and pelvis CT or CT chest and CT urogram.

- TURBT with completion of bladder map† and optional placement of fiducial markers (if using, see Appendix A3)
- Histological confirmation of transitional cell carcinoma
- Full blood count, urea and electrolytes

† Bladder map not required if tumour is clearly visible on imaging.

Participants may be randomised into RAIDER whilst receiving neoadjuvant chemotherapy. Radiotherapy should be planned to commence within 10 weeks following completion of neo-adjuvant chemotherapy. If planned radiotherapy timelines fall outside this window the ICR-CTSU should be contacted for advice prior to randomisation.

8.2. Pre-randomisation assessments

For patients who have not received neo-adjuvant chemotherapy, the following assessments should be conducted prior to randomisation:

- Radiological assessment of muscle invasive bladder cancer within a maximum of 8 weeks prior to randomisation. If imaging was conducted outside the 8 week timeframe this should be repeated prior to randomisation. MRI pelvis and CT chest and abdomen is recommended; the minimum acceptable is a chest, abdomen and pelvis CT or CT chest and CT urogram.
- TURBT with completion of bladder map† and optional placement of fiducial markers (see Appendix A3)
- Histological confirmation of transitional cell carcinoma

† Bladder map not required if tumour is clearly visible on imaging.

8.3. Pre-radiotherapy assessments

For patients who have received neo-adjuvant chemotherapy the following assessments should be conducted within 4-6 weeks prior to the start of radiotherapy:

- Optional cystoscopy with placement of fiducial markers (if using)

The following assessments should be conducted for all participants within 2 weeks prior to the start of radiotherapy:

- Assessment of baseline symptoms (CTCAE v. 4)
- Full blood count, urea and electrolytes
- For participants who have consented to the patient reported outcomes (PRO) sub-study: Baseline PRO questionnaire (PRO-CTCAE, ALERT-B, KHQ, sexual function and EQ5D-5L)

8.4. On-treatment assessments

8.4.1. 32 fraction cohort

Weekly during treatment:

- Acute toxicity assessment (CTCAE v.4)

During weeks 1, 4 and 6 of radiotherapy:

- Full blood count, urea and electrolytes

At last fraction:

- Patient reported outcomes (PRO-CTCAE, ALERT-B, KHQ, sexual function and EQ5D-5L)

For peer review only - http://bmjopen.bmj.com/site/about/guidelines.xhtml

8.4.2. 20 fraction cohort

Weekly during treatment:

- Acute toxicity assessment (CTCAE v.4)

During weeks 1 and 4 of radiotherapy:

- Full blood count, urea and electrolytes

At last fraction:

- Patient reported outcomes (PRO-CTCAE, ALERT-B, KHQ, sexual function and EQ5D-5L)

6 weeks from start of radiotherapy

- Acute toxicity assessment (CTCAE v.4)

8.5. Post radiotherapy assessments

8.5.1. 10 weeks from start of radiotherapy

- Acute toxicity assessment (CTCAE v.4)

8.5.2. 3 months from last radiotherapy fraction

- Rigid cystoscopy and biopsy of tumour bed
- Full blood count, urea and electrolytes
- Chest x-ray
- Acute toxicity assessment (CTCAE v.4)
- Patient reported outcomes (PRO-CTCAE, ALERT-B, KHQ, sexual function and EQ5D-5L)

8.5.3. 6 months from last radiotherapy fraction

- Flexible cystoscopy
- Full blood count, urea and electrolytes
- CT of abdomen and pelvis
- Chest x-ray or CT chest
- Late toxicity assessment (CTCAE v.4 and RTOG (see Appendix A2))
- Patient reported outcomes (PRO-CTCAE, ALERT-B, KHQ, sexual function and EQ5D-5L). (Questionnaire administered to UK participants by ICR-CTSU.)

8.5.4. 9 months from last radiotherapy fraction

- Flexible cystoscopy
- Late toxicity assessment (CTCAE v.4 and RTOG (see Appendix A2))

8.5.5. 12 months from last radiotherapy fraction

- Flexible cystoscopy
- CT of abdomen and pelvis
- Chest x-ray or CT chest
- Late toxicity assessment (CTCAE v.4 and RTOG (see Appendix A2))
- Patient reported outcomes (PRO-CTCAE, ALERT-B, KHQ, sexual function and EQ5D-5L). (Questionnaire administered to UK participants by ICR-CTSU.)

For peer review only - http://bmjopen.bmj.com/site/about/guidelines.xhtml

8.5.6. 18 months from last radiotherapy fraction

- Flexible cystoscopy
- Chest x-ray or CT chest
- Late toxicity assessment (CTCAE v.4 and RTOG (see Appendix A2))
- Patient reported outcomes (PRO-CTCAE, ALERT-B, KHQ, sexual function and EQ5D). (Questionnaire administered to UK participants by ICR-CTSU.)

8.5.7. 24 months from last radiotherapy fraction

- Flexible cystoscopy
- CT of abdomen and pelvis
- Chest x-ray or CT chest
- Late toxicity assessment (CTCAE v.4 and RTOG (see Appendix A2))
- Patient reported outcomes (PRO-CTCAE, ALERT-B, KHQ, sexual function and EQ5D). (Questionnaire administered to UK participants by ICR-CTSU.)

8.5.8. Annually to year 5

- Flexible cystoscopy
- Chest x-ray or CT chest
- Late toxicity assessment (CTCAE v.4 and RTOG (see Appendix A2))

8.5.9. Annually thereafter

Data will be requested annually from standard follow up visits relating to:

- Assessment of disease status
- Survival

8.6. Procedure at disease progression/recurrence

Participants should be treated according to local clinical judgement at disease progression/recurrence. Patients with local or pelvic recurrence should continue to be followed up per protocol.

Following any metastatic recurrence (stage M1a/M1b), data will be requested six monthly from routine visits regarding:

- Assessment of disease status
- Survival

8.7. Withdrawal from treatment or follow-up

Participants may withdraw from trial treatment at any time at their own request, or they may be withdrawn at the discretion of the Principal Investigator. Reasons for withdrawal may include:

- Disease progression
- Unacceptable toxicity
- Co-morbidities

Participants who discontinue treatment should continue to be followed up.

If a patient withdraws from further follow-up, a trial deviation form should be submitted to ICR-CTSU stating whether the patient has withdrawn consent for further information to be sent to the ICR-CTSU or whether

For peer review only - http://bmjopen.bmj.com/site/about/guidelines.xhtml

they simply no longer wish to attend trial follow up visits. In the very rare event that a patient requests that their data is removed from the study entirely, the implications of this should be discussed with the patient first to ensure that this is their intent and, if confirmed, ICR-CTSU should be notified in writing. The patient should be made aware that any information about them that has already been published or submitted for safety monitoring purposes cannot be withdrawn.

For peer review only

For peer review only - http://bmjopen.bmj.com/site/about/guidelines.xhtml

9. SCHEDULE OF ASSESSMENTS

Visit/Assessment	Pre-neoadjuvant chemotherapy (if given)	Pre-randomisation	Pre-radiotherapy	On treatment	6 weeks after start RT¥	10 weeks after start RT	3 months after end RT	6 months after end RT	9 months after end RT	12 months after end RT	18 months after end RT	24 months after end RT	Annually to 5 years	Annually thereafter	At recurrence/ disease progression
Radiological assessment*	X	X												Disease status and survival	Treatment according to local practice. Continue folllow up per protocol after pelvic recurrence. Follow up for disease status and survival after metastatic recurrence
TURBT with completion of bladder map	X	X													
Placement of fiducial markers (optional)	X	X	X[1]												
Assessment of symptoms/toxicity			X[2]	X[2,3]	X[2]	X[2]	X[2]	X[6]	X[6]	X[6]	X[6]	X[6]	X[6]		
Full blood count, urea and electrolytes	X		X	X[4]			X	X							
PRO questionnaire (if participating)			X	X[5]			X	X[7]		X[7]	X[7]	X[7]			
Rigid cystoscopy and biopsy of tumour bed							X								
Chest x-ray							X								
Flexible cystoscopy								X	X	X	X	X	X		
CT of abdomen and pelvis								X		X		X			
Chest x-ray or CT chest								X		X	X	X	X		
Health resource utilisation				X			X	X		X	X	X			

Footnotes

* Recommended imaging: MRI pelvis, CT chest and abdomen. Minimum acceptable is chest, abdomen, pelvis CT or CT chest and CT urogram
† For patients who have not received neo-adjuvant chemotherapy
¥ For patients in the 20f cohort only
1. For patients who received neo-adjuvant chemotherapy
2. CTCAE v.4
3. Weekly on treatment
4. During weeks 1, 4 and 6 (week 6 only if receiving 32f)
5. At last fraction
6. CTCAE v.4 and RTOG
7. Questionnaires administered to UK participants by ICR-CTSU from 6 months onwards

For peer review only - http://bmjopen.bmj.com/site/about/guidelines.xhtml
For peer review only

10. TREATMENT

10.1. Pre-trial treatment

All participants should have a transurethral resection of bladder tumour (TURBT) with completion of bladder tumour map by the urologist performing the procedure. Placement of fiducial markers is recommended either during TURBT or at cystoscopy following neo-adjuvant chemotherapy (see Appendix A3).

10.2. Neo-adjuvant chemotherapy

Neo-adjuvant chemotherapy prior to randomisation according to local practice is permitted. Details will be collected on the relevant case report form.

10.3. Treatment timelines

Radiotherapy should commence within 10 weeks following randomisation or completion of neoadjuvant chemotherapy (if used), to allow sufficient time for planning. If planned radiotherapy timelines fall outside this window the ICR-CTSU should be contacted for advice prior to randomisation.

10.4. Radiotherapy fractionation schedules

Two fractionation schedules are permitted: 32 fractions or 20 fractions. Centres will specify their intended fractionation schedule prior to trial initiation and this should be used to treat all RAIDER participants throughout the trial.

10.5. Radiotherapy planning and delivery

Details of radiotherapy planning are provided in the accompanying RAIDER Radiotherapy Planning and Delivery guidelines, available for UK sites on the Radiotherapy Trials Quality Assurance (RTTQA) website (http://www.rttrialsqa.org.uk/rttqa/) and for sites in Australia and New Zealand on the TROG cancer research RAIDER page (http://trog.com.au/TROG-1402-RAIDER-trial-documents). The current version of the RAIDER radiotherapy planning and delivery guidelines must be used as the primary source for planning and delivering radiotherapy treatment within RAIDER.

10.5.1. Group 1: standard Whole Bladder RT (WBRT) (control)

Radiotherapy will be delivered on an empty bladder. One treatment plan will be generated from the planning CT scan taken immediately after voiding (CT0). 64Gy/32f or 55Gy/20f RT will be given daily for 6 ½ or 4 weeks respectively. Pre-treatment CBCT should be conducted for treatment verification.

10.5.2. Group 2: Standard dose Adaptive tumour focused RT (SART)

RT will be delivered on a partially full bladder. 2 planning CTs will be taken at 30 (CT30) and 60 (CT60) minutes after urination and drinking 350 mls water. 2 target volumes will be defined:

GTV= bladder tumour/tumour bed and extravesical spread.

CTV = GTV +whole bladder and extravesical spread

These volumes will be used to create 3 PTVs as follows:

PTVsmall or PTVmedium or PTVLarge = CTV expanded + corresponding PTV2

Where PTV2 = GTV+ 0.5cm isotropic margin for PTV2small and GTV + anisotropic margin for both PTV2medium and PTV2Large

If filling occurs between CT30 and CT60 (difference in CTV>50 mls), the PTV large will be defined from outlines derived from CT60.

PTV1 will be treated to at least 52Gy/32f or 46Gy/20f (+/-5%) and PTV2 to 64Gy/32f or 55Gy/20f. Treatment will be planned using forward planned IMRT, inversed planned IMRT, VMAT or tomotherapy. Use of alternative techniques will require specific approval from the RAIDER TMG and QA team. Centres will be

For peer review only - http://bmjopen.bmj.com/site/about/guidelines.xhtml

asked to specify their preferred method of treatment delivery and complete the appropriate Quality Assurance program.

Prior to each fraction, a CBCT will be performed and the optimal plan will be selected for that day's treatment by an accredited individual and verified by a second trained individual.

10.5.3. Group 3: Dose escalated Adaptive tumour boost RT (DART)

Plans and treatment delivery technique will be as for group 2 except an escalated dose will be given to the tumour boost volume (PTV2) of 70Gy/32f or 60Gy/20f.

If normal tissue dose constraints for escalation are not met for the medium plan, with the exception of 'other bowel' V45 and/or V50 (V37.5 and/or V41.7 for 20 fraction treatments), planning data should be provided to the RTTQA team prior to treatment to enable prospective central review by an accredited member of the Trial Management Group. If dose constraints are not met following central review, treatment at standard dose (as group 2) is recommended (following discussion with the RTTQA team).

10.6. Treatment scheduling and gaps

Treatment can start on any day of the week and should be given five days a week until completion.

Delays and treatment gaps should be avoided, however if gaps occur please refer to the RAIDER radiotherapy planning and delivery guidelines for further information. If any issues arise during RAIDER participants' treatment, ICR-CTSU and the RTTQA team should be contacted in real time for guidance.

10.7. Concomitant therapy

Participants in all groups will be permitted to receive concomitant radiosensitising therapy, the BC2001 MMC/5FU regimen or gemcitabine, carbogen or cisplatin.

Any other regimens in standard use at participating centres will require approval by the Trial Management Group. Centres should aim to use the same regimen for all patients receiving radiosensitising treatment throughout the trial. If the patient isn't fit for the centre's usual radiosensitising treatment an alternative may be substituted after discussion with the RAIDER trial manager.

10.8. Supportive care guidelines

All medication considered necessary for the patients' welfare and which is not expected to interfere with the evaluation of the treatment may be given at the discretion of the investigator.

In the event of patient catheterisation during the course of treatment it is expected that the participant will continue and complete radiotherapy in accordance with their allocated treatment group. For patients in group 1 (WBRT), as the bladder requires emptying prior to treatment delivery, the catheter must be on free flow in circumstances where there is a leg bag or voided in circumstances where there is a flip-valve. For patients in groups 2 and 3 (SART and DART), the catheter should be clamped 30 minutes before treatment (if possible).

Participants' symptoms should be managed according to local practice, although the following are suggestions for patient care:

Anaemia: Patients should be maintained by transfusion with haemoglobin above 11 grams. Iron deficiency should be treated with iron supplementation.

Dysuria/Frequency: Check for evidence of infection and treat if present with appropriate antibiotics, anticholinergics (eg oxybutynin, tolterodine), NSAIDs, analgesics.

Diarrhoea: Loperamide or opioid

Proctitis: steroid suppository +/- local anaesthetics (e.g. sheriproct, proctosedyl)

For peer review only - http://bmjopen.bmj.com/site/about/guidelines.xhtml

11. RADIOTHERAPY QUALITY ASSURANCE (QA)

A comprehensive QA programme for the RAIDER trial will be designed and implemented by the NCRI Radiotherapy Trials Quality Assurance (NCRI RTTQA) group (UK) and TROG QA group (Australia/NZ). This will include pre-trial and on-trial components. For full details of the QA programme refer to the RAIDER Radiotherapy Planning and Delivery Guidelines.

12. SAFETY REPORTING

12.1. Definitions

Adverse Event (AE)
An AE is any untoward medical occurrence in a patient administered a study treatment; the event does not necessarily have a causal relationship with the treatment.

Serious Adverse Event (SAE)
An SAE is any untoward medical occurrence that occurs after the commencement of radiotherapy and within 30 days of the last fraction of radiotherapy and:

- results in death,
- is life-threatening
- requires hospitalisation or prolongation of existing inpatients´ hospitalisation
- results in persistent or significant disability or incapacity
- is a congenital anomaly or birth defect
- is a dose limiting (grade 4) toxicity

In addition, between 6 and 18 months following completion of radiotherapy the following should be reported as an SAE:

- Radiotherapy related grade 3, 4 or 5 events

Important adverse events that are not immediately life-threatening or do not result in death or hospitalisation but may jeopardise the subject or may require intervention to prevent one of the other outcomes listed in the definition above, may also be considered serious.

Progression of the indicated disease and death due to progression of the indicated disease are not considered SAEs.

Pregnancy or aid in the conception of a child whilst participating in a trial is not itself considered an SAE but should be followed up for congenital anomalies or birth defects.

Serious Adverse Reaction (SAR)
A serious adverse reaction is an SAE that is suspected as having a causal relationship to the trial treatment, as assessed by the investigator responsible for the care of the patient. A suspected causal relationship is defined as possibly, probably or definitely related (see definitions of causality table).

For peer review only - http://bmjopen.bmj.com/site/about/guidelines.xhtml

Definitions of causality

Relationship	Description
Unrelated	There is no evidence of any causal relationship with the trial treatment
Unlikely	There is little evidence to suggest there is a causal relationship (e.g. the event did not occur within a reasonable time after administration of the trial treatment). There is another reasonable explanation for the event (e.g. the patient's clinical condition, other concomitant treatment)
Possible	There is some evidence to suggest a causal relationship (e.g. because the event occurs within a reasonable time after administration of the trial treatment). However, the influence of other factors may have contributed to the event (e.g. the patient's clinical condition, other concomitant treatments)
Probable	There is evidence to suggest a causal relationship, and the influence of other factors is unlikely
Definitely	There is clear evidence to suggest a causal relationship, and other possible contributing factors can be ruled out
Not assessable	There is insufficient or incomplete evidence to make a clinical judgement of the causal relationship.

Related Unexpected Serious Adverse Event
An adverse event that meets the definition of serious and is assessed by the CI or nominative representative as:

- "Related" – that is, it resulted from administration of any of the research procedures, and
- "Unexpected" – that is, the type of event is not listed in the protocol as an expected occurrence (see Appendix A5)

12.2. Reporting adverse events to ICR-CTSU

Any toxicity, sign or symptom that occurs after commencement of study treatment which is not unequivocally due to progression of disease, should be considered an AE.

All AEs must be reported on the relevant toxicity, sign or symptom CRF.

The severity of AEs should be graded according to CTCAE v4 criteria. For each AE, the highest grade observed since the last visit should be reported.

Whenever one or more toxicity/sign/symptom corresponds to a disease or a well-defined syndrome only the main disease/syndrome should be reported.

12.3. Reporting serious adverse events to ICR-CTSU

Any SAE (except those listed below) that occurs from the start of radiotherapy and up to 30 days following the last day of radiotherapy must be reported. In addition, any radiotherapy related grade 3, 4 or 5 events occurring between 6 and 18 months after completion of radiotherapy must be reported.

All SAEs should be reported to ICR-CTSU within 24 hours of the Principal Investigator (or designated representative) becoming aware of the event, by completing the RAIDER SAE form and faxing to:

The ICR-CTSU safety desk
Fax no: **0208 722 4368**
For the attention of the RAIDER Trial team

As much information as possible, including the Principal Investigator's assessment of causality, must be reported to ICR-CTSU in the first instance. Additional follow up information should be reported as soon as it is available.

For peer review only - http://bmjopen.bmj.com/site/about/guidelines.xhtml

All SAE forms must be completed, signed and dated by the Principal Investigator or designated representative.

The Site SAE log should be completed and the SAE form filed in the Site Investigator File.

12.4. Serious adverse events exempt from expedited reporting

The expected adverse events listed in Appendix A5 are exempt from expedited reporting if grade ≤2 but should be reported using the appropriate CRF.

12.5. Review of serious adverse events

The Chief Investigator (or designated representative) will assess all reported SAEs for causality and expectedness (NB. The Chief Investigator cannot down-grade the Principal Investigator's assessment of causality.)

SAEs assessed as having a causal relationship to study treatment and as being unexpected will undergo expedited reporting to the relevant authorities and all other interested parties by ICR-CTSU (see 12.6).

Sites should respond as soon as possible to requests from the Chief Investigator or designated representative (via ICR-CTSU) for further information that may be required for final assessment of an SAE.

12.6. Expedited reporting of related unexpected SAEs

If an SAE is identified as being related and unexpected by the Chief Investigator it will be reported by ICR-CTSU to the main REC, the Sponsor and all other interested parties within 15 days of being notified of the event.

The Principal Investigators at all actively recruiting sites will be informed of any related unexpected SAEs occurring within the trial at appropriate intervals.

The collaborative group in each participating country will report related unexpected SAEs as per their local requirements to IECs and local investigators.

12.7. Follow up of serious adverse events

SAEs should be followed up until clinical recovery is complete or until the condition has stabilised. SAE outcomes should be reported to ICR-CTSU using the relevant section of the SAE form as soon as the Principal Investigator or designee becomes aware of the outcome.

12.8. Annual safety reporting

An annual progress report will be provided to the main REC by ICR-CTSU and copied to the Sponsor and the collaborative group in each participating country at the end of the reporting year. This will include data about related unexpected SAEs and whether any safety concerns have arisen during the reporting period.

12.9. Reporting pregnancies

If any trial participant or a trial participants' partner becomes pregnant while receiving trial treatment or up to 90 days after receiving trial treatment, this should be reported to ICR-CTSU using the pregnancy reporting form. Participants who become pregnant should discontinue from trial treatment immediately. Pregnancies should be followed up until conclusion and all follow-up information should be reported to ICR-CTSU. If the outcome of the pregnancy meets the definition of serious (i.e. congenital abnormality) this should be reported to ICR-CTSU following the serious adverse event reporting procedures described above.

For peer review only - http://bmjopen.bmj.com/site/about/guidelines.xhtml

12.10. Flow diagram for SAE reporting, and action following report

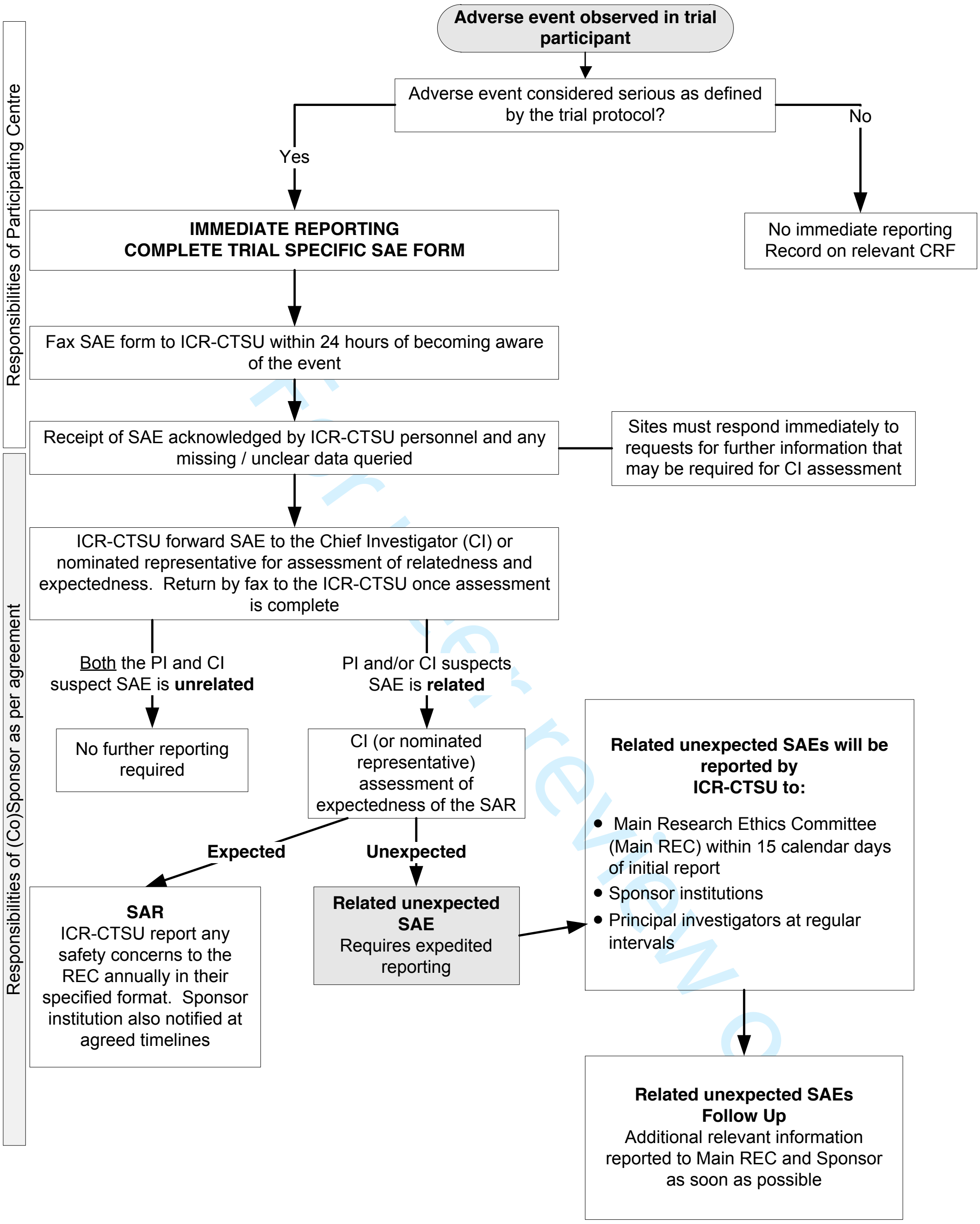

NB. All SAEs should continue to be followed up as specified above

13. STATISTICAL CONSIDERATIONS

13.1. Statistical design and sample size justification

Stage I

Stage I will assess the technical feasibility of delivering DART in a multi-centre setting. Dose constraints will be based on those in the IDEAL trial (36) and predefined by consensus of the co-investigators. Dose constraints will be detailed in the RAIDER radiotherapy planning and delivery guidelines. It is expected that 80% of patients in each DART fractionation cohort will meet dose constraints (as defined in 12.3.1). If less than 50% meet dose constraints then it will be concluded that treatment delivery is not feasible. Using an

For peer review only - http://bmjopen.bmj.com/site/about/guidelines.xhtml

A'Hern single stage design (p0=0.5, p1=0.8, 5%α, 80% power) 18 patients are required in each DART cohort. If at least 13/18 meet dose constraints it will be concluded that treatment is feasible. 36 patients in each fractionation cohort will be randomised (1:1:2 ratio) between control, SART and DART groups. Stage I will therefore require a total of 72 patients. The control and SART groups are included to enable SART to be carried forward to stage II if dose constraints cannot be met in the DART group. It also allows the assessment of equipoise and feasibility of recruitment for any subsequent phase III trial.

Assuming dose constraints are met, stage II will determine whether dose escalated RT can be delivered without detriment to long term toxicity within each fractionation cohort. At the end of stage I, the IDMC will review recruitment and toxicity data and will advise on any adaptions to trial design (e.g. unexpected toxicity in an DART fractionation cohort may lead one fractionation to be dropped; if dose constraints are consistently met for DART the SART group could be dropped for stage II; the overall sample size could be inflated to adjust for dose constraint non-compliance seen in stage I). Recruitment to stage II will continue seamlessly whilst stage I is evaluated, unless advised otherwise by the IDMC.

Stage II

Stage II has a non-comparative design aiming to rule out an upper limit of any late ≥G3 CTCAE toxicity in each DART fractionation cohort. It is expected that the proportion of patients in the control group reporting ≥G3 CTCAE toxicity between 6-18 months post-radiotherapy will be 8% (34). With 57 evaluable patients in each DART fractionation cohort, we can exclude >20% G3+ CTCAE toxicity (power 80%, 1-sided 5% α). We can also exclude >40% G2+ toxicity (with expected 20%) with >90% power, or >35% G2+ with >80% power (both 5% 1-sided α). To provide current toxicity data and allow potential transition to a phase III trial powered on oncological outcomes, stage II will be randomised with patients allocated in a 1:1:2 ratio (unless otherwise advised by the IDMC). Patients from stage I will be included in stage II.

Power calculations originally incorporated an allowance for 5% of patients non-evaluable for late toxicity by 18 months giving a target sample size of 120 patients for each fractionation cohort i.e. a total target sample size of 240 (an additional 169 patients recruited for stage II, 84 for each fractionation cohort). In September 2018 non-evaluability rates were reviewed and with the Independent Data Monitoring Committee's endorsement the target sample size (i.e. the estimate of the number of patients needed to obtain 57 evaluable DART patients) was inflated.

Using a non-evaluability rate of 22% in the 20f cohort gives a revised target sample size of 37 WBRT (control), 37 SART and 73 DART participants under the 1:1:2 allocation ratio (total of 147 patients in the 20f cohort).

Using a non-evaluability rate of 16% in the 32f cohort gives a revised target sample size of 34 WBRT (control), 34 SART and 68 DART participants under the 1:1:2 allocation ratio (total of 136 in in the 32f cohort).

The non-evaluability rate will be monitored and, with IDMC endorsement, cohort recruitment will continue until there are 57 evaluable DART patients per cohort.

Given that the primary interest is in outcomes associated with DART, the continuation of all three arms of the study will continue to be reviewed by the TMG and IDMC during stage II of the study. If it is felt that sufficient information has accrued about the feasibility of randomisation and about outcomes in the WBRT and SART arms, consideration may be given to dropping these arms if this would expedite meeting the aims of the trial or transition to subsequent phase III evaluation.

13.2. Treatment allocation

Participants will be randomised between standard radiotherapy delivery (WBRT control), SART and DART on a 1:1:2 basis separately within each fractionation cohort.

Treatment allocation is by minimisation with a random element; balancing factors will be centre, neo-adjuvant chemotherapy use and concomitant radiosensitising therapy use.

For peer review only - http://bmjopen.bmj.com/site/about/guidelines.xhtml

13.3. Endpoint definitions

13.3.1. Primary endpoints

Stage I

Proportion of randomised patients meeting radiotherapy dose constraints (in the medium plan only) to bladder, bowel and rectum in DART groups (as randomly allocated). RAIDER dose constraints will be specified in the radiotherapy delivery and planning guidelines and data collected on a plan assessment form. A patient in the 32 fraction cohort will be defined as meeting the dose constraints if all of the following are met for the medium plan: rectum 50Gy, 60Gy, 65Gy and 70Gy absolute constraints; bladder outside PTV2 60Gy and 65Gy absolute constraints and small bowel V55, V60, V65, V70 and V74 mandatory constraints. A patient in the 20 fraction cohort will be defined as meeting the dose constraints if all of the following are met for the medium plan: rectum 41.7Gy, 50Gy, 54.2Gy and 58.3Gy absolute constraints; bladder outside PTV2 50Gy and 54.2Gy absolute constraints; and small bowel V45.8, V50, V54.2, V58.3 and V61.7 mandatory constraints.

Stage II

Proportion of evaluable patients experiencing any ≥G3 Common Terminology Criteria for Adverse Events (CTCAE) v.4 late toxicity (occurring 6-18 months post radiotherapy).

13.3.2. Secondary endpoints

Stage I:

- Recruitment rate – this will be assessed overall, by country and by radiotherapy centre. Specific recruitment targets in terms of number of open centres and number of patients recruited will be defined in collaboration with the Trial Steering Committee at the beginning of the trial.
- Ability to deliver SART and DART – this will be measured by the number of patients that received their allocated treatment (technique and dose) overall, by country and by radiotherapy centre. The number of fractions using adaptive radiotherapy will be reported.

Stage II

- Clinician reported acute toxicity – this will be assessed weekly during treatment, at 6 weeks (20f cohort only) and 10 weeks from the start of radiotherapy and 3 months from the last fraction using CTCAE v.4. The worst toxicity recorded during this acute period is of primary interest.
- Patient reported outcomes (PRO) - acute and late bladder and bowel/rectal symptoms – these will be assessed using PRO-CTCAE, the King's Health Questionnaire (KHQ), ALERT-B, sexual function questions (excerpt of the EORTC QLQ-BLM30) and the EQ5D-5L. Acute is defined as 3 months from the last fraction and late is from 6 months onwards. The time point of primary interest is 18 months from the last fraction.
- Health economic related measures - time for outlining, plan generation, selection and delivery, healthcare resource usage subsequent to treatment.
- Loco-regional MIBC control – this will be defined as bladder cancer (muscle and non-muscle invasive) or cancer of the pelvic nodes. The proportion of patients free from loco-regional recurrence at 2 years will be reported.
- Progression-free survival – this will define an event as the first occurrence of local or distant disease or death and time will be measured from randomisation. Patients with no event will be censored on date of last assessment of disease.
- Overall survival – this will include deaths from any cause and time will be measured from randomisation. Patients who are alive at the time of analysis will be censored on date of last clinical assessment.

For peer review only - http://bmjopen.bmj.com/site/about/guidelines.xhtml
For peer review only

13.3.3. Exploratory endpoints

IGRT endpoints:

- Use of adaptive plans – this will be assessed by the number of small or large plans being selected rather than the medium plan for patients receiving adaptive radiotherapy.
- Target coverage – this will be assessed by retrospective outlining of selected post treatment CBCT scans and a descriptive comparison made with the plan used for treatment. A random sample of patients will be re-outlined (with the number of patients chosen based on time constraints and feasibility).
- Online/offline concordance - this will be assessed by an independent reviewer to select an appropriate plan (offline) for a random sample of patients. The concordance between the online and independent reviewer plan selection will be presented.
- Dose volume histogram analysis of adaptive and standard planning – this will be exploratory and used to inform future dose-modelling work. This will include assessment of reduction in normal tissue exposure using SART and DART and correlation of dose volume data with toxicity.

13.4. Statistical analysis plan

Primary endpoint analyses will be conducted separately for the 20f and 32f cohorts. Secondary endpoint analysis populations are defined below as appropriate. Analyses will be conducted at ICR-CTSU.

Stage I

Primary endpoint

Principal analysis will be by intention to treat for stage I. For the primary endpoint, the frequency and percentage of randomised patients able to meet the trial dose constraints in the DART group will be presented. Reasons will be presented for any patient for whom the dose constraints could not be met.

Secondary endpoints

For the secondary endpoint of recruitment, data will be presented as monthly recruitment by centre and country. Actual versus predicted recruitment will be presented graphically. Ability to deliver SART and DART will be presented as the proportion of patients who received their allocated treatment in terms of technique and dose. Data from each fractionation cohort will be presented separately and combined.

Stage II

Primary endpoints

Principal analysis of the primary endpoint will be based on the evaluable population, i.e. DART patients receiving at least one fraction of allocated treatment and having at least one toxicity assessment performed between 6 and 18 months after completion of radiotherapy. Toxicity assessments will be censored one month prior to death, bladder cancer recurrence or progression. To ensure sufficient follow-up time to observe any severe adverse reactions analyses will be conducted after all patients have been on the study for at least 18 months following the completion of radiotherapy. The proportion of patients with any G3+ CTCAE toxicity occurring within 6 to 18 months post radiotherapy will be presented for each randomised treatment group together with the 90% one-sided binomial confidence interval (the 90% two-sided confidence interval will also be presented). A sensitivity analysis will be conducted using a per protocol population. The per protocol population will include evaluable patients who received their complete fractionation schedule (either 32f or 20f) according to their randomised allocation (WBRT (control), SART or DART).

Secondary endpoints

Clinician assessed acute and late toxicity will be summarised as frequency and percentage of each grade of toxicity (CTACE and RTOG (late toxicity only)) at each time point. The distribution of acute and late toxicity will also be presented graphically as stacked barcharts. Kaplan-Meier methods may be used to use present

For peer review only - http://bmjopen.bmj.com/site/about/guidelines.xhtml

time-to-event data e.g. time to first occurrence of a grade 2 or greater event. Analyses will be conducted separately for each fraction cohort and combined.

Planned subgroup analyses will present toxicity data according to country, whether patients received neo-adjuvant and concomitant therapy. As there is limited published safety data on the use of concomitant gemcitabine with a 55Gy/20f fractionation schedule, data from this subgroup will be presented separately. To maximise the amount of data available, these exploratory subgroup analyses will be presented initially for both fractionation cohorts separately but also combining data for the fractionations.

PRO scores will be generated by combining individual items to produce subscale and total scores for each domain for each of the questionnaires using standard algorithms. Descriptive statistics will be used to present data at each time point by treatment group. Analyses to account for the longitudinal nature of the data will be explored.

Health economic related measures: time for outlining, plan generation, selection and delivery, healthcare resource usage subsequent to treatment. Data will be analysed using descriptive statistics with data presented by treatment group, both within fractionation cohorts and overall.

Loco-regional MIBC control rate at 2 years will be presented by treatment group with a 95% confidence interval. The local control rate will be presented as a proportion with patients only included in the denominator if they were able to have an assessment at 2 years. Data from each fractionation cohort will be combined.

Kaplan-Meier methods will be used to analyse progression-free and overall survival. Data will be presented by treatment group. Data from each fractionation cohort will be combined and the log-rank test (stratified by fractionation cohort) used for an exploratory comparison of the treatment groups. Pre-planned exploratory efficacy analyses will be presented according to standard dose (WBRT and SART groups) versus escalated dose (DART).

Exploratory endpoints

Data on use of the adaptive plans will be presented separately for each adaptive group with the frequency of each small, medium and large plans used, the denominator will be the total number of fractions received within the randomised group. Descriptive statistics will be used to summarise all the exploratory endpoints and data generated will be used to inform future dose modelling work.

Further details of analysis methods will be specified in a Statistical Analysis Plan in accordance with ICR-CTSU Standard Operating Procedures.

13.5. Interim analyses and stopping rules

Adherence to randomised treatment will be monitored closely during recruitment by the ICR-CTSU, particularly during stage I to determine feasibility of delivering DART. During stage I, if the medium size plan for any DART patient does not meet dose constraints, centres will be required to notify ICR-CTSU to enable central review by an accredited TMG member prior to treatment delivery. If patients are not able to receive DART (in either fractionation cohort) for any reason then a deviation form will be requested providing details of the deviation from allocated treatment. By design, the trial could be stopped at stage I, following review by the IDMC, if the reason for treatment deviation is failure to meet dose constraints in 6 or more DART patients in each fractionation cohort.

During stage II, if it is felt that sufficient information has accrued about the feasibility of randomisation and about outcomes in the WBRT and SART arms, consideration may be given to dropping these arms, following review by the IDMC, if this would expedite meeting the aims of the trial or transition to subsequent phase III evaluation.

Acute and late toxicity will be monitored at regular intervals by the IDMC. If there are more than 6 ≥G3 emergent radiotherapy related late toxicity events reported in either DART fractionation cohort the event

For peer review only - http://bmjopen.bmj.com/site/about/guidelines.xhtml

rate will exceed the threshold specified in the trial design and, on the IDMC's recommendation, the trial could be stopped or a DART fractionation cohort dropped early.

The safety of giving concomitant gemcitabine with a 55Gy/20f fractionation schedule and concomitant chemotherapy with 20f DART will be monitored by the IDMC as there are few published data for this treatment combination. Toxicity data will be presented separately for IDMC review for the 20f cohort of patients receiving concomitant therapy.

Whilst there are no formal rules for stopping the trial early due to acute toxicity, if, after 6 patients have been treated per cohort, >50% of patients experience acute grade 3 treatment related toxicity, the IDMC would be asked to advise on continuation. The frequency of subsequent acute toxicity review will be determined by the IDMC. Although the study will be underpowered to show non-inferiority of SART in terms of local control, recurrence rates will be monitored closely. A stopping rule will be formalised following discussion with the IDMC. This is likely to be based on the premise that an absolute excess of x loco-regional recurrence or more (where x will be pre-specified in collaboration with the IDMC) would be reason to consider early termination of the trial at the halfway stage of recruitment.

14. TRIAL MANAGEMENT

14.1. Trial management group (TMG)

A Trial Management Group (TMG) will be set up and will include the Chief Investigator, ICR-CTSU Scientific Lead, TROG Trial Chairperson, Co-investigators and identified collaborators, the ICR-CTSU Trial Statistician and Trial Manager. Principal Investigators and key study personnel will be invited to join the TMG as appropriate to ensure representation from a range of sites and professional groups. Membership will include a lay/consumer representative. The TMG will meet at regular intervals, and at least annually. Notwithstanding the legal obligations of the sponsor and Chief Investigator, the TMG have operational responsibility for the conduct of the trial. The Committee's terms of reference, roles and responsibilities will be defined in a charter issued by ICR-CTSU.

14.2. Trial steering committee (TSC)

The RAIDER trial will be overseen by the ICR-CTSU Urology Radiotherapy Trials Steering Committee (TSC) which includes an independent Chairman (not involved directly in the trial other than as a member of the TSC) and not less than two other independent members. The TSC will meet annually. The TSC will provide expert independent oversight of the trial on behalf of the sponsor and funder. The Committee's terms of reference, roles and responsibilities are defined in charter issued by ICR-CTSU.

14.3. Independent data monitoring committee (IDMC)

An Independent Data Monitoring Committee (IDMC) will be set up to monitor the progress of the trial and will comprise a Chairman and at least two further members with clinical or statistical expertise (at least one member must be a statistician). Membership of the IDMC will be proposed by the TMG and approved by the TSC.

The IDMC will meet in confidence at regular intervals, and at least annually. A summary of findings and any recommendations will be produced following each meeting. This summary will be submitted to the TMG and TSC, and if required, the main REC.

The IDMC will reserve the right to release any data on outcomes or side-effects through the TSC to the TMG (and if appropriate to participants) if it determines at any stage that the combined evidence from this and other studies justifies it.

The Committee's terms of reference, roles and responsibilities will be defined in a charter issued by ICR-CTSU.

For peer review only - http://bmjopen.bmj.com/site/about/guidelines.xhtml

15. RESEARCH GOVERNANCE

15.1. Sponsor responsibilities

The Sponsor of this clinical trial is the Institute of Cancer Research (ICR).

A coordinating group in each participating (non-UK) country will be delegated responsibility for trial initiation and conduct in that country on behalf of the Sponsor, as defined in an agreement between the Sponsor and the coordinating group.

15.2. Participating site responsibilities

Responsibilities of participating sites are defined in an agreement between the individual participating site and the Sponsor (UK) or the coordinating group delegated that responsibility by the Sponsor (non-UK).

16. TRIAL ADMINISTRATION AND LOGISTICS

16.1. Site activation

Before activating the trial, participating sites are required to sign an agreement accepting responsibility for all trial activity which takes place within their site.

Sites may commence recruitment once the site agreement has been signed by all required signatories, the required trial documentation is in place (as specified by ICR-CTSU) and a site initiation (visit or teleconference) has taken place. Site initiation visits will be conducted at sites where the Principal Investigator has requested one or where ICR-CTSU deems it is appropriate.

16.2. Investigator training

Each centre will complete the comprehensive pre-trial section of the quality assurance programme prior to commencing recruitment, as detailed in section 11. In addition to this, prior to trial initiation, a practical workshop will be held to educate Principal Investigators, radiographers and physicists in adaptive radiotherapy techniques. The radiotherapy quality assurance programme will continue throughout the trial, with investigator training as required.

Training materials relating to fiducial marker placement will be provided and planning CT images for the first participant at each centre with fiducial markers will be centrally reviewed to ensure consistency with placement guidelines.

16.3. Data acquisition

Electronic (e) Case Report Forms (CRF) will be used for the collection of all trial data. Data from all collaborating groups will be held centrally by the ICR-CTSU.

ICR-CTSU will provide guidance to sites to aid the completion of the eCRFs. The Trial Management Group reserves the right to amend or add to the eCRF template as appropriate. Such changes do not constitute a protocol amendment, and revised or additional forms should be used by sites in accordance with the guidelines provided by ICR-CTSU.

16.4. Central data monitoring

Once data has been entered on the eCRF by the site personnel, ICR-CTSU will review it for compliance with the protocol, and for inconsistent or missing data. Should any missing data or data anomalies be found, queries will be raised for resolution by the site.

Any systematic inconsistencies identified through central data monitoring may trigger an on-site monitoring visit.

For peer review only - http://bmjopen.bmj.com/site/about/guidelines.xhtml

16.5. On-site monitoring

If a monitoring visit is required, ICR-CTSU will contact the site to arrange the visit. Once a date has been confirmed, the site should ensure that full patient notes of participants selected for source data verification are available for monitoring.

ICR-CTSU staff conducting on-site monitoring (at UK sites) will review essential documentation and carry out source data verification to confirm compliance with the clinical trial agreement and trial protocol. If any problems are detected during the course of the monitoring visit, ICR-CTSU will work with the Principal Investigator or delegated individual to resolve issues and determine appropriate action.

16.6. Completion of the study and definition of study end date

The study end date is deemed to be the date of last data capture.

16.7. Archiving

Essential trial documents should be retained according to local policy and for a sufficient period for possible inspection by the regulatory authorities (at least 5 years after the date of last data capture). Documents should be securely stored and access restricted to authorised personnel.

17. PATIENT PROTECTION AND ETHICAL CONSIDERATIONS

17.1. Trial approvals

This trial has been formally assessed for risk by ICR-CTSU.

In the UK, ICR-CTSU, on behalf of the Sponsor, will ensure that the trial has received ethics approval from a research ethics committee for multi-centre trials and global R&D approval via the NIHR Coordinated System for gaining NHS Permission. Before entering patients, the Principal Investigator at each site is responsible for submitting Site Specific Information and gaining local Research and Development approval of this protocol.

The coordinating group in each country, on behalf of the Sponsor, will ensure that the trial has received all relevant ethical, regulatory and institutional approval prior to the recruitment of any patients. Further details are provided in Appendix A6.

17.2. Trial conduct

This trial will be conducted according to the approved protocol and its amendments, supplementary guidance and manuals supplied by the Sponsor and in accordance with relevant national guidelines.

17.3. Informed consent

Patients should be asked to sign the current ethics approved main RAIDER consent form at trial entry after receiving both verbal and written information about the trial, having been given sufficient time to consider this information. All consent forms must be countersigned by the Principal Investigator or a designated individual. A signature log of delegated responsibilities, listing the designated individuals and the circumstances under which they may countersign consent forms, must be maintained at the participating site. This log, together with original copies of all signed patient consent forms, should be retained in the Site Investigator File and must be available for inspection. The current ethics approved RAIDER patient information sheets should be provided in addition to any standard patient information sheets that are provided by the site and used in routine practice.

17.4. Patient confidentiality

Patients will be asked to consent to their full name being collected at registration in addition to their date of birth, hospital number, postcode and NHS number or equivalent to allow linkage with routinely collected healthcare data.

For peer review only - http://bmjopen.bmj.com/site/about/guidelines.xhtml

Each investigator should keep a separate log of all participants' Trial IDs, names, addresses and hospital numbers. The investigator must retain trial documents (e.g. participants' written consent forms) in strict confidence. The investigator must ensure the participants' confidentiality is maintained at all times.

Representatives of ICR-CTSU and the regulatory authorities will require access to participants' hospital notes for quality assurance purposes. ICR-CTSU will maintain the confidentiality of participants at all times and will not reproduce or disclose any information by which participants could be identified.

17.5. Data protection act (DPA)

ICR-CTSU will comply with all applicable data protection laws.

17.6. Liability

The coordinating group in each country will ensure that appropriate indemnity arrangements are place to meet the potential legal liabilities of investigators conducting the trial .

18. FINANCIAL MATTERS

This trial is investigator designed and led and has been approved by the Clinical Trials Advisory and Awards Committee (CTAAC) of Cancer Research UK and Cancer Australia.

ICR has received funding from Cancer Research UK for the central coordination of the trial. In the UK, the trial meets the criteria for R&D support as outlined in the Statement of Partnership on Non-Commercial R&D in the NHS in England. The trial is part of the National Institute for Health Research Clinical Research Network (NCRN) portfolio. NCRN resources should therefore be made available for the trial to cover UK specific research costs.

The coordinating group in other countries will ensure that sufficient funding is available for the coordination and conduct of the trial.

19. PUBLICATION POLICY

The main trial results will be based on data from all collaborative groups and will be published in a peer-reviewed journal, on behalf of all collaborators. The manuscript will be prepared by a writing group, consisting of members of the TMG and selected participating clinicians. All participating clinicians will be acknowledged in the publication.

Any presentations and publications relating to the trial must be authorised by the TMG. Authorship of any secondary publications e.g. those relating to sub-studies, will reflect the intellectual and time input into these studies.

No investigator may present or attempt to publish data relating to the RAIDER trial without prior permission from the TMG.

20. ASSOCIATED STUDIES

20.1. Patient reported outcome measures study

Patient reported outcomes will be a secondary endpoint in the main trial and will be analysed as described in the statistical analysis plan.

Further details are provided in Appendix A4.

20.2. RAIDER translational sample collection

Prospective consent will be sought for access to formalin fixed paraffin embedded (FFPE) tissue blocks routinely obtained at first diagnosis and those from any subsequent first recurrence. FFPE blocks will be requested retrospectively from sites and will be sent to the University of Manchester for storage. Samples

For peer review only - http://bmjopen.bmj.com/site/about/guidelines.xhtml
For peer review only

will be held under the custodianship of the Trial Management Group on behalf of the sponsor. Translational analyses will be conducted at a later date once appropriate funding has been secured.

For peer review only

For peer review only - http://bmjopen.bmj.com/site/about/guidelines.xhtml

A1. WHO PERFORMANCE STATUS

Grade	Performance Status
0	Able to carry out all normal activity without restriction.
1	Restricted in physically strenuous activity but ambulatory and able to carry out light work.
2	Ambulatory and capable of all self-care but unable to carry out any work; up and about more than 50% of waking hours.
3	Capable of only limited self-care; confined to bed or chair more than 50% of waking hours.
4	Completely disabled; cannot carry on any self-care; totally confined to bed or chair.

A2. RTOG/EORTC LATE RADIATION MORBIDITY SCORING SCHEMA

0	1	2	3	4	5
BLADDER					
None	Slight epithelial atrophy<br>Minor telangiectasia (microscopic haematuria)	Moderate frequency<br>Generalized telangiectasia<br>Intermittent macroscopic haematuria	Severe frequency and dysuria<br>Severe generalized telangiectasia (often with petechiae)<br>Frequent haematuria<br>Reduction in bladder capacity (<150 cc)	Necrosis/ Contracted bladder (capacity <100 cc)<br>Severe haemorrhagic cystitis	Death due to toxicity
SMALL/LARGE INTESTINE					
None	Mild diarrhoea<br>Mild cramping<br>Bowel movement 5 times daily<br>Slight rectal discharge or bleeding	Moderate diarrhoea and colic<br>Bowel movement >5 times daily<br>Excessive rectal mucus or intermittent bleeding	Obstruction or bleeding requiring surgery	Necrosis/ Perforation Fistula	Death due to toxicity

For peer review only - http://bmjopen.bmj.com/site/about/guidelines.xhtml

A3. TUMOUR LOCALISATION GUIDELINES

For radiotherapy planning the delineated bladder tumour will be defined using all pre-treatment diagnostic imaging, surgical bladder map and the placement of fiducial markers if possible (see Radiotherapy Planning and Delivery Guidelines).

A3.1 Fiducial marker placement

Prior to radiotherapy, where possible fiducial marker insertion (gold seed or Lipiodol) into the bladder wall surrounding the tumour should be considered. Only patients medically fit to undergo a general anaesthetic should be considered for gold seed insertion. Only patients fit for general anaesthetic and without a history of contrast medium sensitivity or active thyroid disease should be considered for Lipiodol insertion.
The fiducial markers are inserted into the bladder wall to demarcate the maximum extent of visible tumour or tumour bed. Gold seeds need to be inserted via a customised introducer.
The recommended procedure for Lipiodol injection is

1. Undertake cystoscopy under general anaesthetic performing a cystourethroscopy with visual mapping of scars and lesions. Record details on trial proforma. Measure bladder volume. If required biopsy scar plus/minus random biopsy.
2. Lipiodol is inserted using a 5 French 'Botox' needle.
3. Draw up 5 mls of Lipiodol
4. With the bladder full, inject 0.5 mls subepithelially 2cms away from scar or residual tumour. Use 4-6 injections circumferentially around scar. Do not exceed injection volume as this can lead to pelvic leakage.
5. Diathermy injection sites to prevent Lipiodol leaking back out
6. Record details of procedures on bladder map, make note of number of injections, position and distance from scar.

Fiducial marker placement is unlikely to result in side effects over and above the toxicities associated with cystoscopy +/- general anaesthetic.

A3.2 Surgical bladder map

At the time of cystoscopy the urologist will be ask to localise the tumour (size and position) on a surgical bladder map to aid tumour localisation for radiotherapy planning.

A3.3 Training and quality assurance

A video demonstrating the fiducial marker placement technique will be available on the ICR-CTSU website. Each centre will be requested to nominate a lead surgeon providing oversight of fiducial marker placement for RAIDER trial participants (if using). Lead surgeons will be asked to provide details of their centres' fiducial marker placement experience and to provide assurance that those placing fiducial markers have completed the required training.

Planning CT images for the first participant with fiducial marker placement at each centre will be centrally reviewed.

For peer review only - http://bmjopen.bmj.com/site/about/guidelines.xhtml

A4. PATIENT REPORTED OUTCOMES STUDY

A4.1 Background

Patient reported outcomes (PRO) are a key secondary endpoint within RAIDER. PRO within RAIDER will focus on the impact of bladder radiotherapy on symptoms experienced by patients. The aim will be to collect detailed information about the impact of bladder radiotherapy on participants' daily lives, with a focus on side effects being experienced but also including a measure of general wellbeing.

The objective of the PRO sub-study within RAIDER is to compare the impact of adaptive planned radiotherapy on side effects as reported by the participants. This will help to support any differences in toxicity established within the primary endpoint of clinician reported toxicity. In addition, PRO data will be compared with clinician reported toxicity to give an indication of the concordance of the two measures.

A4.2 Hypotheses

1. SART minimises treatment toxicity and improves patient reported symptoms /quality of life
2. DART is tolerated well and has no or minimal impact on patients' reported experiences

A4.3 Quality of life measures

Patient reported outcomes will be measured using the PRO-CTCAE™ questionnaire, King's Health Questionnaire (KHQ), sexual function questions, ALERT-B and the EQ-5D.

PRO-CTCAE is a patient-reported outcome measure developed to evaluate the frequency, severity and interference of symptomatic toxicity in patients on cancer clinical trials. It was designed to be used as a companion to the Common Terminology Criteria for Adverse Events (CTCAE). PRO-CTCAE includes an item library representing symptomatic toxicities drawn from the CTCAE. Items selected for inclusion relate to gastrointestinal symptoms (41).

Urinary side-effects experienced by participants will be captured using the KHQ, which has been validated for use in patients with overactive bladder(42) and captures details of the severity of symptoms and the impact of urinary incontinence on day to day living. Impact on sexual function will be assessed using an excerpt of the EORTC QLQ-BLM30, a muscle invasive bladder cancer specific questionnaire (43).

Participants will also be asked to complete the EQ5D questionnaire, a brief standardised instrument which provides a simple descriptive profile of health status (44) and the three-item ALERT-B Questionnaire which provides a validated screening tool to detect chronic gastrointestinal symptoms after pelvic radiotherapy in cancer survivors (45).

A4.4 Study design

Patients are eligible for the PRO study if they fulfil the RAIDER eligibility criteria. Participants will be asked in the patient information sheet to consent to regular completion of PRO questionnaires. Patients who decline to take part in the RAIDER PRO study will remain eligible for the main trial. PRO is a secondary endpoint in the main trial and the primary timepoint of interest is 18 months after completion of radiotherapy.

A4.5 Timing of data collection

Participants will be asked to complete a questionnaire in clinic within 2 weeks prior to the start of radiotherapy. Further questionnaires will be completed in clinic at the end of treatment delivery and 3 months from the end of treatment. Four further booklets will be sent to participants' homes by ICR-CTSU at 6, 12, 18 and 24 months from the end of treatment.

A4.6 Compliance

Missing data may hamper interpretation of PRO. Missing data may arise because participants do not complete the questionnaires at the appropriate time (unit non-response), or because patients may miss questions within the questionnaires (item non-response). In a population of patients with low performance status such as those included in RAIDER, there is potential for non-response and informative censoring (with

For peer review only - http://bmjopen.bmj.com/site/about/guidelines.xhtml

data not missing at random). During the study, compliance with PRO questionnaire completion will be monitored by the trial oversight committees.

A4.7 Statistical considerations

Patient reported outcome analyses will be used to supplement results of clinician assessed treatment toxicity, therefore a formal sample size calculation has not been performed. An analysis plan will be developed in consultation with the TMG with key endpoints identified from each questionnaire. Standard algorithms will be used to derive scores and handle missing data in quality of life questionnaires. Quality of life data will be presented at individual time-points and analyses to account for the longitudinal nature of the data may be used.

For peer review only

For peer review only - http://bmjopen.bmj.com/site/about/guidelines.xhtml

A5. EXPECTED SERIOUS ADVERSE EVENTS

Hospitalisation for any of the following adverse events is exempt from expedited reporting if the event is grade 2 or less:

- Transfusion secondary to bleeding from bladder tumour or anaemia
- Haematuria
- Dysuria/frequency
- Nausea/vomiting
- Bladder spasms or pain
- Diarrhoea
- Constipation
- Abdominal pain
- Urinary tract infection
- Urinary/clot retention
- Fatigue
- Neutropaenia (related to concomitant chemotherapy)
- Thrombocytopaenia (related to concomitant chemotherapy)
- Neutropaenic sepsis (related to concomitant chemotherapy)

For peer review only

For peer review only - http://bmjopen.bmj.com/site/about/guidelines.xhtml

A6. TRANS TASMAN RADIATION ONCOLOGY GROUP SPECIFIC ADDENDUM

TROG CANCER RESEARCH

TROG 14.02

**Final GSA Version 3 Date:**	23/01/2019

**Collaborating Group:**	Trans Tasman Radiation Oncology Group (TROG)
**ABN:**	45 132 672 292
**Address:**	PO Box 88, Waratah, NSW, Australia, 2298
**Phone:**	+61 2 401 43911
**Email:**	trog@trog.com.au
**Website:**	www.trog.com.au
**TROG Representative:**	Susan Goode

GSA Authorisation:

_______________ *sign* _______________ *date*

**TROG Trial Chair:**	Associate Professor Farshad Foroudi
**Address:**	Olivia Newton-John Cancer & Wellness Centre, Austin Health<br>145 Studley Road<br>PO Box 5555 Heidelberg, VIC, Australia, 3084
**Email:**	farshad.foroudi@austin.org.au
**Phone:**	+61 3 9496 9797

GSA Authorisation:

_______________ *sign* _______________ *date*

FORWARD

The Trans Tasman Radiation Oncology Group (TROG) has been authorised by the Institute of Cancer Research (ICR) to undertake a coordinating role for participants enrolled in Australia and New Zealand on this trial.

The involvement of TROG necessitates a number of changes to the procedures documented in the main body of the RAIDER protocol. The following sections have been adjusted for TROG trial sites and participants and replace, or add to, the above RAIDER protocol sections where relevant.

For peer review only
For peer review only - http://bmjopen.bmj.com/site/about/guidelines.xhtml

A6.1 Group specific committees and contacts

A6.1.1. TROG Trial Coordinating Centre

Additional to protocol page 1

The TROG Trial Coordinating Centre (TCC) will be the liaison between the ICR and the ANZ trial sites.

**TROG Trial Coordinator**	Patrick Wheeler
**Address**	TROG Trial Coordinating Centre<br>PO Box 88, Waratah, NSW<br>2298, Australia
**Email**	RAIDER@trog.com.au
**Phone**	+61 2 4014 3903

A6.2 Randomisation

Replaces section 7 of the protocol

Patients will be randomised by the ICR Clinical Trials and Statistics Unit (ICR-CTSU) via the TROG Central Operations Office (TCOO).

Randomisation Case Report Forms (paper forms) confirming that the patient is eligible and has provided written consent for the trial must be forwarded to the TCOO on:

0061 2 4014 3902 or RAIDER@trog.com.au

Randomisation requests will be processed through the ICR-CTSU system during the next business day after receipt.

Randomisation should take place within 10 weeks prior to the planned start date of radiotherapy. Treatment allocation will be by minimisation (with a random component). An eligibility and randomisation checklist must be completed prior to randomisation.

The following information will be required at randomisation:

- Name of treating and recruiting hospital, consultant and person randomising patient
- Confirmation that patient has given written informed consent for trial and for any sub-studies;
- Confirmation that patient is eligible for the trial by completion of the eligibility checklist

ICR-CTSU will send written confirmation of trial entry, confirming the patient's unique randomisation number (Trial ID) and treatment allocation to the TCOO who shall notify the data management contact at the recruiting centre.

A6.3 Safety reporting

A6.3.1. Reporting serious adverse events to TROG

Replaces section 12.3 of the protocol

Any Serious Adverse Event (SAE) (except those listed in Appendix A5 of the ICR protocol) that occurs from the start of radiotherapy and up to 30 days following the last day of radiotherapy must be reported.

For peer review only - http://bmjopen.bmj.com/site/about/guidelines.xhtml

TROG trial sites in Australia and New Zealand shall report SAEs within 24 hours of the Principal Investigator (or designated representative) becoming aware of the event, by completing the RAIDER SAE form and faxing to:

The TROG Central Operations Office

Fax no: 0061 2 4014 3902

As much information as possible, including the Principal Investigator's assessment of causality, must be reported to the TROG TCC in the first instance. Additional follow up information should be reported as soon as it is available.

All SAE forms must be completed, signed and dated by the Principal Investigator or designated representative.

The Site SAE log should be completed and the SAE form filed in the Site Investigator File.

A6.4 Review of serious adverse events

Replaces section 12.5 of the protocol

The TROG Trial Chairperson (or designated representative) will assess all reported SAEs for Australian and New Zealand (ANZ) sites for causality and expectedness (NB. The TROG Trial Chairperson cannot down-grade the Principal Investigator's assessment of causality.)

SAEs assessed as having a causal relationship to study treatment and as being unexpected will undergo expedited reporting to the relevant regulatory authorities and all other interested parties by the TROG TCC (see 3.3).

Sites should respond as soon as possible to requests from the TROG Trial Chairperson or designated representative (via TROG) for further information that may be required for final assessment of an SAE. ICR-CTSU will be provided with details of every reported SAE once final assessment is completed.

A6.5 Expedited reporting of related unexpected SAEs

Replaces section 12.6 of the protocol

If an SAE is identified as being related and unexpected by the TROG Trial Chairperson it will be reported by the TROG TCC to the lead Human Research Ethics Committee (HREC), the Sponsor (via ICR-CTSU) and all other interested parties within each parties' reporting timelines.

The Principal Investigators at all actively recruiting sites will be informed of any related unexpected SAEs occurring within the trial at appropriate intervals.

A6.6 Follow up of serious adverse events

Replaces section 12.7 of the protocol

SAEs should be followed up until clinical recovery is complete or until the condition has stabilised. SAE outcomes should be reported to the TROG TCC using the relevant section of the SAE form as soon as the Principal Investigator or designee becomes aware of the outcome.

For peer review only

For peer review only - http://bmjopen.bmj.com/site/about/guidelines.xhtml

A6.7 Flow diagram for SAE reporting, and action following report

Replaces section 12.10 of the protocol

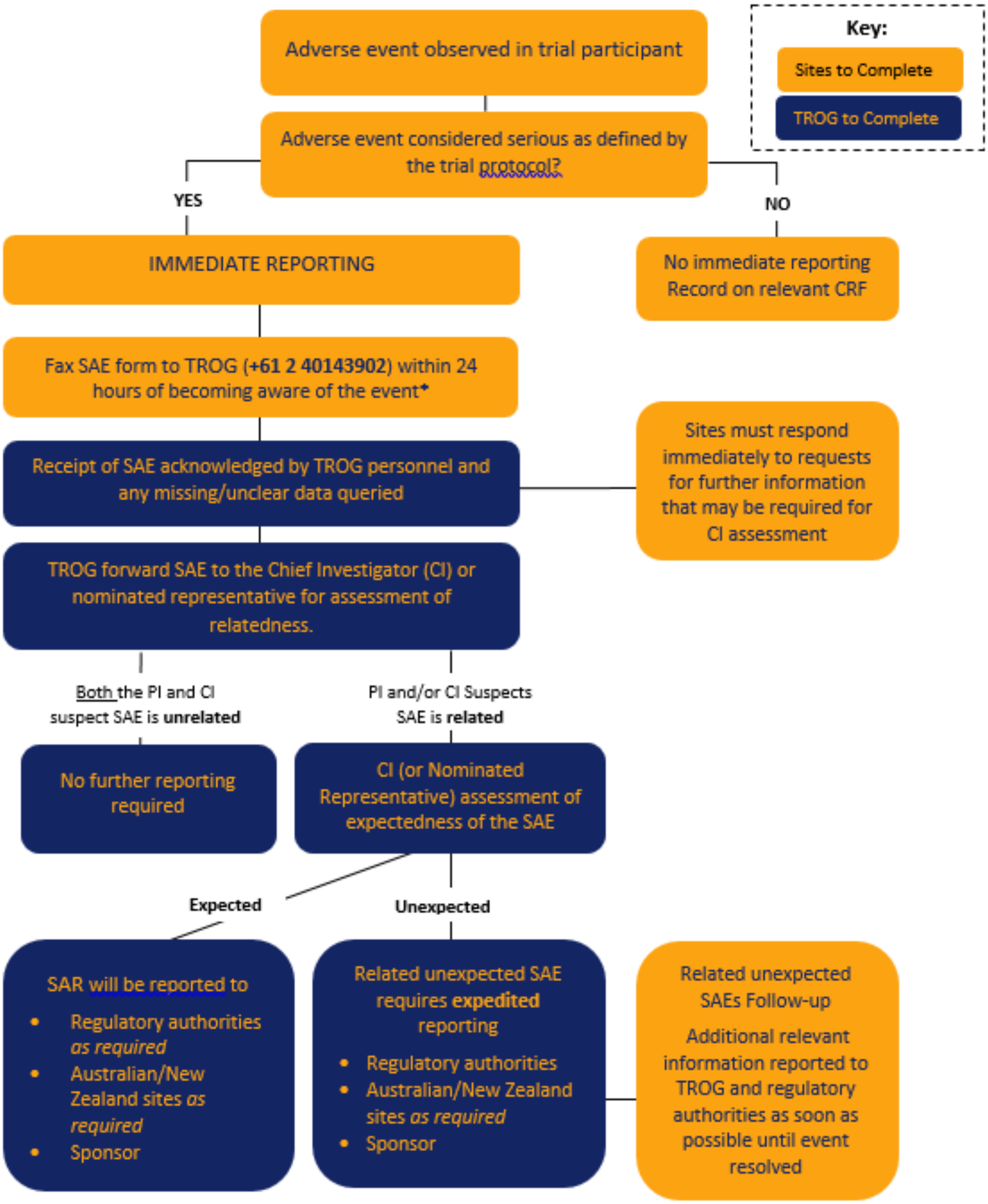

Site investigator to also report SAE to approving HREC and/or RGO as required

A6.8 Trial administration and logistics

Replaces section 16 of the protocol

A6.8.1. Site activation

Before activating the trial, participating sites are required to sign an agreement, issued by TROG, accepting responsibility for all trial activity which takes place within their site.

Sites may commence recruitment once the site agreement has been signed by all required signatories, the required trial documentation is in place (as specified by TROG) and a site initiation (visit or teleconference) has taken place.

A6.8.2. Investigator training

Each centre will complete radiotherapy quality assurance procedures, as described in the TROG 14.02 Radiotherapy Planning and Delivery Guidelines, available on request from the TROG trial coordinator, prior to commencing recruitment. The quality assurance programme will continue throughout the trial, with investigator training as required.

Training materials relating to fiducial marker placement will be provided and planning CT images for the first participant at each centre with fiducial markers will be centrally reviewed to ensure consistency with placement guidelines.

A6.8.3. Data acquisition

Electronic (e) Case Report Forms (CRF) will be used for the collection of all trial data. Data from all collaborating groups will be held centrally by the ICR-CTSU.

The TROG Central Operations Office will provide guidance to ANZ sites to aid the completion of the eCRFs. The Trial Management Group reserves the right to amend or add to the eCRF template as appropriate. Such changes do not constitute a protocol amendment.

A6.8.4. Central data monitoring

Once data has been entered on the eCRF by the site personnel, ICR-CTSU will review it for compliance with the protocol, and for inconsistent or missing data. Should any missing data or data anomalies be found, queries will be raised for resolution by the site.

Any systematic inconsistencies identified through central data monitoring may trigger an on-site monitoring visit.

A6.8.5. On-site monitoring

If a monitoring visit is required, TROG will contact the site to arrange the visit. Once a date has been confirmed, the site should ensure that full patient notes of participants selected for source data verification are available for monitoring.

TROG staff conducting on-site monitoring will review essential documentation and carry out source data verification to confirm compliance with the clinical trial agreement and trial protocol. If any problems are detected during the course of the monitoring visit, TROG will work with the Principal Investigator or delegated individual to resolve issues and determine appropriate action.

A6.8.6. Completion of the study and definition of study end date

The study end date is deemed to be the date of last data capture.

A6.8.7. Archiving

Essential trial documents and source documentation (including medical histories, radiological imaging, laboratory tests, chemotherapy and radiotherapy treatment records, verification films and portal images) , must be retained for 15 years after completion of the trial in accordance with ICH GCP Guidelines. Documents should be securely stored and access restricted to authorised personnel.

A6.9 Patient reported outcomes study

Replaces section A4.5 of the protocol

Participants will be asked to complete a questionnaire in clinic within 2 weeks prior to randomisation. Further questionnaires will be completed in clinic at the end of treatment delivery and at 3, 6, 12, 18 and 24 months from the end of treatment.

For peer review only - http://bmjopen.bmj.com/site/about/guidelines.xhtml

Following progression patients should continue to be asked to complete booklets in accordance with the follow up schedule if they are willing to do so.

A6.10 Financial matters

Replaces section 18 of the protocol

Funding is being sought from competitive grants in Australia and New Zealand. A Cancer Australia grant has been awarded for Australian Sites. A Cancer Society of New Zealand grant has been awarded for New Zealand sites.

A6.11 Research governance

Additional to section 15 of the protocol

A6.11.1. Trial chairperson(s)

TROG is the sponsor's legal representative for this trial in Australia and New Zealand (ANZ). The TROG Trial Chairperson(s) shall be responsible for the conduct of the trial in Australia and New Zealand as set out in the Agreement between TROG and the ICR.

A6.11.2. Trial management committee

The ANZ Trial Management Committee (TMC) will be responsible for monitoring of the progress of the trial in TROG trial sites, decision making, education and information services and reporting as described in TROG Policy Statement TPS E8 Trial Management Committee Responsibilities. The TMC will feedback to the RAIDER Trial Management Group via the TROG Trial Chairperson (who will be a member of the RAIDER Trial Management Group) and other TROG representatives as appropriate.

A6.11.3. Principal Investigator

In each participating centre a Principal Investigator (Radiation Oncologist) will be identified, and will be responsible for identification, recruitment, data collection and completion of CRFs along with follow up of study patients and adherence to the study protocol. Each Principal Investigator will be a member of TROG and adhere to TROG Policy Statements. One investigator per country will be nominated as national coordinator and one investigator per ethics jurisdiction within Australia and within New Zealand will be nominated as Lead Ethics Coordinator. Further details regarding the responsibilities and delegations are set out in the Clinical Trial Agreement between TROG and the participating centre.

A6.12 Patient protection and ethical considerations

Additional to section 17 of the protocol

A6.12.1. Aboriginal and Torres Strait Islander values and principles

TROG recognises and commits to the respect of Aboriginal and Torres Strait Islander cultural values and principles.

Although this trial is not targeted specifically to Aboriginal and Torres Strait Islander peoples, a person from one of these communities may be invited to participate if they meet the eligibility criteria of this trial. This decision will be at the discretion of the Principal Investigator at the Trial Site who shall consent and treat the patient according to the principles set forth in the Guidelines for Ethical Conduct in Aboriginal and Torres Strait Islander Health Research and any specific requirements of the approving Human Research Ethics Committee.

A6.12.2. Insurance and compensation

TROG endorses the principles of the Medicines Australia Guidelines for Compensation for Injury Resulting from Participation in a Company Sponsored Trial and the Research Medicines Industry equivalent in New Zealand.

For peer review only - http://bmjopen.bmj.com/site/about/guidelines.xhtml

RAIDER Protocol
ICR-CTSU

To provide protection for trial participants involved in TROG Clinical Trials, TROG maintains a clinical trials insurance policy.

For peer review only

For peer review only - http://bmjopen.bmj.com/site/about/guidelines.xhtml

A7. GLOSSARY

AE	Adverse Event
APPLY	Adaptive predictive planning for hypofractionated bladder radiotherapy
CBCT	Cone Beam CT
CI	Chief Investigator
CI	Confidence Interval
CIS	Carcinoma In Situ
CRF	Case Report Form
CT	Computed Tomography
CTCAE	Common Terminology Criteria for Adverse Events
CTV	Clinical Target Volume
CXR	Chest X-Ray
DART	Dose escalated Adaptive tumour focused Radiotherapy
DCF	Data Capture Form
DVH	Dose Volume Histogram
dwMRI	Diffusion weighted Magnetic resonance Imaging
EORTC	European Organisation for Research and Treatment of Cancer
f	Fraction
FBC	Full Blood Count
GI	Gastrointestinal
GSA	Group Specific Addendum
GTV	Gross Tumour Volume
GU	Genitourinary
Gy	Gray
HR	Hazard Ratio
ICR	The Institute of Cancer Research
ICR-CTSU	The Institute of Cancer Research Clinical Trials and Statistics Unit
IDMC	Independent Data Monitoring Committee
IGRT	Image Guided Radiotherapy
IMRT	Intensity Modulated Radiotherapy
KHQ	King's Health Questionnaire
MDT	Multi-Disciplinary Team
MIBC	Muscle Invasive Bladder Cancer
MRI	Magnetic Resonance Imaging
NCRI	National Cancer Research Institute
NCRI RTTQA	NCRI Radiotherapy Clinical Trials Quality Assurance group
NICE	National Institute for Health and Clinical Excellence
NSAID	Non-Steroidal Anti-Inflammatory Drug
PI	Principal Investigator
PIS	Patient Information Sheet
PRO	Patient Reported Outcomes
PTV	Planning Target Volume
QA	Quality Assurance
R&D	Research and Development
REC	Research Ethics Committee
RMH	Royal Marsden Hospital
RT	Radiotherapy
RTOG	Radiation Therapy Oncology Group
RTTQA	Radiotherapy Trials Quality Assurance
SAE	Serious Adverse Event
SAR	Serious Adverse Reaction
SART	Standard dose Adaptive tumour focused Radiotherapy
TCC	Transitional Cell Carcinoma
TMG	Trial Management Group
TSC	Trial Steering Committee
TURBT	Transurethral resection of Bladder Tumour
U & Es	Urea and Electrolytes
WHO	World Health Organisation

For peer review only - http://bmjopen.bmj.com/site/about/guidelines.xhtml

A8. REFERENCES

1 Bladder cancer Key Facts. http://publications.cancerresearchuk.org/publicationformat/formatfactsheet/bladderkeyfacts.html: Cancer Research UK; 2014.
2 Cancer in Australia: an overview, 2012. 74 ed. http://www.aihw.gov.au/publication-detail/?id=60129542359: Australian Institute of Health and Welfare & Australasian Association of Cancer Registries 2012.; 2014.
3 Rodel C. Combined-Modality Treatment and Selective Organ Preservation in Invasive Bladder Cancer: Long-Term Results. Journal of Clinical Oncology. 2002;**20**(14):3061-71.
4 NICE. Guidance on Cancer Services Improving Outcomes in Urological Cancers The Manual. online: NICE; 2002.
5 James N, Hall E, Jenkins P, et al. Radiotherapy with or without chemotherapy in muscle invasive bladder cancer. New England Journal of Medicine. 2012;**366**:1477-88.
6 Fokdal L, Honore H, Hoyer M, Meldgaard P, Fode K, von der Maase H. Impact of changes in bladder and rectal filling volume on organ motion and dose distribution of the bladder in radiotherapy for urinary bladder cancer. Int J Radiat Oncol Biol Phys. 2004 Jun 1;**59**(2):436-44.
7 Lotz HT, Pos FJ, Hulshof MC, et al. Tumor motion and deformation during external radiotherapy of bladder cancer. Int J Radiat Oncol Biol Phys. 2006 Apr 1;**64**(5):1551-8.
8 Meijer GJ, Rasch C, Remeijer P, Lebesque JV. Three-dimensional analysis of delineation errors, setup errors, and organ motion during radiotherapy of bladder cancer. Int J Radiat Oncol Biol Phys. 2003 Apr 1;**55**(5):1277-87.
9 Muren LP, Smaaland R, Dahl O. Organ motion, set-up variation and treatment margins in radical radiotherapy of urinary bladder cancer. Radiother Oncol. 2003 Dec;**69**(3):291-304.
10 Sur RK, Clinkard J, Jones WG, et al. Changes in target volume during radiotherapy treatment of invasive bladder carcinoma. Clin Oncol (R Coll Radiol). 1993;**5**(1):30-3.
11 Turner SL, Swindell R, Bowl N, et al. Bladder movement during radiation therapy for bladder cancer: implications for treatment planning. Int J Radiat Oncol Biol Phys. 1997 Sep 1;**39**(2):355-60.
12 Pos F, Remeijer P. Adaptive management of bladder cancer radiotherapy. Semin Radiat Oncol. 2010 Apr;**20**(2):116-20.
13 Lalondrelle S, Huddart R. Improving radiotherapy for bladder cancer: an opportunity to integrate new technologies. Clin Oncol (R Coll Radiol). 2009 Jun;**21**(5):380-4.
14 Lalondrelle S, Huddart R, Warren-Oseni K, et al. Adaptive-predictive organ localization using cone-beam computed tomography for improved accuracy in external beam radiotherapy for bladder cancer. Int J Radiat Oncol Biol Phys. 2011 Mar 1;**79**(3):705-12.
15 Pos FJ, Hulshof M, Lebesque J, et al. Adaptive radiotherapy for invasive bladder cancer: a feasibility study. Int J Radiat Oncol Biol Phys. 2006 Mar 1;**64**(3):862-8.
16 Foroudi F, Wong J, Kron T, et al. Online adaptive radiotherapy for muscle-invasive bladder cancer: results of a pilot study. Int J Radiat Oncol Biol Phys. 2011 Nov 1;**81**(3):765-71.
17 Huddart RA. SJ. What is the most effective way of reducing normal tissue toxicity in radical radiotherapy treatment of muscle invasive bladder cancer? Radiother Oncol 2002;**64(Suppl 1):S305 (1043a)**.
18 Emami B, Lyman J, Brown A, et al. Tolerance of normal tissue to therapeutic irradiation. Int J Radiat Oncol Biol Phys. 1991 May 15;**21**(1):109-22.
19 Marks LB, Carroll PR, Dugan TC, Anscher MS. The response of the urinary bladder, urethra, and ureter to radiation and chemotherapy. Int J Radiat Oncol Biol Phys. 1995 Mar 30;**31**(5):1257-80.
20 Mangar SA, Foo K, Norman A, et al. Evaluating the effect of reducing the high-dose volume on the toxicity of radiotherapy in the treatment of bladder cancer. Clin Oncol (R Coll Radiol). 2006 Aug;**18**(6):466-73.
21 The Radiotherapy Innovation Fund An Evaluation of the Prime Minister's £23 million Fund. http://www.cancerresearchuk.org/prod_consump/groups/cr_common/@nre/@pol/documents/generalcontent/rifevalreport.pdf: Cancer Research UK; 2013 July 2013.
22 National Radiotherapy Implementation Group Report Image Guided Radiotherapy (IGRT) Guidance for implementation and use. http://ncat.nhs.uk/sites/default/files/uploaded/National%20Radiotherapy%20Implementation%20Group%20Report%20IGRTAugust%202012l.pdf: National Cancer Action Team; 2012.
23 Foroudi F, Pham D, Rolfo A, et al. The outcome of a multi-centre feasibility study of online adaptive radiotherapy for muscle-invasive bladder cancer TROG 10.01 BOLART. Radiother Oncol. 2014 May;**111**(2):316-20.
24 Hoskin PJ, Rojas AM, Bentzen SM, Saunders MI. Radiotherapy with concurrent carbogen and nicotinamide in bladder carcinoma. J Clin Oncol. 2010 Nov 20;**28**(33):4912-8.
25 Choudhury A, Swindell R, Logue JP, et al. Phase II study of conformal hypofractionated radiotherapy with concurrent gemcitabine in muscle-invasive bladder cancer. J Clin Oncol. 2011 Feb 20;**29**(6):733-8.

For peer review only - http://bmjopen.bmj.com/site/about/guidelines.xhtml

26 Coppin CM, Gospodarowicz MK, James K, et al. Improved local control of invasive bladder cancer by concurrent cisplatin and preoperative or definitive radiation. The National Cancer Institute of Canada Clinical Trials Group. Journal of Clinical Oncology. 1996 Nov;**14**(11):2901-7.
27 Eustace A, Irlam JJ, Taylor J, et al. Necrosis predicts benefit from hypoxia-modifying therapy in patients with high risk bladder cancer enrolled in a phase III randomised trial. Radiotherapy and Oncology. 2013 Jul;**108**(1):40-7.
28 Tuomikoski L, Collan J, Keyrilainen J, Visapaa H, Saarilahti K, Tenhunen M. Adaptive radiotherapy in muscle invasive urinary bladder cancer--an effective method to reduce the irradiated bowel volume. Radiother Oncol. 2011 Apr;**99**(1):61-6.
29 Burridge N, Amer A, Marchant T, et al. Online adaptive radiotherapy of the bladder: small bowel irradiated-volume reduction. Int J Radiat Oncol Biol Phys. 2006 Nov 1;**66**(3):892-7.
30 Vestergaard A, Sondergaard J, Petersen JB, Hoyer M, Muren LP. A comparison of three different adaptive strategies in image-guided radiotherapy of bladder cancer. Acta Oncol. 2010 Oct;**49**(7):1069-76.
31 Murthy V, Master Z, Adurkar P, et al. 'Plan of the day' adaptive radiotherapy for bladder cancer using helical tomotherapy. Radiother Oncol. 2011 Apr;**99**(1):55-60.
32 Huddart R, McDonald F, Lewis R, Hall E. HYBRID - Evaluating New Radiation Technology in Patients with Unmet Needs. Clin Oncol (R Coll Radiol). 2013 Jun 21.
33 McDonald F, Lalondrelle S, Taylor H, et al. Clinical Implementation of Adaptive Hypofractionated Bladder Radiotherapy for Improvement in Normal Tissue Irradiation. Clinical Oncology. 2013 9//;**25**(9):549-56.
34 Huddart RA, Hall E, Hussain SA, et al. Randomized Noninferiority Trial of Reduced High-Dose Volume Versus Standard Volume Radiation Therapy for Muscle-Invasive Bladder Cancer: Results of the BC2001 Trial (CRUK/01/004). International Journal of Radiation Oncology*Biology*Physics. 2013 10/1/;**87**(2):261-9.
35 Cowan R, McBain C, Ryder W, et al. Radiotherapy for muscle-invasive carcinoma of the bladder: results of a randomized trial comparing conventional whole bladder with dose-escalated partial bladder radiotherapy. Int J Radiat Oncol Biol Phys. 2004;**59**(1):197-207.
36 IDEAL - Image Guided Dose Escalated Adaptive Bladder Radiotherapy. [cited 2013 31/07/2013]; Available from: http://public.ukcrn.org.uk/Search/StudyDetail.aspx?StudyID=7653
37 Hafeez S, Koh M, Sohaib A, Huddart R. The role of functional MRI to characterise tumour boost delineation in patients undergoing radical radiotherapy for muscle invasive bladder cancer (MIBC). NCRI Cancer Conference. Liverpool; 2012.
38 Mangar S, Thompson A, Miles E, Huddart R, Horwich A, Khoo V. A feasibility study of using gold seeds as fiducial markers for bladder localization during radical radiotherapy. Br J Radiol. 2007 Apr;**80**(952):279-83.
39 Chai X, van Herk M, van de Kamer JB, et al. Behavior of lipiodol markers during image guided radiotherapy of bladder cancer. Int J Radiat Oncol Biol Phys. 2010 May 1;**77**(1):309-14.
40 Pos F, Bex A, Dees-Ribbers HM, Betgen A, van Herk M, Remeijer P. Lipiodol injection for target volume delineation and image guidance during radiotherapy for bladder cancer. Radiother Oncol. 2009 Nov;**93**(2):364-7.
41 Basch E, Reeve BB, Mitchell SA, et al. Development of the National Cancer Institute's patient-reported outcomes version of the common terminology criteria for adverse events (PRO-CTCAE). J Natl Cancer Inst. 2014 Sep;**106**(9).
42 Reese P, Pleil A, Okano G, Kelleher C. Multinational study of reliability and validity of the King's Health Questionnaire in patients with overactive bladder. Qual Life Res. 2003 2003/06/01;**12**(4):427-42.
43 Fayers P, NKAaronson, Bjordal K, Groenvold M, Curran D, Bottomley A. EORTC QLQ-C30 Scoring Manual. 3rd ed; 2001.
44 Herdman M, Gudex C, Lloyd A, et al. Development and preliminary testing of the new five-level version of EQ-5D (EQ-5D-5L). Qual Life Res. 2011 Dec;**20**(10):1727-36.
45 Taylor S, Byrne A, Adams R, et al. The Three-item ALERT-B Questionnaire Provides a Validated Screening Tool to Detect Chronic Gastrointestinal Symptoms after Pelvic Radiotherapy in Cancer Survivors. Clin Oncol (R Coll Radiol). 2016 Oct;**28**(10):e139-47.
46 Barajas RF, Jr., Rubenstein JL, Chang JS, Hwang J, Cha S. Diffusion-weighted MR imaging derived apparent diffusion coefficient is predictive of clinical outcome in primary central nervous system lymphoma. AJNR Am J Neuroradiol. 2010 Jan;**31**(1):60-6.
47 Pope WB, Kim HJ, Huo J, et al. Recurrent glioblastoma multiforme: ADC histogram analysis predicts response to bevacizumab treatment. Radiology. 2009 Jul;**252**(1):182-9.
48 Kyriazi S, Collins DJ, Messiou C, et al. Metastatic ovarian and primary peritoneal cancer: assessing chemotherapy response with diffusion-weighted MR imaging--value of histogram analysis of apparent diffusion coefficients. Radiology. 2011 Oct;**261**(1):182-92.
49 Cui Y, Zhang XP, Sun YS, Tang L, Shen L. Apparent diffusion coefficient: potential imaging biomarker for prediction and early detection of response to chemotherapy in hepatic metastases. Radiology. 2008 Sep;**248**(3):894-900.

For peer review only

For peer review only - http://bmjopen.bmj.com/site/about/guidelines.xhtml

50 Harry VN, Semple SI, Gilbert FJ, Parkin DE. Diffusion-weighted magnetic resonance imaging in the early detection of response to chemoradiation in cervical cancer. Gynecologic oncology. 2008 Nov;**111**(2):213-20.
51 Barbaro B, Vitale R, Valentini V, et al. Diffusion-weighted magnetic resonance imaging in monitoring rectal cancer response to neoadjuvant chemoradiotherapy. Int J Radiat Oncol Biol Phys. 2012 Jun 1;**83**(2):594-9.
52 Pearson RA, Thelwall PE, Snell J, et al. Evaluation of early response to neoadjuvant chemotherapy in muscle-invasive bladder cancer using dynamic contrast-enhanced MRI and diffusion weighted MRI: MARBLE study. Journal of Clinical Oncology. 2016;**34**(2_suppl):403-.
53 Messiou C, Collins DJ, Morgan VA, Desouza NM. Optimising diffusion weighted MRI for imaging metastatic and myeloma bone disease and assessing reproducibility. Eur Radiol. 2011 Aug;**21**(8):1713-8.
54 Koh DM, Blackledge M, Collins DJ, et al. Reproducibility and changes in the apparent diffusion coefficients of solid tumours treated with combretastatin A4 phosphate and bevacizumab in a two-centre phase I clinical trial. Eur Radiol. 2009 Nov;**19**(11):2728-38.
55 Bilgili MY. Reproductibility of apparent diffusion coefficients measurements in diffusion-weighted MRI of the abdomen with different b values. Eur J Radiol. 2011 Jul 1.
56 Braithwaite AC, Dale BM, Boll DT, Merkle EM. Short- and midterm reproducibility of apparent diffusion coefficient measurements at 3.0-T diffusion-weighted imaging of the abdomen. Radiology. 2009 Feb;**250**(2):459-65.
57 Yoshida S, Koga F, Kobayashi S, et al. Role of diffusion-weighted magnetic resonance imaging in predicting sensitivity to chemoradiotherapy in muscle-invasive bladder cancer. Int J Radiat Oncol Biol Phys. 2012 May 1;**83**(1):e21-7.
58 Hafeez S, Koh M, Sohaib A, Huddart R. PD-0464: Assessing response to chemotherapy with diffusion weighted MRI (DW-MRI) in muscle invasive bladder cancer (MIBC). Radiotherapy and Oncology. 2013 3//;**106, Supplement 2**(0):S180-S1.
59 Hafeez S, Koh M, Sohaib A, Huddart R. PO-0734: Assessing response to radiotherapy with diffusion weighted MRI (DW-MRI) in muscle invasive bladder cancer (MIBC). Radiotherapy and Oncology. 2014 //;**111, Supplement 1**(0):S34.
60 Hafeez S KM, Sohaib A, Huddart R. Use of diffusion weighted-MRI (DW-MRI) as a biomarker of clinical outcome in muscle invasive bladder cancer (MIBC). NCRI Conference. 2015:http://abstracts.ncri.org.uk/abstract/use-of-diffusion-weighted-mri-dw-mri-as-a-biomarker-of-clinical-outcome-in-muscle-invasive-bladder-cancer-mibc-2/.
61 Hafeez S KM, McQuaid D, Sohaib A, Huddart R. Characterisation of tumour boost with diffusion weighted MRI (DW-MRI) to inform biological target volume for radical radiotherapy in muscle invasive bladder cancer (MIBC). NCRI Conference. 2015:http://abstracts.ncri.org.uk/abstract/characterisation-of-tumour-boost-with-diffusion-weighted-mri-dw-mri-to-inform-biological-target-volume-for-radical-radiotherapy-in-muscle-invasive-bladder-cancer-mibc-2/.
62 Jenkins P, Anjarwalla S, Gilbert H, Kinder R. Defining the clinical target volume for bladder cancer radiotherapy treatment planning. International Journal of Radiation Oncology Biology Physics. 2009 Dec 1;**75**(5):1379-84.
63 Kim JK, Park SY, Ahn HJ, Kim CS, Cho KS. Bladder cancer: analysis of multi-detector row helical CT enhancement pattern and accuracy in tumor detection and perivesical staging. Radiology. 2004 Jun;**231**(3):725-31.
64 Salo JO, Kivisaari L, Lehtonen T. CT in determining the depth of infiltration of bladder tumors. Urol Radiol. 1985;**7**(2):88-93.
65 El-Assmy A, Abou-El-Ghar ME, Mosbah A, et al. Bladder tumour staging: comparison of diffusion- and T2-weighted MR imaging. Eur Radiol. 2009 Jul;**19**(7):1575-81.
66 Weiss E, Hess CF. The impact of gross tumor volume (GTV) and clinical target volume (CTV) definition on the total accuracy in radiotherapy theoretical aspects and practical experiences. Strahlenther Onkol. 2003 Jan;**179**(1):21-30.
67 Njeh CF. Tumor delineation: The weakest link in the search for accuracy in radiotherapy. Journal of medical physics / Association of Medical Physicists of India. 2008 Oct;**33**(4):136-40.
68 Logue JP, Sharrock CL, Cowan RA, Read G, Marrs J, Mott D. Clinical variability of target volume description in conformal radiotherapy planning. Int J Radiat Oncol Biol Phys. 1998 Jul 1;**41**(4):929-31.
69 Hafeez S, Warren-Oseni K, McNair HA, et al. Prospective Study Delivering Simultaneous Integrated High-dose Tumor Boost (</=70 Gy) With Image Guided Adaptive Radiation Therapy for Radical Treatment of Localized Muscle-Invasive Bladder Cancer. Int J Radiat Oncol Biol Phys. 2016 Apr 01;**94**(5):1022-30.

For peer review only - http://bmjopen.bmj.com/site/about/guidelines.xhtml

RAIDER Protocol
ICR-CTSU

For peer review only

This protocol is a controlled document and should not be copied, distributed or reproduced without the written permission of the ICR-CTSU. This document should only be used for the management of patients in the RAIDER trial.

A Randomised phase II trial of Adaptive Image guided standard or Dose Escalated tumour boost Radiotherapy in the treatment of transitional cell carcinoma of the bladder

RADIOTHERAPY PLANNING AND DELIVERY GUIDELINES

Version:4.3

Dated:13/02/2020

Chief Investigator:	Prof Robert Huddart
Sponsor:	The Institute of Cancer Research
Approval:	Cancer Research UK: Clinical Trials Awards& Advisory Committee (CTAAC)
Funders:	Cancer Research UK
Coordinating Trials Unit:	ICR Clinical Trials and Statistics Unit (ICR-CTSU)
	The Institute of Cancer Research

Main REC Reference Number:	15/LO/0539
ISRCTN:	26779187
ClinicalTrials.gov Identifier	NCT02447549
CRUK Reference Number:	CRUK/14/016

The RAIDER trial has been scientifically approved
by Cancer Research UK's Clinical Trials Awards & Advisory Committee (CTAAC)
The RAIDER trial is part of the National Institute for
Health Research Clinical Research Network Trial Portfolio

This is a controlled document which should be referred to in conjunction with the RAIDER protocol and should not be copied, distributed or reproduced without the written permission of the ICR-CTSU. This document should only be used for the management of patients in the RAIDER trial.

For peer review only

RAIDER radiotherapy planning and delivery guidelines

This document sets out guidelines for treatment of patients within the RAIDER trial and should be referred to in combination with the protocol.

This document should only be used for the purpose of the RAIDER trial.

The Trial Management Group reserves the right to amend or add to the radiotherapy guidelines as appropriate. Such changes do not constitute an amendment, and revised guidelines will be circulated to participating centres as needed. Changes between versions will be noted prior to the introduction. Sites treating RAIDER patients for the first time are advised to contact ICR-CTSU to confirm they have the most recent version.

Any questions relating to the detail of the radiotherapy planning and delivery guidelines should be addressed in the first instance to the QA team (See Appendices A& B).

For peer review only

RAIDER radiotherapy planning and delivery guidelines

CONTACT DETAILS

**Scientific Coordination**	**Chief Investigator**<br>Prof Robert Huddart<br>The Royal Marsden NHS Foundation Trust,<br>The Institute of Cancer Research,<br>London,<br>United Kingdom<br><br>Tel: 0208 661 3457<br>Email: Robert.Huddart@icr.ac.uk	**Clinical Coordinator**<br>Dr Shaista Hafeez<br>The Royal Marsden NHS Foundation Trust,<br>The Institute of Cancer Research<br>London<br>United Kingdom<br><br>Tel: 0208 661 3274<br>Email: Shaista.Hafeez@icr.ac.uk
	**TROG lead**<br>Prof Farshad Foroudi<br>Director Radiation Oncology<br>Austin Health<br>Melbourne<br>Australia<br><br>Email: farshad.foroudi@austin.org.au	
**Trial Management**	**ICR Clinical Trials & Statistics Unit**<br>Division of Clinical Studies,<br>The Institute of Cancer Research,<br>Sir Richard Doll Building, Cotswold Road,<br>Sutton, Surrey SM2 5NG<br><br>RAIDER Trial Manager<br>Tel: 0208 722 4613<br>Email: RAIDER-icrctsu@icr.ac.uk	
**Quality Assurance**	**Radiotherapy Trials and Quality Assurance Group:**<br>Amanda Webster<br>Mount Vernon Cancer Centre,<br>Rickmansworth Road,<br>United Kingdom<br><br>Tel: 02038262320<br>Email: raiderqa.enh-tr@nhs.net	

For peer review only - http://bmjopen.bmj.com/site/about/guidelines.xhtml

RAIDER radiotherapy planning and delivery guidelines

CONTENTS

1 INTRODUCTION 12
2 PLANNING CT SCAN FOR RADIOTHERAPY 13
2.1 Patient preparation 13
2.1.1 Group 1, 2 and 3: General Preparation 13
2.1.2 Group 1: Empty Bladder 13
2.1.3 Group 2 and 3: Bladder Filling 13
2.2 Patient positioning 13
2.3 Planning CT acquisition, scan limits and slice thickness 13
2.4 Planning CT export, fusion and evaluation of bladder filling 13
3 LOCALISATION OF THE TARGET VOLUME AND ORGANS AT RISK 15
3.1 Volume definition 15
3.2 Gross tumour volume 15
3.3 Clinical target volume 15
3.4 Organs at risk 15
3.5 Planning target volume 16
3.5.1 Group 1: standard whole bladder RT 16
3.5.2 Group 2 and 3: adaptive tumour focused RT (standard dose and dose escalated) 16
Table 1: CTV to PTV Expansion Details 17
Table 2: GTV to PTV2 Expansion details (group 2 and 3 only) 18
3.6 Voluming Checklist 18
3.7 Nomenclature 22
Table 3: RAIDER Trial Target Volume and OAR Nomenclature 22
4 PLANNING 24
4.1 Standard Planning 24
4.2 Adaptive Tumour Focused Planning 24
Table 4: Prescription doses 25
4.3 Target volume dose objectives 25
Table 5: Target dose objectives 25
4.4 Normal tissue dose constraints 26
Table 6a: OAR Dose Constraints for 32-fraction schedule 26
Table 6b: OAR Dose constraints for 20-fraction schedule 27
4.5 Planning recommendations 28
4.6 Pre-Treatment checks 28
5 TREATMENT DELIVERY 29
5.1 Standard whole bladder RT (group 1) 29

For peer review only - http://bmjopen.bmj.com/site/about/guidelines.xhtml

RAIDER radiotherapy planning and delivery guidelines

5.2 Adaptive Tumour Focused RT (SART and DART) 29
5.2.1 Image Match and Plan Selection Steps: 29
5.2.2 Image Match and Plan Selection Tips: 30
5.3 Post treatment 31
5.4 On completion of radiotherapy 31
6 TREATMENT SCHEDULING AND GAPS 31
APPENDIX A: 33
United Kingdom RAIDER QA Programme 33
APPENDIX B: 36
Trans Tasman Radiation Oncology Group: Australia & NZ 36
APPENDIX C: 40
PRE-TRIAL OUTLINING BENCHMARK CASE 40
Outlining Benchmark Case 1: T2N0M0 Male Pelvis 40
Outlining Benchmark Case 2: T2N0M0 Female Pelvis 42
PRE-TRIAL PLANNING BENCHMARK CASE 43
Benchmark Planning Case: T2N0M0 TCC male pelvis 43
APPENDIX D 45
APPENDIX E 46
APPENDIX F 47
APPENDIX G 52
APPENDIX H 53
APPENDIX I 54

For peer review only - http://bmjopen.bmj.com/site/about/guidelines.xhtml

RAIDER radiotherapy planning and delivery guidelines

VERSION CHANGES

V1.0 - Original RT planning and delivery guidelines
V2.0 – RT planning and delivery guidelines updated to include TROG QA appendix and contact details.
V3.0- The following changes have been made:

- Section 2.4 (Pg 11) and Section 5 (Pg 20)- Clarification provided on what CT30 and CT60 are to be used for
- Section 5 Table 4 & 5 (Pg 21/22)- Clarification regarding which target volumes the dose objectives are for
- Section5: Table 5 (Pg 22)- D50% +/- 1% for PTV2 moved from "Optimal" to "Mandatory"
- Section 5.5 (Pg 24/25)- Planning recommendations added based on pre-trial QA exercise
- Section 7.4 (Pg 28)- Recommendations for exporting CBCT data from Aria/Mosaiq
- Appendix A: QA Programme UK – Pre-trial point 7: IGRT training video now provided
- Appendix B: TROG QA Programme- QA programme updated with IGRT training/assessment information

V4.0- The following changes have been made:

- Section 1 (Pg 10) Clarification on the start date of radiotherapy
- Section 2.3 (Pg 11) Clarification on contrast. Contrast is permitted; however it is recommended to perform the planning CT scan without it
- Section 3.5 (Pg 14) Clarification on treatment volumes for each randomisation group
- Section 3.6 (Pg 16) NEW voluming checklist with clarification on outlining the GTV and CTV
- Section 3.7 (Pg 20) Nomenclature table updated
- Section 4 (Pg 22) Clarification on actions to take when replanning required
- Section 4.3 (Pg 2) Clarification on target dose objectives, with emphasis placed on PTV2
- Section 4.4 (Pg 24) Clarification on normal tissue dose constraints
- Section 4.4 (Pg 24/25) Table 6a and 6b updated to include new optimisation targets for the CTV
- Section 4.5 (Pg 25) Planning recommendations updated, with emphasis placed on PTV2
- Section 4.6 (Pg 26) Further clarification and guidance for pre-treatment checks
- Section 5 (Pg 27) Treatment delivery updated
- Section 5.2 (Pg 27/28) NEW guidance on Adaptive Tumour Focused Radiotherapy (SART & DART)
- Section 5.2.1 (Pg 27/28) NEW plan selection steps
- Sections 5.2.2 (Pg 28) NEW plan selection tips
- Section 5.3 (Pg 28) Clarification on post-treatment CBCTs
- Appendix A: QA Programme UK
    - On trial QA updated – site visits removed and IGRT/POD selection support introduced
    - Clarification on data export
    - NEW Table 7: RAIDER data checklist
- Appendix B: TROG QA Programme
    - On trial QA updated to include IGRT/POD selection support
- Appendix C: QA Benchmark Cases for Outlining and Planning moved to Appendix C
    - Additional diagnostic information for outlining case 1 included (NEW)
- Appendix D: Quick Contouring Checklist (NEW)
- Appendix E: Clarification on treatment interventions
- Appendix F: Text updated to be in line with Section 5.2
- Appendix G: Quick Reference Guide for Exporting CBCTs from ARIA (NEW)
- Appendix H: Quick Reference Guide for Exporting CBCTs from MOSAIQ (NEW)

V4.1- The following changes have been made:

- Section 4.4: Clarification on updated (V4.0) Normal Tissue Dose Constraints

V4.2 - The following changes have been made:

- Front cover updated (Pg 1/2) RAIDER Guidelines only to be used for the purpose of RAIDER

For peer review only - http://bmjopen.bmj.com/site/about/guidelines.xhtml

RAIDER radiotherapy planning and delivery guidelines

- Trial Management Details (Pg 3) ICR-CTSU contact details upated
- RAIDER TRIAL SUMMARY (Pg 7) Recuitment Target updated
- Trial Schema (Pg 9) Updated
- Section 1 (Pg 10) A list of RAIDER QA credentialed staff must retained in site investigator file
- Section 4.4 (Pg 24) Clarification do not compromise dose to PTV2 or PTV in order to meet the normal tissue dose constraints
- Section 5.2.1 (Pg 27-28) Clarification on Image Match and Plan Selection Steps:
    - Consider magnitude of soft tissue shift and review if over 1cm
- Section 5.2.2 (Pg 28-29) Clarification on Image Match and Plan Selection Tips:
    - Fractions must not be omitted or missed due to unfavourable positioning of normal anatomy
    - Review post-treatment CBCTs for intrafraction filling
- Section 6 (Pg 29-30) Clarification on Treatment Scheduling and Gaps:
    - Avoid gaps where possible
    - Further guidance when staff unavailable/machine breakdown
    - Compensation not expected due to toxicity
    - Involved PI
- Appendix G (Pg 50) Additional information for Aria Export

V4.3 - The following changes have been made:

- Appendix A (Pg 33) Data transfer details updated for RTTQA
- Appendix B (Pg 37) Data export and upload updated for TROG
- Appendix G (Pg 51) Please contact RTTQA for information on suitable anonymization software for CBCTs from ARIA
- Appendix I (Pg 53) Please contact RTTQA for information when exporting CBCTs from Raystation

For peer review only - http://bmjopen.bmj.com/site/about/guidelines.xhtml

RAIDER radiotherapy planning and delivery guidelines

RAIDER TRIAL SUMMARY

PROTOCOL TITLE	A Randomised phase II trial of Adaptive Image guided standard or Dose Escalated tumour boost Radiotherapy in the treatment of transitional cell carcinoma of the bladder
TARGET DISEASE	Muscle invasive bladder cancer
STUDY OBJECTIVES	To define a feasible and safe adaptive dose escalated tumour boost radiotherapy schedule for MIBC; to investigate the ability to deliver daily adaptive bladder radiotherapy and assess the impact of delivery on patient reported outcomes and health economic related measures.
STUDY DESIGN	Multicentre two stage, three arm phase II randomised controlled trial
TRIAL POPULATION	Patients receiving radical radiotherapy for muscle invasive bladder cancer
RECRUITMENT TARGET	Minimum 120 in each of two fractionation cohorts i.e. sufficient to accrue 57 evaluable DART patients per cohort
TRIAL TREATMENT	Patients will be randomised (1:1:2) between:<br>1. Standard whole bladder radiotherapy delivery (WBRT) (control)<br>2. Standard dose Adaptive tumour focused radiotherapy (SART)<br>3. Dose escalated Adaptive tumour boost radiotherapy (DART)<br>64Gy/32f and 55Gy/20f fractionation schedules are permitted. Participants in all groups will be permitted to receive concomitant radio sensitising chemotherapy. Full blood count (FBC), urea and electrolytes (U&Es) and acute toxicity will be assessed during radiotherapy. Participants in the Patient Reported Outcomes (PRO) sub-study will be asked to complete questionnaire prior to trial entry and at the end of radiotherapy.
PRIMARY ENDPOINT	Stage I: Proportion of patients meeting radiotherapy dose constraints to bladder, bowel and rectum in DART groups.<br>Stage II: Proportion of patients experiencing any ≥Grade 3 Common Terminology Criteria for Adverse Events (CTCAE) v.4 late toxicity (6-18 months post radiotherapy).
SECONDARY ENDPOINTS	Stage I:<br>• Recruitment rate<br>• Ability to deliver SART and DART<br>Stage II:<br>• Clinician reported acute toxicity<br>• PRO: acute and late bladder and bowel/rectal symptoms;

For peer review only

For peer review only - http://bmjopen.bmj.com/site/about/guidelines.xhtml

	• Health economic related measures: time for outlining, plan generation, selection and delivery, NHS resource usage subsequent to treatment;<br>• Loco-regional MIBC control<br>• Progression-free survival<br>• Overall survival
EXPLORATORY ENDPOINTS	Image Guided Radiotherapy (IGRT) endpoints:<br>• Use of adaptive plans<br>• Target coverage<br>• Online/offline concordance<br>• Dose volume analysis of adaptive vs. standard planning
FOLLOW UP	Participants will subsequently be assessed at the following intervals:<br>**6 weeks from start of radiotherapy (20f cohort only)**<br>Assessment of acute toxicity (CTCAE v.4)<br>**10 weeks from start of radiotherapy:**<br>Assessment of acute toxicity (CTCAE v.4)<br>**3 months from end of radiotherapy:**<br>Rigid cystoscopy and biopsy of tumour bed, FBC, U&Es, chest x-ray (CXR), acute toxicity (CTCAE), PRO questionnaire (if participating in sub-study).<br>**6 months from end of radiotherapy:**<br>Flexible cystoscopy, FBC, U&Es, CXR or CT chest, CT abdomen and pelvis, late toxicity (CTCAE, RTOG), PRO(if participating in sub-study)<br>**9months from end of radiotherapy:**<br>Flexible cystoscopy, late toxicity<br>**12months from end of radiotherapy:**<br>Flexible cystoscopy, CT abdomen and pelvis, CXR or CT chest, late toxicity, PRO(if participating in sub-study)<br>**18months from end of radiotherapy:**<br>Flexible cystoscopy, CXR or CT chest, late toxicity, PRO(if participating in sub-study)<br>**24 months from end of radiotherapy:**<br>Flexible cystoscopy, CT abdomen and pelvis, CXR or CT chest, late toxicity, PRO(if participating in sub-study)<br>**Yearly to year 5:** Flexible cystoscopy, CXR or CT chest, late toxicity

For peer review only

For peer review only - http://bmjopen.bmj.com/site/about/guidelines.xhtml

Annually thereafter: Survival and disease status

For peer review only

For peer review only - http://bmjopen.bmj.com/site/about/guidelines.xhtml

RAIDER radiotherapy planning and delivery guidelines

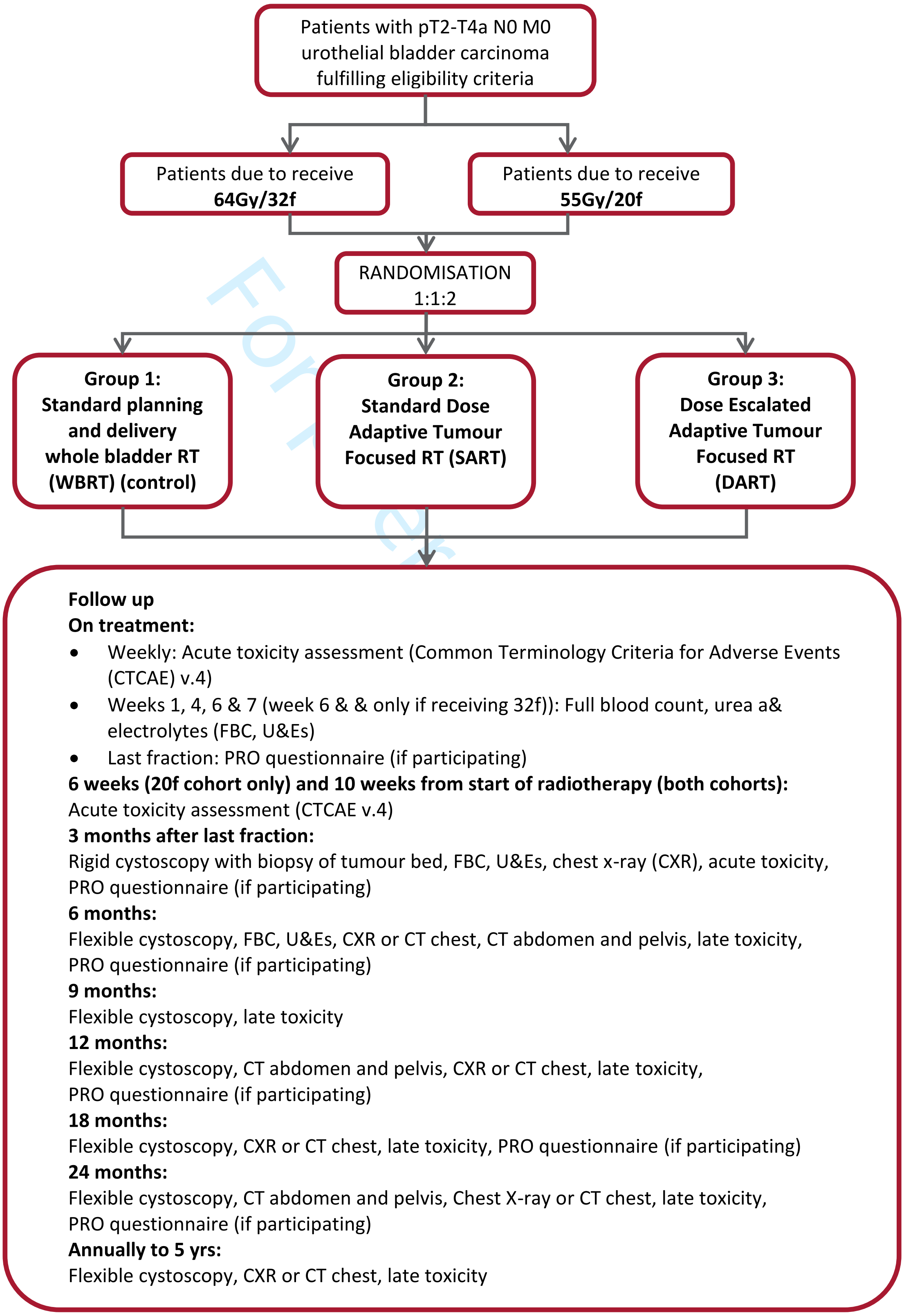

For peer review only - http://bmjopen.bmj.com/site/about/guidelines.xhtml

RAIDER radiotherapy planning and delivery guidelines

1 INTRODUCTION

All patients will be planned to receive radical daily radiotherapy delivered in 32 or 20 fractions. Patients will be randomised between 3 groups;

1. Standard Whole Bladder Radiotherapy (WBRT) (control)
2. Standard dose Adaptive tumour focused Radiotherapy (SART)
3. Dose escalated Adaptive tumour boost Radiotherapy (DART)

In all 3 groups concomitant therapy is permitted at the local investigators discretion.

Radiotherapy should ideally commence within 6 weeks following randomisation. However up to 10 weeks is permitted, to allow sufficient time for chemotherapy and/or radiotherapy planning

Participants allocated to standard planning and delivery (control arm, ***group 1***) will have:

- One (1) radiotherapy plan generated to deliver all fractions on an empty bladder.

The IGRT process is as detailed in the NRIG IGRT 2012 report for this patient group. The NRIG IGRT report contains detailed guidelines for IGRT practice in UK. (It is in the RAIDER trial documents on the TROG website for reference).

Participants allocated to adaptive planning (***group 2 and 3***) will have:

- Three (3) radiotherapy plans generated (small, medium and large).
- A simultaneous integrated boost delivered at conventional dose (group 2) or dose escalation (group 3) to the bladder tumour in a single phase IMRT technique.
- All fractions treated on a partially filled bladder.
- A cone beam CT (MV or kV) taken prior to each treatment delivery to select the most appropriate 'plan of the day' depending on the bladder volume size.

A comprehensive QA programme will be implemented for the RAIDER trial. This will include pre-accrual and during accrual components. Selection of appropriate treatment plans for the adaptive planning group will be independently monitored as part of the on-going RTQA process.

Plan selection is authorised to be carried out only by site personnel who have attained concordance with the gold standard for PTV selection through either RTTQA IGRT QA credentialing for UK centres and TROG IGRT credentialing for Australia/NZ. This is to ensure they have the advanced level skills required for the study. A record of QA credentialed staff should be retained in the site investigator file.

This document should only be used for the management of patients in the RAIDER trial.

For peer review only - http://bmjopen.bmj.com/site/about/guidelines.xhtml

2 PLANNING CT SCAN FOR RADIOTHERAPY

2.1 Patient preparation

2.1.1 Group 1, 2 and 3: General Preparation

Ideally all patients should be encouraged to empty the rectum of flatus and faeces. The routine use of micro enemas (e.g. relaxit) is permissible if it is standard local practice.

Bladder preparation is dependent on patient randomisation.

2.1.2 Group 1: Empty Bladder

Ensure patient has an empty bladder. Therefore all patients should be asked to void immediately before planning CT scan (CT0) is performed and not to drink fluids for 30minutes before the scan.

2.1.3 Group 2 and 3: Bladder Filling

30 minutes prior to the planning CT scan, patients are instructed to empty their bladder and then drink 350ml of water. Two planning scans will be acquired to inform pattern of bladder filling over time:

- The first scan will be at 30 minutes (CT30) following drinking
- The second scan will be at 60 minutes (CT60) following drinking.

Voiding is not permitted between the 2 scans; however if unavoidable, the CT30 scan should be used for planning.

2.2 Patient positioning

All patients will be scanned and treated supine with arms displaced out of the radiotherapy field, using appropriate immobilisation techniques.

2.3 Planning CT acquisition, scan limits and slice thickness

Planning CT scans will be performed in the treatment position at CT slice thickness 3mm or less. It is recommended that the planning CT is performed without contrast, but the use of contrast is permitted if it is local clinical practice. Recommended scanning levels are at least 4cm above the dome of the bladder to 2cm below ischial tuberosities.

For patients treated in group 2 and 3, the planning CT scan is performed (as above) at 'Time=30 minutes' (scan 1, CT30). A second planning CT is acquired at 'Time= 60 minutes' (scan 2, CT60) from end of drinking. The time of each scan is recorded.

2.4 Planning CT export, fusion and evaluation of bladder filling

The planning CT scan is exported via DICOM transfer to the treatment planning system for localization.

For patients treated in group 2 and 3 the bladder filling must be assessed. In order to do so fuse both planning CT scans in the treatment planning system. CT30 will be the primary data set and CT60 will be the fused secondary dataset. To evaluate bladder filling, the whole bladder is localized on the CT30 and on the CT60 scan.

For peer review only - http://bmjopen.bmj.com/site/about/guidelines.xhtml

RAIDER radiotherapy planning and delivery guidelines

- If the difference in bladder filling between the 2 planning scans is less than 50ml i.e. no significant filling occurs all contours are to be created on CT30.
- If difference is greater than 50mls i.e. filling occurs, the large GTV and CTV will be created on CT60.These shall be used to create PTV2 and PTV for the large plan

Please note all planning and dose calculation is to be done on CT30 (See section 4), therefore all volumes should be assigned to a single structure set on the CT30 scan.

For peer review only

For peer review only - http://bmjopen.bmj.com/site/about/guidelines.xhtml

RAIDER radiotherapy planning and delivery guidelines

3 LOCALISATION OF THE TARGET VOLUME AND ORGANS AT RISK

3.1 Volume definition

Volumes will be defined according to the International Commission on Radiation Units and Measurements (ICRU) report 50, supplement report ICRU 62: Prescribing, Recording and Reporting Photon Beam Therapy and ICRU 83: Prescribing, Recording and Reporting Photon-Beam Intensity Modulated Radiotherapy (IMRT). Outlining should be carried out with the aid of all diagnostic information including position of fiducial markers, surgical bladder map, MRI and CT scans.

3.2 Gross tumour volume

The gross tumour volume (GTV) is defined as the bladder tumour/bed. It will be delineated using position of fiducial markers (where available), diagnostic imaging (imaging prior to neoadjuvant chemotherapy where applicable) and the surgical bladder map (where available). When delineating the tumour any extravesical tumour should be included in GTV as should pathological bladder wall thickening unless clearly not due to tumour. If no tumour is visible, the appropriate section of the bladder should be included based on surgical bladder map +/- discussion with urologist who performed TURBT. In these circumstances consider repeating TURBT and placing fiducial markers adjacent to resection scar whenever possible.

For patients treated in group 2 and 3, if the difference in bladder filling between CT30 and CT60 is greater than 50ml i.e. filling occurs, GTV is to be contoured on both scans.

3.3 Clinical target volume

The clinical target volume (CTV) is the contour encompassing the tumour/bed (GTV), the whole bladder and any area of extravesical spread. The CTV should also include 1.5cm of prostatic urethra in males or 1cm of urethra in females if tumour is at the base of bladder or if distant CIS is present.

For patients treated in group 2 and 3, if the difference in bladder filling between CT30 and CT60 is greater than 50ml i.e. filling occurs, CTV is to be contoured on both scans.

3.4 Organs at risk

Organs at risk (OAR) will be outlined as solid structures by defining their outer wall. All OAR should be outlined on the CT0 for group 1 and CT30 only for group 2 and 3. The following OARs should be contoured:

- Rectum:

  The rectum is outlined to include the full circumference and rectal contents. Outlining should extend from the lowest level of ischial tuberosities to the recto-sigmoid junction. The recto-sigmoid junction will be defined as the level at which there is an anterior inflection of the bowel, best appreciated on sagittal reconstructions on the CT planning scan.

For peer review only - http://bmjopen.bmj.com/site/about/guidelines.xhtml

RAIDER radiotherapy planning and delivery guidelines

- Other bowel:

The small and large bowel (including sigmoid colon) will be outlined as a single structure. The entire small and large bowel visible on relevant levels of the planning scan will be outlined as individual bowel loops. The superior extent of outlining should be 2cm beyond the superior extent of PTV (group 1) and Large PTV (group 2 and 3).

- Femoral heads:

Both the femoral heads are outlined to the bottom of the curvature of their heads (femoral necks are not included).

For peer review only

3.5 Planning target volume

The CTV(s) will be expanded, as below, to create the PTV(s).

For the tumour focused RT (group 2 and 3) the GTV(s) will be expanded, as below to create the PTV2s.

3.5.1 Group 1: standard whole bladder RT

For patients in group 1, receiving standard whole bladder RT, a single PTV will be created.

1. PTV= CTV with anisotropic margin applied, as per Table 1.

3.5.2 Group 2 and 3: adaptive tumour focused RT (standard dose and dose escalated)

For patients in group 2 and 3, receiving adaptive tumour focused RT a library of 3 PTVs will be created, from the CTV(s) (Table 1):

1. PTV_Sm

2. PTV_Med

3. PTV_Lar

- For patients in group 2 and 3, if the difference in bladder filling between CT30 and CT60 is less than 50ml i.e. filling does not occur, the PTV_Sm, PTV_Med and PTV_Lar will be produced from the 30 minute CTV.

- However, if the difference in bladder filling between CT30 and CT60 is greater than 50ml i.e. filling occurs, the PTV_Sm and PTV_Med will be produced from the 30 minute CTV. The PTV_Lar will be produced from the 60 minute CTV, as described in Table 1.

For peer review only - http://bmjopen.bmj.com/site/about/guidelines.xhtml

RAIDER radiotherapy planning and delivery guidelines

Table 1: CTV to PTV Expansion Details

Patient Randomisation	CT data set	PTV	CTV to PTV Expansion (cm)				
			Laterally	Anteriorly	Posteriorly	Superiorly	Inferiorly
Group 1<br>Standard Whole Bladder	CT0	PTV	0.8	1.5	1.2	1.5	0.8
Group 2 and 3<br>Adaptive Tumour Focused	CT30	PTV_Sm	0.5	0.5	0.5	0.5	0.5
	CT30	PTV_Med	0.5	1.5	1.0	1.5	0.5
	If CT60-CT30 <50mls then						
	CT30	PTV_Lar_30	0.8	2.0	1.2	2.5	0.8
	If CT60-CT30>50mls then						
	CT60	PTV_Lar_60	0.5	1.5	1.0	1.5	0.5

Additionally 3 PTV2s will also be created, from the GTV(s) (Table 2):

1. PTV2_Sm
2. PTV2_Med
3. PTV2_Lar

- For patients in group 2 and 3, if the difference in bladder filling between CT30 and CT60 is less than 50ml i.e. filling does not occur, the PTV2_Sm, PTV2_Med and PTV2_Lar will be produced from the 30 minute GTV.
- However, if the difference in bladder filling between CT30 and CT60 is greater than 50ml i.e. filling occurs, the PTV2_Sm and PTV2_Med will be produced from the 30 minute GTV. The PTV2_Lar will be produced from the 60 minute GTV, as described in Table 2.

For peer review only

For peer review only - http://bmjopen.bmj.com/site/about/guidelines.xhtml

Table 2: GTV to PTV2 Expansion details (group 2 and 3 only)

**Patient Randomisation**	**CT data set**	**PTV2**	**GTV to PTV2 Expansion (cm)**				
			Laterally	Anteriorly	Posteriorly	Superiorly	Inferiorly
Group 1 Standard Whole Bladder	Not applicable						
Group 2 and 3 Adaptive Tumour Focused	CT30	PTV2_Sm	0.5	0.5	0.5	0.5	0.5
	CT30	PTV2_Med	0.5	1.5	1.0	1.5	0.5
	If CT60-CT30 <50mls then						
	CT30	PTV2_Lar_30	0.8	2.0	1.2	2.5	0.8
	If CT60-CT30>50mls then						
	CT60	PTV2_Lar_60	0.5	1.5	1.0	1.5	0.5

3.6 Voluming Checklist

1. For group 2 and 3, the inferior border of the CTV should be the same on both the CT30 and CT60 following fusion, Figure 1.

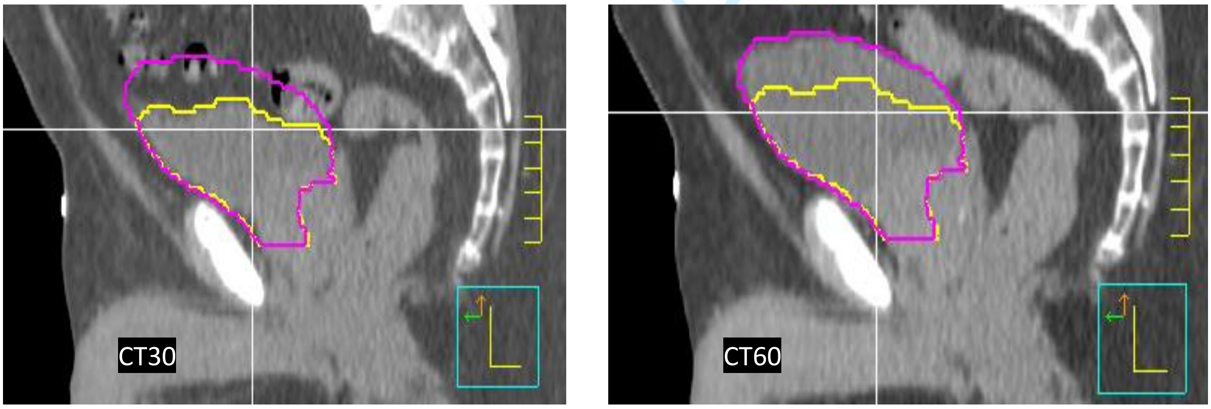

Figure 1: Inferior border of CTV anticipated to be at same level for both scans. Can be achieved by copying inferior contour on fused scan. This ensures that changes in volume are due to filling and not to variation/contouring error between the 2 scans.

For peer review only - http://bmjopen.bmj.com/site/about/guidelines.xhtml

RAIDER radiotherapy planning and delivery guidelines

2. For Groups 1, 2 and 3 please ensure that 1.5cm of prostatic urethra in males or 1cm of urethra in females is included when the tumour is at the bladder base or if distant CIS is present. Please contact RTTQA if further guidance is required on this aspect of outlining.

3. For Group 2 and 3, the CTV drawn on CT60 should not be within/smaller than the CTV drawn on the CT30. Thus, the CTV drawn on CT60 should encompass the CTV drawn on CT30. This could occur as a result of contouring error, however it is also possible that bowel motion/filling between the 2 scans may also cause this (see Figures 2a-2d). In these circumstances it is advised that the CTV drawn on CT60 is summed to include the CTV drawn on CT30; allowing all excursions of the bladder to be included in the volume. This can be done by application of Boolean operators within the treatment planning system used.

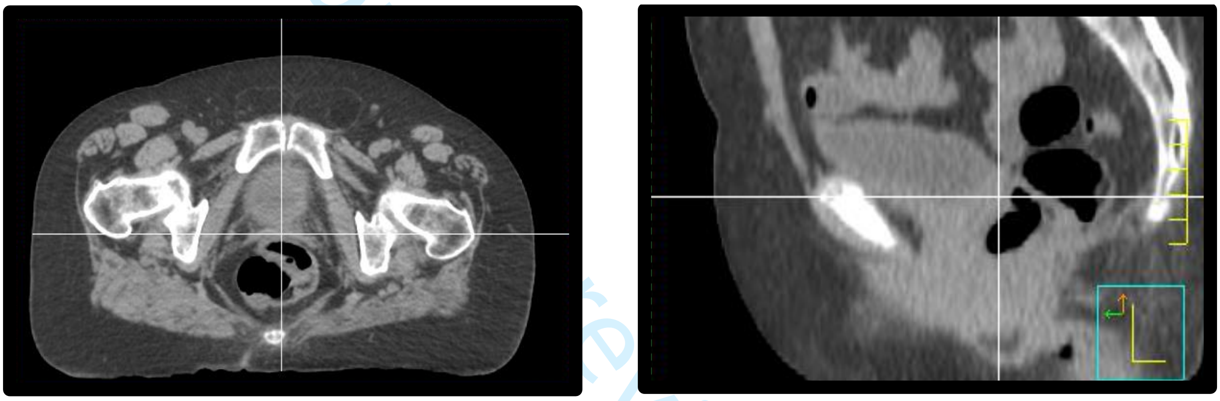

Figure 2a: CT30 scan.

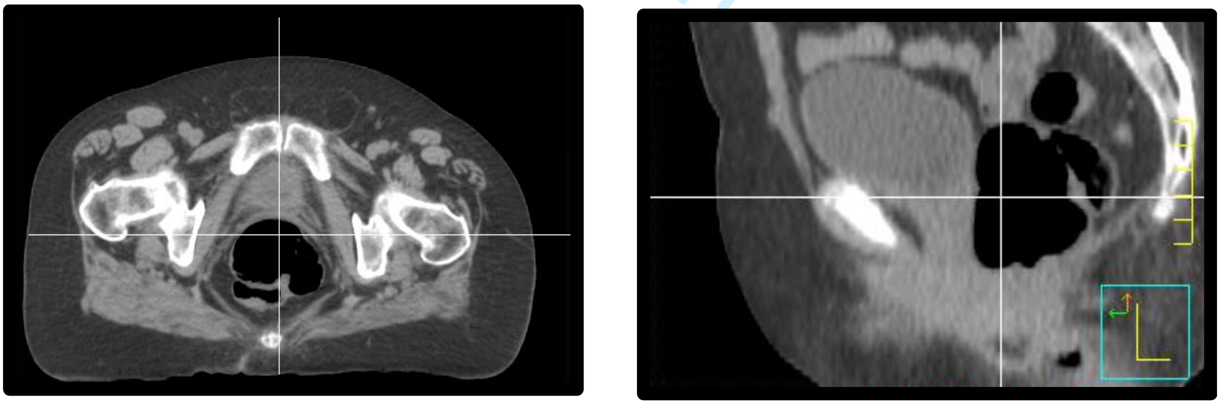

Figure 2b: CT60 scan. Bladder filling occurs but is also pushed anterior due to rectal distension secondary to flatus

For peer review only - http://bmjopen.bmj.com/site/about/guidelines.xhtml

RAIDER radiotherapy planning and delivery guidelines

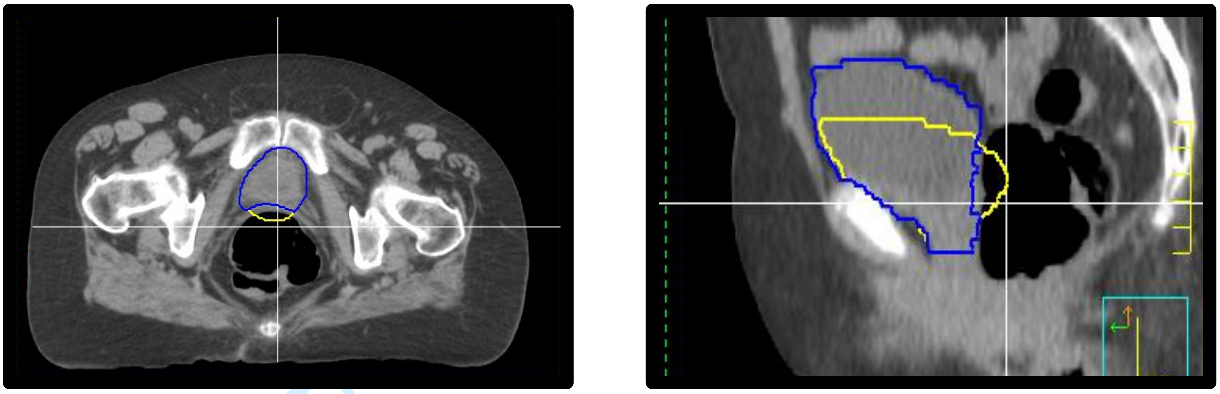

Figure 2c: Yellow contour reflects CTV on CT30. Blue contour reflects CTV on CT60. (Difference between two volumes >50cc).

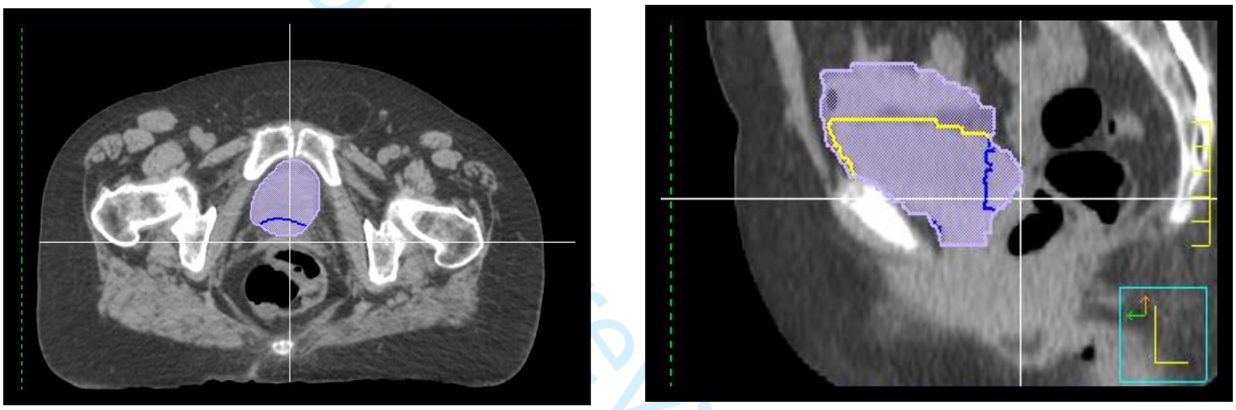

Figure 2d: Boolean operator applied to create summed CTV (purple) from which PTV_Lar_60 (and PTV2_Lar_60) will be created.

4. The GTV should not exceed beyond the CTV outline. Where there is extra-vesical spread included in the GTV, the CTV must be extended outside the bladder wall to include all the GTV. The CTV should be summed to include all the GTV and should share same outer-contour, see Figure 3a-3c.

- The CTV_30 should encompass the GTV_30 and share the same outer contour.
- The CTV_60 should encompass the GTV_60 (and thus the GTV_30) and should share the same outer contour.

For peer review only - http://bmjopen.bmj.com/site/about/guidelines.xhtml

RAIDER radiotherapy planning and delivery guidelines

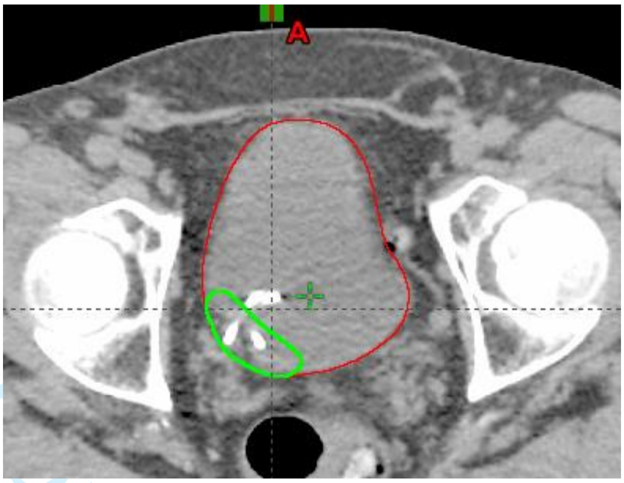

Figure 3a: CT30 GTV (green) shares same bladder wall as CTV (red)

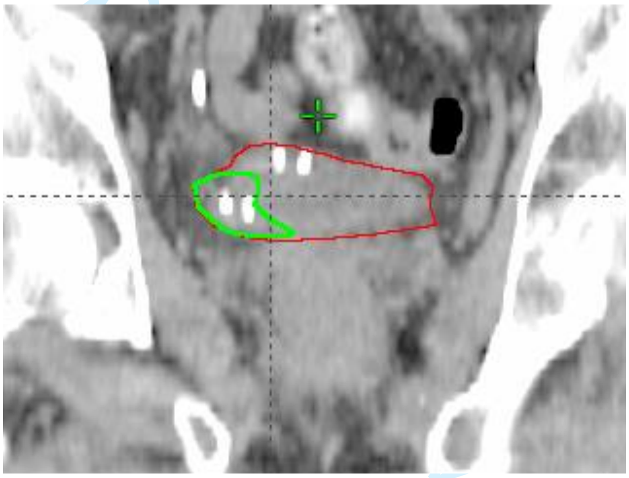

Figure 3b: CT30 GTV (green) shares same bladder wall as CTV (red)

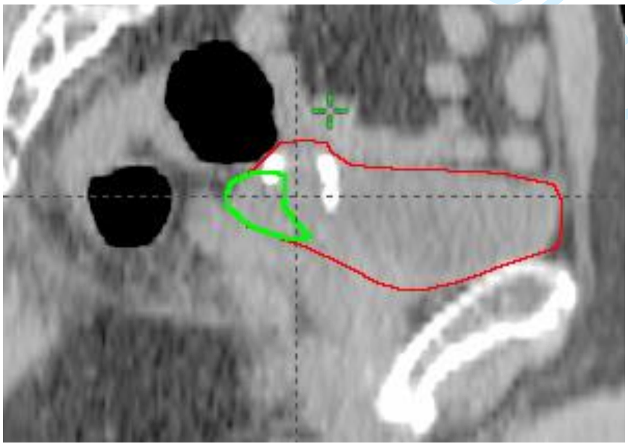

Figure 3c: CT30 GTV (green) shares same bladder wall as CTV (red)

For peer review only - http://bmjopen.bmj.com/site/about/guidelines.xhtml

5. The GTV_60 should generally extend further superiorly and anteriorly, in comparison with the GTV_30, to account for expansion upon filling.

6. The PTV_Lar should encompass the PTV_Med.

7. The PTV2 should not exceed beyond the corresponding PTV outline. For planning purposes, the PTV2 should share the same outer contour as the corresponding PTV outline, i.e. it should 'appear' attached to the outer contour of the PTV.

8. The clinician should indicate to the planner which CT data set (CT30 or CT60) has been used to create the large target volumes.

9. The OTHER_BOWEL should extend to 2cm beyond the largest PTV.

10. The rectum should extend to the lowest level of the ischial tuberosities.

A "Quick Contouring Checklist" has been provided in Appendix E to ensure the above has been successfully achieved.

3.7 Nomenclature

Consistent naming of contoured structures used in radiotherapy treatment planning is essential to facilitate the comparison of dose-volume statistics across patients for quality assurance and outcomes analysis. Maintaining consistency in structure names is particularly important (and challenging) in multi-institutional clinical trials, in which treatment planning data are collected from many participating institutions. A scheme for uniform naming of contoured structures for RAIDER is provided in the following table. The following names must be used for treatment planning of all trial patients.

Table 3: RAIDER Trial Target Volume and OAR Nomenclature

Structure Name	Description
GTV_0	Contouring the primary bladder tumour for patients randomised to Group 1 (WBRT) is not required for trial purposes. Local standard practice should be followed if contouring GTV aids subsequent CTV delineation (on CT_0)
GTV_30	Primary bladder tumour contoured on CT30 for group 2 and 3

For peer review only

RAIDER radiotherapy planning and delivery guidelines

GTV_60	Primary bladder tumour contoured on CT60 for group 2 and 3 if the difference in bladder filling between CT30 and CT60 is greater than 50ml
CTV_0	GTV + whole bladder + extravesical spread (+ 1.5cm of prostatic urethra in males or 1cm of urethra in females if tumour is at the base of bladder or if distant CIS is present) contoured on CT0 for group 1
CTV_30	GTV + whole bladder + extravesical spread (+ 1.5cm of prostatic urethra in males or 1cm of urethra in females if tumour is at the base of bladder or if distant CIS is present) contoured on CT30 for group 2 and 3
CTV_60	GTV + whole bladder + extravesical spread (+ 1.5cm of prostatic urethra in males or 1cm of urethra in females if tumour is at the base of bladder or if distant CIS is present) contoured on CT60 for group 2 and 3
PTV_Std	CTV_0 + anisotropic margin (see table 1)<br>**For Standard Whole Bladder-group 1 only**
PTV_Sm	CTV_30 + margin
PTV_Med	CTV _30 + margins
PTV_Lar_30	CTV_30 +margins, **when no filling occurs between CT30 and CT60)**
PTV_Lar_60	CTV_60 +margins, **when filling occurs between CT30 and CT60)**
PTV2_Sm	GTV_30 + margin
PTV2_Med	GTV_30 +margins
PTV2_Lar_30	GTV_30 + margins, **when no filling occurs between CT30 and CT60)**
PTV2_Lar_60	GTV_60 + 0.5 – 1.5cm margins, **when filling occurs between CT30 and CT60)**
PTV_Sm-PTV2_Sm	PTV_Sm minus PTV2_Sm
PTV_Med-PTV2_Med	PTV_Med minus PTV2_Med
PTV_Lar-PTV2_Lar	PTV_Lar minus PTV2_Lar
RECTUM	Rectum
OTHER_BOWEL	Small and large bowel (including sigmoid colon)
FEMORALJOINT_L (UK) FemJoint_L (Aus)	Left femoral head (ball only)
FEMORALJOINT_R (UK)FemJoint_R (Aus)	Right femoral head (ball only)

For peer review only - http://bmjopen.bmj.com/site/about/guidelines.xhtml

4 PLANNING

It is mandatory for all RAIDER patients to be CT planned. In general, the method of treatment planning will vary from site to site and should be specified in each centre's process document.

For group 1 the use of three or four 3D conformal radiotherapy (3DCRT) fields, five to seven static fields intensity modulated radiotherapy (IMRT) or volumetric modulated radiotherapy (VMAT) treatments (e.g. RapidARC, VMAT, Tomotherapy) are acceptable. Please inform the relevant QA group and the CTU if a group 1 patient requires re-planning (or re-scanning) during treatment.

For group 2 and 3 the aim of the plan is to do partial bladder sparing while maintaining the PTV prescription dose, use of 5-7 static fields IMRT (5 fields are preferred due to shorter treatment time) or VMAT is recommended. The use of forward planned simple IMRT ('field in field') or tomotherapy are also acceptable alternatives. The same technique should be used for all patients randomised to group 2 and 3. All plan and dose calculation is to be done on CT30 irrespective of filling.

Please inform the relevant QA group and the CTU if a group 2 or 3 patient requires re-planning (or re-scanning) during treatment.

The local investigator should ensure appropriate quality assurance methodologies are in place for the chosen planning technique.

4.1 Standard Planning

For patients randomised to group 1 standard planning a single plan will be created.

- Plan_Std = PTV_Std

4.2 Adaptive Tumour Focused Planning

For those randomised to adaptive planning a series of 3 plans will be created using the PTV Small, PTV Medium and PTV Large and the corresponding PTV2. The isocentre for all 3 plans must be identical.

- Plan_Sm = PTV_Sm& PTV2_Sm
- Plan_Med = PTV_Med& PTV2_Med
- Plan_Lar = PTV_Lar_30 and PTV2_Lar_30 or PTV_Lar_60 and PTV2_Lar_60, depending on filling.

For IMRT planning centres may prescribe plan to either a mean or median dose to PTV2, depending on their normal practice, as it is anticipated in an optimised plan the difference between these two parameters will be minimal. For 3D-CRT the prescription should be 100% at the ICRU reference point.

The prescription doses for RAIDER patients are detailed in Table 4.

For peer review only - http://bmjopen.bmj.com/site/about/guidelines.xhtml

RAIDER radiotherapy planning and delivery guidelines

Table 4: Prescription doses

Patient Randomisation	Volume	Dose (Gy)	Fractions	Dose per fraction (Gy)	Dose (Gy)	Fractions	Dose per fraction (Gy)
**Group 1** **Standard plan**	PTV_Std	64	32	2	55	20	2.75
**Group 2** Standard dose adaptive tumour focused **(SART)**	PTV2	64	32	2	55	20	2.75
	PTV (PTV – PTV2)	52	32	1.625	46	20	2.3
**Group 3** Dose escalated adaptive tumour boost RT (DART)	PTV2	70	32	2.1875	60	20	3
	PTV (PTV – PTV2)	52	32	1.625	46	20	2.3

4.3 Target volume dose objectives

Three dimensional dose distributions should be produced. The dose distribution should be assessed for coverage of the PTV and normal tissues using appropriate transverse sagittal and coronal views. The following optimal and mandatory target volume dose constraints are proposed:

Table 5: Target dose objectives

Volume	Dose Constraints	Optimal	Mandatory
PTV2	$D_{98\%}$	≥95% of prescribed dose	≥90% of prescribed dose
	*$D_{50\%}$	-	+/- 1% of prescribed dose
	$D_{2\%}$	≤105% of prescribed dose	≤107% of prescribed dose
PTV (PTV – PTV2)	D98%	≥95% of prescribed dose	≥90% of prescribed dose

For peer review only - http://bmjopen.bmj.com/site/about/guidelines.xhtml

For peer review only

*Please note that D50% constraint refers only to PTV2. PTV D50% is likely to be exceeded depending on size of PTV2. Therefore no compromise to PTV2 coverage should be made at the expense of achieving D50% PTV constraint.

4.4 Normal tissue dose constraints

The dose to OAR should be minimised. The following dose volume constraints are a guide.

Ideally, for Groups 2 & 3 optimal constraints for other bowel should be met for Plan 1 (small) and mandatory constraints for other bowel should be met for Plan 2 (medium). Provided the Plan 3 (large) has been adequately optimised it is recognised that some of the rectum and other bowel mandatory constraints may not be achieved.

Likewise for group 1 patients, provided the plan has been suitably optimised, some rectum and other bowel dose tolerances may be exceeded due to inclusion within the PTV. It is at the local Principal Investigator's discretion to accept the OAR doses and should be noted on the plan assessment form. The DVH assessment for each plan should be with the overall prescribed dose.

For patients in group 3, if mandatory dose constraints are not met on the medium plan advice must be sought from the RAIDER QA team.

For this reason it is recommended for adaptive planning the medium plan is produced first, this should then be copied and the relevant objectives and prescription volume are changed for the small and large plans. **Dose to the PTV2 or PTV must not be compromised in order to meet the normal tissue constraints.**

Table 6a: OAR Dose Constraints for 32-fraction schedule

**Rectum**	**V30Gy**	**80%**	
	**V50Gy**	**60%**	
	**V60Gy**	**50%**	
	**V65Gy**	**30%**	
	**V70Gy**	**15%**	
**Femoral Heads**	**V50Gy**	**50%**	
**Other Bowel**		**Optimal**	**Mandatory**
	**V45Gy**	**116cc**	**139cc**
	**V50Gy**	**104cc**	**127cc**
	**V55Gy**	**91cc**	**115cc**
	**V60Gy**	**73cc**	**98cc**
	**V65Gy**	**23cc**	**40cc**
	**V70Gy**	**0cc**	**10cc**
	**V74Gy**	**0cc**	**0cc**
****Whole bladder constraint (i.e. CTV)**		**Optimal**	**Mandatory**
	**V60Gy**	**50%**	**80%**
	**V65Gy**	**40% only in Group 3 (DART)<br>Otherwise 0% in Group 2 (SART)**	**50% only in Group 3 (DART)<br>Otherwise 5% in Group 2 (SART)**

For peer review only - http://bmjopen.bmj.com/site/about/guidelines.xhtml

RAIDER radiotherapy planning and delivery guidelines

**Body-PTV (Normal Tissue)**	**D_{1cc}**	**≤105% of prescribed dose**	**≤110% of prescribed dose**

******<u>Whole bladder (CTV) constraint specified above (Table 6a) should be used to **inform plan optimisation**.</u> Please contact the RAIDER QA team if you fail to meet this constraint.

Bladder outside PTV2 (i.e. CTV-PTV2) meeting V60Gy and V65Gy absolute constraint of 80% and 50% respectively will be collected for reporting of primary endpoint.

Table 6b: OAR Dose constraints for 20-fraction schedule

<table>
<tr><td rowspan="5">Rectum</td><td>V25Gy</td><td colspan="2">80%</td></tr>
<tr><td>V41.7Gy</td><td colspan="2">60%</td></tr>
<tr><td>V50Gy</td><td colspan="2">50%</td></tr>
<tr><td>V54.2Gy</td><td colspan="2">30%</td></tr>
<tr><td>V58.3Gy</td><td colspan="2">15%</td></tr>
<tr><td>Femoral Heads</td><td>V41.7Gy</td><td colspan="2">50%</td></tr>
<tr><td rowspan="8">Other Bowel</td><td></td><td>Optimal</td><td>Mandatory</td></tr>
<tr><td>V37.5Gy</td><td>116cc</td><td>139cc</td></tr>
<tr><td>V41.7Gy</td><td>104cc</td><td>127cc</td></tr>
<tr><td>V45.8Gy</td><td>91cc</td><td>115cc</td></tr>
<tr><td>V50Gy</td><td>73cc</td><td>98cc</td></tr>
<tr><td>V54.2Gy</td><td>23cc</td><td>40cc</td></tr>
<tr><td>V58.3Gy</td><td>0cc</td><td>10cc</td></tr>
<tr><td>V61.7Gy</td><td>0cc</td><td>0cc</td></tr>
<tr><td rowspan="3">***Whole bladder constraint (i.e. CTV)</td><td></td><td>Optimal</td><td>Mandatory</td></tr>
<tr><td>V50Gy</td><td>50%</td><td>80%</td></tr>
<tr><td>V54.2Gy</td><td>40% only in Group 3 (DART)
Otherwise 0% in Group 2 (SART)</td><td>50% only in Group 3 (DART)
otherwise 5% in Group 2 (SART)</td></tr>
<tr><td>Body-PTV (Normal Tissue)</td><td>D_{1cc}</td><td>≤105% of prescribed dose</td><td>≤110% of prescribed dose</td></tr>
</table>

*******<u>Whole bladder (CTV) constraint specified above (Table 6b) should be used to **inform plan optimisation**.</u> Please contact the RAIDER QA team if you fail to meet this constraint.

Bladder outside PTV2 (i.e. CTV-PTV2) meeting V50Gy and V54.2Gy absolute constraint of 80% and 50% respectively will be collected for reporting of primary endpoint.

Normal tissue dose constraints and dose volume histograms (DVH) for 20 fraction schedule are presented in Table 6b (recalculated from above using linear quadratic equation, assuming that all α/β of organs at risk is 3 and that dose to tumour boost is delivered in 3Gy per fraction).

For peer review only - http://bmjopen.bmj.com/site/about/guidelines.xhtml

4.5 Planning recommendations

- Produce the medium plan first.

- For the planning priorities should be as follows, in order of importance:
  1. ***Achieve 95% coverage of PTV2***
  2. ***Achieve 95% coverage of the PTV***
  3. Achieve mandatory OAR dose constraints
  4. Achieve optimal OAR dose constraints
  5. Reduce areas of high dose within PTV-PTV2 especially away from boost site

Therefore, for the majority of cases dose coverage of the PTV_2 and PTV volumes should not be compromised to reduce dose to OAR without first contacting RTTQA for advice.

- For Group 2 & 3, review all three plans together after planning using the plan comparison function. The dose to the RECTUM and OTHER_BOWEL should stay the same or get progressively higher as the PTV size gets larger i.e. the dose statistics should not be better for the large plan than the small or medium plan.
- If throughout the course of treatment any patient requires re-planning please contact the relevant QA group.
- If the PTV volume is outside the patient's body please contact the relevant QA group.

4.6 Pre-Treatment checks

To minimise risk of error at the time of importing, exporting and plan selection, please ensure that each beam name and ID reflects the assigned plan i.e. Sm_Plan.

At the time of plan exporting, it is recommended to find a way of ensuring that centres' local record and verify systems cannot mix beams from different plans. For example create each plan with slightly different contributions from each field so that only the correct combination of beams can be chosen on any given day (Applicable only to Mosaiq). This could be done by adding 2 points diagonally on the isocentre slice with a dose close to the 100% isodose. Then all beams can be assigned from a plan to each of the points as the reference point.

Please ensure that the safe scheduling of multiple treatment fields and recording the dose delivered is considered for RAIDER patients. Additionally, please ensure that the safe scheduling of imaging only fields is considered. These procedures will be captured in the process document. If you have any questions please contact the relevant QA group.

For peer review only - http://bmjopen.bmj.com/site/about/guidelines.xhtml

5 TREATMENT DELIVERY

It is important to ensure that patients follow the bladder preparation instruction for their group as they have done at the planning CT scan appointment (i.e. empty for group 1 and partially full for group 2 and 3).

5.1 Standard whole bladder RT (group 1)

Acquire pre-treatment cone beam CT images using a full pelvis scan. Image match and register bone, according to the recommendations stated in the NRIG IGRT report.

Make required corrections/shifts as per departmental practice.

The acquired cone beam CT image can be used for assessment of target coverage at the individual department's discretion. Such use will be documented. Any changes made on the basis of the scan should be reported in the CRF and to RTTQA (including exposures not resulting in treatment because of patient factors).

Schedule imaging for Group 1 patients, as per departmental protocol. In addition to this please ensure a post-treatment CBCT is taken in the first week and weekly for these patients.

5.2 Adaptive Tumour Focused RT (SART and DART)

Acquire pre-treatment cone beam CT image and register bone according to the recommendations stated in the NRIG IGRT report. Make any corrections according to departmental practice.

An appropriately RTTQA trained and accredited healthcare professional should review the bone matched CBCT, to assess bladder filling and PTV coverage. The following steps should assist centres when assessing the CBCTs.

The aim in treating adaptive patients is to use the **smallest plan possible**, so that the dose is minimised to the OARs without compromising both PTV and PTV2 coverage.

The steps below describe how this can be best achieved at plan selection. It is important to review overall bladder filling, and PTV2 boost position as determined on the planning CT scans before plan selection.

5.2.1 Image Match and Plan Selection Steps:

1. Following CBCT, the bladder filling and size should be checked against CTV_30 contour.
2. If the bladder is of similar shape and size to the CTV at planning (i.e. CTV_30), then the small plan should be preferably considered in the first instance for treatment.
3. Once the bladder filling and shape has been assessed begin to review the appropriate PTVs. An appropriate plan provides suitable coverage of the PTV2 and PTV, with minimal normal tissue irradiation.
4. Manual (soft tissue) moves should be made to ensure the bladder is adequately covered whilst selecting the smallest plan possible to spare normal tissue. Please be mindful of the magnitude of the soft-tissue shift, moves greater than 1cm can impact on the accuracy of the expected dosimetry. Discuss with planning department if this shifts over 1cm occur and contact RTTQA retrospectively.

For peer review only - http://bmjopen.bmj.com/site/about/guidelines.xhtml

5. Additional manual moves can be undertaken if felt it could further optimise PTV2 coverage. Manual moves prioritising coverage to the boost region over the normal bladder wall is permitted if it avoids excessive normal tissue irradiation that would have occurred by selecting a larger plan. Again, please be mindful of the magnitude of the soft-tissue shift, moves greater than 1cm can impact on the accuracy of the expected dosimetry. Discuss with planning department if this shifts over 1cm occur and contact RTTQA retrospectively.
6. Assess the OAR. Review if they are similar to planning scan. Additionally it is important to check where they are relative to PTV2 i.e. ensuring not in PTV2, especially for DART patients.

The actions taken for each patient must be confirmed by a second appropriately trained and accredited healthcare professional. Once agreement has been reached any correction should be performed and the plan selection agreed and confirmed for treatment.

5.2.2 Image Match and Plan Selection Tips:

This guidance is provided to assist RAIDER trained staff with potential solutions for scenarios that may arise on treatment. Further guidance is provided in APPENDIX E. Fractions must not be omitted or missed due to unfavourable positioning of normal anatomy such as rectal distention due to flatus or faeces.

- Please only choose a large plan if the bladder has filled. (E.g. *If the bladder has filled more than the CTV_30, and the patient has a CTV_60, overlay the CTV_60 to assess the magnitude of the filling).*
- Please consider the nature of bladder filling. We would expect filling to occur in the anterior and superior direction.
- Please be mindful of minimising image match and plan selection time.
- If the bladder is significantly smaller than the CTV 30 contour at planning, it is likely that the PTV2 boost is in the incorrect position and, or does not achieve adequate normal bladder sparing. In these circumstances patient should not proceed but be removed from the couch, and encouraged to fill the bladder by drinking further, and/or increasing the time interval of image acquisition. The local PI and RTTQA/TROG should be informed.
- In the event that the bladder has over filled and none of the library PTVs cover the entire bladder despite manual move the patient should be asked to minimally void and the CBCT is repeated. If this is not possible, patient should void completely and restart drinking protocol but consider reducing the time interval for CBCT acquisition (see appendix C and D). In these circumstances a member of the clinical team should also be notified to ensure the patient is not in urinary retention. If the large plan is being regularly selected, during a single treatment week, please contact the RTTQA/TROG to discuss this patient.
- Caution should be taken when changing the drinking protocol. Please consider changing one aspect of the protocol at a time, i.e. change the timing only or change the amount of water only.
- If no contours are suitable because of rectal gas, then remove the patient from the bed and ask them to void. Repeat the above steps. If contours still not suitable select the most suitable plan, i.e. optimising coverage to PTV2 and minimising the inclusion of Oars. If this occurs repeatedly (e.g. more than twice in 5 fractions) please contact RTTQA for advice.
- All CBCT exposures including those not resulting in treatment should therefore be recorded on the CRF and plan selection form.
- The patient's hydration may be different on chemotherapy days. Care should be taken when adjusting the drinking protocol on these fractions.

For peer review only - http://bmjopen.bmj.com/site/about/guidelines.xhtml

- Please review the post-treatment CBCTs to ensure the patient's bladder has not filled beyond the plan selected. If this has occurred, then consider the length of time taken for plan selection and see section 5.3.

5.3 Post treatment

A post treatment cone beam CT should be taken in the first week and once a week thereafter (for all randomised groups, including group 1). It should be reviewed locally to ensure intra fraction filling has been accommodated in plan selection. Please discuss with the PI and contact RTTQA if there are any issues with intra-fraction filling.

5.4 On completion of radiotherapy

On completion of radiotherapy planning, all plans including CT images, structures, plan and dose matrix and plan assessment form (PAF), should be exported, anonymised and sent to the RTTQA or TROG team electronically following the exporting data guidelines in the QA appendix A&B. On completion of a patient's treatment, the plan selection form, first week and then weekly paired (pre and post radiotherapy) CBCT scans and the registration objects (Aria only) should be sent to the RTTQA or TROG team. Investigators should notify the QA team before deleting any relevant data.

Please ensure that all patient data is anonymised prior to sending it to RTTQA/TROG. If you have any difficulties anonymising the data, please contact RTTQA/TROG.

Sending data from Elekta/Mosaiq systems:

Week 1 and weekly pre and post treatment CBCT data should be exported from the XVI using "Option 3". This ensures the CBCT data is sent to the QA team in the correct treatment position.

Sending data from Varian/Aria systems:

Week 1 and weekly pre and post treatment CBCT data should be exported from Aria along with the online registration object. This ensures that the CBCT data can be reviewed in the treatment position by the QA team. Please refer to guidance in Appendix G& H.

6 TREATMENT SCHEDULING AND GAPS

Treatment interuptions during radiotherapy should be avoided as they have detrimental effect on outcome. Treatment must not be interrupted due to staffing issues. Advice should be sought from the RTTQA team and ICR-CTSU in real time if issues with patient scheduling and gaps arise.

If IGRT is unavailable due to unpredicted staff changes, machine breakdown and/or gap day treatment, patients in group 2 and 3 may be treated for up to 5 days using the PTV medium plan without plan selection. Pre-treatment CBCT where possible should still be acquired.. These pre-treatment CBCTs should be sent to RTTQA for review in addition to the CBCTs which are routinely collected.

For peer review only - http://bmjopen.bmj.com/site/about/guidelines.xhtml

RAIDER radiotherapy planning and delivery guidelines

In the event of machine service, breakdown, or bank holiday, compensation for the missed fraction should be made. In the first instance the local PI should advise on how this should be made, but it is expected to be achieved by either treating at a weekend or undertaking two fractions a day (ideally on a Friday with a minimum 6 hour gap between treatments). If the treatment machine is unavailable for more than 3 days, please contact the ICR-CTSU and QA team.

Should a treatment break occur due to toxicity, sites are advised to contact ICR-CTSU and/or RTTQA in real time. Compensation is not expected in these circumstance.

All missed fractions are to be reported to the ICR-CTSU and QA team.

For peer review only

For peer review only - http://bmjopen.bmj.com/site/about/guidelines.xhtml

APPENDIX A:

United Kingdom RAIDER QA Programme

Contact Details:

Contact for scientific queries	Dr Shaista Hafeez<br>Tel: Tel: 0208 661 3274<br>Email: Shaista.Hafeez@icr.ac.uk
Radiotherapy Trials Quality Assurance Team	Amanda Webster RTTQA Group<br>Tel: 0203 826 2320<br>Email:<br>raiderqa.enh-tr@nhs.net

The UK quality assurance programme for RAIDER comprises the following exercises detailed below:

Pre-Trial Quality Assurance

1. Facility questionnaire: This is designed to gauge the IGRT experience of a centre to date. It collects information regarding the type of IGRT used, action thresholds, frequency of interventions and imaging doses. This survey should be accessed online via: http://www.rttrialsqa.org.uk/rttqa/

2. Process document: Details are collected on all aspects of tasks for the complete patient pathway and includes details on all imaging procedures. A process document template can be found at http://www.rttrialsqa.org.uk/rttqa/

3. Outlining Benchmark cases: One case with lipiodol and one case without lipiodol will be provided by the RTTQA to be completed by the P.I for each recruiting site. The targets and organs at risk are to be named and delineated as per the RAIDER radiotherapy planning and delivery guidelines. Those sites that have successfully completed the outlining for another related NIHR trial will not be required to outline the organs at risk, only the target volumes. Please contact the QA team to confirm.

4. Planning Benchmark case: One planning benchmark case provided by RTTQA is to be planned according to the Group 3 randomisation arm.

5. In house IGRT training programme: It is a requirement of RAIDER that sites have an established IGRT training programme already in place before joining the trial. Sites should be utilising cone beam CT for treatment of bladder patients.

6. Bladder 'Plan of the Day' training: Two practice cases with 6 CBCTS each are provided for centres to work through with an accompanying training document. Case 1 includes answers with some step-by-step instructions, for case 2 the answers only are

For peer review only - http://bmjopen.bmj.com/site/about/guidelines.xhtml

provided. The cases can be provided on an Elekta or Varian database depending on which system is used by a site. Please contact RTTQA for the training guidance document. Those that have successfully completed the 'Plan of the Day' training for HYBRID do not need to complete this again.

7. RAIDER 'Plan of the Day' training video will be available for streaming on the RAIDER ICR website. Please contact RTTQA or ICR for access

8. RAIDER Plan of the Day assessment: Centres will be given details of two patients with 6 CBCTs each (12 match decisions) to allow individuals to make plan of the day decisions/choices. The match results will be exported to the RTTQA group for review. Those staff members that have completed the 'Plan of the Day' assessment for Hybrid will not be required to complete the assessment. All QA approved individuals will receive a confirmation of their RAIDER accreditation to undertake plan of the day assessments for RAIDER patients.

9. Verification of electronic data transfer: Check DICOM or RTOG data can be suitably anonymised and transferred to and from centres. This includes the planning data and on treatment data, i.e. CBCT and registration objects.

On-Trial Quality Assurance

1. Prospective plan review: The outlining and planning for at least the first adaptive patient and the first dose escalated patient (if the first patient is not dose escalated) will be subject to prospective review by the RTTQA group.

2. Ongoing data collection: All planning data (CT,RS, RD, RP files, PAF) and treatment delivery data (weekly pre and post treatment CBCT and registration objects (if available) will be collected by the RTTQA group for retrospective review

3. IGRT/POD selection support: The first adaptive patient randomised by all sites will be subject to a retrospective review. If centres have any difficulties in the plan selection, please do not hesitate to contact RTTQA.

Data Export

Please contact raiderqa.enh-tr@nhs.net for information on data transfer.

Please ensure that data is appropriately labelled when sent to RTTQA, i.e. trial name, randomisation number and type of data (e.g. planning/CBCTs).

For peer review only - http://bmjopen.bmj.com/site/about/guidelines.xhtml

For all patients please ensure the following data is sent for all RAIDER patients:

Table 7:

**RAIDER Data Checklist**	**Sent**
Diagnostic Information (e.g. report, screenshot of imaging)	
ALL planning CTs (e.g. include CT_30 and CT_60 for adaptive patients)	
Structure set, Dose Cube, Plan	
Plan Assessment Form	
Plan Selection Form (For all trial patients)	
Week 1 (#1-5) and weekly pre and post CBCTs	

For peer review only - http://bmjopen.bmj.com/site/about/guidelines.xhtml

APPENDIX B:

Trans Tasman Radiation Oncology Group: Australia & NZ

RAIDER Trial QA programme

Contact Details:

Primary ANZ sponsor contact details	Trans Tasman Radiation Oncology Group
Contact for public queries	Patrick Wheeler<br>+61 2 4014 3903<br>RAIDER@trog.com.au
Contact for scientific queries	Prof Farshad Foroudi<br>+61 3 9496 9797<br>farshad.foroudi@austin.org.au
Radiotherapy Quality Assurance Team	TROG<br>Email: qa@trog.com.au

Pre-Trial QA

1. Facility questionnaire: This is designed to gauge the IGRT experience of a centre to date. It collects information regarding the type of IGRT used, action thresholds, frequency of interventions and imaging doses. For simpler completion at centres, TROG have created two sections, one for Radiation Therapists and another section for Radiation Oncology Medical Physicists, which may be completed separately. This questionnaire can be accessed online at: www.trog.com.au. In addition, sites wishing to use inversely planned techniques must be credentialed to do so. Please contact qa@trog.com.au for further information.

2. Process document: Details are collected on all aspects of tasks for the complete patient pathway and includes details on all imaging procedures. A process document template can be found at http://www.trog.com.au/Trials-and-research-projects, RAIDER, Quality Assurance. TROG website password required.

3. Fiducial Marker Quality Assurance Document: To be completed by sites who have indicated that they intend to use fiducial markers. This document can be found at http://www.trog.com.au/Trials-and-research-projects, RAIDER, Quality Assurance. TROG website password required.

4. Outlining Benchmark cases: The P.I. from each recruiting site should complete the two outlining benchmarking cases available on the TROG website. One male (with lipiodol and one female (without lipiodol). The targets and organs at risk are to be named and delineated as per the RAIDER Radiotherapy Planning, Delivery and QA Guidelines.

   Male Case 1: Download TROG 14.02 BMKXX1 from http://www.trog.com.au/Trials-and-research-projects, RAIDER, Quality Assurance. TROG website password required.

For peer review only - http://bmjopen.bmj.com/site/about/guidelines.xhtml

Female Case 2: Download TROG 14.02 BMKXX2 from http://www.trog.com.au/Trials-and-research-projects, RAIDER, Quality Assurance. TROG website password required.

Once the outlining cases have been completed by the local P.I. an export of the CT images and structure sets in DICOM should be returned to the TROG QA team. All data must be transferred via Central Quality Management System (CQMS). CQMS can be accessed through https://cqms.trog.com.au/login.jsp. If you require a CQMS account, please contact qa@trog.com.au

5. Planning Benchmark case: One planning benchmark is to be planned according to the Group 3 randomisation arm of the trial.

   Download TROG 14.02 BMKXX3 from http://www.trog.com.au/Trials-and-research-projects, RAIDER, Quality Assurance. TROG website password required.

   Once the planning benchmark case (with three plans) has been created (with appropriate review and acceptance by the local P.I.) the export of the CT images, dose matrices, RT plan and structure sets in DICOM should be returned to the TROG QA team. All data must be transferred via the Central Quality Management System (CQMS). CQMS can be accessed through https://cqms.trog.com.au/login.jsp. If you require a CQMS account, please contact qa@trog.com.au.

6. In house IGRT training programme: It is a requirement of the RAIDER trial that sites have an established IGRT training programme already in place before joining the trial. Sites should be utilising cone beam CT for treatment of bladder patients. Successful participation in the TROG 10.01 BOLART trial including the e-learning will be regarded as satisfying these criteria. IGRT training programme details are requested as part of the Facility Questionnaire.

7. IGRT Credentialing:
   a. Bladder 'Plan of the Day' training: Two practice cases with six CBCTS each are provided for centres to work through with an accompanying training document. Case 1 includes answers with some step-by-step instructions, for case 2 the answers only are provided. The cases are provided in DICOM format via the TROG website. Sites must import the two cases with accompanying CBCTS into appropriate image matching/registration software. The training guidance document is accessible via the TROG website.

   b. Bladder 'Plan of the Day' training video: Available on the TROG website: www.trog.com.au

   c. RAIDER Plan of the Day assessment: Two patients with six CBCTs each (twelve match decisions) will be provided to sites to allow individuals to make plan of the day decisions/choices. Staff must record their match results using the Plan of the Day Assessment Form. Assessment forms must be submitted to qa@trog.com.au.

8. Dosimetry Audit: All sites who have participated in the TROG 10.01 BOLART trial will not be required to complete a dosimetry phantom study. For sites who have not participated

For peer review only - http://bmjopen.bmj.com/site/about/guidelines.xhtml

in TROG 10.01 BOLART please contact the TROG QA office at qa@trog.com for further information.

On-Trial QA

Please contact TROG QA for advice as required for the first patient planning and treatment. Teleconference facilities may be made available if required.

1. Quality assurance Pre-treatment Plan Review: Pre-treatment review of at least the **first** adaptive patient and the **first** dose escalated patient (if the first patient is not dose escalated) recruited from each site will be conducted by TROG. The **first** participant with fiducial marker placement from each must also be submitted for review.

   The treatment plan will be required for review at least one week prior to treatment commencement. If the review results are acceptable the participant will proceed to treatment.

   The following **four** adaptive patients from each site will undergo timely review (to be completed during the first week of treatment).

   A checklist of the source data required for each RT QA case will be provided by the TROG QA Office with specifications of timelines for data submission included. See below for instructions for the upload of source data required for real time pre-treatment radiotherapy QA.

2. Quality assurance Post-treatment Plan Review: Adaptive patients will be sampled for QA review at a rate of **one in five** for post-treatment review following the initial pre-treatment/timely sampling.

   Patients in the standard whole bladder radiotherapy arm will be sampled for QA review at a rate of **one in five** for post-treatment review.

   All participants will be required to submit data at the end of treatment. This data should be submitted via CQMS. To assist with this process a checklist of the source data required will be provided to you by TROG QA. Timelines for data submission will also be specified.

3. IGRT/POD selection support: The first adaptive patient randomised by all sites will be subject to a retrospective review. If centres have any difficulties in the plan selection, please do not hesitate to contact the TROG QA team.

Data Export and Upload

Australia and New Zealand sites uploading data to TROG CQMS are not required to anonymise data prior to upload. All DICOM files are automatically de-identified at the point of upload to CQMS.

Radiation Therapy Treatment Plan
An electronic export of the radiotherapy treatment planning data file from the treatment planning system is required in **DICOM-RT format.** Please submit the RT Treatment Plan Export

For peer review only - http://bmjopen.bmj.com/site/about/guidelines.xhtml

[CT files, structure set, RT plan and dose matrix for each PTV] using the **[RT Plan Upload]** function listed in CQMS. Exported files must be uploaded as a single zipped file into CQMS.

- Please include ALL planning CTs (e.g. include CT_30 and CT_60 for adaptive patients)

CBCT Data Upload

The following files are also to be uploaded using the **[RT Plan Upload]** function listed in CQMS for final end of RT QA review:

- All pre and post treatment CBCT (exported in DICOM format). Each CBCT dataset should be uploaded as a single zipped file. As a minimum for all groups, this should include week 1 and weekly post-treatment CBCTs.
- Registration objects
- Please refer to Appendix G, H and I for additional assistance when exporting CBCT datasets from ARIA, MOSAIQ and Raystation

Supplementary Information Data Upload

The following files are to be uploaded using the **[Other Upload]** function listed in CQMS for final end of RT QA review:

- Treatment prescription (including total dose, number of fractions, dose per fraction, prescription isodose)
- Treatment plan summary (including field information and beam parameters)
- Plan of the day decisions (Plan Selection Form)
- Verification images demonstrating correct export of the radiotherapy plan (JPEG of the DVH and isodose distribution).
- Daily Dose Record (including dates of treatment delivery)
- Imaging Log (verification of imaging performed)

For peer review only

For peer review only - http://bmjopen.bmj.com/site/about/guidelines.xhtml

APPENDIX C:

QA Benchmark Cases: Outlining and Planning

PRE-TRIAL OUTLINING BENCHMARK CASE

All centres wishing to participate in the RAIDER trial will need to complete the following contouring exercise. DICOM CT data sets can be downloaded from the RTTQA website (www.rttrialsqa.org.uk) or TROG website (www.trog.com.au). See QA Appendix A& B for more information.

Outlining Benchmark Case 1: T2N0M0 Male Pelvis

History: 72 year old male, presented with haematuria. Proceeded with TURBT and 3 cycles neo-adjuvant chemotherapy (cisplatin-gemcitabine). Post treatment cystoscopy shows pathological complete response (pCR). Patient is planned for chemo-radiotherapy to bladder.

Biopsy: biopsy at diagnosis consistent with pT2a G3 TCC with no associated distant CIS

Initial staging diagnostic information: Information available for contouring/GTV delineation includes:

- bladder map/cystoscopy showing tumour present left lateral wall around left ureteric orifice
- CT baseline (pre-chemotherapy)
- MRI baseline (pre-chemotherapy) and lipiodol injected at tumour / scar (post chemotherapy)

Radiotherapy contouring/planning (according to group 2 and 3): Planning scan at 30 minutes and 60 minutes reflect no filling (<50cc)**.**

Contouring Instructions:

Please import Case 1 into your TPS.
Please outline the following volumes:

- GTV_30
- CTV_30
- CTV_60
- RECTUM
- OTHER_BOWEL
- FEMORALJOINT_L or FemJoint_L
- FEMORALJOINT_R or Femjoint_R

Please create the following PTV volumes using the expansion information contained in Table 1 and Table 2:

- PTV_Sm
- PTV_Med
- PTV_Lar_30
- PTV2_Sm
- PTV2_Med

For peer review only - http://bmjopen.bmj.com/site/about/guidelines.xhtml

- PTV2_Lar_30

NB: Please refer to the additional diagnostic information available for this patient.

Additional Diagnostic Information for Outlining Benchmark Case 1

Letter from surgeon identifying clinical position of tumour at cystoscopy (Bladder map not available):

Diagnoses:
1. **G3 pT2 at least TCC bladder.**
2. **Exercise induced asthma.**

I would be grateful if you could review this pleasant retired nurse urgently. I performed his TURBT on the 19th June and he had a large bladder tumour on the left lateral wall with a solid base. It was invading anteriorly into the prostate and bladder neck. Post-operative EUA revealed a mobile mass and a firm prostate. The histology has come as G3 pT2 tcc bladder.

He initially failed a trial without catheter and is having his catheter removed again today. He is otherwise fit and well.

Yours sincerely

Dictated and approved by Consultant

Post-chemotherapy Cystoscopy

Cystoscopy, bladder biopsy and lipiodol injection

Lithotomy, WHO checklist, pressure points protected, gentamicin.

Bimanual no pelvic mass, smooth prostate

Excellent views with 21 Fr scope and 12° lens. No visible tumour in bladder. Scar above left ureteric orifice - biopsied and lipiodol injected submucosally. New red patch at dome seen– region biopsied and lipiodol injected. Additional three random biopsies taken.

Histopathology:

Fibrosis only at all biopsied sites. Therefore red patch at bladder dome although injected with lipiodol should not to be included in GTV volume)

For peer review only - http://bmjopen.bmj.com/site/about/guidelines.xhtml

Outlining Benchmark Case 2: T2N0M0 Female Pelvis

History: 66 year old female presented to urologist with isolated episode of frank haematuria. Proceeded to TURBT. She started neo-adjuvant chemotherapy (cisplatin-gemcitabine) but suffered deterioration in hearing so went on to have chemo-radiotherapy to bladder after 1 cycle.

Biopsy: G3 pT2 TCC bladder with focal adjacent CIS

Initial staging diagnostic information: Information available for contouring/GTV delineation bladder map/cystoscopy tumour present left posterior wall ureteric orifice, CT baseline (pre-chemotherapy), MRI baseline (pre-chemotherapy, post TURBT) - non-contributory.

Radiotherapy contouring/planning (according to group 2 and 3): Planning scan at 30 min and 60 min reflect filling (>50cc)**.**

Contouring Instructions:

Please import Case 2 into your TPS.

Please outline the following volumes in accordance with instructions

- GTV_30
- GTV_60
- CTV_30
- CTV_60
- RECTUM
- OTHER_BOWEL
- FEMORALJOINT_L or FemJoint_L
- FEMORALJOINT_R or FemJoint_R

Please create the following PTV volumes using the expansion information contained in Table 1 and Table 2:

- PTV_Sm
- PTV_Med
- PTV_Lar_60
- PTV2_Sm
- PTV2_Med
- PTV2_Lar_60

Data Export

Once the outlining benchmark cases have been created, reviewed and accepted by the local PI, the export of the CT images and structure sets in DICOM should be returned to the appropriate QA team. See QA appendix A & B for information on data export.

For peer review only - http://bmjopen.bmj.com/site/about/guidelines.xhtml

For peer review only

PRE-TRIAL PLANNING BENCHMARK CASE

All trial centres must complete and submit the RAIDER pre-trial planning benchmark case. The CT DICOM data and pre-outlined structure set is available for download from www.rttrialsqa.org.uk (UK) or www.trog.com.au (Australia/NZ). Details for the planning cases are given in the next section.

Benchmark Planning Case: T2N0M0 TCC male pelvis

History: 63 year old male presented with frank haematuria, proceeded to TURBT; unsuitable for neo-adjuvant chemotherapy in view of co-morbidities, received chemo-radiotherapy to bladder

Biopsy: G3 pT2a TCC bladder

Initial staging diagnostic information: Information available for contouring/GTV delineation includes:

- Radiotherapy Bladder map/cystoscopy tumour present right bladder wall
- CT baseline,
- MRI baseline (post TURBT non-contributory).

Radiotherapy contouring/planning (according to group 3): Planning scan at 30 min and 60 min; filling (>50cc). PTV small/medium contours on 30minute scan; PTV Large created on 60minute scan

Planning Exercise:

Please import the CT and structure sets into your own TPS system. Following the RAIDER trial protocol and radiotherapy planning and delivery guidelines please produce a series of three radiotherapy plans as the patient is randomised to the dose escalated adaptive tumour boost DART (group 3)

Structures:

The CT has been delineated by a trial clinician with the following structures:

Structure	Volume (cc)
GTV_30	10.0
GTV_60	12.3
CTV_30	153.4
CTV_60	272.2
PTV_Sm_	273.2
PTV_Med_	385.3
PTV_Lar_60	577.0
PTV2_Sm	40.5
PTV2_Med	76.1
PTV2_Lar_60	82.3
RECTUM	65.4
OTHER_BOWEL	314.5
FEMORALJOINT_L	63.1

For peer review only - http://bmjopen.bmj.com/site/about/guidelines.xhtml

FEMORALJOINT_R	62.1

Please DO NOT edit any of these structures. The names and volumes on which the structures appear on have been given so that you can check that the structures have been imported properly.

Data Export

Once the planning benchmark case (three plans) has been created and reviewed and accepted by the local PI, the export of the Plan Assessment Form (PAF), CT images, dose matrices, RTplan and structure sets in DICOM should be returned to the RTTQA team (UK) or TROG team (Australia/NZ). For information on data export see QA appendix A & B.

For peer review only

For peer review only - http://bmjopen.bmj.com/site/about/guidelines.xhtml

APPENDIX D

Quick Contouring Checklist Adaptive Arms

1) Outline CTV_30 on CT30. Check whether appropriate for urethra to be included.
2) Copy and paste CTV_30 onto CT60. Rename CTV_60. Edit this volume to take into consideration bladder filling taking care not to make the volume smaller inferiorly.
3) Check CTV_30 does not expand beyond CTV_60. Use Boolean functions can be used to ensure CTV_60 always encompasses CTV_30.
4) Check volume difference between CTV_30 and CTV_60. If less than 50cc, expand all PTV volumes from target volumes on CT30, if greater than 50cc create large PTVs from CT60 target volumes.
5) Outline GTV_30 (and GTV_60 if applicable)
6) Ensure GTV shares the same bladder wall as CTV by either manual editing and/or use of Boolean operators.
7) Create adaptive PTVs from CTV using the relevant expansions
8) Create adaptive PTV2s from GTV using the relevant expansions
9) Check PTV_Lar encompasses PTV_Med and PTV_Sml
10) All OARs are always outlined on CT30 (FEMORALJOINT_L, FEMORALJOINT_R, RECTUM and OTHER_BOWEL. Check OTHER_BOWEL volume is at least 2.0cm superior to PTV _Lar

For peer review only

For peer review only - http://bmjopen.bmj.com/site/about/guidelines.xhtml

APPENDIX E

Proposed patient intervention to be considered in circumstances where plan selection is not felt to be optimal.

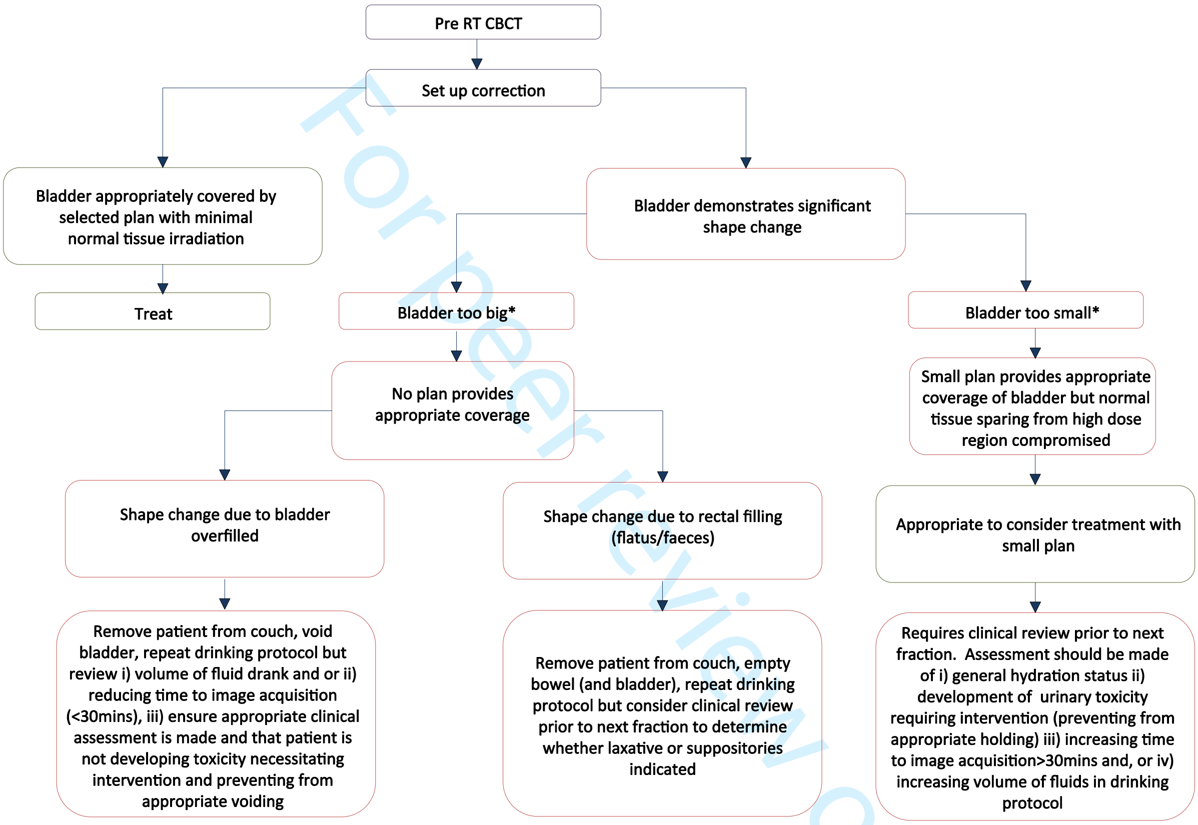

*If no plan provides appropriate coverage (bladder persistently too big or too small) more than twice in 5 fractions despite intervention, it is advised that the RTQA team is contacted for advice as in rare circumstances replan maybe indicated.

If the bladder is too small sites can consider treating with the small plan OR removing the patient from the treatment couch to allow for more filling.

**When considering changing the drinking protocol, only consider 1 timing or drinking change at a time. This will allow centres to establish which intervention has an effect on the patient's bladder.

For peer review only - http://bmjopen.bmj.com/site/about/guidelines.xhtml

APPENDIX F

RAIDER training cases and examples

1. Acceptable for treatment with chosen plan

Reference image

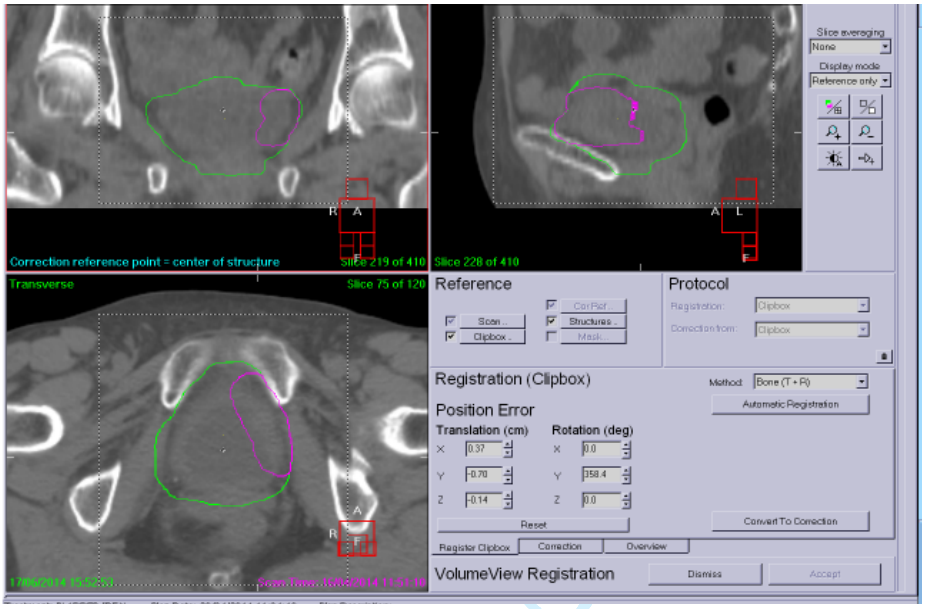

Localisation image

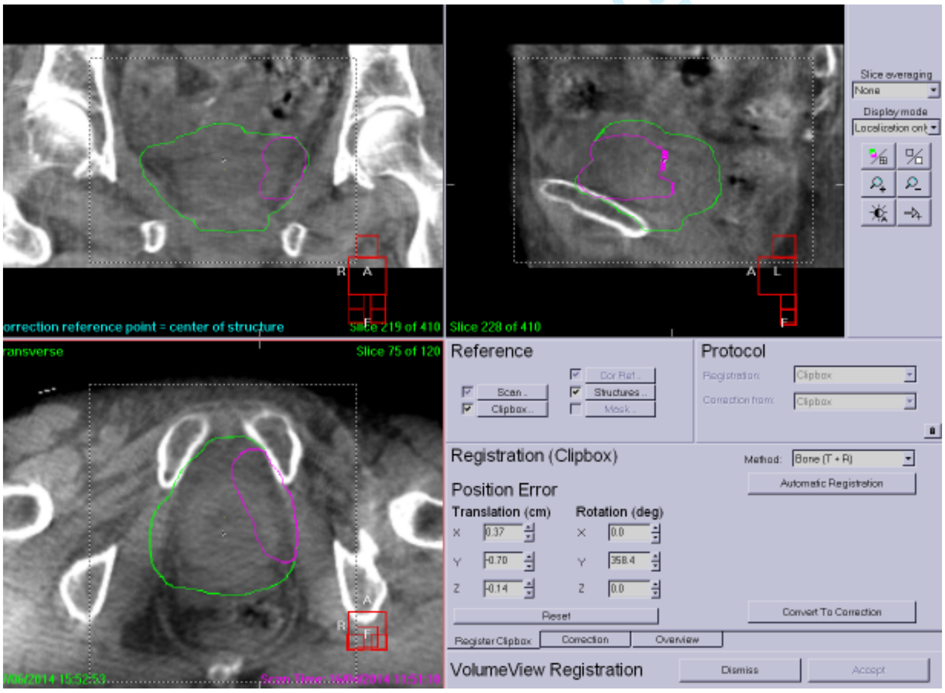

For peer review only - http://bmjopen.bmj.com/site/about/guidelines.xhtml

2. Bladder too small.

Bladder appears significantly under filled compared reference image, most apparent on sagittal localisation view. Note boost dose includes large proportion of bladder.

Acceptable for treatment with chosen plan as no compromise to bladder coverage. Patient review is recommended prior to next fraction to assess drinking protocol and, or time to image acquisition in order to optimise normal tissue sparing.

Reference image

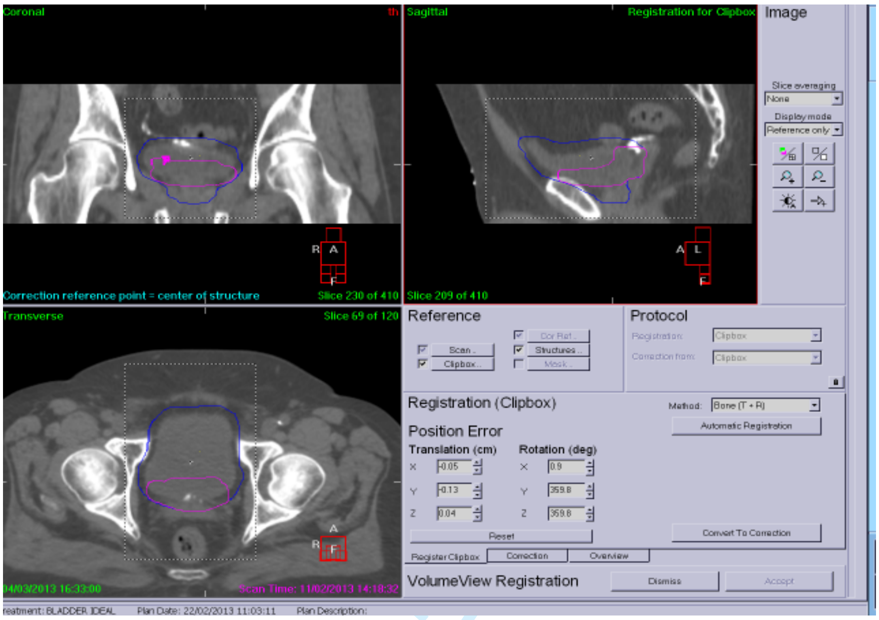

Localisation image

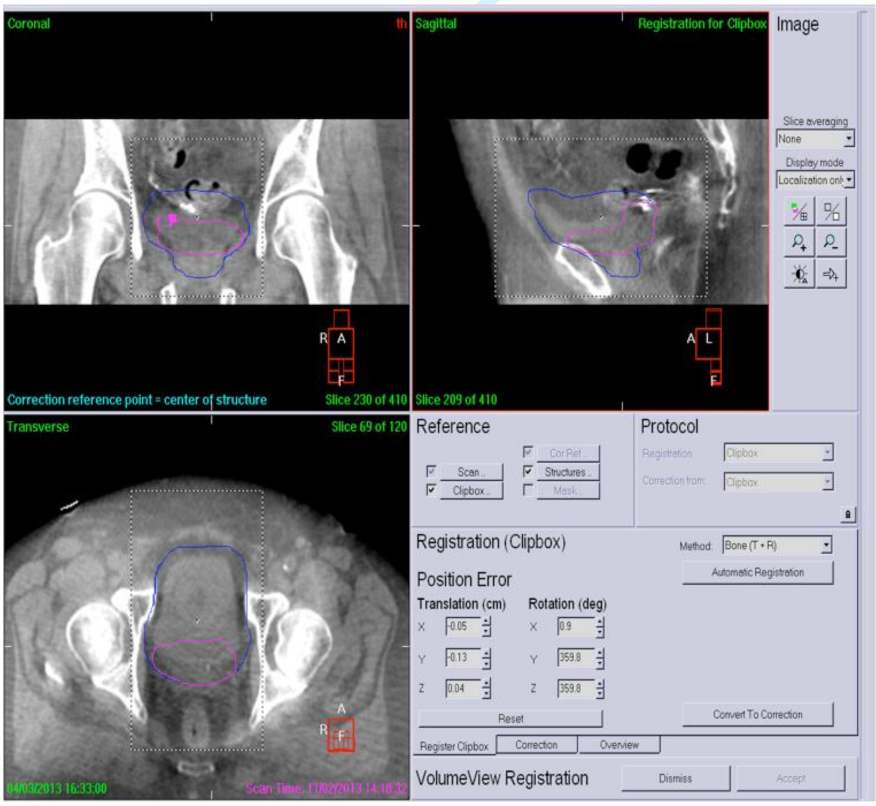

For peer review only - http://bmjopen.bmj.com/site/about/guidelines.xhtml

RAIDER radiotherapy planning and delivery guidelines

3. Bladder too large

No plan selection is acceptable for treatment (without compromise to bladder coverage).

Patient to empty bladder and repeat set-up with review of drinking protocol/time to CBCT acquisition.

Reference image

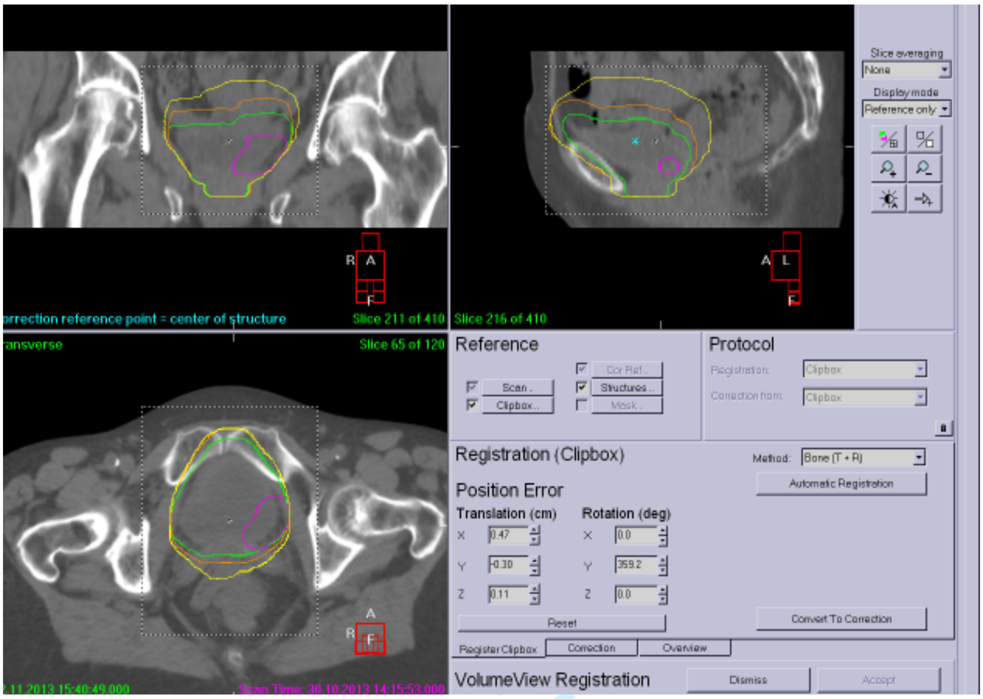

Localisation image with no structures

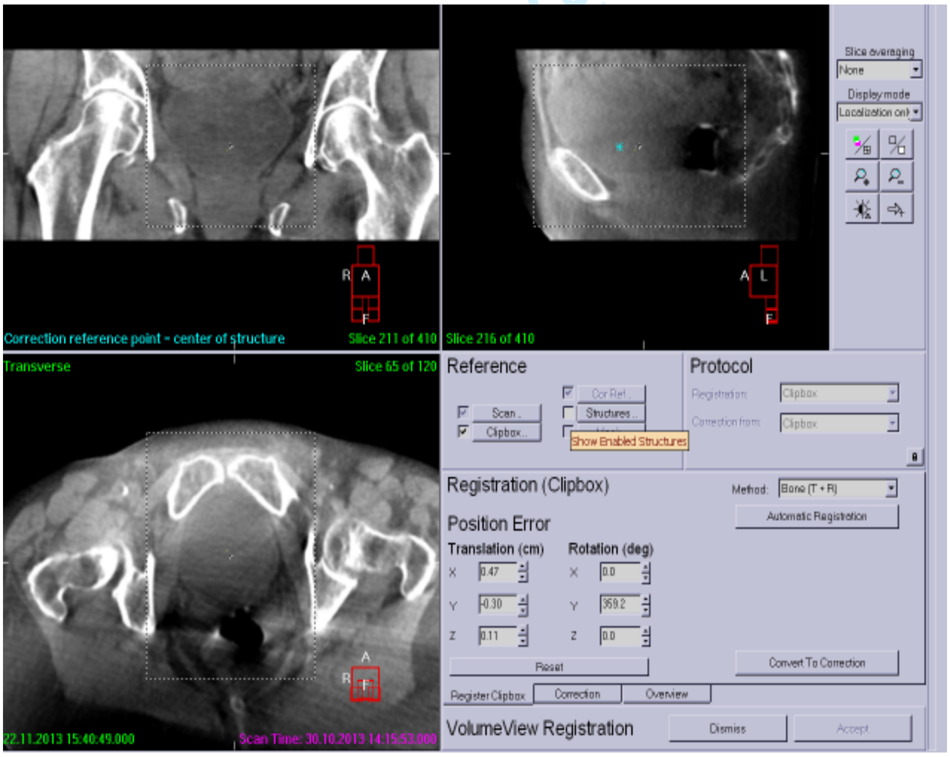

For peer review only - http://bmjopen.bmj.com/site/about/guidelines.xhtml

RAIDER radiotherapy planning and delivery guidelines

Reference image

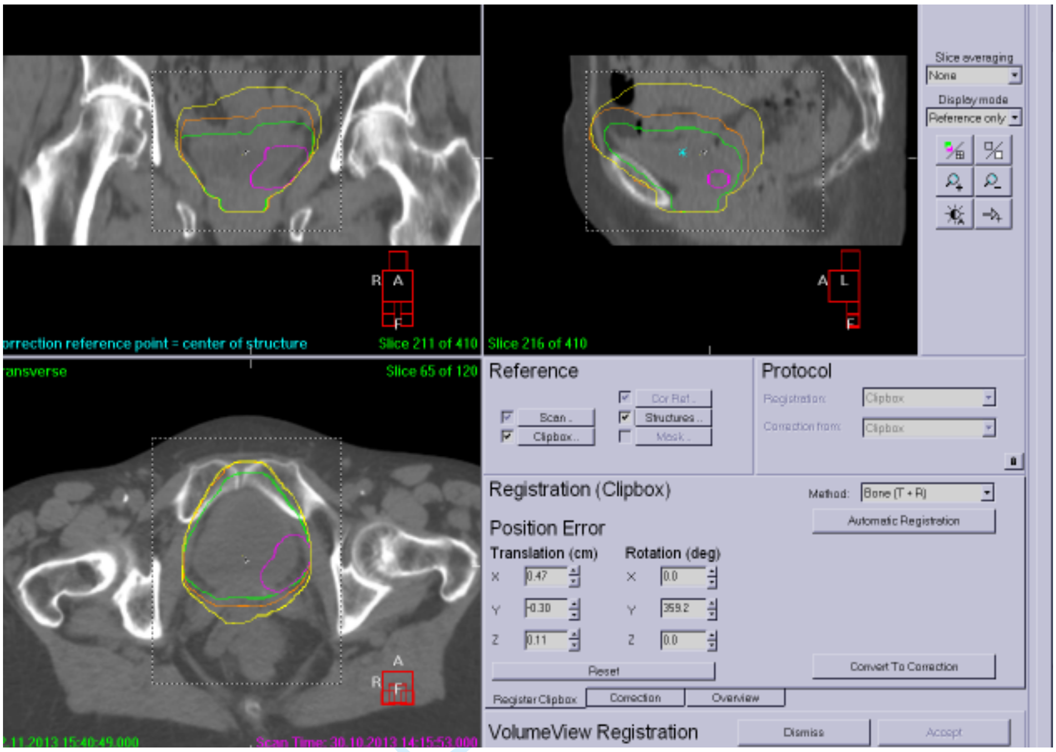

Localisation image with structures

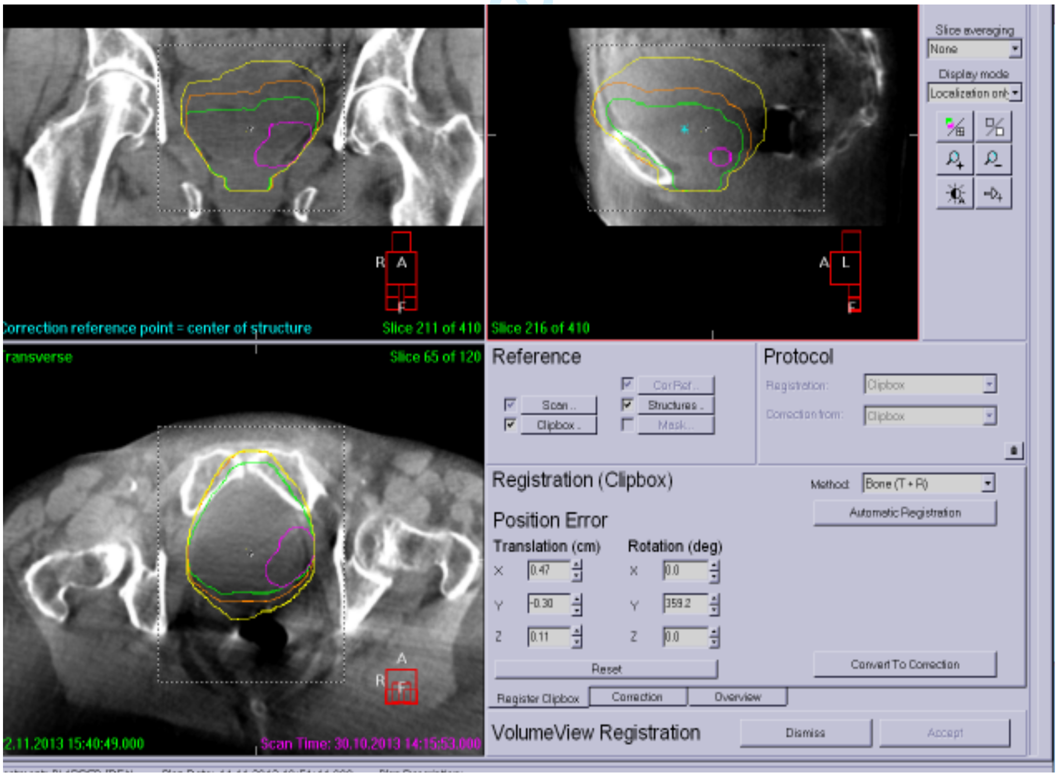

For peer review only - http://bmjopen.bmj.com/site/about/guidelines.xhtml

4. Fiducial marker artefact on CBCT interpretation

Image below demonstrates effect of lipiodol spill outside bladder resulting in significant artefact and degradation of image. Recommendation is that each CBCT should be closely reviewed with reference image and delineated bladder volume.

Reference image

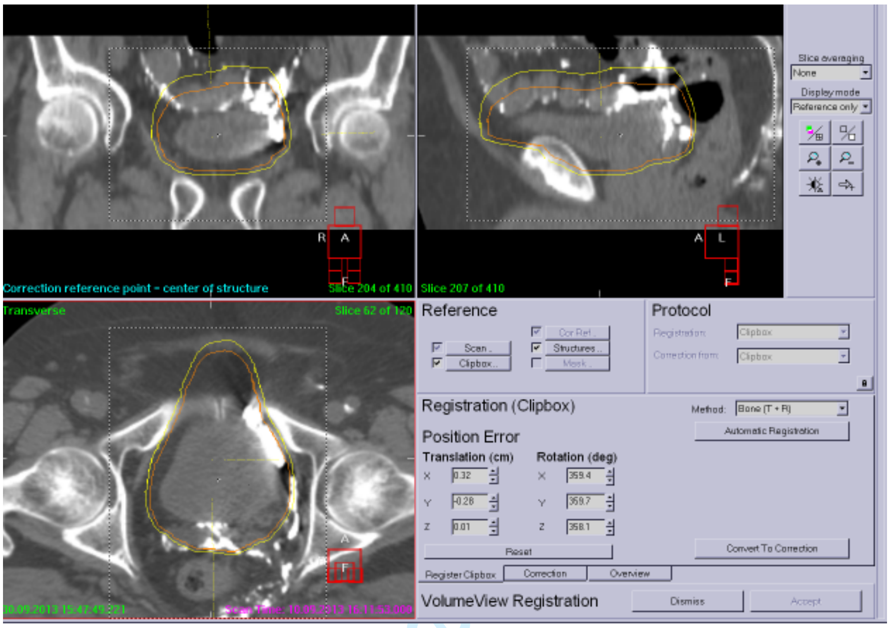

Localisation image

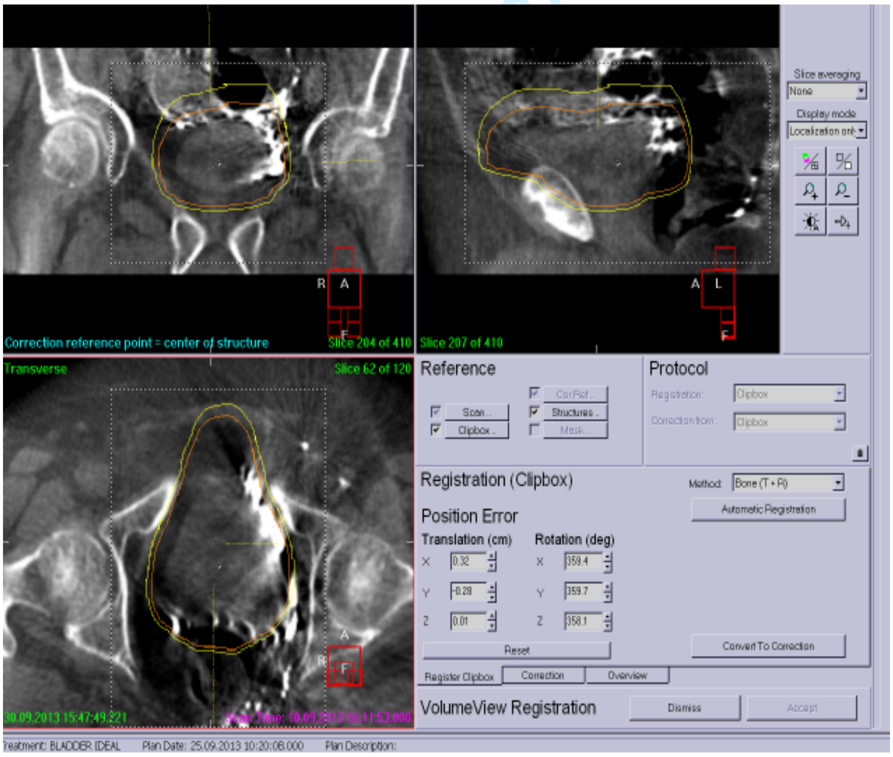

For peer review only - http://bmjopen.bmj.com/site/about/guidelines.xhtml

APPENDIX G

Quick Reference Guide Exporting CBCTs from Aria

These steps are for guidance only. Please contact your QA group if you have difficulties exporting data.

1) Load patient in Aria, go to Offline review and select one (any is ok) of the CBCTs to load
2) Select ‘Session Timeline’ tab at the bottom of the screen
3) Right click on the CBCT to export and select ‘Export to DICOM’ >> ‘To ‘DICOM Export to Pinnacle/External Anonymisation Software/Folder’’
4) The Import Export window loads. Select the ‘Show/Hide Tree’ button to bring up a list of all CBCTs and registration objects
5) Select the radio buttons next to the CBCTs to export, using the date to identify them.
6) Click on the + to expand the folder called ‘Registrations’:

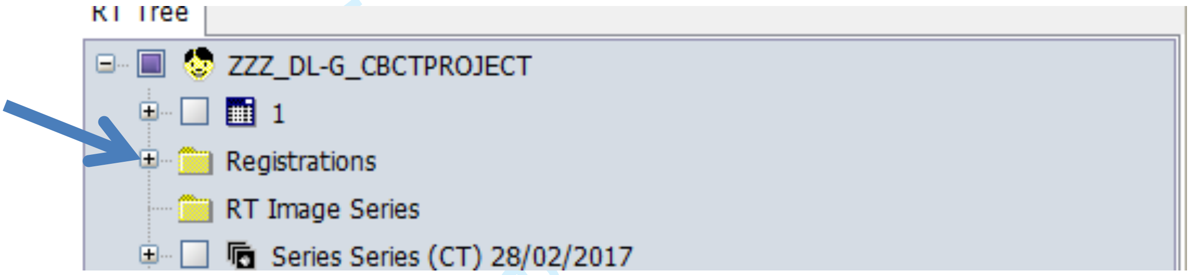

7) Select the radio buttons of the registration objects that have the same date as the CBCTs you wish to export.

8) Click the right arrow to export to the export folder/destination.

9) In windows explorer navigate to the export folder/destination and select the CBCTs and registration object files. Copy them to the DICOM anonymiser folder and run through your anonymiser to anonymise as per standard departmental working instructions. Please check the anonymization software does not remove the registration objects. If this occurs please contact RTTQA.

10) Save in a folder ready for export to RTTQA.

Please note that, for ease, whole patient exports can be submitted by the centre to RTTQA

For further information on suitable anonymisation software for ARIA exports please contact raiderqa.enh-tr@nhs.net

For peer review only - http://bmjopen.bmj.com/site/about/guidelines.xhtml

APPENDIX H

Quick Reference Guide Exporting CBCTs from Elekta/XVI

These steps are for guidance only. Please contact your QA group if you have difficulties exporting data.

Exporting from XVI

Images need to be exported individually, not as a treatment.

1. On the XVI acquisition PC select the image to be exported
2. Select IMAGE from the tool bar
    2.1. EXPORT
    2.2. DICOM SERVER 'select TPS/online server'
    2.3. OK
3. The next screen gives 3 options
    - Option 1 – In the Option 1 list, select a multiple of the voxel size in the reconstructed volume for the CT slice thickness. This can be done without a reference dataset being available and hence imports the CBCT into pinnacle without any co-ordinates related to the reference image. This is not likely to be useful.
    - Option 2 – Only available if image registration was done and approved for this reference image. The position of the VolumeView™ exported is the position **before** registration.
    - Option 3 – Only available if image registration was done and approved for this reference image. The position of the VolumeView™ exported is the position **after** registration.

3.1 Select Option 3 as the information required is as the patient was treated i.e. after registration (e.g. if patient was treated with correction).

NB Registration has to be performed for option 3 to be available.

3.2. In the Export options area, click the Create CT button.

3.3 EXPORT

For peer review only - http://bmjopen.bmj.com/site/about/guidelines.xhtml

APPENDIX I

Exporting CBCTs from Raystation

For centres exporting CBCTs from Raystation please contact raiderqa.enh-tr@nhs.net

For peer review only

This is a controlled document and should not be copied, distributed or reproduced without the written permission of the ICR-CTSU

For peer review only - http://bmjopen.bmj.com/site/about/guidelines.xhtml

Please print on hospital's headed paper

RAIDER

A **R**andomised phase II trial of **A**daptive **I**mage guided standard or **D**ose **E**scalated tumour boost **R**adiotherapy in the treatment of transitional cell carcinoma of the bladder

PATIENT INFORMATION SHEET

We would like to invite you to take part in a research study called RAIDER.

Before you decide whether to take part, it is important that you understand why the research is being done and what it would involve for you. One of your doctors or nurses will go through this information sheet with you and answer any questions you may have. Please take time to read the information carefully and to discuss it with relatives, friends and your GP if you wish. Please ask if anything is unclear or you need any further information.

Thank you for reading this and considering taking part in our research.

Why am I being invited to take part?
We are inviting you to join this study because your doctor has found cancer that has grown into the wall of your bladder (muscle invasive bladder cancer) and you are interested in receiving radiotherapy treatment which will be given in small doses every day for 7 weeks.

What is radiotherapy treatment?
Radiotherapy uses targeted beams of high strength x-rays to kill cancer cells. Because radiotherapy can also cause damage to non-cancer cells, the treatment is carefully planned by doctors and physicists so that only your bladder and a small border surrounding it is treated with the highest radiotherapy dose.

Radiotherapy is individually designed for each patient, based on a CT scan taken a few weeks before treatment. This CT scan tells your doctor about the position and shape of your bladder.

The bladder can move within the body depending on how full it is and because of where it is in relation to the bowel. It is important that the radiotherapy does not miss any of the bladder tumour because of this movement, so a safety margin is added around the bladder on the radiotherapy treatment plan.

For peer review only - http://bmjopen.bmj.com/site/about/guidelines.xhtml

Each patient would usually have one radiotherapy treatment plan designed for them so that the radiotherapy is targeted at their tumour.

When radiotherapy treatment is given, the patient lies still on a bed whilst the radiotherapy machine moves around to send the radiotherapy beams from different directions. These beams all focus on where the bladder is, to make sure the whole bladder receives the highest radiotherapy dose possible.

What is adaptive radiotherapy treatment?
We are now able to take a scan of where the bladder is when a patient is lying on the radiotherapy bed before each treatment. This means we can target the bladder more precisely.

In this study we are looking at whether it is possible to design three treatment plans (small, medium and large) and then choose the one that best fits the size of the bladder on the patient's treatment day. This is called 'adaptive radiotherapy'. Adaptive radiotherapy may allow treatment to be given with smaller safety margins, which may lead to fewer side effects.

What is tumour focused radiotherapy treatment?
It is also possible to focus the highest dose of radiotherapy on the bladder tumour. This means that the rest of the bladder can be given a lower dose of radiotherapy. This may mean fewer side effects as the rest of the bladder will be given less radiotherapy. It also allows us to find out whether a higher dose of radiotherapy can be given only to the tumour (tumour boost), to see if this will reduce the chance of the cancer returning, whilst also keeping any side effects as mild as possible.

What is the purpose of RAIDER?
RAIDER is based on a study of adaptive tumour boost radiotherapy which was conducted at one UK hospital. We hope to show that this complex radiotherapy can be given by radiotherapy departments at different hospitals. If this is possible, we want to look at whether the side effects are similar to those experienced by people receiving standard radiotherapy.

RAIDER is based on several smaller studies conducted at hospitals worldwide, which suggest that treatment using these radiotherapy techniques could help to reduce side effects. Although these smaller studies are promising, RAIDER is the largest study of these techniques and has been designed to give us as much information as possible. We will use the results of RAIDER to develop a future study to investigate whether these techniques could improve how well radiotherapy cures bladder cancer.

What would happen if I took part?
At least 240 people in the UK, Australia and New Zealand will be included in RAIDER. All RAIDER participants will be treated with daily radiotherapy.

For peer review only - http://bmjopen.bmj.com/site/about/guidelines.xhtml

Everyone who agrees to take part in this research study will be in one of three groups:

1. Group 1: Standard radiotherapy: One out of four people taking part will be given standard bladder radiotherapy using the same radiotherapy treatment plan each time, treating their whole bladder to the same dose of radiotherapy.

2. Group 2: Adaptive tumour focused radiotherapy: One out of four participants will be given adaptive tumour focused radiotherapy, using the radiotherapy treatment plan which fits the size of their bladder the best. The standard dose of radiotherapy will be targeted at the tumour, with the rest of the bladder receiving a lower dose than normal.

3. Group 3: Adaptive tumour boost radiotherapy: Two out of four participants will have adaptive tumour boost radiotherapy, using the radiotherapy treatment plan which fits the size of their bladder the best. A higher than standard dose of radiotherapy will be targeted at the tumour, with the rest of the bladder receiving a lower dose than normal.

The only way to make sure that the people in the three groups are as similar as possible is to have the treatment decided upon by chance: a process called randomisation. This process ensures that the treatments are compared fully and fairly.

If you agree to take part, your doctor or nurse will ring the research centre. The centre will then record your details and tell your specialist your treatment, which will be selected by chance. This means you could have any of the three treatments described above. Whichever group you are in, you will be treated with the best possible care and will be monitored closely.

<u>What do I have to do before my radiotherapy treatment?</u>
To make sure your treatment is as effective as possible, it has to be carefully planned by your doctor and other specialised staff (radiographers and physicists).

To help with this, your doctor may wish you to have small markers (fiducial markers) placed in your bladder. If this applies to you, you will be given the bladder tumour marker information leaflet to read.

You will need to visit the hospital for a planning CT scan before you start radiotherapy. The radiographers will also take measurements that are needed for the treatment plan and will make small permanent marks which help to line up the radiation beam. Radiotherapy is a very precise treatment and it is important that you are able to lie in exactly the same position for every treatment.

For peer review only - http://bmjopen.bmj.com/site/about/guidelines.xhtml

Group 1 – standard radiotherapy planning visit:
You will have a planning CT scan taken with your bladder empty. All of the planning procedures for the standard group are part of the routine care for patients receiving bladder radiotherapy, so you would have them even if you choose not to take part in the RAIDER research study. The planning session at the radiotherapy department usually takes place once and lasts about 30 minutes.

Group 2 and 3 – adaptive tumour focused radiotherapy planning visit:
If you are in group 2 or 3, we will ask you to empty your bladder and then to drink 350ml of water (just over ½ pint). We will take the first scan at 30 minutes and the next at 60 minutes after drinking, as your bladder fills. If you decide to join the study you will be given a leaflet describing drinking guidelines. The planning session lasts about 70 minutes in total, but each scan will only take a few minutes.

<u>What do I have to do during my radiotherapy treatment?</u>
Your treatment will be given daily. If you are in group one we will ask you to empty your bladder immediately before each treatment. If you are in group 2 or 3, we will ask you to empty your bladder and then drink 350mls of water 30 minutes before your planned radiotherapy treatment.

For all groups, once the radiographer has helped you to get into position and made sure that you are comfortable, we may take a scan in the treatment room. This will take about 2 minutes.

For patients in group one receiving standard radiotherapy, this scan will be used to make sure the bladder is in the area which will receive the highest dose of radiotherapy.

If you are receiving adaptive tumour focused radiotherapy we will use the information from the pre-treatment scan to choose the best radiotherapy treatment plan to fit your bladder size and the position of your tumour. This will take around five to ten minutes. The plan will be selected by a specially trained doctor or radiographer and checked by a second trained person before you receive your radiotherapy treatment.

You will need to lie still for up to 20 minutes whilst the machine moves around you to deliver the radiotherapy from different angles. You will not feel anything, as it is similar to having an x-ray.

During your radiotherapy treatment you will be seen by your doctor and/or nurse/radiographer every week to record and treat any side effects that you may be experiencing. They will also take a small sample of blood before treatment starts and again during the first, fourth and sixth week of radiotherapy.

For peer review only - http://bmjopen.bmj.com/site/about/guidelines.xhtml

Chemotherapy treatment during radiotherapy

Your doctor may suggest that you also have chemotherapy while you have your radiotherapy treatment. You will be able to take part in RAIDER whether or not you have chemotherapy. Your doctor will discuss the possibility of chemotherapy with you.

How often will I need to visit the hospital after my treatment?

Everyone in the RAIDER study will be asked to visit the hospital for check-ups on the same schedule after treatment,as described below:

- *10 weeks after the start of radiotherapy:* assessment of side effects
- *3 months after the end of radiotherapy:* cystoscopy (inspection of your bladder with a telescope) under general anaesthetic & biopsy of the site of the tumour; blood sample; chest x-ray; assessment of side effects
- *6 months:* cystoscopy under local anaesthetic; blood sample; CT scan of abdomen and pelvis; chest x-ray or CT scan; assessment of side effects
- *9 months:* cystoscopy under local anaesthetic; assessment of side effects
- *12 months:* cystoscopy under local anaesthetic; CT scan of abdomen and pelvis; chest x-ray or CT scan; assessment of side effects
- *18 months:* cystoscopy under local anaesthetic; chest x-ray or CT scan; assessment of side effects
- *24 months:* cystoscopy under local anaesthetic; CT scan of abdomen and pelvis; chest x-ray or CT scan; assessment of side effects
- *36, 48, 60 months:* cystoscopy under local anaesthetic; chest x-ray; assessment of side effects

A summary of these visits is on page 15 of the patient information sheet, for your reference should you decide to join the study.

These visits have been designed to be as similar as possible to what would happen if you decided not to join the RAIDER study. If your cancer is found to have returned during study check-ups your doctor will discuss further treatment options with you.

After your 5 year (60 month) visit, we would like to collect basic information about your health from any routine follow up visits you have, and also from national electronic databases which are kept on everyone’s health status.

For peer review only - http://bmjopen.bmj.com/site/about/guidelines.xhtml

RAIDER
Study Design

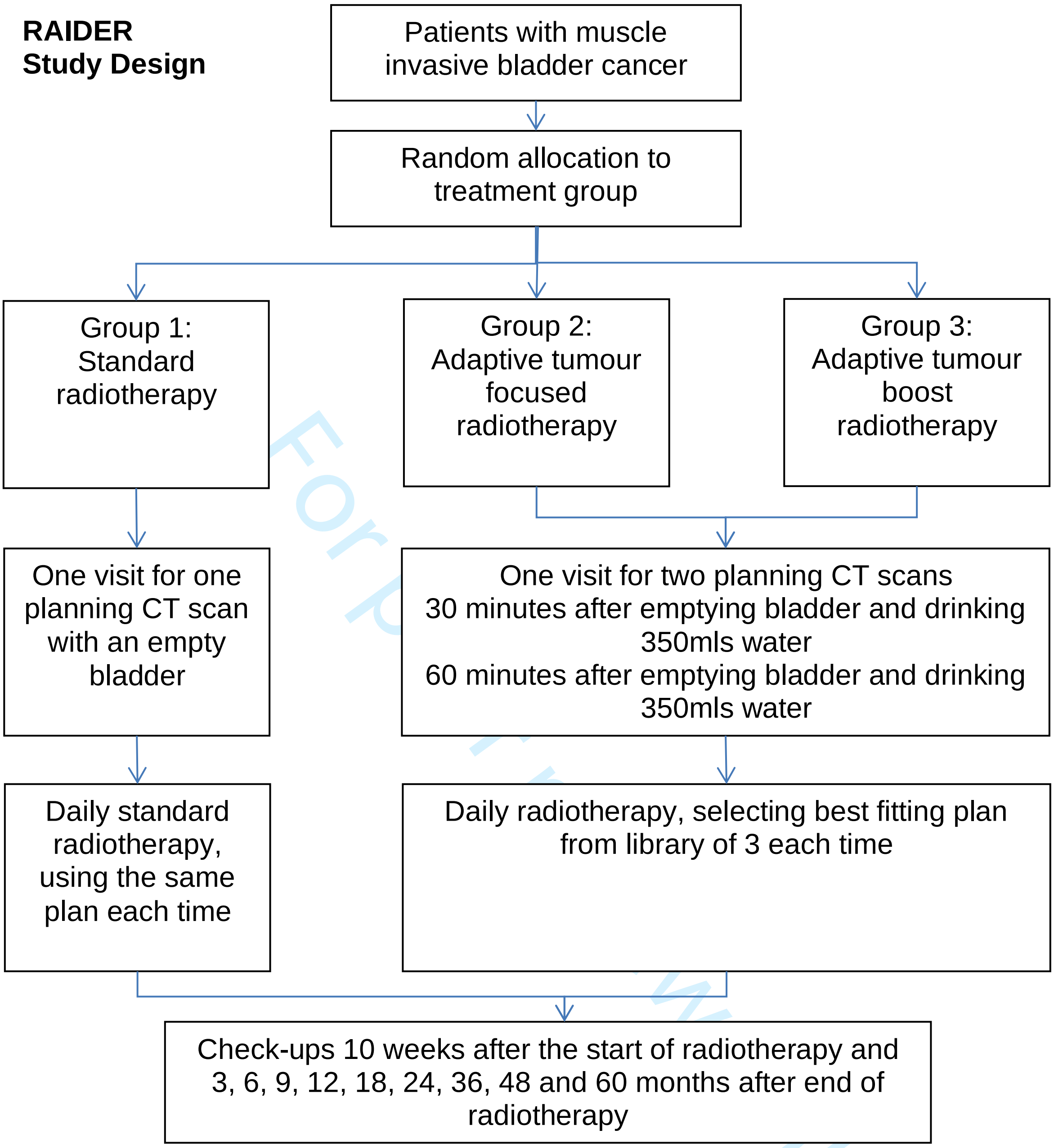

What are the possible side effects of treatment?

Patients who have radiotherapy can experience some side effects. These can occur in anyone receiving radiotherapy to the bladder whether or not they are in the RAIDER study. It is difficult to predict whether you will have some, all, or none of the side effects, or how severe they may be. They are usually mild and short lived but can sometimes be more serious.

Please let your doctor or nurse know about any side effects that you are concerned about so they can advise you what to do. Their telephone numbers are listed on page 12. There is also 24 hour support available from your hospital, to provide access to immediate medical care in the event of any serious problems.

For peer review only - http://bmjopen.bmj.com/site/about/guidelines.xhtml

Not all patients will experience all of these side effects and we can give you medications to treat any side effects you may experience.

You will be able to carry out most of your normal activities during radiotherapy, but you may feel more tired than normal and may need to rest more.

Side effects that can develop during radiotherapy may include:

- diarrhoea (around 3 in 10 people)
- needing to urinate more often (around 5 in 10 people)
- bleeding, pain or discomfort on passing urine (around 2 in 10 people)
- passing stools more frequently or with pain (around 1 in 10 people)

Some side effects can develop several months after radiotherapy ends. These include:

- a need to urinate more often or more urgently (around 2 in 10 people)
- bowel changes due to scarring or bleeding (around 5 in 100 people)
- vaginal scarring (around 3 in 10 women)
- problems with getting and maintaining erections (around 2 in 10 men)
- infertility (all women, around 5 in 10 men)

A few patients may develop long term effects. These are usually mild but may occasionally be serious and require treatment.

If you are in group 3 and receive a tumour focused boost as part of your radiotherapy treatment, you will be receiving a higher total dose of radiotherapy to the tumour than people in other groups. There is therefore a possible increased risk of radiotherapy side effects, but the radiotherapy techniques used in this trial aim to keep the risk of side effects similar to those experienced following the standard dose. Side effects in group 3 will be very closely monitored throughout the study and if they appear to be higher than expected, treatment within this group will be stopped, and everyone in group 3 will receive standard dose tumour focused radiotherapy.

Do I have to take part?

No, it is up to you to decide whether to take part or not. If you decide to take part, you will be given this information sheet to keep and will be asked to sign a consent form. You are free to change your mind and withdraw from the study at any time without giving a reason. If you do choose to withdraw, your doctor will discuss with you the best treatment options available.

What are the alternatives to this study?

If you decide not to participate in this study, it will not affect the usual standard of care you receive. Standard recommended treatments for muscle invasive bladder cancer are surgery to remove the bladder or daily radiotherapy using one treatment plan and the

For peer review only - http://bmjopen.bmj.com/site/about/guidelines.xhtml

same dose to treat the whole bladder. If you do not take part in RAIDER your doctor will discuss all your alternative options with you.

What are the possible benefits of taking part?

Everyone in the study will receive scans regularly throughout treatment. This may make the radiotherapy more accurate than if it was given without the scan. If you are in the adaptive radiotherapy groups you will receive radiotherapy treatment with the smallest possible safety margin each time and this may reduce the risk of side effects. If you are in the tumour focused boost group, the increased radiation dose to your tumour may control your bladder cancer better.

What are the possible disadvantages of taking part?

Tumour focused adaptive radiotherapy could cause an increased risk of missing the tumour, but everyone who gives treatment as part of RAIDER will be fully trained and treatment will be checked by a second trained observer before it is given. There is also a risk that sparing the bladder from exposure to full dose radiotherapy could cause an increased risk of the cancer returning elsewhere in the bladder, however previous studies have not suggested this, and any indication of this will be carefully monitored in all patients who join RAIDER.

It is possible that the side effects of tumour focused boost radiotherapy might be worse than with standard treatment, but this will be monitored for all RAIDER participants and the study will be stopped if people experience bad side effects.

The selection and confirmation of the radiotherapy treatment plan will extend the length of each radiotherapy treatment by about 5 to 10 minutes for patients receiving adaptive radiotherapy.

You may have more CT scans than you would if you did not take part in RAIDER because you will have one before each treatment, and if you are in the tumour focused groups you will have two planning scans rather than one. If you are in the tumour focused boost group you will receive a higher dose of radiotherapy than you would otherwise. All of these factors mean that you could be exposed to more radiation than you would be otherwise, which may lead to an increased risk of developing a second cancer later in life.

Before participating you should also consider if this will affect any insurance you have and seek advice if necessary.

How will confidentiality be maintained?

Your medical notes will be seen by authorised members of the research team at your hospital, so that they can collect information needed for the RAIDER study. When you join the study, your name, date of birth, postcode, hospital number and NHS or Community Health Index (CHI) number will be passed to the Institute of Cancer Research Clinical Trials and Statistics Unit(ICR-CTSU) where the study is being coordinated. You will be

For peer review only - http://bmjopen.bmj.com/site/about/guidelines.xhtml

given a unique registration number, which will be used together with your initials and date of birth on forms that the research staff will send to the ICR-CTSU. All information about you will be coded with this registration number and will be stored securely. It will be treated as strictly confidential and nothing that might identify you will be revealed to any third party.

Scientific employees of ICR-CTSU, and those conducting the study with them, including the national radiotherapy quality assurance team, may need to examine your medical records to ensure the study is being run properly and that the information collected on the forms is correct, but your confidentiality will be protected at all times.

We will contact your hospital over the years to find out how you are getting on. Ideally we would like to do this for life, but patients often change address or GP or lose touch with their hospital. If this happens we would like to use national records which are kept on everyone's health status to find out how you are. One of these is held at the General Register Office (GRO). We will need to give them enough information to identify you. This is usually your name, date of birth and NHS number (or Community Health Index and/or hospital number in Scotland). These details are confidential and will only be used for the purposes of the RAIDER study. Please initial the consent form to show that we have your permission to do this – if you do not agree, we will not seek this information.

The Institute of Cancer Research is the Sponsor for this study based in the United Kingdom. We will be using information from you and/or your medical records in order to undertake this study and will act as the data controller for this study. This means that we are responsible for looking after your information and using it properly. Our legal basis for processing your data is task in the public interest for scientific research purposes. The Institute of Cancer Research will keep identifiable information about you for 20 years after the study has finished.

Your rights to access, change or move your information are limited, as we need to manage your information in specific ways in order for the research to be reliable and accurate. If you withdraw from the study, we will keep the information about you that we have already obtained. To safeguard your rights, we will use the minimum personally-identifiable information possible.

You can find out more about how we use your information at www.icr.ac.uk/our-research/centres-and-collaborations/centres-at-the-icr/clinical-trials-and-statistics-unit/transparency.

[NHS site] will collect information from you and/or your medical records for this research study in accordance with our instructions.

[NHS site] will use your name, NHS number and contact details to contact you about the research study, and make sure that relevant information about the study is recorded for your care, and to oversee the quality of the study. Individuals from The Institute of Cancer Research and regulatory organisations may look at your medical and research records to check the accuracy of the research study. [NHS site] will pass these details to The Institute

For peer review only - http://bmjopen.bmj.com/site/about/guidelines.xhtml

of Cancer Research along with the information collected from you and/or your medical records. The only people in The Institute of Cancer Research who will have access to information that identifies you will be people who need to contact you to send a Quality of Life booklet by post or audit the data collection process. Only members of the research teams at your hospital and the ICR-CTSU will have access to the information that could allow this trial ID number to be linked to you.

[NHS site] will keep identifiable information about you from this study for 20 years after the study has finished.

Data sharing

When you agree to take part in a research study, the information about your health and care may be provided to researchers running other research studies in this organisation and in other organisations. These organisations may be universities, NHS organisations or companies involved in health and care research in this country or abroad. Your information will only be used by organisations and researchers to conduct research in accordance with the UK Policy Framework for Health and Social Care Research.

Your information could be used for research in any aspect of health or care, and could be combined with information about you from other sources held by researchers, the NHS or government.

Where this information could identify you, the information will be held securely with strict arrangements about who can access the information. The information will only be used for the purpose of health and care research, or to contact you about future opportunities to participate in research. It will not be used to make decisions about future services available to you, such as insurance.

Where there is a risk that you can be identified your data will only be used in research that has been independently reviewed by an ethics committee.

What will happen to the results of the research study?
Independent experts will review the safety and progress of the research whilst it is being carried out, and the results will be published in a respected medical journal once we are sure they are reliable. No information that could identify you will be included and you will not be identified in any report or publication.

We will summarise the results for participants once they are available. Your hospital will be able to give you a copy and results will also be available on Cancer Research UK's patient website (www.cancerhelp.org.uk).

What if relevant new information becomes available?
Sometimes we get new information about the treatment being studied. If this happens, your doctor will tell you and discuss whether you should continue in the study. If you decide not to carry on, your doctor will make arrangements for your care to continue.

For peer review only - http://bmjopen.bmj.com/site/about/guidelines.xhtml

Will I be paid for taking part in this study?

No. Neither you nor your doctor will be paid for taking part in this study.

What if there is a problem?

Any complaint about the way you have been dealt with during this study, or any possible harm you might suffer, will be addressed. Your progress will be watched closely and you will be offered whatever help is available to cope with any side effects. Occasionally some patients need a short stay in hospital for side effects to be treated, and on rare occasions these can be serious. If this were to happen, full details of what has happened will be reviewed carefully by the Doctor who has overall responsibility for the RAIDER study . It is unlikely that anything will go wrong with your treatment or care, but if you wish to complain about any aspect of the way you have been approached or treated during the course of the study you can do so using the normal NHS complaints procedure. Concerns should be raised by speaking to a member of staff at your hospital or by talking to the local Patient Advice and Liaison Service (PALS) which has been established in every NHS Trust.

NHS bodies are liable for clinical negligence and other negligent harm to individuals covered by their duty of care. In the event that something does go wrong and you are harmed during the research and this is due to someone‘s negligence then you may have grounds for a legal action for compensation against the NHS Trust but you may have to pay your legal costs. Alternative indemnity arrangements apply to private clinics.

What if I don’t want to carry on with the study?

You are free to withdraw from the study at any time. You do not have to give a reason and your future treatment and care will not be affected. If you change your mind about having treatment or follow up within this study, we would still like to collect information about how you are getting on. The information we need is routinely recorded in your medical records at your standard hospital visits and you would not need to do anything.

Who is organising and funding the research?

RAIDER is organised by doctors at the Royal Marsden Hospital in London, in collaboration with other leading doctors across the country and the Institute of Cancer Research Clinical Trials and Statistics Unit in London. The research is approved and funded by Cancer Research UK, who are providing funding to run the trial in the UK. The National Health Service Research and Development Executive will pay for any extra nursing and administrative costs incurred by participating hospitals and the National Institute for Health Research will pay for some of the costs of conducting the research at participating hospitals.

Who reviewed this study?

All research in the NHS is reviewed by an independent group of people, called a Research Ethics Committee, to protect participants’ safety, rights, wellbeing and dignity. RAIDER has been reviewed and approved by London – Surrey Borders Ethics Committee on behalf

For peer review only - http://bmjopen.bmj.com/site/about/guidelines.xhtml

of all hospitals throughout the UK. It has also been reviewed and approved by Cancer Research UK and reviewed and endorsed by patient and carer representatives from the NCRI Consumer Liaison Group (www.ncri.org.uk).

What happens now?

You will have some time to think about the study and make your decision. Your doctor, nurse or radiographer will be happy to answer any questions. You may wish to discuss it with your family or friends. Once you have reached your decision please let your doctor or nurse know. You will be asked to sign a consent form and will be given a copy to keep together with this information sheet. Please keep this information sheet and copies of the signed consent form. Your GP will be told that you are taking part in the RAIDER study. If at any time you have any questions about the study you should contact your hospital consultant.

Further information

Macmillan Cancer support is a registered charity and helps with all the things that people affected by cancer want and need, from specialist health care and information to practical, emotional and financial support (www.macmillan.org.uk).You can also learn more about clinical trials on the Cancer Research UK's patient website (www.cancerhelp.org.uk).

Contact details

If at any time you have any questions about the study please contact your local study team:

Local consultant's name:

Local research nurse/radiographer:

Address:

Telephone:

24 hour contact number:

Thank you for your interest in our research.

For peer review only

For peer review only - http://bmjopen.bmj.com/site/about/guidelines.xhtml

Optional components to the study:

If you agree to participate in the main RAIDER trial, you will be invited to take part in the following sub-studies:

1. Side effects questionnaire study
2. Donation of routine samples from surgery

If I want to be part of the RAIDER study, do I have to take part in the sub-studies?

No. Taking part in RAIDER does not mean you have to take part in the sub-studies. You will be given the chance to discuss RAIDER and you can then decide whether you want to take part.

The following pages of this information sheet give further details about these sub-studies.

1. Side effects questionnaire study

The main reason we are carrying out the RAIDER study is to look at the side effects of the radiotherapy treatment. If you decide to take part in RAIDER, we would like you to complete short questionnaires to describe any side effects that you may experience.

This is an optional part of the study but completed questionnaires will help us to understand more about the side effects of this radiotherapy treatment from your point of view. Completing a questionnaire should take no longer than 20 minutes.

If you agree to take part, we will ask you to fill in a questionnaire before you start radiotherapy, at the end of your radiotherapy treatment and then at 3, 6, 12, 18 and 24 months afterwards. We know from other patients that they feel such surveys are very important, but you do not have to complete them if you do not want to.

The first questionnaires will be given to you by your hospital. From 6 months onwards questionnaires will be posted to you at your home address by The Institute of Cancer Research Clinical Trials & Statistics Unit (ICR-CTSU).

Before sending you the questionnaire, ICR-CTSU will contact your hospital or GP to check how you are; therefore we would like to ask for your permission to give ICR-CTSU your full name and address as well as your GP's name and address. If you agree to this, please initial the consent form to show that we have your permission.

For peer review only - http://bmjopen.bmj.com/site/about/guidelines.xhtml

2. Donating samples from surgery

RAIDER gives us the opportunity to ask many people with cancer similar to yours whether we can collect samples that will aid future research.

When you were diagnosed bladder cancer, your hospital will have kept a sample of the tumour your surgeon removed during surgery. We would like your permission to collect this and samples which are taken at any future surgery you may have, so that we can look at it in combination with samples from other people who have joined the study. This will allow us to test for genetic differences in the make-up of individuals, indicate why they develop cancer and predict how they react to treatment. If we show that genetic differences do explain why some patients develop bladder cancer or react to their treatment differently, this knowledge could help many patients in the future. Any genetic analysis would be for research purposes only and will not affect any insurance you may hold.

When they are collected from your hospital, the samples will be coded and your personal details will be removed. The coding will maintain your confidentiality whilst allowing biological details to be compared to treatment findings.

The samples collected in this study will be stored indefinitely. It is possible that in the future other research may be carried out on the samples collected within this trial. This research may be conducted in the UK or overseas. Your personal details will not be shared with other researchers. Any future research on samples will be approved by an ethics committee before it is done.

Sample donation is entirely optional and you do not have to participate if you do not wish to. If you chose to join the study there will be a section to complete when you sign the study consent form to indicate if you agree to donate these samples.

For peer review only - http://bmjopen.bmj.com/site/about/guidelines.xhtml

Summary of study assessments

when→ / what↓	10 weeks after start of radiotherapy	3 months*	6 months*	9 months*	12 months*	18 months*	2 years	3 years	4 years	5 years
Assessment of side effects	✓	✓	✓	✓	✓	✓	✓	✓	✓	✓
Blood sample		✓	✓							
Cystoscopy under general anæsthetic with biopsy of tumour site		✓								
Cytoscopy under local anæsthetic			✓	✓	✓	✓	✓	✓	✓	✓
Chest x-ray		✓								
CT scan of abdomen & pelvis			✓		✓		✓			
Chest x-ray or CT scan			✓		✓	✓	✓	✓	✓	✓

*after the end of radiotherapy

For peer review only

For peer review only - http://bmjopen.bmj.com/site/about/guidelines.xhtml

(Form to be printed on hospital's headed paper)

CONSENT FORM

RAIDER:

A Randomised phase II trial of Adaptive Image guided standard or Dose Escalated tumour boost Radiotherapy in the treatment of transitional cell carcinoma of the bladder

Ethics Committee Reference: 15/LO/0539

RAIDER trial ID:

Name of Researcher taking consent:

Please write your initials in the box to the right of each statement if you agree, and please sign at the bottom

		**Initials**
1.	I confirm that I have read and understand the RAIDER Patient Information Sheet 32f Version 2.0 dated 23/01/2019 for the above study. I have had the opportunity to consider the information, ask questions and have had these answered satisfactorily.	
2.	I understand that my participation is voluntary and that I am free to withdraw at any time without giving any reason, without my medical care or legal rights being affected.	
3.	If I withdraw from the study, I consent to my doctor providing authorised researchers with basic clinical information that would be routinely collected and written in my medical records.	
4.	I understand that sections of any of my medical notes may be looked at by responsible individuals from the research team, from ethics committees, or from the NHS Trust, where it is relevant to my taking part in research. I give permission for these individuals to have access to my records.	
5.	I consent to the Institute of Cancer Research using information held by the NHS and national databases to follow up my health status.	
6.	I agree to my GP being informed of my participation in the study.	
7.	*Data sharing: I grant advance authorisation for the possible future sharing of information collected about me with other organisations, with the understanding that I will not be identifiable from this information (optional).*	

RAIDER consent 32f Version 2.0 23/01/2019
2 pages

For peer review only

8. *I agree to participate in the side effects questionnaire study. (optional)* ☐

8a. *I consent to researchers from The Institute of Cancer Research being sent my address and GP contact details.* ☐

8b. *I consent to researchers from The Institute of Cancer Research contacting my GP to confirm I am fit and well to receive questionnaire booklets to be sent out by post.* ☐

8c. *I consent to my GP disclosing my health status to researchers from The Institute of Cancer Research* ☐

9. *I consent to donating routinely collected samples from surgery (optional)* ☐

9a. *I agree that my tumour tissue samples will be analysed for potential changes in DNA (genetic changes)* ☐

9b. *I grant advance authorisation for the possible future sharing of information collected about me with other organisations, with the understanding that I will not be identifiable from this information* ☐

9c. *I grant advance authorisation for possible future research on my stored samples, with the understanding that I will not be identifiable from these samples. I understand that that approval of an ethics committee will be obtained beforehand.* ☐

10. I agree to take part in RAIDER ☐

For peer review only

Name of participant	Date	Signature

Name of person taking consent	Date	Signature

1 copy for participant; 1 copy for Principal Investigator; 1 copy to be kept with hospital notes

For peer review only - http://bmjopen.bmj.com/site/about/guidelines.xhtml

STANDARD PROTOCOL ITEMS: RECOMMENDATIONS FOR INTERVENTIONAL TRIALS

SPIRIT 2013 Checklist: Recommended items to address in a clinical trial protocol and related documents*

Section/item	Item No	Description	Addressed on page number
**Administrative information**			
Title	1	Descriptive title identifying the study design, population, interventions, and, if applicable, trial acronym	p1 manuscript (p1 protocol)
Trial registration	2a	Trial identifier and registry name. If not yet registered, name of intended registry	p7 manuscript (p1 protocol)
	2b	All items from the World Health Organization Trial Registration Data Set	n/a
Protocol version	3	Date and version identifier	V 3.0 dated 23/1/2019 (supplementary material)
Funding	4	Sources and types of financial, material, and other support	p30 manuscript (p36 protocol)
Roles and responsibilities	5a	Names, affiliations, and roles of protocol contributors	p1 manuscript
	5b	Name and contact information for the trial sponsor	p1 manuscript (p1 protocol)
	5c	Role of study sponsor and funders, if any, in study design; collection, management, analysis, and interpretation of data; writing of the report; and the decision to submit the report for publication, including whether they will have ultimate authority over any of these activities	p33-34, 36 protocol

For peer review only

For peer review only - http://bmjopen.bmj.com/site/about/guidelines.xhtml

	5d	Composition, roles, and responsibilities of the coordinating centre, steering committee, endpoint adjudication committee, data management team, and other individuals or groups overseeing the trial, if applicable (see Item 21a for data monitoring committee)	p21 manuscript (p33-34, 36 protocol)
**Introduction**			
Background and rationale	6a	Description of research question and justification for undertaking the trial, including summary of relevant studies (published and unpublished) examining benefits and harms for each intervention	p5-6 manuscript (p10-13 protocol)
	6b	Explanation for choice of comparators	p11 protocol
Objectives	7	Specific objectives or hypotheses	p6 manuscript (p13 protocol)
Trial design	8	Description of trial design including type of trial (eg, parallel group, crossover, factorial, single group), allocation ratio, and framework (eg, superiority, equivalence, noninferiority, exploratory)	p7 manuscript (p14 protocol)
**Methods: Participants, interventions, and outcomes**			
Study setting	9	Description of study settings (eg, community clinic, academic hospital) and list of countries where data will be collected. Reference to where list of study sites can be obtained	p15 protocol
Eligibility criteria	10	Inclusion and exclusion criteria for participants. If applicable, eligibility criteria for study centres and individuals who will perform the interventions (eg, surgeons, psychotherapists)	p8 manuscript (p15 protocol)
Interventions	11a	Interventions for each group with sufficient detail to allow replication, including how and when they will be administered	p8-17 manuscript (p23-24 protocol and radiotherapy planning and delivery protocol)
	11b	Criteria for discontinuing or modifying allocated interventions for a given trial participant (eg, drug dose change in response to harms, participant request, or improving/worsening disease)	p15 manuscript (p20, 30 protocol)

For peer review only - http://bmjopen.bmj.com/site/about/guidelines.xhtml

	11c	Strategies to improve adherence to intervention protocols, and any procedures for monitoring adherence (eg, drug tablet return, laboratory tests)	p10, 17-18 manuscript (p25, 32 protocol and additional radiotherapy planning and delivery protocol)
	11d	Relevant concomitant care and interventions that are permitted or prohibited during the trial	p7 manuscript; 24 protocol
Outcomes	12	Primary, secondary, and other outcomes, including the specific measurement variable (eg, systolic blood pressure), analysis metric (eg, change from baseline, final value, time to event), method of aggregation (eg, median, proportion), and time point for each outcome. Explanation of the clinical relevance of chosen efficacy and harm outcomes is strongly recommended	p7 manuscript (p14-15 protocol)
Participant timeline	13	Time schedule of enrolment, interventions (including any run-ins and washouts), assessments, and visits for participants. A schematic diagram is highly recommended (see Figure)	Table 1 manuscript (Table p22 of protocol)
Sample size	14	Estimated number of participants needed to achieve study objectives and how it was determined, including clinical and statistical assumptions supporting any sample size calculations	p19-20 manuscript (p28 protocol)
Recruitment	15	Strategies for achieving adequate participant enrolment to reach target sample size	p29-30 protocol

Methods: Assignment of interventions (for controlled trials)

Allocation:

Sequence generation	16a	Method of generating the allocation sequence (eg, computer-generated random numbers), and list of any factors for stratification. To reduce predictability of a random sequence, details of any planned restriction (eg, blocking) should be provided in a separate document that is unavailable to those who enrol participants or assign interventions	p16, 25 protocol

Allocation concealment mechanism	16b	Mechanism of implementing the allocation sequence (eg, central telephone; sequentially numbered, opaque, sealed envelopes), describing any steps to conceal the sequence until interventions are assigned	p17,44 protocol
Implementation	16c	Who will generate the allocation sequence, who will enrol participants, and who will assign participants to interventions	p17, 44 protocol
Blinding (masking)	17a	Who will be blinded after assignment to interventions (eg, trial participants, care providers, outcome assessors, data analysts), and how	n/a
	17b	If blinded, circumstances under which unblinding is permissible, and procedure for revealing a participant's allocated intervention during the trial	n/a
**Methods: Data collection, management, and analysis**			
Data collection methods	18a	Plans for assessment and collection of outcome, baseline, and other trial data, including any related processes to promote data quality (eg, duplicate measurements, training of assessors) and a description of study instruments (eg, questionnaires, laboratory tests) along with their reliability and validity, if known. Reference to where data collection forms can be found, if not in the protocol	p34, 36, 40, 47 protocol
	18b	Plans to promote participant retention and complete follow-up, including list of any outcome data to be collected for participants who discontinue or deviate from intervention protocols	p20 protocol
Data management	19	Plans for data entry, coding, security, and storage, including any related processes to promote data quality (eg, double data entry; range checks for data values). Reference to where details of data management procedures can be found, if not in the protocol	p34, 37 protocol
Statistical methods	20a	Statistical methods for analysing primary and secondary outcomes. Reference to where other details of the statistical analysis plan can be found, if not in the protocol	p19-21 manuscript (p31-32 protocol)
	20b	Methods for any additional analyses (eg, subgroup and adjusted analyses)	p19-21 manuscript (p31-32 protocol)
	20c	Definition of analysis population relating to protocol non-adherence (eg, as randomised analysis), and any statistical methods to handle missing data (eg, multiple imputation)	p19 manuscript (p29, 32, 34-35 40-41, 47 protocol)

**Methods: Monitoring**			
Data monitoring	21a	Composition of data monitoring committee (DMC); summary of its role and reporting structure; statement of whether it is independent from the sponsor and competing interests; and reference to where further details about its charter can be found, if not in the protocol. Alternatively, an explanation of why a DMC is not needed	p22 manuscript (p33 protocol)
	21b	Description of any interim analyses and stopping guidelines, including who will have access to these interim results and make the final decision to terminate the trial	p19-20 manuscript (p32-33 protocol)
Harms	22	Plans for collecting, assessing, reporting, and managing solicited and spontaneously reported adverse events and other unintended effects of trial interventions or trial conduct	p21 manuscript (p25-28 protocol)
Auditing	23	Frequency and procedures for auditing trial conduct, if any, and whether the process will be independent from investigators and the sponsor	n/a
**Ethics and dissemination**			
Research ethics approval	24	Plans for seeking research ethics committee/institutional review board (REC/IRB) approval	p21 manuscript (p1, p35 protocol)
Protocol amendments	25	Plans for communicating important protocol modifications (eg, changes to eligibility criteria, outcomes, analyses) to relevant parties (eg, investigators, REC/IRBs, trial participants, trial registries, journals, regulators)	p2 protocol
Consent or assent	26a	Who will obtain informed consent or assent from potential trial participants or authorised surrogates, and how (see Item 32)	p16, 35 protocol
	26b	Additional consent provisions for collection and use of participant data and biological specimens in ancillary studies, if applicable	p36-37 protocol
Confidentiality	27	How personal information about potential and enrolled participants will be collected, shared, and maintained in order to protect confidentiality before, during, and after the trial	p35-36 protocol
Declaration of interests	28	Financial and other competing interests for principal investigators for the overall trial and each study site	p30 manuscript

For peer review only

Access to data	29	Statement of who will have access to the final trial dataset, and disclosure of contractual agreements that limit such access for investigators	p30 manuscript
Ancillary and post-trial care	30	Provisions, if any, for ancillary and post-trial care, and for compensation to those who suffer harm from trial participation	p36 protocol
Dissemination policy	31a	Plans for investigators and sponsor to communicate trial results to participants, healthcare professionals, the public, and other relevant groups (eg, via publication, reporting in results databases, or other data sharing arrangements), including any publication restrictions	p36 protocol
	31b	Authorship eligibility guidelines and any intended use of professional writers	p36 protocol
	31c	Plans, if any, for granting public access to the full protocol, participant-level dataset, and statistical code	n/a
**Appendices**			
Informed consent materials	32	Model consent form and other related documentation given to participants and authorised surrogates	Included as supplementary
Biological specimens	33	Plans for collection, laboratory evaluation, and storage of biological specimens for genetic or molecular analysis in the current trial and for future use in ancillary studies, if applicable	p36 protocol

*It is strongly recommended that this checklist be read in conjunction with the SPIRIT 2013 Explanation & Elaboration for important clarification on the items. Amendments to the protocol should be tracked and dated. The SPIRIT checklist is copyrighted by the SPIRIT Group under the Creative Commons "Attribution-NonCommercial-NoDerivs 3.0 Unported" license.

For peer review only
